# Ligand-enabled Ni-catalysed enantioconvergent intermolecular Alkyl-Alkyl cross-coupling between distinct Alkyl halides

**DOI:** 10.1038/s41467-023-38702-3

**Published:** 2023-05-22

**Authors:** Wen-Tao Zhao, Jian-Xin Zhang, Bi-Hong Chen, Wei Shu

**Affiliations:** 1 Shenzhen Grubbs Institute and Department of Chemistry, Southern University of Science and Technology, Shenzhen, 518055 Guangdong P. R. China; 2 State Key Laboratory of Elemento-Organic Chemistry, Nankai University, Tianjin, 300071 P. R. China

**Keywords:** Asymmetric catalysis, Synthetic chemistry methodology, Stereochemistry

## Abstract

α-Tertiary aliphatic amides are key elements in organic molecules, which are abundantly present in natural products, pharmaceuticals, agrochemicals, and functional organic materials. Enantioconvergent alkyl-alkyl bond-forming process is one of the most straightforward and efficient, yet highly challenging ways to build such stereogenic carbon centers. Herein, we report an enantioselective alkyl-alkyl cross-coupling between two different alkyl electrophiles to access α-tertiary aliphatic amides. With a newly-developed chiral tridentate ligand, two distinct alkyl halides were successfully cross-coupled together to forge an alkyl-alkyl bond enantioselectively under reductive conditions. Mechanistic investigations reveal that one alkyl halides exclusively undergo oxidative addition with nickel versus in-situ formation of alkyl zinc reagents from the other alkyl halides, rendering formal reductive alkyl-alkyl cross-coupling from easily available alkyl electrophiles without preformation of organometallic reagents.

## Abstract (web summary)

Accessing saturated stereogenic centers by asymmetric alkyl-alkyl formation is attractive yet challenging. Here, the authors develop a Ni-catalysed cross-coupling reaction of two alkyl halides enabled by the in-situ formation of alkyl zinc from one alkyl halide.

## Introduction

**Fig. 1:**
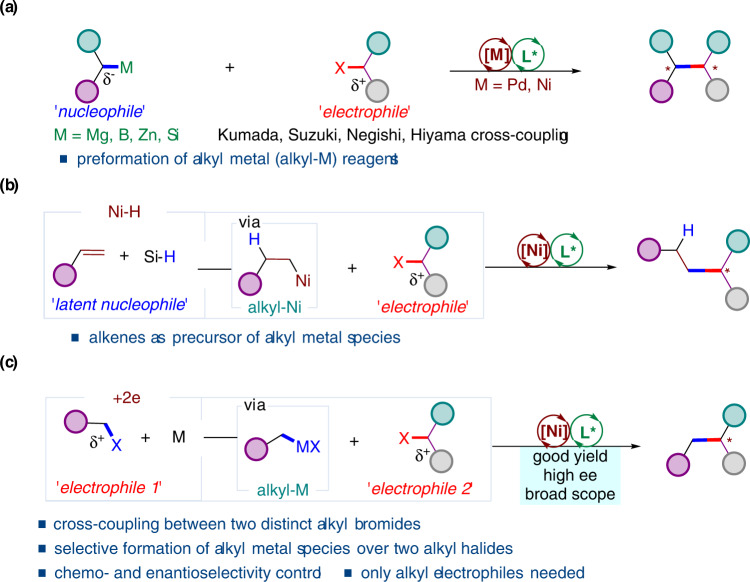
Strategies for intermolecular enantioselective construction of alkyl-alkyl bonds. **a** Enantioselective C_sp3_-C_sp3_ bond-forming from alkyl-M and alkyl electrophiles. **b** Enantioselective C_sp3_-C_sp3_ bond-forming from alkenes and alkyl electrophiles. **c** Enantioselective C_sp3_-C_sp3_ bond-forming from two alkyl electrophiles.

α-Tertiary aliphatic amides with a α-saturated stereogenic carbon center are key structural units in chemistry, functional materials and many related areas^1–5^. Thus, the development of versatile and straightforward methods to access saturated stereogenic centers in a highly enantioenriched manner has been attracting long-term interests from chemistry community^6^. Early efforts have been paid to the employing of chiral auxiliaries to control the desired stereochemistry, resulting in the use of stoichiometric amount of chiral auxiliaries as well as additional steps for their installation and removal from the target molecules^7^. Over the past decades, studies have been increasingly focused on catalytic approaches to access such stereogenic centers^8,9^, including Ni-catalysed enantioconvergent cross-coupling between an alkyl electrophile and an alkyl nucleophile (Fig. 1a)^10,11^. Over the past years, significant progress has been achieved in nickel-catalysed enantioselective cross-coupling of racemic secondary alkyl electrophiles with organometallic reagents^12–20^. This reaction mode has been well-developed and evolved into an inevitable tool for constructing saturated stereogenic carbon centers. Although the significant advances, this reaction mode requires stoichiometric, reactive, and often sensitive organometallic reagents, which usually require time-consuming preformation. To this end, one alternative is to use alkenes as masked alkyl nucleophiles in the presence of metal hydride to undergo enantioselective alkyl-alkyl cross-coupling^21–24^. Hydrometallation of alkenes through metal hydride insertion generates alkyl metallic intermediates in situ as alkyl nucleophiles. In 2019, Fu group reported a seminal work on Ni-H catalysed enantioselective alkyl-alkyl cross-couplings of 1-substituted alkenes as a surrogate of carbon nucleophile to couple with secondary alkyl bromides adjacent to amides and esters (Fig. 1b)^25,26^. More recently, secondary alkyl bromides next to phosphates and ethers were successfully involved^27–33^. Accordingly, this strategy has evolved into an efficient cross-coupling of diverse alkenes with alkyl electrophiles to build saturated stereogenic carbon centers in the presence of metal hydrides^34,35^.

However, direct reductive cross-coupling between two distinct electrophiles is still one of the most straightforward, cost-effective, thus ideal alternatives to construct saturated stereogenic carbon centers^36–38^. Ni-catalysed cross-coupling reactions between organ-electrophiles under reductive conditions have been extensively investigated for C_*sp2*_-C_*sp3*_ bond formation^39–44^. To date, no example of non-enzyme-catalysed enantioselective C_*sp3*_-C_*sp3*_ bond formation was reported^45^. Herein, we report a Ni-catalysed intermolecular cross-coupling between two different alkyl electrophiles under reductive conditions (Fig. 1c). The use of newly-developed chiral ligand enables construction of C_*sp3*_-C_*sp3*_ bond by selective coupling of two distinct alkyl electrophiles, without the preformation of organometallic reagents.

## Results

### Optimization of the reaction conditions

To prove the concept, we commenced the investigation using **1a** and **2a** as the prototype substrates using nickel catalysis to evaluate the reaction parameters. After extensive preliminary evaluation (See Supplementary Tables 1–5), we found the use of pyridine-BOX type ligands gave better results compared to other types of ligands in the presence of zinc (3.0 equiv) as sacrificing reductant, ferrous chloride (25 mol%), and cesium iodide (3.0 equiv) as additives. Among the tested known ligands, **L1** gave the best result, delivering the desired cross-electrophile coupling product **3a** in 67% yield with 70% ee (Table 1, entry 1). Modifying the substituents at α-position to oxygen on the oxazolidine ring of *i*Pr-PyBOX significantly altered the efficiency of the ligand for this reaction (Table 1, entries 2–7). Linear substituents at α-position to oxygen of *i*Pr-PyBOX (**L2**-**L4**) substantially diminished the yield and enantioselectivity (Table 1, entries 2–4). Cyclic substituents slightly increased the enantioselectivity of **3a** from 70% to 76% and 71%, respectively (Table 1, entries 5 and 6). Introducing two phenyl groups onto α-position to oxygen (**L7**) led to trace amount of **3a** (Table 1, entry 7). Next, Bn-PyBOX derived ligands (**L8**-**L12**) were applied to this asymmetric cross-electrophile coupling reaction (Table 1, entries 8-12). Bn-PyBOX delivered the desired product **3a** in 29% yield with 60% ee (Table 1, entry 8). Increasing the steric hindrance at the α-position to oxygen improved the enantiomeric excess of **3a** to 84% (Table 1, entry 10). Ligands derived from *i*Bu-PyBOX (**L13**-**L15**) gave inferior yields and enantioselectivity (Table 1, entries 13-15). When Et-PyBOX based ligand **L16** was used, **3a** was obtained in 29% yield with 90% ee (Table 1, entry 16). Then, Me-PyBOX derived ligands (**L17**-**L23**) were tested (Table 1, entries 17-23). The use of propyl Me-PyBOX (**L20**) furnished **3a** in 21% yield with 94% ee (Table 1, entry 20). Further evaluation of additive and solvent effect revealed that the addition of 15-crown-5 (10 mol%) in a mixture of DMA and diglyme (1:3) afforded **3a** in 85% yield with 94% ee (See Supplementary Tables, 7-11). The use of ferrous chloride may facilitate the cross-coupling of **1a** with **2a**. In addition, the addition of 15-crown-5 may serve as an additive to enhance the solubility of inorganic salts in organic phase.

**Table 1:** Ligand effect on the enantioselective formal reductive alkyl-alkyl cross-coupling reaction^*a*^


Entry	L	Yield (%)	ee (%)	Entry	L	Yield (%)	ee (%)	Entry	L	Yield (%)	ee (%)
1	**L1**	67	70	9	**L9**	24	72	17	**L17**	8	50
2	**L2**	15	36	10	**L10**	49	84	18	**L18**	44	86
3	**L3**	16	38	11	**L11**	30	83	19	**L19**	45	91
4	**L4**	27	42	12	**L12**	22	62	20	**L20**	21	94
5	**L5**	39	76	13	**L13**	31	60	21	**L21**	18	91
6	**L6**	30	71	14	**L14**	26	68	22	**L22**	10	78
7	**L7**	trace	—	15	**L15**	18	62	23	**L23**	23	87
8	**L8**	29	60	16	**L16**	29	90	24^b^	**L20**	85 (76)^c^	94

*DME* dimethoxyethane, *DMA* dimethylacetamide.

^a^The reaction was conducted using **1a** (0.2 mmol), **2a** (0.6 mmol) under indicated conditions for 24 h. Yield was determined by GC using *n*-dodecane as internal standard. The enantiomeric excess was determined by HPLC using a chiral column with stationary chiral phase.

^b^Reaction conditions: The reaction was run using **1a** (0.2 mmol), **2a** (0.6 mmol) with NiCl2‧DME (8 mol%), **L20** (8 mol%), FeCl2 (25 mol%), Zn (2.0 equiv), CsI (3.0 equiv), 15-crown-5 (10 mol%) in DMA/diglyme = 1:3 (0.1 M) at room temperature for 24 h.

^c^Isolated yield after flash chromatography.

### Scope of the reaction

**Fig. 2:**
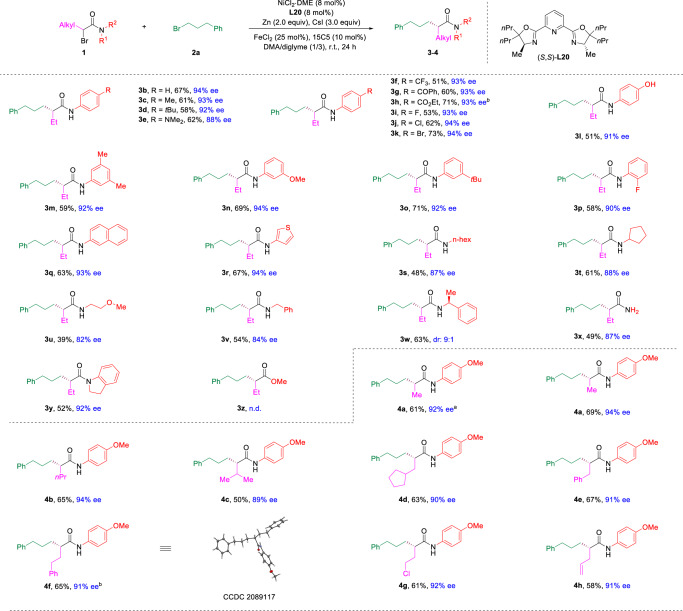
Scope of *α*-bromoamides. The reaction was performed on a 0.2 mmol scale under the conditions in Table 1, entry 24. 15C5 = 15-crown-5. DME dimethoxyethane, DMA dimethylacetamide. Note: ^a^*α*-Chloroamide was used instead of *α*-bromoamide. ^b^3-Phenyl-1-iodopropane was used instead of **2a**.

**Fig. 3:**
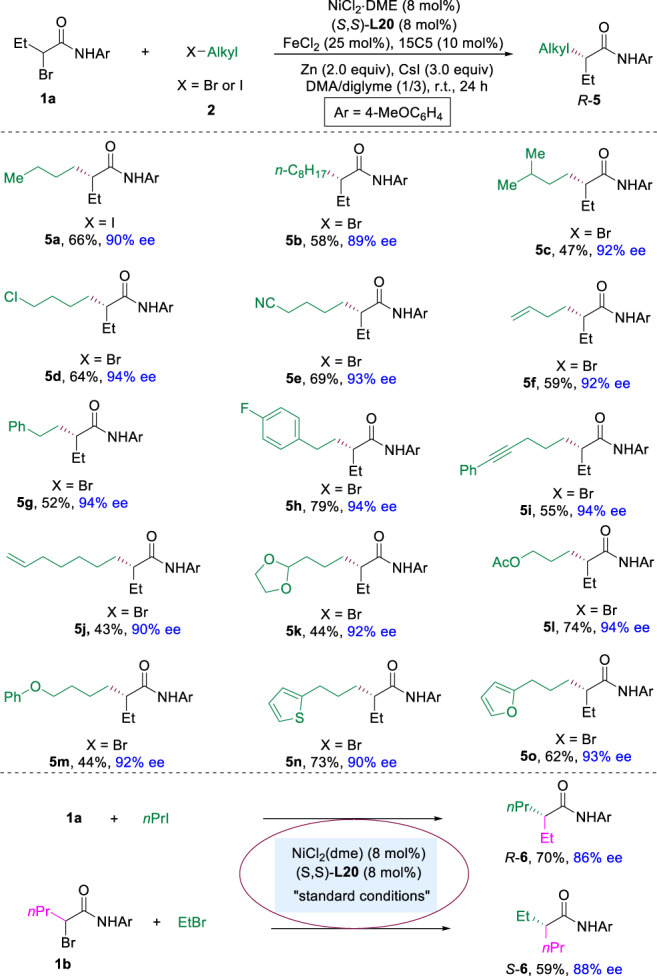
Scope of alkyl halides. The reaction was performed on 0.2 mmol scale under the conditions in Table 1, entry 24. 15C5 = 15-crown-5. DME dimethoxyethane, DMA dimethylacetamide.

With the optimized conditions in hand, we turned to test the scope of this reaction (Figs. 2 and 3). First, we examined the viability of *α*-bromoamides **1** (Fig. 2). Various substituted aniline derived *α*-bromoamides were good substrates for this enantioselective cross-electrophile alkyl-alkyl coupling reaction (**3b**-**3r**). Electron-donating group substituted aniline based amides delivered the desired enantioenriched *α*-alkylated amides in 58%–67% yields with 88–94% ee (**3b**-**3e**). Electron-withdrawing groups were also tolerated in the reaction, delivering the corresponding *α*-tertiary amides in 51–73% yields with 93–94% ee (**3f**-**3k**). Ketones and esters were compatible under the reaction conditions, giving the ketone and ester containing *α*-tertiary amides in 60% and 71% yields with 93% ee (**3g**-**3h**). Halides, such as fluorine, chlorine, and bromine, were also well-tolerated in this nickel-catalysed reductive process (**3i**-**3k**), leaving halides as a chemical handle for further elaboration. Notably, free phenol was tolerated in the catalytic process, furnishing desired enantioenriched amide **3l** in 51% yield with 91% ee. Moreover, *meta-* and *ortho-*substituted as well as 2-naphthylamine derived α-bromoamides could be converted to corresponding α-alkylated amides in 58–71% yields with 92–94% ee (**3m-3q**). Thiophene amine was tolerated in the reaction, giving the desired product **3r** in 67% yield with 94% ee. Aliphatic amines, including the linear, branched, and benzylic amine based α-bromoamides were all good substrates, giving the desired products in synthetic useful yields with 82–88% ee (**3s-3v**). α-Bromoamide from chiral amine was converted to α-alkylated amide **3w** in 63% yield with 9:1 dr. Impressively, unprotected α-bromoamides, which are challenging for enantioselective coupling reactions, could be tolerated to deliver corresponding reductive cross alkyl-alkyl coupling product **3x** in 49% yield with 87% ee. In addition, α-bromo-*N*,*N*-disubstituted amide is applicable in this reaction, affording the cross-coupling product (**3y**) in 52% yield with 92% ee. Unfortunately, α-bromo ester failed to deliver the desired cross-coupling product (**3z**) under the reaction conditions. Next, we embarked to test the scope of α-substituent of amides. Diverse alkyl substituents with different chain length were good substrates, delivering corresponding α-alkylated amides (**4a** and **4b**) in 69% and 65% yields with 94% ee, respectively. Notably, α-chloroamides successfully underwent asymmetric alkyl-alkyl cross-coupling with **2a** to give **4a** in 61% yield with 92% ee. More steric demanding substituents, such as isopropyl, cyclopentylmethyl were also compatible in the reaction, giving **4c** and **4d** in 50% and 63% yields with 89% and 90% ee. Benzyl, phenylethyl, chloroethyl, and allyl could be tolerated in the reaction, giving the desired products **4e-4h** in 58%-67% yields with 91%-92% ee. Notably, the enantioenriched amides with similar steric hindrance at α-position could be achieved with excellent enantioselectivity (**4f**). The absolute configuration of the product was further confirmed by the X-ray diffraction analysis of **4f**.

Next, the scope of the other alkyl electrophile was evaluated (Fig. 3). Different alkyl bromides were good substrates for this enantioselective cross-electrophile coupling reaction, giving corresponding alkyl-alkyl products (**5a**-**5c**) in 47–66% yields with 89–92% ee. Many functional groups, such as chlorine, nitrile, amide, alkene, alkyne, acetal, ester, ether containing alkyl bromides could be coupled to deliver desired products in moderate to good yields with 90–94% ee (**5d**-**5m**). Heterocycles, such as thiophene and furan substituted alkyl halides were transformed into corresponding products (**5n** and **5o**) in 65% and 62% yields with 90% and 93% ee, respectively. Unfortunately, secondary unactivated alkyl halides remain unsuccessful for the reaction. In addition, both isomers of **6** were obtained under identical reaction conditions with the same chiral ligand. In the presence of (*S*,*S*)-**L20**, the reaction of **1a** with 1-iodopropane gave *R*-**6** in 70% yield with 86% ee, while the reaction of **1b** with ethyl bromide furnished the other isomer *S*-**6** in 59% yield with 88% ee.

### Mechanistic study

**Fig. 4:**
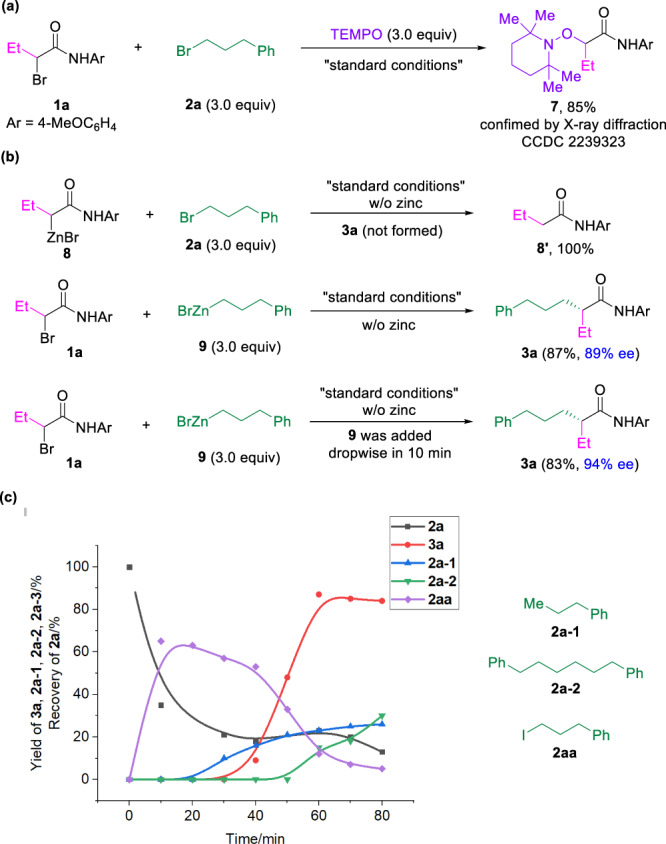
Control experiments and mechanistic investigations. **a** Quench of radical intermediates. TEMPO tetramethylpiperidine oxide. **b** Reactions with alkyl zinc reagents. **c** Time course for **2a** and **3a** under standard conditions.

In order to gain insight into the mechanism of the reaction, we set up a series of reactions to shed light on the reaction pathways (Fig. 4). First, the reaction of **1a** with **2a** in the presence of a radical scavenger TEMPO under otherwise identical to standard conditions was conducted (Fig. 4a). The desired intermolecular cross-coupling product **3a** was not formed. Instead, the adduct **7** of TEMPO with **1a** was obtained in 85% yield, indicating α-bromoamides underwent a single electron transfer process in this transformation. Next, the reactions of alkyl bromides with preformed alkyl zinc reagents under the standard conditions were tested (Fig. 4b). When alkyl zinc reagent **8** was used instead of **1a** to couple with **2a** under standard conditions, no desired product **3a** was detected, and only protonated product **8’** was formed quantitatively, indicating alkyl zinc reagent **8** could not mediate the reaction under the standard reaction conditions. In contrast, the reaction of **1a** with alkyl zinc reagent **9** under standard conditions delivered the desired cross-coupling product **3a** in 87% yield with 89% ee. Further conducting the reaction with slow addition of alkyl zinc reagent **9** led to the formation of **3a** in 83% yield with 94% ee, which is identical to the standard reaction conditions. These results suggest slow formation of alkyl zinc intermediate **9** in-situ to serve as intermediate for the reaction. To further prove the formation of alkyl zinc intermediates during the reaction, a real-time reaction course was conducted (Fig. 4c). The monitor the reaction process of **1a** with **2a** under standard conditions showed that no formation of **3a** in the first 30 min, although the consumption of **2a** was observed, indicating the induction time to form significant amount of alkyl intermediate to initiate the coupling reaction to generate **3a**.

**Fig. 5:**
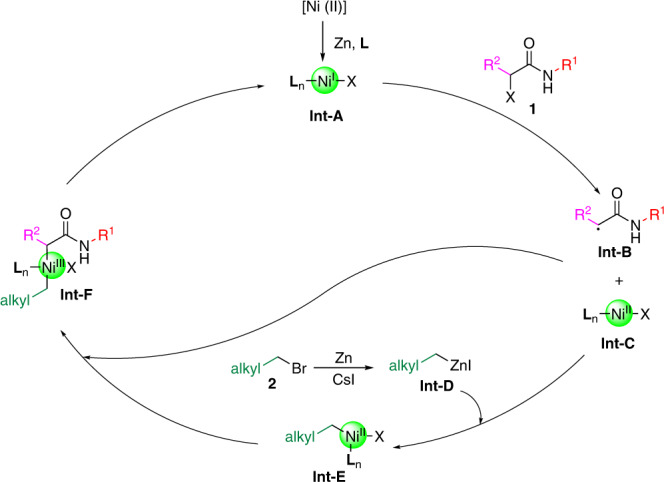
Proposed mechanism for the reaction. A plausible reaction mechanism for the intermolecular alkyl-alkyl cross-coupling between distinct alkyl halides based on all experimental results and previous literature evidence.

On the basis of experimental results and literature precedence^46–48^, a plausible mechanism is depicted in Fig. 5. First, Ni(II) was reduced by zinc to generate the ligated nickel (I) species (**Int-A**) in the presence of chiral ligand (**L**), which could undergo single electron transfer to **1** to give alkyl radical intermediate **Int-B** and Ni (II) intermediate **Int-C**. In the meantime, alkyl zinc reagent **Int-D** could be formed from **2** and zinc in the assistance with iodide, which could undergo transmetalation with **Int-C** to generate alkyl Ni(II) species **Int-E**. The rebound of intermediates **Int-B** and **Int-E** could form dialkyl Ni (III) intermediate **Int-F**, which would facilitate reductive elimination to furnish the final product **3** and regenerate Ni (I) species.

## Discussion

In summary, an intermolecular enantioselective alkyl-alkyl cross-coupling between two alkyl electrophiles has been developed enabled by the efficient and selective cross-coupling reaction between two distinct alkyl halides under reductive conditions, representing an alternative for the construction of chiral C_*sp3*_-C_*sp3*_ bonds. One alkyl halides in-situ formed alkyl nucleophiles with reducing metal to cross-couple with the other alkyl halides in a chemo- and enantioselective manner, circumventing the tedious and time-consuming preformation of alkyl metal species. We anticipate this will inspire enantioselective in-situ cross-coupling between alkyl electrophiles under reductive conditions to be evolved into one of the major strategies to build saturated carbon centers via enantioselective C_*sp3*_-C_*sp3*_ bond-formation.

## Methods

### General procedure for Ni-catalysed enantioconvergent intermolecular alkyl-alkyl cross-coupling

In a nitrogen-filled glovebox, NiCl_2_**·**glyme (0.016 mmol, 8 mol%), chiral ligand **L20** (0.016 mmol, 8 mol%) and diglyme (1.0 mL) were added to a 10-mL vial equipped with a stir bar. The mixture was allowed to stir for 1 h, after which it was an orange solution. Then, FeCl_2_ (0.05 mmol, 25 mol%), 15C5 (0.02 mmol, 10 mol%), CsI (0.6 mmol, 300 mol%), Zn (0.4 mmol, 200 mol%), **1** (0.2 mmol), **2** (0.6 mmol, 300 mol%), DMA (0.5 mL) and diglyme (0.5 mL) were added. The reaction mixture was transferred out of the glovebox and stirred (~1400 rpm) at room temperature for 24 h. Next, ethyl acetate (20.0 mL) was added, and the mixture was washed with water (10.0 mL) and brine (10.0 mL), dried over Na_2_SO_4_, filtered, and concentrated under vacuum. The residue was purified by flash chromatography on silica gel to afford the enantioselective alkyl-alkyl cross-coupling product.

## Supplementary information

Supplementary Information

## Data Availability

The X-ray crystallographic coordinates for structures that support the findings of this study have been deposited at the Cambridge Crystallographic Data Center (CCDC) with the accession code CCDC 2089117 (**4f**) and CCDC 2239323 (**7**) (www.ccdc.cam.ac.uk/data_request/cif). The authors declare that all other data supporting the findings of this study are available within the article and Supplementary Information files, and also are available from the corresponding author upon request.
