# Supplementary material for: Ligand-enabled Ni-catalysed enantioconvergent intermolecular Alkyl-Alkyl cross-coupling between distinct Alkyl halides
Source: Nat Commun. 2023 May 22;14:2938. doi: 10.1038/s41467-023-38702-3 (PMC10203360; doi:10.1038/s41467-023-38702-3)
Supplement: Supplementary file 1 — Supplementary Information [file 41467_2023_38702_MOESM1_ESM.pdf]

Supplementary Information

Ligand-Enabled Ni-Catalysed Enantioconvergent Intermolecular Alkyl-Alkyl Cross-Coupling between Distinct Alkyl Halides

Wen-Tao Zhao,[1] Jian-Xin Zhang,[1] Bi-Hong Chen[1] & Wei Shu[1,2,*]

[1]*Shenzhen Grubbs Institute and Department of Chemistry, Southern University of Science and Technology, Shenzhen 518055, Guangdong, P. R. China*

[2]*State Key Laboratory of Elemento-Organic Chemistry, Nankai University, Tianjin 300071, P. R. China*

*E-mail: shuw@sustech.edu.cn

Table of Contents

1. Supplementary Methods S2

2. Supplementary Notes..S3

2.1 Synthesis of 2-Bromoamides S3

2.2 Synthesis of Chiral Ligands...S19

2.3 Optimization of Reaction Parameters..S31

2.4 Enantioconvergent Reductive Alkyl-Alkyl Cross-Coupling S40

2.5 Mechanistic Experiments S74

2.6 HPLC of Racemic and Enantioenriched Products S89

2.7 ^{1}H-NMR and ^{13}C-NMR Spectra Data S141

2.8 X-Ray Diffraction Data of 4f and 7...S247

3. Supplementary References...S250

1. Supplementary Methods

Unless otherwise stated, reactions were performed under a nitrogen atmosphere using dried solvents. Tetrahydrofuran (THF), methylene chloride (CH_2Cl_2), and dimethylacetamide (DMA), were dried by calcium hydride (CaH). Diethyl ether (Et_2O), and diethylene glycol dimethyl ether (diglyme) were dried by sodium. Zinc powder was activated by hydrochloric acid (1 mol/L). Commercially available reagents were used without further purification. Thin layer chromatography (TLC) was performed using Jiangyou TLC silica gel plates HSG F254 and visualized using UV light or phosphomolybdic acid (PMA). Flash column chromatography was performed over silica gel (200-300 mesh). ^1H and ^{13}C NMR spectra were recorded in $CDCl_3$, unless otherwise noted, on a Bruker AVANCE 600 MHz or a Bruker AVANCE 400 MHz spectrometer. Chemical shifts in ^1H NMR spectra were reported in parts per million (ppm) on the δ scale from an internal standard of residual chloroform (7.26 ppm). Data for ^1H NMR spectra are reported as follows: chemical shift (δ ppm) (multiplicity, coupling constant (Hz), integration). Multiplicity and qualifier abbreviations are as follows: s = singlet, d = doublet, t = triplet, q = quartet, m = multiplet, br = broad. Data for ^{13}C NMR spectra were reported in terms of chemical shift in ppm from the central peak of $CDCl_3$ (77.0 ppm). HRMS experiments were performed on a Thermo Scientific Q Exactive. Analytical chiral HPLC was performed with an Agilent 1260 Series HPLC utilizing chiralpak® AS-H (4.6 mm x 25.0 cm) or chiralcel® OD-H columns (4.6 mm x 25.0 cm) obtained from Daicel Chemical Industries, Ltd with visualization at 254 nm or 210 nm.

2. Supplementary Notes

2.1 Synthesis of 2-Bromoamides

General procedure A: Triethylamine (30.0 mmol) and aniline (20.0 mmol) were stirred in DCM (50.0 mL) at 0 °C, 2-bromobutyric acid bromide (20.0 mmol) was added dropwise, and stirred for 0.5 h. The cooling bath was removed and the mixture was stirred overnight. The mixture was washed with water and extracted with DCM. Solvents were evaporated, and the product was purified by flash chromatography or recrystallization.

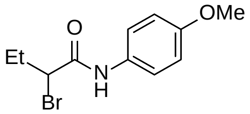

2-Bromo-*N*-(4-methoxyphenyl)butanamide (1a). The title compound was synthesized according to General procedure A from 2-bromobutanoyl bromide (4.6 g, 20.0 mmol), 4-methoxyaniline (2.5 g, 20.0 mmol). The product was purified by flash chromatography (20% ethyl acetate/hexanes). White solid. 4.6 g (85% yield).

^{1}H NMR (400 MHz, Chloroform-*d*) δ 8.10 (s, 1H), 7.51 – 7.38 (m, 2H), 6.94 – 6.78 (m, 2H), 4.41 (dd, *J* = 7.7, 5.2 Hz, 1H), 3.79 (s, 3H), 2.31 – 2.06 (m, 2H), 1.09 (t, *J* = 7.3 Hz, 3H).

^{13}C NMR (101 MHz, $CDCl_3$) δ 166.6, 156.9, 130.1, 122.0, 114.2, 55.5, 53.8, 29.4, 11.7.

HRMS (ESI) m/z $(M+H)^+$ calcd for $C_{11}H_{15}BrNO_2$: 272.0281, found: 272.0283.

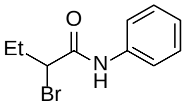

2-Bromo-*N*-phenylbutanamide (1b). The title compound was synthesized according to General procedure A from 2-bromobutanoyl bromide (4.6 g, 20.0 mmol), aniline (1.9 g, 20.0 mmol). The product was purified by flash chromatography (10% ethyl acetate/hexanes). White solid. 4.2 g (88% yield).

^{1}H NMR (400 MHz, Chloroform-*d*) δ 8.21 (s, 1H), 7.54 (d, *J* = 7.8 Hz, 2H), 7.34 (t, *J* = 7.9 Hz, 2H), 7.15 (t, *J* = 7.4 Hz, 1H), 4.41 (dd, *J* = 7.7, 5.3 Hz, 1H), 2.36 – 2.18 (m,

1H), 2.17 – 2.08 (m, 1H), 1.09 (t, *J* = 7.3 Hz, 3H).

^{13}C NMR (101 MHz, $CDCl_3$) δ 166.6, 137.1, 129.1, 125.0, 120.0, 53.9, 29.4, 11.8.

HRMS (ESI) m/z $(M+H)^+$ calcd for $C_{10}H_{13}BrNO$: 242.0175, found: 242.0175.

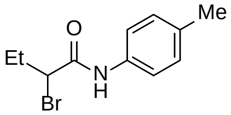

2-Bromo-*N*-(*p*-tolyl)butanamide (1c). The title compound was synthesized according to General procedure A from 2-bromobutanoyl bromide (2.3 g, 10.0 mmol), *p*-toluidine (1.1 g, 10.0 mmol). The product was purified by recrystallization (3% ethyl acetate/hexanes). White solid. 0.9 g (35% yield).

^{1}H NMR (600 MHz, Chloroform-*d*) δ 8.08 (s, 1H), 7.41 (d, *J* = 8.2 Hz, 2H), 7.15 (d, *J* = 8.1 Hz, 2H), 4.42 (dd, *J* = 7.6, 5.2 Hz, 1H), 2.32 (s, 3H), 2.27 – 2.21 (m, 1H), 2.18 – 2.11 (m, 1H), 1.10 (t, *J* = 7.3 Hz, 3H).

^{13}C NMR (151 MHz, $CDCl_3$) δ 166.5, 134.7, 134.5, 129.5, 120.1, 54.0, 29.4, 20.9, 11.7.

HRMS (ESI) m/z $(M+H)^+$ calcd for $C_{11}H_{15}BrNO$: 256.0332, found: 256.0333.

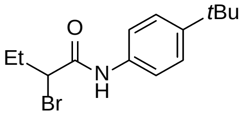

2-Bromo-*N*-(4-(*tert*-butyl)phenyl)butanamide (1d). The title compound was synthesized according to General procedure A from 2-bromobutanoyl bromide (2.3 g, 10.0 mmol), 4-(*tert*-butyl)aniline (1.5 g, 10.0 mmol). The product was purified by recrystallization (3% ethyl acetate/hexanes). White solid. 1.2 g (40% yield).

^{1}H NMR (600 MHz, Chloroform-*d*) δ 8.03 (s, 1H), 7.45 (d, *J* = 8.4 Hz, 2H), 7.37 (d, *J* = 8.4 Hz, 2H), 4.42 (dd, *J* = 7.4, 5.3 Hz, 1H), 2.29 – 2.21 (m, 1H), 2.18 – 2.11 (m, 1H), 1.31 (s, 9H), 1.10 (t, *J* = 7.2 Hz, 3H).

^{13}C NMR (151 MHz, $CDCl_3$) δ 166.5, 148.1, 134.5, 125.9, 119.8, 53.9, 34.4, 31.3, 29.4, 11.7.

HRMS (ESI) m/z $(M+H)^+$ calcd for $C_{14}H_{21}BrNO$: 298.0801, found: 298.0803.

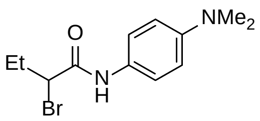

2-Bromo-*N*-(4-(dimethylamino)phenyl)butanamide (1e). The title compound was synthesized according to General procedure A from 2-bromobutanoyl bromide (2.3 g, 10.0 mmol), *N*[1],*N*[1]-dimethylbenzene-1,4-diamine (1.4 g, 10.0 mmol). The product was purified by recrystallization (3% ethyl acetate/hexanes). Gray solid. 2.1 g (74% yield).

^{1}H NMR (600 MHz, Chloroform-*d*) δ 8.01 (s, 1H), 7.42 – 7.32 (m, 2H), 6.71 (d, *J* = 9.0 Hz, 2H), 4.41 (dd, *J* = 7.7, 5.1 Hz, 1H), 2.93 (s, 6H), 2.27 – 2.20 (m, 1H), 2.16 – 2.11 (m, 1H), 1.09 (t, *J* = 7.3 Hz, 3H).

^{13}C NMR (151 MHz, $CDCl_3$) δ 166.3, 148.3, 126.8, 121.9, 112.9, 54.2, 40.8, 29.5, 11.7.

HRMS (ESI) m/z $(M+H)^+$ calcd for $C_{12}H_{18}BrN_2O$: 285.0597, found: 285.0600.

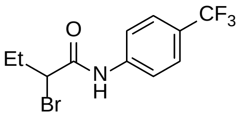

2-Bromo-*N*-(4-(trifluoromethyl)phenyl)butanamide (1f). The title compound was synthesized according to General procedure A from 2-bromobutanoyl bromide (2.3 g, 10.0 mmol), 4-(trifluoromethyl)aniline (1.6 g, 10.0 mmol). The product was purified by recrystallization (3% ethyl acetate/hexanes). White solid. 1.0 g (32% yield).

^{1}H NMR (600 MHz, Chloroform-*d*) δ 8.23 (s, 1H), 7.68 (d, *J* = 8.4 Hz, 2H), 7.61 (d, *J* = 8.5 Hz, 2H), 4.43 (dd, *J* = 7.6, 5.2 Hz, 1H), 2.30 – 2.23 (m, 1H), 2.19 – 2.11 (m, 1H), 1.12 (t, *J* = 7.3 Hz, 3H).

^{13}C NMR (151 MHz, $CDCl_3$) δ 167.1, 140.2, 126.8 (q, *J* = 33.2 Hz), 126.3 (q, *J* = 4.5 Hz), 123.9 (q, *J* = 272 Hz), 119.7, 53.3, 29.2, 11.8.

^{19}F NMR (565 MHz, $CDCl_3$) δ -62.22.

HRMS (ESI) m/z $(M+H)^+$ calcd for $C_{11}H_{12}BrF_3NO$: 310.0049, found: 310.0045.

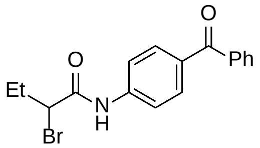

***N*-(4-Benzoylphenyl)-2-bromobutanamide (1g).** The title compound was synthesized according to General procedure A from 2-bromobutanoyl bromide (2.3 g,

10.0 mmol), (4-aminophenyl)(phenyl)methanone (2.0 g, 10.0 mmol). The product was purified by flash chromatography (20% ethyl acetate/hexanes). White solid. 1.9 g (55% yield).

^{1}H NMR (400 MHz, Chloroform-*d*) δ 8.52 (s, 1H), 7.83 (d, *J* = 8.7 Hz, 2H), 7.80 – 7.73 (m, 2H), 7.70 (d, *J* = 8.7 Hz, 2H), 7.58 (t, *J* = 7.4 Hz, 1H), 7.48 (t, *J* = 7.6 Hz, 2H), 4.42 (dd, *J* = 7.7, 5.5 Hz, 1H), 2.31 – 2.20 (m, 1H), 2.19 – 2.07 (m, 1H), 1.09 (t, *J* = 7.3 Hz, 3H).

^{13}C NMR (101 MHz, $CDCl_3$) δ 195.7, 167.1, 141.3, 137.6, 133.6, 132.4, 131.5, 129.9, 128.3, 119.1, 53.1, 29.1, 11.8.

HRMS (ESI) m/z $(M+H)^+$ calcd for $C_{17}H_{17}BrNO_2$: 346.0437, found: 346.0441.

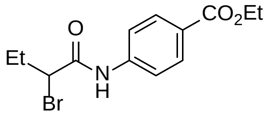

Ethyl 4-(2-bromobutanamido)benzoate (1h). The title compound was synthesized according to General procedure A from 2-bromobutanoyl bromide (2.3 g, 10.0 mmol), benzocaine (1.7 g, 10.0 mmol). The product was purified by flash chromatography (30% ethyl acetate/hexanes). White solid. 2.1 g (67% yield).

^{1}H NMR (400 MHz, Chloroform-d) δ 8.26 (s, 1H), 8.04 (d, *J* = 8.7 Hz, 2H), 7.63 (d, *J* = 8.7 Hz, 2H), 4.46 – 4.30 (m, 3H), 2.30 – 2.21 (m, 1H), 2.20 – 2.12 (m, 1H), 1.39 (t, *J* = 7.1 Hz, 3H), 1.11 (t, *J* = 7.3 Hz, 3H).

^{13}C NMR (151 MHz, Chloroform-d) δ 167.3, 166.2, 141.4, 130.8, 126.6, 119.2, 61.1, 52.8, 29.0, 14.3, 11.9.

HRMS (ESI) m/z $(M+H)^+$ calcd for $C_{13}H_{17}BrNO_3$: 314.0386, found: 314.0391.

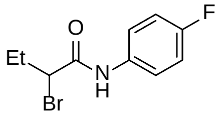

2-Bromo-*N*-(4-fluorophenyl)butanamide (1i). The title compound was synthesized according to General procedure A from 2-bromobutanoyl bromide (2.3 g, 10.0 mmol), 4-fluoroaniline (1.1 g, 10.0 mmol). The product was purified by recrystallization (3% ethyl acetate/hexanes). White solid. 1.6 g (62% yield).

^{1}H NMR (400 MHz, Chloroform-*d*) δ 8.16 (s, 1H), 7.57 – 7.41 (m, 2H), 7.10 – 6.96

(m, 2H), 4.41 (dd, *J* = 7.7, 5.2 Hz, 1H), 2.30 – 2.20 (m, 1H), 2.20 – 2.08 (m, 1H), 1.10 (t, *J* = 7.3 Hz, 3H).

^{19}F NMR (376 MHz, $CDCl_3$) δ -116.89.

^{13}C NMR (101 MHz, $CDCl_3$) δ 166.7, 159.8 (d, *J* = 243.2 Hz), 133.1 (d, *J* = 3.0 Hz), 122.0 (d, *J* = 8.0 Hz), 115.7 (d, *J* = 22.5 Hz), 53.7, 29.3, 11.7.

HRMS (ESI) m/z $(M+H)^+$ calcd for $C_{10}H_{12}BrFNO$: 260.0081, found: 260.0082.

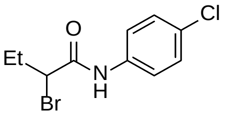

2-Bromo-*N*-(4-chlorophenyl)butanamide (1j). The title compound was synthesized according to General procedure A from 2-bromobutanoyl bromide (2.3 g, 10.0 mmol), 4-chloroaniline (1.3 g, 10.0 mmol). The product was purified by recrystallization (3% ethyl acetate/hexanes). White solid. 1.6 g (58% yield).

^{1}H NMR (600 MHz, Chloroform-*d*) δ 8.13 (s, 1H), 7.49 (d, *J* = 8.6 Hz, 2H), 7.31 (d, *J* = 8.6 Hz, 2H), 4.41 (dd, *J* = 7.5, 5.3 Hz, 1H), 2.28 – 2.21 (m, 1H), 2.17 – 2.09 (m, 1H), 1.10 (t, *J* = 7.2 Hz, 3H).

^{13}C NMR (151 MHz, $CDCl_3$) δ 166.9, 135.6, 130.1, 129.0, 121.4, 53.3, 29.2, 11.8.

HRMS (ESI) m/z $(M+H)^+$ calcd for $C_{10}H_{12}BrClNO$: 275.9785, found: 275.9786.

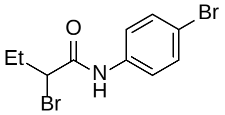

2-Bromo-*N*-(4-bromophenyl)butanamide (1k). The title compound was synthesized according to General procedure A from 2-bromobutanoyl bromide (2.3 g, 10.0 mmol), 4-bromoaniline (1.7 g, 10.0 mmol). The product was purified by recrystallization (3% ethyl acetate/hexanes). White solid. 1.4 g (44% yield).

^{1}H NMR (400 MHz, Chloroform-*d*) δ 8.10 (s, 1H), 7.45 (t, *J* = 2.6 Hz, 4H), 4.41 (dd, *J* = 7.7, 5.1 Hz, 1H), 2.31 – 2.19 (m, 1H), 2.18 – 2.07 (m, 1H), 1.10 (t, *J* = 7.3 Hz, 3H).

^{13}C NMR (151 MHz, $CDCl_3$) δ 166.7, 136.2, 132.0, 121.6, 117.7, 53.7, 29.3, 11.8.

HRMS (ESI) m/z $(M+H)^+$ calcd for $C_{10}H_{12}Br_2NO$: 319.9280, found: 319.9281.

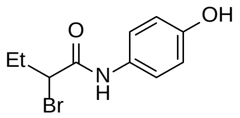

2-Bromo-*N*-(4-hydroxyphenyl)butanamide (1l). The title compound was synthesized according to General procedure A from 2-bromobutanoyl bromide (2.3 g, 10.0 mmol), 4-aminophenol (1.1 g, 10.0 mmol). The product was purified by flash chromatography (30% ethyl acetate/hexanes). White solid. 1.4 g (54% yield).

^{1}H NMR (400 MHz, Chloroform-*d*) δ 8.00 (s, 1H), 7.45 – 7.30 (m, 2H), 6.91 – 6.74 (m, 2H), 5.16 (s, 1H), 4.42 (dd, *J* = 7.7, 5.0 Hz, 1H), 2.32 – 2.08 (m, 2H), 1.11 (t, *J* = 7.3 Hz, 3H).

^{13}C NMR (151 MHz, $CDCl_3$) δ 166.6, 153.1, 130.0, 122.3, 115.7, 54.1, 29.5, 11.7.

HRMS (ESI) m/z $(M+H)^+$ calcd for $C_{10}H_{13}BrNO_2$: 258.0124, found: 258.0128.

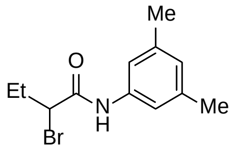

2-Bromo-*N*-(3,5-dimethylphenyl)butanamide (1m). The title compound was synthesized according to General procedure A from 2-bromobutanoyl bromide (2.3 g, 10.0 mmol), 3,5-dimethylaniline (1.2 g, 10.0 mmol). The product was purified by recrystallization (3% ethyl acetate/hexanes). White solid. 1.7 g (63% yield).

^{1}H NMR (400 MHz, Chloroform-*d*) δ 8.08 (s, 1H), 7.18 (s, 2H), 6.79 (s, 1H), 4.41 (dd, *J* = 7.6, 5.3 Hz, 1H), 2.30 (s, 6H), 2.27 – 2.07 (m, 2H), 1.09 (t, *J* = 7.3 Hz, 3H).

^{13}C NMR (101 MHz, $CDCl_3$) δ 166.6, 138.8, 136.9, 126.7, 117.7, 53.9, 29.4, 21.3, 11.7.

HRMS (ESI) m/z $(M+H)^+$ calcd for $C_{12}H_{17}BrNO$: 270.0488, found: 270.0488.

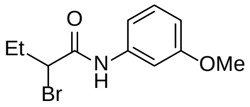

2-Bromo-*N*-(3-methoxyphenyl)butanamide (1n). The title compound was synthesized according to General procedure A from 2-bromobutanoyl bromide (1.2 g, 5.0 mmol), 3-methoxyaniline (0.6 g, 5.0 mmol). The product was purified by recrystallization (3% ethyl acetate/hexanes). White solid. 1.3 g (87% yield).

^{1}H NMR (400 MHz, Chloroform-*d*) δ 8.20 (s, 1H), 7.22 (s, 1H), 7.14 (t, *J* = 8.1 Hz,

1H), 6.94 (d, *J* = 9.0 Hz, 1H), 6.62 (dd, *J* = 8.3, 2.2 Hz, 1H), 4.31 (dd, *J* = 7.5, 5.6 Hz, 1H), 3.71 (s, 3H), 2.19 – 1.99 (m, 2H), 1.00 (t, *J* = 7.2 Hz, 3H).

^{13}C NMR (101 MHz, $CDCl_3$) δ 160.1, 138.3, 129.7, 112.2, 110.9, 105.7, 55.3, 53.4, 29.2, 11.8.

HRMS (ESI) m/z $(M+H)^+$ calcd for $C_{11}H_{15}BrNO_2$: 272.0281, found: 272.0279.

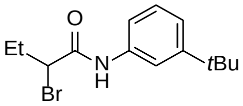

2-Bromo-*N*-(3-(*tert*-butyl)phenyl)butanamide (1o). The title compound was synthesized according to General procedure A from 2-bromobutanoyl bromide (1.2 g, 5.0 mmol), 3-(*tert*-butyl)aniline (0.8 g, 5.0 mmol). The product was purified by recrystallization (3% ethyl acetate/hexanes). White solid. 1.0 g (63% yield).

^{1}H NMR (400 MHz, Chloroform-*d*) δ 8.48 (s, 1H), 7.57 (s, 1H), 7.47 (d, *J* = 7.6 Hz, 1H), 7.29 (t, *J* = 7.8 Hz, 1H), 7.21 (d, *J* = 7.6 Hz, 1H), 4.57 – 4.35 (m, 1H), 2.30 – 2.10 (m, 2H), 1.33 (s, 9H), 1.10 (t, *J* = 7.2 Hz, 3H).

^{13}C NMR (101 MHz, $CDCl_3$) δ 167.0, 152.2, 136.9, 128.6, 122.0, 117.4, 117.2, 53.2, 34.7, 31.2, 29.1, 11.8.

HRMS (ESI) m/z $(M+H)^+$ calcd for $C_{14}H_{21}BrNO$: 298.0801, found: 298.0801.

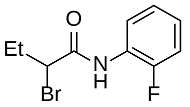

2-Bromo-*N*-(2-fluorophenyl)butanamide (1p). The title compound was synthesized according to General procedure A from 2-bromobutanoyl bromide (2.3 g, 10.0 mmol), 2-fluoroaniline (1.1 g, 10.0 mmol). The product was purified by flash chromatography (20% ethyl acetate/hexanes). White solid. 1.6 g (64% yield).

^{1}H NMR (400 MHz, Chloroform-d) δ 8.33 – 8.15 (m, 2H), 7.15 – 6.99 (m, 3H), 4.37 (dd, *J* = 7.7, 5.2 Hz, 1H), 2.32 – 2.17 (m, 1H), 2.15 – 2.00 (m, 2H), 1.05 (t, *J* = 7.3 Hz, 3H).

^{19}F NMR (376 MHz, Chloroform-d) δ -130.72.

^{13}C NMR (101 MHz, Chloroform-d) δ 166.8, 152.8 (d, *J* = 243.0 Hz), 125.5 (d, *J* =10.2 Hz), 125.1 (d, *J* = 7.6 Hz), 124.4 (d, *J* =3.7 Hz), 121.8, 114.9 (d, J = 18.9 Hz),

53.0, 29.1, 11.7.

HRMS (ESI) m/z $(M+H)^+$ calcd for $C_{10}H_{12}BrFNO$: 260.0081, found: 260.0085.

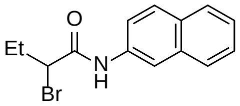

2-Bromo-*N*-(naphthalen-2-yl)butanamide (1q). The title compound was synthesized according to General procedure A from 2-bromobutanoyl bromide (2.3 g, 10.0 mmol), naphthalen-2-amine (1.4 g, 10.0 mmol). The product was purified by recrystallization (3% ethyl acetate/hexanes). White solid. 1.1 g (38% yield).

^{1}H NMR (400 MHz, Chloroform-*d*) δ 8.32 (s, 1H), 8.23 (s, 1H), 7.86 – 7.74 (m, 3H), 7.53 – 7.38 (m, 3H), 4.48 (dd, *J* = 7.6, 5.3 Hz, 1H), 2.37 – 2.11 (m, 2H), 1.13 (t, *J* = 7.3 Hz, 3H).

^{13}C NMR (101 MHz, $CDCl_3$) δ 166.8, 134.5, 133.7, 130.9, 128.9, 127.7, 127.6, 126.6, 125.4, 119.7, 117.1, 53.9, 29.4, 11.8.

HRMS (ESI) m/z $(M+H)^+$ calcd for $C_{14}H_{15}BrNO$: 292.0332, found: 292.0333.

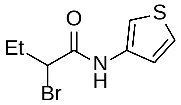

2-Bromo-*N*-(thiophen-3-yl)butanamide (1r). The title compound was synthesized according to General procedure A from 2-bromobutanoyl bromide (2.3 g, 10.0 mmol), 3-aminothiophene (0.9 g, 10.0 mmol). The product was purified by flash chromatography (20% ethyl acetate/hexanes). White solid. 1.4 g (67% yield).

^{1}H NMR (400 MHz, Chloroform-d) δ 8.44 (s, 1H), 7.64 – 7.62 (m, 1H), 7.52 – 7.17 (m, 1H), 7.07 – 7.06 (m, 1H), 4.46 – 4.43 (m, 1H), 2.33 – 2.11 (m, 2H), 1.12 (t, *J* = 7.3 Hz, 3H).

^{13}C NMR (151 MHz, Chloroform-d) δ 166.9, 134.8, 124.7, 121.2, 111.3, 52.0, 29.0, 11.8.

HRMS (ESI) m/z $(M+H)^+$ calcd for $C_8H_{11}BrNOS$: 247.9739, found: 247.9744.

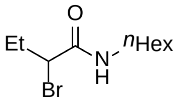

2-Bromo-*N*-hexylbutanamide (1s). The title compound was synthesized according

to General procedure A from 2-bromobutanoyl bromide (2.3 g, 10.0 mmol), hexan-1-amine (1.0 g, 10.0 mmol). The product was purified by flash chromatography (5% ethyl acetate/hexanes). Colorless oil. 2.1 g (84% yield).

^{1}H NMR (400 MHz, Chloroform-*d*) δ 6.46 (s, 1H), 4.30 (dd, *J* = 7.6, 5.0 Hz, 1H), 3.33 – 3.19 (m, 2H), 2.22 – 1.98 (m, 2H), 1.55 – 1.48 (m, 2H), 1.35 – 1.25 (m, 6H), 1.03 (t, *J* = 7.3 Hz, 3H), 0.88 (t, *J* = 6.4 Hz, 3H).

^{13}C NMR (101 MHz, $CDCl_3$) δ 168.4, 54.2, 40.1, 31.4, 29.4, 29.2, 26.4, 22.5, 13.9, 11.6.

HRMS (ESI) m/z $(M+H)^+$ calcd for $C_{10}H_{21}BrNO$: 250.0801, found: 250.0802.

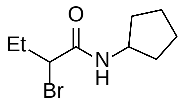

2-Bromo-*N*-cyclopentylbutanamide (1t). The title compound was synthesized according to General procedure A from 2-bromobutanoyl bromide (2.3 g, 10.0 mmol), cyclopentylamine (0.9 g, 10.0 mmol). The product was purified by flash chromatography (20% ethyl acetate/hexanes). White solid. 1.6 g (72% yield).

^{1}H NMR (600 MHz, Chloroform-d) δ 6.34 (s, 1H), 4.28 (dd, *J* = 7.3, 5.1 Hz, 1H), 4.20 – 4.15 (m, 1H), 2.19 – 2.12 (m, 1H), 2.12 – 2.03 (m, 3H), 1.71 – 1.55 (m, 4H), 1.43 – 1.39 (m, 2H), 1.03 (t, *J* = 7.2 Hz, 3H).

^{13}C NMR (151 MHz, Chloroform-d) δ 168.0, 53.9, 51.7, 32.8, 29.2, 23.7, 11.5.

HRMS (ESI) m/z $(M+H)^+$ calcd for $C_9H_{17}BrNO$: 234.0488, found: 234.0492.

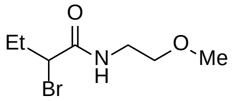

2-Bromo-*N*-(2-methoxyethyl)butanamide (1u). The title compound was synthesized according to General procedure A from 2-bromobutanoyl bromide (2.3 g, 10.0 mmol), 1-methoxy-2-aminoethane (0.8 g, 10.0 mmol). The product was purified by flash chromatography (20% ethyl acetate/hexanes). Colorless oil. 1.4 g (66% yield).

^{1}H NMR (400 MHz, Chloroform-d) δ 6.75 (s, 1H), 4.34 – 4.22 (m, 1H), 3.52 – 3.41 (m, 4H), 3.36 (d, *J* = 1.6 Hz, 3H), 2.22 – 1.96 (m, 2H), 1.08 – 0.98 (m, 3H).

^{13}C NMR (101 MHz, Chloroform-d) δ 168.9, 70.4, 58.4, 52.1, 39.4, 28.7, 11.4.

HRMS (ESI) m/z $(M+H)^+$ calcd for $C_7H_{15}BrNO_2$: 224.0281, found: 224.0284.

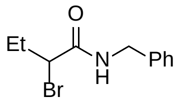

***N*-Benzyl-2-bromobutanamide (1v).** The title compound was synthesized according to General procedure A from 2-bromobutanoyl bromide (2.3 g, 10.0 mmol), benzylamine (1.1 g, 10.0 mmol). The product was purified by flash chromatography (20% ethyl acetate/hexanes). White solid. 1.8 g (72% yield).

^{1}H NMR (400 MHz, Chloroform-d) δ 7.33 – 7.13 (m, 5H), 6.65 (s, 1H), 4.47 – 4.35 (m, 2H), 4.28 (dd, *J* = 7.7, 5.0 Hz, 1H), 2.17 – 2.08 (m, 1H), 2.07 – 1.98 (m, 1H), 0.99 (t, *J* = 7.3 Hz, 3H).

^{13}C NMR (101 MHz, Chloroform-d) δ 168.5, 137.5, 128.8, 127.6, 127.6, 53.6, 53.5, 44.1, 29.3, 29.3, 11.7.

HRMS (ESI) m/z $(M+H)^+$ calcd for $C_{11}H_{15}NO$: 256.0332, found: 256.0335.

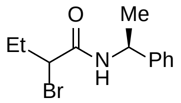

2-Bromo-*N*-((*S*)-1-phenylethyl)butanamide (1w). The title compound was synthesized according to General procedure A from 2-bromobutanoyl bromide (2.3 g, 10.0 mmol), L-1-phenylethylamine (1.2 g, 10.0 mmol). The product was purified by flash chromatography (20% ethyl acetate/hexanes). White solid. 1.8 g (67% yield).

^{1}H NMR (400 MHz, Chloroform-d) δ 7.37 – 7.27 (m, 5H), 6.66 (s, 1H), 5.13 – 5.06 (m, 1H), 4.32 – 4.26 (m, 1H), 2.21 – 2.14 (m, 1H), 2.12 – 2.04 (m, 2H), 1.52 (d, *J* = 6.9 Hz, 3H), 1.05 (t, *J* = 7.3 Hz, 3H).

^{13}C NMR (101 MHz, Chloroform-d) δ 128.7, 127.5, 126.0, 53.2, 53.2, 49.4, 29.2, 11.6.

HRMS (ESI) m/z $(M+H)^+$ calcd for $C_{12}H_{17}BrNO$: 270.0488, found: 270.10491.

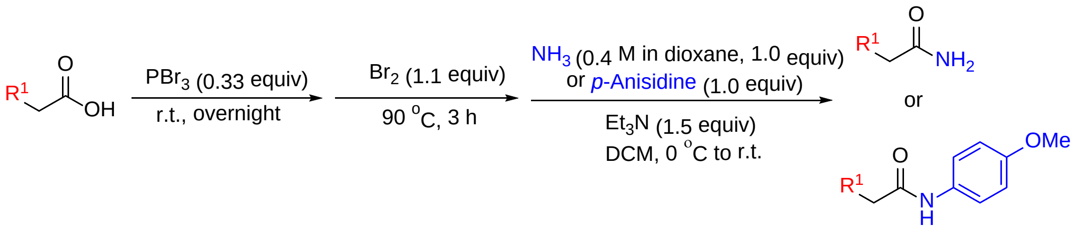

General procedure B: Alkyl carboxylic acid (10.0 mmol) and PBr_3 (0.3 mL, 3.7 mmol) were sequentially added to a 10-mL vial equipped with a stir bar under nitrogen atmosphere. The resulting mixture vigorously stirred under nitrogen atmosphere overnight at room temperature. After this time, Br_2 (0.5 mL, 11.0 mmol) was added to the reaction mixture and the temperature was raised up 110 ℃. The resulting mixture was vigorously stirred under nitrogen atmosphere for 3 h at 110 ℃. Next, additional Br_2 (0.3 mL, 5.6 mmol) was added to the mixture to complete the reaction. After stirring for 3 h at 110 ℃, the mixture was cooled to room temperature and cyclohexene was added to the mixture (2.0 mL, 40.0 mmol). The resulting crude α-bromo acid bromide was used for the next step.

Triethylamine (15.0 mmol) and ammonia (0.4 M in dioxane, 10.0 mmol) or *p*-anisidine (1.3 g, 10.0 mmol) were stirred in DCM (30.0 mL) at 0 ℃, the α-bromo acid bromide was dissolved in DCM (5.0 mL) and added dropwise, the mixture was stirred for 0.5 h. The cooling bath was removed and the mixture was stirred overnight. The mixture was wash with water and extracted with DCM. Solvents were evaporated, and the product was purified by flash chromatography.

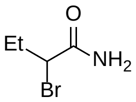

2-bromobutanamide (1x). The title compound was synthesized according to General procedure B from 2-bromobutanoyl bromide (2.3 g, 10.0 mmol), NH_3 (0.4 M in 1,4-dioxane) (25.0 mL, 10.0 mmol). The product was purified by flash chromatography (50% ethyl acetate/hexanes). White solid (0.6 g, 38% yield).

^{1}H NMR (400 MHz, Chloroform-d) δ 6.36 (s, 1H), 5.73 (s, 1H), 4.28 (dd, *J* = 7.7, 5.2 Hz, 1H), 2.22 – 2.13 (m, 1H), 2.12 – 2.03 (m, 1H), 1.08 (t, *J* = 7.3 Hz, 3H).

^{13}C NMR (151 MHz, Chloroform-d) δ 171.6, 52.3, 29.2, 11.6.

HRMS (ESI) m/z $(M+H)^+$ calcd for C_4H_9BrNO: 165.9862, found: 165.9864.

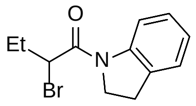

2-Bromo-1-(indolin-1-yl)butan-1-one (1y). The title compound was synthesized according to General procedure A from 2-bromobutanoyl bromide (2.3 g, 10.0 mmol), indoline (1.2 g, 10.0 mmol). The product was purified by flash chromatography (10% ethyl acetate/hexanes). White solid. 2.2 g (82% yield).

^1H NMR (400 MHz, Chloroform-*d*) δ 8.30 (d, *J* = 8.0 Hz, 1H), 7.28 – 7.17 (m, 2H), 7.09 (t, *J* = 7.3 Hz, 1H), 4.44 – 4.26 (m, 2H), 4.14 – 4.07 (m, 1H), 3.33 – 3.21 (m, 2H), 2.36 – 2.25 (m, 1H), 2.21 – 2.12 (m, 1H), 1.09 (t, *J* = 7.3 Hz, 3H).

^{13}C NMR (101 MHz, $CDCl_3$) δ 166.6, 142.7, 131.4, 127.7, 124.6, 124.4, 117.6, 48.2, 47.8, 28.0, 27.9, 12.3.

HRMS (ESI) m/z $(M+H)^+$ calcd for $C_{12}H_{15}BrNO$: 268.0332, found: 268.0330.

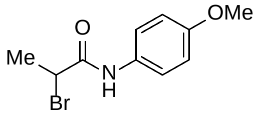

2-Bromo-3-methylbutanamide (1z). The title compound was synthesized according to General procedure B from 2-bromopropanoyl bromide (2.1 g, 10.0 mmol), 4-methoxyaniline (1.3 g, 10.0 mmol) The product was purified by flash chromatography (20% ethyl acetate/hexanes). White solid. 1.6 g (65% yield).

^1H NMR (400 MHz, Chloroform-d) δ 8.05 (s, 1H), 7.42 (d, *J* = 9.0 Hz, 2H), 6.87 (d, *J* = 9.0 Hz, 2H), 4.54 (q, *J* = 7.0 Hz, 1H), 3.79 (s, 3H), 1.95 (d, *J* = 7.0 Hz, 3H).

^{13}C NMR (101 MHz, Chloroform-d) δ 167.3, 156.9, 130.1, 122.0, 114.1, 55.4, 45.3, 22.9.

HRMS (ESI) m/z $(M+H)^+$ calcd for $C_{10}H_{13}BrNO_2$: 258.0124, found: 258.0126.

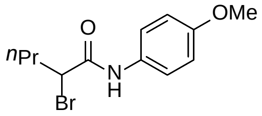

2-Bromo-*N*-(4-methoxyphenyl)pentanamide (1aa). The title compound was synthesized according to General procedure B from 2-bromopentanoyl bromide (2.4 g, 10.0 mmol), 4-methoxyaniline (1.3 g, 10.0 mmol). The product was purified by flash

chromatography (20% ethyl acetate/hexanes). White solid. 1.9 g (67% yield).

^{1}H NMR (600 MHz, Chloroform-d) δ 8.19 (s, 1H), 7.42 (d, *J* = 7.9 Hz, 2H), 6.86 (d, *J* = 8.3 Hz, 2H), 4.43 (d, *J* = 12.7 Hz, 1H), 3.79 (s, 3H), 2.17 (t, *J* = 14.2 Hz, 1H), 2.06 (q, *J* = 13.7, 11.4 Hz, 1H), 1.62 – 1.41 (m, 2H), 0.96 (t, *J* = 7.2 Hz, 3H).

^{13}C NMR (101 MHz, Chloroform-d) δ 167.1, 156.8, 130.2, 122.1, 114.1, 55.4, 51.4, 37.7, 20.5, 13.2.

HRMS (ESI) m/z $(M+H)^+$ calcd for $C_{12}H_{17}BrNO_2$: 286.0437, found: 286.0441.

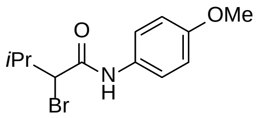

2-Bromo-*N*-(4-methoxyphenyl)-3-methylbutanamide (1ab). The title compound was synthesized according to General procedure B from 2-bromo-3-methylbutanoyl bromide (2.4 g, 10.0 mmol), 4-methoxyaniline (1.3 g, 10.0 mmol). The product was purified by flash chromatography (20% ethyl acetate/hexanes). White solid. 1.8 g (63% yield).

^{1}H NMR (400 MHz, Chloroform-*d*) δ 8.13 (s, 1H), 7.43 (d, *J* = 8.9 Hz, 2H), 6.88 (d, *J* = 8.7 Hz, 2H), 4.44 (dd, *J* = 4.5, 1.5 Hz, 1H), 3.80 (s, 3H), 2.52 – 2.45 (m, 1H), 1.11 (d, *J* = 6.6 Hz, 3H), 1.04 (d, *J* = 6.6 Hz, 3H).

^{13}C NMR (101 MHz, $CDCl_3$) δ 157.0, 130.1, 122.0, 114.2, 62.0, 62.0, 55.5, 32.6, 21.0, 18.4.

HRMS (ESI) m/z $(M+H)^+$ calcd for $C_{12}H_{17}BrNO_2$: 286.0437, found: 286.0436.

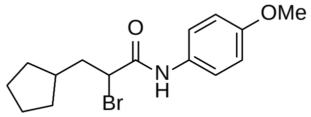

2-Bromo-3-cyclopentyl-*N*-(4-methoxyphenyl)propenamide (1ac). The title compound was synthesized according to General procedure B from 2-bromo-3-cyclopentylpropanoyl bromide (2.8 g, 10.0 mmol), 4-methoxyaniline (1.3 g, 10.0 mmol) The product was purified by flash chromatography (20% ethyl acetate/hexanes). White solid. 2.0 g (63% yield).

^{1}H NMR (600 MHz, Chloroform-d) δ 8.06 (s, 1H), 7.43 (d, *J* = 7.8 Hz, 2H), 6.87 (d, *J* = 7.8 Hz, 2H), 4.44 – 4.37 (m, 1H), 3.79 (s, 3H), 2.20 (dt, *J* = 13.5, 6.6 Hz, 1H), 2.08

(ddt, *J* = 39.3, 15.1, 7.2 Hz, 2H), 1.89 – 1.75 (m, 2H), 1.55 (s, 4H), 1.26 – 1.06 (m, 2H).

^{13}C NMR (101 MHz, Chloroform-d) δ 167.3, 156.8, 130.3, 122.0, 114.1, 55.4, 50.9, 41.9, 38.1, 32.4, 31.8, 25.0.

HRMS (ESI) m/z $(M+H)^+$ calcd for $C_{15}H_{21}BrNO_2$: 326.0750, found: 326.0751.

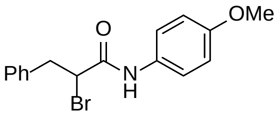

2-Bromo-*N*-(4-methoxyphenyl)-3-phenylpropanamide (1ad). The title compound was synthesized according to General procedure B from 2-bromo-3-phenylpropanoyl bromide (2.9 g, 10.0 mmol), 4-methoxyaniline (1.3 g, 10.0 mmol) The product was purified by flash chromatography (20% ethyl acetate/hexanes). White solid. 1.8 g (55% yield).

^{1}H NMR (400 MHz, Chloroform-*d*) δ 7.72 (s, 1H), 7.35 – 7.08 (m, 7H), 6.78 (d, *J* = 9.0 Hz, 2H), 4.54 (dd, *J* = 7.5, 5.8 Hz, 1H), 3.72 (s, 3H), 3.54 (dd, *J* = 14.3, 5.8 Hz, 1H), 3.28 (dd, *J* = 14.3, 7.5 Hz, 1H).

^{13}C NMR (101 MHz, Chloroform-d) δ 166.0, 157.0, 136.7, 129.9, 129.5, 128.5, 127.3, 122.1, 114.2, 55.5, 51.8, 41.6.

HRMS (ESI) m/z $(M+H)^+$ calcd for $C_{16}H_{17}BrNO_2$: 334.0437, found: 334.0439.

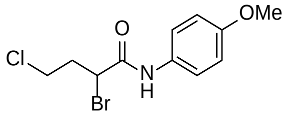

2-Bromo-4-chloro-*N*-(4-methoxyphenyl)butanamide (1ae). The title compound was synthesized according to General procedure B from 2-bromo-4-chlorobutanoyl bromide (2.6 g, 10.0 mmol), 4-methoxyaniline (1.3 g, 10.0 mmol) The product was purified by flash chromatography (20% ethyl acetate/hexanes). White solid. 1.6 g (56% yield).

^{1}H NMR (600 MHz, Chloroform-*d*) δ 7.94 (s, 1H), 7.42 (d, *J* = 8.9 Hz, 2H), 6.88 (d, *J* = 8.9 Hz, 2H), 4.68 (dd, *J* = 8.9, 4.9 Hz, 1H), 3.80 (s, 3H), 3.76 (h, *J* = 6.0 Hz, 2H), 2.72 – 2.65 (m, 1H), 2.48 (ddt, *J* = 14.6, 10.0, 5.2 Hz, 1H).

^{13}C NMR (151 MHz, $CDCl_3$) δ 165.7, 157.0, 130.0, 122.0, 114.2, 55.5, 47.5, 42.1, 37.7.

HRMS (ESI) m/z $(M+H)^+$ calcd for $C_{11}H_{14}BrClNO_2$: 305.9891, found: 305.9892.

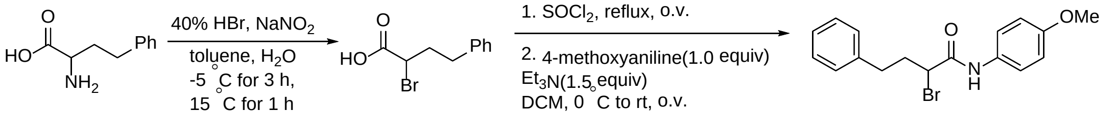

2-Bromo-*N*-(4-methoxyphenyl)-4-phenylbutanamide (1af). A 100 mL flask was charged with 2-amino-4-phenylbutanoic acid (1.8 g, 10.0 mmol), NaBr (3.6 g, 35.0 mmol, 3.5 equiv), and 30.0 mL H_2O, and the mixture was cooled with ice-salt baths. The flask was fitted with a gas outlet adapter attached to a bubbler that was immersed in a 1.0 N aqueous NaOH solution. To the vigorously stirred solution was added 2.5 mL of concentrated H_2SO_4 followed by 2.5 mL of an aqueous solution of 40 wt % $NaNO_2$ (15.3 mmol, 1.3 equiv) at a rate such that no gas evolution was observed. The mixture was stirred at 0 °C for 45 min, then warmed to room temperature and stirred for 6 h. Following this time, the solution was transferred to a separatory funnel and extracted with 3 × 25.0 mL of EtOAc. The combined organic fractions were washed with 1 × 25.0 mL of saturated aqueous NaCl, dried over Na_2SO_4, filtered and concentrated under reduced pressure to a yellow oil. $SOCl_2$ (3.5 mL, 50.0 mmol) was added to the result material and the mixture was refluxed overnight. The excessive $SOCl_2$ was evaporated under vacuum and the result material was dissolved in DCM (5.0 mL), which was added dropwise to a flask equipped with a DCM solution of 4-methoxyaniline (1.2 g, 10.0 mmol) and Et_3N (1.5 g, 15.0 mmol) at 0 °C. The reaction mixture was stirred at room temperature for 12 h. Reaction was quenched with water and washed sequentially with HCl (aq. 1M), Na_2CO_3 (sat.) and brine. Organic layer was collected and dried over Na_2SO_4. Removing the solvent under vacuum, the residue was purified through flash chromatography (20% ethyl acetate/hexanes) to give the amide. White solid. 1.2 g (34% yield).

^{1}H NMR (400 MHz, Chloroform-*d*) δ 7.94 (s, 1H), 7.41 (d, *J* = 9.0 Hz, 2H), 7.33 – 7.20 (m, 5H), 6.87 (d, *J* = 9.0 Hz, 2H), 4.35 (dd, *J* = 8.6, 4.9 Hz, 1H), 3.79 (s, 3H), 3.00 – 2.79 (m, 2H), 2.64 – 2.51 (m, 1H), 2.51 – 2.32 (m, 1H).

^{13}C NMR (101 MHz, Chloroform-d) δ 166.5, 157.0, 139.9, 130.2, 128.6, 126.4, 122.0, 114.2, 55.5, 51.0, 37.2, 33.2.

HRMS (ESI) m/z $(M+H)^+$ calcd for $C_{17}H_{19}BrNO_2$: 348.0594, found: 348.0594.

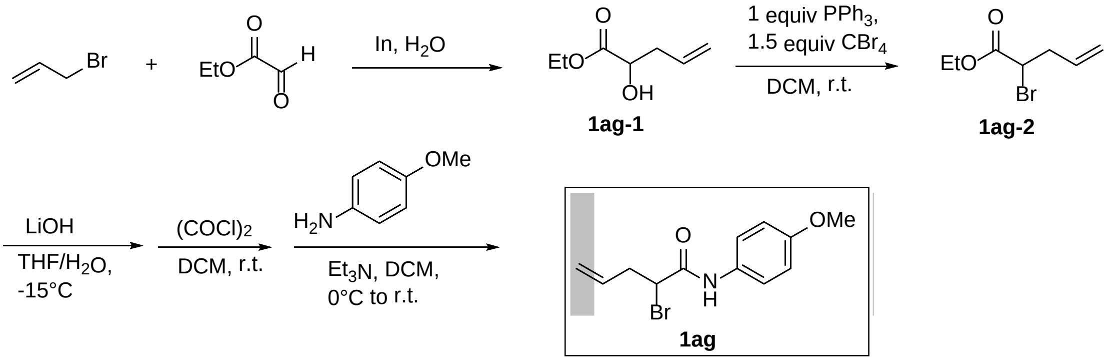

2-Bromo-*N*-(4-methoxyphenyl)pent-4-enamide (1ag). To a slurry of indium powder (2.5 g, 22.0 mmol) in water (100.0 mL) was added ethyl glyoxalate (2.0 g, 20.0 mmol) and allyl bromide (2.6 ml, 30.0 mmol) and the mixture stirred for 24 h. During this time a white solid was precipitated. Ethyl acetate (50.0 ml) was then added and the mixture stirred for 0.5 h. The mixture was partitioned with ethyl acetate (3×50.0 ml), the organic phases dried over sodium sulfate and concentrated in vacuo to give a pale yellow oil. The product was purified by column chromatography, eluting with 20% ethyl acetate in 40/60 petrol to give **1ag-1** as a pale straw coloured liquid (2.0 g, 70%).

To a stirred solution of **1ag-1** (2.0 g, 14.0 mmol) in CH_2Cl_2 (50.0 mL) at 0 °C were added imidazole (1.4 g, 20.0 mmol) and PPh_3 (3.7 g, 14.0 mmol) followed by CBr_4 (10.0 g, 30.0 mmol). The reaction mixture was stirred at room temperature overnight. Upon completion (monitored by TLC), the reaction was quenched by the addition of a saturated aqueous NH_4Cl solution (50.0 mL) and then extracted with CH_2Cl_2 (2 × 50.0 mL). The combined organic extracts were washed with saturated aqueous $NaHCO_3$ solution and brine (50.0 mL), dried with anhydrous Na_2SO_4, and concentrated under reduced pressure. The residue was purified by column chromatography on silica gel (EtOAc/hexane, 1:6) to give bromide **1ag-2** (2.0 g, 71 %) as a colorless oil.

To a solution of **1ag-2** (2.0 g, 10.0 mmol) in a mixture of THF (20.0 mL) and water (10.0 mL), solid LiOH (15.0 mmol) was added and the mixture was stirred at -20 °C. Upon completion (monitored by TLC), the mixture was allowed to warm to room temperature and part of the organic solvents was distilled at reduced pressure. The mixture was acidified with 2 N HCl and extracted with ethyl acetate (3 × 10.0 mL). The combined organic phases were dried with anhydrous Na_2SO_4 and concentrated in vacuo. Then the resulting material was dissolved in CH_2Cl_2 (50.0 mL) with drops of DMF,

oxalyl chloride (2.5 g, 20.0 mmol) was added dropwise at 0 °C, the mixture was stirred at room temperature overnight. The excessive oxalyl chloride was evaporated under vacuum and the result material was dissolved in DCM (5.0 mL), which was added dropwise to a flask equipped with a DCM solution of 4-methoxyaniline (1.2 g, 10.0 mmol) and Et_3N (1.5 g, 15.0 mmol) at 0 °C. The reaction mixture was stirred at room temperature for 12 h. Reaction was quenched with water and washed sequentially with HCl (aq. 1.0 M), Na_2CO_3 (sat.) and brine. Organic layer was collected and dried over Na_2SO_4. Removing the solvent under vacuum, the residue was purified through flash chromatography (20% ethyl acetate/hexanes) to give the amide. White solid. 1.0 g (62% yield).

^{1}H NMR (600 MHz, Chloroform-*d*) δ 7.99 (s, 1H), 7.42 (d, *J* = 8.9 Hz, 2H), 6.88 (d, *J* = 8.9 Hz, 2H), 5.84 (ddt, *J* = 17.1, 10.1, 6.9 Hz, 1H), 5.26 – 5.18 (m, 2H), 4.46 (dd, *J* = 7.4, 5.4 Hz, 1H), 3.80 (s, 3H), 3.05 – 2.95 (m, 1H), 2.87 (dt, *J* = 14.8, 7.3 Hz, 1H).

^{13}C NMR (151 MHz, $CDCl_3$) δ 165.9, 157.0, 133.1, 130.0, 122.0, 119.4, 114.2, 55.5, 50.6, 39.9.

HRMS (ESI) m/z $(M+H)^+$ calcd for $C_{12}H_{15}BrNO_2$: 284.0281, found: 284.0282.

2.2 Synthesis of Chiral Ligands

Chiral ligands **L1**[1], **L8**[2], **L13**[3] and **L17**[4] were prepared according to literature procedures. Chiral ligands **L2**[5], **L7**[6], **L9**, **L10** and **L14** were prepared according to General procedure **C**. Chiral ligands **L11** and **L15** were prepared according to General procedure **D**. Chiral ligands **L16**, **L18**[7], **L19**[7], **L21**, **L22** and **L23** were prepared according to General procedure **E**.

Synthesis of L1

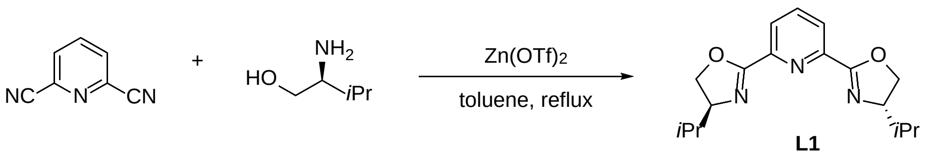

A 100.0 mL round bottom flask was charged with pyridine 2,6-dicarbonitrile (1.3 g, 10.0 mmol, 1.0 equiv), zinc triflate (254.8 mg, 0.7 mmol, 0.07 equiv), and anhydrous toluene (50.0 mL). A solution of (*S*)-valinol (2.0 g, 20.0 mmol, 2.0 equiv) in toluene (10.0 mL) was added. The flask was fit with a reflux condenser and purged several

times with nitrogen. The solution was heated under reflux for 48 h. After cooling, the reaction mixture was diluted with EtOAc (100 mL). The solution was then washed with saturated NaCl (3x50.0 mL), saturated $NaHCO_3$ (3x50.0 mL), and water (50.0 mL). The organic layer was then dried over Na_2SO_4, filtered, and concentrated under reduced pressure to give the product as an off-white crystalline solid. The crude product was purified by recrystallization from hexanes and EtOAc to give the pure product as a white crystalline solid (2.6 g, 86 % yield). ^{1}H NMR (400 MHz, Chloroform-*d*) δ 8.18 (d, *J* = 7.8 Hz, 2H), 7.85 (t, *J* = 7.9 Hz, 1H), 4.61 – 4.45 (m, 2H), 4.22 (t, *J* = 8.4 Hz, 2H), 4.18 – 4.08 (m, 2H), 1.90 – 1.82 (m, 2H), 1.04 (d, *J* = 6.7 Hz, 6H), 0.93 (d, *J* = 6.7 Hz, 6H). ^{13}C NMR (101 MHz, $CDCl_3$) δ 162.2, 146.8, 137.2, 125.7, 72.9, 71.0, 32.8, 19.0, 18.3. The NMR spectra are consistent with the literature data[1].

Synthesis of L3 (General procedure C)

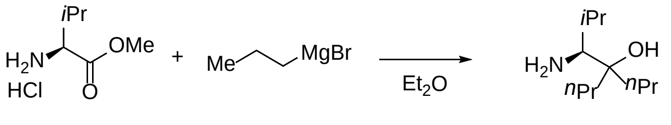

L3-1 (1.0 equiv.) **L3-2** (6.0 equiv.) **L3-3**

To a dried 1000.0 mL three-necked flask, was added magnesium chips (810.0 mmol, 19.4 g), Et_2O (200.0 mL) and two particles of iodine. The mixture was cooled to 0 °C and 1-bromopropane (540.0 mmol, 66.4 g, 49.0 mL) was added dropwise, while the mixture was slight boiling. The reaction was stirred for another two hours to afford the propylmagnesium bromide. Then **L3-1** (90.0 mmol, 15.0 g) was added dropwise at 0 °C, and the reaction mixture was reflux overnight. Saturated ammonium chloride was added at 0 °C until no more solids precipitated out. The mixture was filtered and the organic phase was dried (Na_2SO_4), evaporated in vacuo. The residue was purified by distillation in vacuo (105 °C – 110 °C) to afford **L3-3** as colorless oil (3.4 g, 20% yield).

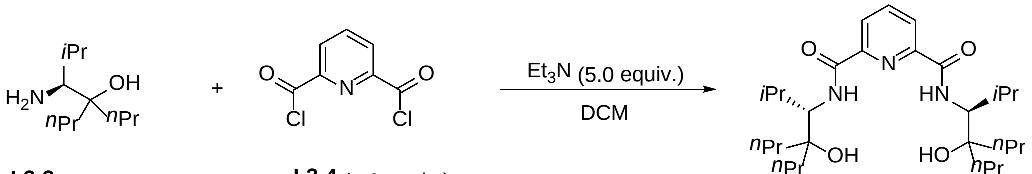

L3-3 (2.0 equiv.) **L3-4** (1.0 equiv.) **L3-5**

To a stirred solution of **L3-3** (1.9 g, 2.0 equiv, 10.0 mmol) in DCM (50.0 mL) was added Et_3N (3.5 mL, 5.0 equiv, 25.0 mmol), the mixture was cooled to 0 °C and **L3-4**

(1.0 g, 1.0 equiv, 5.0 mmol) in DCM (10.0 mL) was added dropwise. Then the mixture was stirred overnight at room temperature. To the reaction mixture was added a saturated aqueous solution of $NaHCO_3$ (100.0 mL) and extracted with DCM (50.0 mL × 2), the combined organic phase was dried (Na_2SO_4) and evaporated in vacuo. The residue was purified by silica gel column chromatography, eluted with hexane/ethyl acetate (2:1) to afford **L3-5** as a white solid (0.8 g, 32% yield).

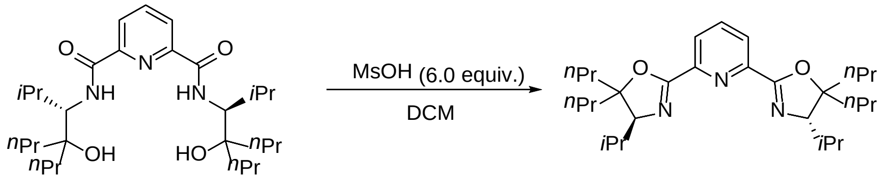

L3-5 (1.0 equiv.) **L3**

To a stirred solution of **L3-5** (0.8 g, 1.5 mmol, 1.0 equiv) in DCM (50.0 mL) under nitrogen atmosphere at 0 °C, was added methanesulfonic acid (0.9 g, 9.0 mmol) dropwise. The solution was allowed to warm to room temperature and stirred for 36 h. To the reaction mixture was added a saturated aqueous solution of $NaHCO_3$ (50.0 mL) and extracted with DCM (30.0 mL × 2), the combined organic phase was dried (Na_2SO_4) and evaporated in vacuo. The residue was purified by silica gel column chromatography, eluted with hexane/ethyl acetate (2:1) to afford **L3** as a white solid (0.2 g, 29% yield). ^{1}H NMR (400 MHz, Chloroform-*d*) δ 7.98 (d, *J* = 7.8 Hz, 2H), 7.87 – 7.78 (m, 1H), 3.73 (s, 2H), 1.97 (dq, *J* = 13.3, 6.6 Hz, 2H), 1.88 – 1.74 (m, 4H), 1.69 – 1.36 (m, 13H), 1.15 (d, *J* = 6.5 Hz, 6H), 1.06 (d, *J* = 6.6 Hz, 6H), 0.96 – 0.89 (m, 12H). ^{13}C NMR (101 MHz, $CDCl_3$) δ 160.6, 147.1, 137.3, 125.0, 92.0, 78.0, 40.2, 35.2, 28.4, 21.8, 20.6, 17.3, 16.8, 14.7, 14.4.

HRMS (ESI) m/z $(M+H)^+$ calcd for $C_{29}H_{48}N_3O_2$: 470.3741, found: 470.3742.

Synthesis of L4

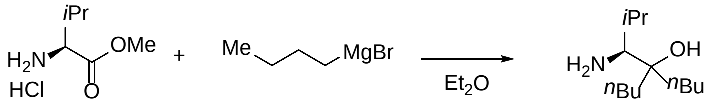

L4-1 (1.0 equiv.) **L4-2** (6.0 equiv.) **L4-3**

To a dried 1000.0 mL three-necked flask, was added magnesium chips (810.0 mmol, 19.4 g), Et_2O (200.0 mL) and two particles of iodine. The mixture was cooled to 0 °C and 1-bromobutane (540.0 mmol, 73.4 g, 57.6 mL) was added dropwise, while the

mixture was slight boiling. The reaction was stirred for another two hours to afford the butylmagnesium bromide. Then **L4-1** (90.0 mmol, 15.0 g) was added in batches at 0 °C, and the reaction mixture was reflux overnight. Saturated ammonium chloride was added at 0 °C until no more solids precipitated out. The mixture was filtered and the organic phase was dried (Na_2SO_4), evaporated in vacuo. The residue was purified by distillation in vacuo (119 °C – 125 °C) to afford **L4-3** as colorless oil (6.3 g, 54% yield).

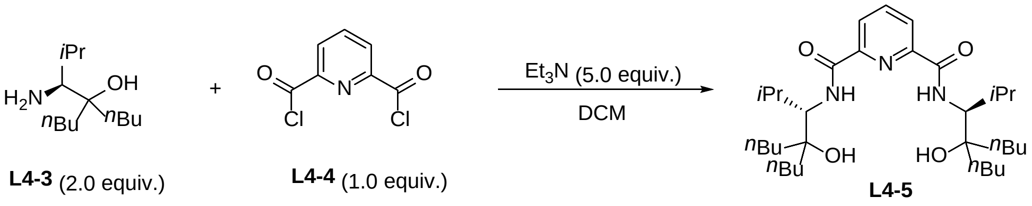

To a stirred solution of **L4-3** (2.2 g, 2.0 equiv, 10.0 mmol) in DCM (50.0 mL) was added Et_3N (3.5 mL, 5.0 equiv, 25.0 mmol), the mixture was cooled to 0 °C and **L4-4** (1.0 g, 1.0 equiv, 5.0 mmol) in DCM (10.0 mL) was added dropwise. Then the mixture was stirred overnight at room temperature. To the reaction mixture was added a saturated aqueous solution of $NaHCO_3$ (100.0 mL) and extracted with DCM (50.0 mL × 2), the combined organic phase was dried (Na_2SO_4) and evaporated in vacuo. The residue was purified by silica gel column chromatography, eluted with hexane/ethyl acetate (2:1) to afford **L4-5** as a white solid (1.3 g, 46% yield).

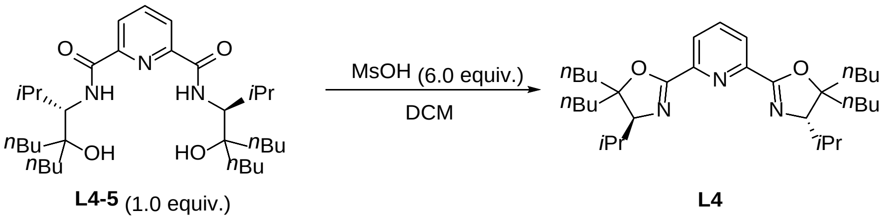

To a stirred solution of **L4-5** (841.0 mg, 1.5 mmol, 1.0 equiv) in DCM (50.0 mL) under nitrogen atmosphere at 0 °C, was added methanesulfonic acid (0.9 g, 9.0 mmol) dropwise. The solution was allowed to warm to room temperature and stirred for 36 h. To the reaction mixture was added a saturated aqueous solution of $NaHCO_3$ (50.0 mL) and extracted with DCM (30.0 mL × 2), the combined organic phase was dried (Na_2SO_4) and evaporated in vacuo. The residue was purified by silica gel column chromatography, eluted with hexane/ethyl acetate (2:1) to afford **L4** as a white solid (0.5 g, 63% yield).

^{1}H NMR (400 MHz, Chloroform-*d*) δ 7.89 (t, *J* = 6.4 Hz, 2H), 7.83 – 7.68 (m, 1H), 3.68 (d, *J* = 7.4 Hz, 2H), 1.95 – 1.86 (m, 2H), 1.81 – 1.70 (m, 4H), 1.62 – 1.54 (m, 4H), 1.47 – 1.40 (m, 2H), 1.34 – 1.20 (m, 14H), 1.09 (d, *J* = 6.5 Hz, 6H), 1.00 (d, *J* = 6.6 Hz, 6H), 0.86 – 0.82 (m, 12H).

^{13}C NMR (101 MHz, $CDCl_3$) δ 160.5, 147.2, 137.4, 124.9, 91.9, 78.0, 37.6, 32.6, 28.4, 26.1, 25.7, 23.3, 23.0, 21.8, 20.6, 14.0.

Synthesis of L5 (General procedure **D)**

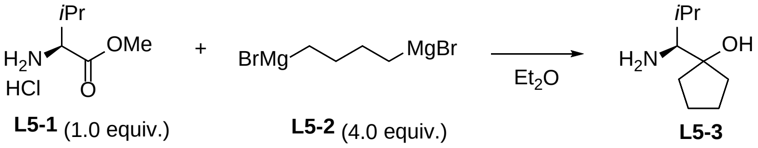

To a dried 1000mL three-necked flask, was added magnesium chips (850.0 mmol, 20.4 g), Et_2O (300.0 mL) and two particles of iodine. The mixture was cooled to 0 °C and 1,4-dibromobutane (400.0 mmol, 85.6 g, 56.0 mL) was added dropwise, while the mixture was slight boiling. The reaction was stirred overnight. Then **L5-1** (100.0 mmol, 16.8 g) was added dropwise at 0 °C, and the reaction mixture was stirred at room temperature for 12 h. Saturated ammonium chloride was added at 0 °C until no more solids precipitated out. The mixture was filtered and the organic phase was dried (Na_2SO_4), evaporated in vacuo. The residue was purified by distillation in vacuo (80 °C) to afford **L5-3** as yellow oil (4.3 g, 27% yield).

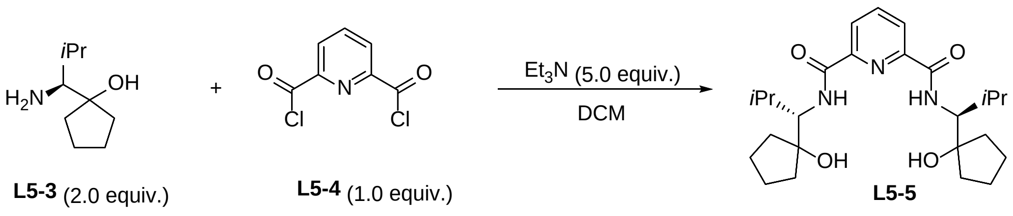

To a stirred solution of **L5-3** (0.8 g, 2.0 equiv, 5.0 mmol) in DCM (50.0 mL) was added Et_3N (1.8 mL, 5.0 equiv, 13.0 mmol), the mixture was cooled to 0 °C and **L5-4** (0.5 g, 1.0 equiv, 2.5 mmol) in DCM (10.0 mL) was added dropwise. Then the mixture was stirred overnight at room temperature. To the reaction mixture was added a saturated aqueous solution of $NaHCO_3$ (100.0 mL) and extracted with DCM (50.0 mL × 2), the combined organic phase was dried (Na_2SO_4) and evaporated in vacuo. The residue was purified by silica gel column chromatography, eluted with hexane/ethyl

acetate (2:1) to afford **L5-5** as a white solid, yield: 0.7 g (61%).

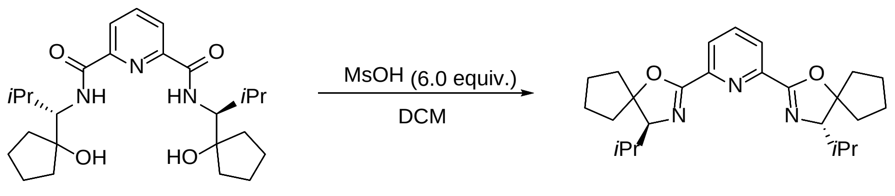

To a stirred solution of **L5-5** (668.0 mg, 1.5 mmol, 1.0 equiv) in DCM (50.0 mL) under nitrogen atmosphere at 0 °C, was added methanesulfonic acid (864.0 mg, 9.0 mmol, 6.0 equiv) dropwise. The solution was allowed to warm to room temperature and stirred for 36 h. To the reaction mixture was added a saturated aqueous solution of $NaHCO_3$ (50.0 mL) and extracted with DCM (30.0 mL × 2), the combined organic phase was dried (Na_2SO_4) and evaporated in vacuo. The residue was purified by silica gel column chromatography, eluted with hexane/ethyl acetate (2:1) to afford **L5** as a white solid, yield: 124.0 mg (20% yield). ^{1}H NMR (400 MHz, Chloroform-*d*) δ 7.99 (d, *J* = 7.8 Hz, 2H), 7.82 (t, *J* = 7.8 Hz, 1H), 3.85 (d, *J* = 6.4 Hz, 2H), 2.16 (p, *J* = 8.8, 7.7 Hz, 2H), 1.99 – 1.82 (m, 11H), 1.81 – 1.67 (m, 6H), 1.07 (dd, *J* = 6.6, 1.6 Hz, 12H). ^{13}C NMR (101 MHz, $CDCl_3$) δ 160.7, 147.1, 137.2, 125.1, 98.8, 77.4, 40.9, 32.3, 30.1, 24.3, 23.1, 21.2, 19.3.

HRMS (ESI) m/z $(M+H)^+$ calcd for $C_{25}H_{36}N_3O_2$: 410.2802, found: 410.2800.

Synthesis of L6

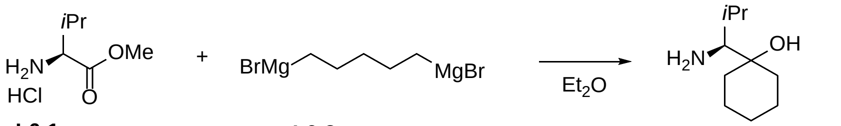

To a dried 1000.0 mL three-necked flask, was added magnesium chips (850.0 mmol, 20.4 g), Et_2O (500.0 mL) and two particles of iodine. The mixture was cooled to 0 °C and 1,5-dibromopentane (400.0 mmol, 91.2 g, 54.0 mL) was added dropwise, while the mixture was slight boiling. The reaction was stirred overnight. Then **L6-1** (100.0 mmol, 16.8 g) was added in batches at 0 °C, and the reaction mixture was stirred at room temperature for 12 h. Saturated ammonium chloride was added at 0 °C until no more solids precipitated out. The mixture was filtered and the organic phase was dried (Na_2SO_4), evaporated in vacuo. The residue was purified by distillation in vacuo (108-

113 °C) to afford **L6-3** as yellow oil (6.5 g, 38% yield).

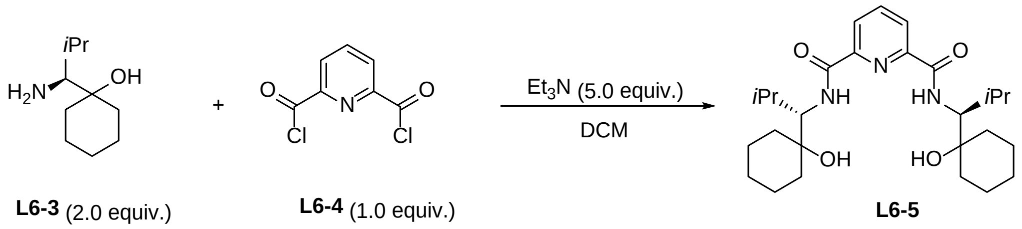

To a stirred solution of **L6-3** (855.0 mg, 2.0 equiv, 5.0 mmol) in DCM (50.0 mL) was added Et_3N (1.8 mL, 5.0 equiv, 13.0 mmol), the mixture was cooled to 0 °C and **L6-4** (0.5 g, 1.0 equiv, 2.5 mmol) in DCM (10.0 mL) was added dropwise. Then the mixture was stirred overnight at room temperature. To the reaction mixture was added a saturated aqueous solution of $NaHCO_3$ (100.0 mL) and extracted with DCM (50.0 mL × 2), the combined organic phase was dried (Na_2SO_4) and evaporated in vacuo. The residue was purified by silica gel column chromatography, eluted with hexane/ethyl acetate (2:1) to afford **L6-5** as a white solid (0.8 g, 68% yield).

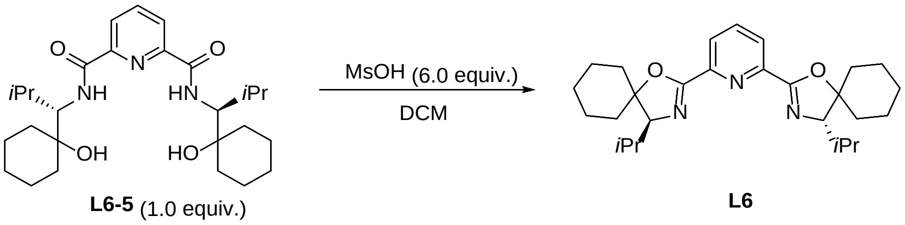

To a stirred solution of **L6-5** (709.5 mg, 1.5 mmol, 1.0 equiv) in DCM (50.0 mL) under nitrogen atmosphere at 0 °C, was added methane sulfonic acid (864.0 mg, 9.0 mmol, 6.0 equiv) dropwise. The solution was allowed to warm to room temperature and stirred for 36 h. To the reaction mixture was added a saturated aqueous solution of $NaHCO_3$ (50.0 mL) and extracted with DCM (30.0 mL × 2), the combined organic phase was dried (Na_2SO_4) and evaporated in vacuo. The residue was purified by silica gel column chromatography, eluted with hexane/ethyl acetate (2:1) to afford **L6** as a white solid (42.3 mg, 32% yield). ^{1}H NMR (400 MHz, Chloroform-*d*) δ 8.01 (d, *J* = 7.8 Hz, 2H), 7.89 – 7.77 (m, 1H), 3.50 (d, *J* = 7.4 Hz, 2H), 1.97 – 1.90 (m, 6H), 1.82 – 1.51 (m, 14H), 1.37 – 1.23 (m, 2H), 1.13 (d, *J* = 6.5 Hz, 6H), 1.07 (d, *J* = 6.6 Hz, 6H). ^{13}C NMR (101 MHz, $CDCl_3$) δ 160.5, 147.3, 137.3, 125.1, 89.3, 80.6, 38.0, 30.0, 28.2, 25.5, 23.0, 22.5, 21.7, 20.4.

Synthesis of L8

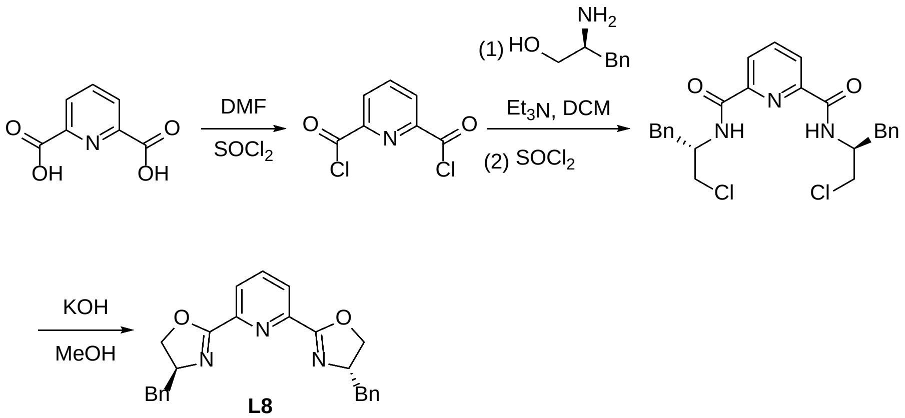

To a mixture of pyridine-2,6-dicarboxylic acid (1.7 g, 10.0 mmol) in DMF (0.05 mL) was added $SOCl_2$ (20.0 mL). The mixture was stirred at reflux for 2 h, during which time the insoluble powder gradually dissolved. The excess amount of $SOCl_2$ was recovered by atmospheric distillation, and the residual amount of $SOCl_2$ was removed under reduced pressure by adding a small amount of toluene. To a solution of (*S*)-2-amino-3-phenylpropan-1-ol (3.8 g, 25 mmol) and triethylamine (4.3 mL, 30.0 mmol) in dichloromethane (30.0 mL), cooled in an ice bath, was added a solution pyridine-2,6-dicarbonyl dichloride (2.0 g, 10.0 mmol) in dichloromethane (30.0 mL) over 10 min; a white precipitate was generated immediately. After stirring at room temperature for 24 h, $SOCl_2$ (20.0 mL) was added to the reaction mixture, which was cooled in an ice-bath. The solution was stirred at reflux for 2 h. The white precipitate dissolved, and the color of the solution turned violent. The solution was concentrated under reduced pressure, and the residual amount of $SOCl_2$ was removed under reduced pressure by adding a small amount of toluene. The resulting residue was triturated with dichloromethane (30.0 mL). The solution was washed with saturated sodium hydrogen carbonate solution (15.0 mL), water (15.0 mL), and brine (15.0 mL) and then dried with sodium sulfate. The solvent was removed, and the crude product was used directly in next step.

To a solution of *N*,*N'*-bis((*S*)-1-chloro-3-phenylpropan-2-yl)pyridine-2,6-dicarboxamide (1.5 g, 5 mmol) in methanol (50.0 mL) was added KOH (0.7 g, 12.5 mmol). The solution was stirred at reflux for 4 h, and white precipitate was generated

during the reaction. The solution was concentrated under reduced pressure. The resulting residue was triturated with dichloromethane (30 mL), and the solution was washed with water (15.0 mL) and brine (15.0 mL) and then dried with sodium sulfate. The crude product was purified by recrystallization from hexanes and EtOAc to give the pure product as a white crystalline solid **L8** (1.5 g, 76 % yield). ^{1}H NMR (400 MHz, Chloroform-*d*) δ 8.24 (d, *J* = 7.8 Hz, 2H), 7.91 (t, *J* = 7.8 Hz, 1H), 7.38 – 7.23 (m, 10H), 4.71 – 4.63 (m, 2H), 4.48 (t, *J* = 9.1 Hz, 2H), 4.28 (t, *J* = 8.1 Hz, 2H), 3.29 (dd, *J* = 13.7, 5.1 Hz, 2H), 2.77 (dd, *J* = 13.7, 9.0 Hz, 2H). ^{13}C NMR (101 MHz, $CDCl_3$) δ 162.7, 146.8, 137.7, 137.3, 129.2, 128.6, 128.6, 126.6, 125.8, 72.6, 68.1, 41.7. The NMR spectra shows good agreement with the literature data[2].

Synthesis of L12

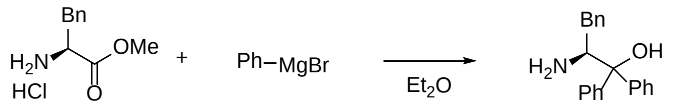

L12-1 (1.0 equiv.) **L12-2** (6.0 equiv.) **L12-3**

To a dried 250.0 mL three-necked flask, was added magnesium chips (270.0 mmol, 6.5 g), Et_2O (100.0 mL) and two particles of iodine. The mixture was cooled to 0 °C and bromobenzene (180.0 mmol, 28.3 g, 19.0 mL) was added dropwise, while the mixture was slight boiling. The reaction was stirred for another two hours to afford the propylmagnesium bromide. Then **L12-1** (30.0 mmol, 6.5 g) was added in batches at 0 °C, and the reaction mixture was reflux overnight. Saturated ammonium chloride was added at 0 °C until no more solids precipitated out. The mixture was filtered and the organic phase was dried (Na_2SO_4), evaporated in vacuo and **L12-3** was afforded as white solid (6.5 g, 72% yield).

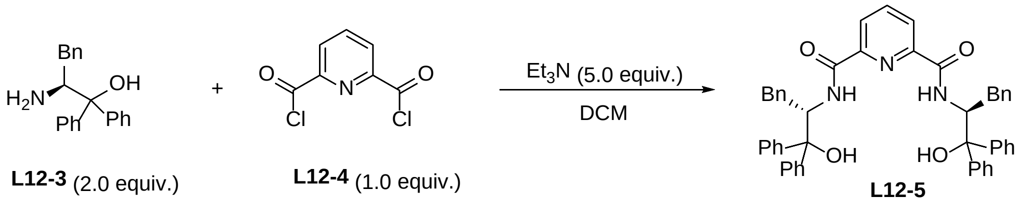

L12-3 (2.0 equiv.) **L12-4** (1.0 equiv.) **L12-5**

To a stirred solution of **L12-3** (3.0 g, 2.0 equiv, 10.0 mmol) in DCM (50.0 mL) was added Et_3N (3.5 mL, 5.0 equiv, 25.0 mmol), the mixture was cooled to 0 °C and **L12-4** (1.0 g, 1.0 equiv, 5.0 mmol) in DCM (10.0 mL) was added dropwise. Then the mixture

was stirred overnight at room temperature. To the reaction mixture was added a saturated aqueous solution of $NaHCO_3$ (100.0 mL) and extracted with DCM (50.0 mL × 2), the combined organic phase was dried (Na_2SO_4) and evaporated in vacuo. The residue was purified by silica gel column chromatography, eluted with hexane/ethyl acetate (4:1) to afford **L12-5** as a white solid (1.9 g, 52%).

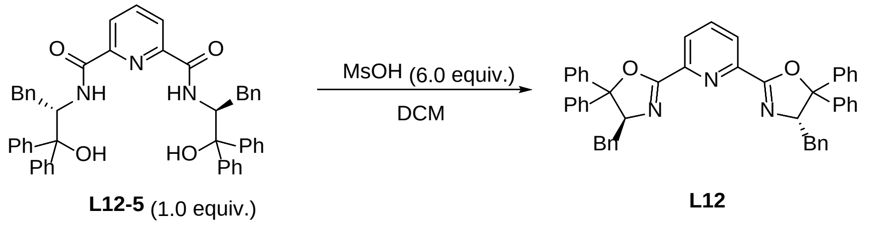

To a stirred solution of **L12-5** (1.1 g, 1.5 mmol, 1.0 equiv) in DCM (50.0 mL) under nitrogen atmosphere at 0 °C, was added methanesulfonic acid (0.9 g, 9.0 mmol) dropwise. The solution was allowed to warm to room temperature and stirred for 36 h. To the reaction mixture was added a saturated aqueous solution of $NaHCO_3$ (50.0 mL) and extracted with DCM (30.0 mL × 2), the combined organic phase was dried (Na_2SO_4) and evaporated in vacuo. The residue was purified by silica gel column chromatography, eluted with hexane/ethyl acetate (4:1) to afford **L12** as a white solid (0.7 g, 67% yield). ^{1}H NMR (400 MHz, Chloroform-*d*) δ 8.13 (d, *J* = 7.8 Hz, 2H), 7.79 (t, *J* = 7.8 Hz, 1H), 7.45 (d, *J* = 7.5 Hz, 4H), 7.26 (t, *J* = 7.4 Hz, 4H), 7.21 – 7.05 (m, 18H), 7.00 (d, *J* = 7.0 Hz, 4H), 5.17 (t, *J* = 7.0 Hz, 2H), 2.59 (d, *J* = 6.6 Hz, 4H). ^{13}C NMR (101 MHz, $CDCl_3$) δ 161.1, 147.1, 143.6, 140.1, 138.7, 137.4, 129.2, 128.3, 128.1, 127.9, 127.8, 127.6, 127.1, 126.4, 126.1, 125.9, 93.8, 77.2, 39.9.

Synthesis of L13

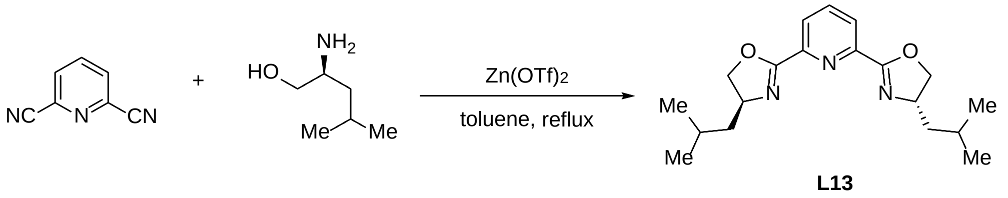

A 100.0 mL round bottom flask was charged with pyridine 2,6-dicarbonitrile (1.3 g, 10.0 mmol, 1.0 equiv), zinc triflate (363.5 mg, 1.0 mmol, 0.1 equiv), and anhydrous toluene (100.0 mL). A solution of (*S*)-2-amino-4-methylpentan-1-ol (2.3 g, 20.0 mmol, 2.0 equiv) in toluene (10.0 mL) was added. The flask was fit with a reflux condenser

and purged several times with nitrogen. The solution was heated under reflux for 48 h. After cooling, the reaction mixture was diluted with EtOAc (100.0 mL). The solution was then washed with saturated NaCl (3x50.0 mL), saturated $NaHCO_3$ (3x50.0 mL), and water (50.0 mL). The organic layer was then dried over Na_2SO_4, filtered, and concentrated under reduced pressure to give the product as an off-white crystalline solid. The crude product was purified by recrystallization from hexanes and EtOAc to give the pure product as a white crystalline solid (2.3 g, 70 % yield). ^{1}H NMR (600 MHz, Chloroform-*d*) δ 8.07 (d, *J* = 7.8 Hz, 2H), 7.79 (t, *J* = 7.8 Hz, 1H), 4.54 (dd, *J* = 9.5, 8.3 Hz, 2H), 4.35 – 4.29 (m, 2H), 4.02 (t, *J* = 8.3 Hz, 2H), 1.82 – 1.75 (m, 2H), 1.69 – 1.64 (m, 2H), 1.39 – 1.29 (m, 2H), 0.92 (d, *J* = 6.6 Hz, 6H), 0.90 (d, *J* = 6.6 Hz, 6H). ^{13}C NMR (151 MHz, $CDCl_3$) δ 162.1, 146.8, 137.3, 125.5, 73.8, 65.3, 45.4, 25.4, 22.7, 22.7. The NMR spectra are consistent with the literature data[3].

Synthesis of L17

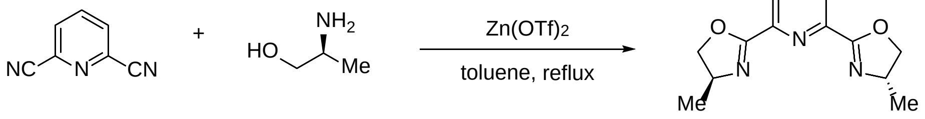

L17

A solution of pyridine-2,6-dicarbonitrile (1.3 g, 10.0 mmol, 1.0 equiv), (*S*)-2-aminopropan-1-ol (750.0 mg, 20.0 mmol, 2.0 equiv) and zinc(II) triflate (363.5 mg, 1.0 mmol, 0.1 equiv.) in toluene (100.0 mL) was heated under reflux for 48 h. The reaction mixture was then cooled to r.t. and diluted with EtOAc (100.0 mL). The organic layer was washed with brine (3 × 50.0 mL), saturated aqueous $NaHCO_3$ solution (3 × 50.0 mL), dried (Na_2SO_4) and evaporated under reduced pressure. The crude product was purified by recrystallization from hexanes and EtOAc to give the pure product as a white crystalline solid (1.9 g, 78 % yield). ^{1}H NMR (400 MHz, Chloroform-*d*) δ 8.14 (d, *J* = 7.9 Hz, 2H), 7.86 (t, *J* = 7.9 Hz, 1H), 4.61 (dd, *J* = 9.5, 8.2 Hz, 2H), 4.51 – 4.35 (m, 2H), 4.06 (t, *J* = 8.1 Hz, 2H), 1.37 (d, *J* = 6.6 Hz, 6H). The NMR spectra shows good agreement with the literature data[4].

Synthesis of (*S,S*)-L20 (General procedure E**)**

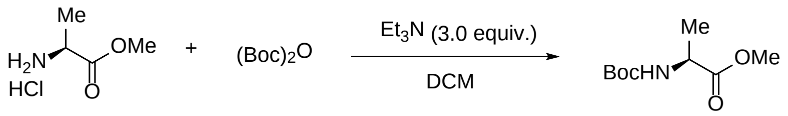

L20-1 (1.0 equiv.) **L20-2** (1.1 equiv.) **L20-3**

To a stirred solution of L-alanine methyl ester hydrochloride (1.0 equiv, 200.0 mmol, 28.0 g) and Et_3N (3.0 equiv, 600.0 mmol, 83.0 mL) in DCM (150.0 mL) under nitrogen atmosphere at 0 °C, was added di-*tert*-butyl dicarbonate (1.1 equiv, 220.0 mmol, 50.0 mL) dropwise. Then the mixture was stirred at room temperature for 12 h. The reaction mixture was washed with saturated citric acid solution (50.0 mL × 2), the organic phase was dried with anhydrous sodium sulfate and evaporated in vacuo to afford **L20-3** as a colorless oil (42.4 g, 104% yield), which was used without further purification.

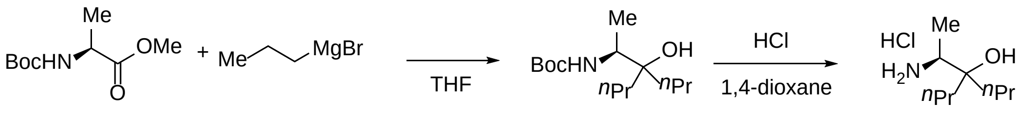

L20-3 (1.0 equiv.) **L20-4** (6.0 equiv.) **L20-5** **L20-6**

To a dried 1000.0 mL three-necked flask, was added magnesium chips (450.0 mmol, 10.8 g), THF (200.0 mL) and two particles of iodine. The mixture was cooled to 0 °C and 1-bromopropane (300.0 mmol, 36.9 g, 27.3 mL) was added dropwise, while the mixture was slight boiling. The reaction was stirred for another two hours to afford the propylmagnesium bromide. Then **L20-3** (50.0 mmol, 10.2 g) was added dropwise at 0 °C, and the reaction mixture was stirred overnight at room temperature. Saturated ammonium chloride was added at 0 °C until no more solids and bubbles. The mixture was extracted with EA (100.0 mL × 3), and the combined organic phase was dried (Na_2SO_4) and evaporated in vacuo. The residue was dissolved in 1,4-dioxane (100.0 mL) and bubbled hydrogen chloride for 4 h, then the mixture was stirred overnight. The solvent was evaporated in vacuo and EA (20.0 mL) was added with white solid precipitating out. The mixture was filtered and washed with EA to afford **L20-6** as white solid (6.2 g, 78% yield), which was used without further purification.

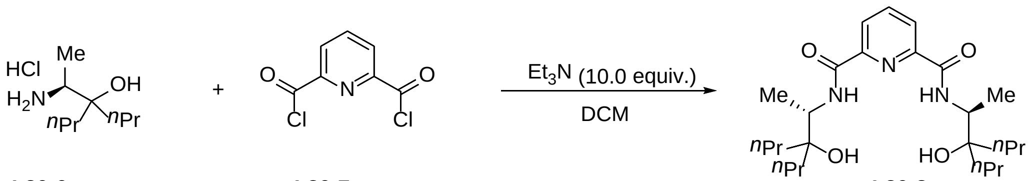

L20-6 (2.0 equiv.) **L20-7** (1.0 equiv.) **L20-8**

To a stirred solution of **L20-6** (3.9 g, 2.0 equiv, 20.0 mmol) in DCM (100.0 mL) was

added Et_3N (13.6 mL, 10.0 equiv, 100.0 mmol), the mixture was cooled to 0 °C and **L20-7** (2.0 g, 1.0 equiv, 10.0 mmol) in DCM (20.0 mL) was added dropwise. Then the mixture was stirred overnight at room temperature. To the reaction mixture was added a saturated aqueous solution of NH_4Cl (150.0 mL) and extracted with DCM (100.0 mL × 2), the combined organic phase was dried (Na_2SO_4) and evaporated in vacuo. The residue was purified by silica gel column chromatography, eluted with hexane/ethyl acetate (2:1) to afford **L20-8** as a white solid (3.5 g, 78% yield).

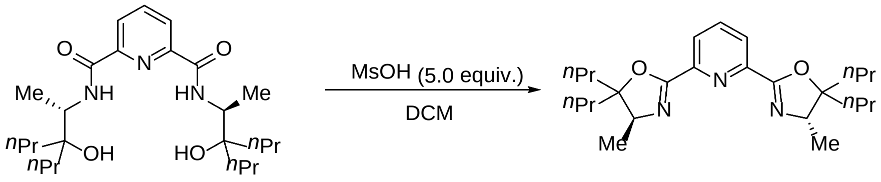

L20-8 (1.0 equiv.) **L20**

To a stirred solution of **L20-8** (2.9 g, 6.5 mmol, 1.0 equiv) in DCM (70.0 mL) under nitrogen atmosphere at 0 °C, was added methanesulfonic acid (3.1 g, 32.5 mmol) dropwise. The solution was allowed to warm to room temperature and stirred for 36 h. To the reaction mixture was added a saturated aqueous solution of $NaHCO_3$ (50.0 mL) and extracted with DCM (30.0 mL × 2), the combined organic phase was dried (Na_2SO_4) and evaporated in vacuo. The residue was purified by silica gel column chromatography, eluted with hexane/ethyl acetate (2:1) to afford ***(S,S)*-L20** as a colorless oil (1.9 g, 71%).

^{1}H NMR (400 MHz, Chloroform-*d*) δ 8.01 (d, *J* = 7.8 Hz, 2H), 7.85 – 7.74 (m, 1H), 4.10 (q, *J* = 7.0 Hz, 2H), 1.84 – 1.60 (m, 6H), 1.59 – 1.32 (m, 9H), 1.27 (d, *J* = 7.1 Hz, 6H), 0.93 (dt, *J* = 10.5, 7.2 Hz, 12H).

^{13}C NMR (101 MHz, $CDCl_3$) δ 160.8, 147.6, 137.0, 125.2, 91.1, 69.0, 40.1, 34.7, 17.3, 16.5, 16.5, 14.6, 14.5.

HRMS (ESI) m/z $(M+H)^+$ calcd for $C_{25}H_{40}N_3O_2$: 414.3115, found: 414.3115.

2.3 Optimization of Reaction Parameters

General procedure: In a nitrogen-filled glovebox, catalyst (0.016 mmol, 8 mol%), chiral ligand (0.016 mmol, 8 mol%) and solvent (1.0 mL) were added to a 10-mL vial equipped with a stir bar. The mixture was allowed to stir for 1 h. Then, additive, Zn, **1**

(51.4 mg, 0.2 mmol), **2a** (118.8 mg, 0.6 mmol, 300 mol%), solvent (2.0 mL) were added. The reaction mixture was transferred out of the glovebox and stirred (~1400 rpm) at room temperature for 24 h. Next, ethyl acetate (20.0 mL) was added, and the mixture was washed with water (10.0 mL) and brine (10.0 mL), dried over Na_2SO_4, and analyzed by GC. The yield was determined versus the internal standard (dodecane).

Supplementary Table 1. Ligand screening

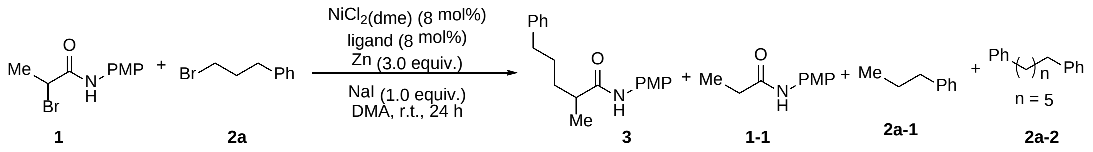

entry	ligand	yield (**1-1**)/%	yield (**2a-1**)/%	yield (**2a-2**)/%	yield (**3**)/%	ee (**3**)/%
1	**S-L1**	82%	17%	11%	0%	--
2	**S-L2**	76%	20%	45%	9%	--
3	**S-L3**	76%	14%	79%	2%	--
4	**S-L4**	78%	13%	76%	2%	--
5	**S-L5**	48%	10%	59%	0%	--
6	**S-L6**	61%	18%	10%	0%	--
7	**S-L7**	49%	12%	54%	8%	50%
8	**S-L8**	41%	9%	56%	29%	60%
9	**S-L9**	29%	8%	58%	33%	64%
10	**S-L10**	64%	10%	68%	10%	52%
11	**S-L11**	32%	20%	24%	27%	53%
12	**S-L12**	55%	17%	35%	23%	55%
13	**S-L13**	41%	15%	20%	32%	53%
14	**S-L14**	42%	11%	54%	8%	--
15	**S-L15**	55%	17%	18%	5%	--
16	**S-L16**	38%	12%	36%	3%	--
17	**S-L17**	49%	15%	42%	3%	--
18	**S-L18**	50%	9%	22%	0%	--
19	**S-L19**	43%	15%	18%	7%	--
20	**S-L20**	25%	19%	52%	38%	51%

Reaction conditions: $NiCl_2$(dme) (0.016 mmol, 8 mol%), ligand (0.016 mmol, 8 mol%), and DMA (2.0 mL) were premixed for 1 h, then added NaI (0.2 mmol, 1.0 equiv), Zn (0.6 mmol, 3.0 equiv), **1** (0.2 mmol, 1.0 equiv) and **2a** (0.6 mmol, 3.0 equiv), the mixture was stirred at r.t. for 24 h. Yields were determined by GC analysis with dodecane as an internal standard.

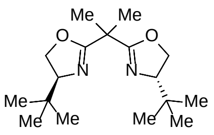

S-L1

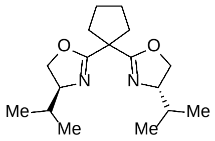

S-L2

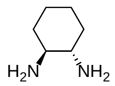

S-L3

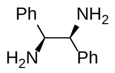

S-L4

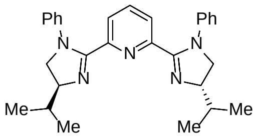

S-L5

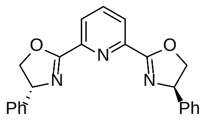

S-L6

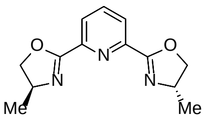

S-L7

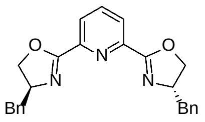

S-L8

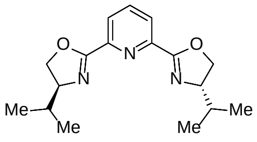

S-L9

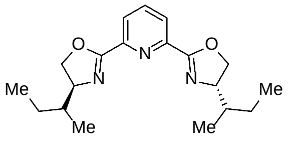

S-L10

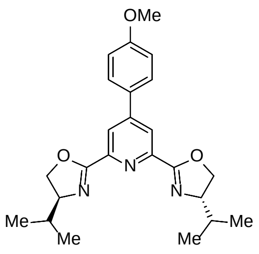

S-L11

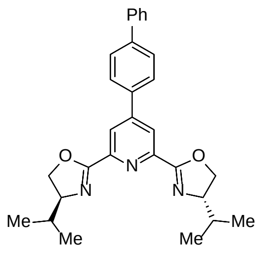

S-L12

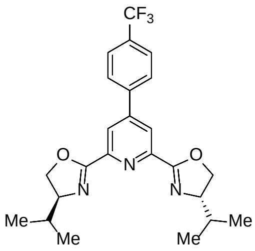

S-L13

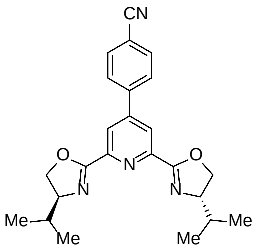

S-L14

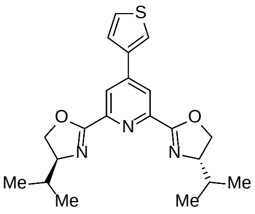

S-L15

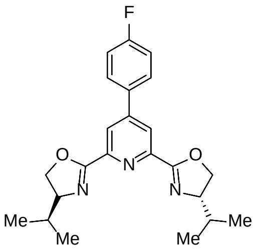

S-L16

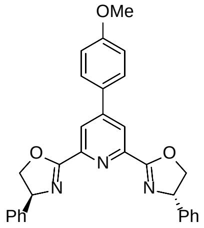

S-L17

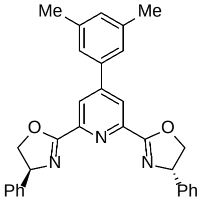

S-L18

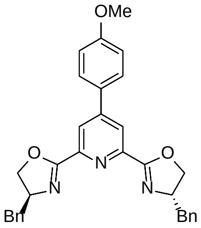

S-L19

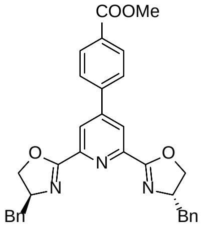

S-L20

Supplementary Table 2. Additive screening

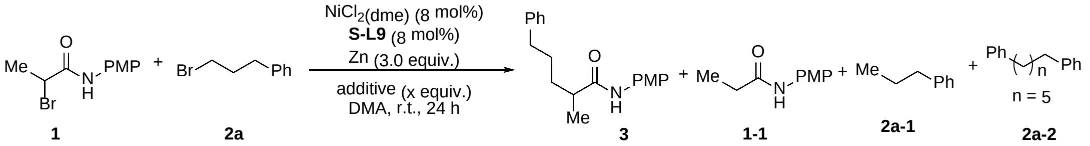

entry[a]	additive	yield (**1-1**)/%	yield (**2a-1**)/%	yield (**2a-2**)/%	yield (**3**)/%[b]	ee (**3**)/%
1	NaI (2.0 equiv)	40%	12%	59%	38%	62%
2	NaI (3.0 equiv)	35%	10%	60%	45%	62%
3	NaI (4.0 equiv)	31%	8%	58%	50%	60%
4	NaI (5.0 equiv)	27%	9%	56%	55%	62%
5	NaI (10.0 equiv)	30%	11%	48%	53%	62%
6	KI (5.0 equiv)	42%	12%	50%	41%	62%
7	Bu_4NI (5.0 equiv)	40%	15%	55%	33%	62%
8	CsI (5.0 equiv)	38%	16%	62%	43%	66%
9[c]	CsI (5.0 equiv)	45%	19%	65%	41%[d]	70%

[a] Reaction conditions: $NiCl_2$(dme) (0.016 mmol, 8 mol%), ligand (0.016 mmol, 8 mol%), and DMA (2.0 mL) were premixed for 1 h, then added additive (x equiv), Zn (0.6 mmol, 3.0 equiv), **1** (0.2 mmol, 1.0 equiv) and **2a** (0.6 mmol, 3.0 equiv), the mixture was stirred at r.t. for 24 h. Yields were determined by GC analysis with dodecane as an internal standard. [b] Determined by GC; [c] **1a** in stead of **1**; [d] **3a** was formed.

Supplementary Table 3. Loading of CsI

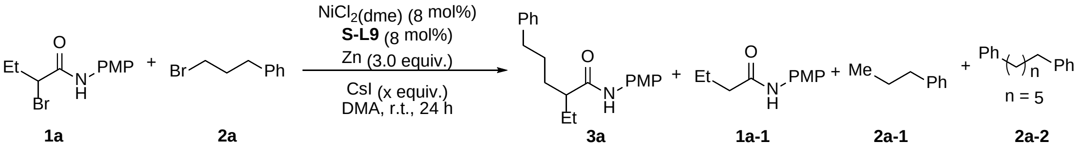

entry[a]	x	yield (1a-1)/%	yield (2a-1)/%	yield (2a-2)/%	yield (3a)/%[b]	ee (3a)/%
1	1.0	75%	4%	65%	18%	66%
2	3.0	51%	7%	66%	42%	70%
3	8.0	49%	13%	67%	41%	68%
4	10.0	48%	16%	63%	41%	68%
5	13.0	52%	18%	68%	39%	68%
6	16.0	51%	20%	64%	42%	68%
7	20.0	52%	17%	70%	43%	68%

[a] Reaction conditions: $NiCl_2$(dme) (0.016 mmol, 8 mol%), ligand (0.016 mmol, 8 mol%), and DMA (2.0 mL) were premixed for 1 h, then added additive (x equiv), Zn (0.6 mmol, 3.0 equiv), **1a** (0.2 mmol, 1.0 equiv) and **2a** (0.6 mmol, 3.0 equiv), the mixture was stirred at r.t. for 24 h. Yields were determined by GC analysis with dodecane as an internal standard. [b] Determined by GC.

Supplementary Table 4. Additive screening

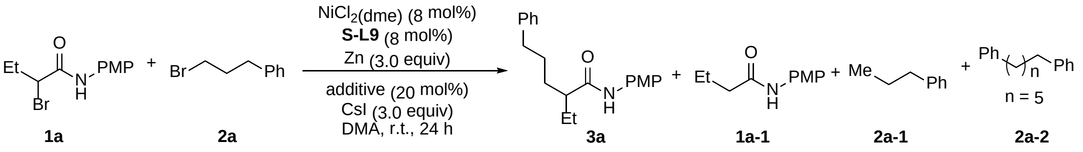

entry[a]	additive	yield (1a-1)/%	yield (2a-1)/%	yield (2a-2)/%	yield (3a)/%[b]	ee (3a)/%
1	ZnI_2	63%	12%	66%	29%	66%
2	$CeCl_3$	79%	16%	65%	10%	50%
3	$ZnCl_2$	68%	10%	67%	27%	64%
4	$Zn(OTf)_2$	65%	9%	62%	23%	62%
5	TMSCl	85%	8%	78%	5%	--
6	$ZnBr_2$	62%	7%	65%	31%	70%
7	$Yb(OTf)_3$	47%	9%	56%	48%	68%
8	$Sc(OTf)_3$	62%	11%	66%	29%	60%
9	$La(OTf)_3$	60%	10%	68%	29%	68%
10	NaOTf	60%	7%	61%	36%	68%
11	$Cu(OTf)_2$	82%	8%	74%	13%	66%
12	$CrCl_2$	88%	15%	78%	7%	--
13	$FeCl_2$	32%	6%	56%	63%	71%
14	$FeCl_3$	34%	7%	60%	62%	70%
15	$CoCl_2$	41%	9%	65%	48%	68%
16	$CuBr_2$	83%	18%	80%	7%	54%
17	CuBr	82%	19%	78%	7%	--
18	CoI_2	70%	11%	66%	18%	54%
19	$CoBr_2$	45%	10%	59%	43%	62%

[a] Reaction conditions: $NiCl_2$(dme) (0.016 mmol, 8 mol%), ligand (0.016 mmol, 8 mol%), and DMA (2.0 mL) were premixed for 1 h, then added additive (20 mol%),CsI (0.6 mmol), Zn (0.6 mmol, 3.0 equiv), **1a** (0.2 mmol, 1.0 equiv) and **2a** (0.6 mmol, 3.0 equiv), the mixture was stirred at r.t. for 24 h. Yields were determined by GC analysis with dodecane as an internal standard. [b] Determined by GC.

Supplementary Table 5. Loading of $FeCl_2$

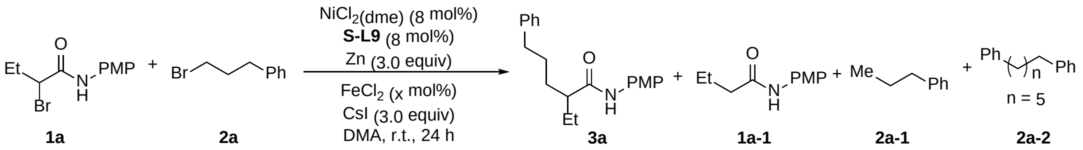

entry[a]	x	yield (**1a-1**)/%	yield (**2a-1**)/%	yield (**2a-2**)/%	yield (**3a**)/%[b]	ee (**3a**)/%
1	5	61%	17%	65%	36%	68%
2	10	40%	15%	57%	56%	70%
3	15	31%	11%	56%	60%	70%
4	25	25%	9%	55%	67%	70%
5	30	23%	9%	58%	65%	70%
6	40	59%	11%	61%	38%	71%
7	50	58%	15%	62%	36%	70%
8	60	75%	12%	68%	22%	70%
9	70	70%	14%	69%	28%	70%
10	80	71%	13%	66%	17%	70%

[a] Reaction conditions: $NiCl_2$(dme) (0.016 mmol, 8 mol%), ligand (0.016 mmol, 8 mol%), and DMA (2.0 mL) were premixed for 1 h, then added $FeCl_2$ (x mol%), CsI (0.6 mmol), Zn (0.6 mmol, 3.0 equiv), **1a** (0.2 mmol, 1.0 equiv) and **2a** (0.6 mmol, 3.0 equiv), the mixture was stirred at r.t. for 24 h. Yields were determined by GC analysis with dodecane as an internal standard. [b] Determined by GC.

Supplementary Table 6. Ligand screening

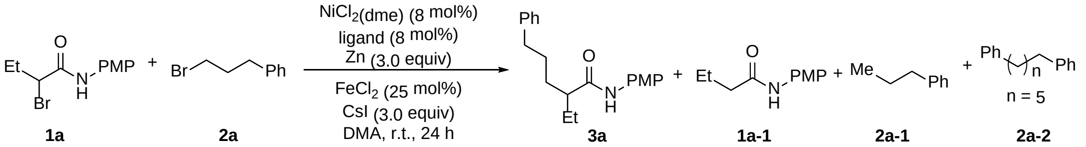

entry[a]	ligand	yield (**1a-1**)/%	yield (**2a-1**)/%	yield (**2a-2**)/%	yield (**3a**)/%[b]	ee (**3a**)/%
1	**L1**	30%	8%	63%	67%	70%
2	**L2**	79%	9%	60%	15%	36%
3	**L3**	77%	10%	74%	16%	38%
4	**L4**	58%	7%	68%	27%	42%
5	**L5**	56%	8%	55%	39%	76%
6	**L6**	61%	9%	62%	30%	71%
7	**L7**	91%	15%	77%	trace	--
8	**L8**	63%	6%	69%	29%	60%
9	**L9**	65%	11%	72%	24%	72%
10	**L10**	36%	12%	70%	49%	84%
11	**L11**	67%	8%	65%	30%	83%
12	**L12**	75%	10%	67%	22%	62%
13	**L13**	68%	11%	67%	31%	60%
14	**L14**	69%	5%	74%	26%	68%
15	**L15**	73%	20%	71%	18%	62%
16	**L16**	60%	18%	65%	29%	90%
17	**L17**	89%	17%	78%	8%	50%
18	**L18**	51%	17%	72%	44%	86%
19	**L19**	52%	14%	63%	45%	91%
20	**L20**	77%	9%	60%	21%	94%
21	**L21**	81%	16%	61%	18%	91%
22	**L22**	88%	7%	58%	10%	78%
23	**L23**	75%	18%	62%	23%	87%

[a] Reaction conditions: $NiCl_2$(dme) (0.016 mmol, 8 mol%), ligand (0.016 mmol, 8 mol%), and DMA (2.0 mL) were premixed for 1 h, then added $FeCl_2$ (25 mol%),CsI (0.6 mmol), Zn (0.6 mmol, 3.0 equiv), **1a** (0.2 mmol, 1.0 equiv) and **2a** (0.6 mmol, 3.0 equiv), the mixture was stirred at r.t. for 24 h. Yields were determined by GC analysis with dodecane as an internal standard. [b] Determined by GC.

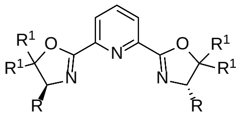

L1: R = *i*Pr, R^1 = H
L2: R = *i*Pr, R^1 = Et
L3: R = *i*Pr, R^1 = nPr
L4: R = *i*Pr, R^1 = nBu
L5: R = *i*Pr, R^1 = -$(CH_2)_4$-
L6: R = *i*Pr, R^1 = -$(CH_2)_5$-
L7: R = *i*Pr, R^1 = Ph
L8: R = Bn, R^1 = H
L9: R = Bn, R^1 = Me
L10: R = Bn, R^1 = Et
L11: R = Bn, R^1 = -$(CH_2)_4$-
L12: R = Bn, R^1 = Ph
L13: R = *i*Bu, R^1 = H
L14: R = *i*Bu, R^1 = Et
L15: R = *i*Bu, R^1 = -$(CH_2)_4$-
L16: R = Et, R^1 = Et
L17: R = Me, R^1 = H
L18: R = Me, R^1 = Me
L19: R = Me, R^1 = Et
L20: R = Me, R^1 = nPr
L21: R = Me, R^1 = nBu
L22: R = Me, R^1 = -$(CH_2)_4$-
L23: R = Me, R^1 = Ph

Supplementary Table 7. Cosolvent screening

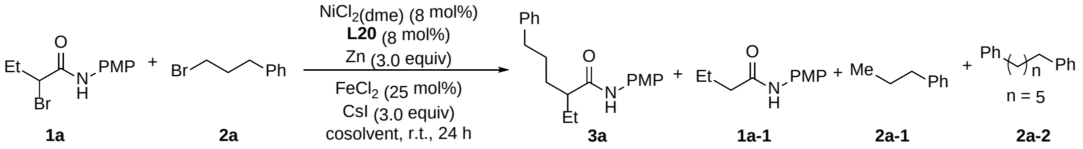

entry[a]	cosolvent	yield (**1a-1**)/%	yield (**2a-1**)/%	yield (**2a-2**)/%	yield (**3a**)/%[b]	ee (**3a**)/%
1	DMA/DMF (3/1)	58%	9%	35%	34%	87%
2	DMA/THF (3/1)	56%	14%	64%	36%	93%
3	DMA/1,4-dioxane (3/1)	45%	8%	66%	51%	91%
4	DMA/DME (3/1)	41%	7%	57%	56%	94%
5	DMA/Et_2O (3/1)	45%	10%	53%	48%	94%
6	DMA/DMI (3/1)	39%	10%	68%	57%	93%
7	DMA/NMP (3/1)	66%	11%	55%	24%	90%
8	DMA/DMSO (3/1)	73%	16%	52%	13%	82%
9	DMA/PhCl (3/1)	70%	13%	53%	25%	90%
10	DMA/MeCN (3/1)	52%	7%	57%	30%	81%
11	DMA/DMPU (3/1)	23%	n.d.	25%	26%	92%

[a] Reaction conditions: $NiCl_2$(dme) (0.016 mmol, 8 mol%), ligand (0.016 mmol, 8 mol%), and solvent (2.0 mL) were premixed for 1 h, then added $FeCl_2$ (25 mol%),CsI (0.6 mmol), Zn (0.6 mmol, 3.0 equiv), **1a** (0.2 mmol, 1.0 equiv) and **2a** (0.6 mmol, 3.0 equiv), the mixture was stirred at r.t. for 24 h. Yields were determined by GC analysis with dodecane as an internal standard. [b] Determined by GC.

Supplementary Table 8. Cosolvent screening

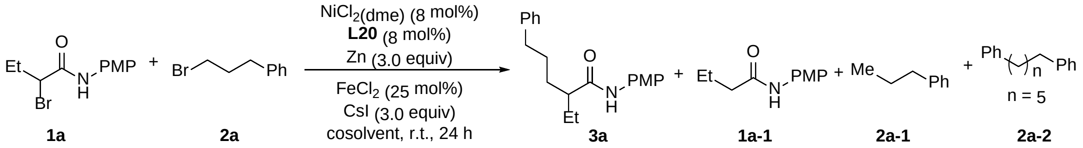

entry[a]	cosolvent	yield (**1a-1**)/%	yield (**2a-1**)/%	yield (**2a-2**)/%	yield (**3a**)/%[b]	ee (**3a**)/%
1	DMA/DME (6/1)	34%	6%	72%	59%	93%
2	DMA/DME (5/1)	36%	6%	70%	52%	94%
3	DMA/DME (4/1)	38%	7%	65%	52%	94%
4	DMA/DME (2/1)	47%	8%	60%	46%	94%
5	DMA/DME (1/1)	58%	8%	52%	33%	94%
6	DMA/DME (1/2)	46%	9%	51%	48%	95%
7	DMA/DME (1/3)	46%	8%	50%	44%	96%
8	DMA/DME (1/4)	45%	6%	41%	24%	96%
9	DMA/DME (1/5)	40%	6%	35%	29%	96%
10	DMA/DME (1/6)	35%	5%	21%	23%	96%
11	DMA/DME (0/1)	<5%	n.d.	<5%	n.d.	--

[a] Reaction conditions: $NiCl_2$(dme) (0.016 mmol, 8 mol%), ligand (0.016 mmol, 8 mol%), and solvent (2.0 mL) were premixed for 1 h, then added $FeCl_2$ (25 mol%),CsI (0.6 mmol), Zn (0.6 mmol, 3.0 equiv), **1a** (0.2 mmol, 1.0 equiv) and **2a** (0.6 mmol, 3.0 equiv), the mixture was stirred at r.t. for 24 h. Yields were determined by GC analysis with dodecane as an internal standard. [b] Determined by GC.

Supplementary Table 9. Cosolvent and additive screening

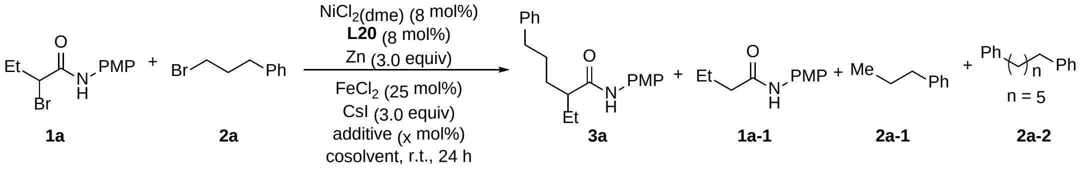

entry[a]	cosolvent	additive	yield (**1a-1**)/%	yield (**2a-1**)/%	yield (**2a-2**)/%	yield (**3a**)/%[b]	ee (**3a**)/%
1	DMA/DME (1/3)	15C5 (10 mol%)	60%	9%	62%	34%	96%
2	DMA/triglyme (1/3)	15C5 (10 mol%)	46%	12%	65%	42%	93%
3	DMA/diglyme (1/3)	15C5 (10 mol%)	12%	14%	66%	85%	94%
4	DMA/diglyme (1/3)	15C5 (20 mol%)	13%	16%	65%	85%	94%
5	DMA/diglyme (1/3)	15C5 (30 mol%)	15%	15%	68%	79%	94%
6	DMA/diglyme (1/3)	15C5 (50 mol%)	11%	13%	65%	82%	94%
7	DMA/diglyme (1/3)	15C5 (100 mol%)	10%	16%	67%	85%	94%
8	DMA/diglyme (1/3)	15C5 (200 mol%)	15%	18%	70%	82%	94%
9	DMA/diglyme (1/3)	15C5 (400 mol%)	19%	20%	61%	74%	93%

[a] Reaction conditions: $NiCl_2$(dme) (0.016 mmol, 8 mol%), ligand (0.016 mmol, 8 mol%), and solvent (2.0 mL) were premixed for 1 h, then added $FeCl_2$ (25 mol%),CsI (0.6 mmol), additive (x mol%), Zn (0.6 mmol, 3.0 equiv), **1a** (0.2 mmol, 1.0 equiv) and **2a** (0.6 mmol, 3.0 equiv), the mixture was stirred at r.t. for 24 h. Yields were determined by GC analysis with dodecane as an internal standard. [b] Determined by GC.

Supplementary Table 10. Loading of Zn and 2a

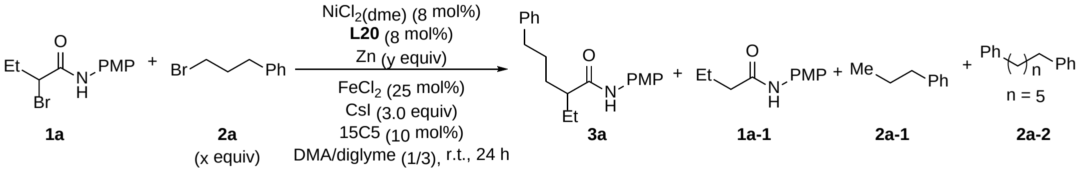

entry[a]	Zn	2a	yield (**1a-1**)/%	yield (**2a-1**)/%	yield (**2a-2**)/%	yield (**3a**)/%[b]	ee (**3a**)/%
1	2.0	3.0	12%	16%	35%	85% (76%)	94%
2	1.5	3.0	32%	10%	24%	65%	94%
3	3.0	2.0	40%	9%	22%	47%	94%
4	3.0	1.5	69%	3%	9%	18%	94%

[a] Reaction conditions: $NiCl_2$(dme) (0.016 mmol, 8 mol%), ligand (0.016 mmol, 8 mol%), and DMA/diglyme (1/3) (2.0 mL) were premixed for 1 h, then added $FeCl_2$ (25 mol%),CsI (0.6 mmol), 15C5 (10 mol%), Zn, **1a** (0.2 mmol, 1.0 equiv) and **2a**, the mixture was stirred at r.t. for 24 h. Yields were determined by GC analysis with dodecane as an internal standard. [b] Determined by GC, isolated yield in parentheses.

Supplementary Table 11. Control Experiments

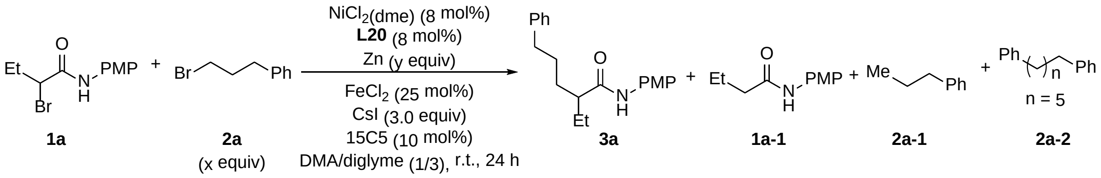

Entry[a]	**2a** (x equiv)	Zn (y equiv)	CsI (z equiv)	**1a-1** (%)[b]	**2a-1** (%)[b]	**2a-2** (%)[b]	**3a** (%)[b]	ee (%)
1	2.0	2.0	3.0	40%	9%	22%	47%	94%
2	1.0	2.0	3.0	75%	3%	16%	15%	94%
3	3.0	1.5	3.0	32%	10%	24%	65%	94%
4	3.0	1.0	3.0	28%	5%	9%	45%	94%
5	3.0	2.0	2.0	34%	6%	58%	58%	94%
6	3.0	2.0	1.0	57%	7%	55%	29%	94%

a Reaction conditions: $NiCl_2$(dme) (0.016 mmol, 8 mol%), **L20** (0.016 mmol, 8 mol%) and diglyme (1.0 mL) was premixed for 1 h, then added CsI (z equiv), $FeCl_2$ (25 mol%), Zn (y equiv), **1a** (0.2 mmol, 1.0 equiv), **2a** (x equiv), diglyme (0.5 mL) and DMA (0.5 mL), the mixture was stirred at r.t. for 24 h. b Determined by GC.

2.4 Enantioconvergent Reductive Alkyl-Alkyl Cross-Coupling

General procedure: In a nitrogen-filled glovebox, $NiCl_2$·glyme (3.5 mg, 0.016 mmol, 8 mol%), chiral ligand ***(S,S)*-L20** (6.6 mg, 0.016 mmol, 8 mol%) and diglyme (1.0 mL) were added to a 10-mL vial equipped with a stir bar. The mixture was allowed to stir for 1 h, after which it was an orange solution. Then, $FeCl_2$ (6.4 mg, 0.05 mmol, 25 mol%), 15C5 (4.4 mg, 0.02 mmol, 10 mol%), CsI (155.9 mg, 0.6 mmol, 300 mol%), Zn (26.2 mg, 0.4 mmol, 200 mol%), **1** (0.2 mmol), **2** (0.6 mmol, 300 mol%), DMA (0.5 mL) and diglyme (0.5 mL) were added. The reaction mixture was transferred out of the glovebox and stirred (~1400 rpm) at room temperature for 24 h. Next, ethyl acetate (20.0 mL) was added, and the mixture was washed with water (10.0 mL) and brine (10.0 mL), dried over Na_2SO_4, filtered, and concentrated under vacuum. The residue was purified by flash chromatography on silica gel.

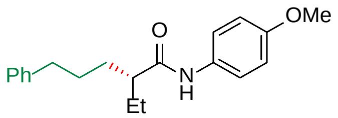

(*R*)-2-Ethyl-*N*-(4-methoxyphenyl)-5-phenylpentanamide (*R*-3a). The title compound was synthesized according to General procedure from (3-bromopropyl)benzene (118.8 mg, 0.6 mmol) and 2-bromo-*N*-(4-methoxyphenyl)butanamide (54.2 mg, 0.2 mmol). The product was purified by flash chromatography (15% ethyl acetate/hexanes). White solid. 47.1 mg (76% yield), 94% ee.

The ee was determined by HPLC on a Daicel CHIRALPAK® OD-H column (10% *i*-PrOH/hexanes, 1.0 mL/min); retention times for compound obtained using ***(S,S)*-L20**: 18.1 min (major), 21.5 min (minor)

$[\alpha]_D^{25} = -58.0$ (c = 0.6, $CHCl_3$).

^{1}H NMR (400 MHz, Chloroform-*d*) δ 7.39 (dd, *J* = 9.6, 2.6 Hz, 3H), 7.25 (t, *J* = 7.3 Hz, 2H), 7.19 – 7.09 (m, 3H), 6.87 – 6.75 (m, 2H), 3.75 (s, 3H), 2.67 – 2.52 (m, 2H), 2.08 (tt, *J* = 9.1, 5.0 Hz, 1H), 1.68 (dtd, *J* = 20.2, 14.0, 12.9, 5.1 Hz, 4H), 1.51 (dq, *J* = 13.2, 7.6, 6.1 Hz, 2H), 0.92 (t, *J* = 7.4 Hz, 3H).

^{13}C NMR (101 MHz, $CDCl_3$) δ 174.0, 156.3, 142.2, 130.9, 128.4, 128.3, 125.7,

121.8, 114.0, 55.4, 50.4, 36.0, 32.5, 29.5, 26.2, 12.1;

HRMS (ESI) m/z $(M+H)^+$ calcd for $C_{20}H_{26}NO_2$: 312.1958, found: 312.1954.

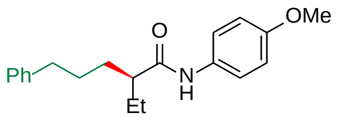

(*S*)-2-Ethyl-*N*-(4-methoxyphenyl)-5-phenylpentanamide (*S*-3a). The title compound was synthesized according to General procedure from (3-bromopropyl)benzene (118.8 mg, 0.6 mmol) and 2-bromo-*N*-(4-methoxyphenyl)butanamide (54.2 mg, 0.2 mmol), using ***(R,R)*-L20** as the ligand. The product was purified by flash chromatography (15% ethyl acetate/hexanes). White solid. 46.7 mg (75% yield), -94% ee.

The ee was determined by HPLC on a Daicel CHIRALPAK® OD-H column (10% *i*-PrOH/hexanes, 1.0 mL/min); retention times for compound obtained using ***(R,R)*-L20**: 19.3 min (major), 21.6 min (minor).

$[\alpha]_D^{25}$ = +49.7 (c = 0.6, $CHCl_3$).

^{1}H NMR (400 MHz, Chloroform-*d*) δ 7.36 – 7.30 (m, 2H), 7.20 – 7.16 (m, 3H), 7.11 – 7.06 (m, 3H), 6.79 – 6.70 (m, 2H), 3.69 (s, 3H), 2.59 – 2.47 (m, 2H), 2.03 – 1.96 (m, 1H), 1.73 – 1.52 (m, 4H), 1.48 – 1.40 (m, 2H), 0.85 (t, J = 7.4 Hz, 3H).

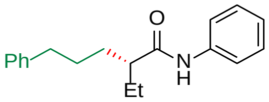

(*R*)-2-Ethyl-*N*,5-diphenylpentanamide (3b). The title compound was synthesized according to General procedure from (3-bromopropyl)benzene (118.8 mg, 0.6 mmol) and 2-bromo-*N*-phenylbutanamide (48.2 mg, 0.2 mmol). The product was purified by flash chromatography (10% ethyl acetate/hexanes). White solid. 37.3 mg (67% yield), 94% ee.

The ee was determined by HPLC on a Daicel CHIRALPAK® OD-H column (10% *i*-PrOH/hexanes, 0.5 mL/min); retention times for compound obtained using ***(S,S)*-L20**: 28.1 min (major), 32.2 min (minor).

$[\alpha]_D^{25}$ = −31.5 (c = 0.4, $CHCl_3$).

^{1}H NMR (600 MHz, Chloroform-*d*) δ 7.44 (d, *J* = 8.0 Hz, 2H), 7.26 – 7.16 (m, 4H), 7.12 – 7.06 (m, 3H), 7.02 (t, *J* = 7.4 Hz, 1H), 2.55 (dqd, *J* = 15.7, 8.0, 7.3, 4.2 Hz, 2H), 2.01 (tt, *J* = 9.4, 5.1 Hz, 1H), 1.73 – 1.58 (m, 4H), 1.47 (ddd, *J* = 12.9, 6.3, 4.2 Hz, 2H), 0.86 (t, *J* = 7.4 Hz, 3H);

^{13}C NMR (151 MHz, $CDCl_3$) δ 174.1, 142.1, 137.8, 128.9, 128.4, 128.3, 125.8, 124.2, 119.9, 50.7, 36.0, 32.5, 29.4, 26.2, 12.1;

HRMS (ESI) m/z $(M+H)^+$ calcd for $C_{19}H_{24}NO$: 282.1852, found: 282.1856.

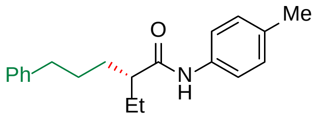

(*R*)-2-Ethyl-5-phenyl-*N*-(*p*-tolyl)pentanamide (3c). The title compound was synthesized according to General procedure from (3-bromopropyl)benzene (118.8 mg, 0.6 mmol) and 2-bromo-*N*-(*p*-tolyl)butanamide (51.0 mg, 0.2 mmol). The product was purified by flash chromatography (10% ethyl acetate/hexanes). White solid. 35.9 mg (61% yield), 93% ee.

The ee was determined by HPLC on a Daicel CHIRALPAK® OD-H column (10% *i*-PrOH/hexanes, 0.5 mL/min); retention times for compound obtained using ***(S,S)*-L20**: 25.9 min (major), 31.6 min (minor).

$[\alpha]_D^{25} = -28.5$ (*c* = 0.4, $CHCl_3$).

^{1}H NMR (600 MHz, Chloroform-*d*) δ 7.32 (d, *J* = 7.6 Hz, 2H), 7.19 (t, *J* = 7.2 Hz, 2H), 7.14 – 7.02 (m, 5H), 7.00 (s, 1H), 2.55 (ddd, *J* = 17.1, 13.8, 6.8 Hz, 2H), 2.23 (s, 3H), 1.99 (dt, *J* = 9.4, 4.3 Hz, 1H), 1.65 (ddd, *J* = 29.0, 14.1, 8.6 Hz, 4H), 1.51 – 1.40 (m, 2H), 0.86 (t, *J* = 7.3 Hz, 3H);

^{13}C NMR (151 MHz, $CDCl_3$) δ 174.1, 142.2, 135.2, 133.8, 129.4, 128.4, 128.3, 125.7, 120.01, 50.5, 36.0, 32.5, 29.5, 26.2, 20.8, 12.0;

HRMS (ESI) m/z $(M+H)^+$ calcd for C20H26NO: 296.2009, found: 296.2013.

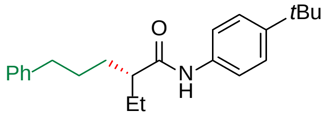

(*R*)-*N*-(4-(*tert*-Butyl)phenyl)-2-ethyl-5-phenylpentanamide (3d). The title compound was synthesized according to General procedure from (3-

bromopropyl)benzene (118.8 mg, 0.6 mmol) and 2-bromo-*N*-(4-(*tert*-butyl)phenyl)butanamide (59.4 mg, 0.2 mmol). The product was purified by flash chromatography (10% ethyl acetate/hexanes). White solid. 39.1 mg (58% yield), 92% ee.

The ee was determined by HPLC on a Daicel CHIRALPAK® OD-H column (10% *i*-PrOH/hexanes, 0.5 mL/min); retention times for compound obtained using **(*S,S)*-L20**: 21.7 min (major), 27.4 min (minor).

$[\alpha]_D^{25}$ = −35.0 (*c* = 0.5, $CHCl_3$).

^{1}H NMR (400 MHz, Chloroform-*d*) δ 7.37 (d, *J* = 8.7 Hz, 2H), 7.26 (d, *J* = 8.7 Hz, 2H), 7.22 – 7.16 (m, 2H), 7.14 – 7.05 (m, 3H), 7.02 (s, 1H), 2.55 (dt, *J* = 8.3, 6.0 Hz, 2H), 1.99 (dq, *J* = 9.3, 4.7 Hz, 1H), 1.76 – 1.56 (m, 4H), 1.48 (dt, *J* = 11.4, 4.8 Hz, 2H), 1.23 (s, 9H), 0.86 (t, *J* = 7.4 Hz, 3H);

^{13}C NMR (101 MHz, $CDCl_3$) δ 174.1, 147.2, 142.1, 135.2, 128.4, 128.3, 125.7, 125.7, 119.7, 50.5, 36.0, 34.3, 32.5, 31.3, 29.4, 26.2, 12.1;

HRMS (ESI) m/z $(M+H)^+$ calcd for $C_{23}H_{32}NO$: 338.2478, found: 338.2483.

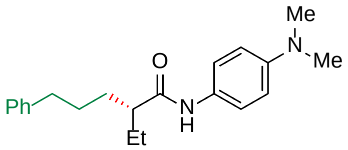

(*R*)-*N*-(4-(Dimethylamino)phenyl)-2-ethyl-5-phenylpentanamide (3e). The title compound was synthesized according to General procedure from (3-bromopropyl)benzene (118.8 mg, 0.6 mmol) and 2-bromo-*N*-(4-(dimethylamino)phenyl)butanamide (56.8 mg, 0.2 mmol). The product was purified by flash chromatography (15% ethyl acetate/hexanes). White solid. 39.8 mg (62% yield), 88% ee.

The ee was determined by HPLC on a Daicel CHIRALPAK® OD-H column (10% *i*-PrOH/hexanes, 1.0 mL/min); retention times for compound obtained using **(*S,S)*-L20**: 32.6 min (major), 38.5 min (minor).

$[\alpha]_D^{25}$ = −41.5 (*c* = 0.5, $CHCl_3$).

^{1}H NMR (600 MHz, Chloroform-*d*) δ 7.32 – 7.23 (m, 2H), 7.21 – 7.12 (m, 3H), 7.12 – 7.02 (m, 3H), 6.63 – 6.53 (m, 2H), 2.81 (s, 6H), 2.52 (pd, *J* = 13.9, 6.4 Hz, 2H), 1.98

(tt, J = 9.4, 5.0 Hz, 1H), 1.72 – 1.52 (m, 4H), 1.43 (ddt, J = 14.6, 7.4, 4.9 Hz, 2H), 0.84 (t, J = 7.4 Hz, 3H).

^{13}C NMR (101 MHz, $CDCl_3$) δ 173.8, 148.0, 142.2, 128.4, 128.32, 128.25, 127.8, 125.7, 121.8, 113.0, 50.4, 40.9, 36.0, 32.6, 29.5, 26.2, 12.1.

HRMS (ESI) m/z $(M+H)^+$ calcd for $C_{21}H_{29}N_2O$: 325.2274, found: 325.2278.

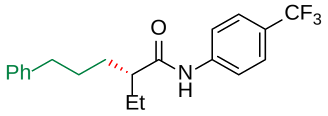

(*R*)-2-Ethyl-5-phenyl-*N*-(4-(trifluoromethyl)phenyl)pentanamide (3f). The title compound was synthesized according to General procedure from (3-bromopropyl)-benzene (118.8 mg, 0.6 mmol) and 2-bromo-*N*-(4-(trifluoromethyl)phenyl)butanamide (61.8 mg, 0.2 mmol). The product was purified by flash chromatography (10% ethyl acetate/hexanes). White solid. 36.2 mg (51% yield), 93% ee.

The ee was determined by HPLC on a Daicel CHIRALPAK® OD-H column (5% *i*-PrOH/hexanes, 1.0 mL/min); retention times for compound obtained using **(*S,S*)-L20**: 20.1 min (minor), 26.7 min (major).

$[\alpha]_D^{25} = -14.2$ (c = 0.4, $CHCl_3$).

^{1}H NMR (600 MHz, Chloroform-*d*) δ 7.57 (d, J = 8.3 Hz, 2H), 7.47 (d, J = 8.3 Hz, 2H), 7.28 (s, 1H), 7.19 (t, J = 7.5 Hz, 2H), 7.09 (dd, J = 22.1, 7.4 Hz, 3H), 2.54 (hept, J = 7.1 Hz, 2H), 2.05 (tt, J = 9.3, 5.1 Hz, 1H), 1.65 (ddd, J = 30.2, 14.4, 9.0 Hz, 4H), 1.49 (dd, J = 13.0, 6.2 Hz, 2H), 0.86 (t, J = 7.4 Hz, 3H).

^{13}C NMR (151 MHz, $CDCl_3$) δ 174.5, 142.0, 140.8, 128.40, 128.37, 126.2 (J = 14.4 Hz), 124.0 (J = 1080 Hz), 119.4, 50.7, 35.9, 32.4, 29.4, 26.1, 12.0.

^{19}F NMR (565 MHz, $CDCl_3$) δ -62.12.

HRMS (ESI) m/z $(M+H)^+$ calcd for $C_{20}H_{23}F_3NO$: 350.1726, found: 350.1731.

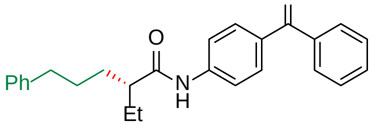

(*R*)-*N*-(4-Benzoylphenyl)-2-ethyl-5-phenylpentanamide (3g). The title compound was synthesized according to General procedure from (3-bromopropyl)benzene (118.8 mg, 0.6 mmol) and *N*-(4-benzoylphenyl)-2-bromobutanamide (69.0 mg, 0.2 mmol).

The product was purified by flash chromatography (15% ethyl acetate/hexanes). Colorless oil. 46.0 mg (60% yield), 93% ee.

The ee was determined by HPLC on a Daicel CHIRALPAK® OD-H column (10% *i*-PrOH/hexanes, 1.0 mL/min); retention times for compound obtained using **(*S*,*S*)-L20**: 22.7 min (major), 30.7 min (minor).

$[\alpha]_D^{25} = -74.5$ (c = 0.6, $CHCl_3$).

^{1}H NMR (600 MHz, Chloroform-*d*) δ 7.73 (s, 2H), 7.68 (s, 2H), 7.58 (d, *J* = 7.5 Hz, 2H), 7.50 (t, *J* = 7.3 Hz, 1H), 7.40 (t, *J* = 7.5 Hz, 2H), 7.20 – 7.17 (m, 2H), 7.09 (dd, *J* = 18.4, 7.2 Hz, 3H), 2.54 (dd, *J* = 15.2, 6.9 Hz, 2H), 2.15 – 2.00 (m, 1H), 1.73 – 1.55 (m, 4H), 1.55 – 1.48 (m, 2H), 0.87 (t, *J* = 7.3 Hz, 3H).

^{13}C NMR (151 MHz, $CDCl_3$) δ 195.8, 174.7, 142.0, 141.9, 137.8, 132.8, 132.2, 131.6, 129.8, 128.4, 128.32, 128.25, 125.8, 118.8, 50.6, 35.9, 32.4, 29.4, 26.1, 12.0.

HRMS (ESI) m/z $(M+H)^+$ calcd for $C_{26}H_{28}NO_2$: 386.2115, found: 386.2119.

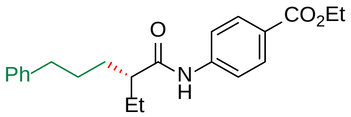

Ethyl (*R*)-4-(2-ethyl-5-phenylpentanamido)benzoate (3h). The title compound was synthesized according to General procedure from (3-iodopropyl)benzene (147.6 mg, 0.6 mmol) and ethyl 4-(2-bromobutanamido)benzoate (62.6 mg, 0.2 mmol). The product was purified by flash chromatography (15% ethyl acetate/hexanes). White solid. 50.3 mg (71% yield), 93% ee.

The ee was determined by HPLC on a Daicel CHIRALPAK® OD-H column (10% *i*-PrOH/hexanes, 1.0 mL/min); retention times for compound obtained using **(*S*,*S*)-L20**: 32.6 min (major), 38.5 min (minor).

$[\alpha]_D^{25} = -0.3$ (c = 0.5, $CHCl_3$).

^{1}H NMR (400 MHz, Chloroform-*d*) δ 7.97 – 7.88 (m, 2H), 7.53 (d, *J* = 8.7 Hz, 2H), 7.20 (d, *J* = 6.1 Hz, 2H), 7.13 – 7.03 (m, 3H), 4.28 (q, *J* = 7.1 Hz, 2H), 2.55 (hept, *J* = 7.1 Hz, 2H), 2.05 (tt, *J* = 8.9, 5.1 Hz, 1H), 1.75 – 1.56 (m, 4H), 1.54 – 1.44 (m, 2H), 1.31 (t, *J* = 7.1 Hz, 3H).

^{13}C NMR (101 MHz, $CDCl_3$) δ 174.5, 166.1, 142.0, 141.9, 130.7, 130.7, 128.4, 128.3,

125.8, 118.8, 60.9, 50.7, 35.9, 32.3, 29.4, 26.1, 14.3, 12.0.

HRMS (ESI) m/z $(M+H)^+$ calcd for $C_{22}H_{28}NO_3$: 354.2064, found: 354.2066.

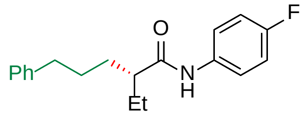

(*R*)-2-Ethyl-*N*-(4-fluorophenyl)-5-phenylpentanamide (3i). The title compound was synthesized according to General procedure from (3-bromopropyl)benzene (118.8 mg, 0.6 mmol) and 2-bromo-*N*-(4-fluorophenyl)butanamide (51.8 mg, 0.2 mmol). The product was purified by flash chromatography (10% ethyl acetate/hexanes). White solid. 32.2 mg (53% yield), 93% ee.

The ee was determined by HPLC on a Daicel CHIRALPAK® OD-H column (10% *i*-PrOH/hexanes, 0.5 mL/min); retention times for compound obtained using **(*S*,*S*)-L20**: 22.8 min (major), 25.5 min (minor).

$[\alpha]_D^{25} = -14.4$ (c = 0.3, $CHCl_3$).

^{1}H NMR (600 MHz, Chloroform-*d*) δ 7.46 – 7.33 (m, 2H), 7.21 – 7.16 (m, 2H), 7.15 – 7.04 (m, 4H), 6.96 – 6.85 (m, 2H), 2.62 – 2.46 (m, 2H), 2.00 (tt, *J* = 9.0, 5.1 Hz, 1H), 1.72 – 1.56 (m, 4H), 1.47 (dddd, *J* = 15.6, 7.5, 6.2, 2.2 Hz, 2H), 0.86 (t, *J* = 7.4 Hz, 3H).

^{19}F NMR (565 MHz, $CDCl_3$) δ -118.03, -118.04, -118.04, -118.05, -118.06, -118.06, -118.07.

^{13}C NMR (101 MHz, $CDCl_3$) δ 174.1, 159.3 (*d, J* = 244.4 Hz), 142.1, 133.7 (d, *J* = 2.9 Hz), 128.4 (d, *J* = 5.1 Hz), 125.8, 121.8 (d, *J* = 7.9 Hz), 115.5 (d, *J* = 22.4 Hz), 50.5, 36.0, 32.4, 29.5, 26.2, 12.1.

HRMS (ESI) m/z $(M+H)^+$ calcd for $C_{19}H_{23}FNO$: 300.1758, found: 300.1765.

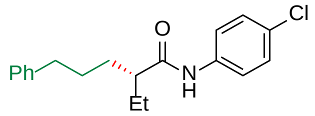

(*R*)-*N*-(4-Chlorophenyl)-2-ethyl-5-phenylpentanamide (3j). The title compound was synthesized according to General procedure from (3-bromopropyl)benzene (118.8 mg, 0.6 mmol) and 2-bromo-*N*-(4-chlorophenyl)butanamide (55.0 mg, 0.2 mmol). The product was purified by flash chromatography (10% ethyl acetate/hexanes). White solid.

39.0 mg (62% yield), 94% ee.

The ee was determined by HPLC on a Daicel CHIRALPAK® IA column (10% *i*-PrOH/hexanes, 0.5 mL/min); retention times for compound obtained using **(*S*,*S*)-L20**: 14.1 min (minor), 16.9 min (major).

$[\alpha]_D^{25} = -39.1$ (c = 0.4, $CHCl_3$).

^1H NMR (400 MHz, Chloroform-*d*) δ 7.42 – 7.33 (m, 2H), 7.24 (s, 1H), 7.21 – 7.14 (m, 4H), 7.13 – 7.02 (m, 3H), 2.62 – 2.45 (m, 2H), 2.09 – 1.93 (m, 1H), 1.69 – 1.54 (m, 4H), 1.46 (dddd, J = 13.8, 7.3, 4.8, 2.7 Hz, 2H), 0.84 (t, J = 7.4 Hz, 3H).

^{13}C NMR (101 MHz, $CDCl_3$) δ 174.3, 142.1, 136.3, 129.2, 128.9, 128.4, 128.3, 125.8, 121.2, 50.5, 35.9, 32.4, 29.4, 26.1, 12.0.

HRMS (ESI) m/z $(M+H)^+$ calcd for $C_{19}H_{23}ClNO$: 316.1463, found: 316.1469.

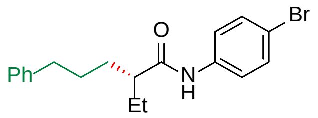

(*R*)-*N*-(4-Bromophenyl)-2-ethyl-5-phenylpentanamide (3k). The title compound was synthesized according to General procedure from (3-bromopropyl)benzene (118.8 mg, 0.6 mmol) and 2-bromo-*N*-(4-bromophenyl)butanamide (63.8 mg, 0.2 mmol). The product was purified by flash chromatography (10% ethyl acetate/hexanes). White solid. 53.0 mg (73% yield), 94% ee.

The ee was determined by HPLC on a Daicel CHIRALPAK® IA column (10% *i*-PrOH/hexanes, 0.5 mL/min); retention times for compound obtained using **(*S*,*S*)-L20**: 15.0 min (minor), 18.3 min (major).

$[\alpha]_D^{25} = -30.8$ (c = 0.5, $CHCl_3$).

^1H NMR (600 MHz, Chloroform-*d*) δ 7.35 – 7.28 (m, 4H), 7.18 (t, J = 7.5 Hz, 2H), 7.13 – 6.97 (m, 3H), 2.59 – 2.44 (m, 2H), 2.02 (tt, J = 9.3, 5.1 Hz, 1H), 1.70 – 1.52 (m, 4H), 1.50 – 1.37 (m, 2H), 0.83 (t, J = 7.4 Hz, 3H).

^{13}C NMR (101 MHz, $CDCl_3$) δ 174.4, 142.0, 136.8, 131.8, 128.34, 128.32, 125.8, 121.5, 116.8, 50.5, 35.9, 32.4, 29.4, 26.1, 12.0.

HRMS (ESI) m/z $(M+H)^+$ calcd for $C_{19}H_{23}BrNO$: 360.0958, found: 360.0962.

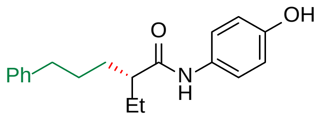

(*R*)-2-Ethyl-*N*-(4-hydroxyphenyl)-5-phenylpentanamide (3l). The title compound was synthesized according to General procedure from (3-bromopropyl)benzene (118.8 mg, 0.6 mmol) and 2-bromo-*N*-(4-hydroxyphenyl)butanamide (51.4 mg, 0.2 mmol). The product was purified by flash chromatography (30% ethyl acetate/hexanes). White solid. 30.1 mg (51% yield), 91% ee.

The ee was determined by HPLC on a Daicel CHIRALPAK® OD-H column (10% *i*-PrOH/hexanes, 1.0 mL/min); retention times for compound obtained using **(*S*,*S*)-L20**: 12.1 min (major), 13.3 min (minor).

$[\alpha]_D^{25} = -8.9$ (c = 0.3, $CHCl_3$).

^{1}H NMR (400 MHz, Chloroform-*d*) δ 7.21 – 7.14 (m, 4H), 7.14 – 7.01 (m, 4H), 6.66 (d, J = 8.8 Hz, 2H), 2.62 – 2.47 (m, J = 6.9 Hz, 2H), 2.00 (tt, J = 9.1, 5.0 Hz, 1H), 1.71 – 1.56 (m, 4H), 1.51 – 1.41 (m, 2H), 0.86 (t, J = 7.4 Hz, 3H).

^{13}C NMR (151 MHz, $CDCl_3$) δ 174.8, 153.6, 142.2, 129.6, 128.4, 128.3, 125.8, 122.9, 115.8, 50.2, 35.9, 32.4, 29.5, 26.2, 12.1.

HRMS (ESI) m/z $(M+H)^+$ calcd for $C_{19}H_{24}NO_2$: 298.1802, found: 298.1806.

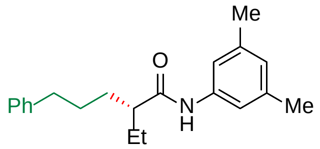

(*R*)-*N*-(3,5-Dimethylphenyl)-2-ethyl-5-phenylpentanamide (3m). The title compound was synthesized according to General procedure from (3-bromopropyl)benzene (118.8 mg, 0.6 mmol) and 2-bromo-*N*-(3,5-dimethylphenyl)butanamide (53.8 mg, 0.2 mmol). The product was purified by flash chromatography (10% ethyl acetate/hexanes). White solid. 36.8 mg (59% yield), 92% ee.

The ee was determined by HPLC on a Daicel CHIRALPAK® OD-H column (5% *i*-PrOH/hexanes, 1.0 mL/min); retention times for compound obtained using **(*S*,*S*)-L20**: 25.9 min (major), 29.4 min (minor).

$[\alpha]_D^{25} = -20.1$ (c = 0.4, $CHCl_3$).

^{1}H NMR (400 MHz, Chloroform-*d*) δ 7.23 – 7.16 (m, 3H), 7.08 (d, *J* = 6.9 Hz, 4H), 6.92 (s, 1H), 6.67 (s, 1H), 2.55 (h, *J* = 7.1 Hz, 2H), 2.22 (s, 6H), 1.97 (tt, *J* = 9.0, 5.0 Hz, 1H), 1.75 – 1.53 (m, 4H), 1.52 – 1.47 (m, 2H), 0.86 (t, *J* = 7.4 Hz, 3H).

^{13}C NMR (101 MHz, $CDCl_3$) δ 174.1, 142.2, 138.7, 137.6, 128.4, 128.3, 125.9, 125.8, 117.5, 50.8, 36.0, 32.5, 29.5, 26.3, 21.3, 12.1.

HRMS (ESI) m/z $(M+H)^+$ calcd for $C_{21}H_{28}NO$: 310.2165, found: 310.2169.

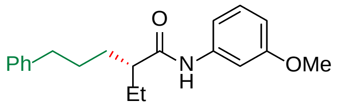

(*R*)-2-Ethyl-*N*-(3-methoxyphenyl)-5-phenylpentanamide (3n). The title compound was synthesized according to General procedure from (3-bromopropyl)benzene (118.8 mg, 0.6 mmol) and 2-bromo-*N*-(3-methoxyphenyl)butanamide (54.2 mg, 0.2 mmol). The product was purified by flash chromatography (20% ethyl acetate/hexanes). White solid. 42.7 mg (69% yield), 94% ee.

The ee was determined by HPLC on a Daicel CHIRALPAK® OD-H column (5% *i*-PrOH/hexanes, 1.0 mL/min); retention times for compound obtained using **(*S*,*S*)-L20**: 15.8 min (major), 23.5 min (minor).

$[\alpha]_D^{25} = -16.7$ (c = 0.5, $CHCl_3$).

^{1}H NMR (600 MHz, Chloroform-*d*) δ 7.28 (s, 1H), 7.20 – 7.16 (m, 3H), 7.11 – 7.06 (m, 4H), 6.87 (d, *J* = 7.9 Hz, 1H), 6.57 (d, *J* = 8.1 Hz, 1H), 3.70 (s, 3H), 2.58 – 2.48 (m, 2H), 2.00 (s, 1H), 1.68 – 1.55 (m, 4H), 1.47 – 1.42 (m, 2H), 0.85 (t, *J* = 7.3 Hz, 3H).

^{13}C NMR (151 MHz, Chloroform-*d*) δ 174.3, 160.1, 142.1, 139.1, 129.5, 128.4, 128.3, 125.8, 111.8, 110.2, 105.4, 55.2, 50.7, 36.0, 32.5, 29.4, 26.2, 12.0.

HRMS (ESI) m/z $(M+H)^+$ calcd for $C_{20}H_{26}NO_2$: 312.1958, found: 312.1955.

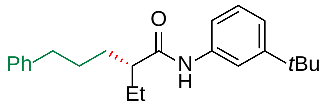

(*R*)-*N*-(3-(*tert*-Butyl)phenyl)-2-ethyl-5-phenylpentanamide (3o). The title compound was synthesized according to General procedure from (3-bromopropyl)benzene (118.8 mg, 0.6 mmol) and 2-bromo-*N*-(3-(*tert*-

butyl)phenyl)butanamide (59.4 mg, 0.2 mmol). The product was purified by flash chromatography (10% ethyl acetate/hexanes). White solid. 47.9 mg (71% yield), 92% ee.

The ee was determined by HPLC on a Daicel CHIRALPAK® OD-H column (10% *i*-PrOH/hexanes, 1.0 mL/min); retention times for compound obtained using **(*S,S*)-L20**: 12.4 min (major), 13.5 min (minor).

$[\alpha]_D^{25} = -19.0$ (c = 0.6, $CHCl_3$).

^{1}H NMR (600 MHz, Chloroform-*d*) δ 7.43 (s, 1H), 7.35 – 7.28 (m, 1H), 7.21 – 7.14 (m, 3H), 7.10 – 7.04 (m, 5H), 2.61 – 2.47 (m, 2H), 2.03 – 1.98 (m, 1H), 1.72 – 1.54 (m, 4H), 1.51 – 1.42 (m, 2H), 1.23 (s, 9H), 0.87 (t, J = 7.4 Hz, 3H).

^{13}C NMR (151 MHz, Chloroform-*d*) δ 174.1, 152.2, 142.2, 137.6, 128.6, 128.4, 128.3, 125.8, 121.3, 117.1, 116.9, 50.7, 36.0, 34.7, 32.5, 31.3, 29.5, 26.2, 12.1.

HRMS (ESI) m/z $(M+H)^+$ calcd for $C_{23}H_{32}NO$: 338.2478, found: 338.2478.

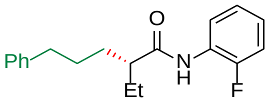

(*R*)-2-Ethyl-*N*-(2-fluorophenyl)-5-phenylpentanamide (3p). The title compound was synthesized according to General procedure from (3-bromopropyl)benzene (118.8 mg, 0.6 mmol) and 2-bromo-*N*-(2-fluorophenyl)butanamide (51.8 mg, 0.2 mmol). The product was purified by flash chromatography (7% ethyl acetate/hexanes). White solid. 34.3 mg (58% yield), 90% ee.

The ee was determined by HPLC on a Daicel CHIRALPAK® AS-H column (10% *i*-PrOH/hexanes, 0.5 mL/min); retention times for compound obtained using **(*S,S*)-L20**: 10.4 min (minor), 11.3 min (major).

$[\alpha]_D^{25} = -27.9$ (c = 0.4, $CHCl_3$).

^{1}H NMR (400 MHz, Chloroform-*d*) δ 8.27 (td, J = 8.1, 1.7 Hz, 1H), 7.32 – 7.16 (m, 3H), 7.15 – 6.84 (m, 6H), 2.67 – 2.44 (m, 2H), 2.08 (tt, J = 8.9, 5.1 Hz, 1H), 1.78 – 1.57 (m, 4H), 1.55 – 1.43 (m, 2H), 0.87 (t, J = 7.4 Hz, 3H).

^{19}F NMR (376 MHz, $CDCl_3$) δ -131.48.

^{13}C NMR (101 MHz, $CDCl_3$) δ 174.2, 152.4 (d, J = 243.6 Hz), 142.0, 128.3 (d, J = 5.1 Hz), 126.3 (d, J = 10.1 Hz), 125.8, 124.5 (d, J = 3.6 Hz), 124.2 (d, J = 7.7 Hz), 121.9, 114.7 (d, J = 19.4 Hz), 50.7, 35.9, 32.4, 29.3, 26.1, 12.0.

HRMS (ESI) m/z $(M+H)^+$ calcd for $C_{19}H_{23}FNO$: 300.1758, found: 300.1762.

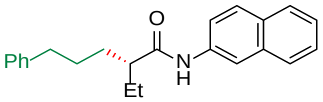

(*R*)-2-Ethyl-*N*-(naphthalen-2-yl)-5-phenylpentanamide (3q). The title compound was synthesized according to General procedure from (3-bromopropyl)benzene (118.8 mg, 0.6 mmol) and 2-bromo-*N*-(naphthalen-2-yl)butanamide (58.2 mg, 0.2 mmol). The product was purified by flash chromatography (10% ethyl acetate/hexanes). White solid. 42.1 mg (63% yield), 93% ee.

The ee was determined by HPLC on a Daicel CHIRALPAK® OD-H column (10% *i*-PrOH/hexanes, 1.0 mL/min); retention times for compound obtained using **(*S*,*S*)-L20**: 25.3 min (major), 31.1 min (minor).

$[\alpha]_D^{25}$ = −46.1 (c = 0.5, $CHCl_3$).

^{1}H NMR (600 MHz, Chloroform-*d*) δ 8.22 – 8.11 (m, 1H), 7.68 (d, J = 8.8 Hz, 3H), 7.45 – 7.27 (m, 4H), 7.21 – 7.15 (m, 2H), 7.15 – 7.00 (m, 3H), 2.55 (qt, J = 13.9, 6.8 Hz, 2H), 2.07 (tt, J = 9.3, 5.1 Hz, 1H), 1.76 – 1.57 (m, 4H), 1.49 (tt, J = 9.9, 4.7 Hz, 2H), 0.88 (t, J = 7.4 Hz, 3H).

^{13}C NMR (101 MHz, $CDCl_3$) δ 174.4, 142.1, 135.2, 133.8, 130.6, 128.7, 128.4, 128.3, 127.6, 127.5, 126.5, 125.8, 125.0, 119.9, 116.7, 50.7, 36.0, 32.5, 29.5, 26.3, 12.1.

HRMS (ESI) m/z $(M+H)^+$ calcd for $C_{23}H_{26}NO$: 332.2009, found: 332.2013.

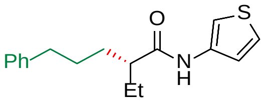

(*R*)-2-Ethyl-5-phenyl-*N*-(thiophen-3-yl)pentanamide (3r). The title compound was synthesized according to General procedure from (3-bromopropyl)benzene (118.8 mg, 0.6 mmol) and 2-bromo-*N*-(thiophen-3-yl)butanamide (49.4 mg, 0.2 mmol). The product was purified by flash chromatography (10% ethyl acetate/hexanes). White solid.

38.1 mg (67% yield), 94% ee.

The ee was determined by HPLC on a Daicel CHIRALPAK® OD-H column (10% *i*-PrOH/hexanes, 1.0 mL/min); retention times for compound obtained using **(*S,S*)-L20**: 13.8 min (major), 16.9 min (minor).

$[\alpha]_D^{25}$ = −9.7 (*c* = 0.4, $CHCl_3$).

^{1}H NMR (600 MHz, Chloroform-*d*) δ 7.60 (dd, *J* = 3.2, 1.3 Hz, 1H), 7.41 (s, 1H), 7.26 (t, *J* = 7.5 Hz, 2H), 7.21 (dd, *J* = 5.1, 3.3 Hz, 1H), 7.19 – 7.17 (m, 1H), 7.16 – 7.14 (m, 2H), 6.98 (d, *J* = 5.1 Hz, 1H), 2.70 – 2.54 (m, 2H), 2.10 – 2.05 (m, 1H), 1.78 – 1.60 (m, 4H), 1.58 – 1.51 (m, 2H), 0.93 (t, *J* = 7.4 Hz, 3H).

^{13}C NMR (101 MHz, $CDCl_3$) δ 173.5, 142.1, 135.5, 128.4, 128.3, 125.8, 124.4, 121.0, 110.2, 50.0, 35.9, 32.5, 29.5, 26.2, 12.1.

HRMS (ESI) m/z $(M+H)^+$ calcd for $C_{17}H_{22}NOS$: 288.1417, found: 288.1420.

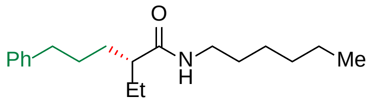

(*R*)-2-Ethyl-*N*-hexyl-5-phenylpentanamide (3s). The title compound was synthesized according to General procedure from (3-bromopropyl)benzene (118.8 mg, 0.6 mmol) and 2-bromo-*N*-hexylbutanamide (49.8 mg, 0.2 mmol). The product was purified by flash chromatography (10% ethyl acetate/hexanes). White solid. 27.8 mg (48% yield), 87% ee.

The ee was determined by HPLC on a Daicel CHIRALPAK® OD-H column (10% *i*-PrOH/hexanes, 1.0 mL/min); retention times for compound obtained using **(*S,S*)-L20**: 5.6 min (minor), 6.0 min (major).

$[\alpha]_D^{25}$ = −0.1 (*c* = 0.3, $CHCl_3$).

^{1}H NMR (600 MHz, Chloroform-*d*) δ 7.24 – 7.14 (m, 2H), 7.14 – 7.01 (m, 3H), 5.35 (t, *J* = 5.8 Hz, 1H), 3.17 (ddt, *J* = 16.0, 13.3, 6.1 Hz, 2H), 2.60 – 2.44 (m, 2H), 1.81 (tt, *J* = 9.4, 5.0 Hz, 1H), 1.65 – 1.45 (m, 4H), 1.45 – 1.31 (m, 4H), 1.25 – 1.18 (m, 6H), 0.80 (td, *J* = 7.2, 2.1 Hz, 6H).

^{13}C NMR (101 MHz, $CDCl_3$) δ 175.5, 142.3, 128.4, 128.3, 125.7, 49.8, 39.3, 36.0, 32.5, 31.4, 29.7, 29.6, 26.6, 26.1, 22.5, 14.0, 12.1.

HRMS (ESI) m/z $(M+H)^+$ calcd for $C_{19}H_{32}NO$: 290.2478, found: 290.2481.

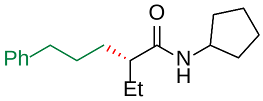

(*R*)-*N*-Cyclopentyl-2-ethyl-5-phenylpentanamide (3t). The title compound was synthesized according to General procedure from (3-bromopropyl)benzene (118.8 mg, 0.6 mmol) and 2-bromo-*N*-cyclopentylbutanamide (46.6 mg, 0.2 mmol). The product was purified by flash chromatography (20% ethyl acetate/hexanes). White solid. 33.3 mg (61% yield), 88% ee.

The ee was determined by HPLC on a Daicel CHIRALPAK® AS-H column (5% *i*-PrOH/hexanes, 0.5 mL/min); retention times for compound obtained using **(*S,S*)-L20**: 21.4 min (minor), 22.9 min (major).

$[\alpha]_D^{25} = -6.3$ (c = 0.4, $CHCl_3$).

^{1}H NMR (400 MHz, Chloroform-*d*) δ 7.20 (tt, *J* = 7.9, 1.4 Hz, 2H), 7.15 – 7.00 (m, 3H), 5.36 – 5.15 (m, 1H), 4.15 (h, *J* = 7.0 Hz, 1H), 2.63 – 2.43 (m, 2H), 1.91 (dddd, *J* = 17.6, 8.7, 4.8, 2.3 Hz, 2H), 1.77 (td, *J* = 9.1, 4.8 Hz, 1H), 1.65 – 1.43 (m, 8H), 1.44 – 1.23 (m, 4H), 0.79 (t, *J* = 7.4 Hz, 3H).

^{13}C NMR (101 MHz, $CDCl_3$) δ 175.1, 142.3, 128.34, 128.25, 125.7, 50.9, 49.7, 36.0, 33.2, 33.2, 32.5, 29.5, 26.1, 23.7, 12.1.

HRMS (ESI) m/z $(M+H)^+$ calcd for $C_{18}H_{28}NO$: 274.2165, found: 274.2168.

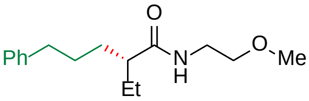

(*R*)-2-Ethyl-*N*-(2-methoxyethyl)-5-phenylpentanamide (3u). The title compound was synthesized according to General procedure from (3-bromopropyl)benzene (118.8 mg, 0.6 mmol) and 2-bromo-*N*-(2-methoxyethyl)butanamide (44.6 mg, 0.2 mmol). The product was purified by flash chromatography (20% ethyl acetate/hexanes). Colorless oil. 21.0 mg (39% yield), 82% ee.

The ee was determined by HPLC on a Daicel CHIRALPAK® OD-H column (10% *i*-PrOH/hexanes, 1.0 mL/min); retention times for compound obtained using **(*S,S*)-L20**: 6.4 min (minor), 7.6 min (major).

$[\alpha]_D^{25} = -4.4$ (c = 0.3, $CHCl_3$).

^1H NMR (400 MHz, Chloroform-*d*) δ 7.28 – 7.24 (m, 2H), 7.20 – 7.12 (m, 3H), 5.78 (s, 1H), 3.51 – 3.38 (m, 4H), 3.32 (s, 3H), 2.59 (tt, *J* = 10.0, 5.0 Hz, 2H), 1.93 (tt, *J* = 9.2, 5.0 Hz, 1H), 1.68 – 1.57 (m, 4H), 1.51 – 1.40 (m, 2H), 0.87 (t, *J* = 7.4 Hz, 3H).

^{13}C NMR (101 MHz, $CDCl_3$) δ 175.7, 142.3, 128.4, 128.3, 125.7, 71.4, 58.7, 49.6, 38.9, 36.0, 32.4, 29.5, 26.0, 12.0.

HRMS (ESI) m/z $(M+H)^+$ calcd for $C_{16}H_{26}NO_2$: 264.1958, found: 264.1958.

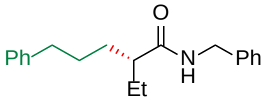

(*R*)-*N*-Benzyl-2-ethyl-5-phenylpentanamide (3v). The title compound was synthesized according to General procedure from (3-bromopropyl)benzene (118.8 mg, 0.6 mmol) and *N*-benzyl-2-bromobutanamide (51.0 mg, 0.2 mmol). The product was purified by flash chromatography (10% ethyl acetate/hexanes). White solid. 32.2 mg (54% yield), 84% ee.

The ee was determined by HPLC on a Daicel CHIRALPAK® OD-H column (10% *i*-PrOH/hexanes, 1.0 mL/min); retention times for compound obtained using **(*S,S)*-L20**: 12.9 min (major), 15.1 min (minor).

$[\alpha]_D^{25} = -1.2$ (c = 0.4, $CHCl_3$).

^1H NMR (600 MHz, Chloroform-*d*) δ 7.25 (t, *J* = 7.3 Hz, 2H), 7.19 (t, *J* = 6.9 Hz, 5H), 7.10 (t, *J* = 7.4 Hz, 1H), 7.06 (d, *J* = 7.5 Hz, 2H), 5.60 (s, 1H), 4.37 (q, *J* = 9.2, 7.4 Hz, 2H), 2.61 – 2.44 (m, *J* = 7.6 Hz, 2H), 1.88 (s, 1H), 1.67 – 1.49 (m, 4H), 1.41 (dq, *J* = 13.2, 6.2 Hz, 2H), 0.82 (t, *J* = 7.3 Hz, 3H).

^{13}C NMR (151 MHz, $CDCl_3$) δ 175.5, 142.2, 138.5, 128.6, 128.32, 128.26, 127.8, 127.4, 125.7, 77.0, 49.7, 43.4, 35.9, 32.5, 29.5, 26.1, 12.1.

HRMS (ESI) m/z $(M+H)^+$ calcd for $C_{20}H_{26}NO$: 296.2009, found: 296.2013.

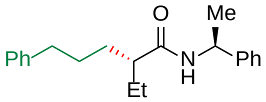

(*R*)-2-Ethyl-5-phenyl-*N*-((*R*)-1-phenylethyl)pentanamide (3w). The title

compound was synthesized according to General procedure from (3-bromopropyl)benzene (118.8 mg, 0.6 mmol) and 2-bromo-*N*-(1-phenylethyl)butanamide (53.8 mg, 0.2 mmol). The product was purified by flash chromatography (20% ethyl acetate/hexanes). White solid. 39.0 mg (63% yield), 9:1 dr.

^{1}H NMR (600 MHz, Chloroform-*d*) δ 7.25 – 7.15 (m, 7H), 7.09 (t, *J* = 7.7 Hz, 1H), 7.01 (d, *J* = 7.2 Hz, 2H), 5.63 – 5.48 (m, 1H), 5.11 (p, *J* = 6.3 Hz, 1H), 2.48 (h, *J* = 6.6 Hz, 2H), 1.83 (s, 1H), 1.64 – 1.47 (m, 4H), 1.40 (dd, *J* = 21.4, 6.5 Hz, 5H), 0.83 (t, *J* = 7.2 Hz, 3H).

^{13}C NMR (151 MHz, $CDCl_3$) δ 174.6, 143.3, 142.2, 128.6, 128.3, 128.2, 127.3, 126.2, 125.6, 49.6, 48.4, 35.8, 32.5, 29.4, 26.1, 21.6, 12.1.

HRMS (ESI) m/z $(M+H)^+$ calcd for $C_{21}H_{28}NO$: 310.2165, found: 310.2196.

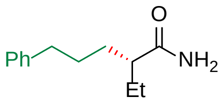

(*R*)-2-Ethyl-5-phenylpentanamide (3x). The title compound was synthesized according to General procedure from (3-bromopropyl)benzene (118.8 mg, 0.6 mmol) and 2-bromobutanamide (33.0 mg, 0.2 mmol). The product was purified by flash chromatography (33% ethyl acetate/hexanes). White solid. 20.2 mg (49% yield), 87% ee.

The ee was determined by HPLC on a Daicel CHIRALPAK® OD-H column (10% *i*-PrOH/hexanes, 1.0 mL/min); retention times for compound obtained using **(*S*,*S*)-L20**: 11.0 min (major), 14.6 min (minor).

$[\alpha]_D^{25} = -2.7$ (*c* = 0.3, $CHCl_3$).

^{1}H NMR (400 MHz, Chloroform-*d*) δ 7.25 – 7.17 (m, 2H), 7.16 – 7.02 (m, 3H), 5.40 (d, *J* = 58.5 Hz, 2H), 2.62 – 2.45 (m, 2H), 1.97 (tt, *J* = 8.8, 5.1 Hz, 1H), 1.65 – 1.36 (m, 6H), 0.85 (t, *J* = 7.4 Hz, 3H).

^{13}C NMR (101 MHz, $CDCl_3$) δ 178.2, 142.2, 128.4, 128.3, 125.8, 48.8, 35.9, 32.3, 29.4, 26.0, 12.0.

HRMS (ESI) m/z $(M+H)^+$ calcd for $C_{13}H_{20}NO$: 206.1539, found: 206.1543.

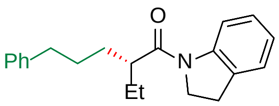

(*R*)-2-Ethyl-1-(indolin-1-yl)-5-phenylpentan-1-one (3y). The title compound was synthesized according to General procedure from (3-bromopropyl)benzene (118.8 mg, 0.6 mmol) and 2-bromo-1-(indolin-1-yl)butan-1-one (53.4 mg, 0.2 mmol). The product was purified by flash chromatography (10% ethyl acetate/hexanes). White solid. 32.0 mg (52% yield), 92% ee.

The ee was determined by HPLC on a Daicel CHIRALPAK® OD-H column (5% *i*-PrOH/hexanes, 1.0 mL/min); retention times for compound obtained using**(*S,S*)-L20**: 19.2 min (major), 21.6 min (minor).

$[\alpha]_D^{25} = -9.6$ (c = 0.4, $CHCl_3$).

^{1}H NMR (400 MHz, Chloroform-*d*) δ 8.24 (d, J = 8.1 Hz, 1H), 7.21 – 7.04 (m, 7H), 6.93 (t, J = 7.4 Hz, 1H), 4.07 – 3.95 (m, 2H), 3.09 (t, J = 8.5 Hz, 2H), 2.61 – 2.43 (m, 3H), 1.77 – 1.47 (m, 6H), 0.86 (t, J = 7.4 Hz, 3H).

^{13}C NMR (101 MHz, Chloroform-*d*) δ 174.7, 143.1, 142.2, 131.3, 128.4, 128.3, 127.5, 125.7, 124.5, 123.7, 117.4, 48.2, 46.4, 36.1, 32.4, 29.6, 27.9, 26.0, 12.1.

HRMS (ESI) m/z $(M+H)^+$ calcd for $C_{21}H_{26}NO$: 308.2009, found: 308.2007.

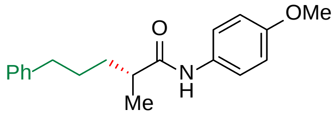

(*R*)-*N*-(4-Methoxyphenyl)-2-methyl-5-phenylpentanamide (4a from 2-chloro-*N*-(4-methoxyphenyl)propanamide) (4a). The title compound was synthesized according to General procedure from (3-bromopropyl)benzene (118.8 mg, 0.6 mmol) and 2-chloro-*N*-(4-methoxyphenyl)propanamide (42.6 mg, 0.2 mmol). The product was purified by flash chromatography (15% ethyl acetate/hexanes). White solid. 36.1 mg (61% yield), 92% ee.

The ee was determined by HPLC on a Daicel CHIRALPAK® OD-H column (10% *i*-PrOH/hexanes, 1.0 mL/min); retention times for compound obtained using **(*S,S*)-L20**: 25.0 min (minor), 33.0 min (major).

$[\alpha]_D^{25} = -45.2$ (c = 0.5, $CHCl_3$).

^{1}H NMR (400 MHz, Chloroform-*d*) δ 7.41 (d, *J* = 7.0 Hz, 2H), 7.27 (t, *J* = 7.3 Hz, 2H), 7.18 (t, *J* = 8.2 Hz, 3H), 7.08 (s, 1H), 6.84 (d, *J* = 9.0 Hz, 2H), 3.78 (s, 3H), 2.69 – 2.56 (m, 2H), 2.34 – 2.25 (m, 1H), 1.86 – 1.74 (m, 1H), 1.71 – 1.65 (m, 2H), 1.58 – 1.46 (m, 1H), 1.22 (d, *J* = 6.8 Hz, 3H).

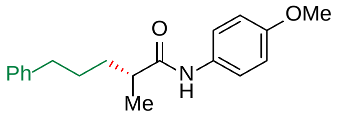

(*R*)-*N*-(4-Methoxyphenyl)-2-methyl-5-phenylpentanamide (4a from 2-bromo-*N*-(4-methoxyphenyl)propanamide) (4a). The title compound was synthesized according to General procedure from (3-bromopropyl)benzene (118.8 mg, 0.6 mmol) and 2-bromo-*N*-(4-methoxyphenyl)propanamide (51.4 mg, 0.2 mmol). The product was purified by flash chromatography (15% ethyl acetate/hexanes). White solid. 41.3 mg (69% yield), 94% ee.

The ee was determined by HPLC on a Daicel CHIRALPAK® AS-H column (10% *i*-PrOH/hexanes, 1.0 mL/min); retention times for compound obtained using **(*S,S*)-L20**: 19.0 min (minor), 29.0 min (major).

$[\alpha]_D^{25} = -45.4$ (c = 0.5, $CHCl_3$).

^{1}H NMR (400 MHz, Chloroform-*d*) δ 7.39 – 7.27 (m, 2H), 7.19 (t, *J* = 7.3 Hz, 2H), 7.13 – 7.04 (m, 3H), 6.83 – 6.66 (m, 2H), 3.69 (s, 3H), 2.70 – 2.41 (m, *J* = 7.6 Hz, 2H), 2.22 (h, *J* = 6.8 Hz, 1H), 1.75 – 1.56 (m, 3H), 1.42 (dt, *J* = 16.5, 6.1 Hz, 1H), 1.13 (d, *J* = 6.8 Hz, 3H).

^{13}C NMR (101 MHz, $CDCl_3$) δ 174.5, 156.3, 142.2, 131.0, 128.4, 128.3, 125.8, 121.7, 114.1, 55.5, 42.4, 35.9, 34.0, 29.3, 17.9.

HRMS (ESI) m/z $(M+H)^+$ calcd for $C_{19}H_{24}NO_2$: 298.1802, found: 298.1802.

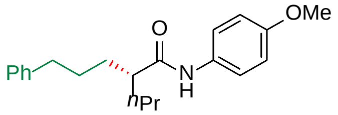

(*R*)-*N*-(4-Methoxyphenyl)-5-phenyl-2-propylpentanamide (4b). The title compound was synthesized according to General procedure from (3-bromopropyl)benzene (118.8 mg, 0.6 mmol) and 2-bromo-*N*-(4-methoxyphenyl)pentanamide (57.0 mg, 0.2 mmol). The product was purified by flash

chromatography (15% ethyl acetate/hexanes). White solid. 41.8 mg (65% yield), 94% ee.

The ee was determined by HPLC on a Daicel CHIRALPAK® AS-H column (10% *i*-PrOH/hexanes, 1.0 mL/min); retention times for compound obtained using **(*S*,*S*)-L20**: 10.8 min (minor), 14.5 min (major).

$[\alpha]_D^{25} = -44.4$ (c = 0.5, $CHCl_3$).

^1H NMR (400 MHz, Chloroform-*d*) δ 7.38 – 7.29 (m, 2H), 7.22 – 7.16 (m, 2H), 7.14 – 7.00 (m, 4H), 6.85 – 6.66 (m, 2H), 3.70 (s, 3H), 2.62 – 2.46 (m, J = 7.0 Hz, 2H), 2.07 (tt, J = 9.3, 4.9 Hz, 1H), 1.72 – 1.53 (m, 5H), 1.48 – 1.21 (m, 4H), 0.82 (t, J = 7.2 Hz, 3H).

^{13}C NMR (101 MHz, $CDCl_3$) δ 174.1, 156.3, 142.2, 130.9, 128.4, 128.32, 128.25, 125.7, 121.8, 114.0, 55.4, 48.5, 36.0, 35.4, 32.8, 29.5, 20.8, 14.1.

HRMS (ESI) m/z $(M+H)^+$ calcd for $C_{21}H_{28}NO_2$: 326.2115, found: 326.2115.

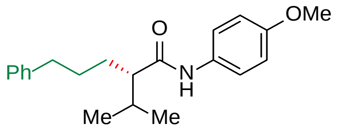

(*S*)-2-Isopropyl-*N*-(4-methoxyphenyl)-5-phenylpentanamide (4c). The title compound was synthesized according to General procedure from (3-bromopropyl)benzene (118.8 mg, 0.6 mmol) and 2-bromo-*N*-(4-methoxyphenyl)-3-methylbutanamide (57.0 mg, 0.2 mmol). The product was purified by flash chromatography (20% ethyl acetate/hexanes). White solid. 32.0 mg (50% yield), 89% ee.

The ee was determined by HPLC on a Daicel CHIRALPAK® AS-H column (10% *i*-PrOH/hexanes, 1.0 mL/min); retention times for compound obtained using **(*S*,*S*)-L20**: 8.7 min (minor), 16.7 min (major).

$[\alpha]_D^{25} = -17.1$ (c = 0.4, $CHCl_3$).

^1H NMR (400 MHz, Chloroform-*d*) δ 7.37 – 7.27 (m, 2H), 7.18 (t, J = 7.4 Hz, 2H), 7.13 – 7.05 (m, 3H), 6.99 (s, 1H), 6.80 – 6.71 (m, 2H), 3.70 (s, 3H), 2.55 (t, J = 6.5 Hz, 2H), 1.87 – 1.73 (m, 2H), 1.71 – 1.62 (m, 2H), 1.58 – 1.46 (m, 2H), 0.90 (d, J = 6.3 Hz, 6H).

^{13}C NMR (101 MHz, $CDCl_3$) δ 173.6, 156.4, 142.2, 130.9, 128.4, 128.3, 125.7, 121.9, 114.07, 55.9, 55.5, 36.1, 31.2, 30.0, 29.8, 20.9, 20.5.

HRMS (ESI) m/z $(M+H)^+$ calcd for $C_{21}H_{28}NO_2$: 326.2115, found: 326.2114.

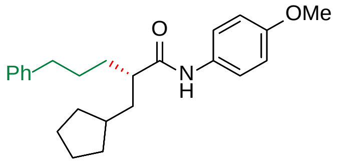

(*S*)-2-(Cyclopentylmethyl)-*N*-(4-methoxyphenyl)-5-phenylpentanamide (4d). The title compound was synthesized according to General procedure from (3-bromopropyl)benzene (118.8 mg, 0.6 mmol) and 2-bromo-3-cyclopentyl-*N*-(4-methoxyphenyl)propanamide (65.0 mg, 0.2 mmol). The product was purified by flash chromatography (15% ethyl acetate/hexanes). White solid. 45.9 mg (63% yield), 90% ee.

The ee was determined by HPLC on a Daicel CHIRALPAK® OD-H column (10% *i*-PrOH/hexanes, 1.0 mL/min); retention times for compound obtained using **(*S*,*S*)-L20**: 15.6 min (major), 27.0 min (minor).

$[\alpha]_D^{25} = -3.5$ (c = 0.6, $CHCl_3$).

^{1}H NMR (400 MHz, Chloroform-*d*) δ 7.32 (d, J = 9.0 Hz, 2H), 7.18 (t, J = 7.3 Hz, 2H), 7.14 – 7.00 (m, 4H), 6.75 (d, J = 9.0 Hz, 2H), 3.70 (s, 3H), 2.60 – 2.45 (m, J = 7.3 Hz, 2H), 2.11 (tt, J = 9.2, 4.9 Hz, 1H), 1.76 – 1.39 (m, 13H), 1.01 (m, 2H).

^{13}C NMR (101 MHz, $CDCl_3$) δ 174.2, 156.3, 142.2, 131.0, 128.4, 128.3, 125.7, 121.8, 114.0, 55.5, 48.0, 39.6, 38.1, 36.0, 33.3, 33.1, 32.5, 29.4, 25.1, 25.1.

HRMS (ESI) m/z $(M+H)^+$ calcd for $C_{24}H_{32}NO_2$: 366.2428, found: 366.2428.

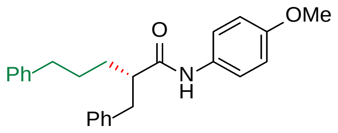

(*S*)-2-Benzyl-*N*-(4-methoxyphenyl)-5-phenylpentanamide (4e). The title compound was synthesized according to General procedure from (3-bromopropyl)benzene (118.8 mg, 0.6 mmol) and 2-bromo-*N*-(4-methoxyphenyl)-3-phenylpropanamide (66.6 mg, 0.2 mmol). The product was purified by flash chromatography (15% ethyl acetate/hexanes). White solid. 50.2 mg (67% yield), 91%

ee.

The ee was determined by HPLC on a Daicel CHIRALPAK® AS-H column (10% *i*-PrOH/hexanes, 1.0 mL/min); retention times for compound obtained using **(*S,S*)-L20**: 17.7 min (major), 25.3 min (minor).

$[\alpha]_D^{25}$ = +46.9 (*c* = 0.7, $CHCl_3$).

^1H NMR (400 MHz, Chloroform-*d*) δ 7.21 – 7.03 (m, 12H), 6.69 (d, *J* = 9.0 Hz, 3H), 3.67 (s, 3H), 2.88 (dd, *J* = 13.4, 9.5 Hz, 1H), 2.69 (dd, *J* = 13.4, 5.3 Hz, 1H), 2.59 – 2.48 (m, 2H), 2.33 – 2.25 (m, 1H), 1.88 – 1.74 (m, 1H), 1.69 – 1.47 (m, 3H).

^{13}C NMR (101 MHz, $CDCl_3$) δ 173.0, 156.4, 142.1, 139.7, 130.5, 128.9, 128.5, 128.4, 128.3, 126.4, 125.8, 122.2, 113.9, 55.4, 51.1, 39.5, 35.9, 32.5, 29.5.

HRMS (ESI) m/z $(M+H)^+$ calcd for $C_{25}H_{28}NO_2$: 374.2115, found: 374.2114.

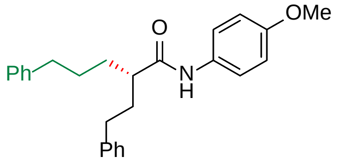

(*S*)-*N*-(4-Methoxyphenyl)-2-phenethyl-5-phenylpentanamide (4f). The title compound was synthesized according to General procedure from (3-bromopropyl)benzene (118.8 mg, 0.6 mmol) and 2-bromo-*N*-(4-methoxyphenyl)-4-phenylbutanamide (69.4 mg, 0.2 mmol). The product was purified by flash chromatography (10% ethyl acetate/hexanes). White solid. 50.7 mg (65% yield), 91% ee.

The ee was determined by HPLC on a Daicel CHIRALPAK® AD-H column (10% *i*-PrOH/hexanes, 1.0 mL/min); retention times for compound obtained using **(*S,S*)-L20**: 23.8 min (minor), 38.8 min (major).

$[\alpha]_D^{25}$ = +20.4 (*c* = 0.7, $CHCl_3$).

^1H NMR (400 MHz, Chloroform-*d*) δ 7.40 – 7.25 (m, 2H), 7.25 – 6.96 (m, 11H), 6.81 – 6.68 (m, 2H), 3.69 (s, 3H), 2.63 (ddd, *J* = 13.9, 9.0, 5.0 Hz, 1H), 2.48 (dt, *J* = 13.8, 7.6 Hz, 3H), 2.11 – 1.91 (m, 2H), 1.77 – 1.65 (m, 2H), 1.60 – 1.39 (m, 3H).

^{13}C NMR (101 MHz, $CDCl_3$) δ 173.6, 156.4, 142.1, 141.5, 130.8, 128.41, 128.36, 128.3, 126.0, 125.8, 121.8, 114.1, 55.5, 47.6, 35.9, 34.2, 33.5, 32.8, 29.3.

HRMS (ESI) m/z $(M+H)^+$ calcd for $C_{26}H_{30}NO_2$: 388.2271, found: 388.2271.

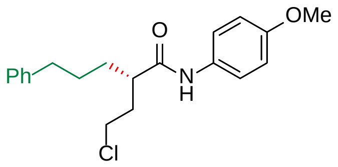

(*S*)-2-(2-Chloroethyl)-*N*-(4-methoxyphenyl)-5-phenylpentanamide (4g). The title compound was synthesized according to General procedure from (3-bromopropyl)benzene (118.8 mg, 0.6 mmol) and 2-bromo-4-chloro-*N*-(4-methoxyphenyl)butanamide (61.0 mg, 0.2 mmol). The product was purified by flash chromatography (15% ethyl acetate/hexanes). White solid. 40.1 mg (61% yield), 92% ee.

The ee was determined by HPLC on a Daicel CHIRALPAK® AS-H column (10% *i*-PrOH/hexanes, 1.0 mL/min); retention times for compound obtained using **(*S*,*S*)-L20**: 17.8 min (minor), 29.4 min (major).

$[\alpha]_D^{25}$ = +2.7 (*c* = 0.6, $CHCl_3$).

^{1}H NMR (400 MHz, Chloroform-*d*) δ 7.37 – 7.29 (m, 2H), 7.21 (d, *J* = 7.1 Hz, 2H), 7.11 (dt, *J* = 11.4, 5.5 Hz, 4H), 6.82 – 6.74 (m, 2H), 3.71 (s, 3H), 3.58 (dt, *J* = 10.3, 5.1 Hz, 1H), 3.48 – 3.37 (m, 1H), 2.57 (tt, *J* = 14.1, 6.6 Hz, 2H), 2.44 (tt, *J* = 9.4, 4.7 Hz, 1H), 2.11 (tt, *J* = 9.8, 4.7 Hz, 1H), 1.80 (dtd, *J* = 18.0, 9.0, 8.0, 3.8 Hz, 2H), 1.68 – 1.57 (m, 2H), 1.46 (ddd, *J* = 12.6, 10.4, 6.4 Hz, 1H).

^{13}C NMR (101 MHz, $CDCl_3$) δ 172.7, 156.5, 141.9, 130.6, 128.42, 12836, 125.9, 121.9, 114.1, 55.5, 45.0, 43.4, 35.8, 35.2, 32.3, 29.2.

HRMS (ESI) m/z $(M+H)^+$ calcd for $C_{20}H_{25}ClNO_2$: 346.1568, found: 346.1567.

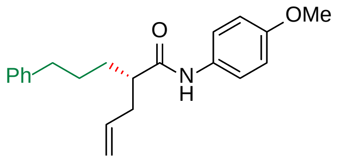

(*S*)-*N*-(4-Methoxyphenyl)-2-(3-phenylpropyl)pent-4-enamide (4h). The title compound was synthesized according to General procedure from (3-bromopropyl)benzene (118.8 mg, 0.6 mmol) and 2-bromo-*N*-(4-methoxyphenyl)pent-4-enamide (56.6 mg, 0.2 mmol). The product was purified by flash chromatography (10% ethyl acetate/hexanes). White solid. 38.1 mg (58% yield), 91% ee.

The ee was determined by HPLC on a Daicel CHIRALPAK® AS-H column (10% *i*-

PrOH/hexanes, 1.0 mL/min); retention times for compound obtained using **(*S*,*S*)-L20**: 14.6 min (minor), 19.9 min (major).

$[\alpha]_D^{25}$ = −4.3 (*c* = 0.6, $CHCl_3$).

^{1}H NMR (600 MHz, Chloroform-*d*) δ 7.35 – 7.26 (m, 2H), 7.22 – 7.14 (m, 2H), 7.13 – 7.02 (m, 4H), 6.80 – 6.71 (m, 2H), 5.70 (ddd, *J* = 17.2, 10.1, 7.3 Hz, 1H), 4.98 (dd, *J* = 37.3, 13.6 Hz, 2H), 3.70 (s, 3H), 2.54 (dq, *J* = 15.5, 7.1 Hz, 2H), 2.36 (dt, *J* = 13.8, 7.6 Hz, 1H), 2.15 (ddt, *J* = 13.8, 8.7, 5.4 Hz, 2H), 1.74 – 1.63 (m, 2H), 1.56 (dq, *J* = 17.1, 6.1 Hz, 1H), 1.53 – 1.44 (m, 1H).

^{13}C NMR (151 MHz, $CDCl_3$) δ 173.2, 156.4, 142.1, 135.6, 130.8, 128.37, 128.35, 128.3, 125.8, 121.9, 117.0, 114.1, 55.5, 48.4, 37.2, 35.9, 32.2, 29.3.

HRMS (ESI) m/z $(M+H)^+$ calcd for $C_{21}H_{26}NO_2$: 324.1958, found: 324.1958.

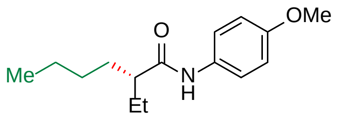

(*R*)-2-Ethyl-*N*-(4-methoxyphenyl)hexanamide (5a). The title compound was synthesized according to General procedure from 1-iodobutane (110.4 mg, 0.6 mmol) and 2-bromo-*N*-(4-methoxyphenyl)butanamide (54.2 mg, 0.2 mmol). The product was purified by flash chromatography (5% ethyl acetate/hexanes). White solid. 33.1 mg (66% yield), 90% ee.

The ee was determined by HPLC on a Daicel CHIRALPAK® AS-H column (10% *i*-PrOH/hexanes, 1.0 mL/min); retention times for compound obtained using **(*S*,*S*)-L20**: 8.7 min (minor), 10.5 min (major).

$[\alpha]_D^{25}$ = −2.7 (*c* = 0.6, $CHCl_3$).

^{1}H NMR (400 MHz, Chloroform-*d*) δ 7.44 – 7.26 (m, 2H), 7.10 (s, 1H), 6.87 – 6.65 (m, 2H), 3.71 (s, 3H), 1.99 (tt, *J* = 9.1, 5.1 Hz, 1H), 1.63 (dq, *J* = 13.9, 7.0, 6.6 Hz, 2H), 1.45 (dq, *J* = 21.0, 8.2, 7.6 Hz, 2H), 1.20 (s, 4H), 0.88 (t, *J* = 7.4 Hz, 3H), 0.81 (t, *J* = 6.7 Hz, 3H).

^{13}C NMR (101 MHz, $CDCl_3$) δ 174.2, 156.3, 131.1, 121.8, 114.1, 55.5, 50.6, 32.6, 29.9, 26.2, 22.8, 14.0, 12.1.

HRMS (ESI) m/z $(M+H)^+$ calcd for $C_{15}H_{24}NO_2$: 250.1802, found: 250.1801.

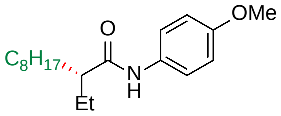

(*R*)-2-Ethyl-*N*-(4-methoxyphenyl)decanamide (5b). The title compound was synthesized according to General procedure from 1-bromooctane (115.2 mg, 0.6 mmol) and 2-bromo-*N*-(4-methoxyphenyl)butanamide (54.2 mg, 0.2 mmol). The product was purified by flash chromatography (10% ethyl acetate/hexanes). White solid. 36.2 mg (58% yield), 89% ee.

The ee was determined by HPLC on a Daicel CHIRALPAK® AS-H column (25% *i*-PrOH/hexanes, 1.0 mL/min); retention times for compound obtained using **(*S*,*S*)-L20**: 5.8 min (minor), 6.9 min (major).

$[\alpha]_D^{25} = -12.7$ (c = 0.4, $CHCl_3$).

^1H NMR (400 MHz, Chloroform-*d*) δ 7.56 – 7.34 (m, 2H), 7.27 (s, 1H), 6.90 – 6.68 (m, 2H), 3.78 (s, 3H), 2.07 (tt, *J* = 9.4, 5.1 Hz, 1H), 1.69 (dq, *J* = 14.6, 7.4 Hz, 2H), 1.50 (ddq, *J* = 28.3, 13.9, 6.3, 5.2 Hz, 2H), 1.38 – 1.15 (m, 12H), 0.94 (t, *J* = 7.4 Hz, 3H), 0.86 (t, *J* = 6.8 Hz, 3H).

^{13}C NMR (101 MHz, $CDCl_3$) δ 174.3, 156.3, 131.1, 121.8, 114.1, 55.5, 50.6, 32.9, 31.8, 29.7, 29.5, 29.3, 27.7, 26.2, 22.6, 14.1, 12.1.

HRMS (ESI) m/z $(M+H)^+$ calcd for $C_{19}H_{32}NO_2$: 306.2428, found: 306.2430.

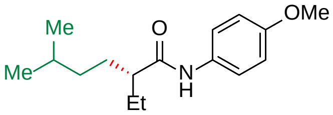

(*R*)-2-Ethyl-*N*-(4-methoxyphenyl)-5-methylhexanamide (5c). The title compound was synthesized according to General procedure from 1-bromo-3-methylbutane (90.0 mg, 0.6 mmol) and 2-bromo-*N*-(4-methoxyphenyl)butanamide (54.2 mg, 0.2 mmol). The product was purified by flash chromatography (15% ethyl acetate/hexanes). White solid. 25.0 mg (48% yield), 92% ee.

The ee was determined by HPLC on a Daicel CHIRALPAK® AS-H column (10% *i*-PrOH/hexanes, 1.0 mL/min); retention times for compound obtained using **(*S*,*S*)-L20**: 7.4 min (minor), 9.8 min (major).

$[\alpha]_D^{25} = -6.1$ (c = 0.3, $CHCl_3$).

^{1}H NMR (400 MHz, Chloroform-*d*) δ 7.44 (d, *J* = 9.0 Hz, 2H), 7.29 (s, 1H), 6.84 (d, *J* = 9.0 Hz, 2H), 3.78 (s, 3H), 2.04 (tt, *J* = 9.0, 5.1 Hz, 1H), 1.75 – 1.61 (m, 2H), 1.62 – 1.42 (m, 3H), 1.20 (dq, *J* = 10.4, 6.4, 6.0 Hz, 2H), 0.94 (t, *J* = 7.4 Hz, 3H), 0.86 (dd, *J* = 6.6, 3.0 Hz, 6H).

^{13}C NMR (101 MHz, $CDCl_3$) δ 174.2, 156.3, 131.1, 121.8, 114.0, 55.5, 50.8, 36.8, 30.7, 28.2, 26.2, 22.6, 22.5, 12.1.

HRMS (ESI) m/z $(M+H)^+$ calcd for $C_{16}H_{26}NO_2$: 264.1958, found: 264.1955.

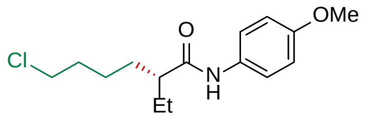

(*R*)-6-Chloro-2-ethyl-*N*-(4-methoxyphenyl)hexanamide (5d). The title compound was synthesized according to General procedure from 1-bromo-4-chlorobutane (102.0 mg, 0.6 mmol) and 2-bromo-*N*-(4-methoxyphenyl)butanamide (54.2 mg, 0.2 mmol). The product was purified by flash chromatography (15% ethyl acetate/hexanes). White solid. 35.9 mg (64% yield), 94% ee.

The ee was determined by HPLC on a Daicel CHIRALPAK® AS-H column (10% *i*-PrOH/hexanes, 1.0 mL/min); retention times for compound obtained using **(*S,S)*-L20**: 18.3 min (minor), 35.6 min (major).

$[\alpha]_D^{25} = -22.7$ (*c* = 0.4, $CHCl_3$).

^{1}H NMR (400 MHz, Chloroform-*d*) δ 7.43 (d, *J* = 8.9 Hz, 2H), 7.23 (s, 1H), 6.85 (d, *J* = 8.9 Hz, 2H), 3.78 (s, 3H), 3.64 – 3.40 (m, 2H), 2.08 (tt, *J* = 9.4, 4.6 Hz, 1H), 1.81 – 1.67 (m, 4H), 1.52 (ddd, *J* = 28.1, 13.8, 7.3 Hz, 4H), 0.95 (t, *J* = 7.4 Hz, 3H).

^{13}C NMR (101 MHz, $CDCl_3$) δ 173.9, 156.4, 130.9, 121.9, 114.1, 55.5, 50.3, 44.9, 32.6, 32.0, 26.2, 25.0, 12.0.

HRMS (ESI) m/z $(M+H)^+$ calcd for $C_{15}H_{23}ClNO_2$: 284.1412, found: 284.1409.

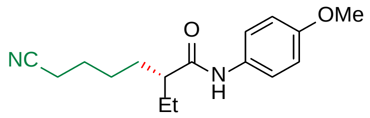

(*R*)-6-Cyano-2-ethyl-*N*-(4-methoxyphenyl)hexanamide (5e). The title compound was synthesized according to General procedure from 5-bromopentanenitrile (96.6 mg, 0.6 mmol) and 2-bromo-*N*-(4-methoxyphenyl)butanamide (54.2 mg, 0.2 mmol). The

product was purified by flash chromatography (25% ethyl acetate/hexanes). White solid. 38.0 mg (69% yield), 93% ee.

The ee was determined by HPLC on a Daicel CHIRALPAK® AS-H column (25% *i*-PrOH/hexanes, 1.0 mL/min); retention times for compound obtained using **(*S,S*)-L20**: 10.7 min (minor), 23.5 min (major).

$[\alpha]_D^{25} = -37.2$ (c = 0.4, $CHCl_3$).

^{1}H NMR (400 MHz, Chloroform-*d*) δ 7.41 – 7.32 (m, 2H), 7.16 (s, 1H), 6.84 – 6.73 (m, 2H), 3.72 (s, 3H), 2.35 – 2.17 (m, 2H), 2.02 (tt, J = 9.4, 4.8 Hz, 1H), 1.64 (ddt, J = 28.8, 14.3, 7.9 Hz, 5H), 1.53 – 1.38 (m, 4H), 0.89 (t, J = 7.4 Hz, 3H).

^{13}C NMR (101 MHz, $CDCl_3$) δ 173.7, 156.4, 130.9, 121.8, 119.7, 114.1, 55.5, 50.0, 31.8, 26.7, 26.3, 25.4, 17.0, 12.0.

HRMS (ESI) m/z $(M+H)^+$ calcd for $C_{16}H_{23}N_2O_2$: 275.1754, found: 275.1750.

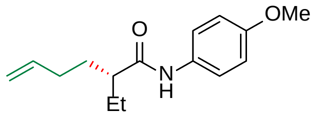

(*R*)-2-Ethyl-*N*-(4-methoxyphenyl)hex-5-enamide (5f). The title compound was synthesized according to General procedure from 4-bromobut-1-ene (80.4 mg, 0.6 mmol) and 2-bromo-*N*-(4-methoxyphenyl)butanamide (54.2 mg, 0.2 mmol). The product was purified by flash chromatography (15% ethyl acetate/hexanes). White solid. 28.8 mg (59% yield), 92% ee.

The ee was determined by HPLC on a Daicel CHIRALPAK® AS-H column (10% *i*-PrOH/hexanes, 1.0 mL/min); retention times for compound obtained using **(*S,S*)-L20**: 11.2 min (minor), 12.8 (majar).

$[\alpha]_D^{25} = -10.7$ (c = 0.3, $CHCl_3$).

^{1}H NMR (400 MHz, Chloroform-*d*) δ 7.50 – 7.36 (m, 2H), 7.18 (s, 1H), 6.90 – 6.76 (m, 2H), 5.80 (dddd, J = 17.4, 10.2, 7.3, 6.0 Hz, 1H), 5.10 – 4.89 (m, 2H), 3.78 (s, 3H), 2.28 – 1.93 (m, 3H), 1.83 – 1.68 (m, 2H), 1.65 – 1.48 (m, 2H), 0.95 (t, J = 7.4 Hz, 3H).

^{13}C NMR (101 MHz, $CDCl_3$) δ 173.9, 156.3, 138.3, 131.0, 121.8, 115.1, 114.0, 55.4, 49.4, 31.7, 31.7, 26.2, 12.0.

HRMS (ESI) m/z $(M+H)^+$ calcd for $C_{15}H_{22}NO_2$: 248.1645, found: 248.1643.

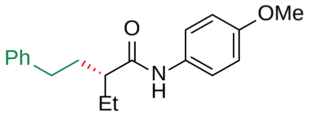

(*R*)-2-Ethyl-*N*-(4-methoxyphenyl)-4-phenylbutanamide (5g). The title compound was synthesized according to General procedure from (2-bromoethyl)benzene (110.4 mg, 0.6 mmol) and 2-bromo-*N*-(4-methoxyphenyl)butanamide (54.2 mg, 0.2 mmol). The product was purified by flash chromatography (15% ethyl acetate/hexanes). White solid. 31.0 mg (52% yield), 94% ee.

The ee was determined by HPLC on a Daicel CHIRALPAK® AS-H column (10% *i*-PrOH/hexanes, 1.0 mL/min); retention times for compound obtained using ***(S,S)*-L20**: 12.5 min (minor), 17.0 min (major).

$[\alpha]_D^{25}$ = −29.2 (c = 0.3, $CHCl_3$).

^{1}H NMR (400 MHz, Chloroform-*d*) δ 7.44 – 7.28 (m, 2H), 7.26 – 7.17 (m, 2H), 7.16 – 7.07 (m, 3H), 7.06 (s, 1H), 6.84 – 6.74 (m, 2H), 3.71 (s, 3H), 2.66 (ddd, J = 13.9, 9.5, 4.8 Hz, 1H), 2.58 – 2.44 (m, 1H), 1.99 (dhept, J = 13.2, 4.1 Hz, 2H), 1.81 – 1.62 (m, 2H), 1.55 – 1.39 (m, 1H), 0.86 (t, J = 7.4 Hz, 3H).

^{13}C NMR (101 MHz, $CDCl_3$) δ 173.7, 156.4, 141.6, 130.9, 128.42, 128.39, 126.0, 121.74, 121.72, 114.11, 114.07, 55.5, 49.4, 34.0, 33.6, 26.3, 12.0.

HRMS (ESI) m/z $(M+H)^+$ calcd for $C_{19}H_{24}NO_2$: 298.1802, found: 298.1806.

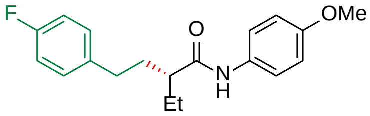

(*R*)-2-Ethyl-4-(4-fluorophenyl)-*N*-(4-methoxyphenyl)butanamide (5h). The title compound was synthesized according to General procedure from 1-(2-bromoethyl)-4-fluorobenzene (121.2 mg, 0.6 mmol) and 2-bromo-*N*-(4-methoxyphenyl)butanamide (54.2 mg, 0.2 mmol). The product was purified by flash chromatography (10% ethyl acetate/hexanes). White solid. 48.9 mg (79% yield), 94% ee.

The ee was determined by HPLC on a Daicel CHIRALPAK® OD-H column (10% *i*-PrOH/hexanes, 1.0 mL/min); retention times for compound obtained using ***(S,S)*-L20**: 14.7 min (major), 19.5 min (minor).

$[\alpha]_D^{25}$ = −36.6 (c = 0.6, $CHCl_3$).

^{1}H NMR (400 MHz, Chloroform-*d*) δ 7.52 – 7.37 (m, 2H), 7.27 (s, 1H), 7.11 (dd, *J* = 8.4, 5.5 Hz, 2H), 6.95 (t, *J* = 8.7 Hz, 2H), 6.89 – 6.79 (m, 2H), 3.78 (s, 3H), 2.75 – 2.48 (m, 2H), 2.04 (dtt, *J* = 23.4, 9.3, 4.9 Hz, 2H), 1.83 – 1.68 (m, 2H), 1.56 (dq, *J* = 13.4, 7.3, 6.1 Hz, 1H), 0.94 (t, *J* = 7.4 Hz, 3H).

^{13}C NMR (101 MHz, $CDCl_3$) δ 173.6, 161.3 (d, *J* = 242.2 Hz), 156.4, 137.2 (d, *J* = 3.3Hz), 130.9, 129.7 (d, *J* = 7.8Hz), 121.7, 115.1 (d, *J* = 20.9 Hz), 114.1, 55.5, 49.4, 34.2, 32.8, 26.3, 12.0.

^{19}F NMR (376 MHz, $CDCl_3$) δ -117.47.

HRMS (ESI) m/z $(M+H)^+$ calcd for $C_{19}H_{23}FNO_2$: 316.1707, found: 316.1709.

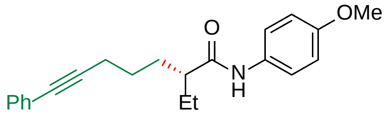

(*R*)-2-Ethyl-*N*-(4-methoxyphenyl)-7-phenylhept-6-ynamide (5i). The title compound was synthesized according to General procedure from (5-bromopent-1-yn-1-yl)benzene (133.2 mg, 0.6 mmol) and 2-bromo-*N*-(4-methoxyphenyl)butanamide (54.2 mg, 0.2 mmol). The product was purified by flash chromatography (15% ethyl acetate/hexanes). White solid. 37.1 mg (55% yield), 94% ee.

The ee was determined by HPLC on a Daicel CHIRALPAK® AS-H column (10% *i*-PrOH/hexanes, 1.0 mL/min); retention times for compound obtained using **(*S*,*S*)-L20**: 21.8 min (minor), 30.9 min (major).

$[\alpha]_D^{25} = -24.4$ (*c* = 0.5, $CHCl_3$).

^{1}H NMR (400 MHz, Chloroform-*d*) δ 7.42 – 7.26 (m, 4H), 7.23 – 7.10 (m, 4H), 6.84 – 6.70 (m, 2H), 3.70 (s, 3H), 2.47 – 2.26 (m, 2H), 2.19 – 2.03 (m, 1H), 1.86 – 1.44 (m, 7H), 0.90 (t, *J* = 7.4 Hz, 3H).

^{13}C NMR (101 MHz, $CDCl_3$) δ 173.8, 156.3, 131.5, 130.9, 128.2, 127.6, 123.8, 121.8, 114.1, 89.9, 81.0, 55.5, 49.9, 32.1, 26.6, 26.2, 19.5, 12.1.

HRMS (ESI) m/z $(M+H)^+$ calcd for $C_{22}H_{26}NO_2$: 336.1958, found: 336.1954.

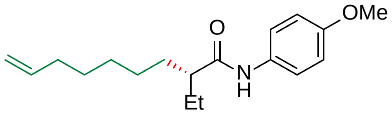

(*R*)-2-Ethyl-*N*-(4-methoxyphenyl)non-8-enamide (5j). The title compound was

synthesized according to General procedure from 7-bromohept-1-ene (105.6 mg, 0.6 mmol) and 2-bromo-*N*-(4-methoxyphenyl)butanamide (54.2 mg, 0.2 mmol). The product was purified by flash chromatography (10% ethyl acetate/hexanes). White solid. 25.2 mg (43% yield), 90% ee.

The ee was determined by HPLC on a Daicel CHIRALPAK® AD-H column (10% *i*-PrOH/hexanes, 1.0 mL/min); retention times for compound obtained using **(*S*,*S*)-L20**: 11.0 min (minor), 13.6 min (major).

$[\alpha]_D^{25} = -8.9$ (c = 0.3, $CHCl_3$).

^{1}H NMR (400 MHz, Chloroform-*d*) δ 7.41 – 7.29 (m, 2H), 7.15 (s, 1H), 6.81 – 6.73 (m, 2H), 5.71 (ddt, *J* = 16.9, 10.2, 6.7 Hz, 1H), 4.95 – 4.81 (m, 2H), 3.71 (s, 3H), 2.05 – 1.90 (m, 3H), 1.66 – 1.60 (m, 2H), 1.52 – 1.37 (m, 2H), 1.34 – 1.22 (m, 6H), 0.88 (t, *J* = 7.4 Hz, 3H).

^{13}C NMR (101 MHz, $CDCl_3$) δ 174.2, 156.3, 139.0, 131.0, 121.8, 114.2, 114.1, 55.47, 50.6, 33.7, 32.8, 29.2, 28.7, 27.5, 26.2, 12.1.

HRMS (ESI) m/z $(M+H)^+$ calcd for $C_{18}H_{28}NO_2$: 290.2115, found: 290.2113.

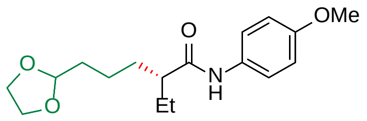

(*R*)-5-(1,3-Dioxolan-2-yl)-2-ethyl-*N*-(4-methoxyphenyl)pentanamide (5k). The title compound was synthesized according to General procedure from 2-(3-bromopropyl)-1,3-dioxolane (116.4 mg, 0.6 mmol) and 2-bromo-*N*-(4-methoxyphenyl)butanamide (54.2 mg, 0.2 mmol). The product was purified by flash chromatography (20% ethyl acetate/hexanes). White solid. 27.0 mg (44% yield), 92% ee.

The ee was determined by HPLC on a Daicel CHIRALPAK® AS-H column (25% *i*-PrOH/hexanes, 1.0 mL/min); retention times for compound obtained using **(*S*,*S*)-L20**: 8.4 min (minor), 9.8 min (major).

$[\alpha]_D^{25} = -13.5$ (c = 0.3, $CHCl_3$).

^{1}H NMR (400 MHz, Chloroform-*d*) δ 7.39 – 7.33 (m, 2H), 7.29 (s, 1H), 6.85 – 6.68 (m, 2H), 4.76 (t, *J* = 4.8 Hz, 1H), 3.91 – 3.82 (m, 2H), 3.82 – 3.73 (m, 2H), 3.71 (s,

3H), 2.01 (dq, J = 9.4, 4.6 Hz, 1H), 1.74 – 1.56 (m, 4H), 1.51 – 1.34 (m, 4H), 0.87 (t, J = 7.4 Hz, 3H).

^{13}C NMR (101 MHz, $CDCl_3$) δ 173.9, 156.3, 131.1, 121.8, 114.0, 104.3, 64.8, 55.4, 50.2, 33.7, 32.6, 26.1, 22.1, 12.6.

HRMS (ESI) m/z $(M+H)^+$ calcd for $C_{17}H_{26}NO_4$: 308.1856, found: 308.1854.

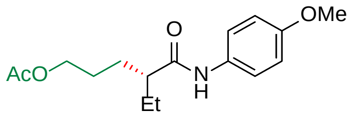

(*R*)-4-((4-Methoxyphenyl)carbamoyl)hexyl acetate (5l). The title compound was synthesized according to General procedure from 3-bromopropyl acetate (108.0 mg, 0.6 mmol) and 2-bromo-*N*-(4-methoxyphenyl)butanamide (54.2 mg, 0.2 mmol). The product was purified by flash chromatography (50% ethyl ether/hexanes). White solid. 43.1 mg (74% yield), 94% ee.

The ee was determined by HPLC on a Daicel CHIRALPAK® OD-H column (25% *i*-PrOH/hexanes, 1.0 mL/min); retention times for compound obtained using **(*S,S*)-L20**: 6.8 min (major), 10.9 min (minor).

$[\alpha]_D^{25} = -9.4$ (c = 0.5, $CHCl_3$).

^{1}H NMR (600 MHz, Chloroform-*d*) δ 7.49 – 7.39 (m, 2H), 7.33 (s, 1H), 6.90 – 6.79 (m, 2H), 4.07 (ddt, J = 33.1, 10.9, 6.5 Hz, 2H), 3.78 (d, J = 0.9 Hz, 3H), 2.12 (tt, J = 9.4, 5.0 Hz, 1H), 2.04 (s, 3H), 1.80 – 1.61 (m, 5H), 1.58 – 1.48 (m, 2H), 0.95 (t, J = 7.4 Hz, 3H).

^{13}C NMR (151 MHz, $CDCl_3$) δ 173.6, 171.3, 156.4, 130.9, 121.8, 114.1, 64.1, 55.5, 49.7, 29.0, 26.6, 26.1, 21.0, 12.0.

HRMS (ESI) m/z $(M+H)^+$ calcd for $C_{16}H_{24}NO_4$: 294.1705, found: 294.1700.

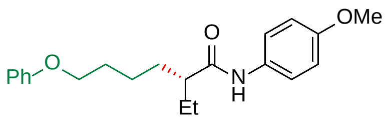

(*R*)-2-Ethyl-*N*-(4-methoxyphenyl)-6-phenoxyhexanamide (5m). The title compound was synthesized according to General procedure from (4-bromobutoxy)benzene (136.8 mg, 0.6 mmol) and 2-bromo-*N*-(4-

methoxyphenyl)butanamide (54.2 mg, 0.2 mmol). The product was purified by flash chromatography (15% ethyl ether/hexanes). White solid. 30.1 mg (44% yield), 92% ee.

The ee was determined by HPLC on a Daicel CHIRALPAK® AS-H column (10% *i*-PrOH/hexanes, 1.0 mL/min); retention times for compound obtained using **(*S,S)*-L20**: 18.2 min (minor), 27.7 min (major).

$[\alpha]_D^{25} = -33.1$ (c = 0.4, $CHCl_3$).

^{1}H NMR (400 MHz, Chloroform-*d*) δ 7.50 – 7.36 (m, 2H), 7.31 – 7.23 (m, 2H), 7.16 (s, 1H), 6.92 (tt, *J* = 7.4, 1.1 Hz, 1H), 6.90 – 6.76 (m, 4H), 4.06 – 3.85 (m, 2H), 3.78 (s, 3H), 2.10 (dt, *J* = 9.0, 4.5 Hz, 1H), 1.88 – 1.67 (m, 5H), 1.65 – 1.40 (m, 4H), 0.96 (t, *J* = 7.4 Hz, 3H).

^{13}C NMR (101 MHz, $CDCl_3$) δ 174.0, 158.9, 156.3, 130.9, 129.4, 121.9, 120.5, 114.4, 114.0, 67.5, 55.4, 50.3, 32.5, 29.3, 26.2, 24.2, 12.1.

HRMS (ESI) m/z $(M+H)^+$ calcd for $C_{21}H_{28}NO_3$: 342.2069, found: 342.2060.

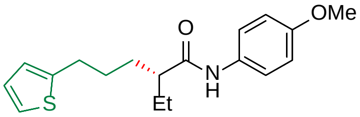

(*R*)-2-Ethyl-N-(4-methoxyphenyl)-5-(thiophen-2-yl)pentanamide (5n). The title compound was synthesized according to General procedure from 2-(3-bromopropyl)thiophene (122.4 mg, 0.6 mmol) and 2-bromo-*N*-(4-methoxyphenyl)butanamide (54.2 mg, 0.2 mmol). The product was purified by flash chromatography (15% ethyl acetate/hexanes). White solid. 46.2 mg (73% yield), 90% ee.

The ee was determined by HPLC on a Daicel CHIRALPAK® AS-H column (10% *i*-PrOH/hexanes, 1.0 mL/min); retention times for compound obtained using **(*S,S)*-L20**: 15.0 min (minor), 24.3 min (major).

$[\alpha]_D^{25} = -23.7$ (c = 0.6, $CHCl_3$).

^{1}H NMR (400 MHz, Chloroform-*d*) δ 7.47 – 7.37 (m, 2H), 7.24 (s, 1H), 7.10 (dd, *J* = 5.1, 1.2 Hz, 1H), 6.90 (dd, *J* = 5.1, 3.4 Hz, 1H), 6.88 – 6.80 (m, 2H), 6.79 – 6.73 (m, 1H), 3.78 (s, 3H), 2.91 – 2.76 (m, 2H), 2.15 – 2.02 (m, 1H), 1.81 – 1.63 (m, 4H), 1.63 – 1.50 (m, 2H), 0.94 (t, *J* = 7.4 Hz, 3H).

^{13}C NMR (101 MHz, $CDCl_3$) δ 173.9, 156.4, 145.0, 130.9, 126.7, 124.2, 122.9, 121.9, 114.06, 55.5, 50.2, 32.2, 29.9, 29.8, 26.2, 12.1.

HRMS (ESI) m/z $(M+H)^+$ calcd for C18H24NO2S: 318.1522, found: 318.1519.

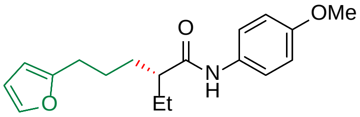

(*R*)-2-Ethyl-5-(furan-2-yl)-*N*-(4-methoxyphenyl)pentanamide (5o). The title compound was synthesized according to General procedure from 2-(3-bromopropyl)furan (112.8 mg, 0.6 mmol) and 2-bromo-*N*-(4-methoxyphenyl)butanamide (54.2 mg, 0.2 mmol). The product was purified by flash chromatography (10% ethyl acetate/hexanes). White solid. 36.8 mg (62% yield), 93% ee.

The ee was determined by HPLC on a Daicel CHIRALPAK® OD-H column (10% *i*-PrOH/hexanes, 1.0 mL/min); retention times for compound obtained using **(*S*,*S*)-L20**: 17.8 min (major), 24.2 min (minor).

$[\alpha]_D^{25} = -23.8$ (c = 0.4, $CHCl_3$).

^{1}H NMR (600 MHz, Chloroform-*d*) δ 7.42 (d, *J* = 8.9 Hz, 2H), 7.28 (d, *J* = 1.8 Hz, 1H), 7.22 (s, 1H), 6.84 (d, *J* = 8.9 Hz, 2H), 6.26 (t, *J* = 2.4 Hz, 1H), 5.97 (d, *J* = 3.1 Hz, 1H), 3.78 (s, 3H), 2.62 (hept, *J* = 7.5 Hz, 2H), 2.07 (dt, *J* = 9.1, 4.3 Hz, 1H), 1.73 (ddtd, *J* = 34.1, 15.4, 8.8, 7.7, 5.0 Hz, 5H), 1.53 (ddt, *J* = 14.9, 7.3, 5.2 Hz, 2H), 0.94 (t, *J* = 7.4 Hz, 3H).

^{13}C NMR (101 MHz, $CDCl_3$) δ 173.9, 156.4, 155.8, 140.7, 130.9, 121.9, 114.1, 110.1, 104.9, 55.5, 50.1, 32.3, 27.9, 26.2, 26.1, 12.0.

HRMS (ESI) m/z $(M+H)^+$ calcd for $C_{18}H_{24}NO_3$: 302.1751, found: 302.1751.

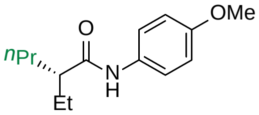

(*R*)-2-Ethyl-*N*-(4-methoxyphenyl)pentanamide (*R*-6). The title compound was synthesized according to General procedure from 1-iodopropane (102.0 mg, 0.6 mmol) and 2-bromo-*N*-(4-methoxyphenyl)butanamide (54.2 mg, 0.2 mmol). The product was

purified by flash chromatography (15% ethyl ether/hexanes). White solid. 33.1 mg (70% yield), 86% ee.

The ee was determined by HPLC on a Daicel CHIRALPAK® AS-H column (25% *i*-PrOH/hexanes, 1.0 mL/min); retention times for compound obtained using **(*S*,*S*)-L20**: 9.9 min (minor), 10.7 min (major).

$[\alpha]_D^{25} = -1.5$ (c = 0.4, $CHCl_3$).

^{1}H NMR (400 MHz, Chloroform-*d*) δ 7.42 (dd, *J* = 10.6, 8.3 Hz, 3H), 6.88 – 6.77 (m, 2H), 3.77 (s, 3H), 2.09 (tt, *J* = 9.4, 5.0 Hz, 1H), 1.68 (tdd, *J* = 12.3, 9.2, 5.9 Hz, 2H), 1.57 – 1.28 (m, 4H), 0.92 (dt, *J* = 13.7, 7.2 Hz, 6H).

^{13}C NMR (101 MHz, $CDCl_3$) δ 174.3, 156.3, 131.1, 121.9, 114.0, 55.4, 50.3, 35.1, 26.2, 20.8, 14.1, 12.1.

HRMS (ESI) m/z $(M+H)^+$ calcd for $C_{14}H_{22}NO_2$: 236.1645, found: 236.1643.

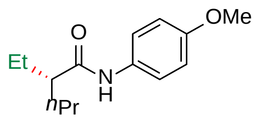

(*S*)-2-Ethyl-*N*-(4-methoxyphenyl)pentanamide (*S*-6). The title compound was synthesized according to General procedure from bromoethane (64.8 mg, 0.6 mmol) and 2-bromo-*N*-(4-methoxyphenyl)pentanamide (57.0 mg, 0.2 mmol). The product was purified by flash chromatography (20% ethyl ether/hexanes). White solid. 28.0 mg (59% yield), 88% ee.

The ee was determined by HPLC on a Daicel CHIRALPAK® AS-H column (25% *i*-PrOH/hexanes, 1.0 mL/min); retention times for compound obtained using **(*S*,*S*)-L20**: 9.9 min (major), 10.9 min (minor).

$[\alpha]_D^{25} = +0.2$ (c = 0.3, $CHCl_3$).

^{1}H NMR (400 MHz, Chloroform-*d*) δ 7.43 – 7.30 (m, 2H), 7.27 (s, 1H), 6.85 – 6.70 (m, 2H), 3.71 (s, 3H), 2.02 (tt, *J* = 9.3, 5.0 Hz, 1H), 1.69 – 1.53 (m, 2H), 1.52 – 1.20 (m, 4H), 0.85 (dt, *J* = 14.1, 7.3 Hz, 6H).

^{13}C NMR (101 MHz, $CDCl_3$) δ 174.3, 156.3, 131.1, 121.9, 114.0, 55.5, 50.3, 35.1, 26.2, 20.8, 14.1, 12.1.

HRMS (ESI) m/z $(M+H)^+$ calcd for $C_{14}H_{22}NO_2$: 236.1645, found: 236.1643.

Supplementary Table 12. Failed substrates

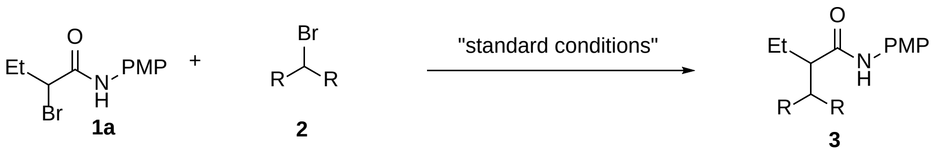

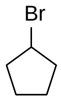	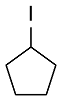	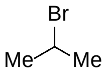	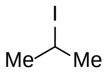
n.d.<br>conv. **1a**: 100%	n.d.<br>conv. **1a**: 100%	n.d.<br>conv. **1a**: 100%	n.d.<br>conv. **1a**: 100%

Reaction conditions: $NiCl_2$(dme) (0.016 mmol, 8 mol%), (*S*'*S*)-**L20** (0.016 mmol, 8 mol%) and diglyme (1.0 mL) was premixed for 1 h, then added CsI (3.0 equiv), 15C5 (10 mol%), $FeCl_2$ (25 mol%), Zn (2.0 equiv), **1a** (0.2 mmol, 1.0 equiv), **2** (3.0 equiv), diglyme (0.5 mL) and DMA (0.5 mL), the mixture was stirred at r.t. for 24 h. Yields were determined by GC.

2.5 Mechanistic Experiments

2.5.1 Quench of radical intermediates

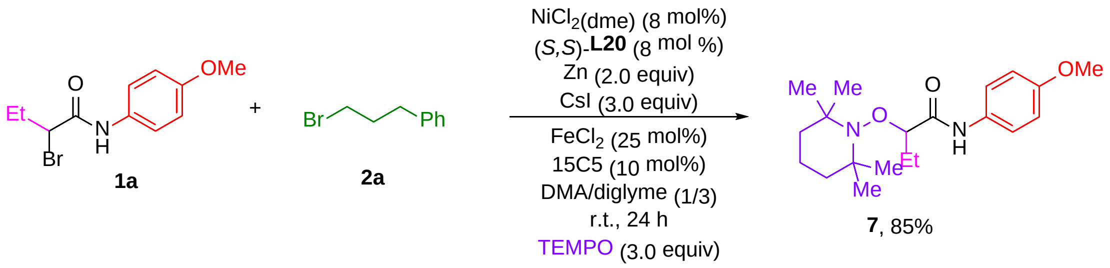

***N*-(4-Methoxyphenyl)-2-((2,2,6,6-tetramethylpiperidin-1-yl)oxy)butanamide (7).** In a nitrogen-filled glovebox, $NiCl_2$·glyme (4.4 mg, 0.02 mmol, 10 mol%), chiral ligand ***(S,S)*-L20** (6.6 mg, 0.016 mmol, 8 mol%) and diglyme (1.0 mL) were added to a 10-mL vial equipped with a stir bar. The mixture was allowed to stir for 1 h, after which it was an orange solution. Then, $FeCl_2$ (6.4 mg, 0.05 mmol, 25 mol%), 15C5 (4.4 mg, 0.02 mmol, 10 mol%), CsI (156.0 mg, 0.6 mmol, 300 mol%), Zn (26.0 mg, 0.4 mmol, 200 mol%), **1a** (54.2 mg, 0.2 mmol), **2a** (118.8 mg, 0.6 mmol, 300 mol%), TEMPO (3.0 equiv, 93.6 mg), DMA (0.5 mL) and diglyme (0.5 mL) were added. The reaction mixture was transferred out of the glovebox and stirred (~1400 rpm) at room temperature for 24 h. Next, ethyl acetate (20.0 mL) was added, and the mixture was washed with water (10.0 mL) and brine (10.0 mL), dried over Na_2SO_4, filtered, and concentrated under vacuum. The product was purified by flash chromatography (15% ethyl ether/hexanes). White solid. 58.8 mg (85% yield).

^{1}H NMR (400 MHz, Chloroform-*d*) δ 8.29 (s, 1H), 7.53 – 7.43 (m, 2H), 6.95 – 6.81 (m, 2H), 4.36 (dd, J = 6.0, 3.3 Hz, 1H), 3.79 (s, 3H), 2.12 – 1.95 (m, 2H), 1.49 (s, 6H), 1.23 (s, 6H), 1.16 (s, 6H), 0.93 (t, J = 7.5 Hz, 3H).

^{13}C NMR (101 MHz, $CDCl_3$) δ 170.8, 156.4, 130.5, 121.3, 114.2, 87.1, 55.5, 40.4, 24.6, 16.9, 7.8.

HRMS (ESI) m/z $(M+H)^+$ calcd for $C_{20}H_{33}N_2O_3$: 349.2486, found: 349.2485.

2.5.2 Secondary alkyl zinc reagent

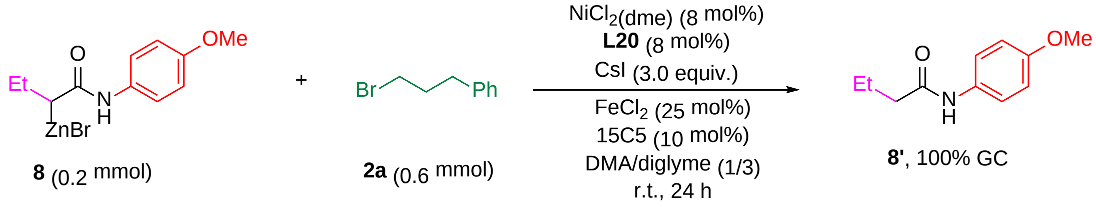

In a nitrogen-filled glovebox, **1a** (54.2 mg, 0.2 mmol), Zn (13.0 mg, 0.2 mmol, 100 mol%), CsI (5.2 mg, 0.02 mmol), DMA (0.5 mL) were added to a 10-mL vial equipped with a stir bar. The mixture was allowed to stir for 1 h, after which it was a white slurry. Then, $NiCl_2$•glyme (3.5 mg, 0.016 mmol, 8 mol%), chiral ligand **(*S,S)*-L20** (6.6 mg, 0.016 mmol, 8 mol%) (catalyst and ligand were premixed in another vial), $FeCl_2$ (6.4 mg, 0.05 mmol, 25 mol%), 15C5 (4.4 mg, 0.02 mmol, 10 mol%), CsI (156.0 mg, 0.6 mmol, 300 mol%), **2a** (118.8 mg, 0.6 mmol, 300 mol%) and diglyme (1.5 mL) were added. The reaction mixture was transferred out of the glovebox and stirred (~1400 rpm) at room temperature for 24 h. The reaction mixture was detected by GC using dodecane as the internal standard.

2.5.3 Fu's optimal catalytic condition

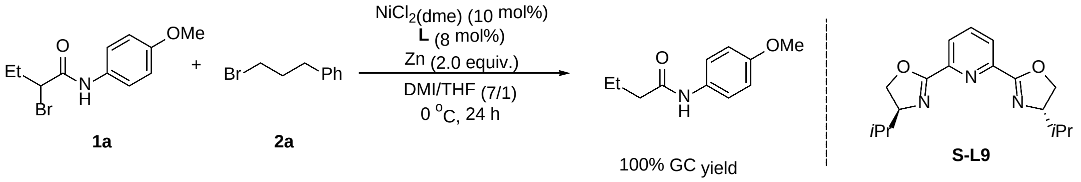

According to literature procedure,[8] in a nitrogen-filled glovebox, $NiCl_2$•dme (2.2 mg, 0.01 mmol, 1 mol%), chiral ligand ***S*-L9** (2.4 mg, 0.008 mmol, 8 mol%) and DMI/THF (7/1, 1.0 mL) were added to a 10-mL vial equipped with a stir bar. The mixture was allowed to stir for 0.5 h. Then, **1a** (27.1 mg, 0.1 mmol), **2a** (59.4 mg, 0.3 mmol), Zn (13.0 mg, 0.2 mmol) were added. The reaction mixture was transferred out of the glovebox and stirred (~1400 rpm) at room temperature for 24 h. The reaction mixture was detected by GC using dodecane as the internal standard. *N*-(4-methoxyphenyl)butyramide was formed in 100% yield and **3a** was not detected.

2.5.4 Primary alkyl zinc reagents

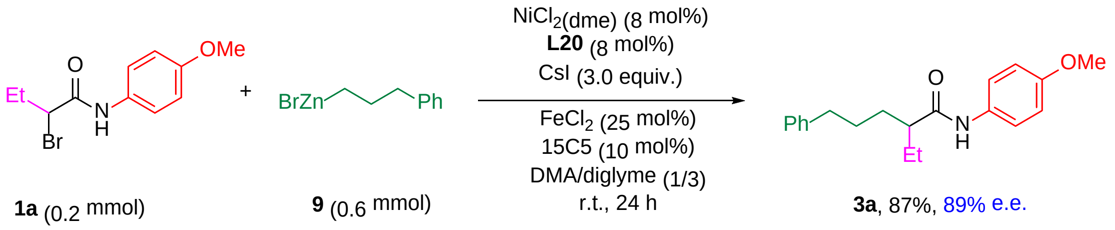

Preparation of the organozinc reagent. According to literature procedure,[9] a 20.0 mL vial charged with zinc dust (1.0 g, 15.0 mmol, 1.5 equiv) and a cross-shaped stir bar was capped with a PTFE-lined pierceable cap, and heated at 70 °C under high vacuum for 1 h. After the vial had been placed under nitrogen and allowed to cool to room temperature, iodine (127.0 mg, 0.5 mmol, 0.05 equiv) in DMA (3.0 mL) was added under N_2, and the suspension was stirred until the solution was colorless. In turn, DMA (6.0 mL) and the primary alkyl bromide (10.0 mmol, 1.0 equiv) were added under N_2 via syringe. The suspension was heated at 70 °C for 12 h (stir-rate: 1400 rpm). Next, the mixture was cooled to room temperature and filtered with a syringe filter (pore-size: 0.45 μM, PTFE).

In a nitrogen-filled glovebox, $NiCl_2$•glyme (3.5 mg, 0.016 mmol, 8 mol%), chiral ligand ***(S,S)*-L20** (6.6 mg, 0.016 mmol, 8 mol%) and diglyme (1.0 mL) were added to a 10-mL vial equipped with a stir bar. The mixture was allowed to stir for 1 h, after which it was an orange solution. Then, $FeCl_2$ (6.4 mg, 0.05 mmol, 25 mol%), 15C5 (4.4 mg, 0.02 mmol, 10 mol%), CsI (156.0 mg, 0.6 mmol, 300 mol%), **1a** (54.2 mg, 0.2 mmol), **9** (0.375 mL, 1.6M in DMA, 0.6 mmol, 300 mol%), DMA (0.125 mL) and diglyme (0.5 mL) were added. The reaction mixture was transferred out of the glovebox and stirred (~1400 rpm) at room temperature for 24 h. Next, ethyl acetate (20.0 mL) was added, and the mixture was washed with water (10.0 mL) and brine (10.0 mL), dried over Na_2SO_4, filtered, and concentrated under vacuum. The residue was purified by flash chromatography on silica gel (15% ethyl ether/hexanes). White solid. 54.1 mg (87% yield), 89% ee.

2.5.5 Slow addition of organozinc reagent

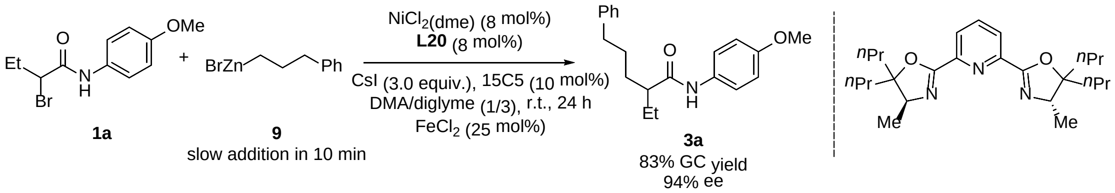

In a nitrogen-filled glovebox, $NiCl_2$·dme (3.5 mg, 0.016 mmol, 8 mol%), chiral ligand ***(S,S)*-L20** (6.6 mg, 0.016 mmol, 8 mol%) and diglyme (1.0 mL) were added to a 10-mL vial equipped with a stir bar. The mixture was allowed to stir for 1 h, after which it was an orange solution. Then, $FeCl_2$ (6.4 mg, 0.05 mmol, 25 mol%), 15C5 (4.4 mg, 0.02 mmol, 10 mol%), CsI (156.0 mg, 0.6 mmol, 300 mol%), **1a** (54.2 mg, 0.2 mmol), DMA (0.125 mL) and diglyme (0.5 mL) were added. The reaction mixture was stirred and **9** (0.375 mL, 1.6M in DMA, 0.6 mmol, 300 mol%) was added slowly in 10 minutes by syringe. The reaction mixture was transferred out of the glovebox and stirred (~1400 rpm) at room temperature for 24 h. The yield was detected by GC using dodecane as the internal standard and the ee was detected by HPLC.

2.5.6 Consumption of primary alkyl bromide with zinc and CsI

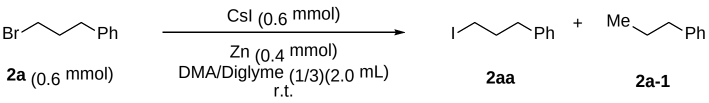

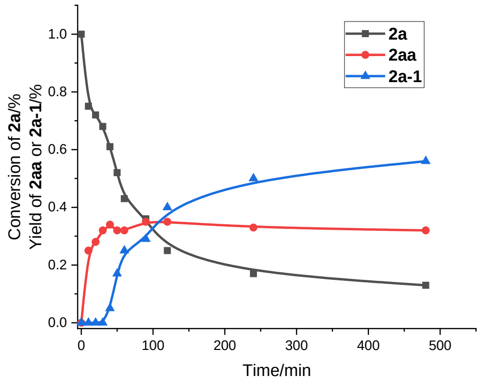

Supplementary Figure 1 Consumption of primary alkyl bromide with zinc and CsI.

In a nitrogen-filled glovebox, CsI (156.0 mg, 0.6 mmol, 300 mol%), Zn (26..0 mg, 0.4 mmol, 200 mol%), **2a** (118.8 mg, 0.6 mmol, 300 mol%), DMA (0.5 mL) and diglyme (1.5 mL) were added. The reaction mixture was transferred out of the glovebox

and stirred (~1400 rpm) at room temperature. The reaction mixture was detected by GC and NMR using dodecane and 1,3,5-trimethoxybenzene as the internal standard.

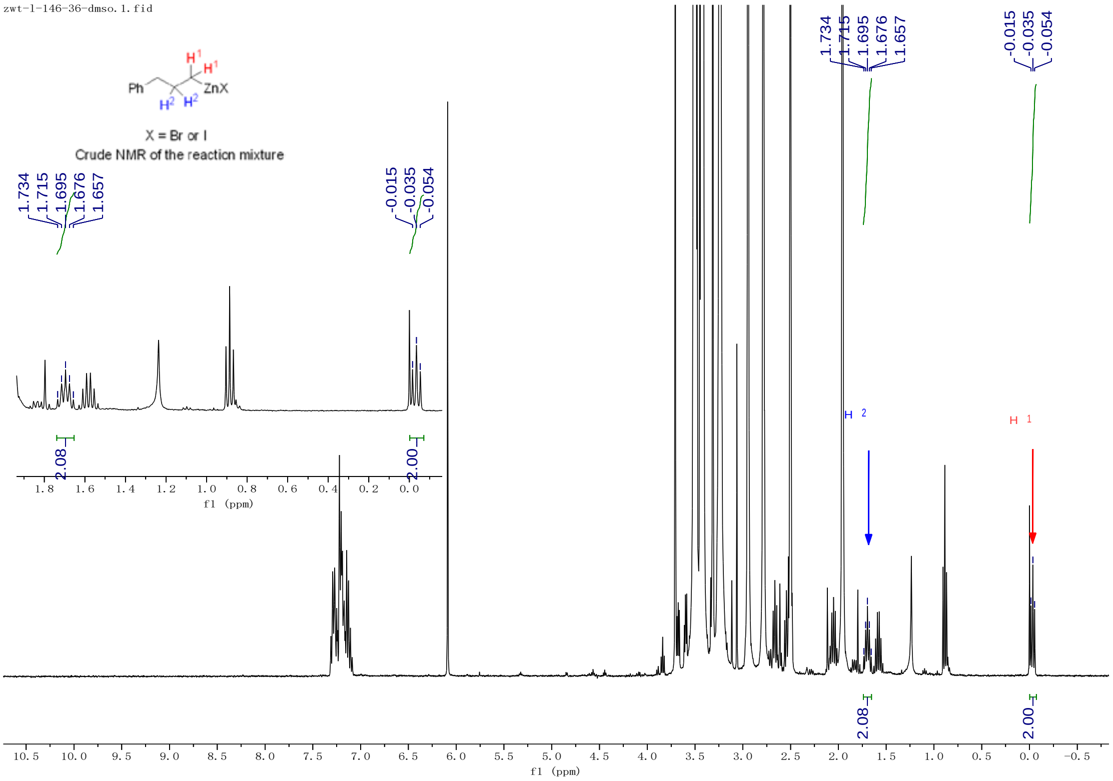

Supplementary Figure 2 ^{1}H NMR (400 MHz, DMSO-d_6, 25 °C) spectra of the reaction at 360 min

2.5.7 Control experiments

Control experiments without $NiCl_2$·dme

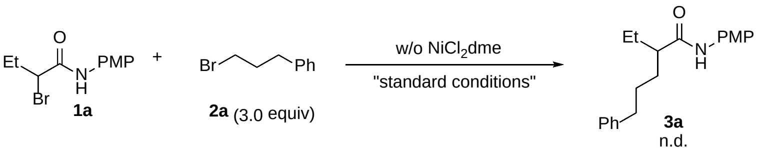

In a nitrogen-filled glovebox, chiral ligand **(*S,S*)-L20** (6.6 mg, 0.016 mmol, 8 mol%), $FeCl_2$ (6.4 mg, 0.05 mmol, 25 mol%), 15C5 (4.4 mg, 0.02 mmol, 10 mol%), CsI (156.0 mg, 0.6 mmol, 300 mol%), Zn (26.0 mg, 0.4 mmol, 200 mol%), **1a** (54.2 mg, 0.2 mmol), **2a** (118.8 mg, 0.6 mmol, 300 mol%), DMA (0.5 mL) and diglyme (1.5 mL) were added to a 10-mL vial equipped with a stir bar. The reaction mixture was transferred out of the glovebox and stirred (~1400 rpm) at room temperature for 24 h. Next, ethyl acetate (20.0 mL) was added, and the mixture was washed with water (10.0 mL) and brine

(10.0 mL), dried over Na_2SO_4, filtered, and concentrated under vacuum. The mixture was detected by GC using dodecane as the internal standard.

Control experiments without $FeCl_2$

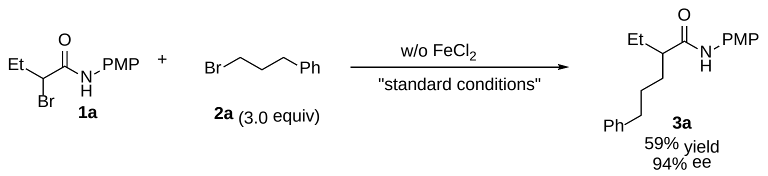

In a nitrogen-filled glovebox, $NiCl_2$•glyme (4.4 mg, 0.02 mmol, 10 mol%), chiral ligand **(*S,S*)-L20** (6.6 mg, 0.016 mmol, 8 mol%) and diglyme (1.0 mL) were added to a 10-mL vial equipped with a stir bar. The mixture was allowed to stir for 1 h, after which it was an orange solution. Then, 15C5 (4.4 mg, 0.02 mmol, 10 mol%), CsI (156.0 mg, 0.6 mmol, 300 mol%), Zn (26.0 mg, 0.4 mmol, 200 mol%), **1a** (54.2 mg, 0.2 mmol), **2a** (118.8 mg, 0.6 mmol, 300 mol%), DMA (0.5 mL) and diglyme (0.5 mL) were added. The reaction mixture was transferred out of the glovebox and stirred (~1400 rpm) at room temperature for 24 h. Next, ethyl acetate (20.0 mL) was added, and the mixture was washed with water (10.0 mL) and brine (10 mL), dried over Na_2SO_4, filtered, and concentrated under vacuum. **3a** was afforded in 59% yield and 94% ee. The yield was detected by GC using dodecane as the internal standard and the ee was detected by HPLC.

Control experiments using primary alkyl iodide without CsI and 15C5.

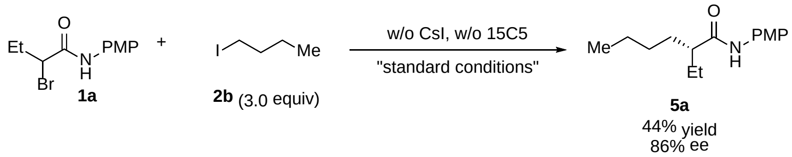

In a nitrogen-filled glovebox, $NiCl_2$•glyme (4.4 mg, 0.02 mmol, 10 mol%), chiral ligand **(*S,S*)-L20** (6.6 mg, 0.016 mmol, 8 mol%) and diglyme (1.0 mL) were added to a 10-mL vial equipped with a stir bar. The mixture was allowed to stir for 1 h, after which it was an orange solution. Then, $FeCl_2$ (6.4 mg, 0.05 mmol, 25 mol%), Zn (26.0 mg, 0.4 mmol, 200 mol%), **1a** (54.2 mg, 0.2 mmol), 1-iodobutane (**2b**) (110.4 mg, 0.6 mmol), DMA (0.5 mL) and diglyme (0.5 mL) were added. The reaction mixture was transferred out of the glovebox and stirred (~1400 rpm) at room temperature for 24 h.

Next, ethyl acetate (20.0 mL) was added, and the mixture was washed with water (10.0 mL) and brine (10.0 mL), dried over Na_2SO_4, filtered, and concentrated under vacuum. **5a** was afforded in 44% yield and 86% ee. The yield was detected by GC using dodecane as the internal standard and the ee was determined by chiral HPLC. The ee was determined by HPLC on a Daicel CHIRALPAK® AS-H column (5% *i*-PrOH/hexanes, 1.0 mL/min); retention times for compound obtained using **(*S,S*)-L20**: 7.8 min (minor), 9.4 min (major).

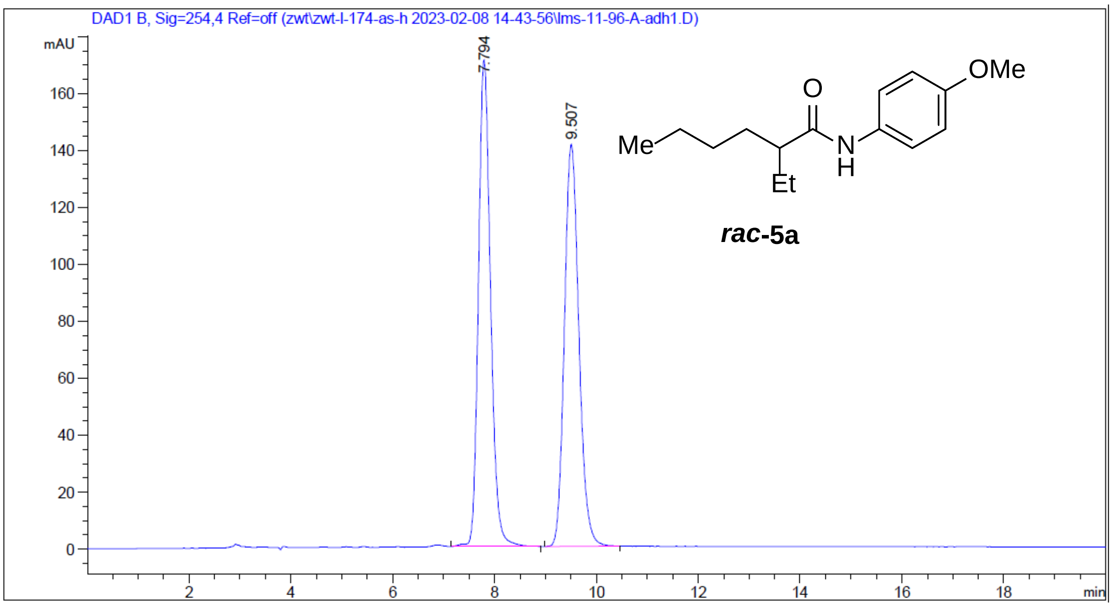

Peak #	RetTime [min]	Type	Width [min]	Area [mAU*s]	Height [mAU]	Area %
1	7.794	BB	0.2543	2815.49902	170.81931	50.2906
2	9.507	BB	0.3060	2782.96118	141.15082	49.7094

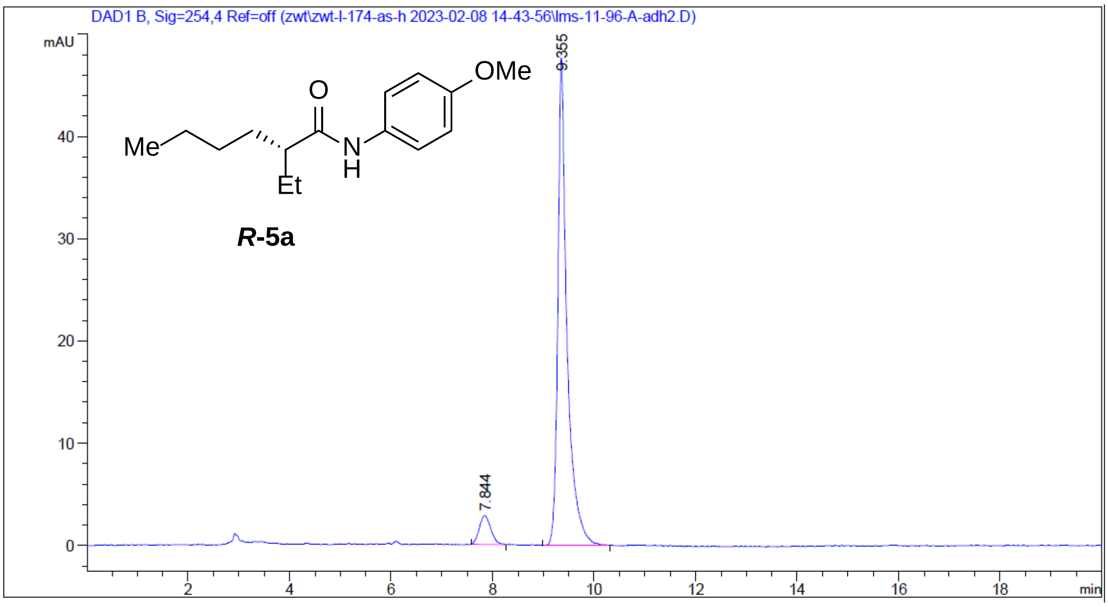

Peak #	RetTime [min]	Type	Width [min]	Area [mAU*s]	Height [mAU]	Area %
1	7.844	BB	0.2177	44.19193	2.80642	6.8513
2	9.355	BB	0.1749	600.82416	47.70834	93.1487

Supplementary Figure 3 HPLC spectra of **5a**

Control experiments using alkyl zinc reagent without CsI, Zn and 15C5.

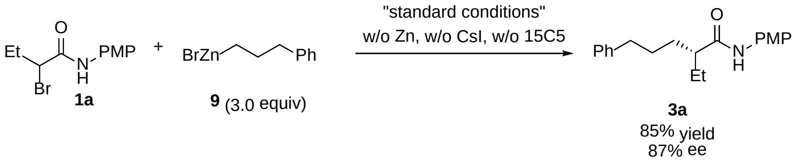

In a nitrogen-filled glovebox, $NiCl_2$•dme (3.5 mg, 0.016 mmol, 8 mol%), chiral ligand ***(S,S)*-L20** (6.6 mg, 0.016 mmol, 8 mol%) and diglyme (1.0 mL) were added to a 10-mL vial equipped with a stir bar. The mixture was allowed to stir for 1 h, after which it was an orange solution. Then, $FeCl_2$ (6.4 mg, 0.05 mmol, 25 mol%), **1a** (54.2 mg, 0.2 mmol), DMA (0.125 mL) and diglyme (0.5 mL) were added. The reaction mixture was stirred and **9** (0.375 mL, 1.6M in DMA, 0.6 mmol, 300 mol%) was added. The reaction mixture was transferred out of the glovebox and stirred (~1400 rpm) at room temperature for 24 h to afford **3a** in 85% yield and 87% ee. The yield was detected by GC using dodecane as the internal standard and the ee was determined by chiral HPLC. The ee was determined by HPLC on a Daicel CHIRALPAK® OD-H column (5% *i*-PrOH/hexanes, 1.0 mL/min); retention times for compound obtained using ***(S,S)*-L20**: 19.3 min (major), 22.0 min (minor).

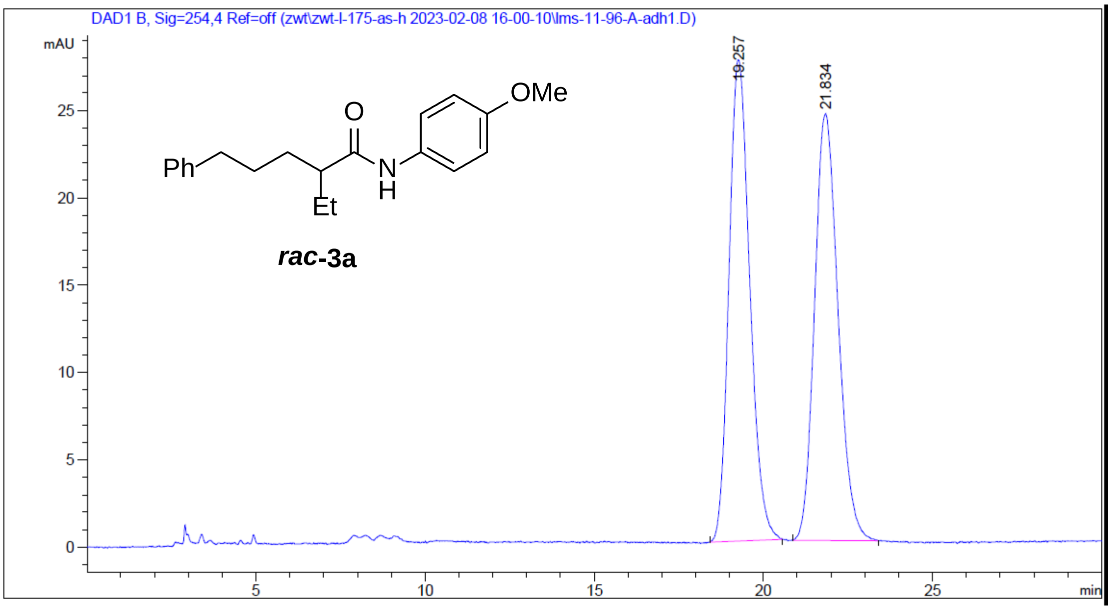

Peak #	RetTime [min]	Type	Width [min]	Area [mAU*s]	Height [mAU]	Area %
1	19.257	BB	0.6325	1177.20752	27.54948	49.9678
2	21.834	BB	0.6652	1178.72583	24.41816	50.0322

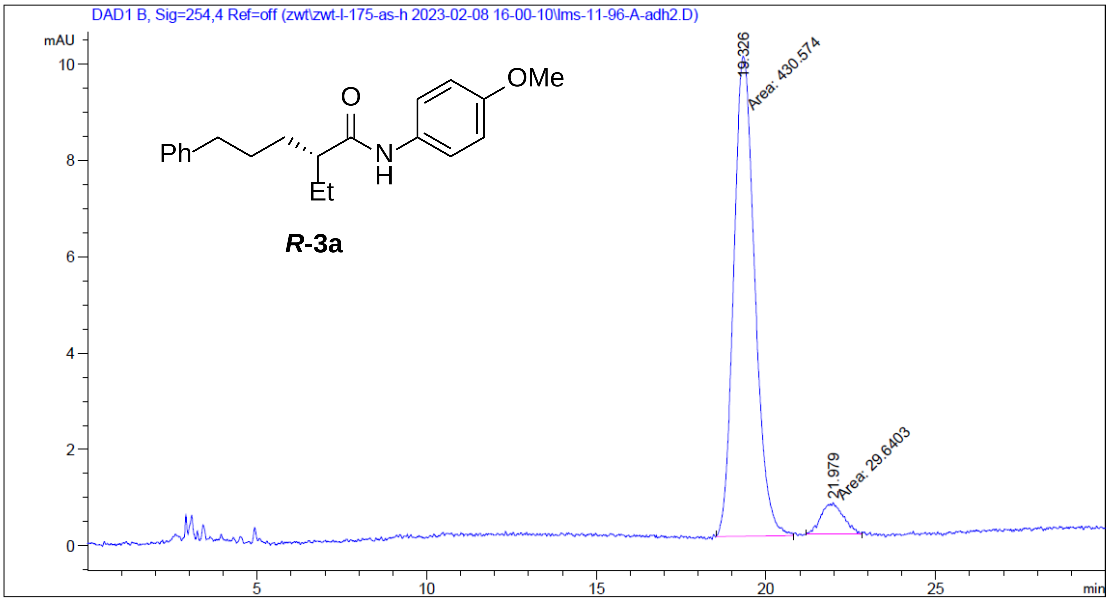

Peak #	RetTime [min]	Type	Width [min]	Area [mAU*s]	Height [mAU]	Area %
1	19.326	MM	0.7199	430.57449	9.96821	93.5595
2	21.979	MM	0.7574	29.64028	6.52273e-1	6.4405

Supplementary Figure 4 HPLC spectra of **3a**

2.5.8 Selectivity between primary alkyl bromide and primary alkyl iodide

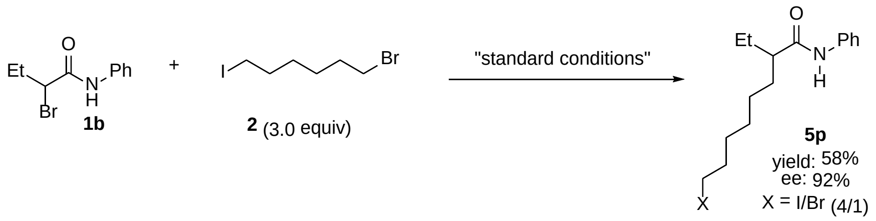

In a nitrogen-filled glovebox, $NiCl_2$·glyme (4.4 mg, 0.02 mmol, 10 mol%), chiral ligand ***(S,S)*-L20** (6.6 mg, 0.016 mmol, 8 mol%) and diglyme (1.0 mL) were added to a 10-mL vial equipped with a stir bar. The mixture was allowed to stir for 1 h, after which it was an orange solution. Then, 15C5 (4.4 mg, 0.02 mmol, 10 mol%), CsI (156.0 mg, 0.6 mmol, 300 mol%), $FeCl_2$ (6.3 mg, 0.05 mmol, 25 mol%), Zn (26.0 mg, 0.4 mmol, 200 mol%), **1b** (48.2 mg, 0.2 mmol), **2** (174.0 mg, 0.6 mmol, 300 mol%), DMA (0.5 mL) and diglyme (0.5 mL) were added. The reaction mixture was transferred out of the glovebox and stirred (~1400 rpm) at room temperature for 24 h. Next, ethyl acetate (20.0 mL) was added, and the mixture was washed with water (10.0 mL) and brine (10.0 mL), dried over Na_2SO_4, filtered, and concentrated under vacuum. Flash chromatography on silica gel afforded **5p** as white solid (43.2 mg, 58% yield, 92% ee).

The ratio between iodide and bromide was determined by the ^{1}H NMR of crude mixture of the reaction. The ee was determined by HPLC on a Daicel CHIRALPAK® OD-H column (5% *i*-PrOH/hexanes, 1.0 mL/min); retention times for compound obtained using ***(S,S)*-L20**: 9.5 min (major), 15.7 min (minor).

^{1}H NMR (400 MHz, Chloroform-*d*) δ 7.55 (d, *J* = 7.8 Hz, 2H), 7.32 (t, *J* = 7.9 Hz, 2H), 7.23 (s, 1H), 7.10 (t, *J* = 7.4 Hz, 1H), 3.38 (t, J = 7.0 Hz, 0.45H), 3.16 (t, *J* = 7.0 Hz, 1.59H), 2.13 – 2.06 (m, 1H), 1.85 – 1.66 (m, 4H), 1.60 – 1.45 (m, 2H), 1.41 – 1.28 (m, 6H), 0.96 (t, *J* = 7.4 Hz, 3H).

^{13}C NMR (101 MHz, $CDCl_3$) δ 174.2, 137.8, 129.07, 124.2, 119.8, 50.8, 33.4, 32.7, 30.3, 28.6, 27.5, 26.2, 12.1, 7.2.

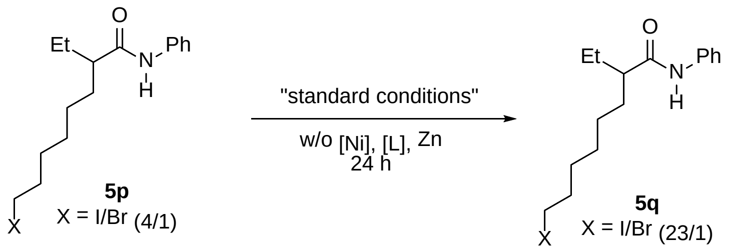

In a nitrogen-filled glovebox, **5p** (7.5 mg, 0.02 mmol), 15C5 (0.4 mg, 0.002 mmol, 10 mol%), CsI (15.6 mg, 0.06 mmol, 300 mol%), $FeCl_2$ (0.6 mg, 0.005 mmol, 25 mol%), DMA (0.05 mL) and diglyme (0.15 mL) were added to a 10-mL vial equipped with a stir bar. The reaction mixture was transferred out of the glovebox and stirred (~1400 rpm) at room temperature for 24 h. Next, ethyl acetate (5.0 mL) was added, and the mixture was washed with water (3.0 mL) and brine (2.0 mL), dried over Na_2SO_4, filtered, and concentrated under vacuum. Flash chromatography on silica gel afforded **5q** as white solid (7.2 mg, 96% yield).

^{1}H NMR (400 MHz, Chloroform-*d*) δ 7.63 – 7.49 (m, 2H), 7.39 – 7.28 (m, 2H), 7.22 – 7.07 (m, 2H), 3.39 (t, *J* = 7.0 Hz, 0.08H), 3.17 (t, *J* = 7.0 Hz, 1.89H), 2.12 – 2.05 (m, 1H), 1.83 – 1.66 (m, 4H), 1.59 – 1.46 (m, 2H), 1.42 – 1.28 (m, 6H), 0.96 (t, *J* = 7.4 Hz, 3H).

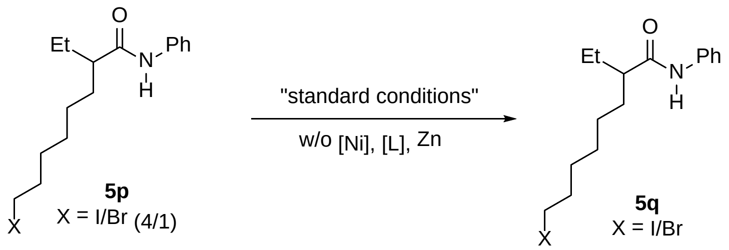

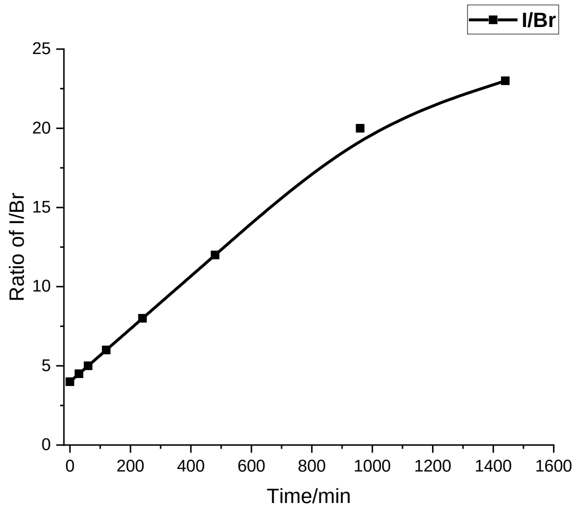

Supplementary Figure 5 Time course for halogen exchange of **5p**

In a nitrogen-filled glovebox, **5p** (7.5 mg, 0.02 mmol), 15C5 (0.4 mg, 0.002 mmol, 10 mol%), CsI (15.6 mg, 0.06 mmol, 300 mol%), $FeCl_2$ (0.6 mg, 0.005 mmol, 25 mol%), DMA (0.05 mL) and diglyme (0.15 mL) were added to a 10-mL vial equipped with a stir bar. The reaction mixture was transferred out of the glovebox and stirred (~1400 rpm) at room temperature. The reaction mixture was detected by NMR using 1,3,5-trimethoxybenzene as the internal standard.

2.5.9 Radical cyclization experiment

Supplementary Table 13. Radical cyclization experiment

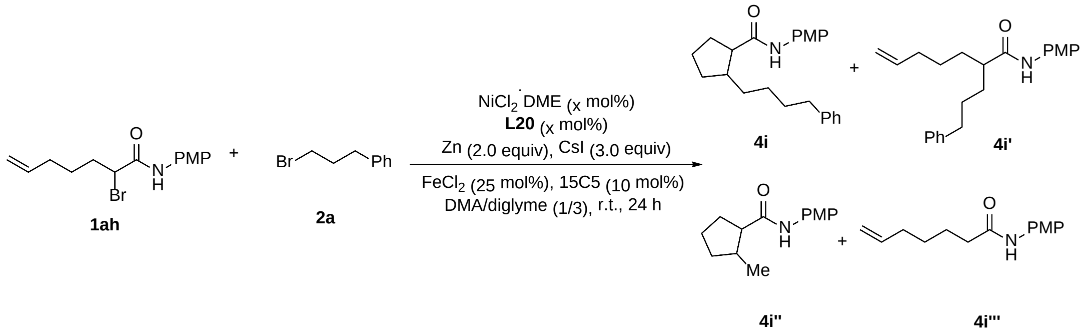

entry	[Ni]/L	conv.(1ah)/%	yield (4i)/%	d.r. (4i)	yield (4i')/%	yield (4i'')/%	yield (4i''')/%
1	2 mol%	>99%	<5%	--	n.d.	33%	58%
2	4 mol%	>99%	13%	1:1	n.d.	25%	55%
3	8 mol%	>99%	25%	1:1	n.d.	26%	42%
4	12 mol%	>99%	31%	1:1	n.d.	19%	44%

a Reaction conditions: $NiCl_2$(dme) (x mol%), **L20** (x mol%) and diglyme (1.0 mL) was premixed for 1 h, then added CsI (3.0 equiv), $FeCl_2$ (25 mol%), Zn (2.0 equiv), **1ah** (0.2 mmol, 1.0 equiv), **2** (3.0 equiv), diglyme (0.5 mL) and DMA (0.5 mL), the mixture was stirred at r.t. for 24 h. b yieldds was determined by NMR using dibromomethane as internal standard.

Procedure for entry 3:

In a nitrogen-filled glovebox, $NiCl_2$·glyme (4.4 mg, 0.02 mmol, 10 mol%), chiral ligand ***(S,S)*-L20** (6.6 mg, 0.016 mmol, 8 mol%) and diglyme (1.0 mL) were added to a 10-mL vial equipped with a stir bar. The mixture was allowed to stir for 1 h, after which it was an orange solution. Then, 15C5 (4.4 mg, 0.02 mmol, 10 mol%), CsI (156.0 mg, 0.6 mmol, 300 mol%), $FeCl_2$ (6.3 mg, 0.05 mmol, 25 mol%), Zn (26.0 mg, 0.4 mmol, 200 mol%), **1ah** (62.2 mg, 0.2 mmol), **2a** (118.8 mg, 0.6 mmol, 300 mol%), DMA (0.5 mL) and diglyme (0.5 mL) were added. The reaction mixture was transferred out of the glovebox and stirred (~1400 rpm) at room temperature for 24 h. Next, ethyl acetate (20 mL) was added, and the mixture was washed with water (10.0 mL) and brine (10.0 mL), dried over Na_2SO_4, filtered, and concentrated under vacuum. Flash chromatography on silica gel afforded **4i"** as white solid (17.7 mg, 25% yield, d.r. 1:1).

The d.r. ratio was determined by the ^{1}H NMR of crude mixture of the reaction.

^{1}H NMR (400 MHz, Chloroform-*d*) δ 7.42 (d, *J* = 9.0 Hz, 2H), 7.26 – 7.21 (m, 2H), 7.15 (t, *J* = 7.9 Hz, 3H), 7.00 (s, 1H), 6.86 (d, *J* = 9.0 Hz, 2H), 3.79 (s, 3H), 2.61 – 2.56

(m, 2H), 2.24 – 2.10 (m, 2H), 2.02 – 1.86 (m, 3H), 1.79 – 1.67 (m, 1H), 1.68 – 1.59 (m, 3H), 1.44 – 1.15 (m, 5H).

^{13}C NMR (101 MHz, $CDCl_3$) δ 174.1, 156.3, 142.8, 131.2, 128.4, 128.2, 125.6, 121.5, 114.1, 55.5, 54.1, 44.6, 35.8, 35.2, 32.6, 31.6, 31.1, 28.0, 24.8.

HRMS (ESI) m/z $(M+H)^+$ calcd for $C_{23}H_{30}NO_2$: 352.2271, found: 352.2267.

2.6 HPLC of Racemic and Enantioenriched Products

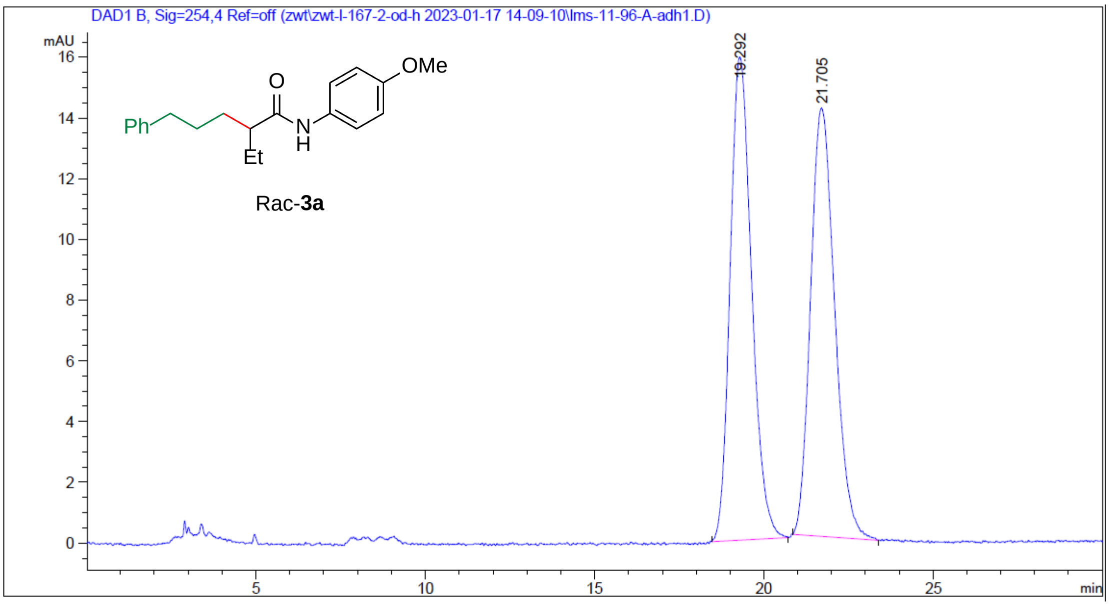

Peak #	RetTime [min]	Type	Width [min]	Area [mAU*s]	Height [mAU]	Area %
1	19.292	BB	0.6497	703.18231	15.90829	50.1681
2	21.705	BB	0.6235	698.47076	14.10936	49.8319

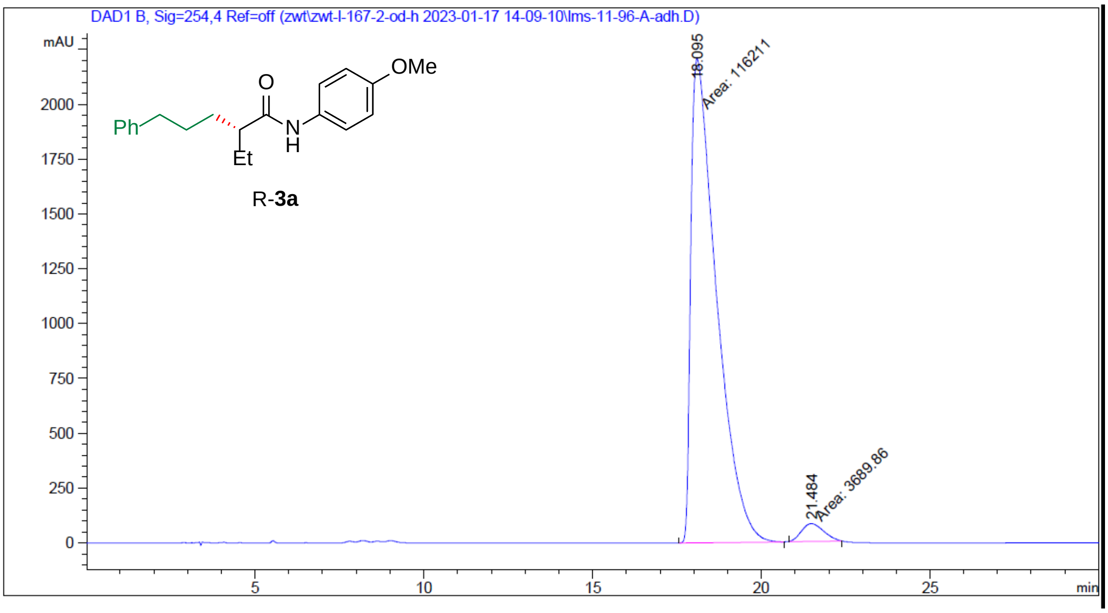

Peak #	RetTime [min]	Type	Width [min]	Area [mAU*s]	Height [mAU]	Area %
1	18.095	MM	0.8760	1.16211e5	2210.90771	96.9226
2	21.484	MM	0.7606	3689.85620	80.85405	3.0774

Supplementary Figure 6 HPLC spectra of **3a**

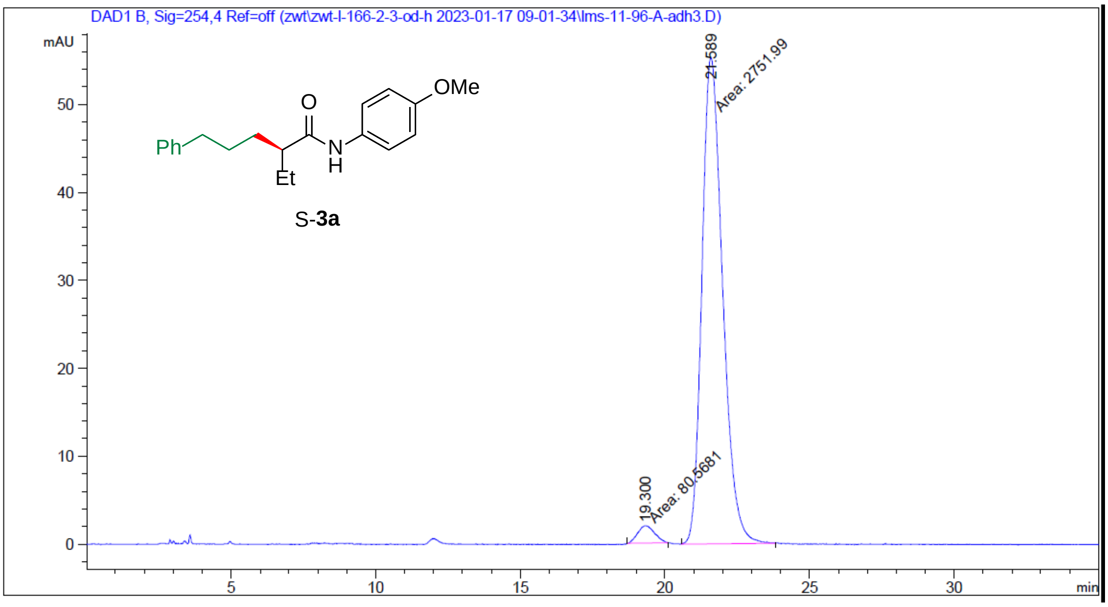

Peak #	RetTime [min]	Type	Width [min]	Area [mAU*s]	Height [mAU]	Area %
1	19.300	MM	0.6798	80.56806	1.97517	2.8444
2	21.589	MM	0.8299	2751.99268	55.26721	97.1556

Supplementary Figure 7 HPLC spectra of ***S*-3a**

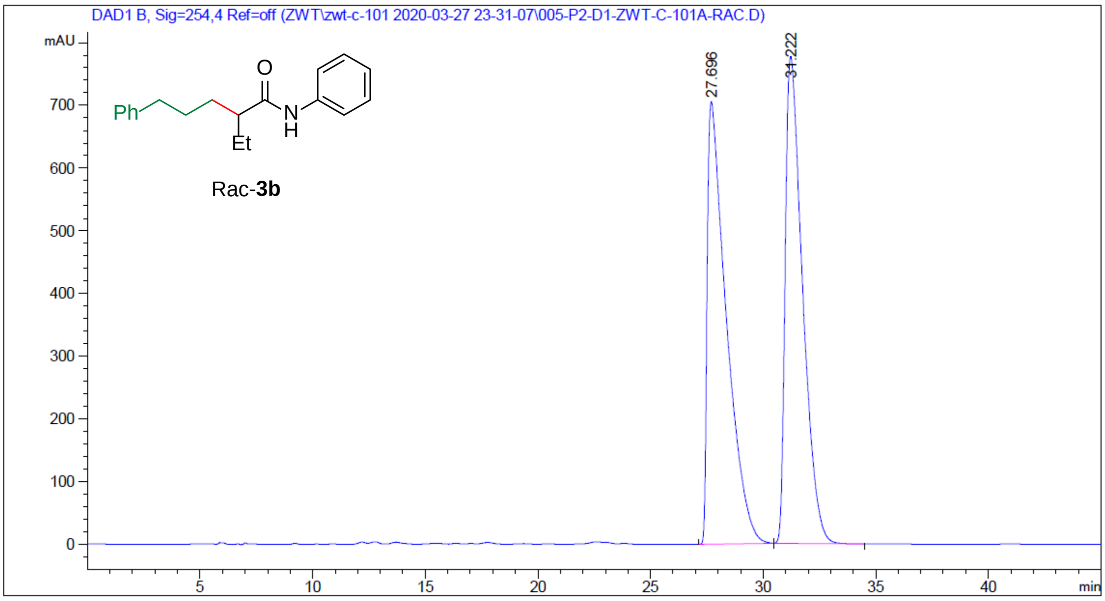

Peak #	RetTime [min]	Type	Width [min]	Area [mAU*s]	Height [mAU]	Area %
1	27.696	BB	0.8383	4.15317e4	706.05322	50.0546
2	31.222	BB	0.8064	4.14411e4	776.80756	49.9454

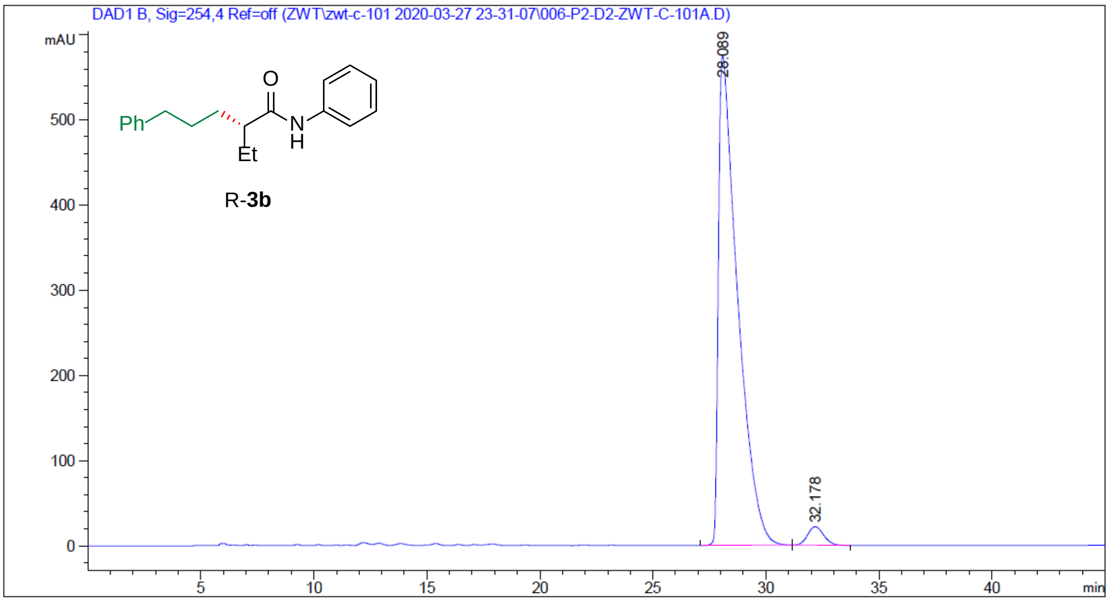

Peak #	RetTime [min]	Type	Width [min]	Area [mAU*s]	Height [mAU]	Area %
1	28.089	BB	0.8380	3.38157e4	575.05920	96.9286
2	32.178	BB	0.7581	1071.53552	21.70034	3.0714

Supplementary Figure 8 HPLC spectra of **3b**

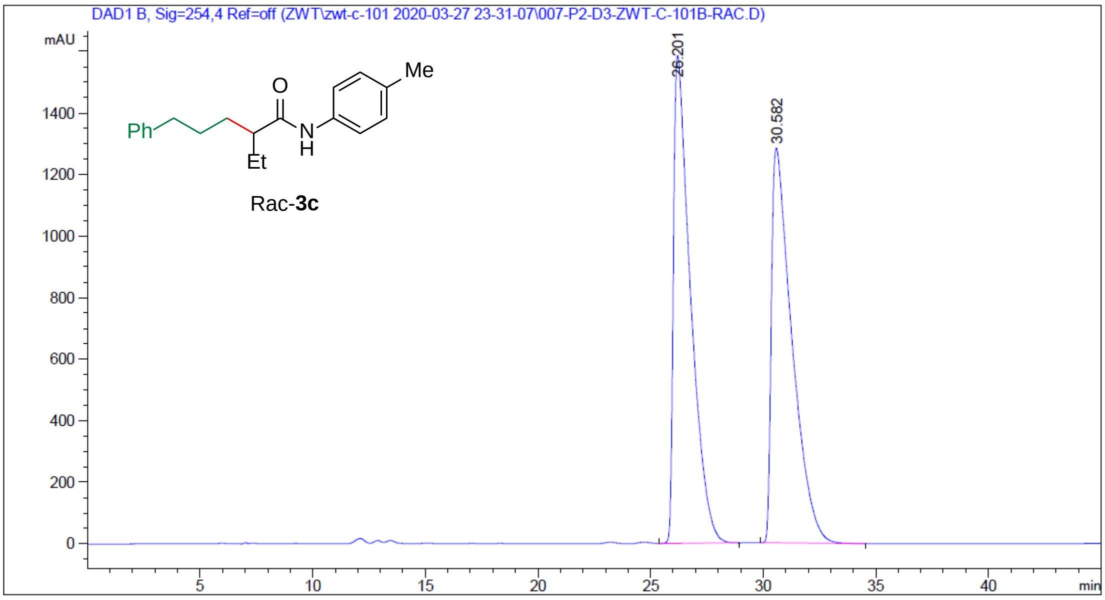

Peak #	RetTime [min]	Type	Width [min]	Area [mAU*s]	Height [mAU]	Area %
1	26.201	BB	0.7591	8.32838e4	1584.86426	50.5832
2	30.582	BB	0.9197	8.13634e4	1283.20239	49.4168

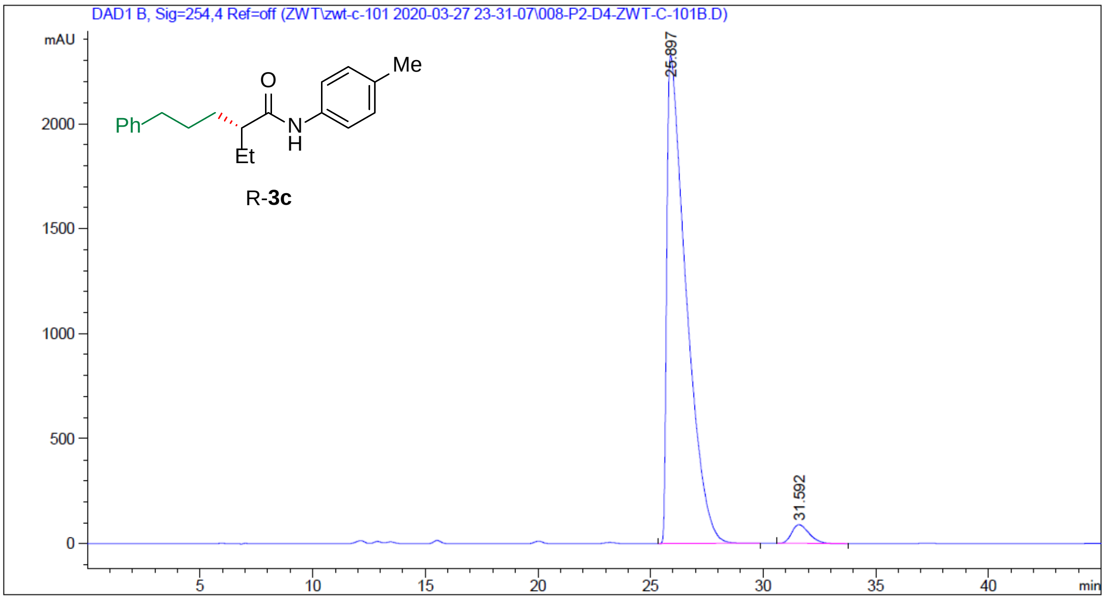

Peak #	RetTime [min]	Type	Width [min]	Area [mAU*s]	Height [mAU]	Area %
1	25.897	BB	0.8485	1.38345e5	2324.02002	96.6341
2	31.592	BB	0.8337	4818.71533	89.27510	3.3659

Supplementary Figure 9 HPLC spectra of **3c**

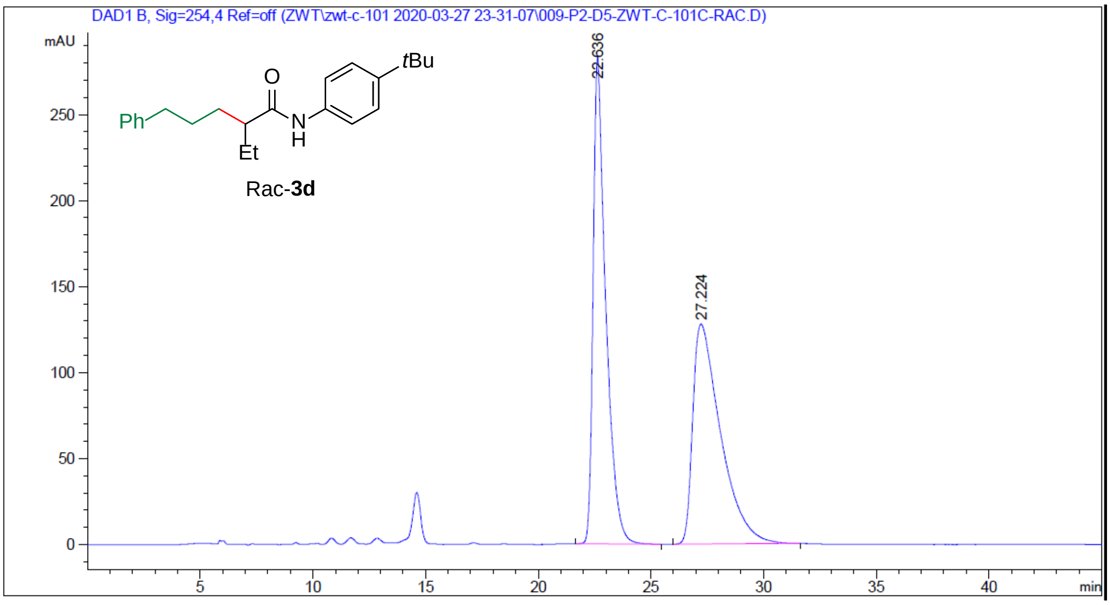

Peak #	RetTime [min]	Type	Width [min]	Area [mAU*s]	Height [mAU]	Area %
1	22.636	BB	0.5488	1.10319e4	282.98584	50.1545
2	27.224	BB	1.2421	1.09639e4	127.89331	49.8455

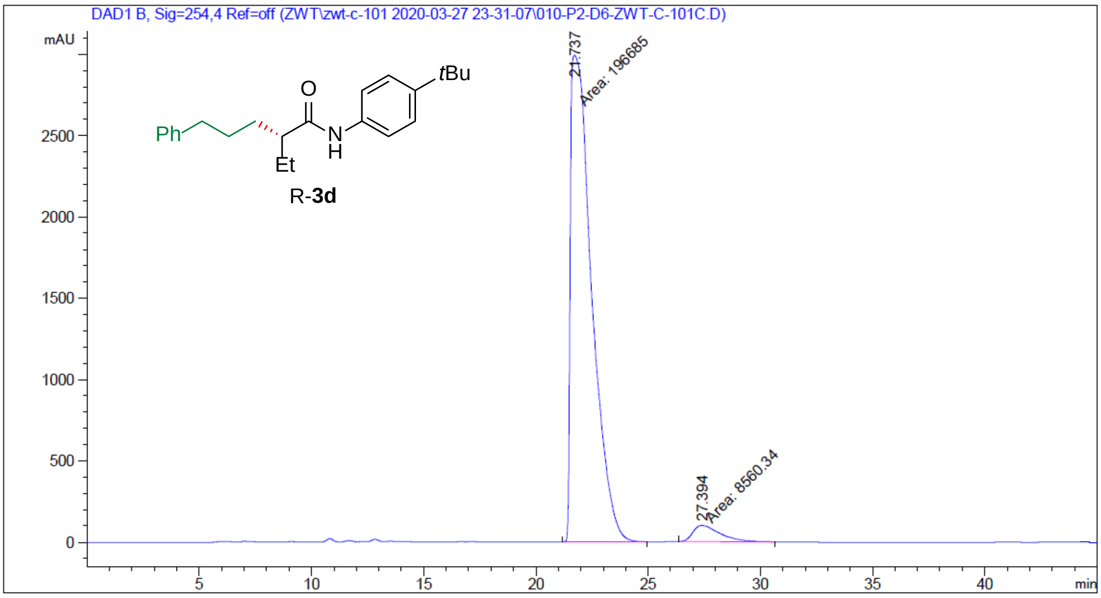

Peak #	RetTime [min]	Type	Width [min]	Area [mAU*s]	Height [mAU]	Area %
1	21.737	MM	1.0952	1.96685e5	2993.18799	95.8292
2	27.394	MM	1.4216	8560.33691	100.36264	4.1708

Supplementary Figure 10 HPLC spectra of **3d**

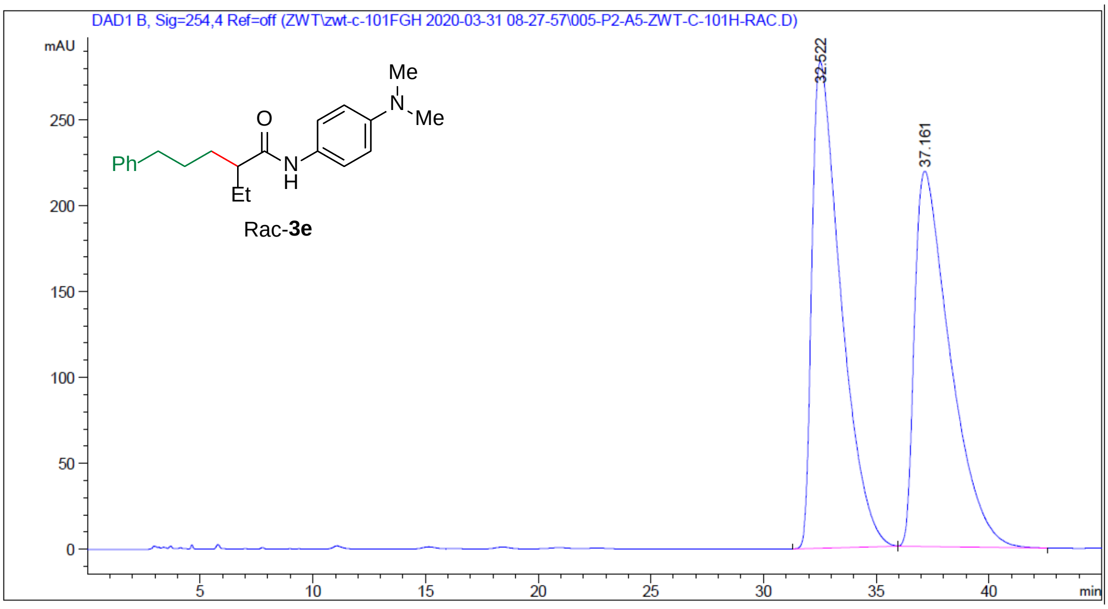

Peak #	RetTime [min]	Type	Width [min]	Area [mAU*s]	Height [mAU]	Area %
1	32.522	BB	1.2922	2.52565e4	283.28439	51.3855
2	37.161	BB	1.5858	2.38946e4	218.50560	48.6145

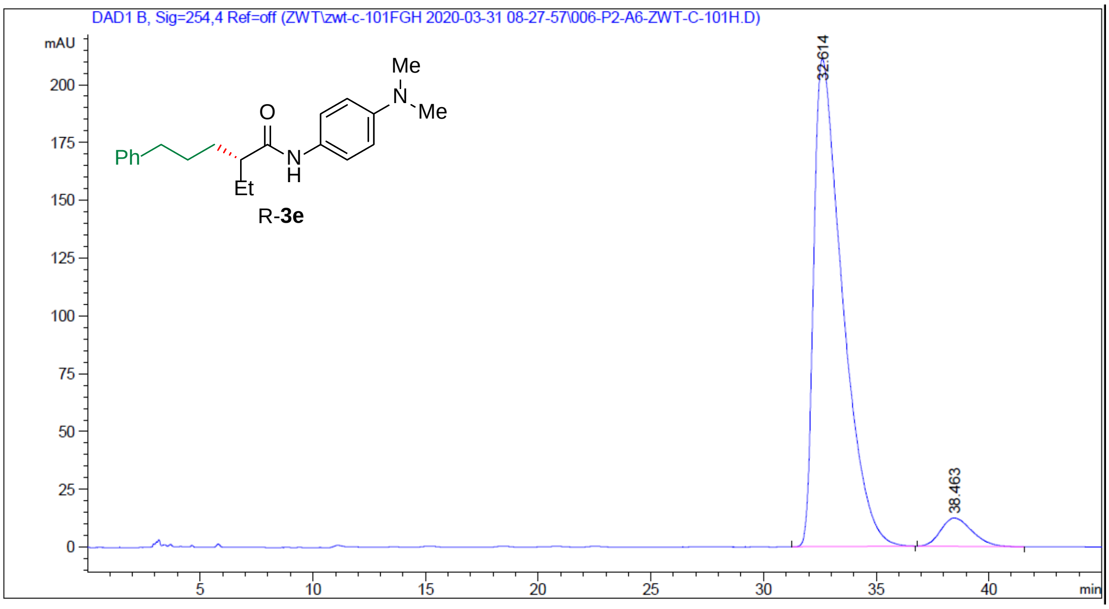

Peak #	RetTime [min]	Type	Width [min]	Area [mAU*s]	Height [mAU]	Area %
1	32.614	BB	1.2639	1.86175e4	210.90541	94.0231
2	38.463	BB	1.1858	1183.47754	12.28588	5.9769

Supplementary Figure 11 HPLC spectra of **3e**

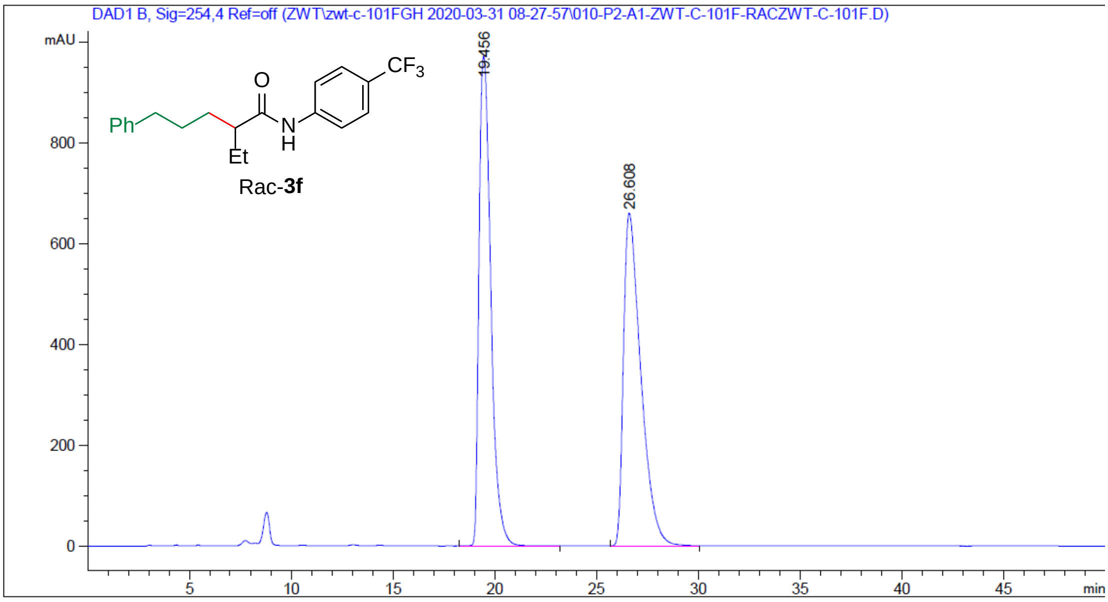

Peak #	RetTime [min]	Type	Width [min]	Area [mAU*s]	Height [mAU]	Area %
1	19.456	BB	0.6513	3.91129e4	972.32709	49.9295
2	26.608	BB	0.8888	3.92233e4	660.52441	50.0705

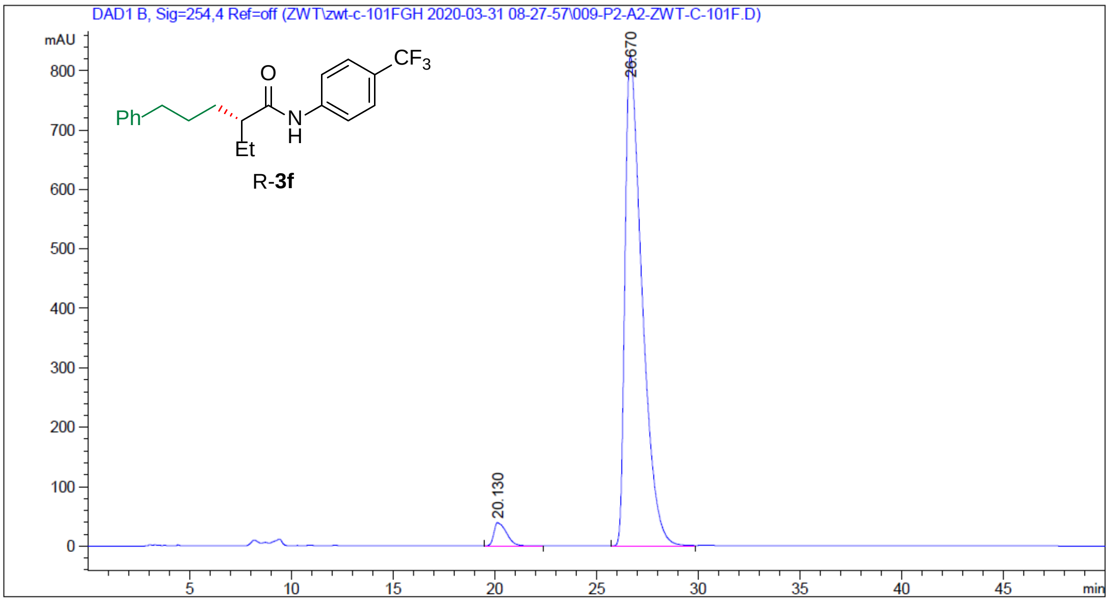

Peak #	RetTime [min]	Type	Width [min]	Area [mAU*s]	Height [mAU]	Area %
1	20.130	BB	0.5615	1694.45972	39.26527	3.4076
2	26.670	BB	0.8678	4.80319e4	824.26123	96.5924

Supplementary Figure 12 HPLC spectra of **3f**

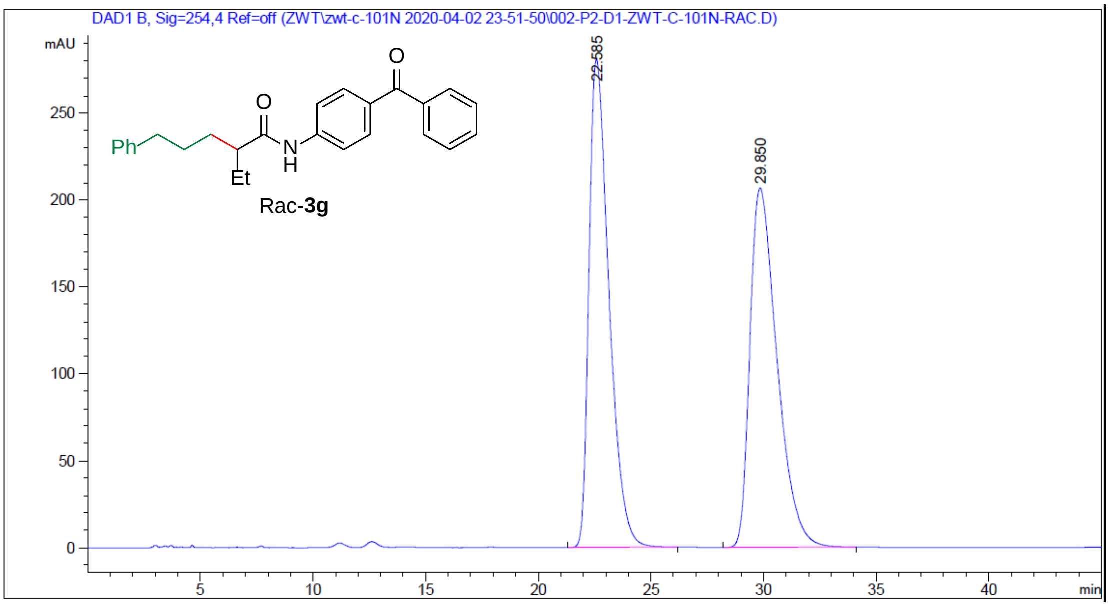

Peak #	RetTime [min]	Type	Width [min]	Area [mAU*s]	Height [mAU]	Area %
1	22.585	BB	0.9452	1.72990e4	280.60977	50.0487
2	29.850	BB	1.2479	1.72653e4	206.83388	49.9513

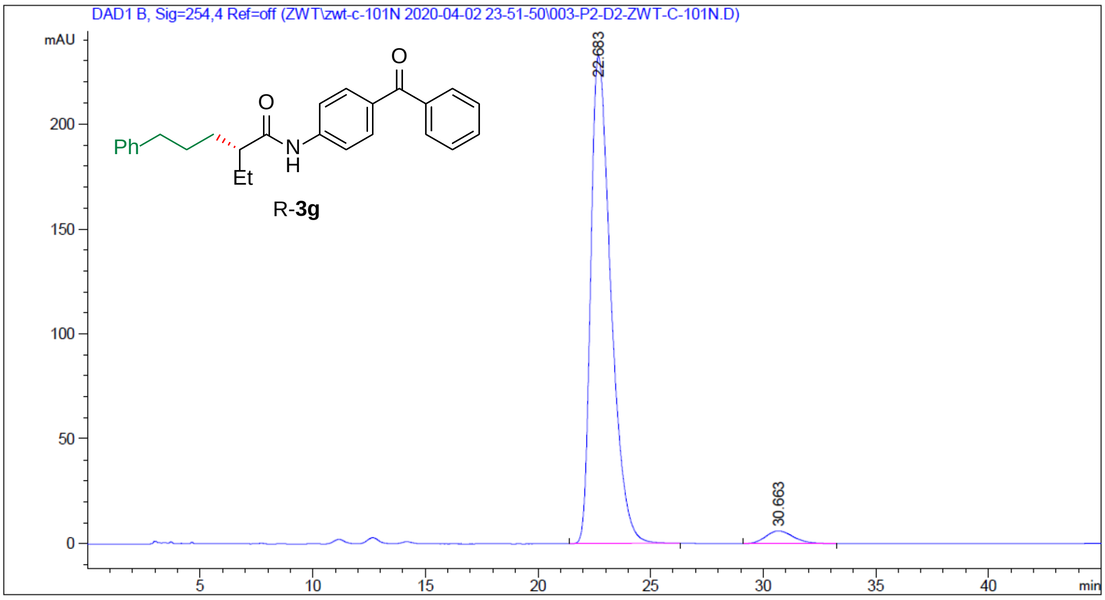

Peak #	RetTime [min]	Type	Width [min]	Area [mAU*s]	Height [mAU]	Area %
1	22.683	BB	0.9545	1.44013e4	232.55113	96.5135
2	30.663	BB	1.0169	520.24103	6.06412	3.4865

Supplementary Figure 13 HPLC spectra of **3g**

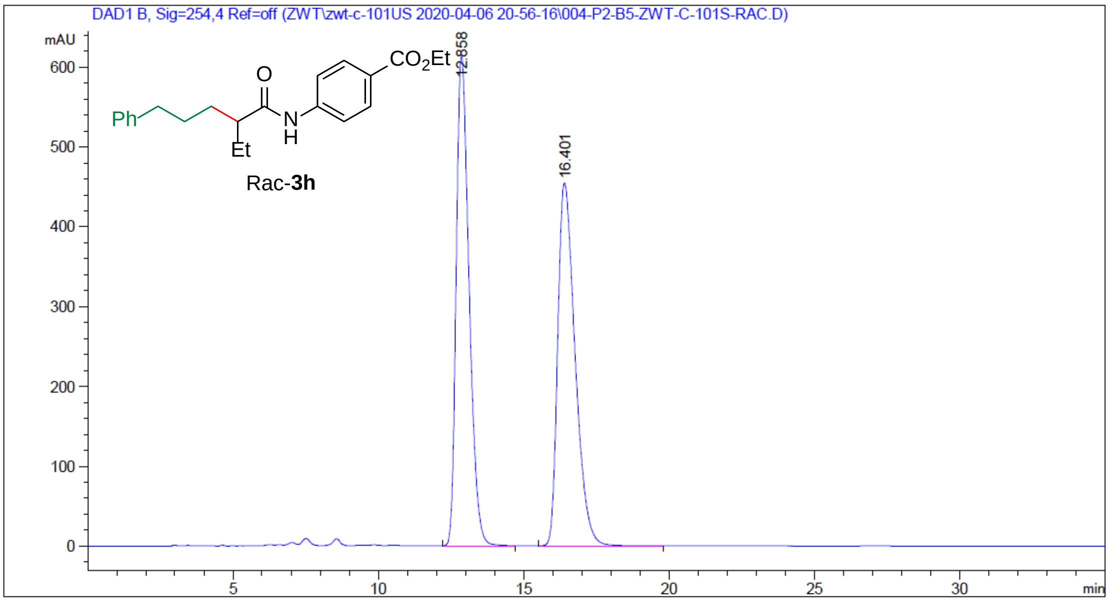

Peak #	RetTime [min]	Type	Width [min]	Area [mAU*s]	Height [mAU]	Area %
1	12.858	BB	0.4741	1.88331e4	613.52039	49.9627
2	16.401	BB	0.6341	1.88612e4	454.56516	50.0373

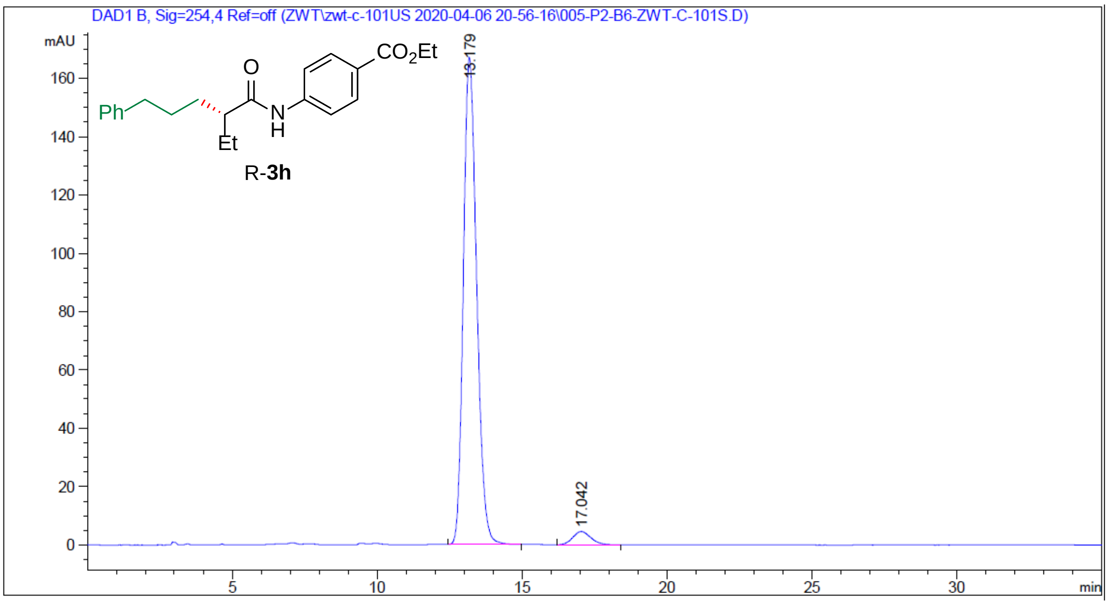

Peak #	RetTime [min]	Type	Width [min]	Area [mAU*s]	Height [mAU]	Area %
1	13.179	BB	0.4950	5308.60449	166.95876	96.4481
2	17.042	BB	0.5920	195.50285	4.50740	3.5519

Supplementary Figure 14 HPLC spectra of **3h**

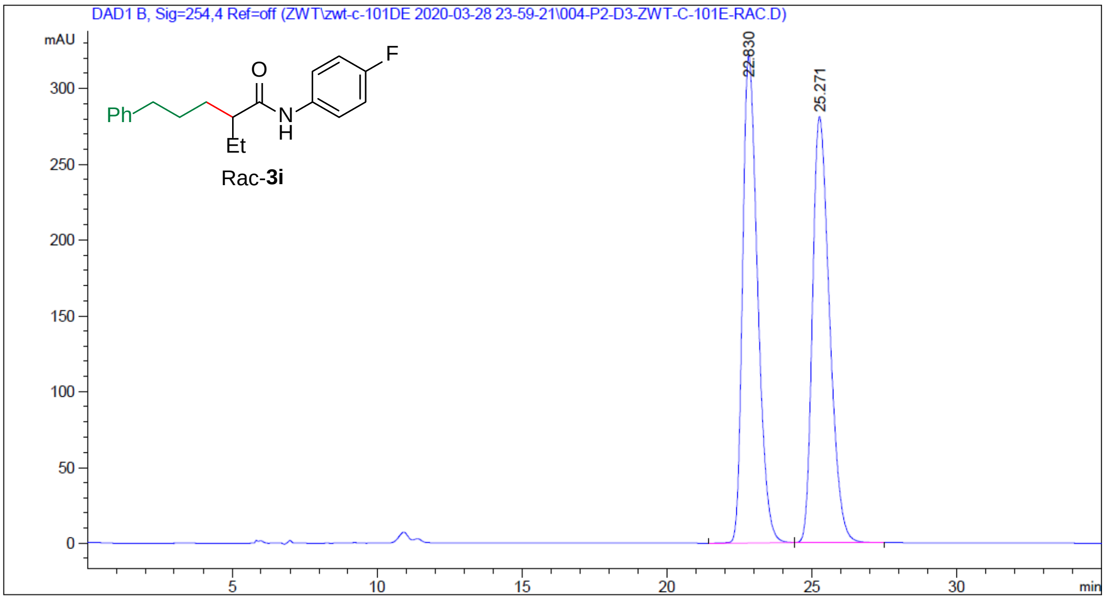

Peak #	RetTime [min]	Type	Width [min]	Area [mAU*s]	Height [mAU]	Area %
1	22.830	BB	0.5524	1.15264e4	321.42035	50.0381
2	25.271	BB	0.6338	1.15089e4	281.04349	49.9619

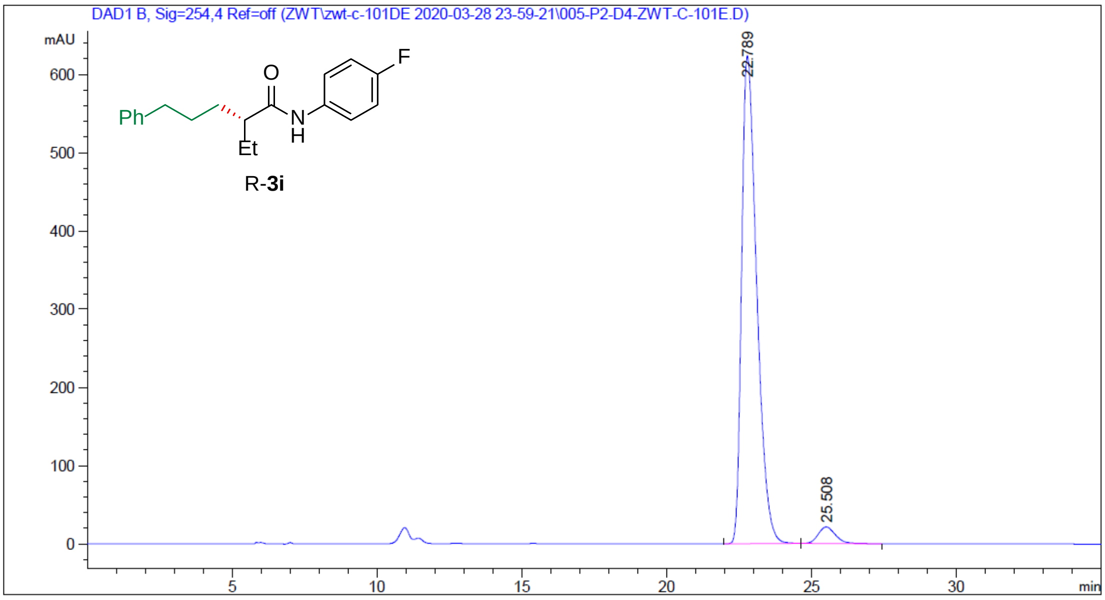

Peak #	RetTime [min]	Type	Width [min]	Area [mAU*s]	Height [mAU]	Area %
1	22.789	BB	0.5538	2.25445e4	623.50415	96.2719
2	25.508	BB	0.6311	873.03961	21.17085	3.7281

Supplementary Figure 15 HPLC spectra of **3i**

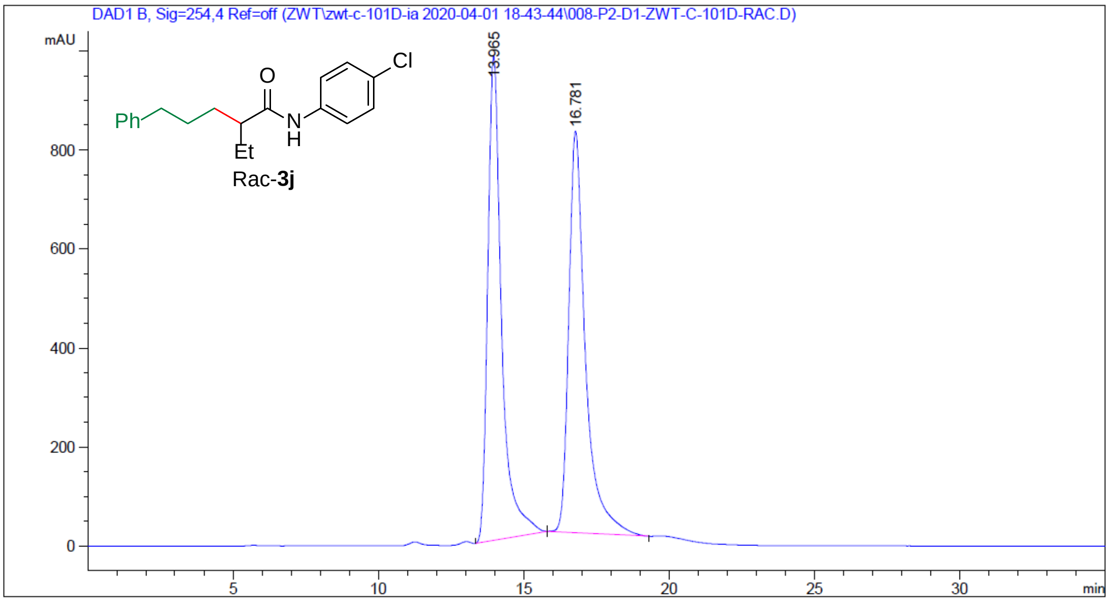

Peak #	RetTime [min]	Type	Width [min]	Area [mAU*s]	Height [mAU]	Area %
1	13.965	BB	0.4972	3.23059e4	978.36975	50.0504
2	16.781	BB	0.5875	3.22409e4	811.49512	49.9496

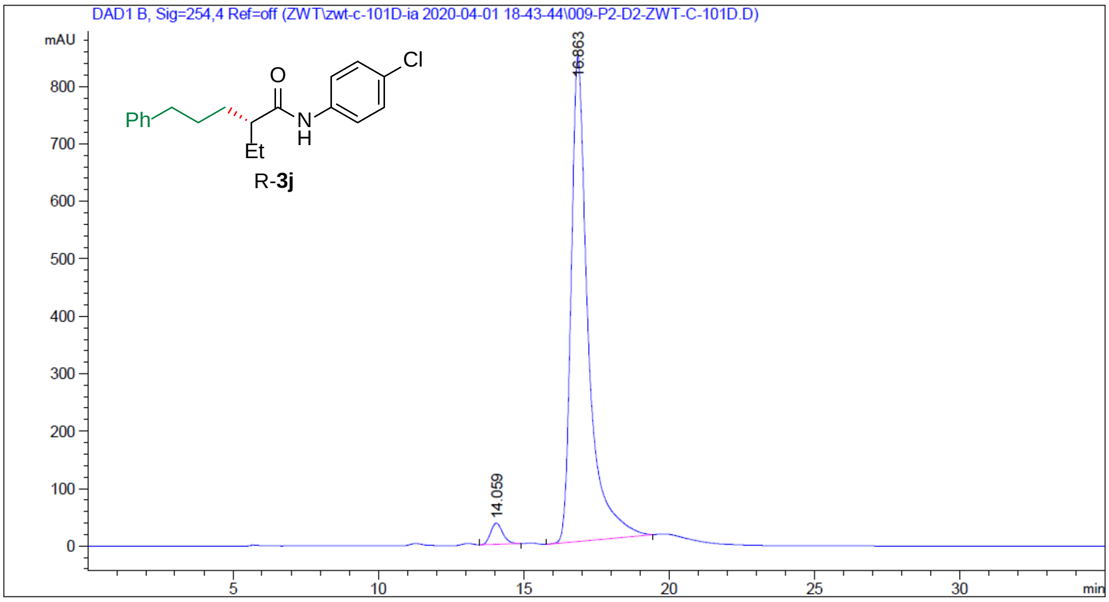

Peak #	RetTime [min]	Type	Width [min]	Area [mAU*s]	Height [mAU]	Area %
1	14.059	BB	0.4592	1089.44983	37.03378	3.0799
2	16.863	BB	0.5955	3.42837e4	844.74554	96.9201

Supplementary Figure 16 HPLC spectra of **3j**

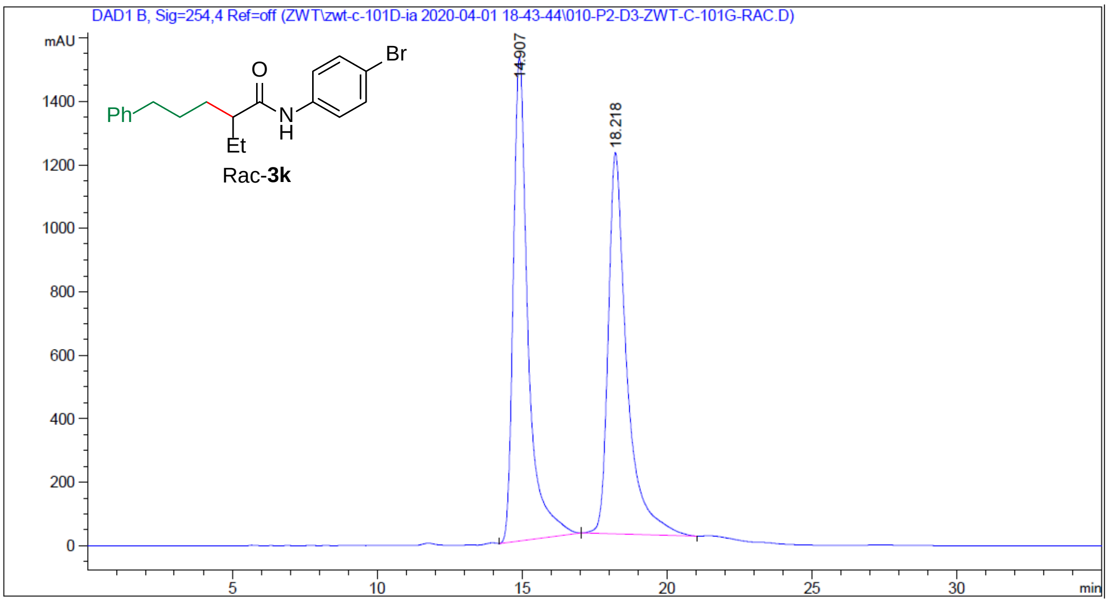

Peak #	RetTime [min]	Type	Width [min]	Area [mAU*s]	Height [mAU]	Area %
1	14.907	BB	0.5285	5.38520e4	1523.21558	50.2561
2	18.218	BB	0.6394	5.33031e4	1201.87756	49.7439

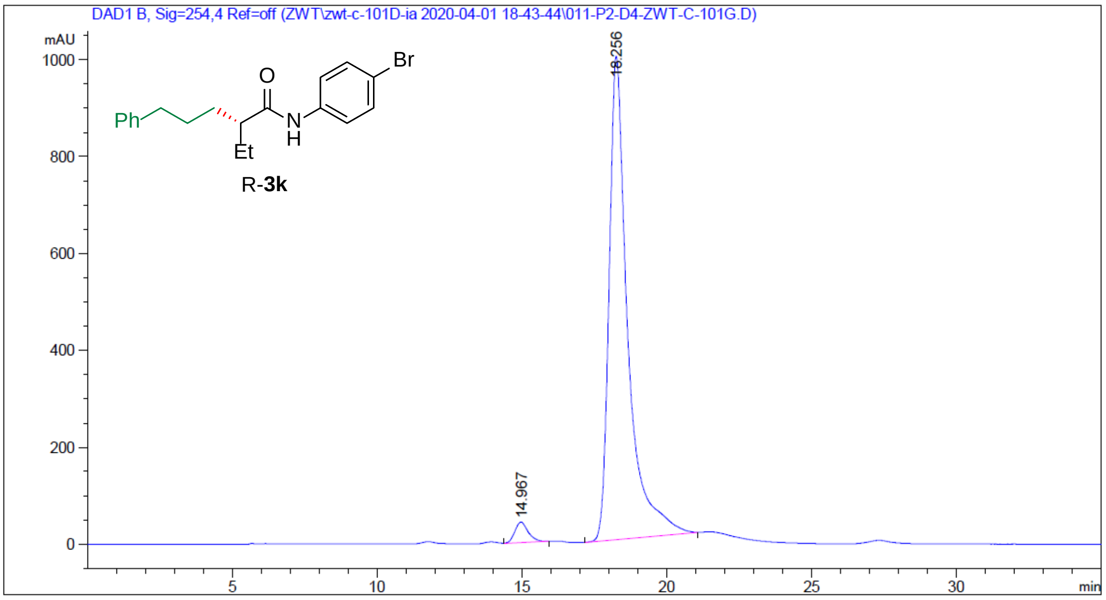

Peak #	RetTime [min]	Type	Width [min]	Area [mAU*s]	Height [mAU]	Area %
1	14.967	BB	0.4897	1349.23975	42.11822	2.9358
2	18.256	BB	0.6413	4.46096e4	998.35736	97.0642

Supplementary Figure 17 HPLC spectra of **3k**

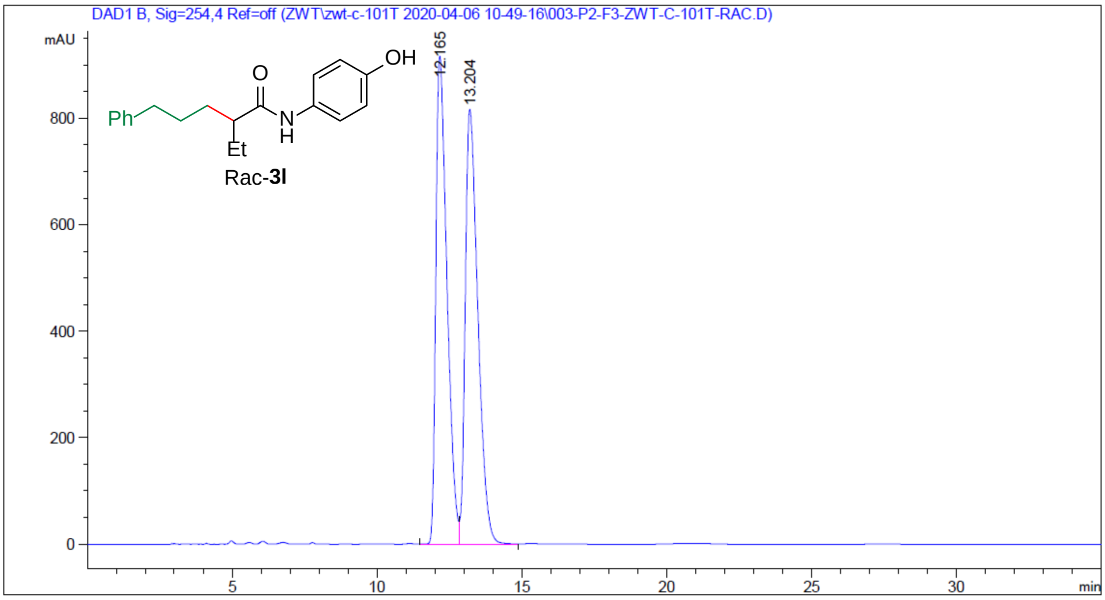

Peak #	RetTime [min]	Type	Width [min]	Area [mAU*s]	Height [mAU]	Area %
1	12.165	BV	0.4043	2.43833e4	916.81927	49.6745
2	13.204	VB	0.4632	2.47028e4	815.96069	50.3255

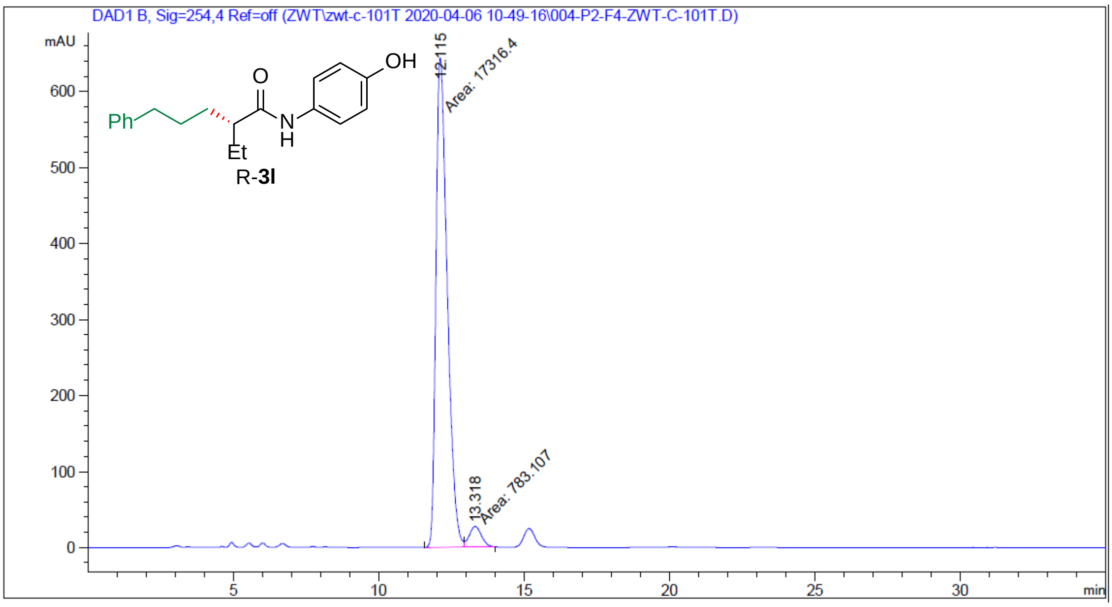

Peak #	RetTime [min]	Type	Width [min]	Area [mAU*s]	Height [mAU]	Area %
1	12.115	MM	0.4480	1.73164e4	644.22400	95.6733
2	13.318	MM	0.4855	783.10663	26.88452	4.3267

Supplementary Figure 18 HPLC spectra of **3l**

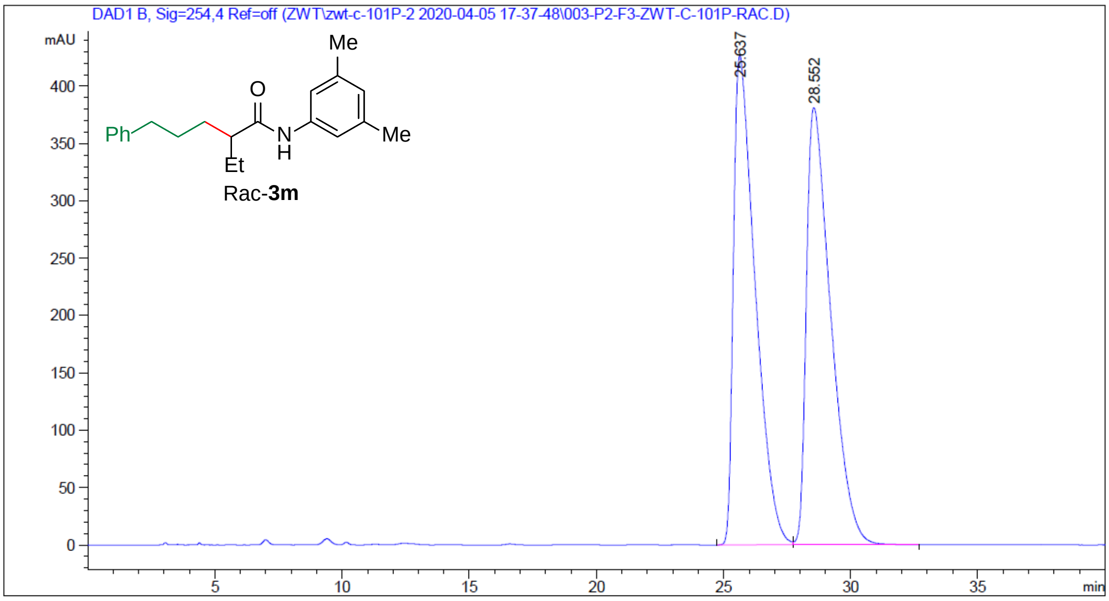

Peak #	RetTime [min]	Type	Width [min]	Area [mAU*s]	Height [mAU]	Area %
1	25.637	BV	0.9147	2.53309e4	426.60690	49.9308
2	28.552	VB	0.9894	2.54011e4	381.21448	50.0692

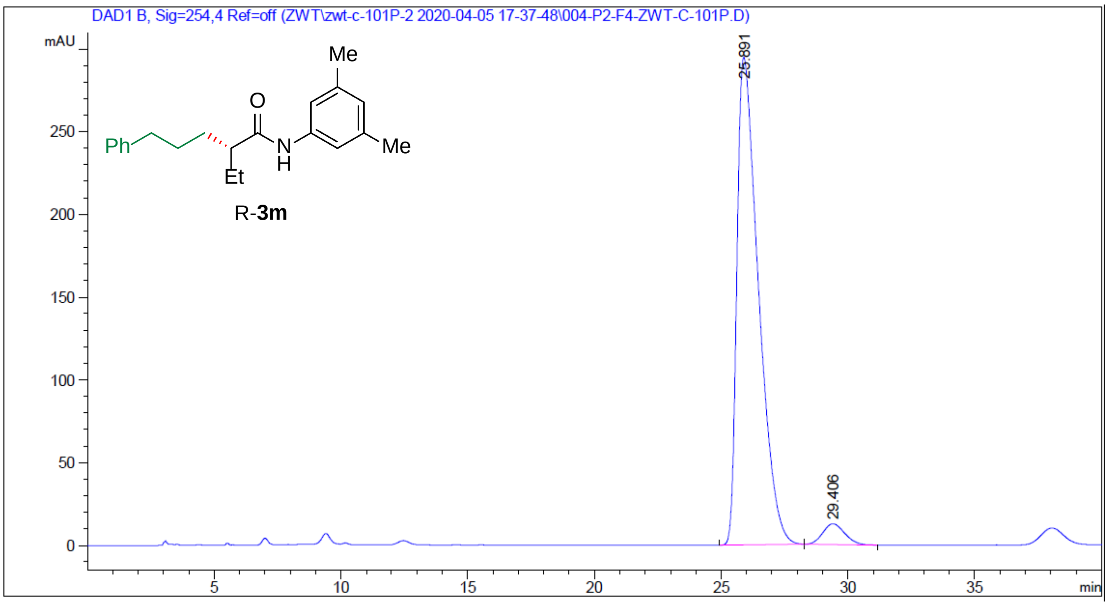

Peak #	RetTime [min]	Type	Width [min]	Area [mAU*s]	Height [mAU]	Area %
1	25.891	BB	0.8746	1.73006e4	294.81274	95.8471
2	29.406	BB	0.8703	749.60443	12.48888	4.1529

Supplementary Figure 19 HPLC spectra of **3m**

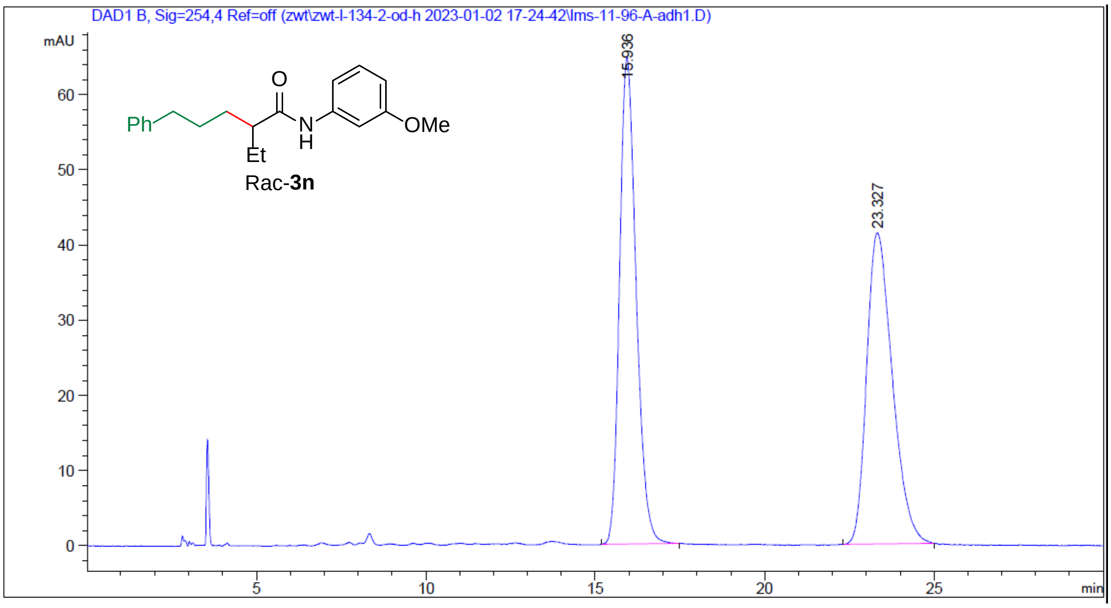

Peak #	RetTime [min]	Type	Width [min]	Area [mAU*s]	Height [mAU]	Area %
1	15.936	BB	0.5223	2199.16675	64.74591	50.2269
2	23.327	BB	0.7881	2179.30029	41.40022	49.7731

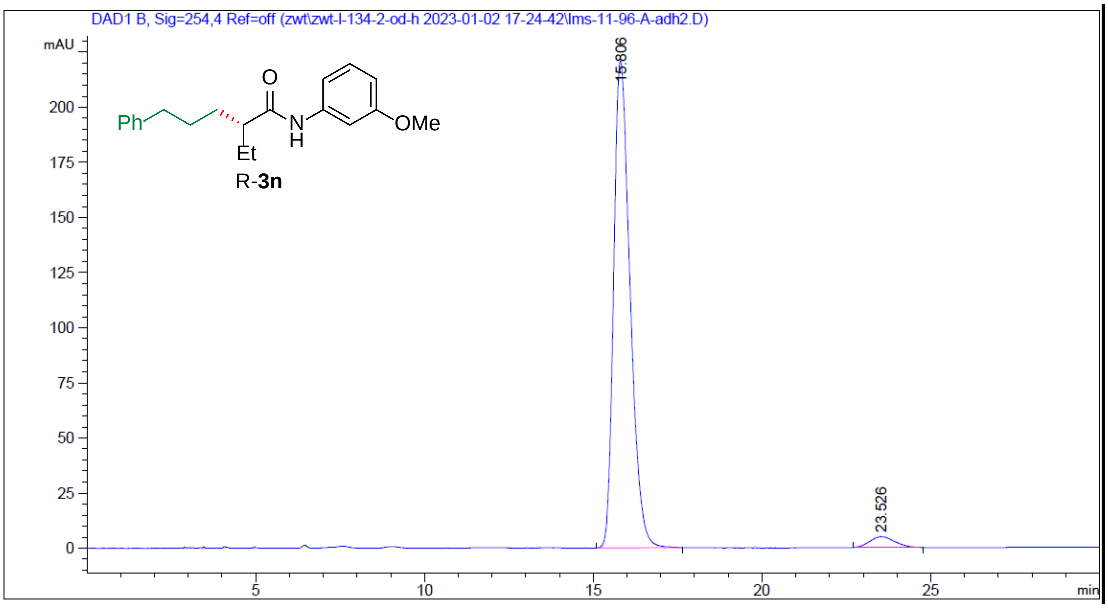

Peak #	RetTime [min]	Type	Width [min]	Area [mAU*s]	Height [mAU]	Area %
1	15.806	BB	0.5176	7447.54346	220.77335	96.7467
2	23.526	BB	0.6159	250.43678	4.85415	3.2533

Supplementary Figure 20 HPLC spectra of **3n**

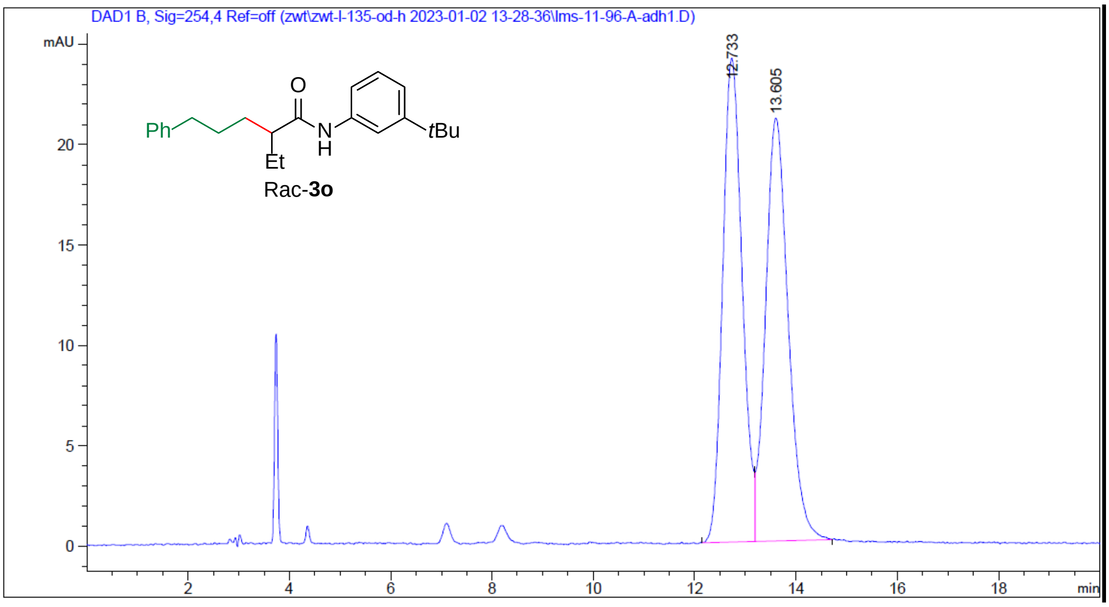

Peak #	RetTime [min]	Type	Width [min]	Area [mAU*s]	Height [mAU]	Area %
1	12.733	BV	0.4144	640.65497	24.08145	49.4102
2	13.605	VB	0.4757	655.94916	21.04028	50.5898

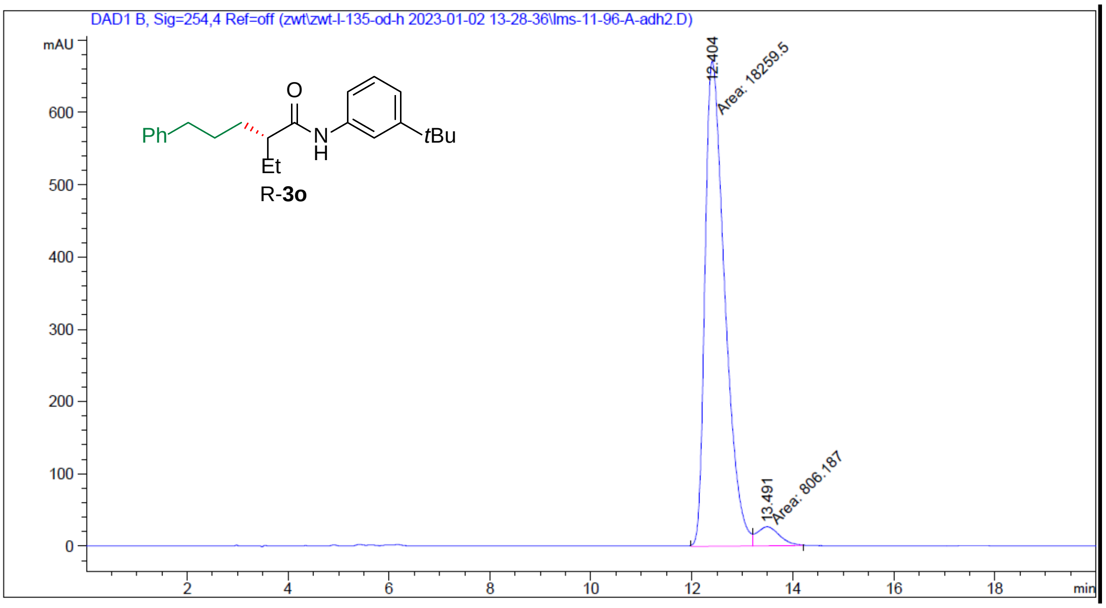

Peak #	RetTime [min]	Type	Width [min]	Area [mAU*s]	Height [mAU]	Area %
1	12.404	MF	0.4532	1.82595e4	671.43463	95.7715
2	13.491	FM	0.5145	806.18683	26.11769	4.2285

Supplementary Figure 21 HPLC spectra of **3o**

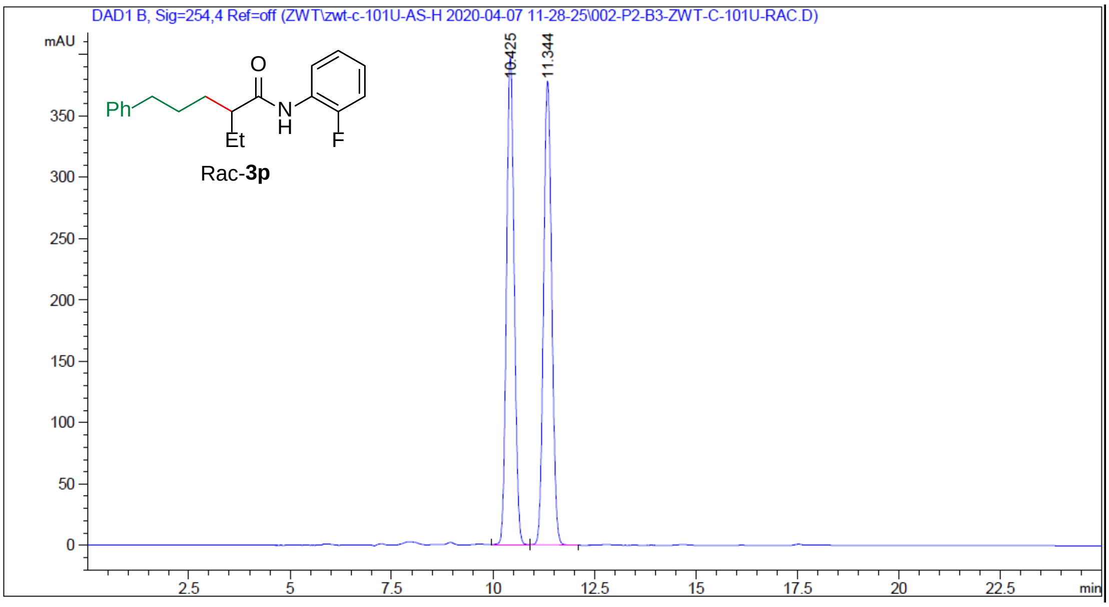

Peak #	RetTime [min]	Type	Width [min]	Area [mAU*s]	Height [mAU]	Area %
1	10.425	BB	0.2080	5242.26660	397.12839	49.7615
2	11.344	BB	0.2214	5292.50977	377.92068	50.2385

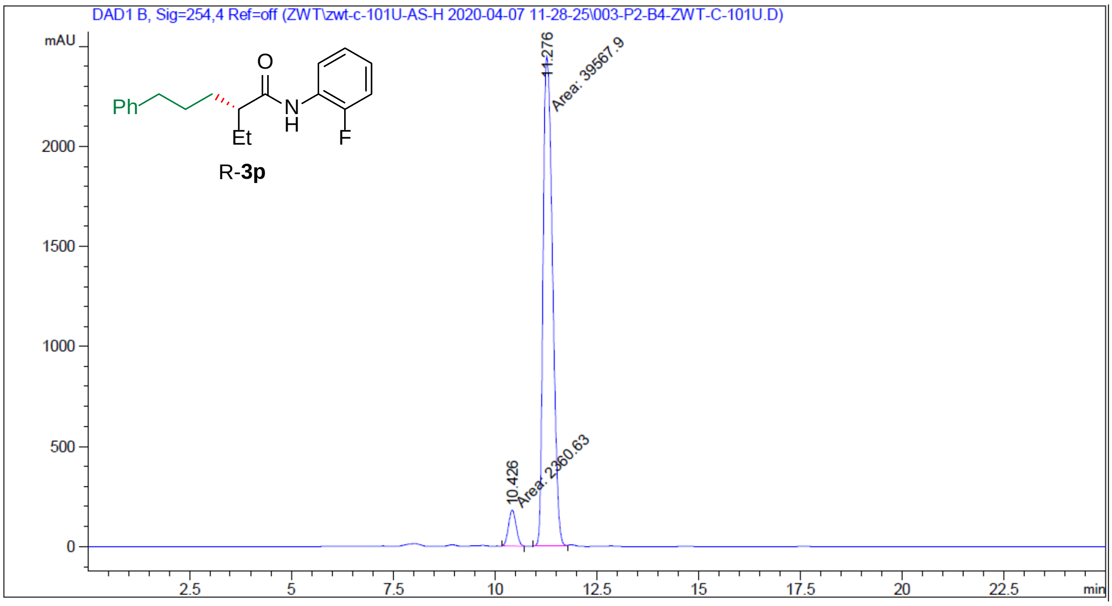

Peak #	RetTime [min]	Type	Width [min]	Area [mAU*s]	Height [mAU]	Area %
1	10.426	MM	0.2192	2360.62939	179.48854	5.6301
2	11.276	MM	0.2697	3.95679e4	2445.25635	94.3699

Supplementary Figure 22 HPLC spectra of **3p**

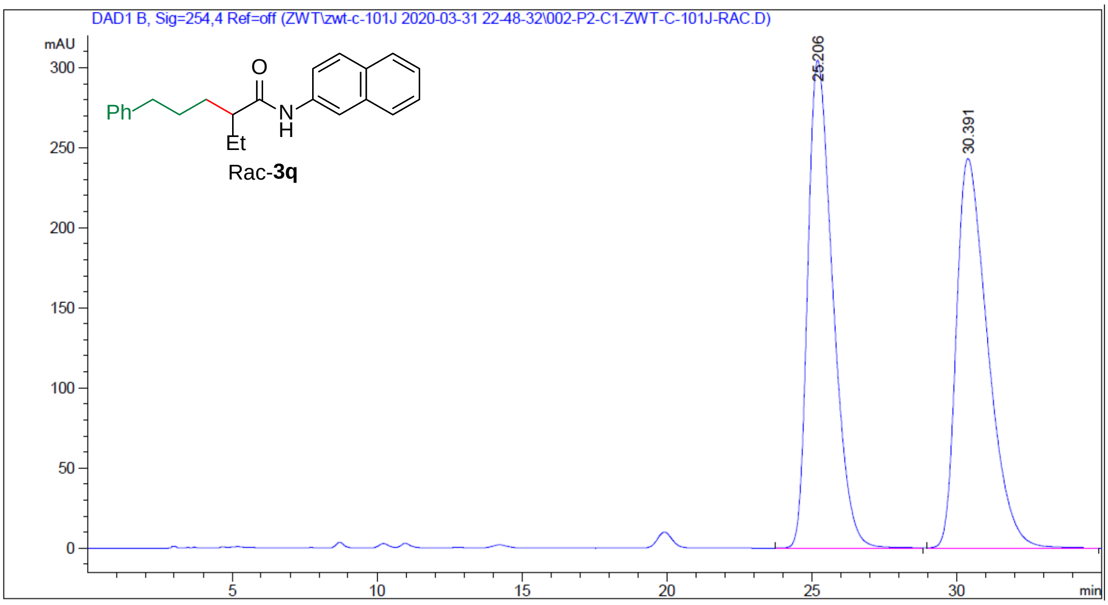

Peak #	RetTime [min]	Type	Width [min]	Area [mAU*s]	Height [mAU]	Area %
1	25.206	BB	0.9029	1.82341e4	304.37375	49.9630
2	30.391	BBA	1.1191	1.82612e4	242.88638	50.0370

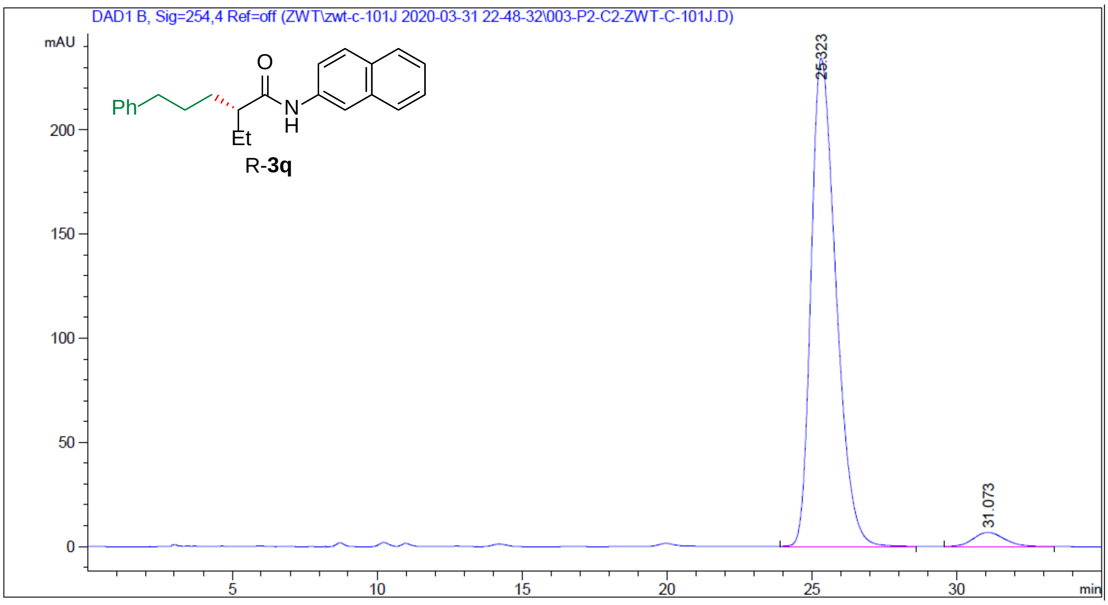

Peak #	RetTime [min]	Type	Width [min]	Area [mAU*s]	Height [mAU]	Area %
1	25.323	BB	0.9293	1.39972e4	234.17549	96.4990
2	31.073	BB	0.8991	507.81403	6.78548	3.5010

Supplementary Figure 23 HPLC spectra of **3q**

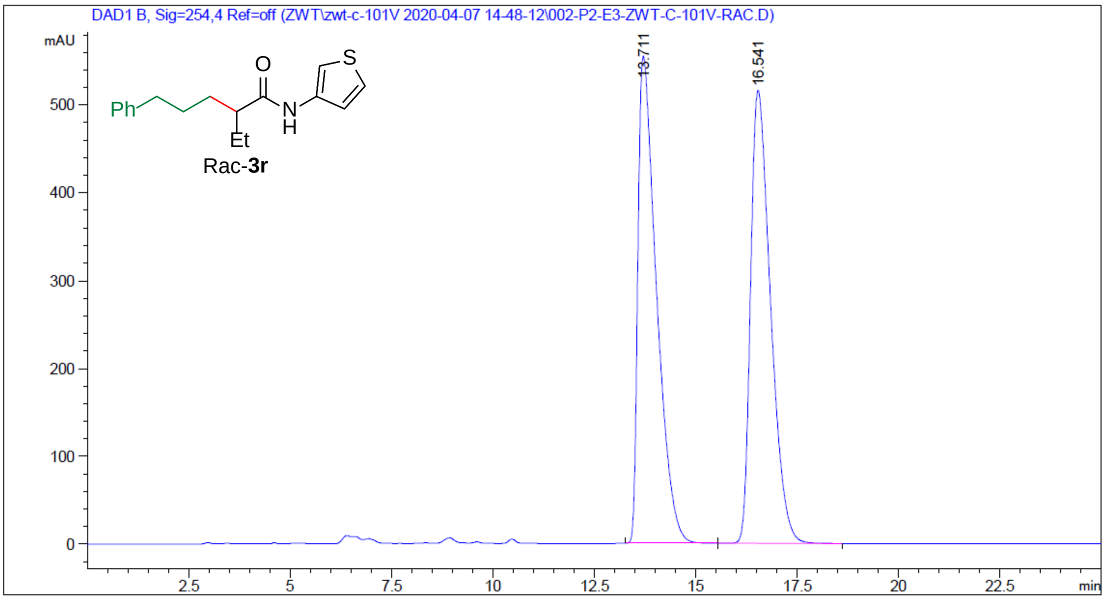

Peak #	RetTime [min]	Type	Width [min]	Area [mAU*s]	Height [mAU]	Area %
1	13.711	BB	0.4739	1.76044e4	555.18811	49.9640
2	16.541	BB	0.5184	1.76298e4	516.23499	50.0360

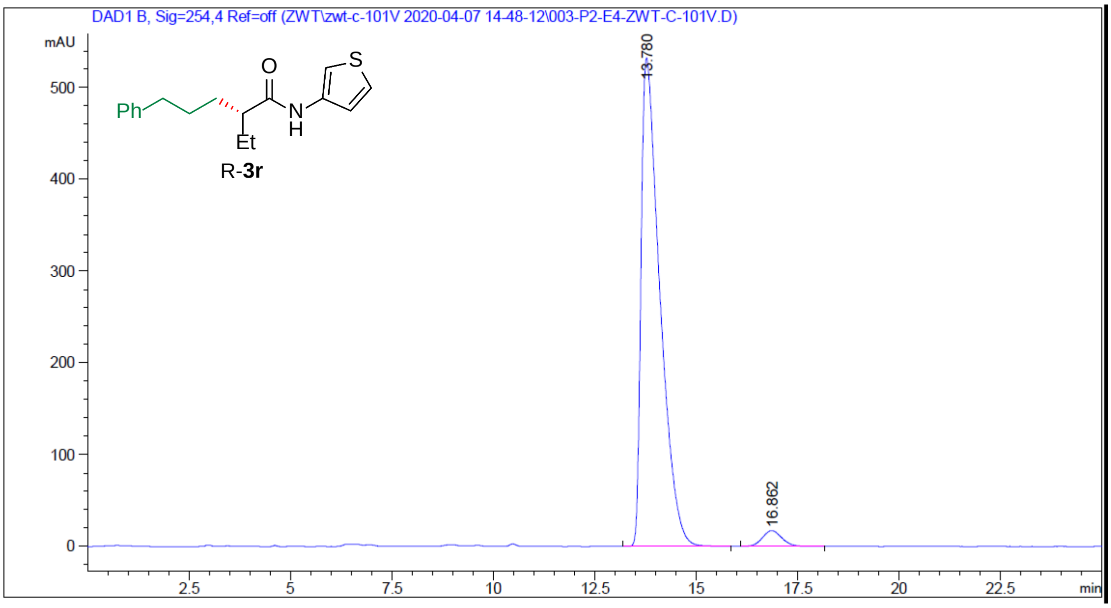

Peak #	RetTime [min]	Type	Width [min]	Area [mAU*s]	Height [mAU]	Area %
1	13.780	BB	0.4764	1.70766e4	532.06207	96.8401
2	16.862	BB	0.5129	557.21228	16.89125	3.1599

Supplementary Figure 24 HPLC spectra of **3r**

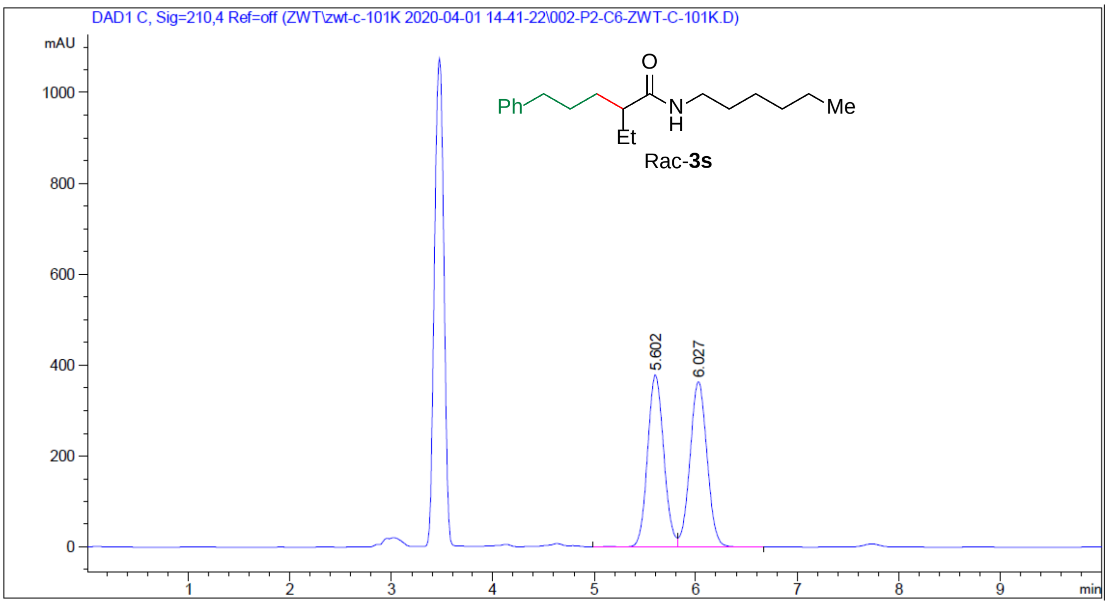

Peak #	RetTime [min]	Type	Width [min]	Area [mAU*s]	Height [mAU]	Area %
1	5.602	VV R	0.1732	4190.18652	378.49869	49.6929
2	6.027	VB	0.1820	4241.97314	363.90186	50.3071

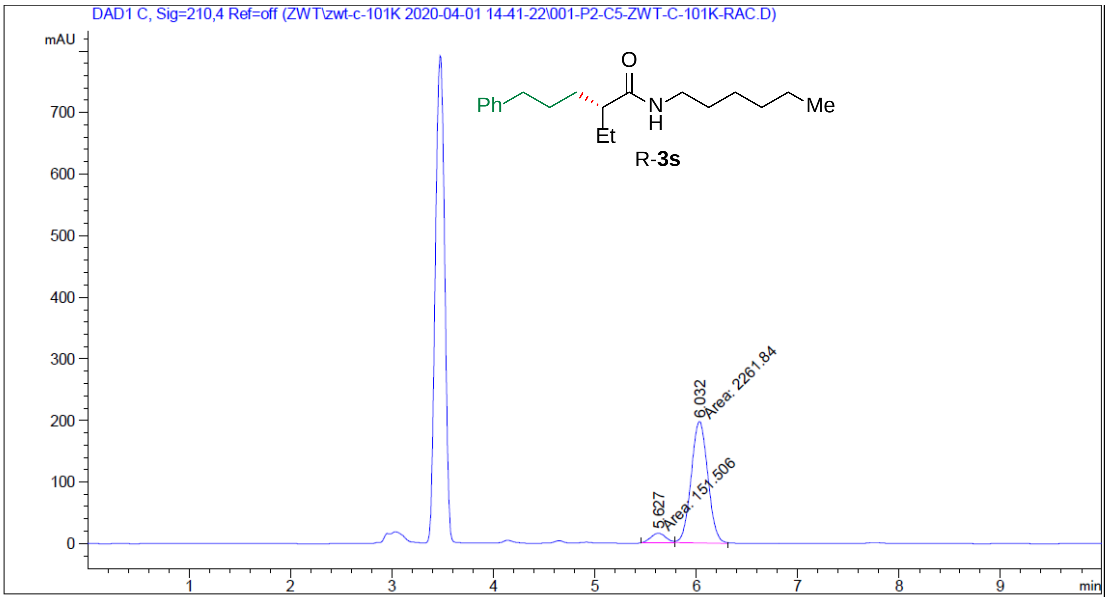

Peak #	RetTime [min]	Type	Width [min]	Area [mAU*s]	Height [mAU]	Area %
1	5.627	MM	0.1686	151.50616	14.97715	6.2778
2	6.032	MM	0.1907	2261.84155	197.66463	93.7222

Supplementary Figure 25 HPLC spectra of **3s**

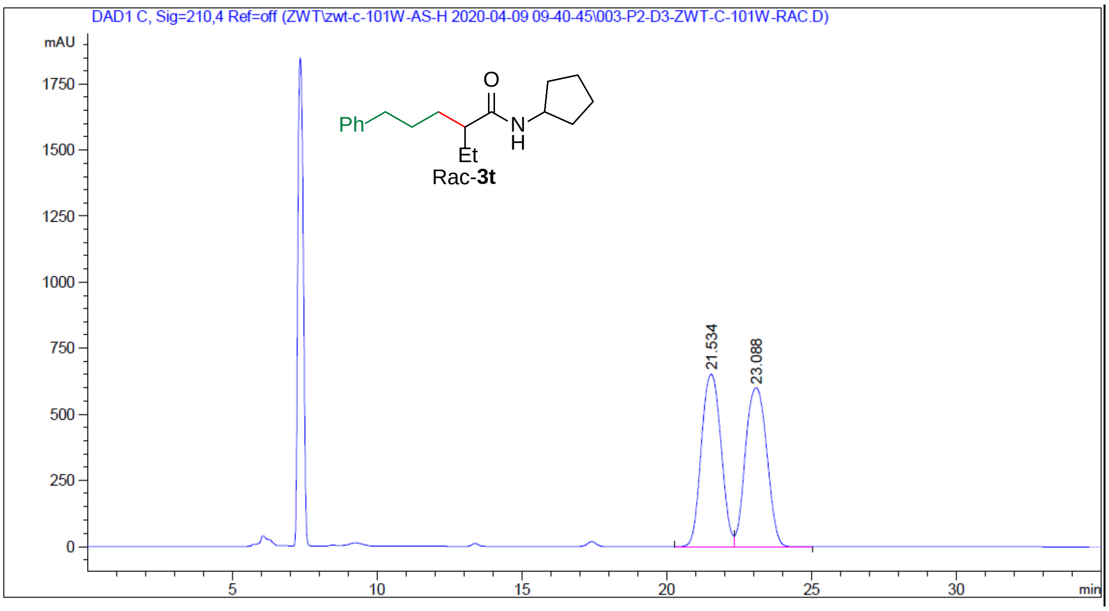

Peak #	RetTime [min]	Type	Width [min]	Area [mAU*s]	Height [mAU]	Area %
1	21.534	BV	0.7701	3.08833e4	652.62695	49.8692
2	23.088	VB	0.8445	3.10453e4	601.19342	50.1308

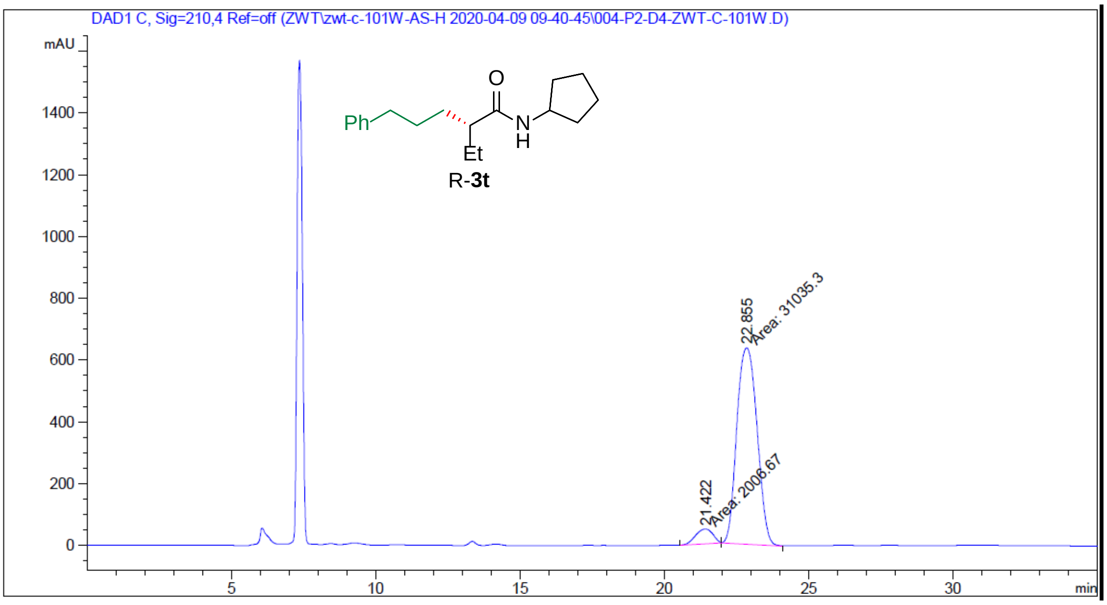

Peak #	RetTime [min]	Type	Width [min]	Area [mAU*s]	Height [mAU]	Area %
1	21.422	MM	0.7006	2006.66614	47.73684	6.0731
2	22.855	MM	0.8132	3.10353e4	636.09412	93.9269

Supplementary Figure 26 HPLC spectra of **3t**

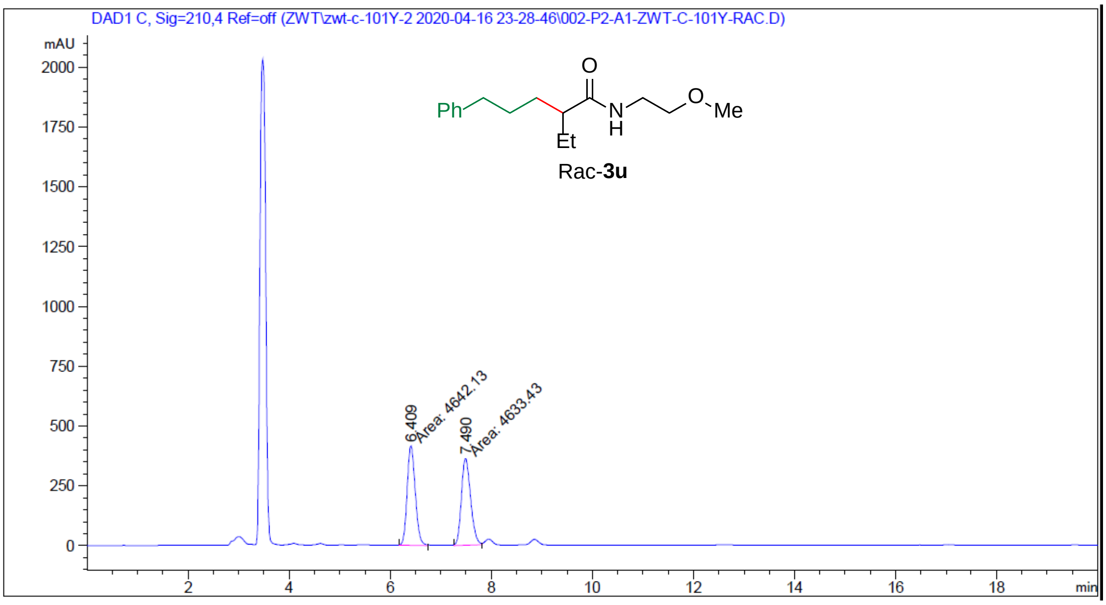

Peak #	RetTime [min]	Type	Width [min]	Area [mAU*s]	Height [mAU]	Area %
1	6.409	MM	0.1857	4642.12842	416.72531	50.0469
2	7.490	MM	0.2128	4633.43457	362.91632	49.9531

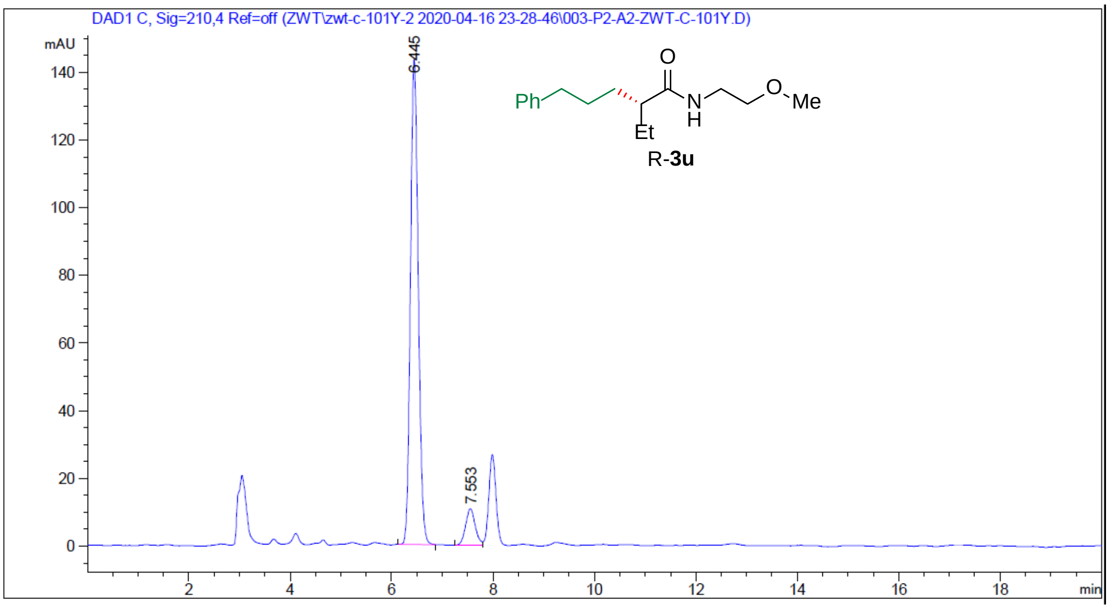

Peak #	RetTime [min]	Type	Width [min]	Area [mAU*s]	Height [mAU]	Area %
1	6.445	BB	0.1666	1530.76221	143.28091	91.5241
2	7.553	BV	0.2037	141.76166	10.76017	8.4759

Supplementary Figure 27 HPLC spectra of **3u**

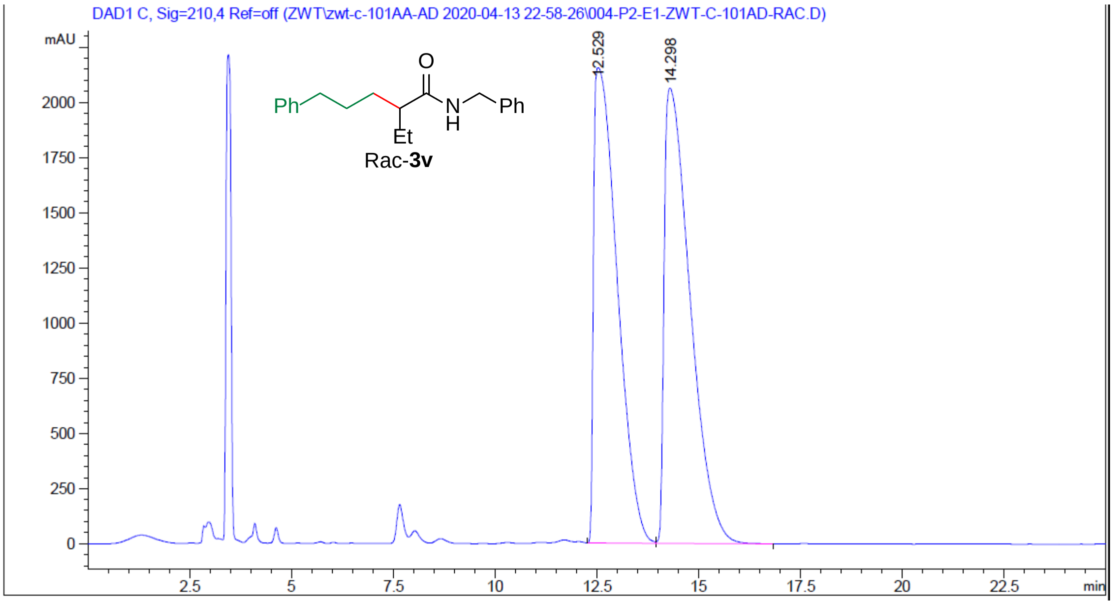

Peak #	RetTime [min]	Type	Width [min]	Area [mAU*s]	Height [mAU]	Area %
1	12.529	BV	0.6222	8.64497e4	2154.06006	48.6374
2	14.298	VB	0.6704	9.12935e4	2062.39355	51.3626

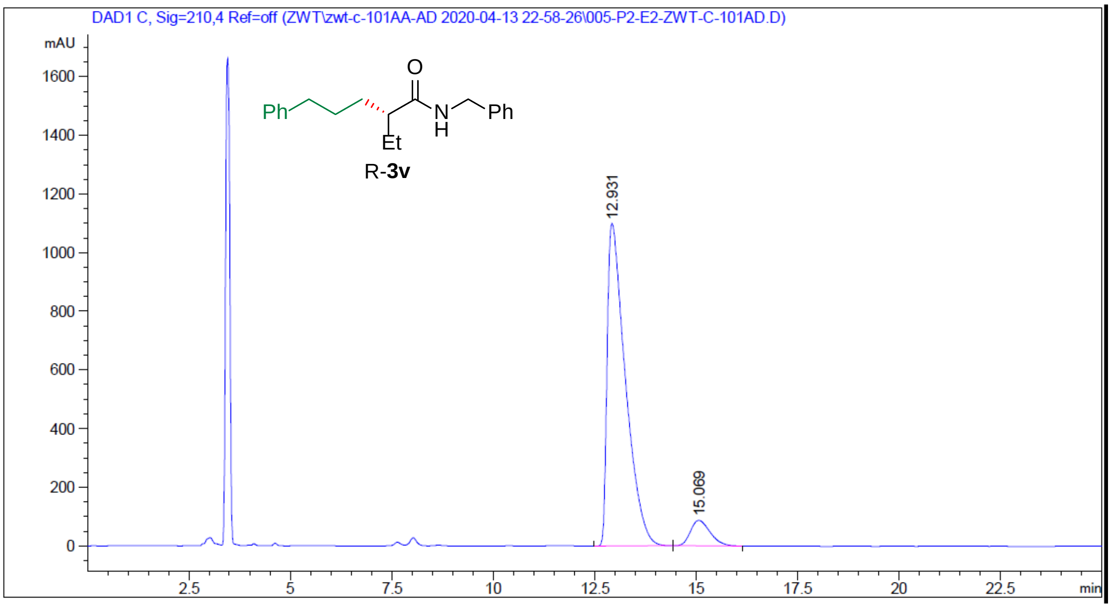

Peak #	RetTime [min]	Type	Width [min]	Area [mAU*s]	Height [mAU]	Area %
1	12.931	BB	0.4619	3.43126e4	1100.08618	92.1288
2	15.069	BB	0.5150	2931.56079	87.03236	7.8712

Supplementary Figure 28 HPLC spectra of **3v**

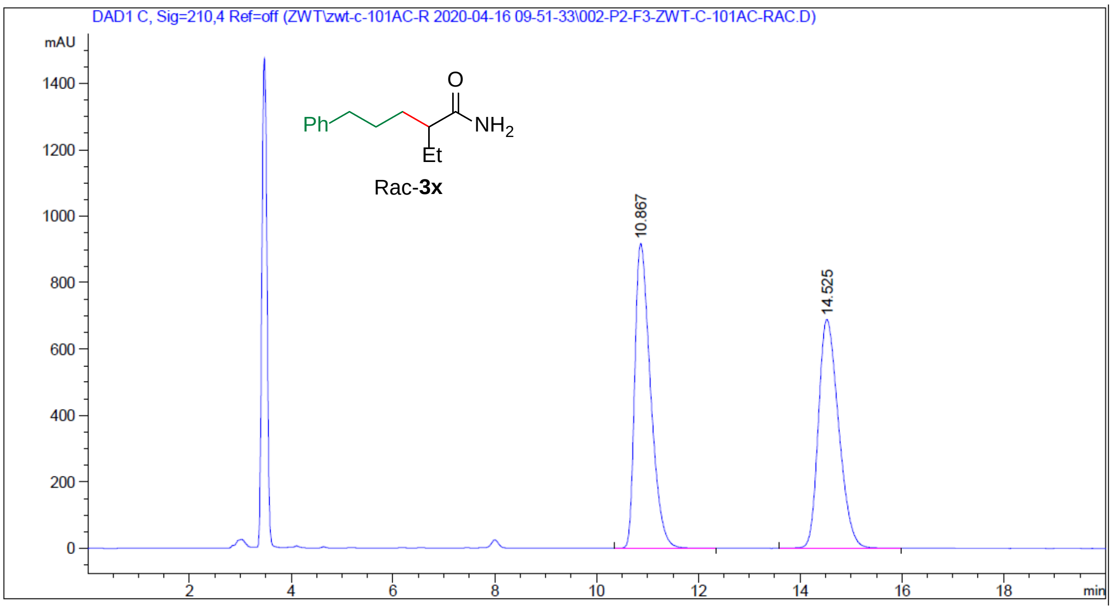

Peak #	RetTime [min]	Type	Width [min]	Area [mAU*s]	Height [mAU]	Area %
1	10.867	BB	0.3304	1.96996e4	917.91217	50.9372
2	14.525	BB	0.4255	1.89747e4	688.47717	49.0628

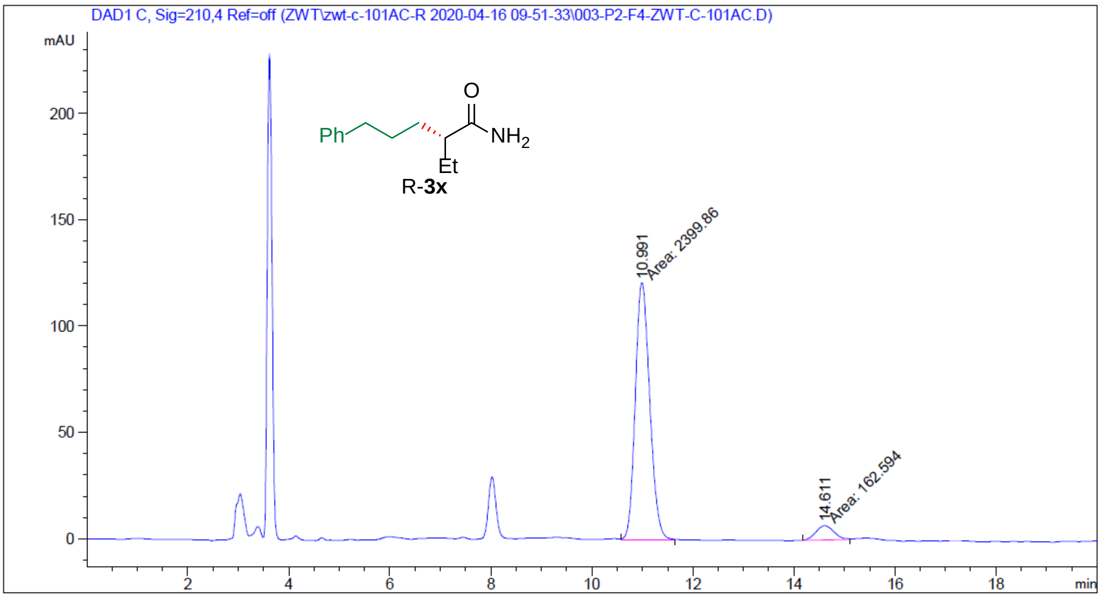

Peak #	RetTime [min]	Type	Width [min]	Area [mAU*s]	Height [mAU]	Area %
1	10.991	MM	0.3308	2399.86011	120.91199	93.6547
2	14.611	MM	0.4105	162.59424	6.60224	6.3453

Supplementary Figure 29 HPLC spectra of **3x**

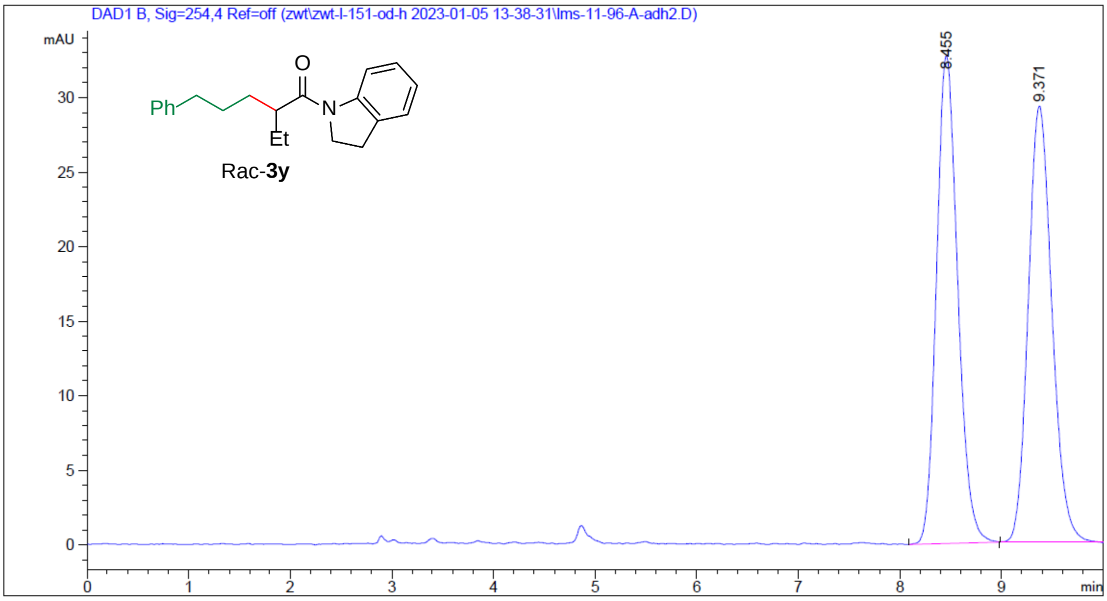

Peak #	RetTime [min]	Type	Width [min]	Area [mAU*s]	Height [mAU]	Area %
1	8.455	BB	0.2235	475.53006	32.72454	50.0292
2	9.371	BB	0.2514	474.97464	29.26450	49.9708

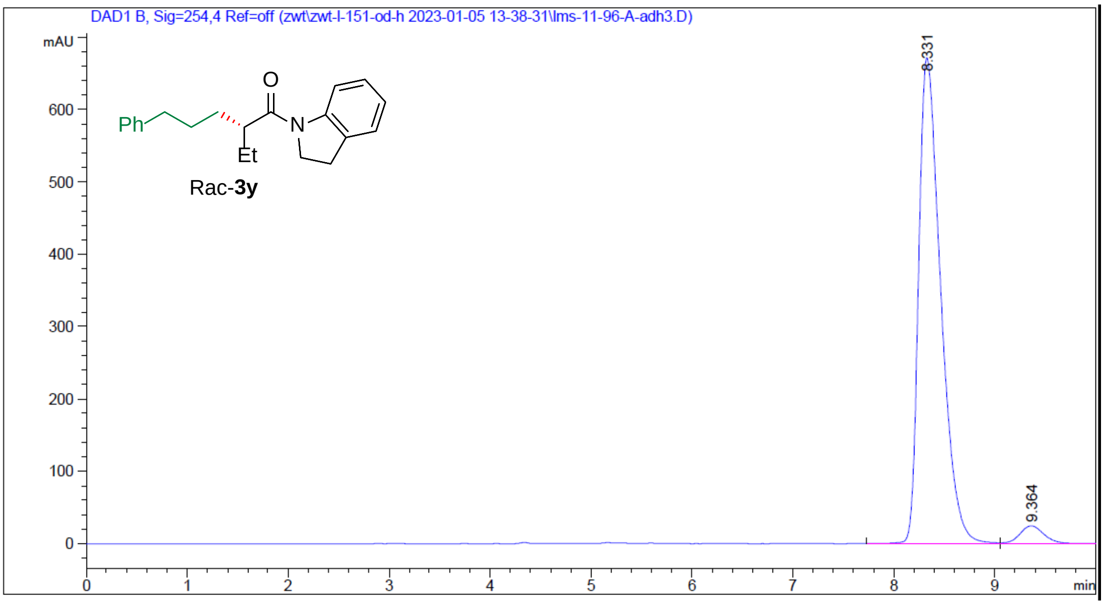

Peak #	RetTime [min]	Type	Width [min]	Area [mAU*s]	Height [mAU]	Area %
1	8.331	BV	0.2305	1.01557e4	671.17267	96.1341
2	9.364	VB	0.2566	408.40109	24.48825	3.8659

Supplementary Figure 30 HPLC spectra of **3y**

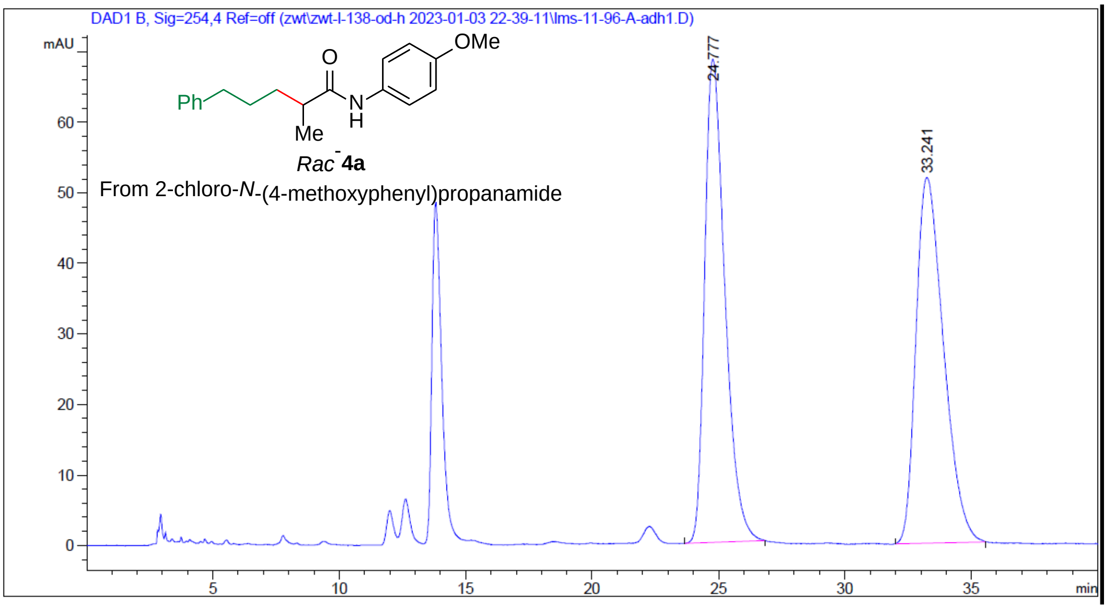

Peak #	RetTime [min]	Type	Width [min]	Area [mAU*s]	Height [mAU]	Area %
1	24.777	BB	0.8087	3810.32520	68.52283	49.2242
2	33.241	BB	1.1026	3930.43750	51.83617	50.7758

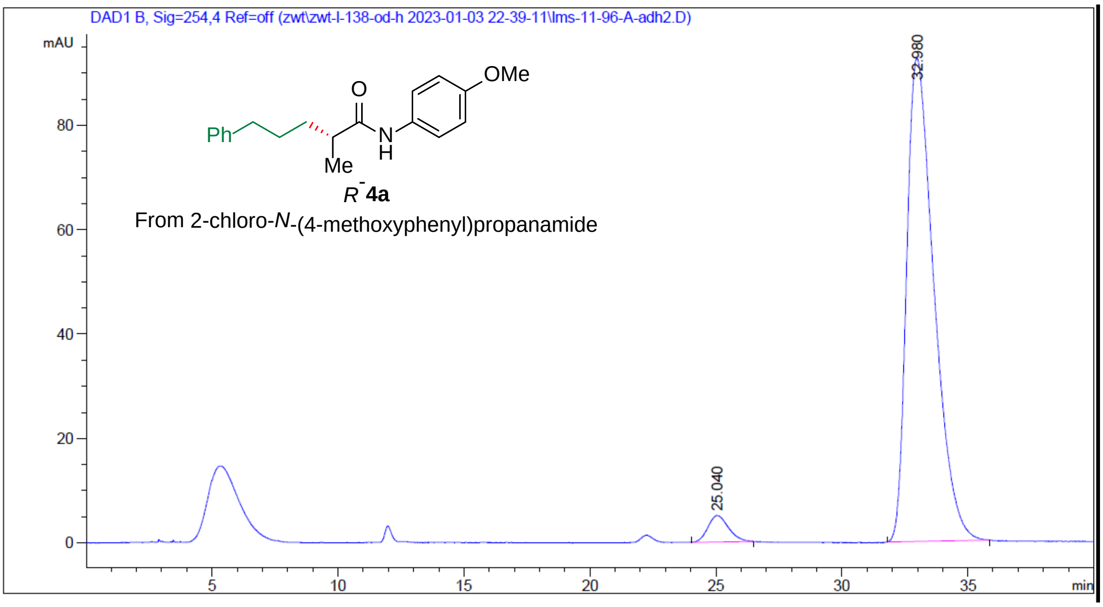

Peak #	RetTime [min]	Type	Width [min]	Area [mAU*s]	Height [mAU]	Area %
1	25.040	BB	0.6733	288.42621	5.10419	3.9722
2	32.980	BB	1.1005	6972.73730	92.59969	96.0278

Supplementary Figure 31 HPLC spectra of **4a**

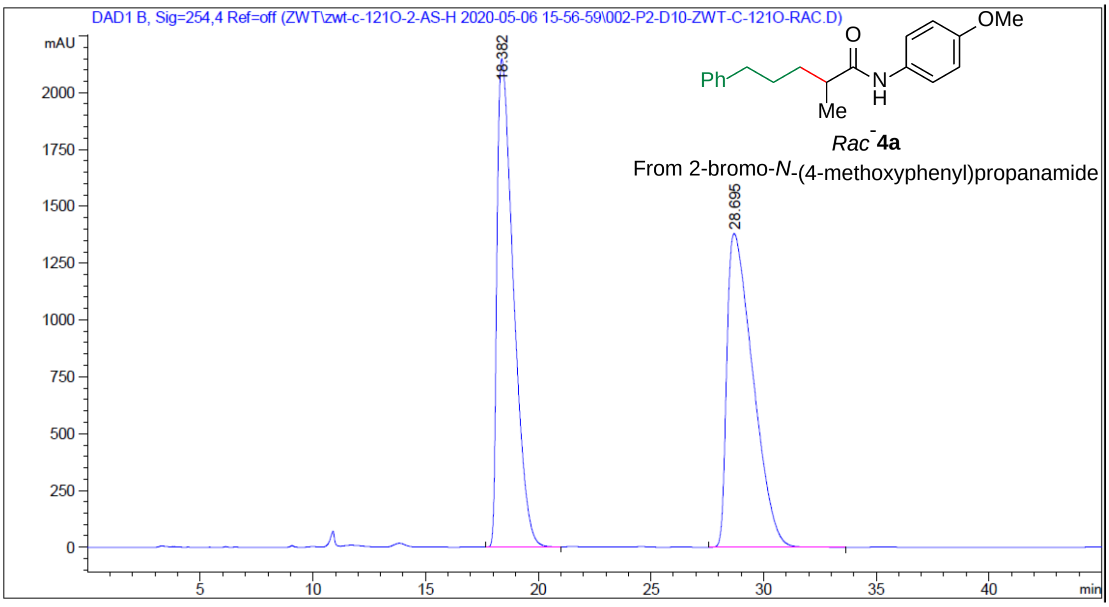

Peak #	RetTime [min]	Type	Width [min]	Area [mAU*s]	Height [mAU]	Area %
1	18.382	BB	0.7634	1.08927e5	2148.66162	49.6638
2	28.695	BB	1.1990	1.10402e5	1380.46460	50.3362

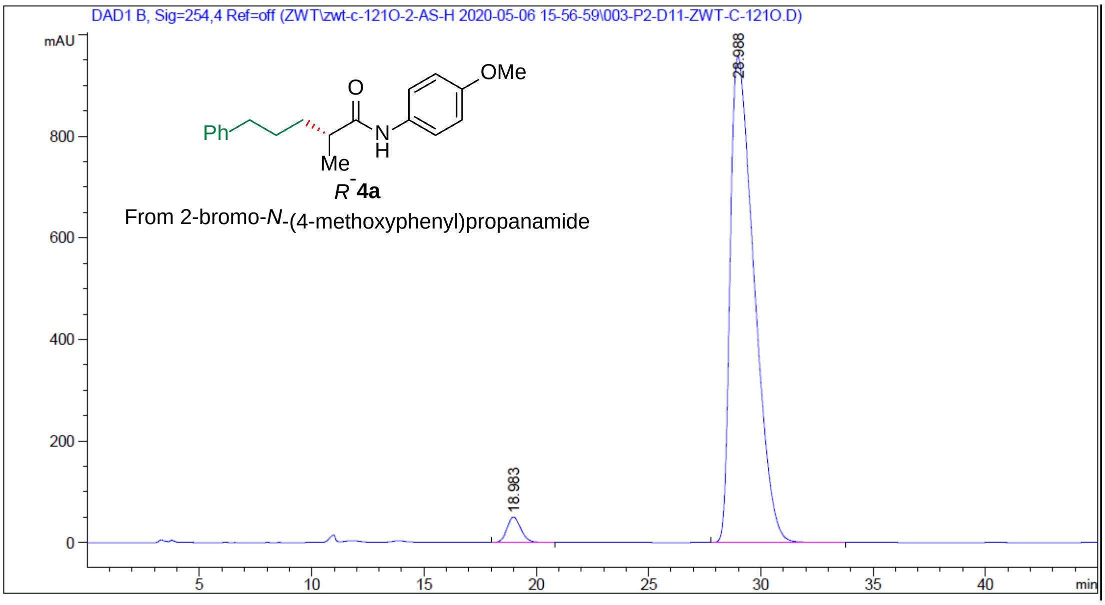

Peak #	RetTime [min]	Type	Width [min]	Area [mAU*s]	Height [mAU]	Area %
1	18.983	BB	0.6630	2132.21118	49.84698	2.9361
2	28.988	BB	1.1135	7.04891e4	956.85345	97.0639

Supplementary Figure 32 HPLC spectra of **4a**

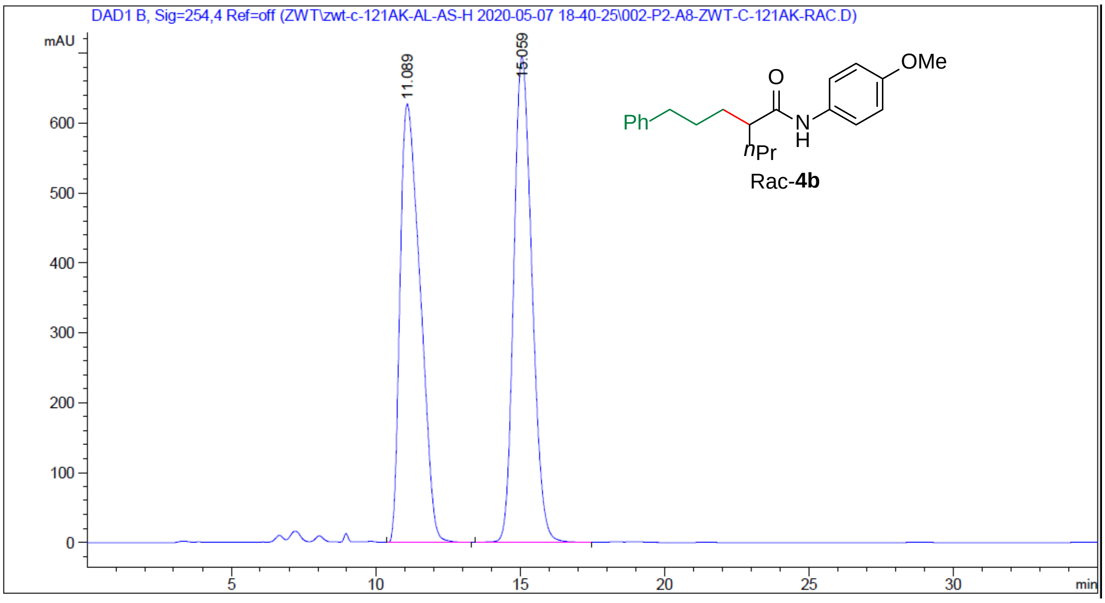

Peak #	RetTime [min]	Type	Width [min]	Area [mAU*s]	Height [mAU]	Area %
1	11.089	BB	0.7523	3.03243e4	626.91461	49.9613
2	15.059	BB	0.6821	3.03712e4	694.59851	50.0387

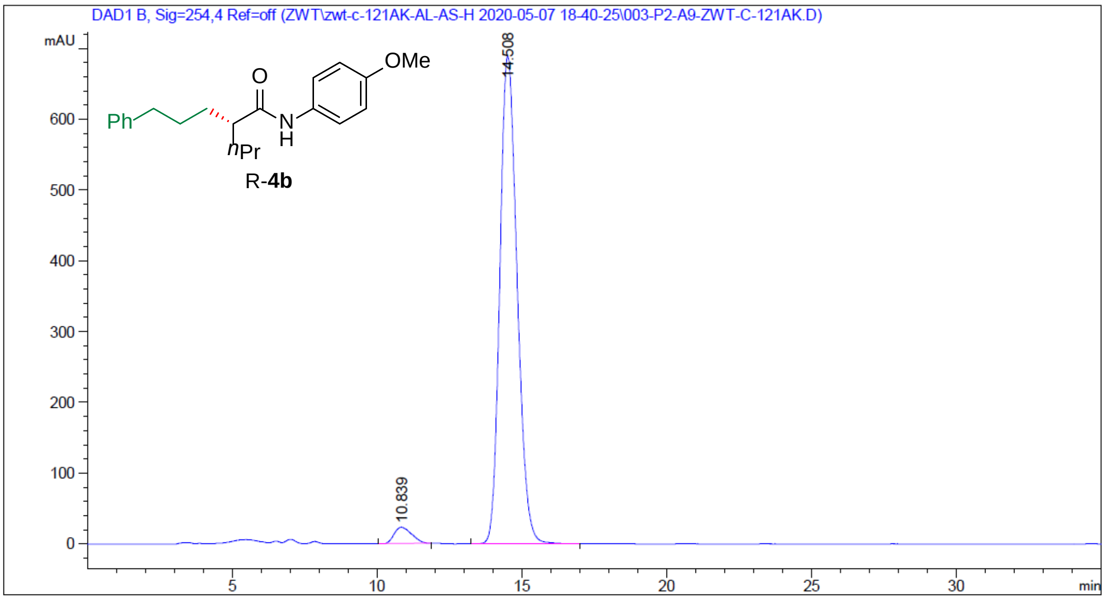

Peak #	RetTime [min]	Type	Width [min]	Area [mAU*s]	Height [mAU]	Area %
1	10.839	BB	0.6490	961.00726	23.02319	3.2570
2	14.508	BB	0.6435	2.85451e4	688.85291	96.7430

Supplementary Figure 33 HPLC spectra of **4b**

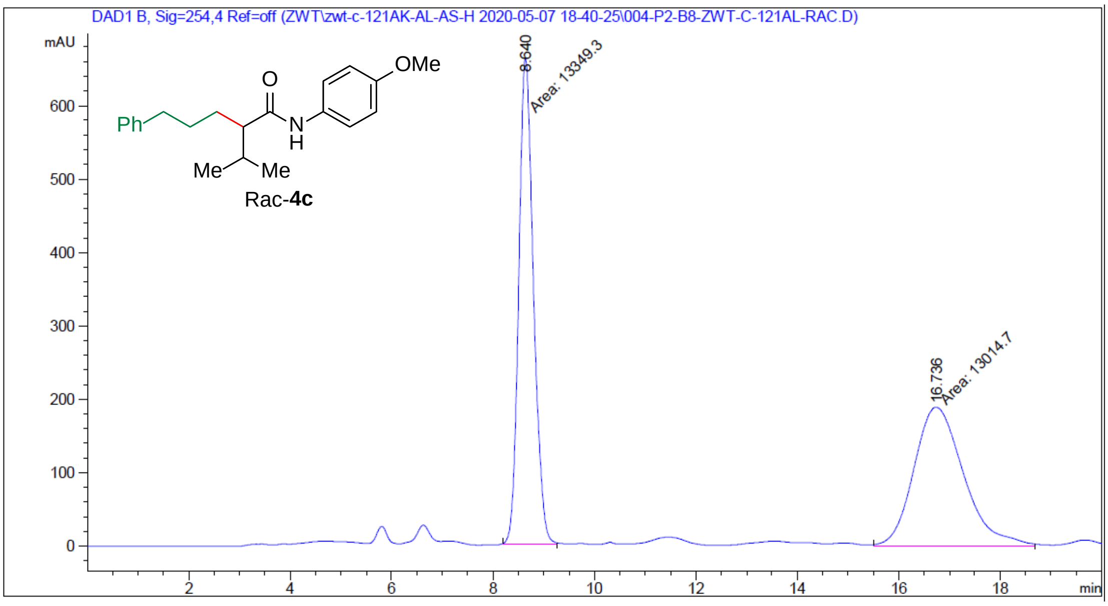

Peak #	RetTime [min]	Type	Width [min]	Area [mAU*s]	Height [mAU]	Area %
1	8.640	MM	0.3359	1.33493e4	662.26971	50.6346
2	16.736	MM	1.1425	1.30147e4	189.85638	49.3654

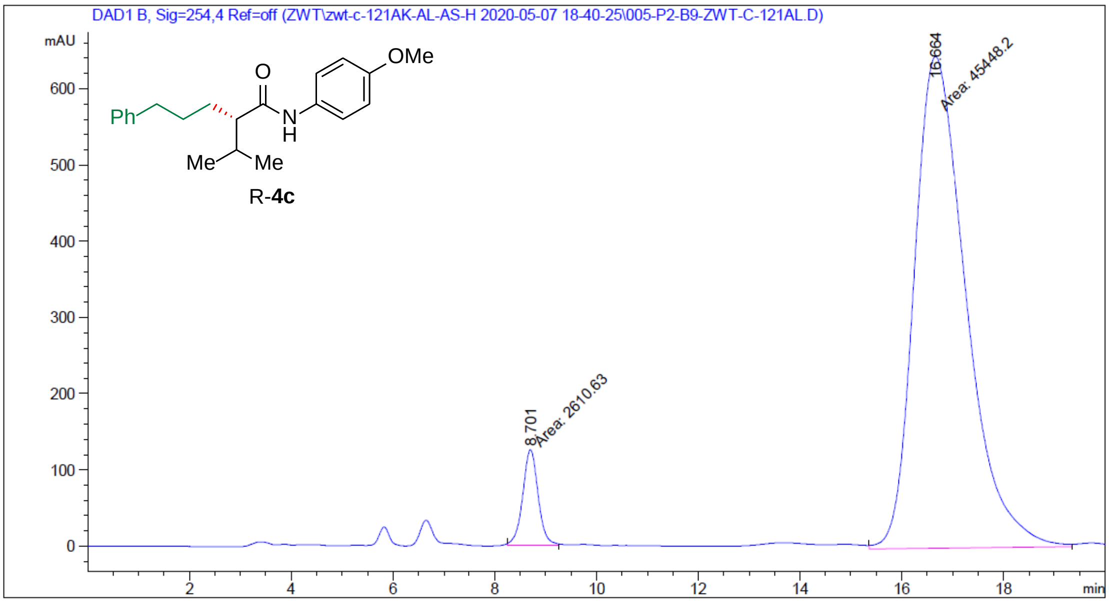

Peak #	RetTime [min]	Type	Width [min]	Area [mAU*s]	Height [mAU]	Area %
1	8.701	MM	0.3486	2610.62842	124.79862	5.4321
2	16.664	MM	1.1743	4.54482e4	645.02728	94.5679

Supplementary Figure 34 HPLC spectra of **4c**

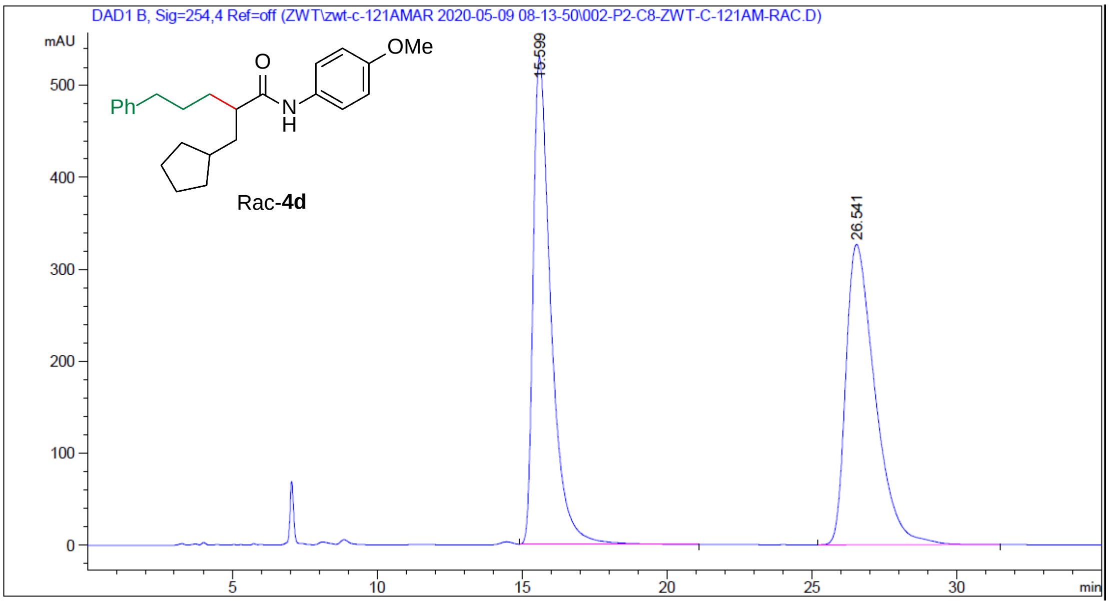

Peak #	RetTime [min]	Type	Width [min]	Area [mAU*s]	Height [mAU]	Area %
1	15.599	BB	0.6376	2.25199e4	530.17261	49.5881
2	26.541	BB	1.0599	2.28940e4	326.54953	50.4119

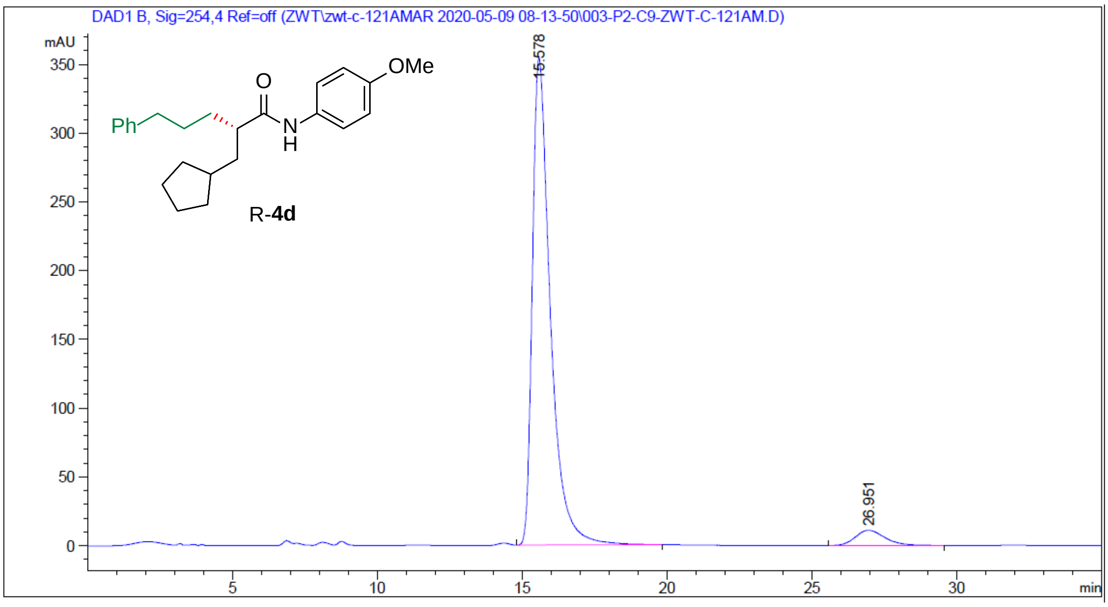

Peak #	RetTime [min]	Type	Width [min]	Area [mAU*s]	Height [mAU]	Area %
1	15.578	BB	0.6453	1.52867e4	354.35040	95.1440
2	26.951	BB	0.9278	780.21381	11.07665	4.8560

Supplementary Figure 35 HPLC spectra of **4d**

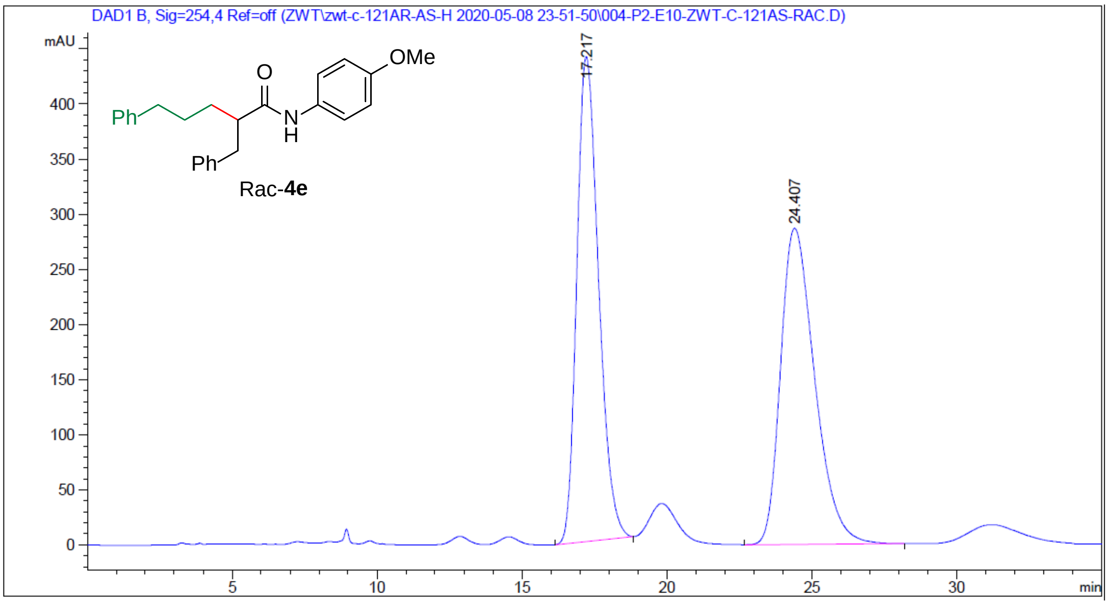

Peak #	RetTime [min]	Type	Width [min]	Area [mAU*s]	Height [mAU]	Area %
1	17.217	BB	0.8050	2.29494e4	439.63116	49.4405
2	24.407	BB	1.2552	2.34688e4	286.65881	50.5595

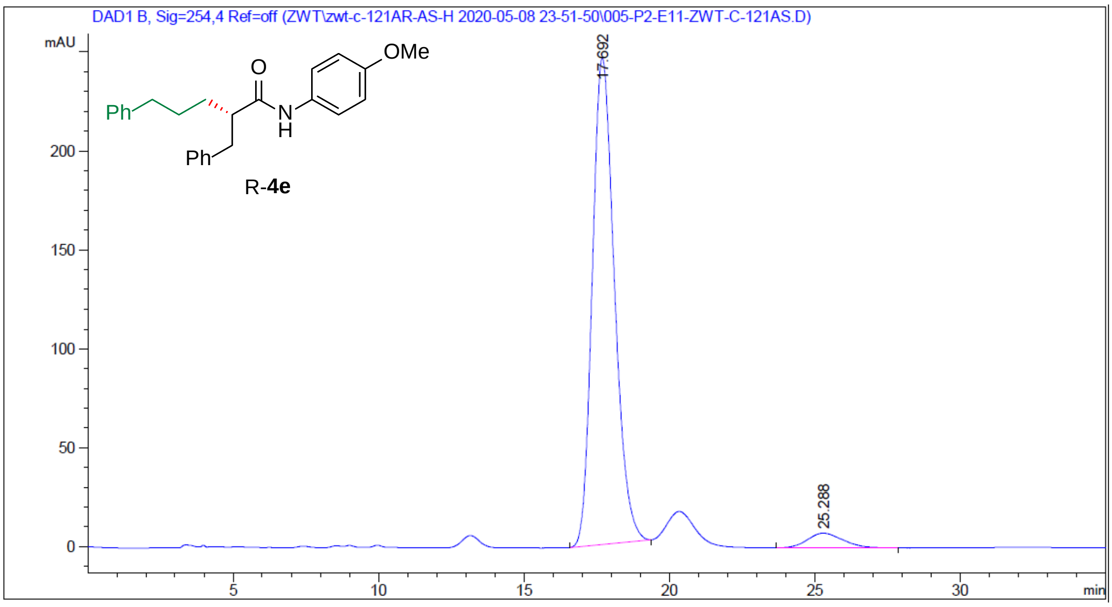

Peak #	RetTime [min]	Type	Width [min]	Area [mAU*s]	Height [mAU]	Area %
1	17.692	BB	0.8135	1.29689e4	245.81795	95.4075
2	25.288	BB	1.0390	624.26093	7.33459	4.5925

Supplementary Figure 36 HPLC spectra of **4e**

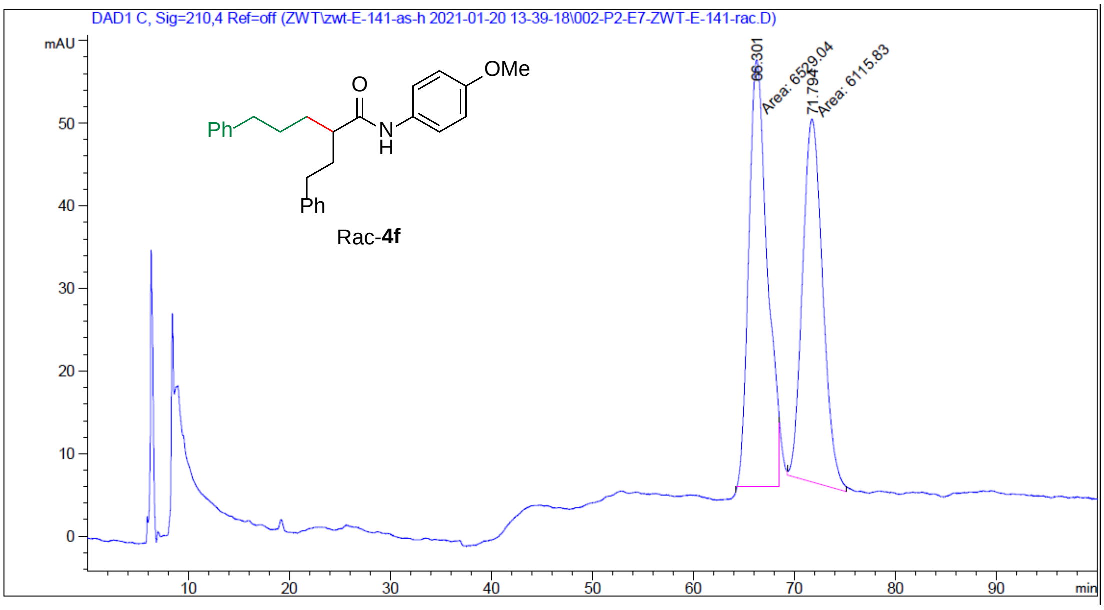

Peak #	RetTime [min]	Type	Width [min]	Area [mAU*s]	Height [mAU]	Area %
1	66.301	MM	2.1106	6529.04102	51.55787	51.6339
2	71.794	MM	2.3221	6115.83447	43.89596	48.3661

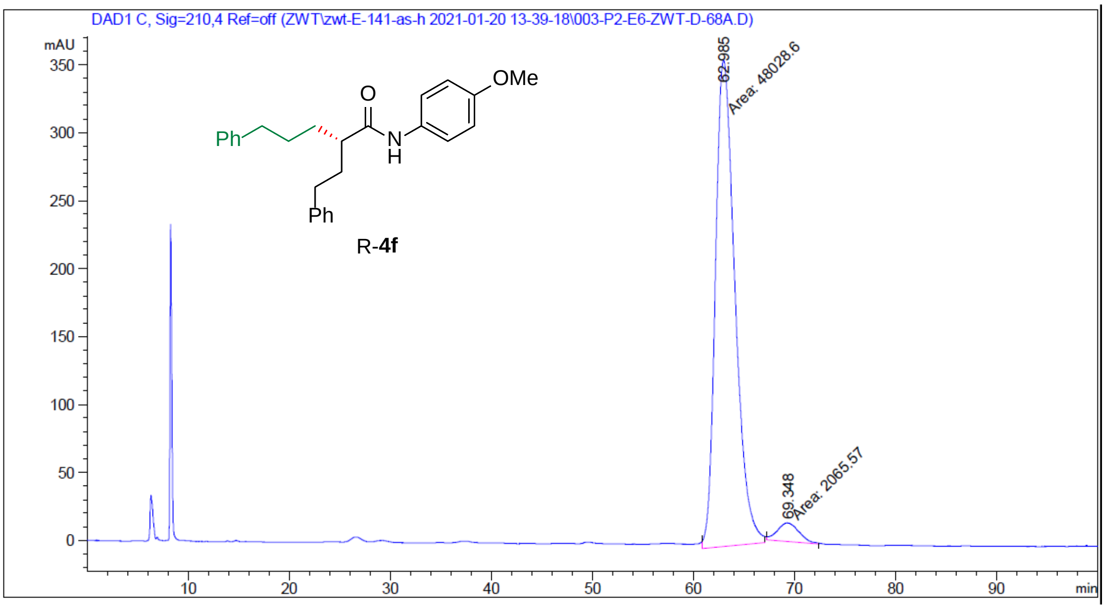

Peak #	RetTime [min]	Type	Width [min]	Area [mAU*s]	Height [mAU]	Area %
1	62.985	MM	2.2385	4.80286e4	357.59570	95.8766
2	69.348	MM	2.5146	2065.56934	13.69066	4.1234

Supplementary Figure 37 HPLC spectra of **4f**

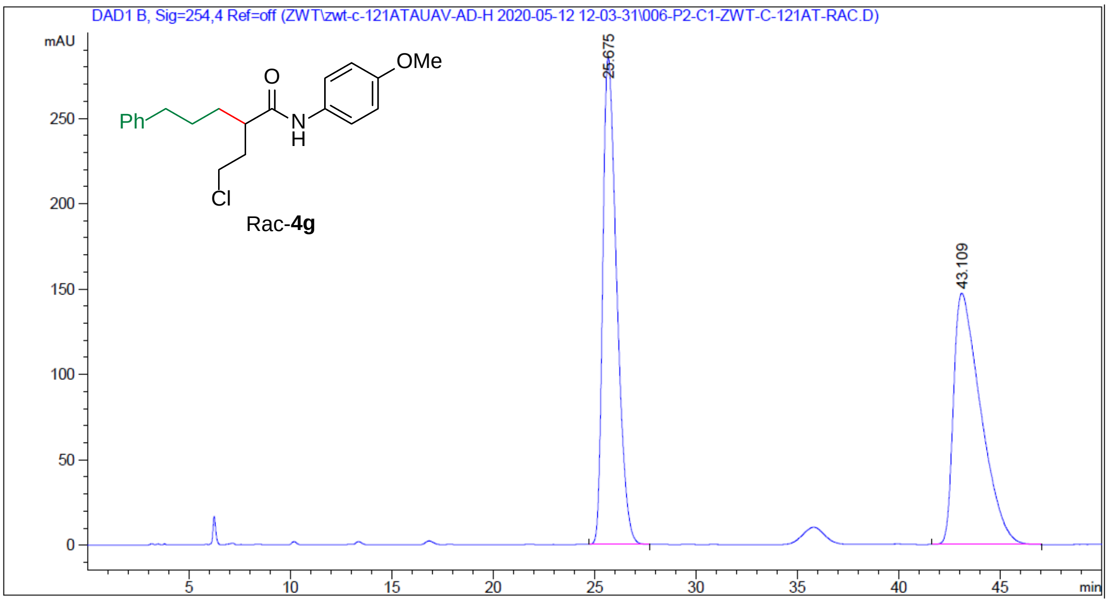

Peak #	RetTime [min]	Type	Width [min]	Area [mAU*s]	Height [mAU]	Area %
1	25.675	BB	0.7243	1.36557e4	285.35257	49.8710
2	43.109	BB	1.3155	1.37263e4	147.24985	50.1290

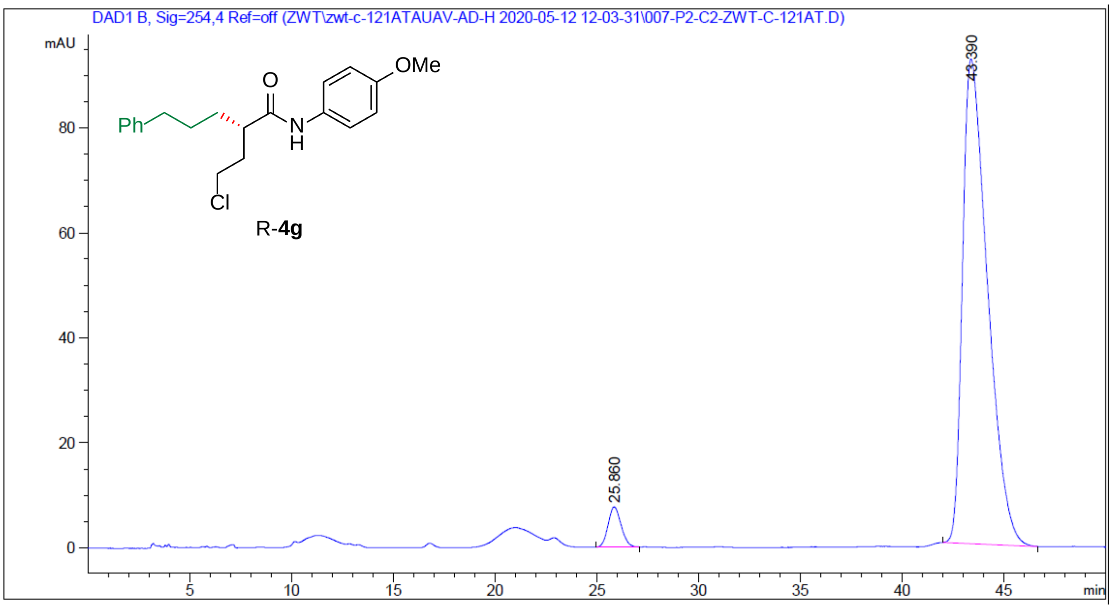

Peak #	RetTime [min]	Type	Width [min]	Area [mAU*s]	Height [mAU]	Area %
1	25.860	BB	0.6299	339.17471	7.64063	4.1333
2	43.390	BB	1.2685	7866.81299	92.28447	95.8667

Supplementary Figure 38 HPLC spectra of **4g**

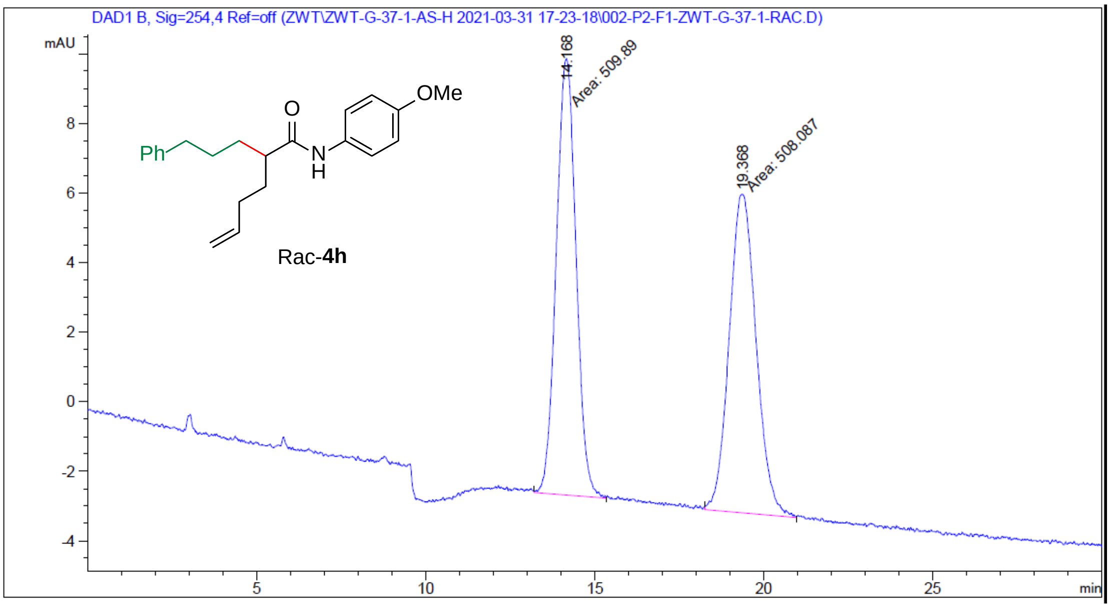

Peak #	RetTime [min]	Type	Width [min]	Area [mAU*s]	Height [mAU]	Area %
1	14.168	MM	0.6775	509.88965	12.54366	50.0885
2	19.368	MM	0.9244	508.08704	9.16091	49.9115

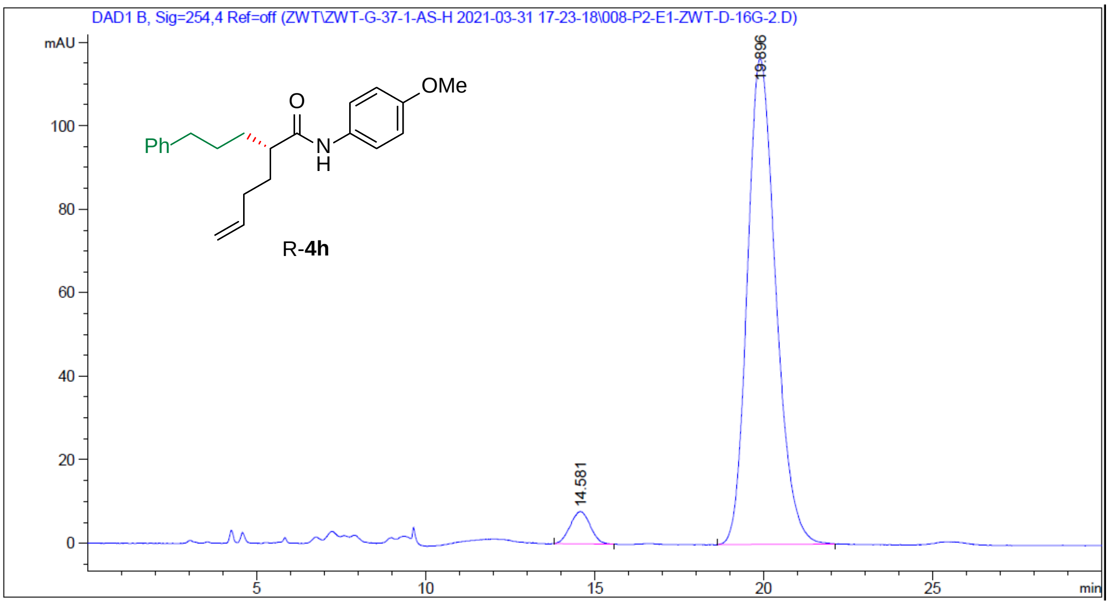

Peak #	RetTime [min]	Type	Width [min]	Area [mAU*s]	Height [mAU]	Area %
1	14.581	BB	0.5066	320.09772	7.77068	4.5005
2	19.896	BB	0.8649	6792.32959	116.38329	95.4995

Supplementary Figure 39 HPLC spectra of **4h**

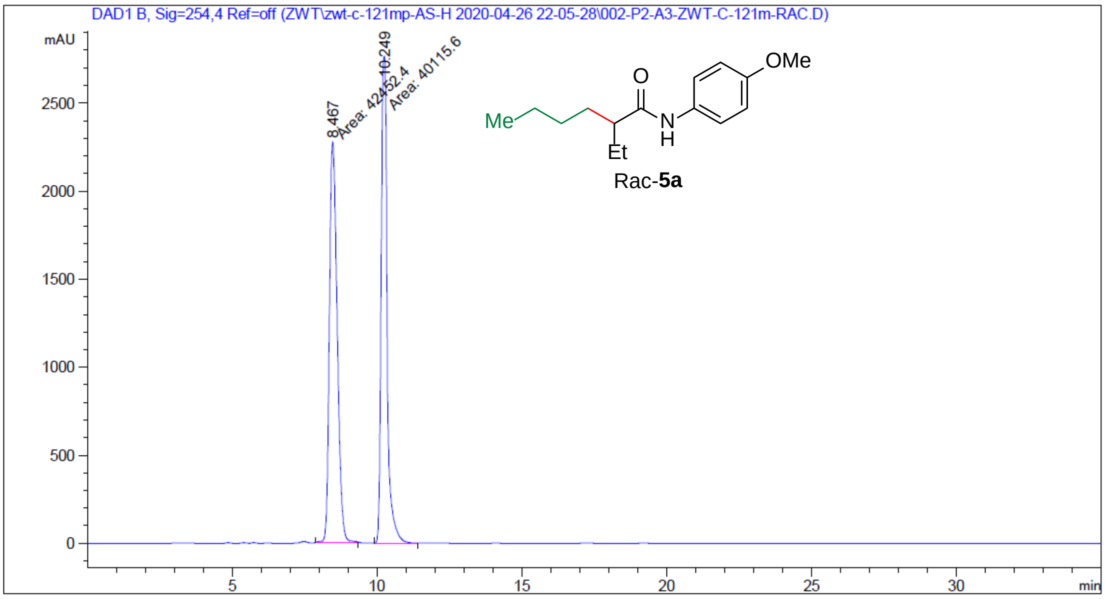

Peak #	RetTime [min]	Type	Width [min]	Area [mAU*s]	Height [mAU]	Area %
1	8.467	MM	0.3112	4.24524e4	2273.47925	51.4151
2	10.249	MM	0.2415	4.01156e4	2768.47314	48.5849

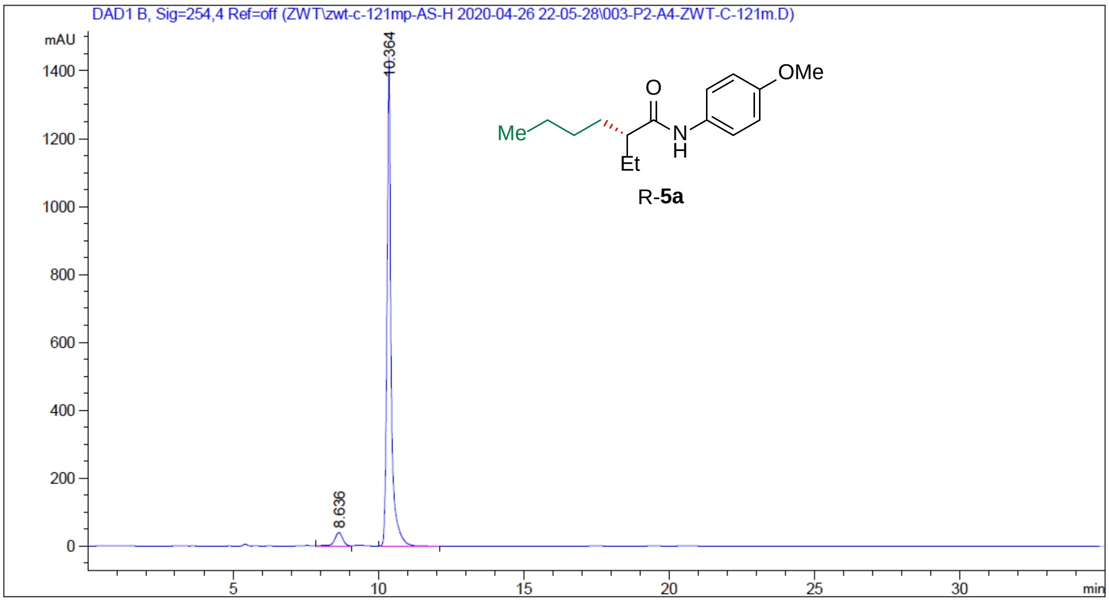

Peak #	RetTime [min]	Type	Width [min]	Area [mAU*s]	Height [mAU]	Area %
1	8.636	BB	0.2915	749.64093	39.49156	5.0261
2	10.364	BB	0.1404	1.41652e4	1444.70190	94.9739

Supplementary Figure 40 HPLC spectra of **5a**

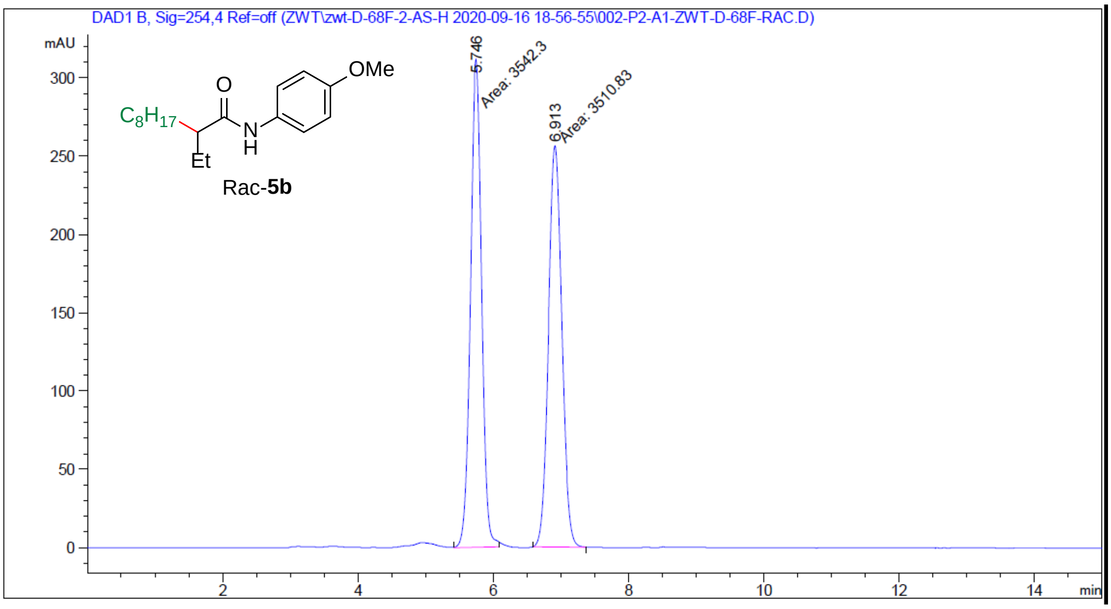

Peak #	RetTime [min]	Type	Width [min]	Area [mAU*s]	Height [mAU]	Area %
1	5.746	MM	0.1894	3542.29639	311.68204	50.2231
2	6.913	MM	0.2282	3510.83081	256.41693	49.7769

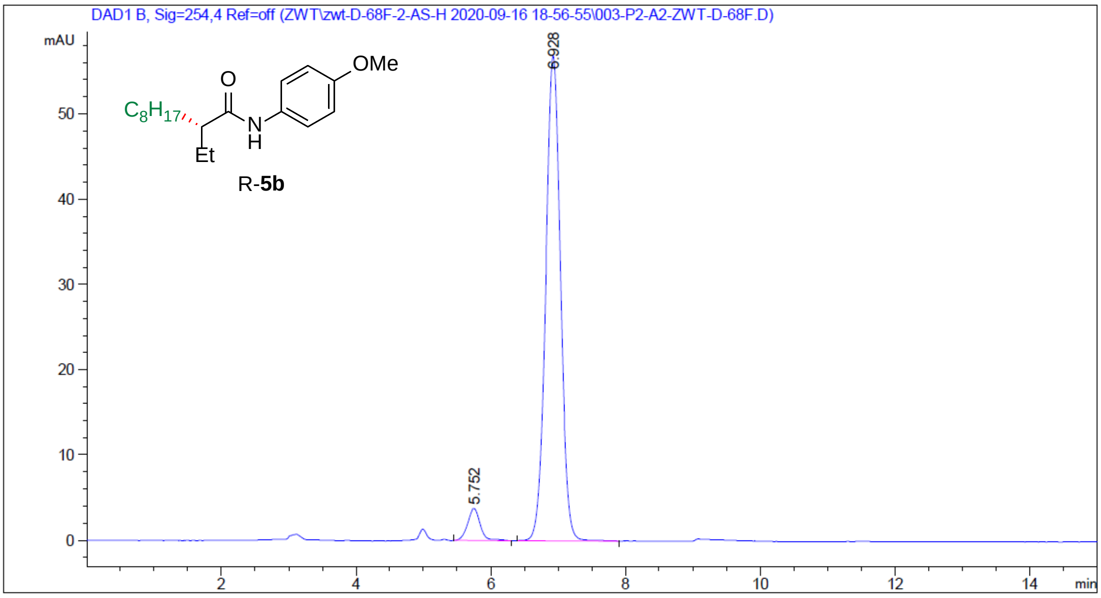

Peak #	RetTime [min]	Type	Width [min]	Area [mAU*s]	Height [mAU]	Area %
1	5.752	BB	0.1965	49.45812	3.78340	5.4154
2	6.928	BB	0.2352	863.82806	56.87895	94.5846

Supplementary Figure 41 HPLC spectra of **5b**

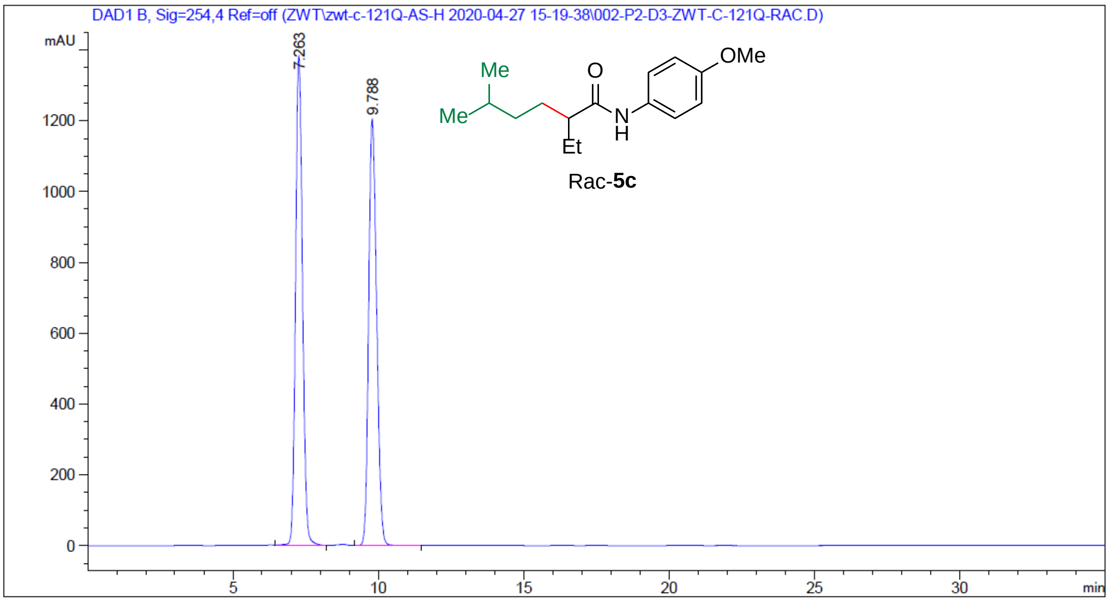

Peak #	RetTime [min]	Type	Width [min]	Area [mAU*s]	Height [mAU]	Area %
1	7.263	BB	0.2572	2.28692e4	1380.69189	49.9267
2	9.788	BB	0.2983	2.29364e4	1203.72302	50.0733

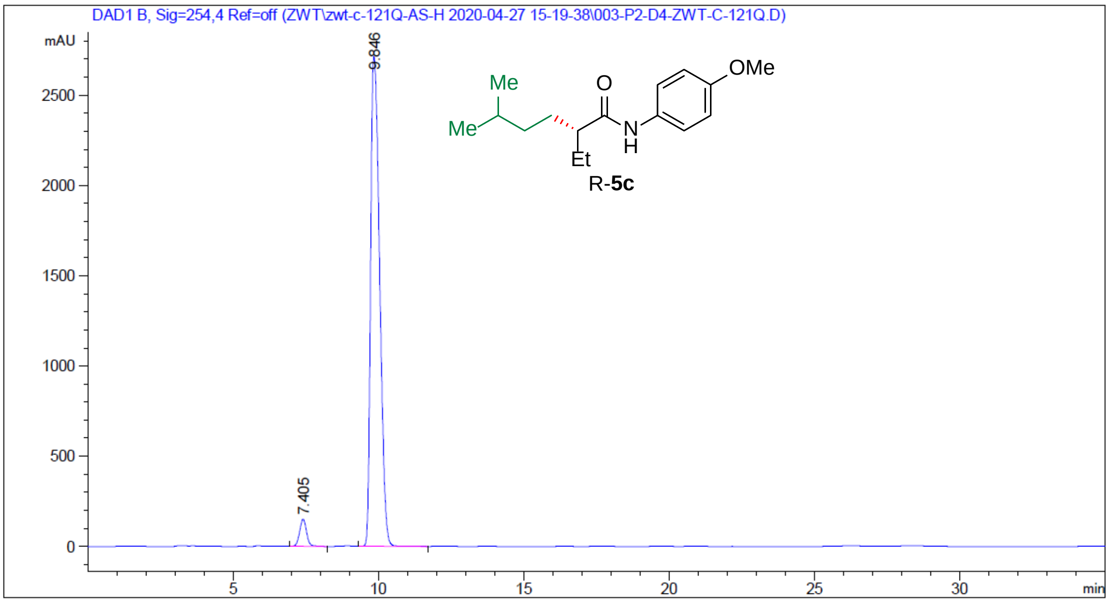

Peak #	RetTime [min]	Type	Width [min]	Area [mAU*s]	Height [mAU]	Area %
1	7.405	BB	0.2556	2473.60449	150.62645	4.0756
2	9.846	BB	0.3402	5.82198e4	2714.55762	95.9244

Supplementary Figure 42 HPLC spectra of **5c**

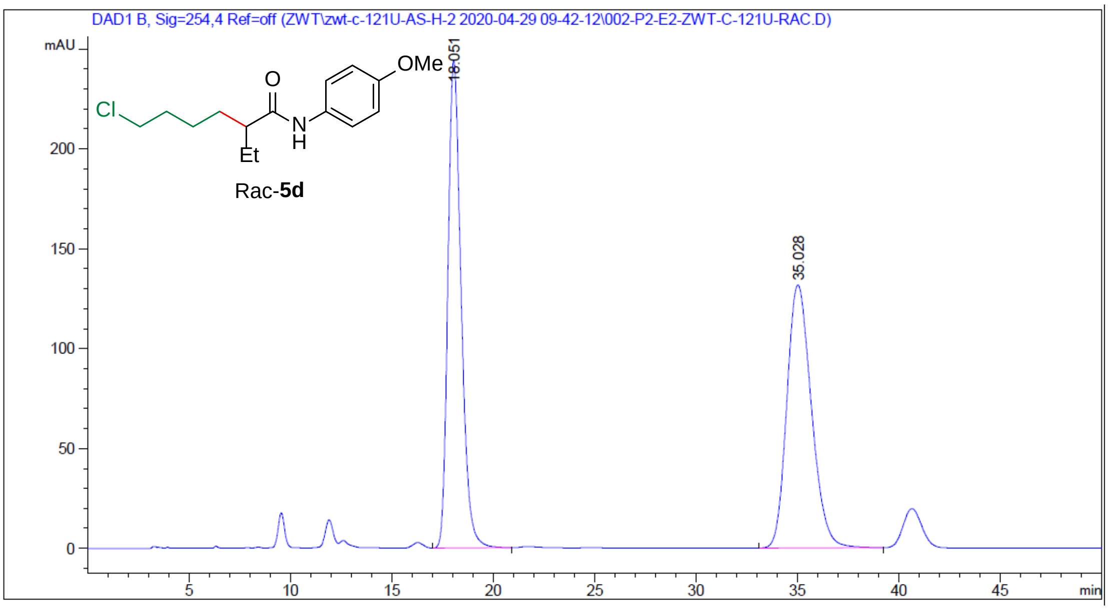

Peak #	RetTime [min]	Type	Width [min]	Area [mAU*s]	Height [mAU]	Area %
1	18.051	BB	0.6986	1.10549e4	243.98129	49.9243
2	35.028	BB	1.2864	1.10884e4	131.67757	50.0757

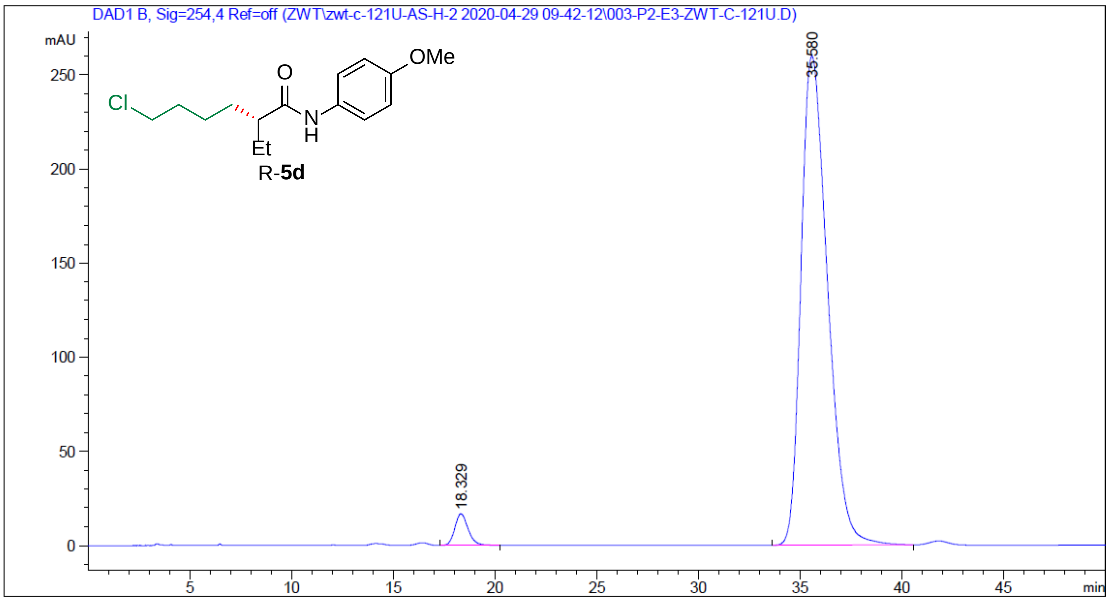

Peak #	RetTime [min]	Type	Width [min]	Area [mAU*s]	Height [mAU]	Area %
1	18.329	BB	0.6776	757.16620	16.67892	3.1189
2	35.580	BB	1.4044	2.35192e4	259.74652	96.8811

Supplementary Figure 43 HPLC spectra of **5d**

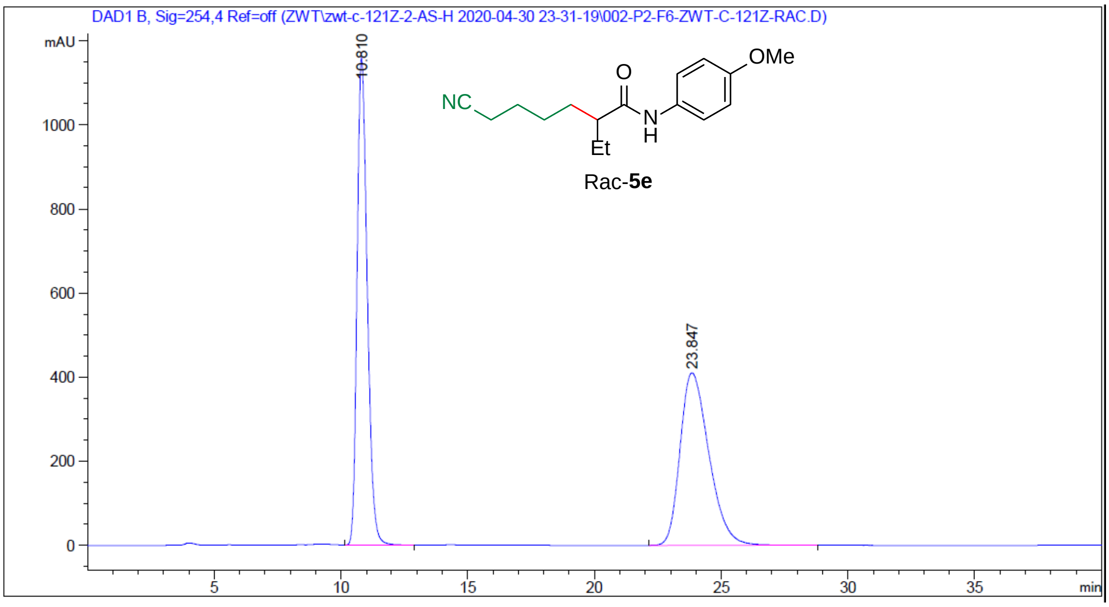

Peak #	RetTime [min]	Type	Width [min]	Area [mAU*s]	Height [mAU]	Area %
1	10.810	BB	0.4322	3.22042e4	1158.84705	49.8507
2	23.847	BB	1.2238	3.23972e4	410.03809	50.1493

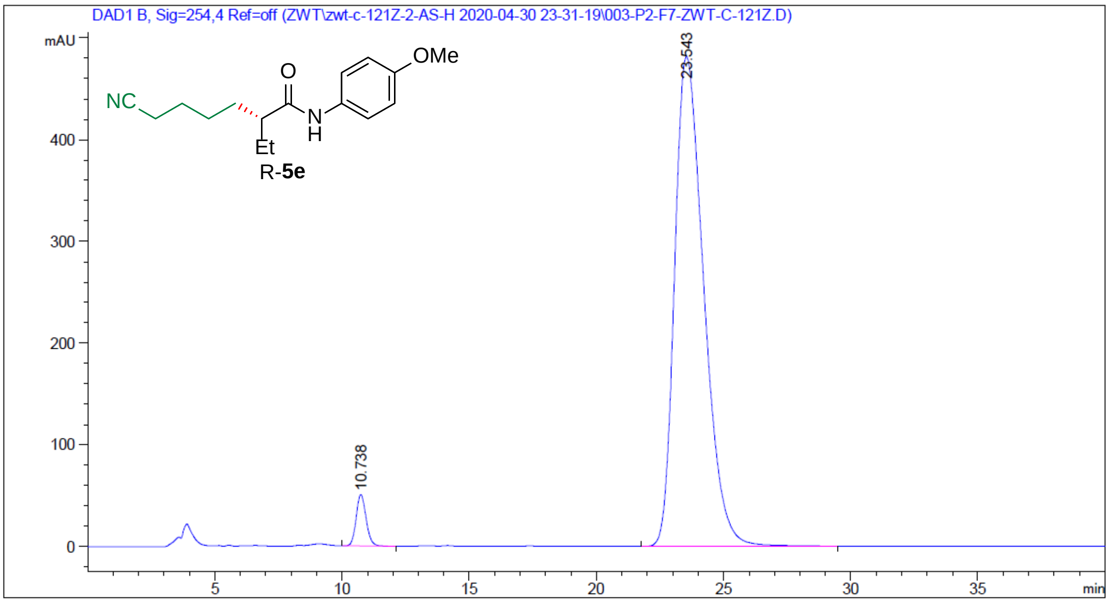

Peak #	RetTime [min]	Type	Width [min]	Area [mAU*s]	Height [mAU]	Area %
1	10.738	BB	0.4250	1382.81641	50.58066	3.4875
2	23.543	BB	1.2370	3.82673e4	481.60547	96.5125

Supplementary Figure 44 HPLC spectra of **5e**

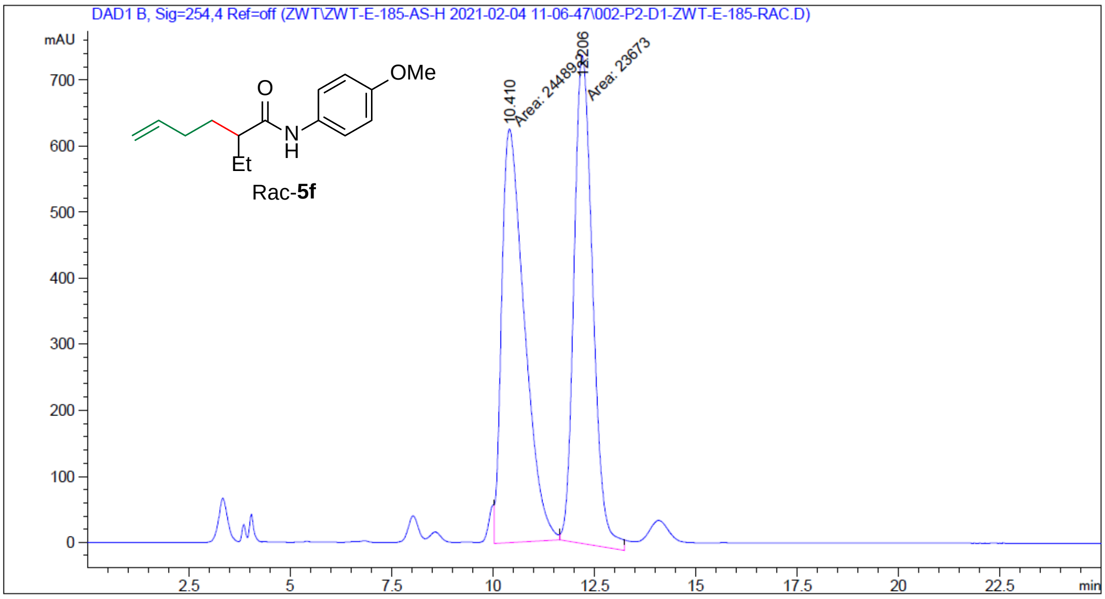

Peak #	RetTime [min]	Type	Width [min]	Area [mAU*s]	Height [mAU]	Area %
1	10.410	MM	0.6524	2.44892e4	625.66333	50.8473
2	12.206	MM	0.5345	2.36730e4	738.19446	49.1527

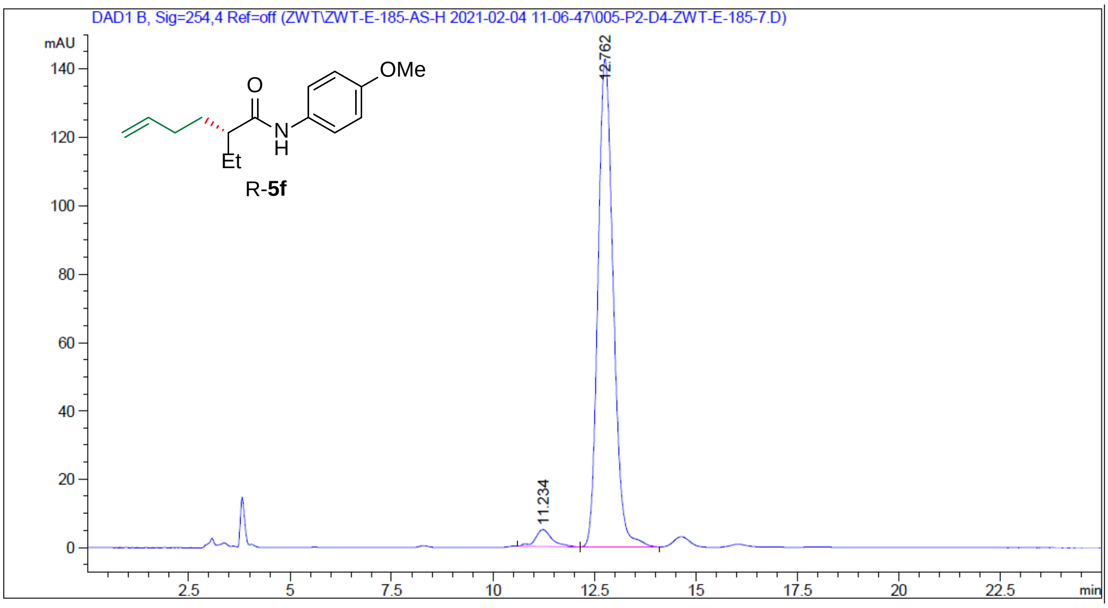

Peak #	RetTime [min]	Type	Width [min]	Area [mAU*s]	Height [mAU]	Area %
1	11.234	BB	0.4244	144.58733	4.95604	3.7158
2	12.762	BB	0.4063	3746.56958	142.72322	96.2842

Supplementary Figure 45 HPLC spectra of **5f**

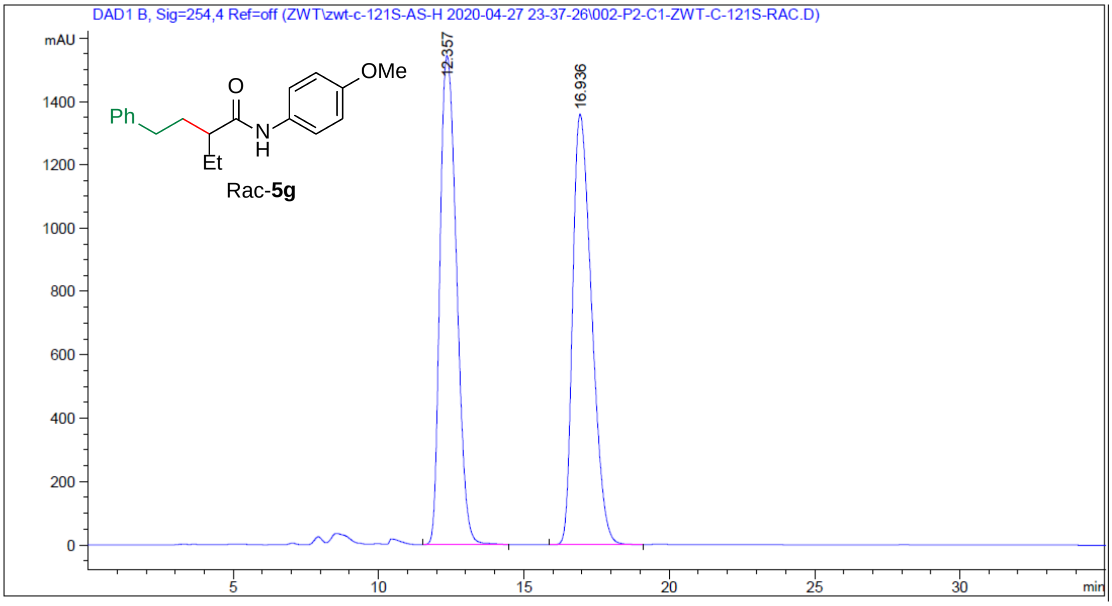

Peak #	RetTime [min]	Type	Width [min]	Area [mAU*s]	Height [mAU]	Area %
1	12.357	BB	0.6371	6.12395e4	1542.85974	49.8252
2	16.936	BB	0.7173	6.16692e4	1359.29773	50.1748

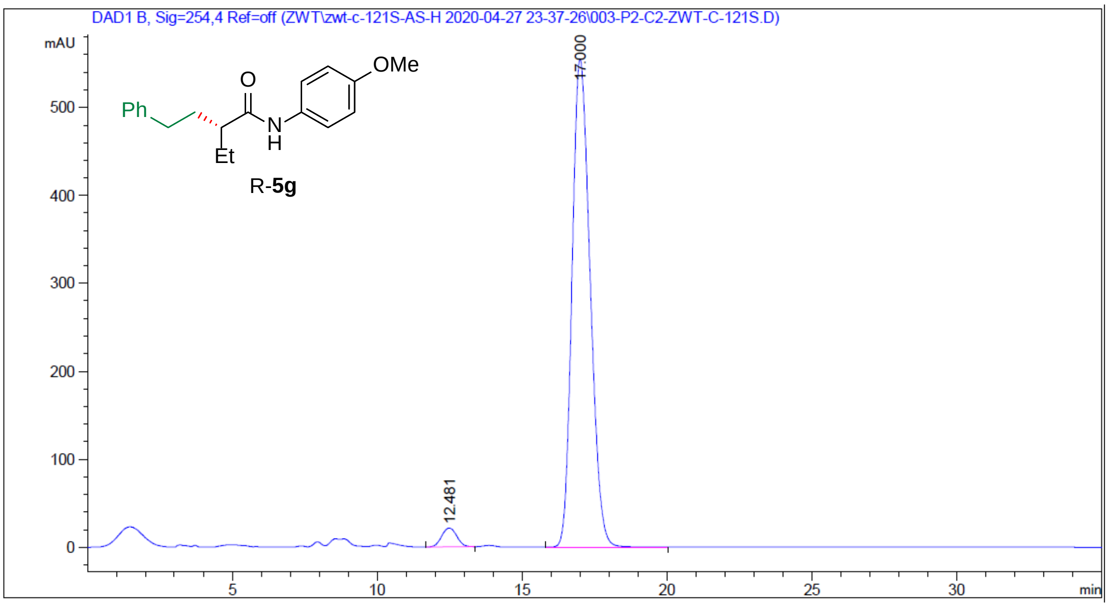

Peak #	RetTime [min]	Type	Width [min]	Area [mAU*s]	Height [mAU]	Area %
1	12.481	BB	0.5665	777.43707	21.36446	3.1354
2	17.000	BB	0.6793	2.40179e4	554.57019	96.8646

Supplementary Figure 46 HPLC spectra of **5g**

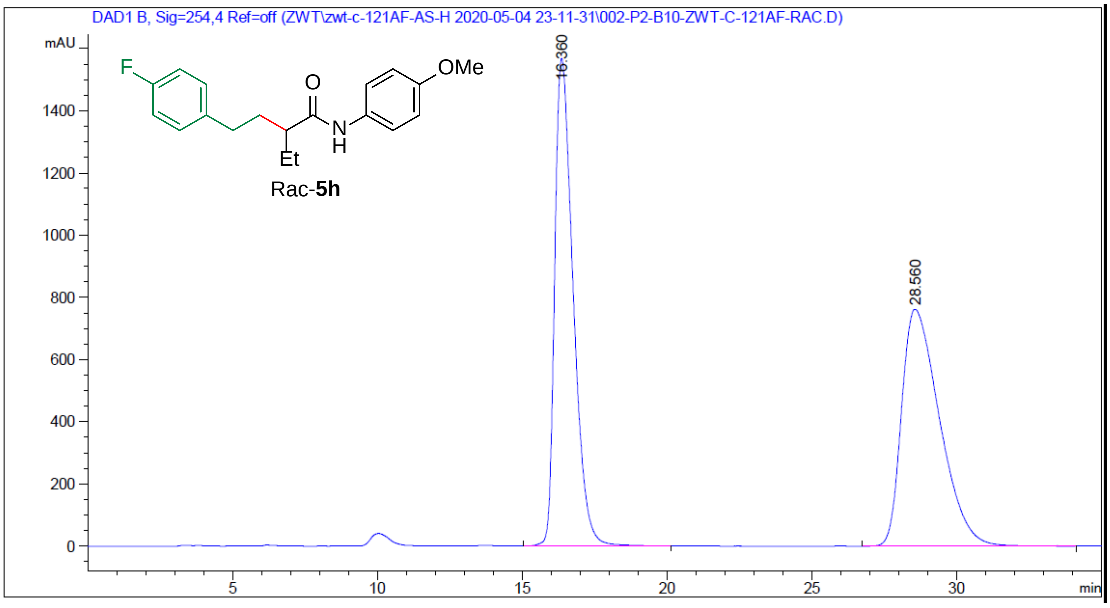

Peak #	RetTime [min]	Type	Width [min]	Area [mAU*s]	Height [mAU]	Area %
1	16.360	BB	0.6763	6.91407e4	1568.31030	49.9269
2	28.560	BB	1.3977	6.93430e4	761.95850	50.0731

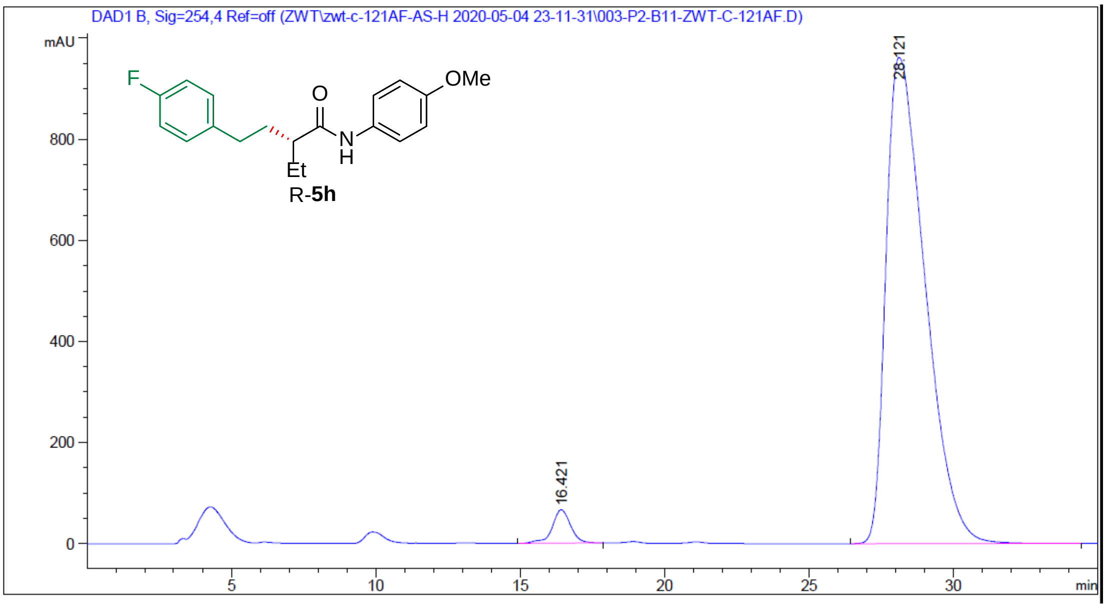

Peak #	RetTime [min]	Type	Width [min]	Area [mAU*s]	Height [mAU]	Area %
1	16.421	BB	0.6750	2949.95630	66.30722	3.1607
2	28.121	BB	1.3462	9.03824e4	961.89661	96.8393

Supplementary Figure 47 HPLC spectra of **5h**

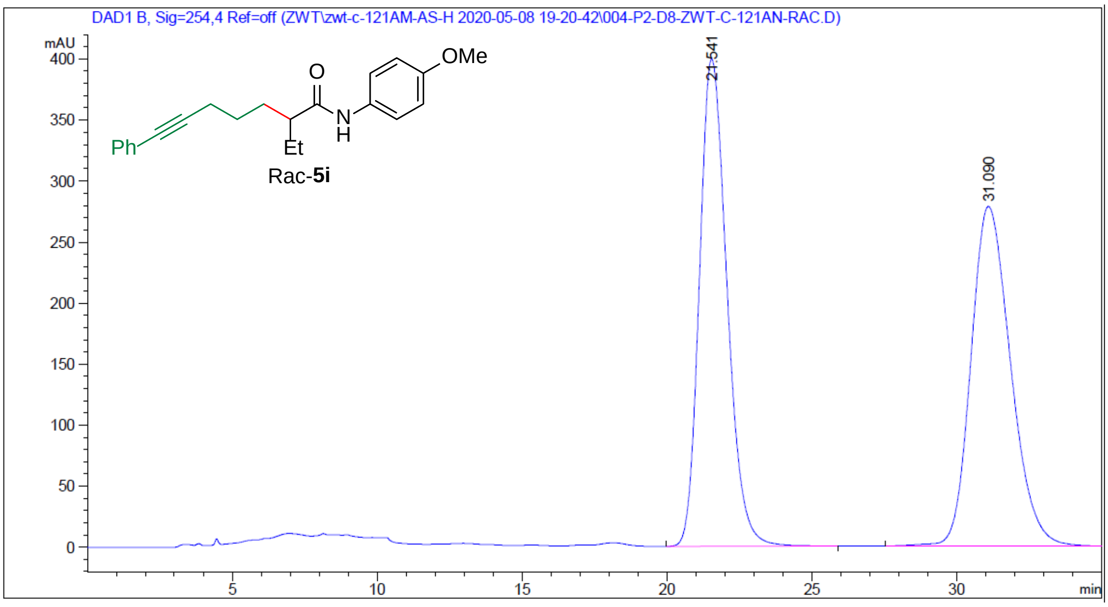

Peak #	RetTime [min]	Type	Width [min]	Area [mAU*s]	Height [mAU]	Area %
1	21.541	BB	1.0127	2.63175e4	399.43945	49.8653
2	31.090	BBA	1.4511	2.64597e4	278.34012	50.1347

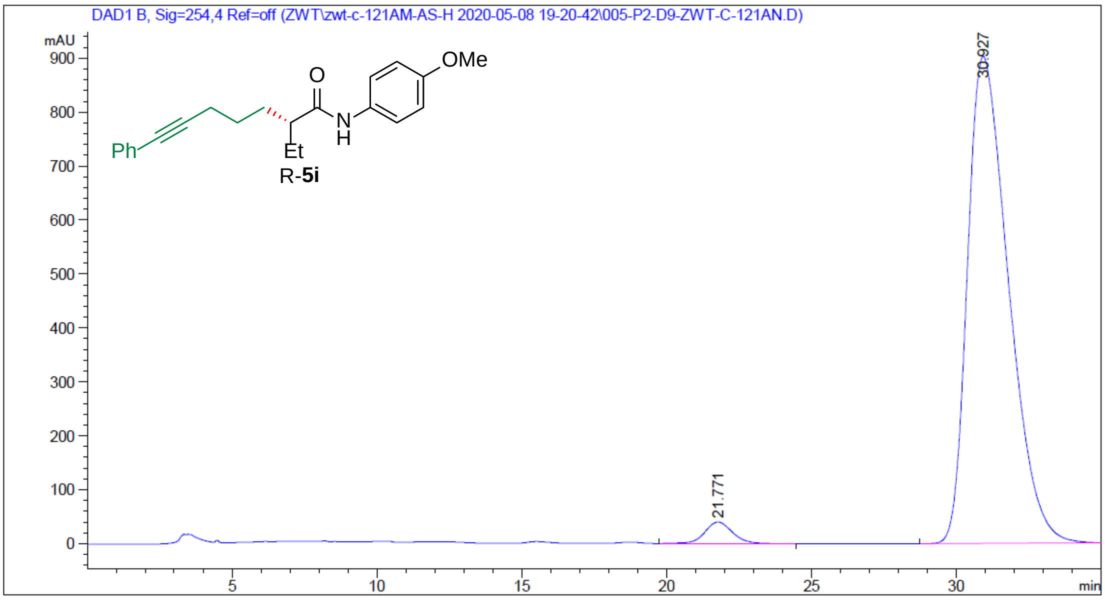

Peak #	RetTime [min]	Type	Width [min]	Area [mAU*s]	Height [mAU]	Area %
1	21.771	BB	0.9692	2660.69897	40.04501	2.9150
2	30.927	BBA	1.5186	8.86150e4	903.26758	97.0850

Supplementary Figure 48 HPLC spectra of **5i**

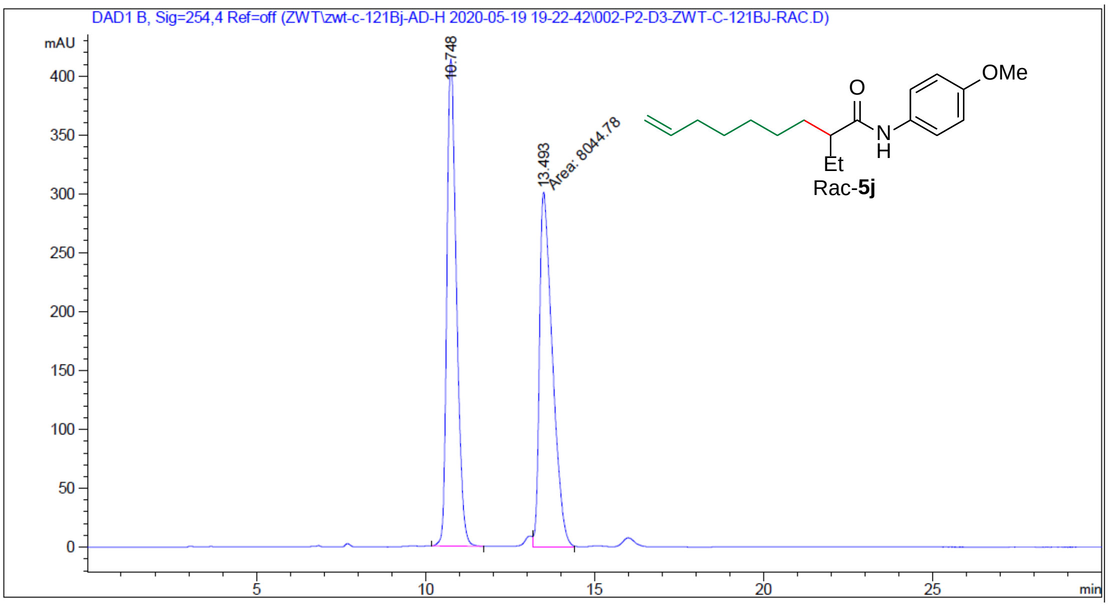

Peak #	RetTime [min]	Type	Width [min]	Area [mAU*s]	Height [mAU]	Area %
1	10.748	BB	0.3009	8109.42188	413.37579	50.2001
2	13.493	MM	0.4449	8044.77979	301.34198	49.7999

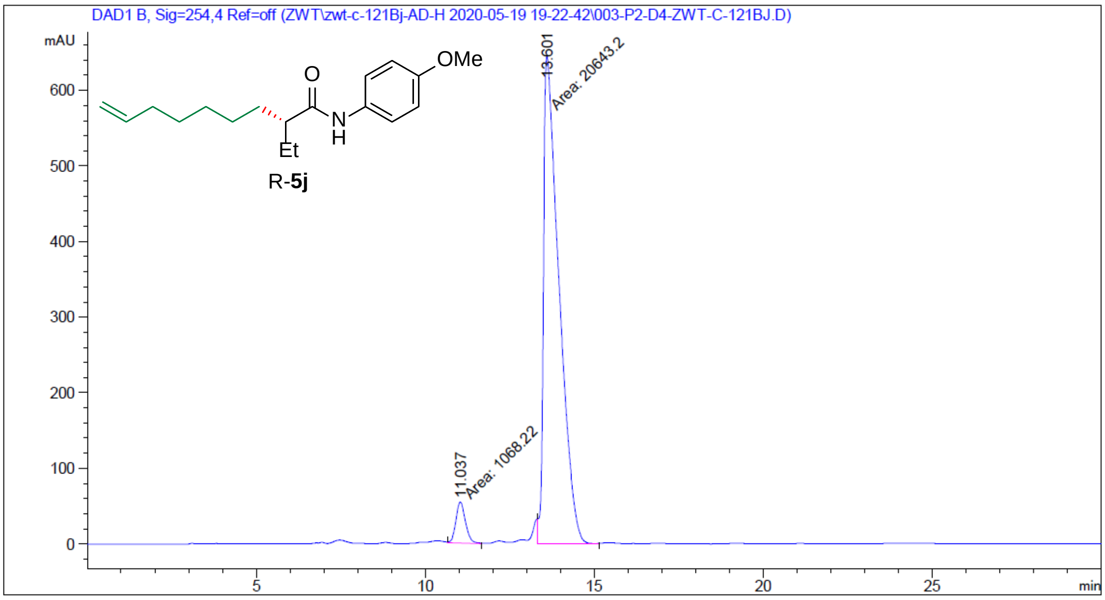

Peak #	RetTime [min]	Type	Width [min]	Area [mAU*s]	Height [mAU]	Area %
1	11.037	MM	0.3287	1068.22412	54.17225	4.9201
2	13.601	MM	0.5329	2.06432e4	645.58978	95.0799

Supplementary Figure 49 HPLC spectra of **5j**

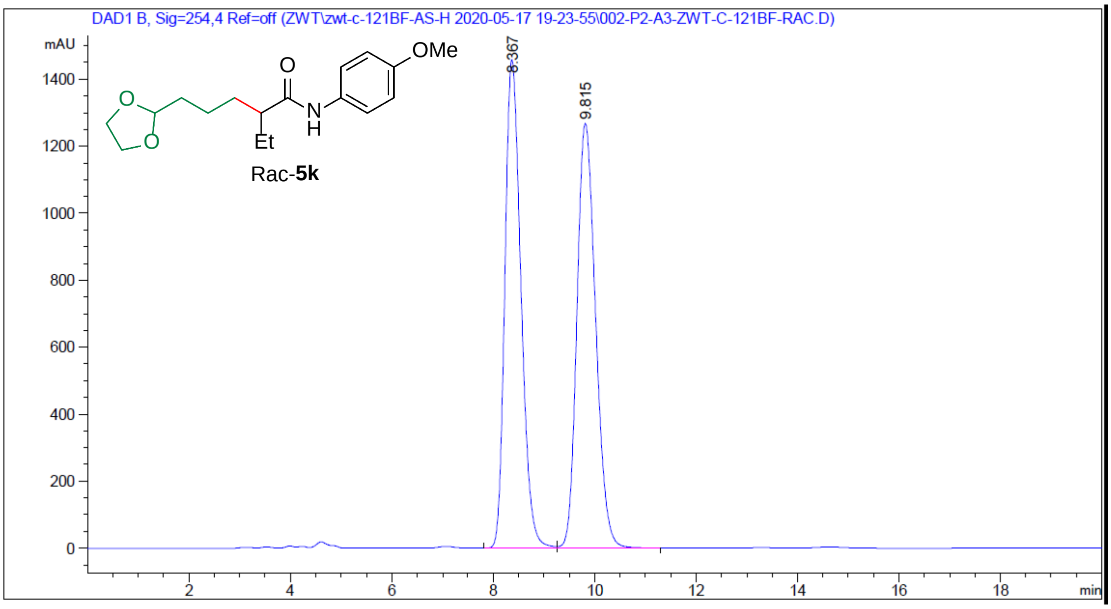

Peak #	RetTime [min]	Type	Width [min]	Area [mAU*s]	Height [mAU]	Area %
1	8.367	BV	0.3421	3.19945e4	1457.66394	49.6871
2	9.815	VB	0.4002	3.23974e4	1267.55493	50.3129

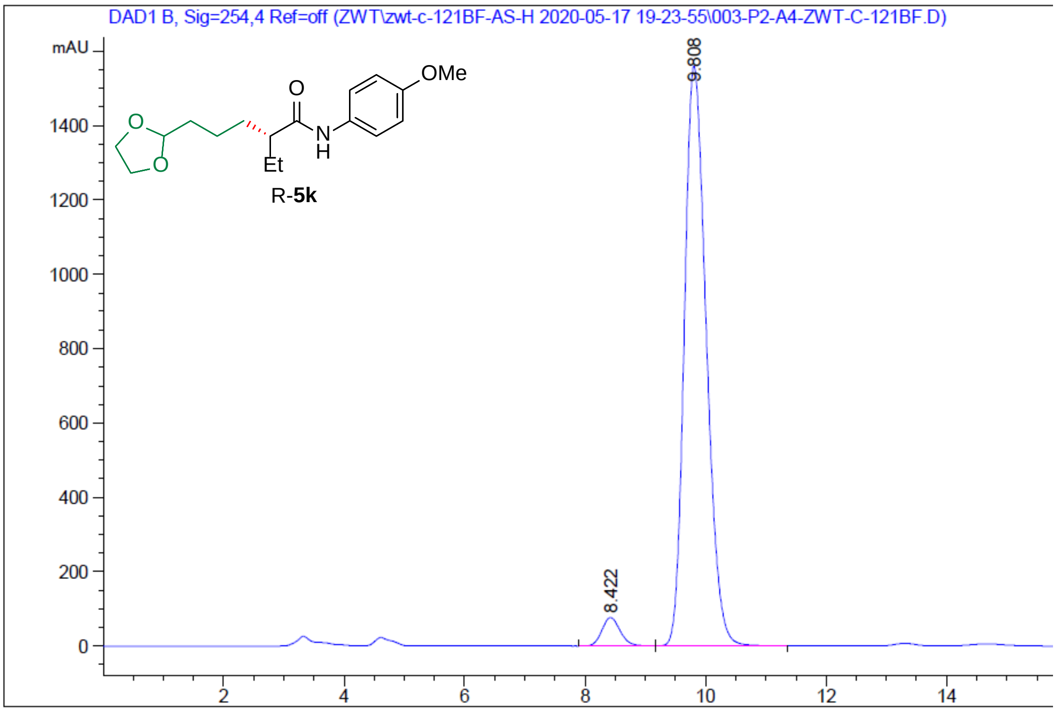

Peak #	RetTime [min]	Type	Width [min]	Area [mAU*s]	Height [mAU]	Area %
1	8.422	BB	0.3238	1594.39673	76.29082	3.9066
2	9.808	BB	0.3913	3.92183e4	1559.99512	96.0934

Supplementary Figure 50 HPLC spectra of **5k**

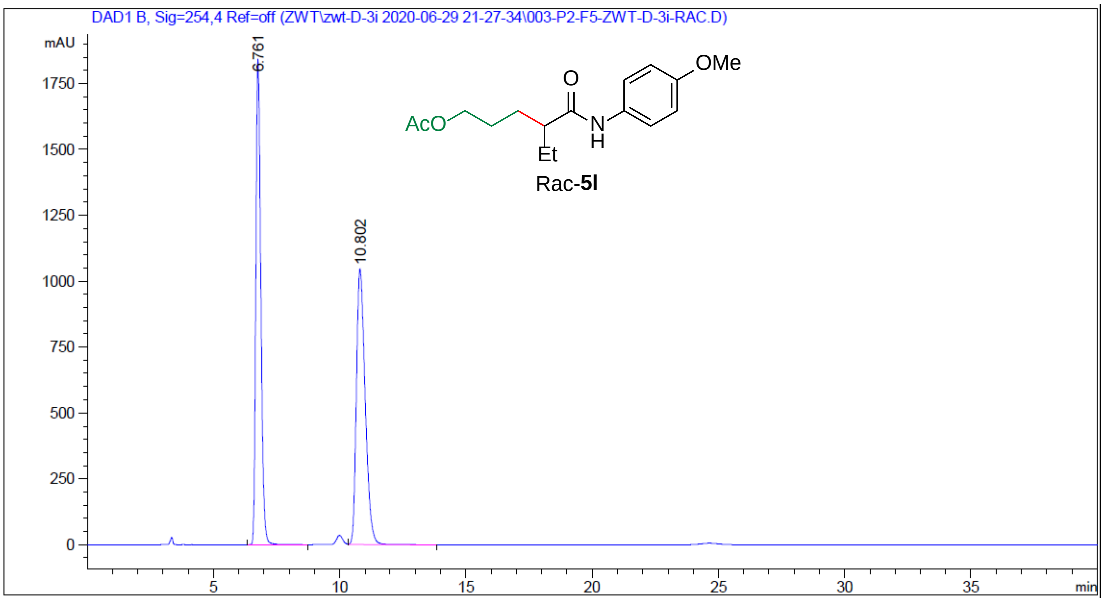

Peak #	RetTime [min]	Type	Width [min]	Area [mAU*s]	Height [mAU]	Area %
1	6.761	BB	0.2119	2.52621e4	1843.46655	49.8500
2	10.802	VB	0.3752	2.54141e4	1046.96155	50.1500

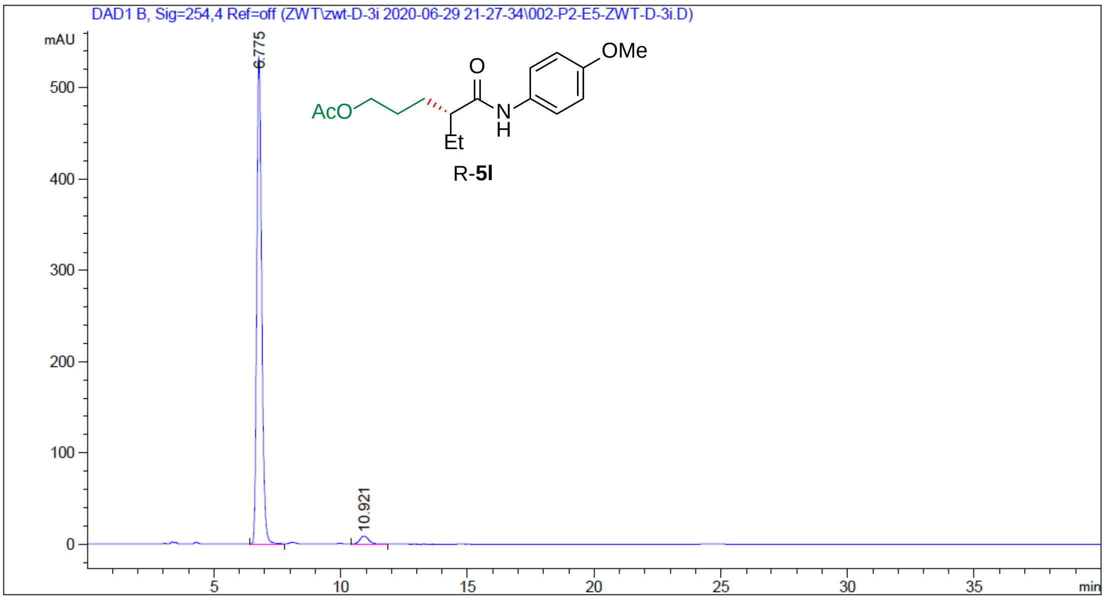

Peak #	RetTime [min]	Type	Width [min]	Area [mAU*s]	Height [mAU]	Area %
1	6.775	BB	0.2099	7245.11084	535.24762	97.0628
2	10.921	BB	0.3763	219.24254	8.99572	2.9372

Supplementary Figure 51 HPLC spectra of **5l**

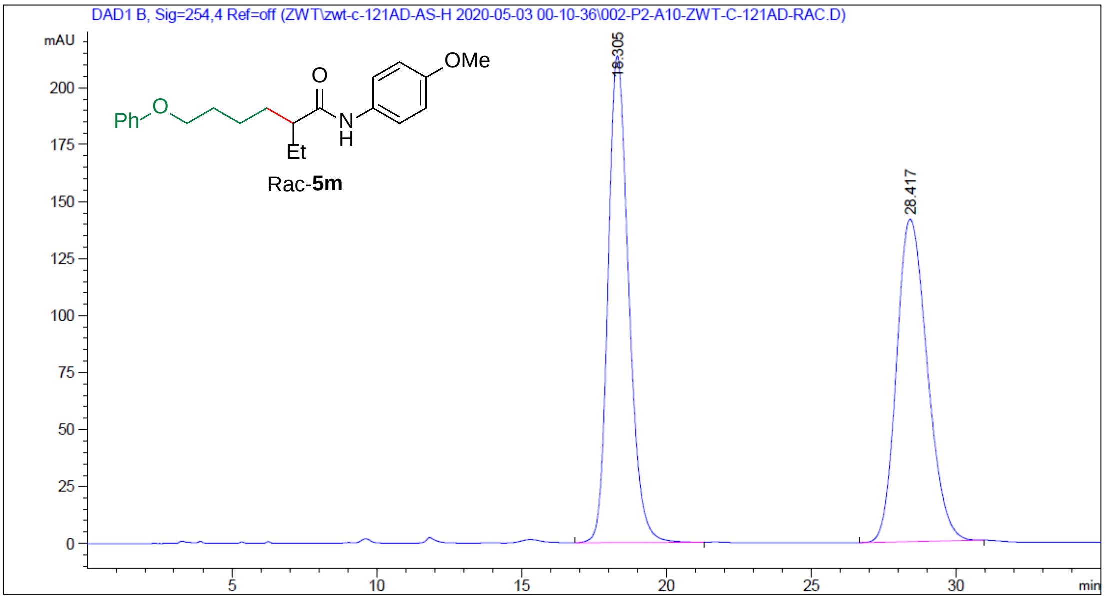

Peak #	RetTime [min]	Type	Width [min]	Area [mAU*s]	Height [mAU]	Area %
1	18.305	BB	0.7660	1.05782e4	213.50287	50.3403
2	28.417	BB	1.1369	1.04351e4	141.40991	49.6597

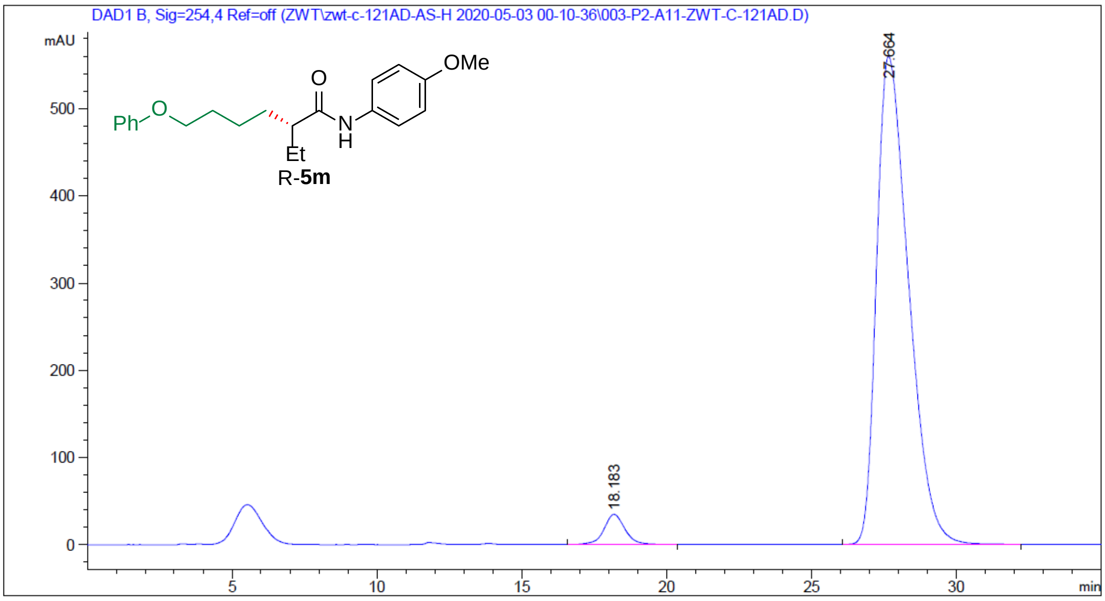

Peak #	RetTime [min]	Type	Width [min]	Area [mAU*s]	Height [mAU]	Area %
1	18.183	BB	0.7665	1734.78430	34.51064	3.8931
2	27.664	BB	1.1731	4.28251e4	559.42096	96.1069

Supplementary Figure 52 HPLC spectra of **5m**

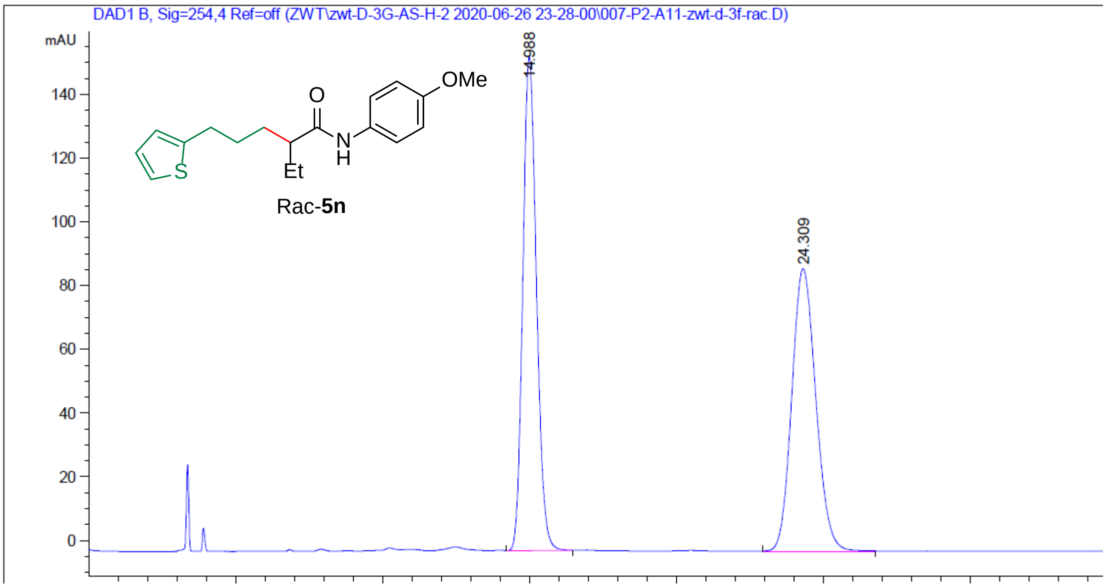

Peak #	RetTime [min]	Type	Width [min]	Area [mAU*s]	Height [mAU]	Area %
1	14.988	BB	0.4999	4995.59131	155.05029	49.8632
2	24.309	BB	0.8773	5022.99463	88.62237	50.1368

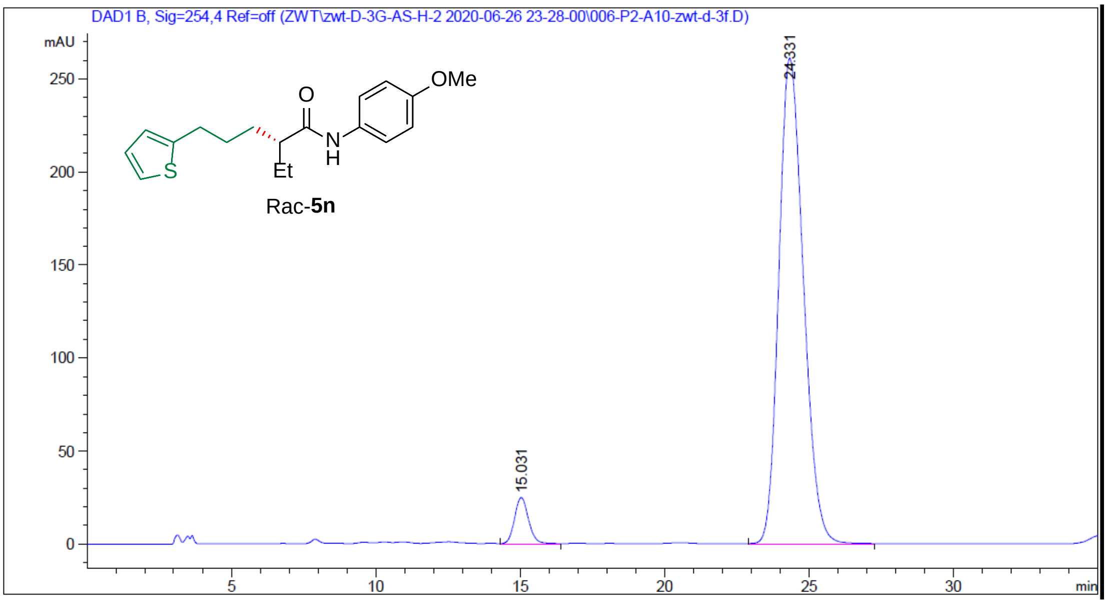

Peak #	RetTime [min]	Type	Width [min]	Area [mAU*s]	Height [mAU]	Area %
1	15.031	BB	0.5119	825.52588	24.82646	5.0983
2	24.331	BB	0.9070	1.53665e4	260.96042	94.9017

Supplementary Figure 53 HPLC spectra of **5n**

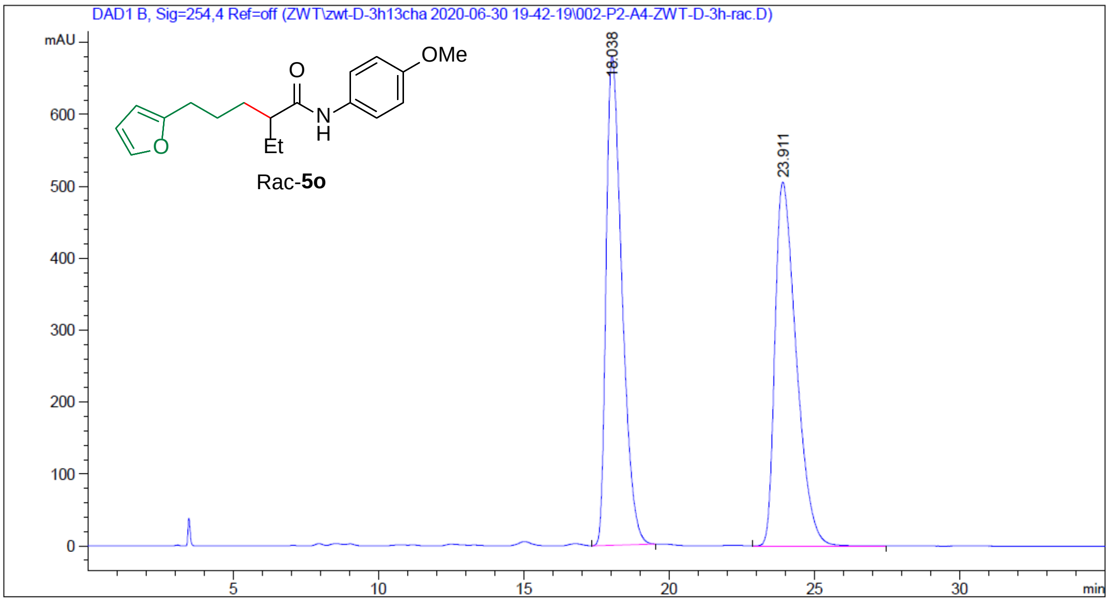

Peak #	RetTime [min]	Type	Width [min]	Area [mAU*s]	Height [mAU]	Area %
1	18.038	BB	0.5761	2.56508e4	680.01367	49.9250
2	23.911	BB	0.7774	2.57279e4	505.84158	50.0750

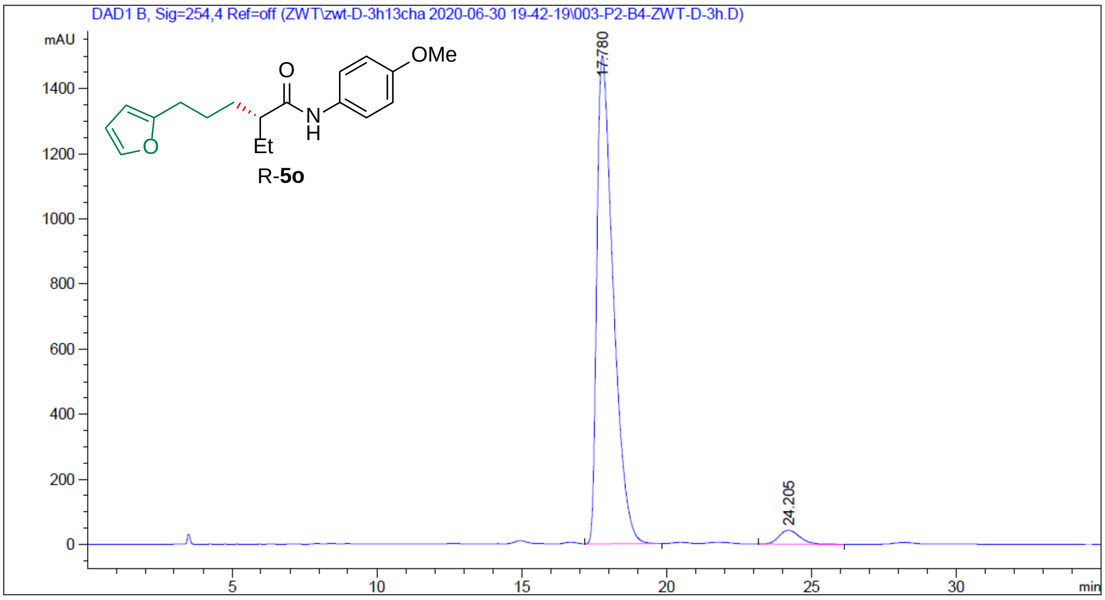

Peak #	RetTime [min]	Type	Width [min]	Area [mAU*s]	Height [mAU]	Area %
1	17.780	VB	0.5936	5.88001e4	1499.17969	96.4975
2	24.205	BB	0.7657	2134.25439	43.09987	3.5025

Supplementary Figure 54 HPLC spectra of **5o**

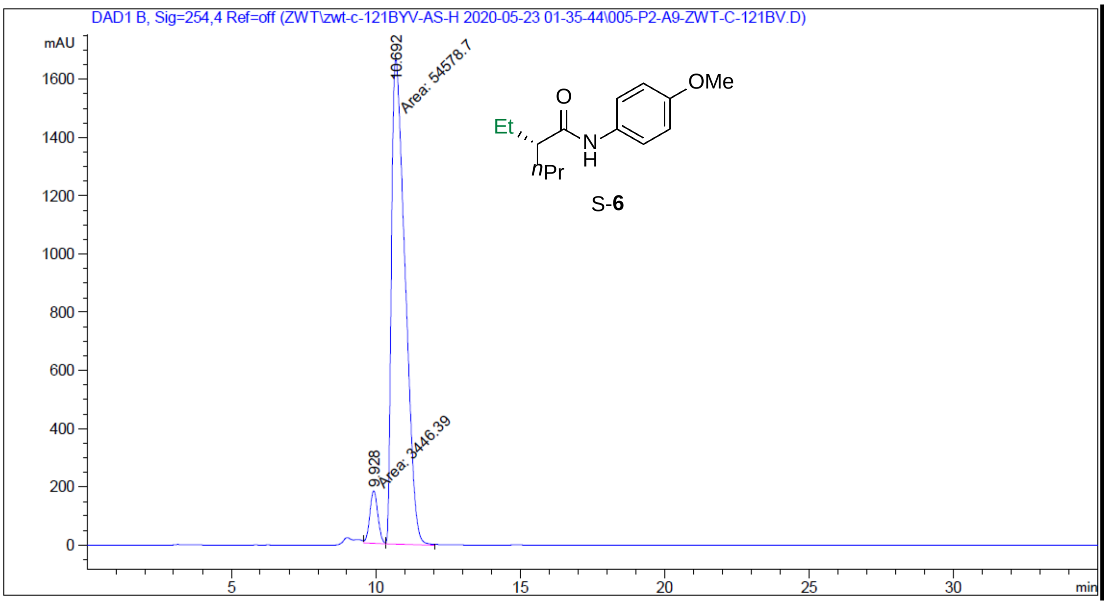

Peak #	RetTime [min]	Type	Width [min]	Area [mAU*s]	Height [mAU]	Area %
1	9.928	MM	0.3193	3446.39331	179.89151	5.9395
2	10.692	MM	0.5461	5.45787e4	1665.61707	94.0605

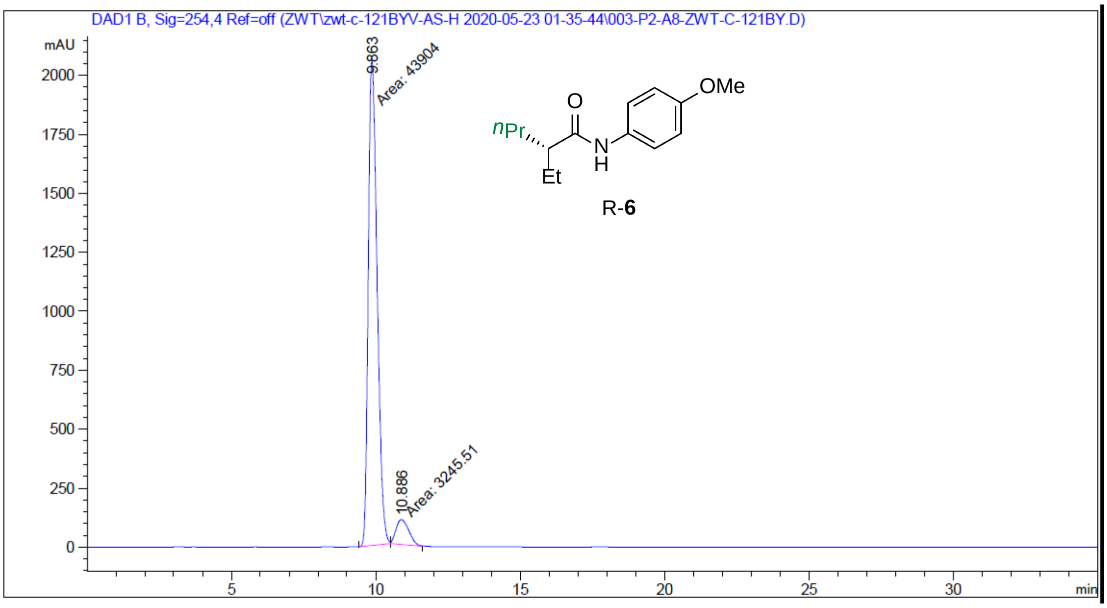

Peak #	RetTime [min]	Type	Width [min]	Area [mAU*s]	Height [mAU]	Area %
1	9.863	MM	0.3561	4.39040e4	2055.05566	93.1165
2	10.886	MM	0.5158	3245.51367	104.87955	6.8835

Supplementary Figure 55 HPLC spectra of **6**

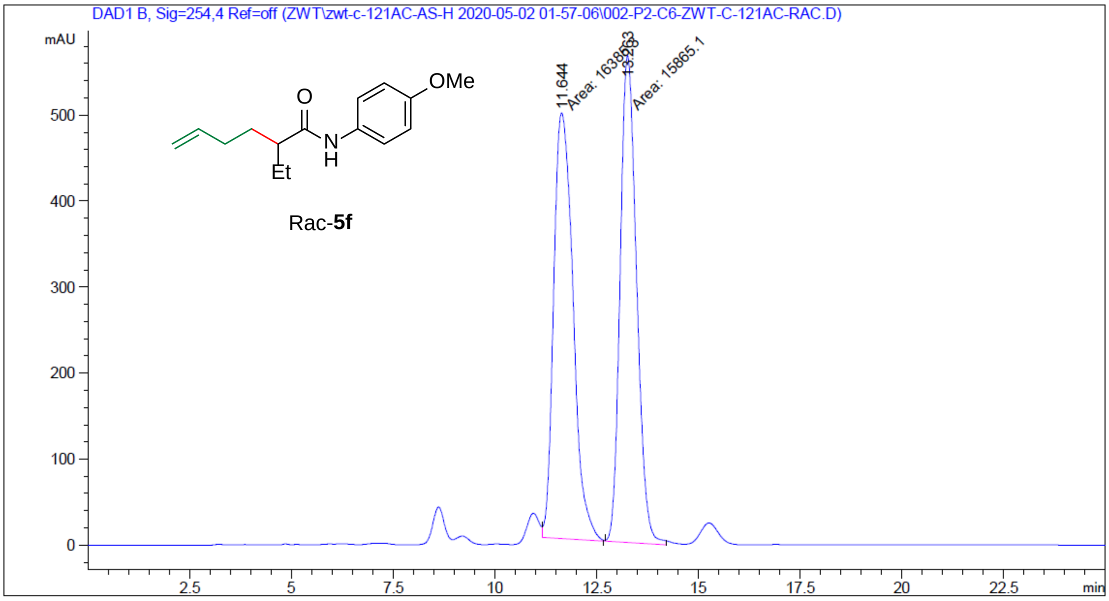

Peak #	RetTime [min]	Type	Width [min]	Area [mAU*s]	Height [mAU]	Area %
1	11.644	MM	0.5514	1.63853e4	495.26215	50.8065
2	13.263	MM	0.4671	1.58651e4	566.07434	49.1935

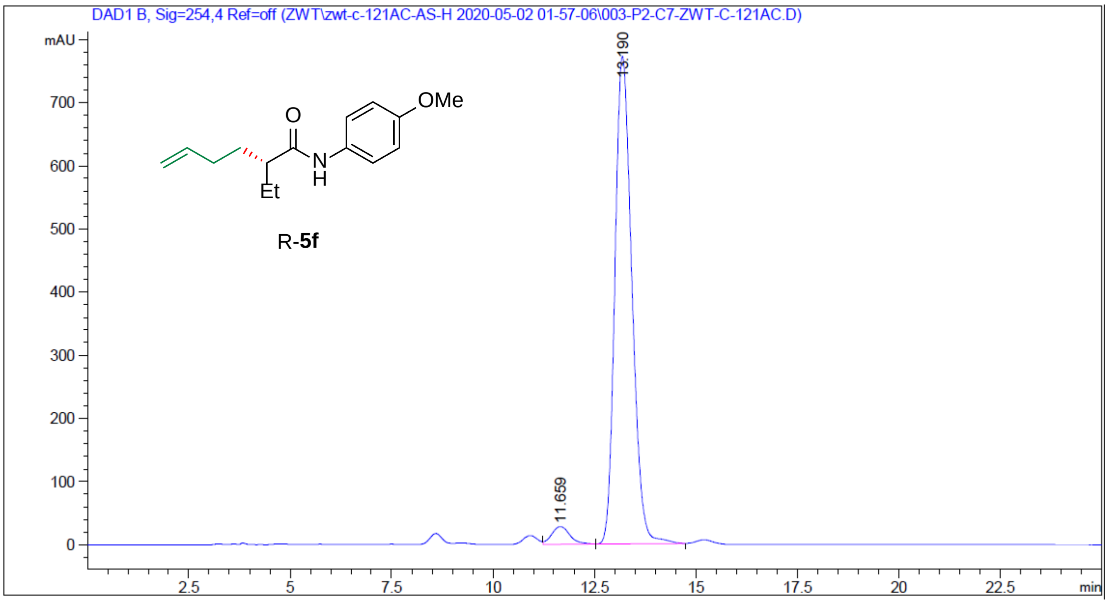

Peak #	RetTime [min]	Type	Width [min]	Area [mAU*s]	Height [mAU]	Area %
1	11.659	VB	0.4919	874.23456	27.72603	3.7519
2	13.190	BB	0.4510	2.24271e4	771.82605	96.2481

Supplementary Figure 56 HPLC spectra of **5f**

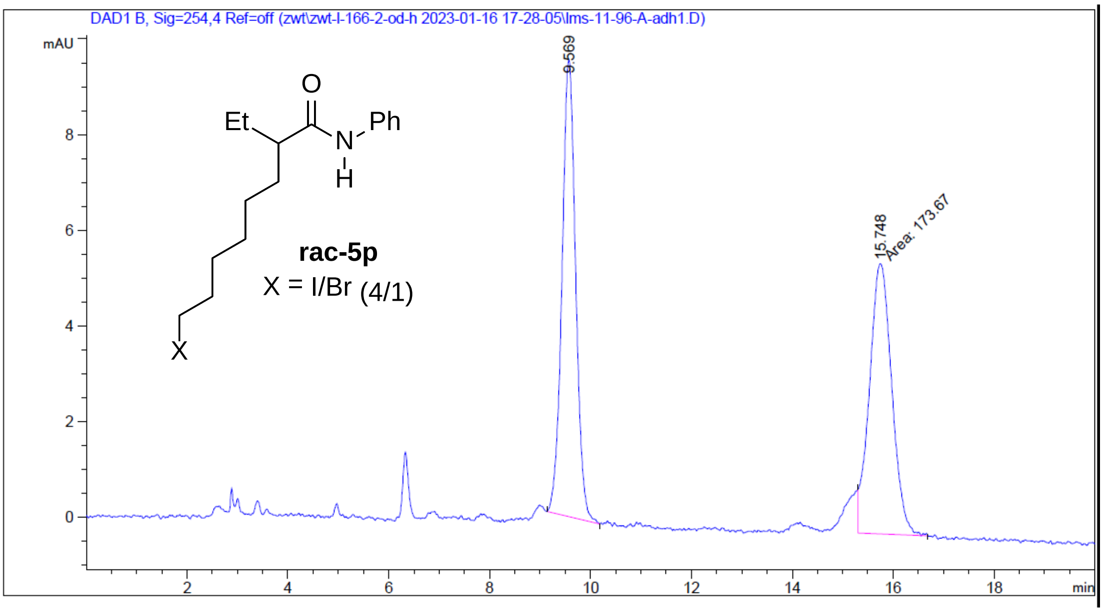

Peak #	RetTime [min]	Type	Width [min]	Area [mAU*s]	Height [mAU]	Area %
1	9.569	BB	0.2873	179.69246	9.55970	50.8522
2	15.748	FM	0.5123	173.66965	5.64992	49.1478

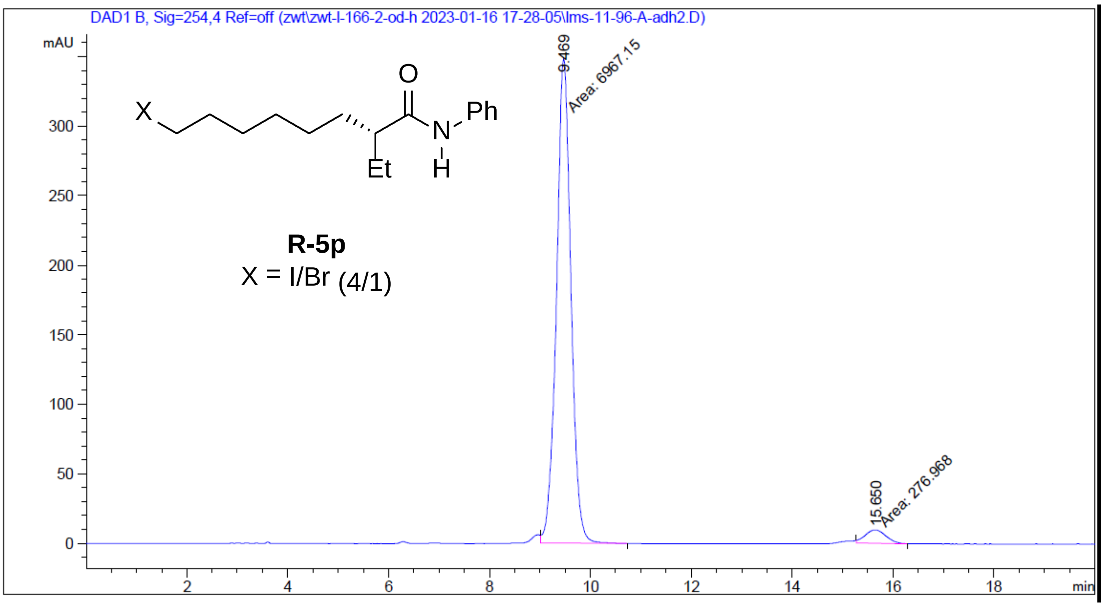

Peak #	RetTime [min]	Type	Width [min]	Area [mAU*s]	Height [mAU]	Area %
1	9.469	FM	0.3333	6967.15234	348.35828	96.1766
2	15.650	MM	0.4809	276.96832	9.59881	3.8234

Supplementary Figure 57 HPLC spectra of **5p**

2.7 ^{1}H-NMR and ^{13}C-NMR Spectra Data

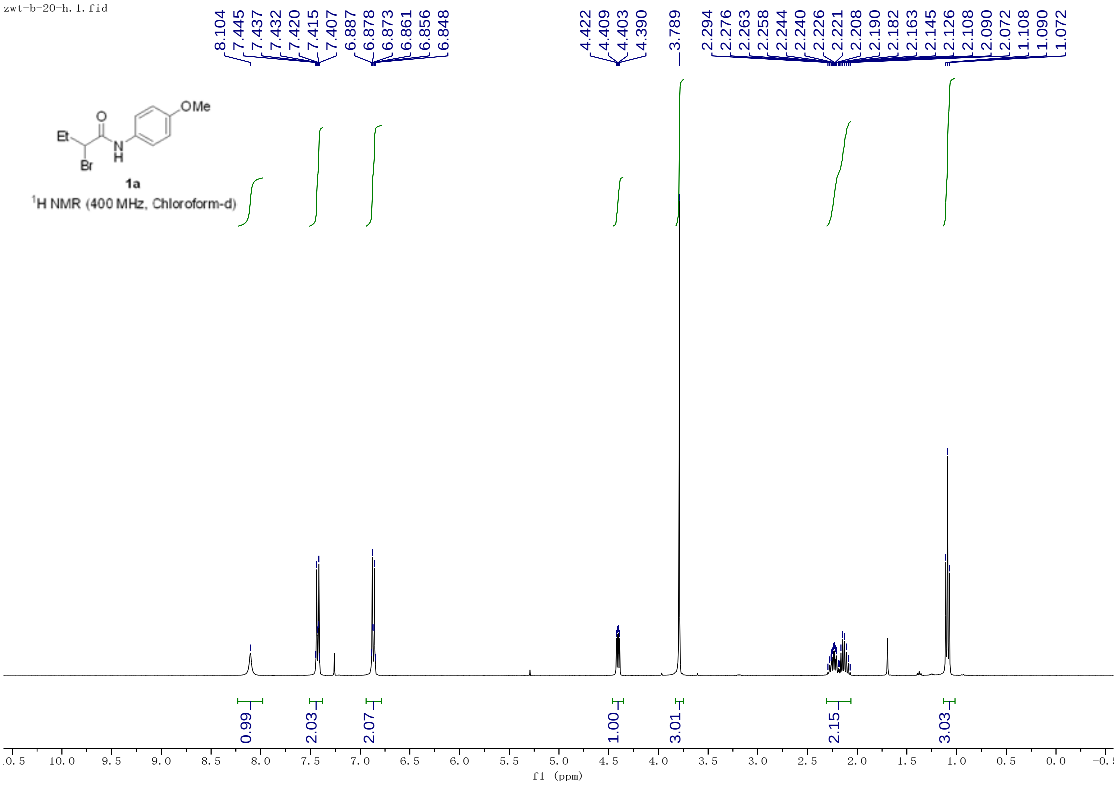

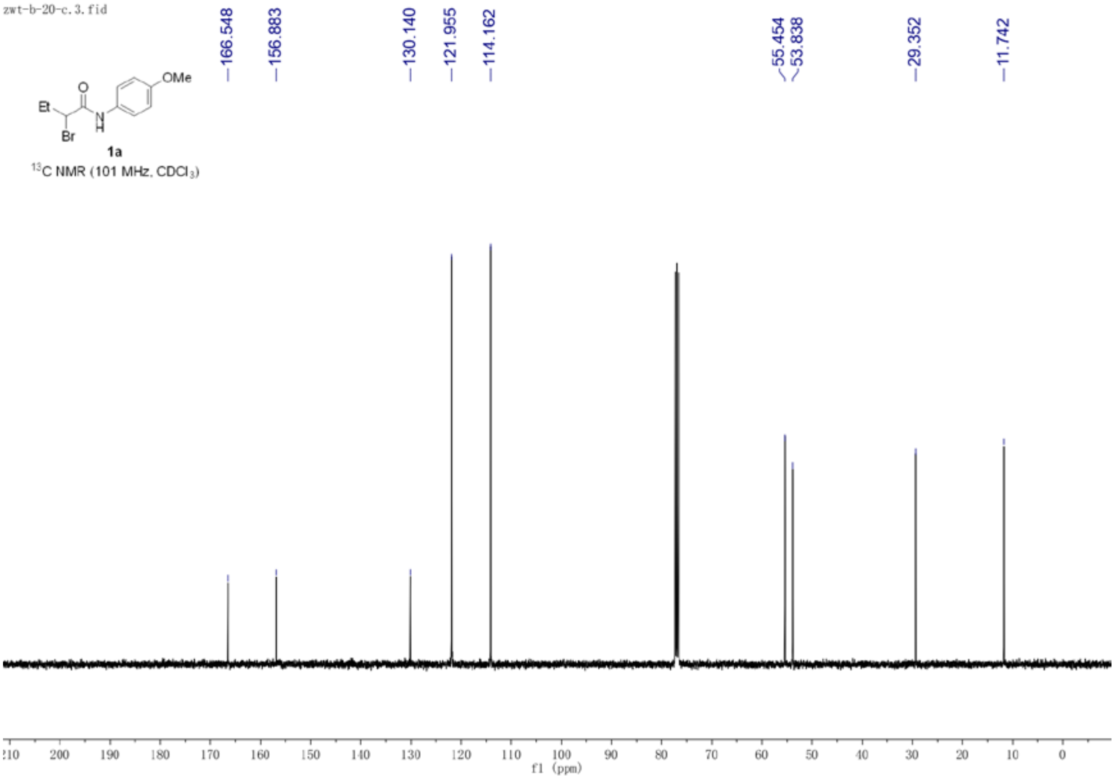

Supplementary Figure 58 ^{1}H-NMR (400 Mz, $CHCl_3$, 25 ℃) and ^{13}C-NMR (101 MHz, $CHCl_3$, 25 ℃) spectra of **1a**

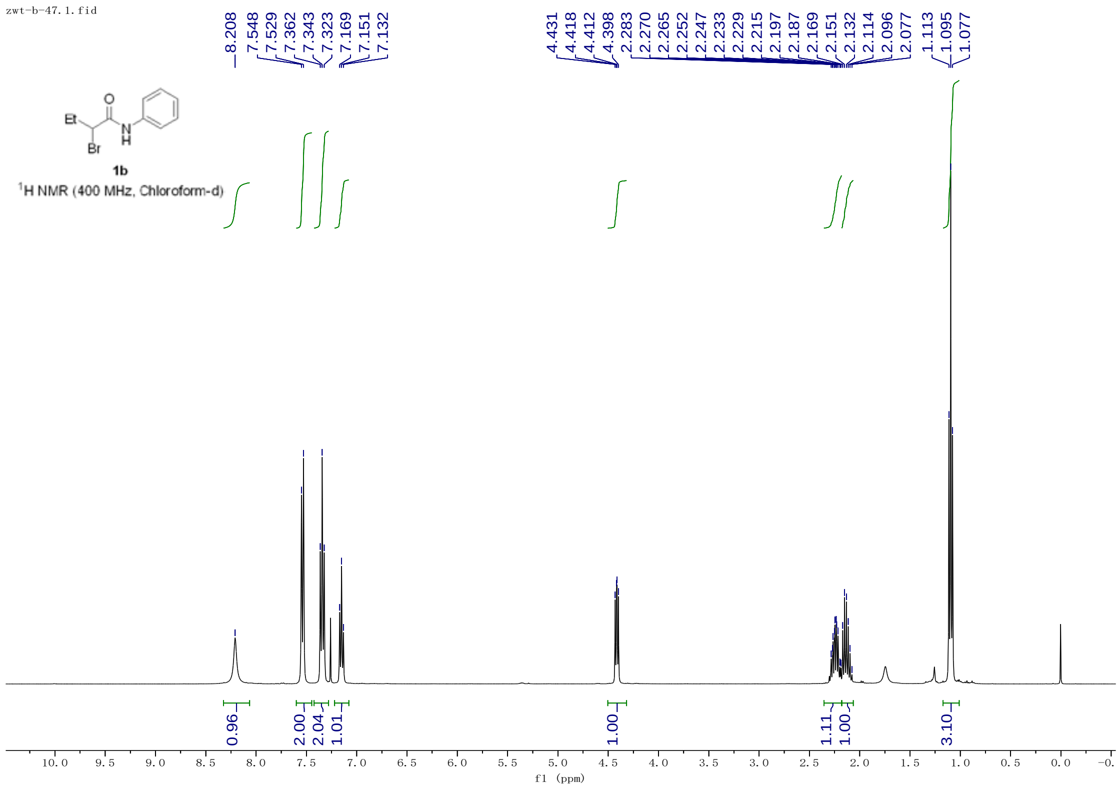

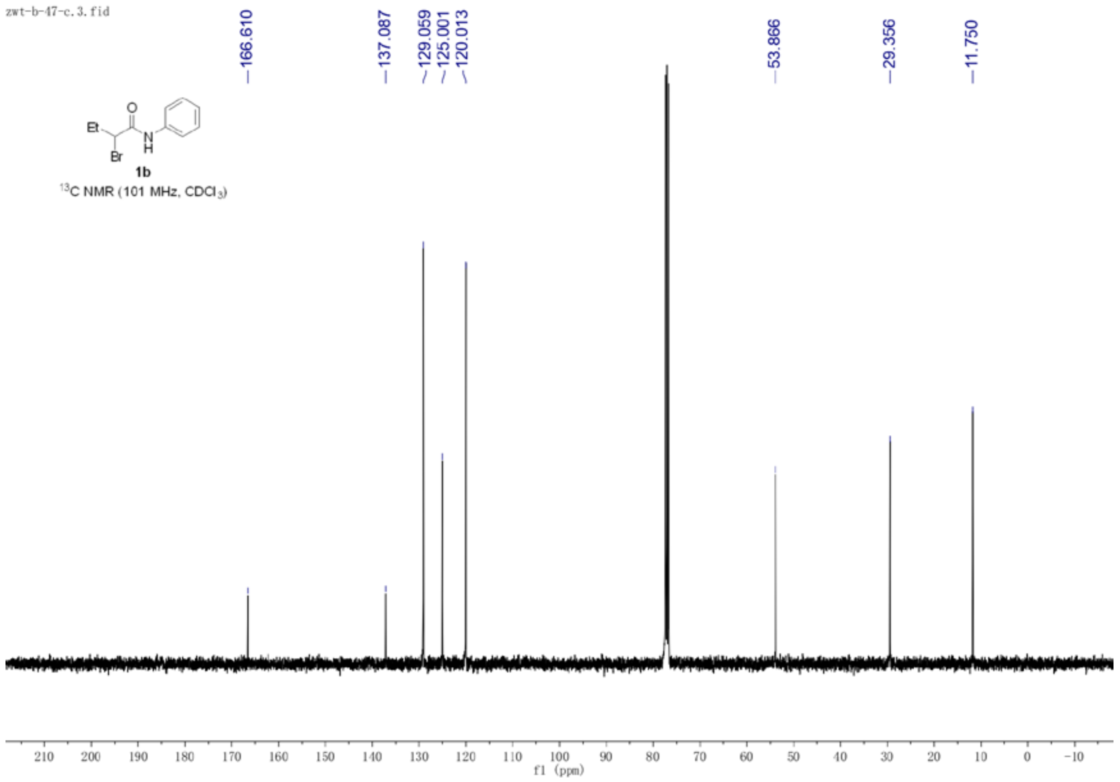

Supplementary Figure 59 ^{1}H-NMR (400 Mz, $CHCl_3$, 25 °C) and ^{13}C-NMR (101 MHz, $CHCl_3$, 25 °C) spectra of **1b**

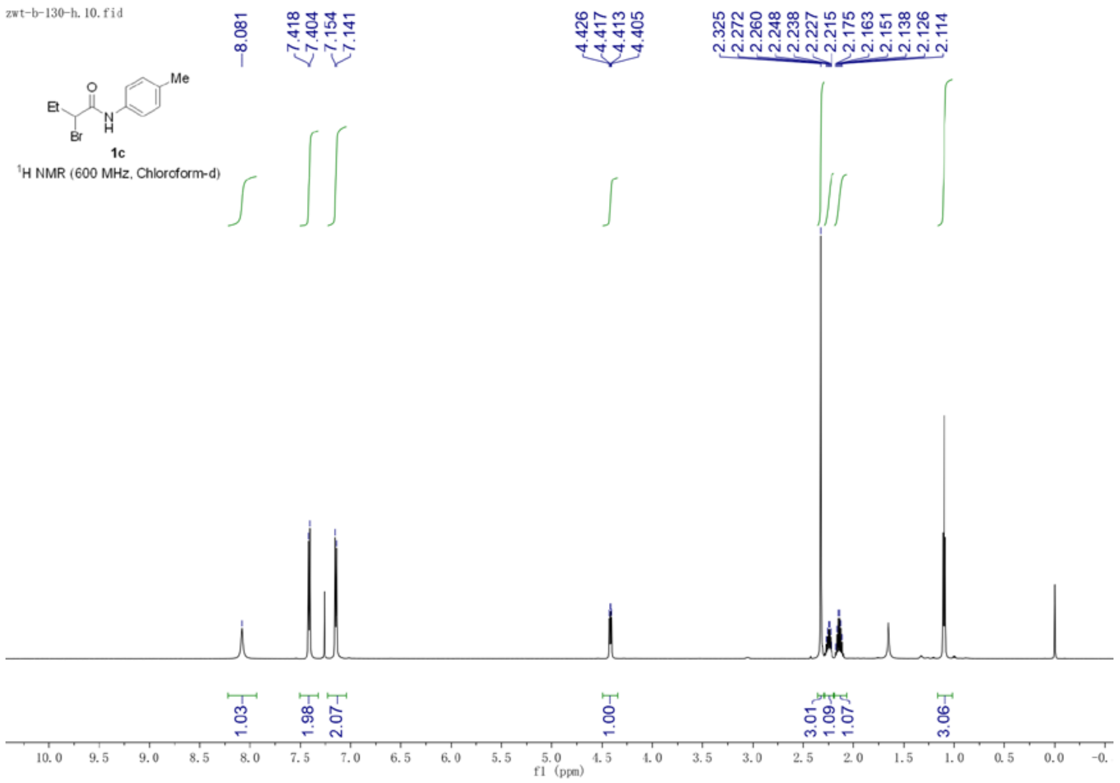

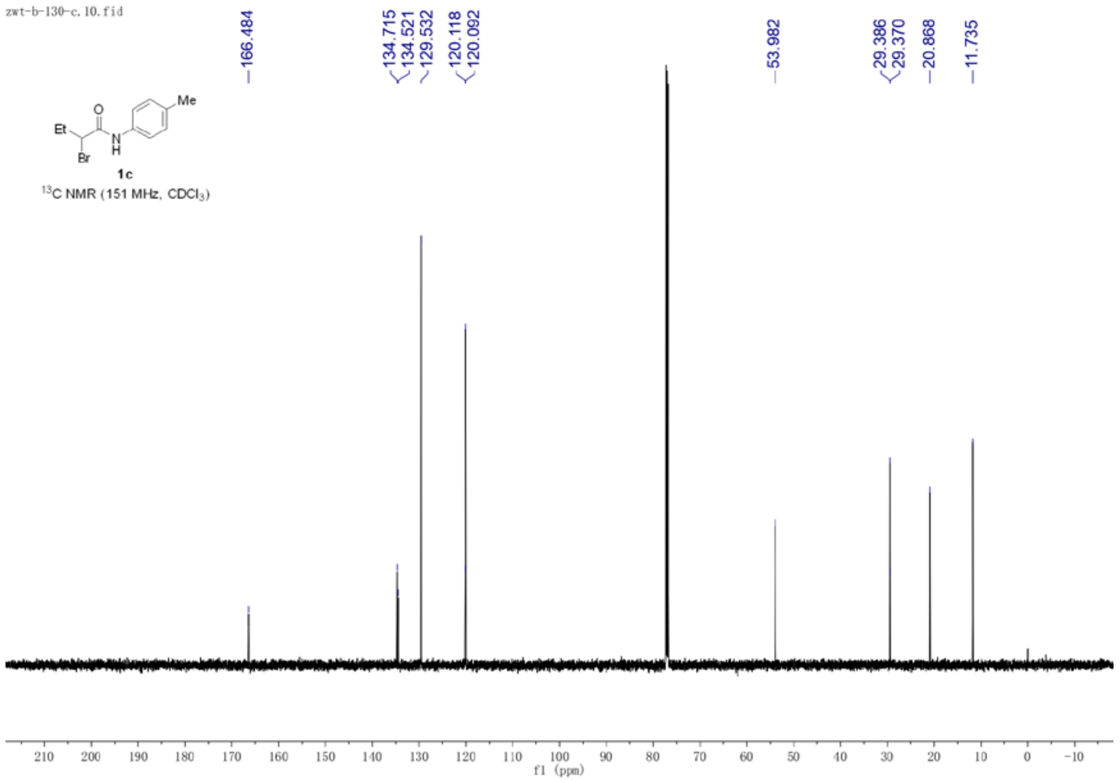

Supplementary Figure 60 ^{1}H-NMR (600 Mz, $CHCl_3$, 25 °C) and ^{13}C-NMR (151 MHz, $CHCl_3$, 25 °C) spectra of **1c**

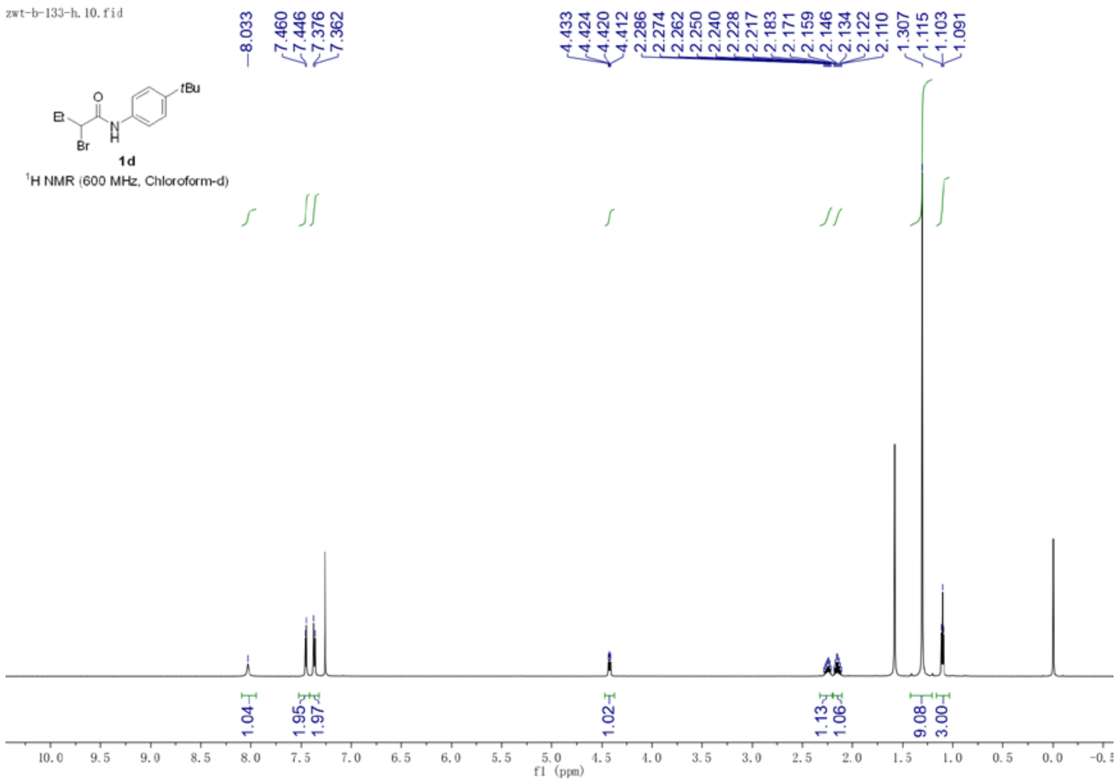

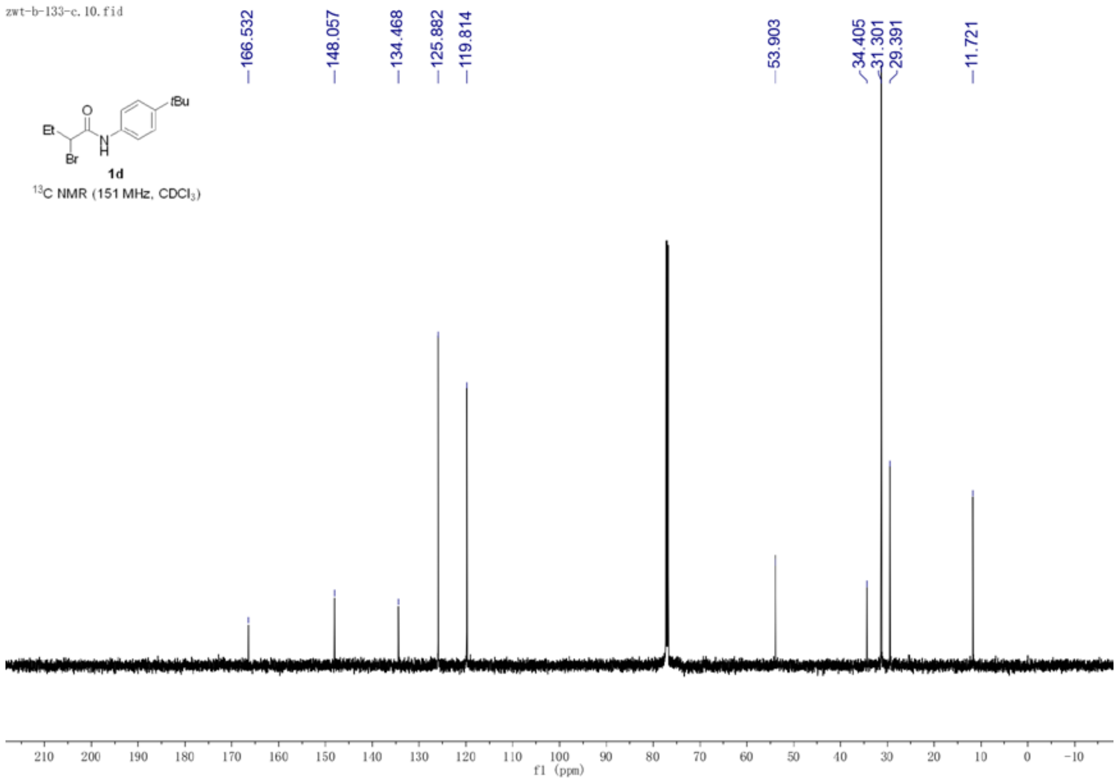

Supplementary Figure 61 ^{1}H-NMR (600 Mz, $CHCl_3$, 25 °C) and ^{13}C-NMR (151 MHz, $CHCl_3$, 25 °C) spectra of **1d**

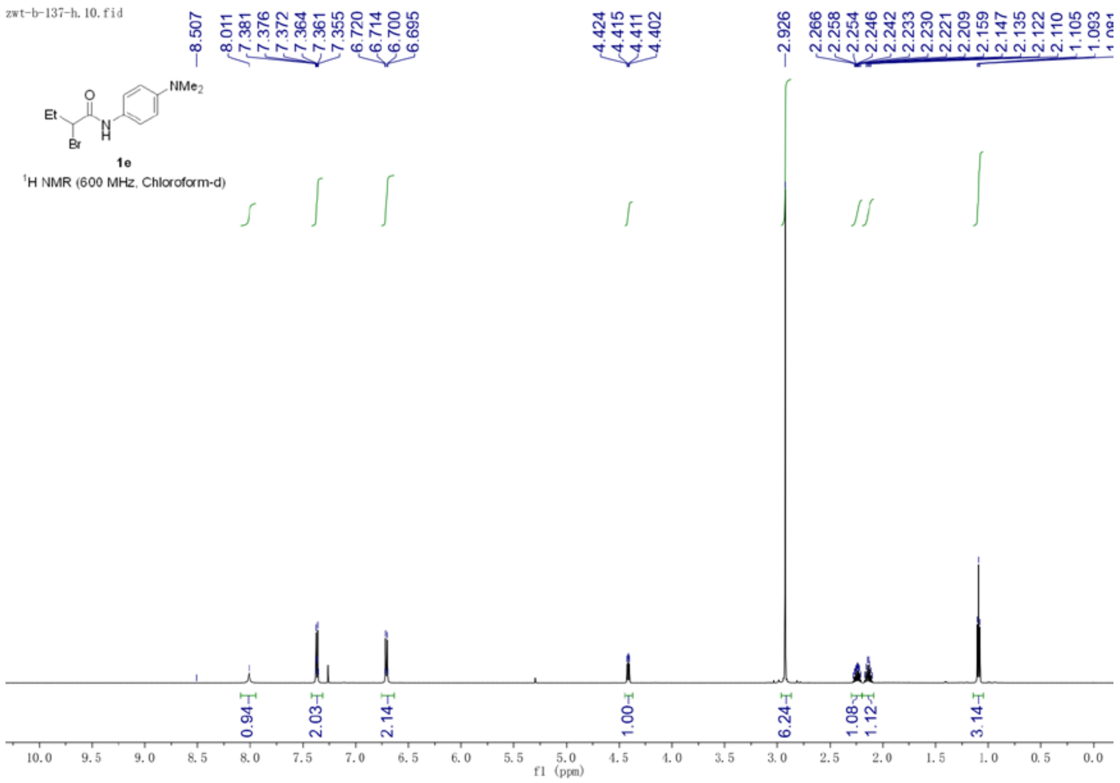

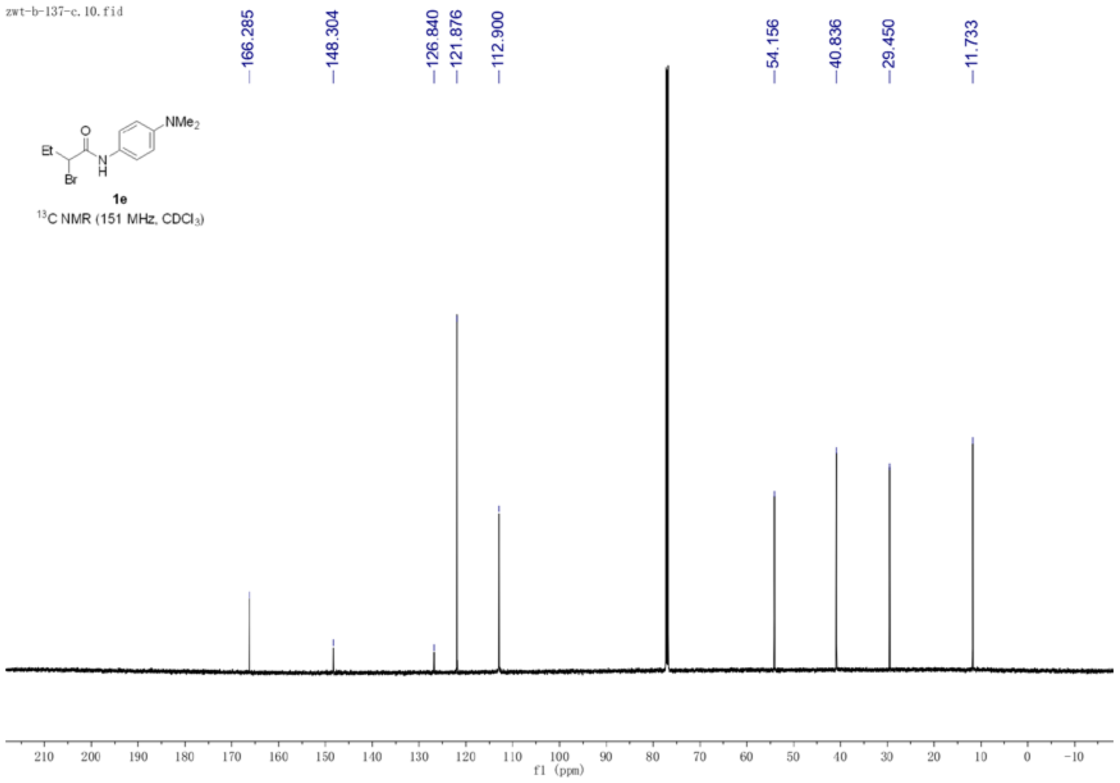

Supplementary Figure 62 ^{1}H-NMR (600 Mz, $CHCl_3$, 25 °C) and ^{13}C-NMR (151 MHz, $CHCl_3$, 25 °C) spectra of **1e**

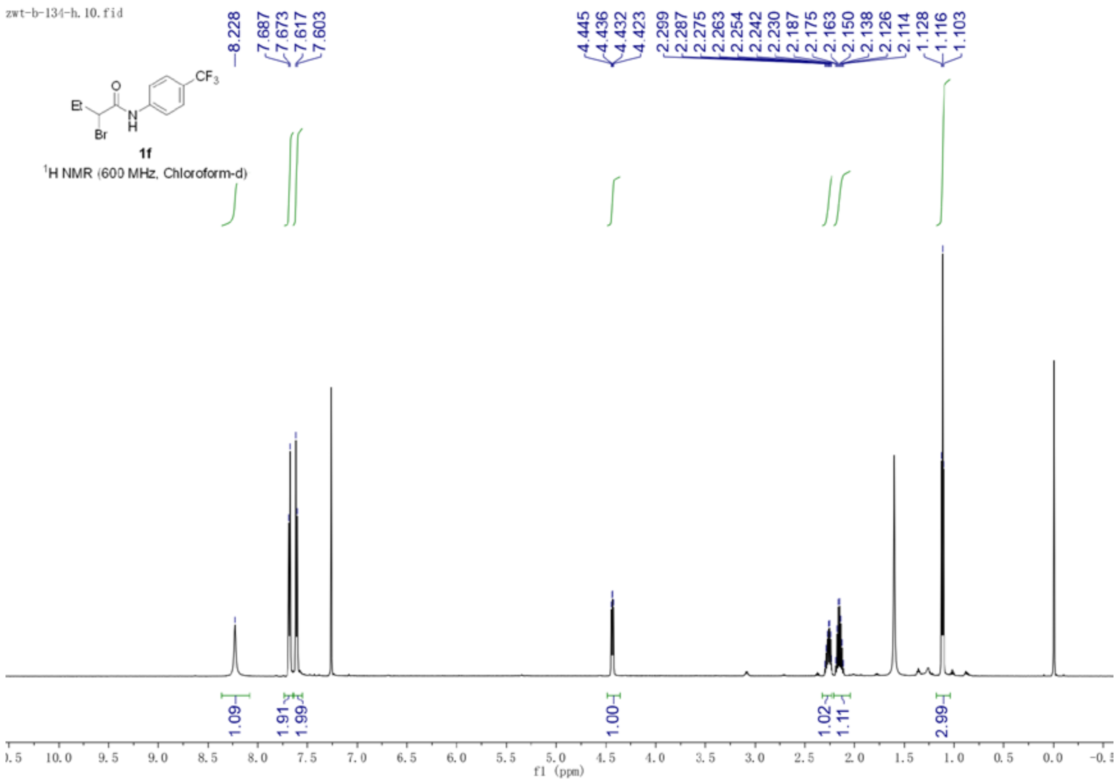

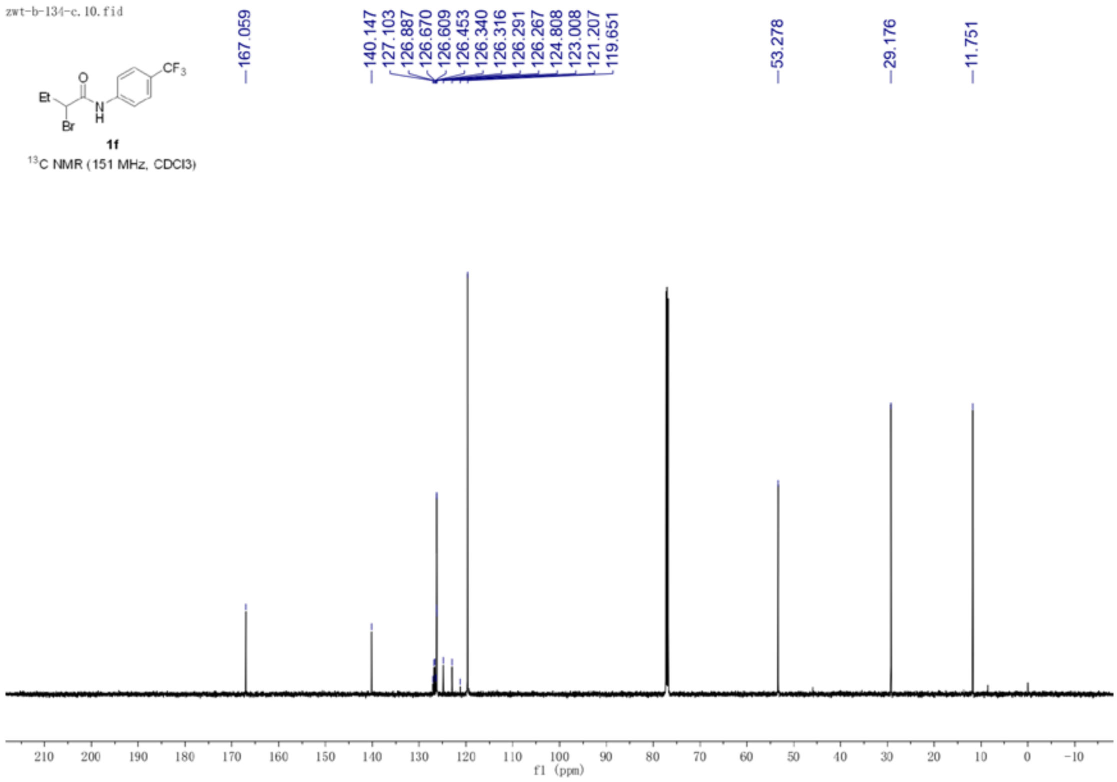

Supplementary Figure 63 ^{1}H-NMR (600 Mz, $CHCl_3$, 25 °C) and ^{13}C-NMR (151 MHz, $CHCl_3$, 25 °C) spectra of **1f**

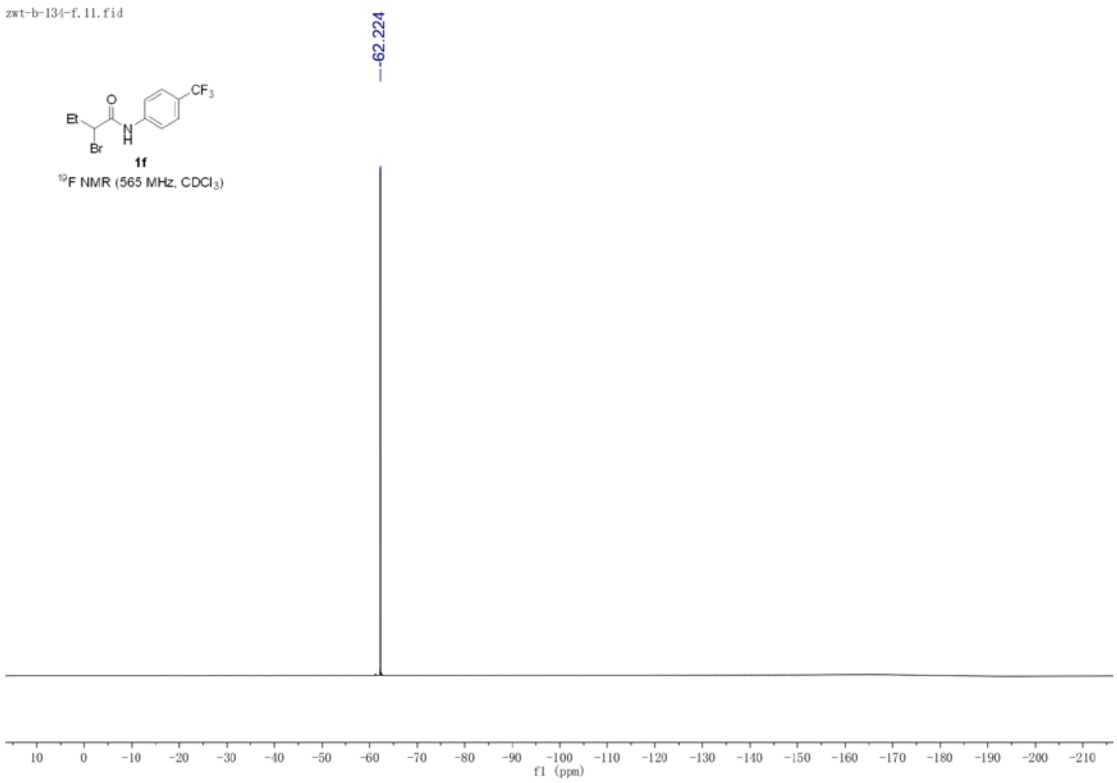

Supplementary Figure 64 ^{19}F-NMR (565 Mz, $CHCl_3$, 25 ℃) spectra of **1f**

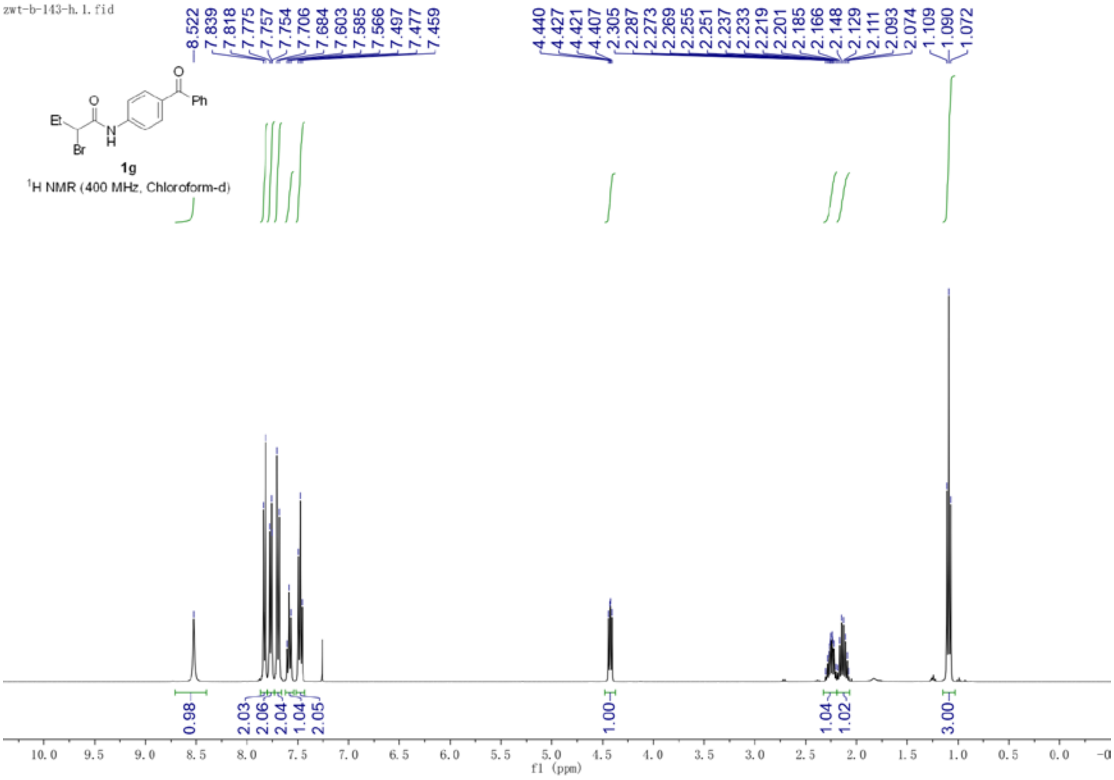

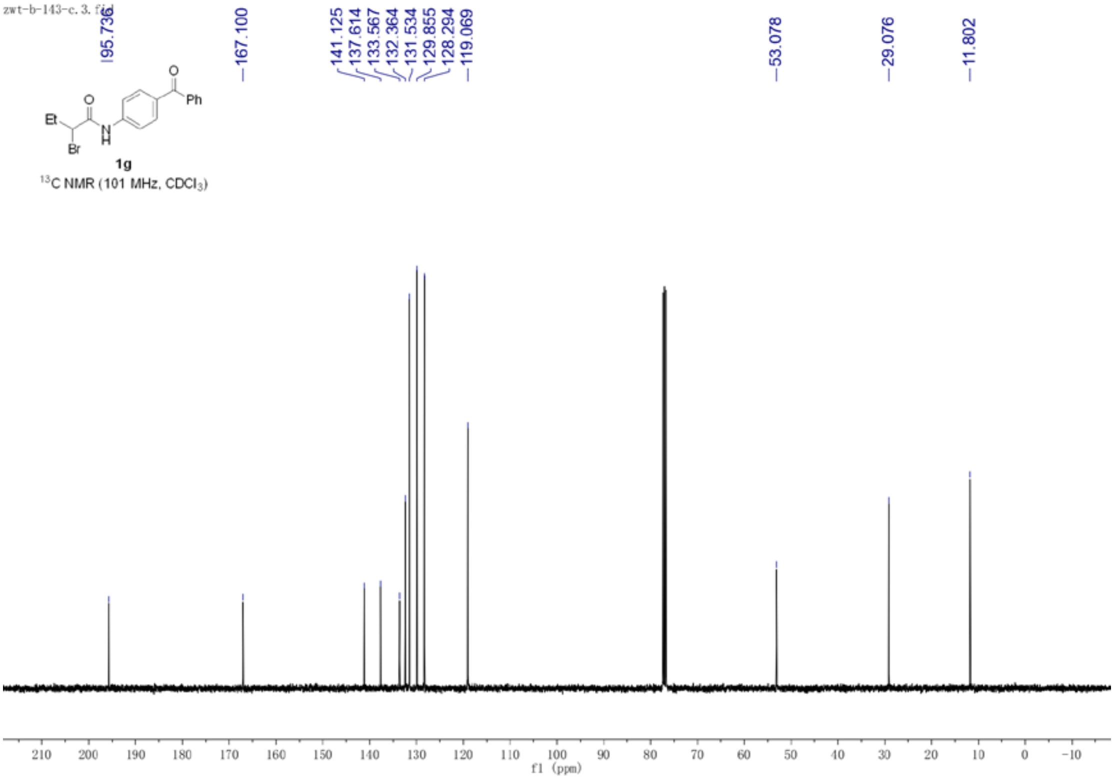

Supplementary Figure 65 ^{1}H-NMR (400 Mz, $CHCl_3$, 25 °C) and ^{13}C-NMR (101 MHz, $CHCl_3$, 25 °C) spectra of **1g**

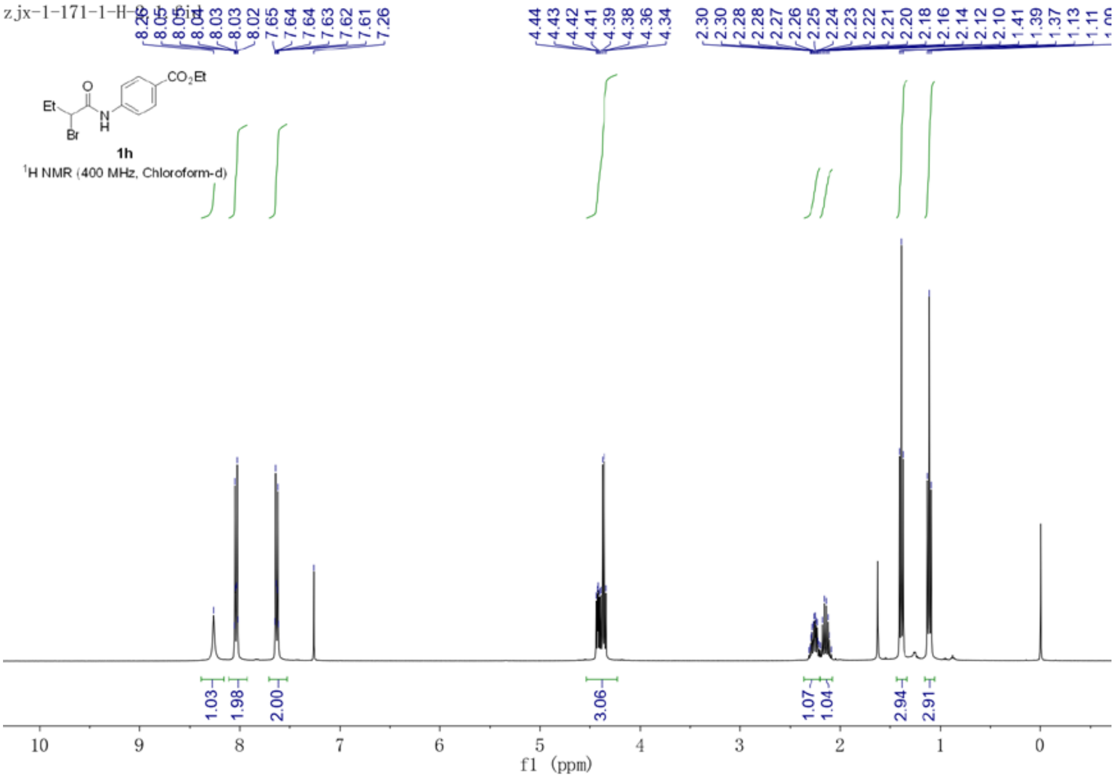

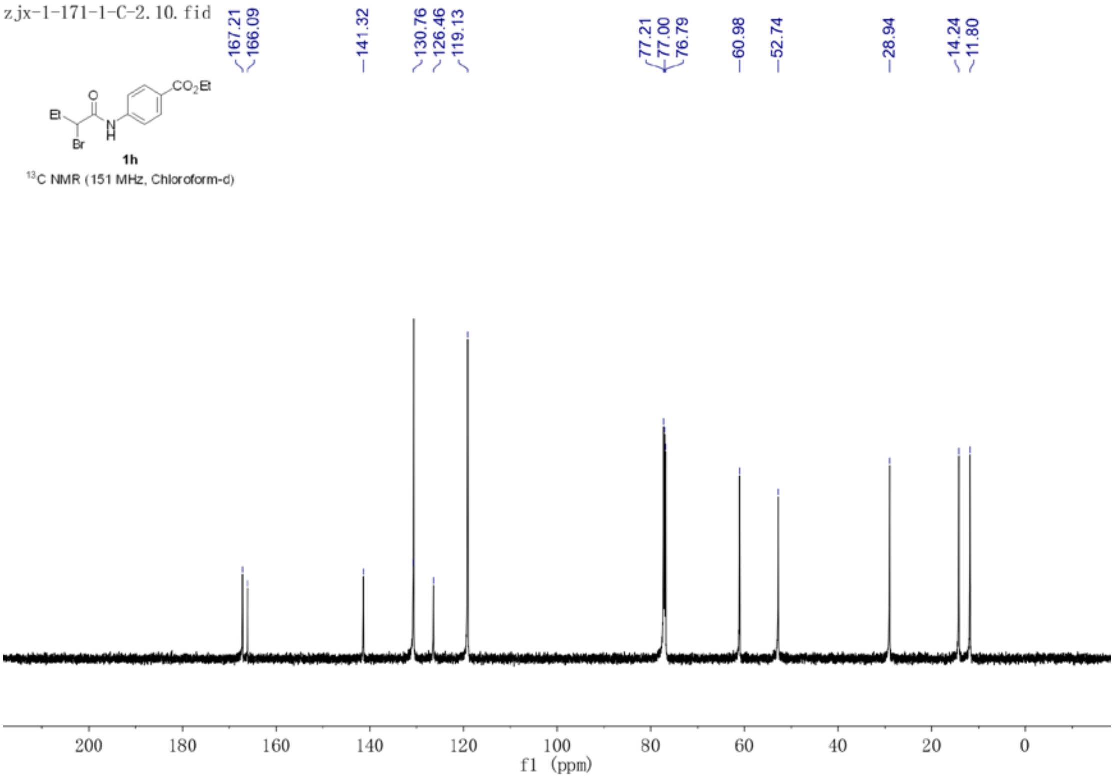

Supplementary Figure 66 ^{1}H-NMR (400 Mz, $CHCl_3$, 25 °C) and ^{13}C-NMR (151 MHz, $CHCl_3$, 25 °C) spectra of **1h**

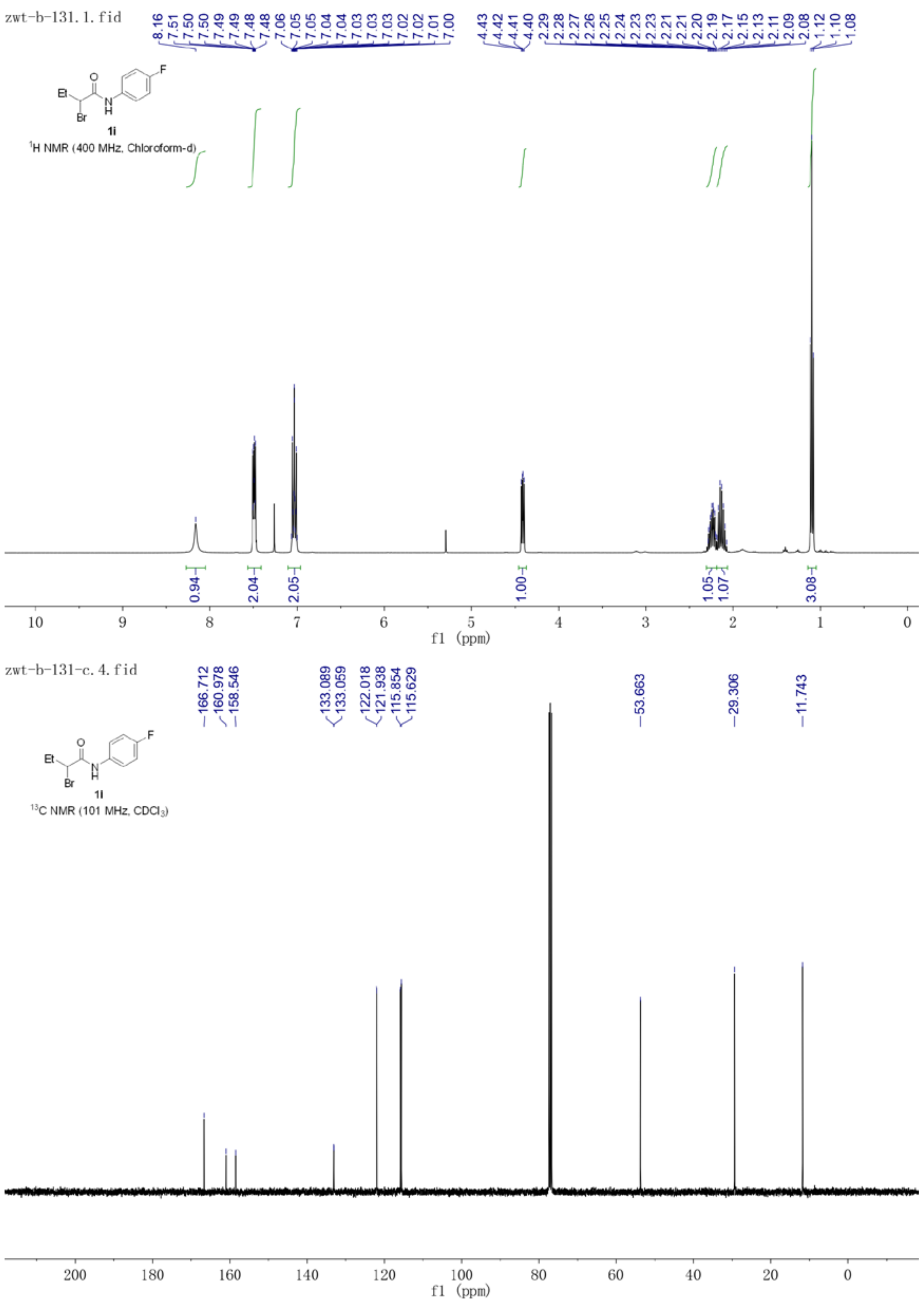

Supplementary Figure 67 ^{1}H-NMR (400 Mz, $CHCl_3$, 25 °C) and ^{13}C-NMR (101 MHz, $CHCl_3$, 25 °C) spectra of **1i**

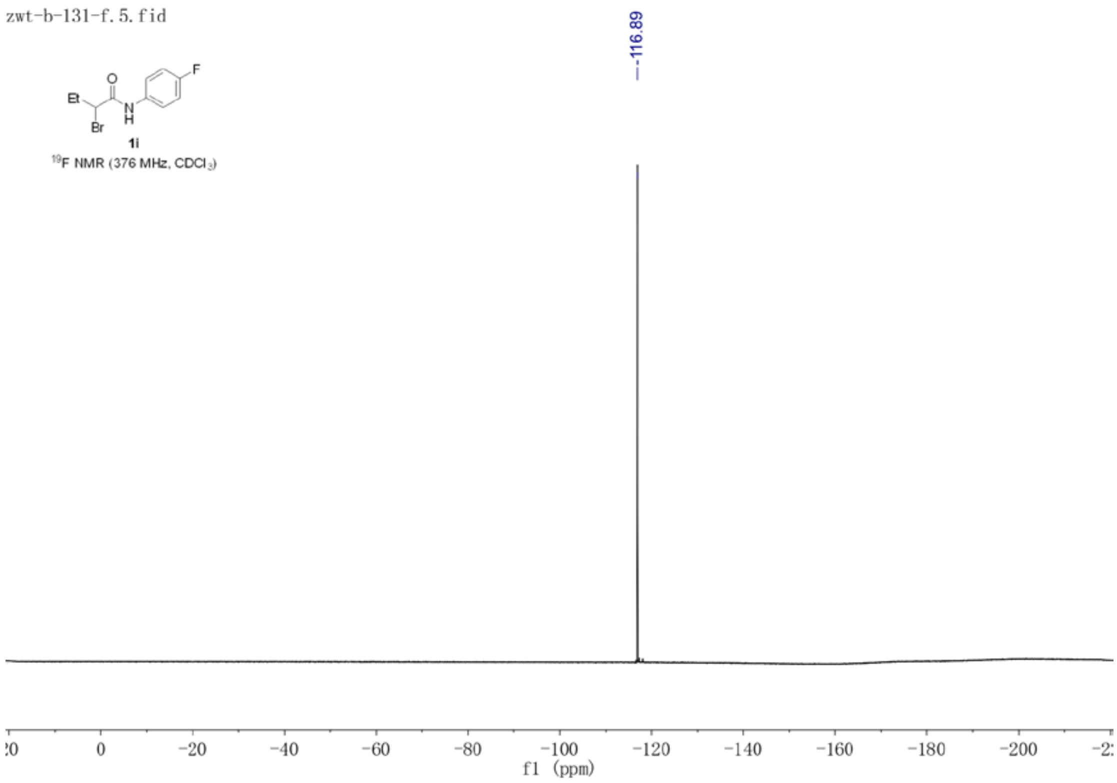

Supplementary Figure 68 ^{19}F-NMR (376 Mz, $CHCl_3$, 25 ℃) spectra of **1i**

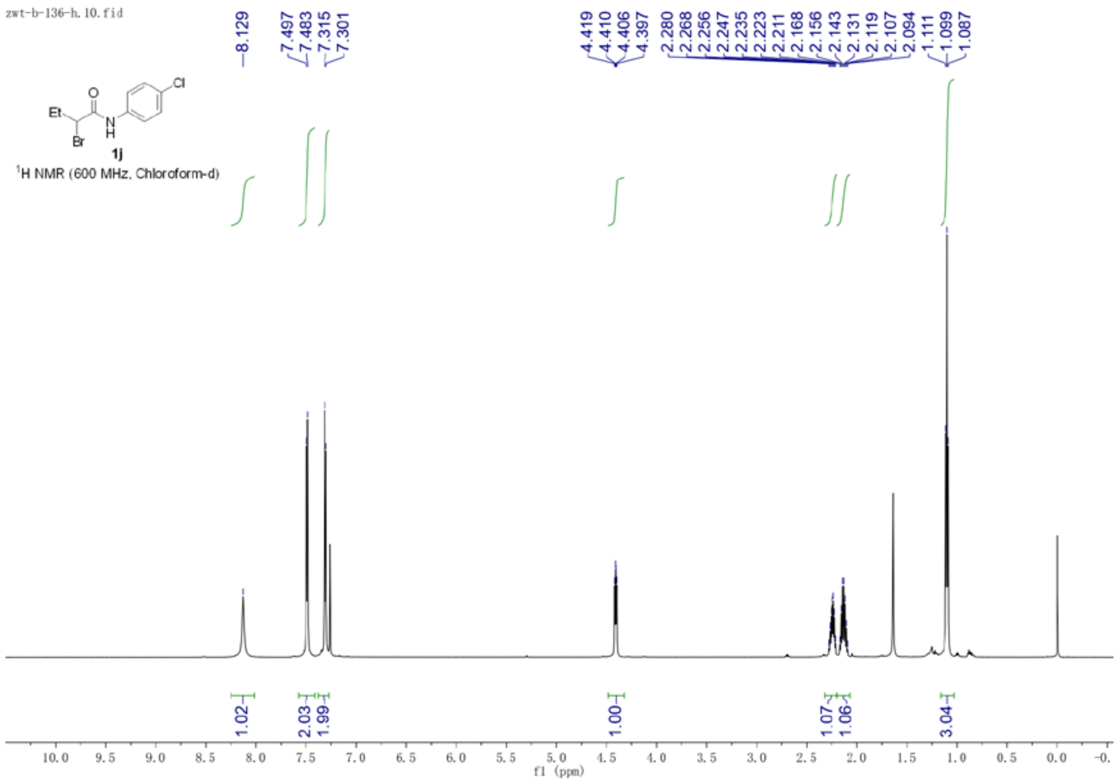

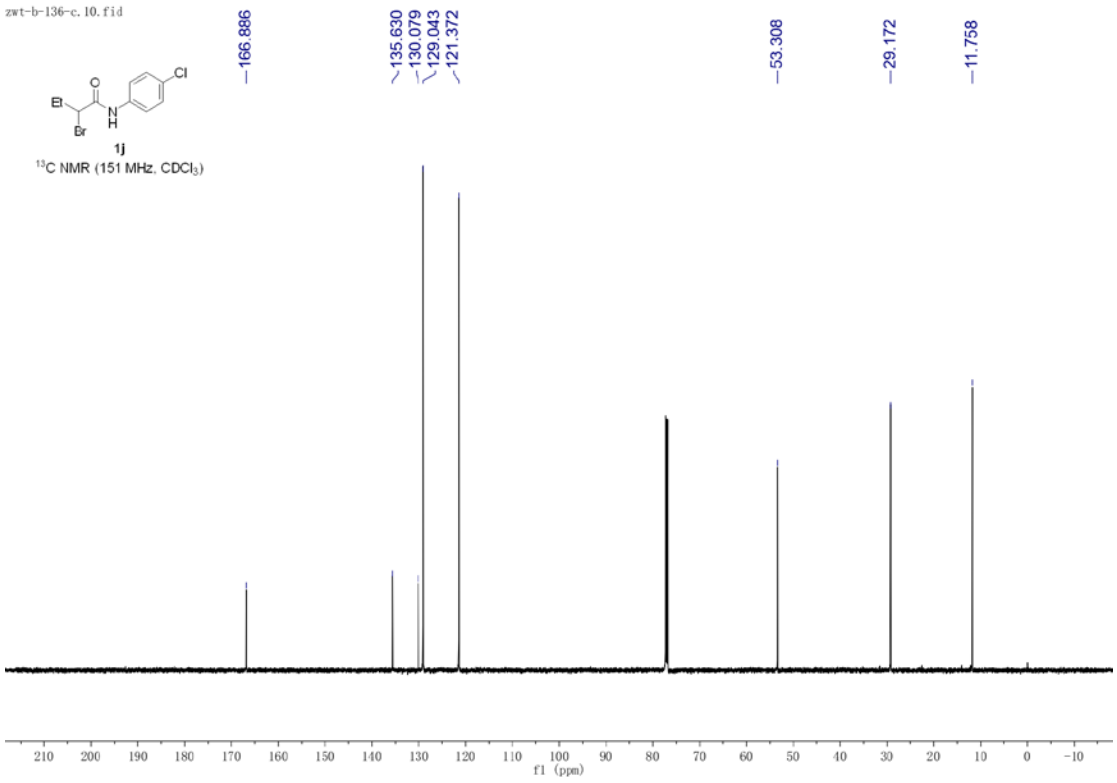

Supplementary Figure 69 ^{1}H-NMR (600 Mz, $CHCl_3$, 25 °C) and ^{13}C-NMR (151 MHz, $CHCl_3$, 25 °C) spectra of **1j**

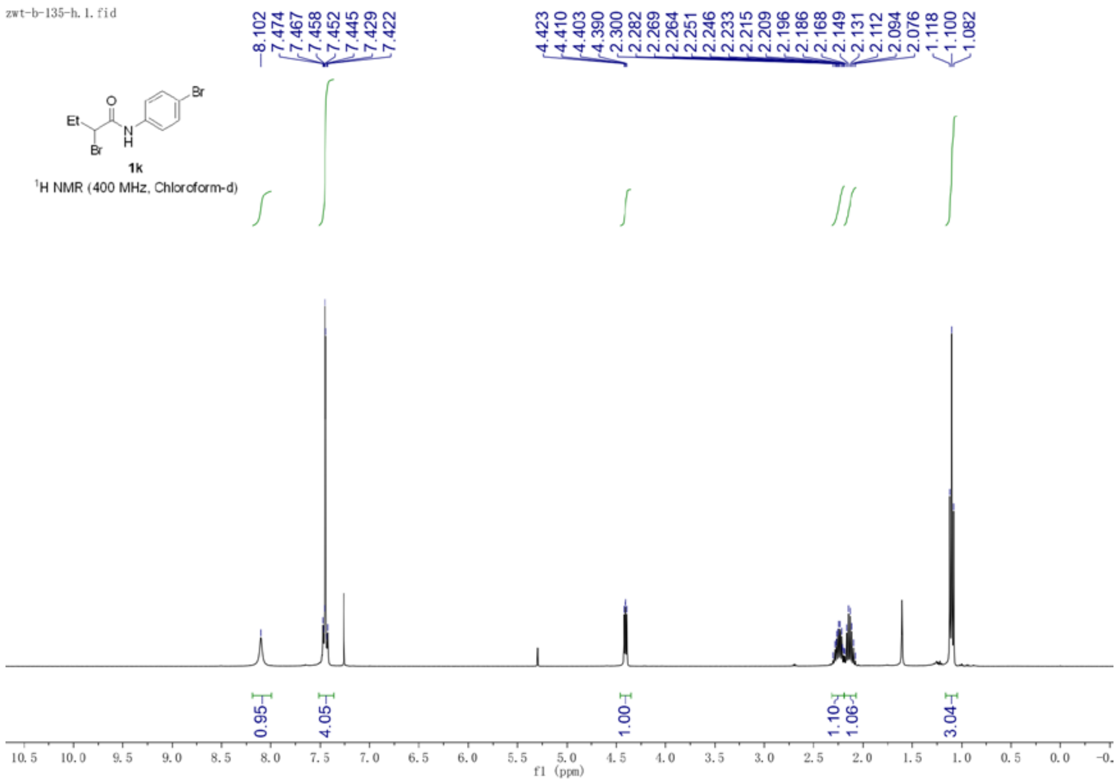

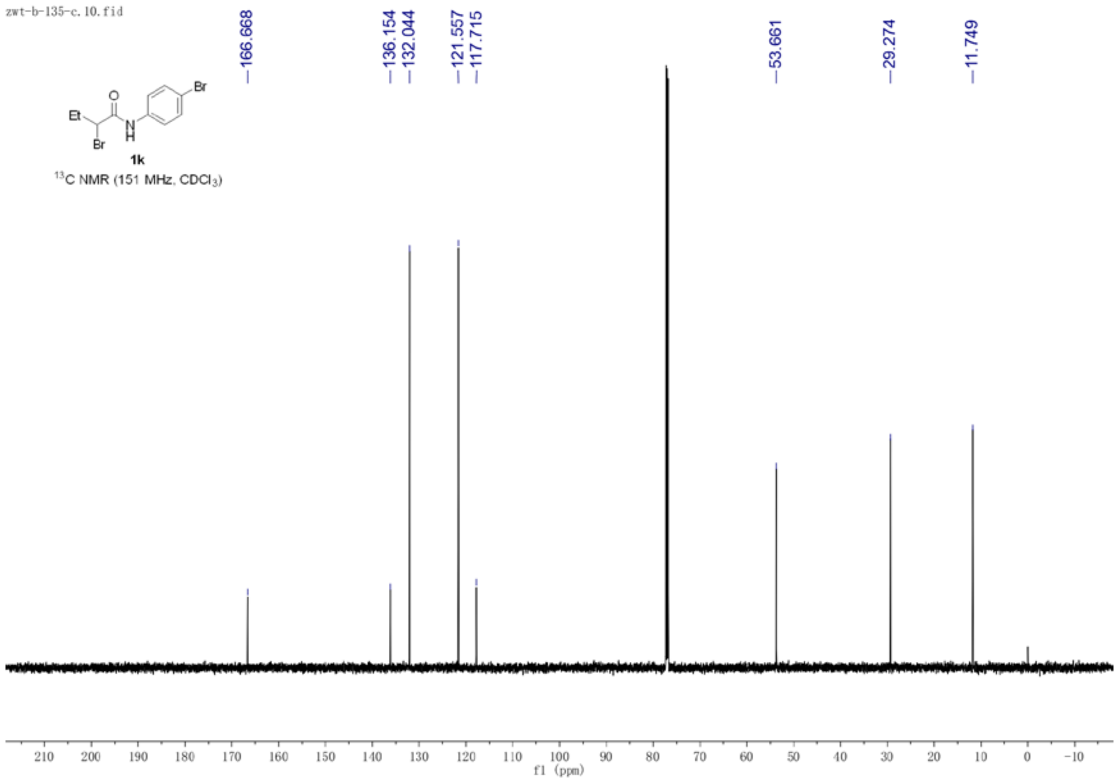

Supplementary Figure 70 ^{1}H-NMR (400 Mz, $CHCl_3$, 25 °C) and ^{13}C-NMR (151 MHz, $CHCl_3$, 25 °C) spectra of **1k**

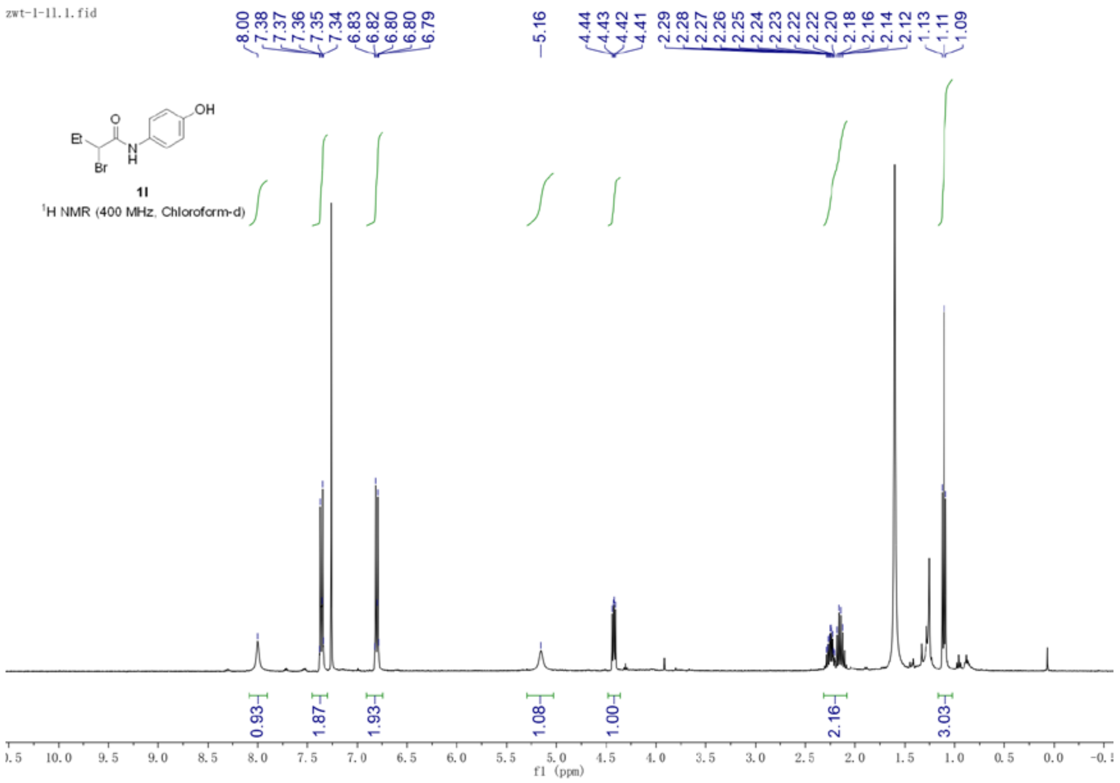

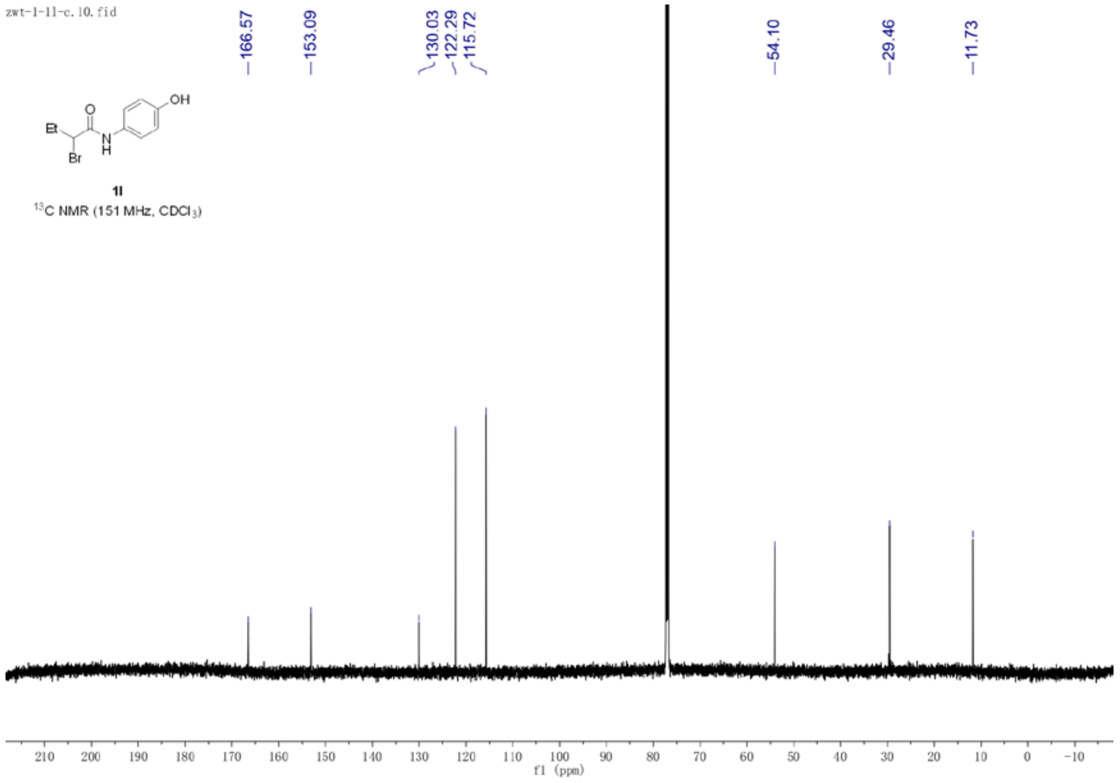

Supplementary Figure 71 ^{1}H-NMR (400 Mz, $CHCl_3$, 25 °C) and ^{13}C-NMR (151 MHz, $CHCl_3$, 25 °C) spectra of **1l**

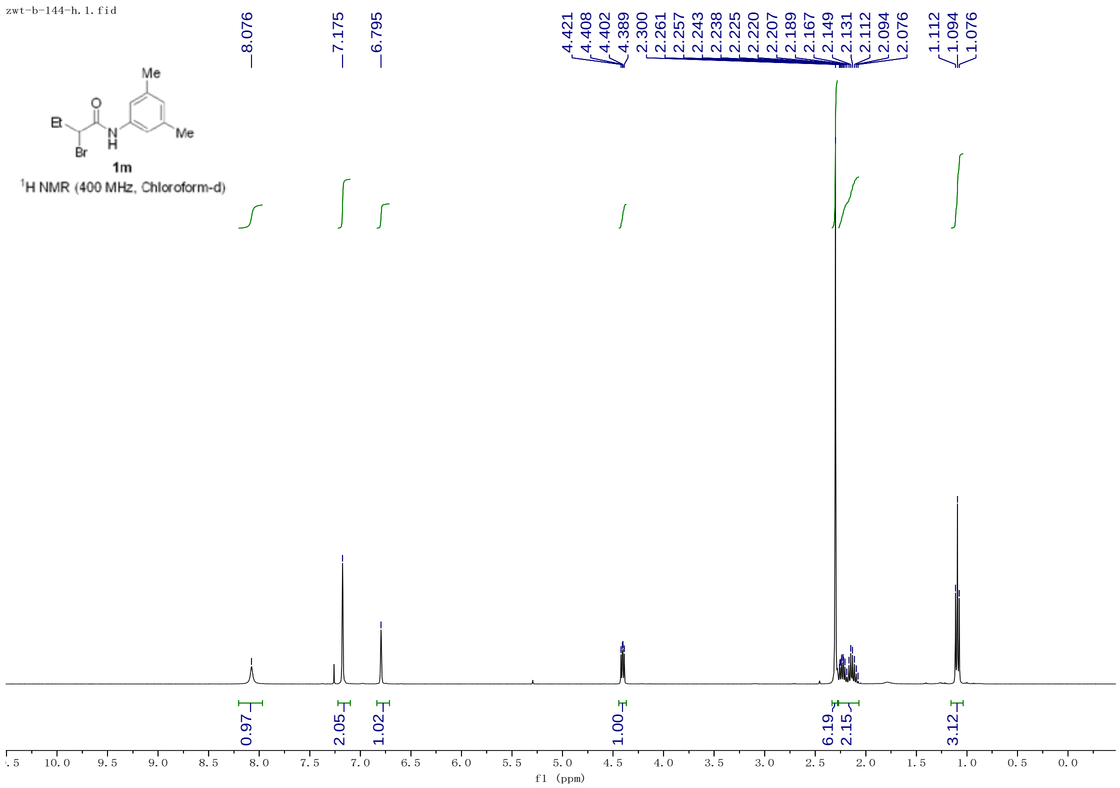

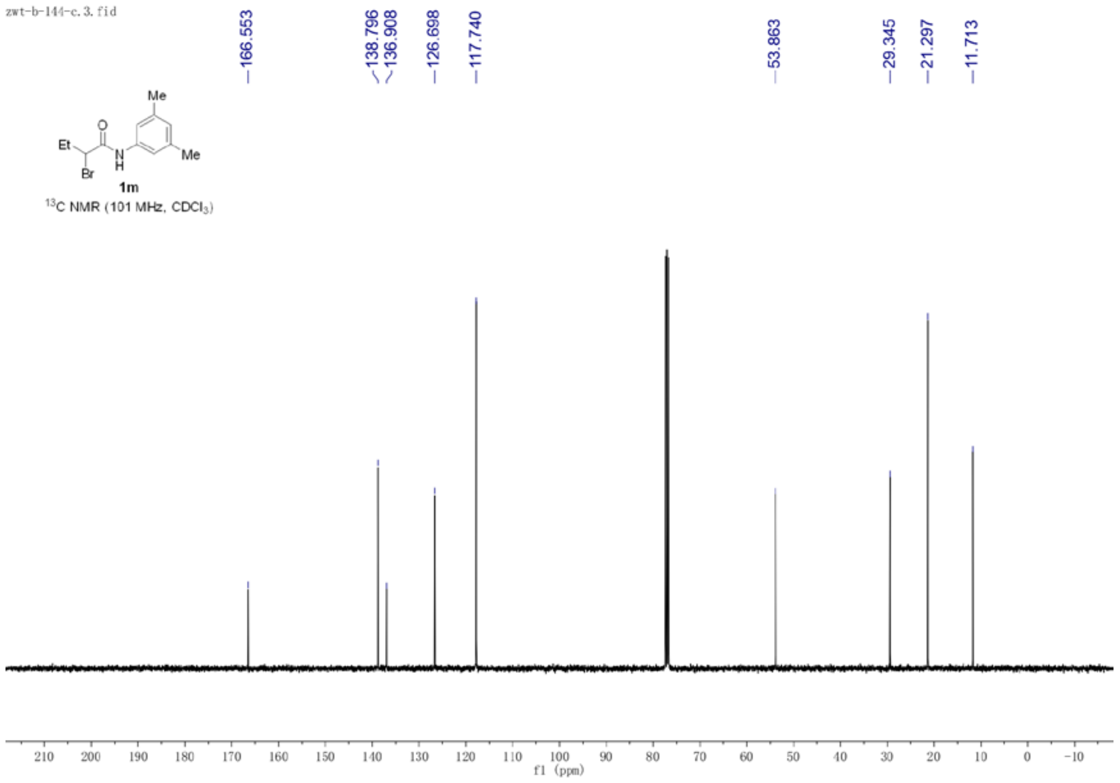

Supplementary Figure 72 ^{1}H-NMR (400 Mz, $CHCl_3$, 25 °C) and ^{13}C-NMR (101 MHz, $CHCl_3$, 25 °C) spectra of **1m**

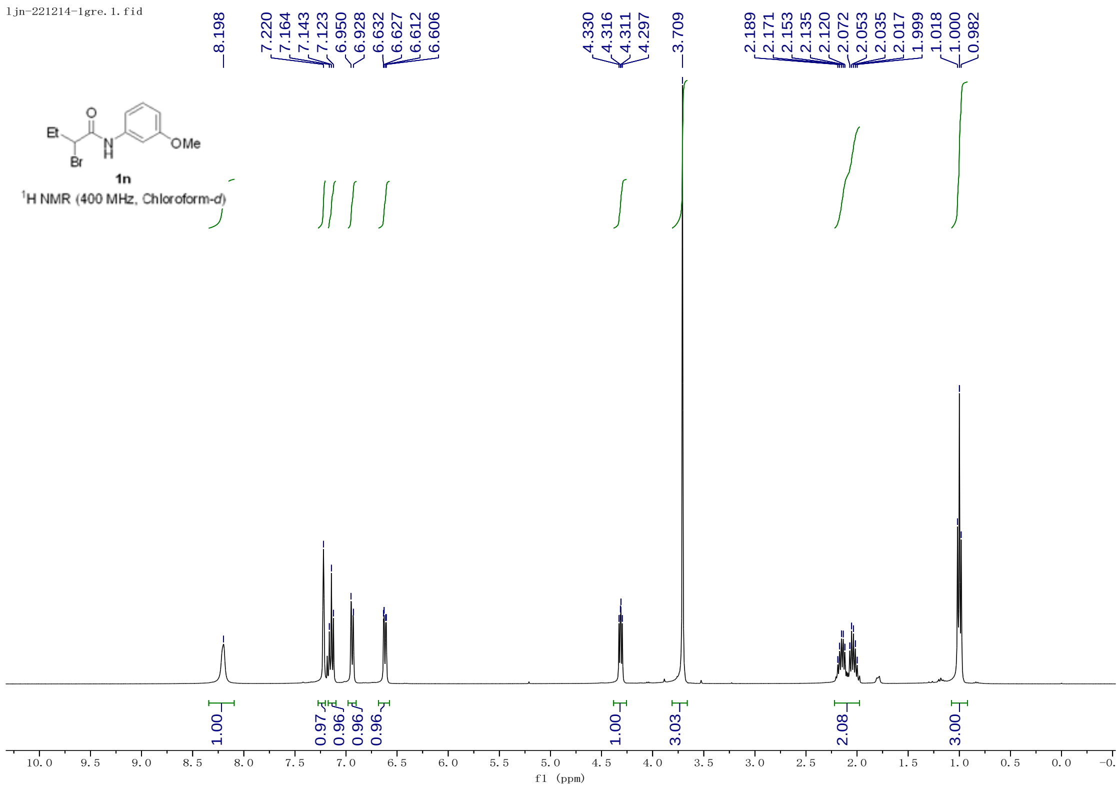

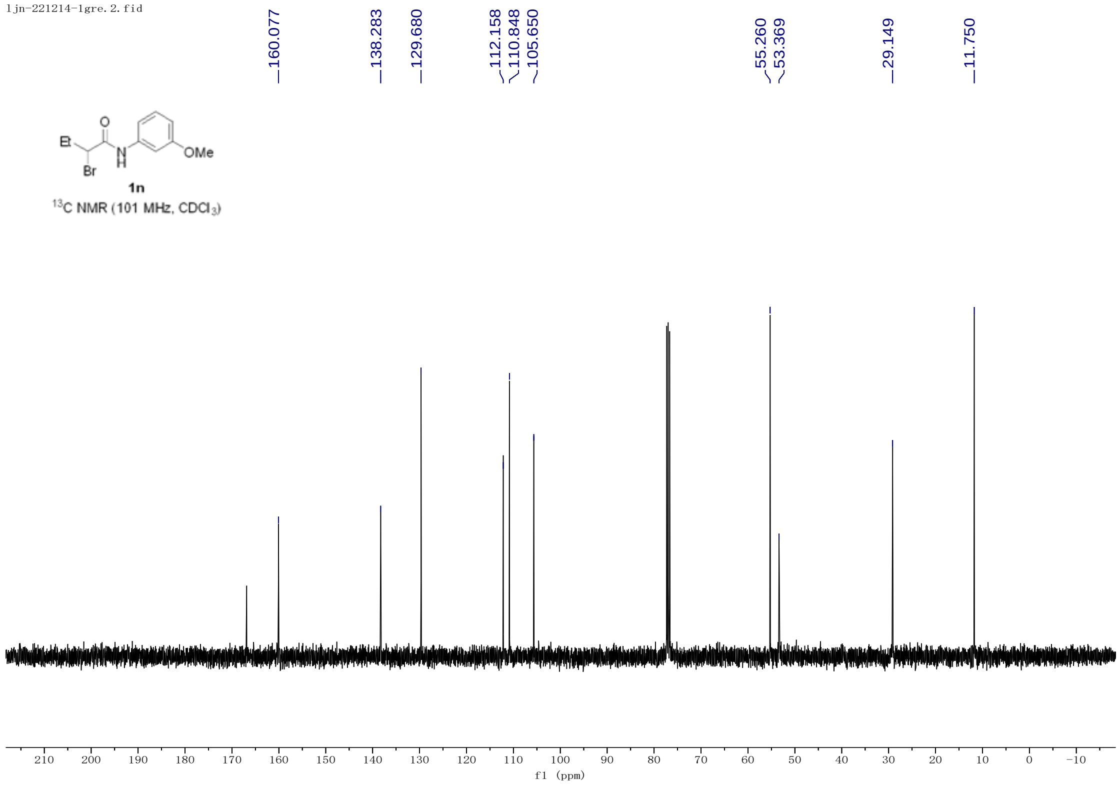

Supplementary Figure 73 ^{1}H-NMR (400 Mz, $CHCl_3$, 25 °C) and ^{13}C-NMR (101 MHz, $CHCl_3$, 25 °C) spectra of **1n**

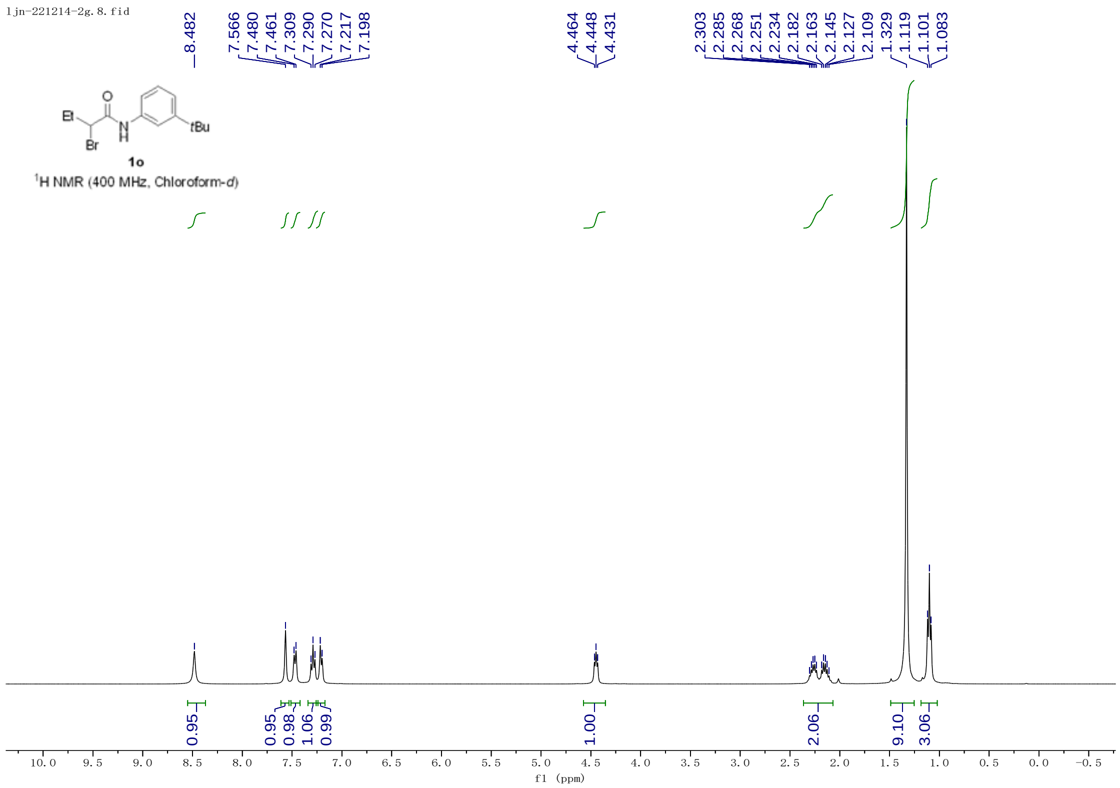

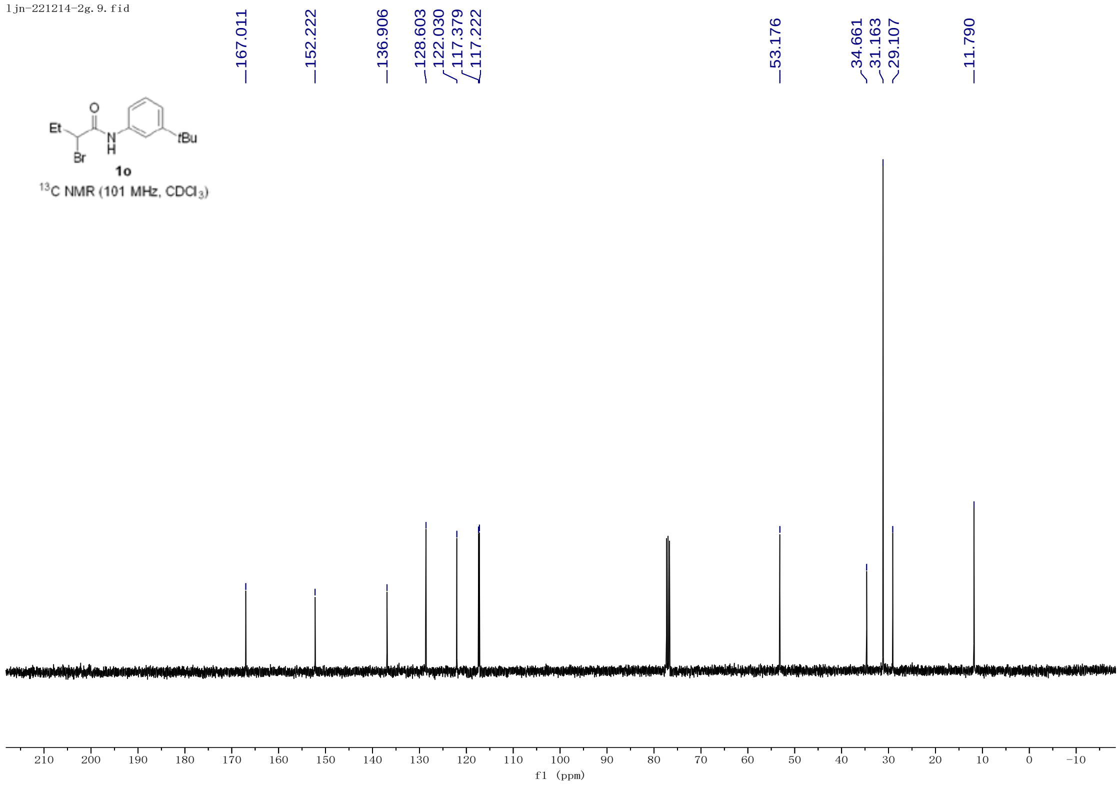

Supplementary Figure 74 ^{1}H-NMR (400 Mz, $CHCl_3$, 25 °C) and ^{13}C-NMR (101 MHz, $CHCl_3$, 25 °C) spectra of **1o**

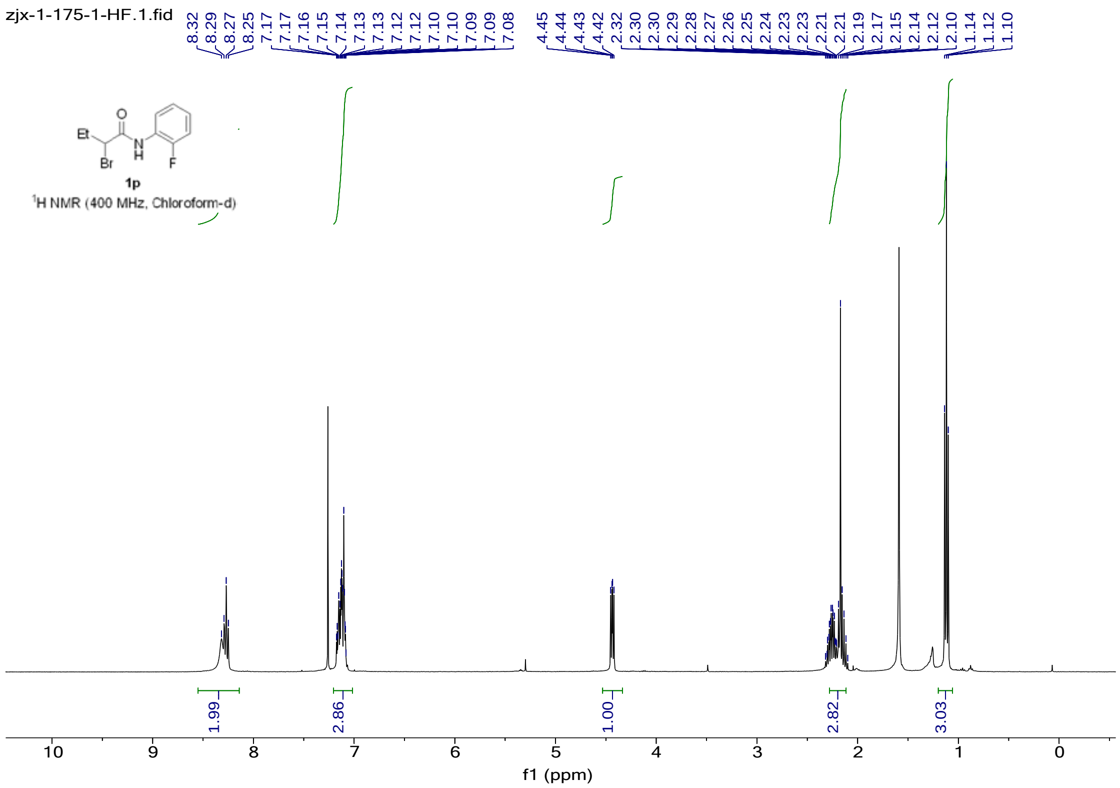

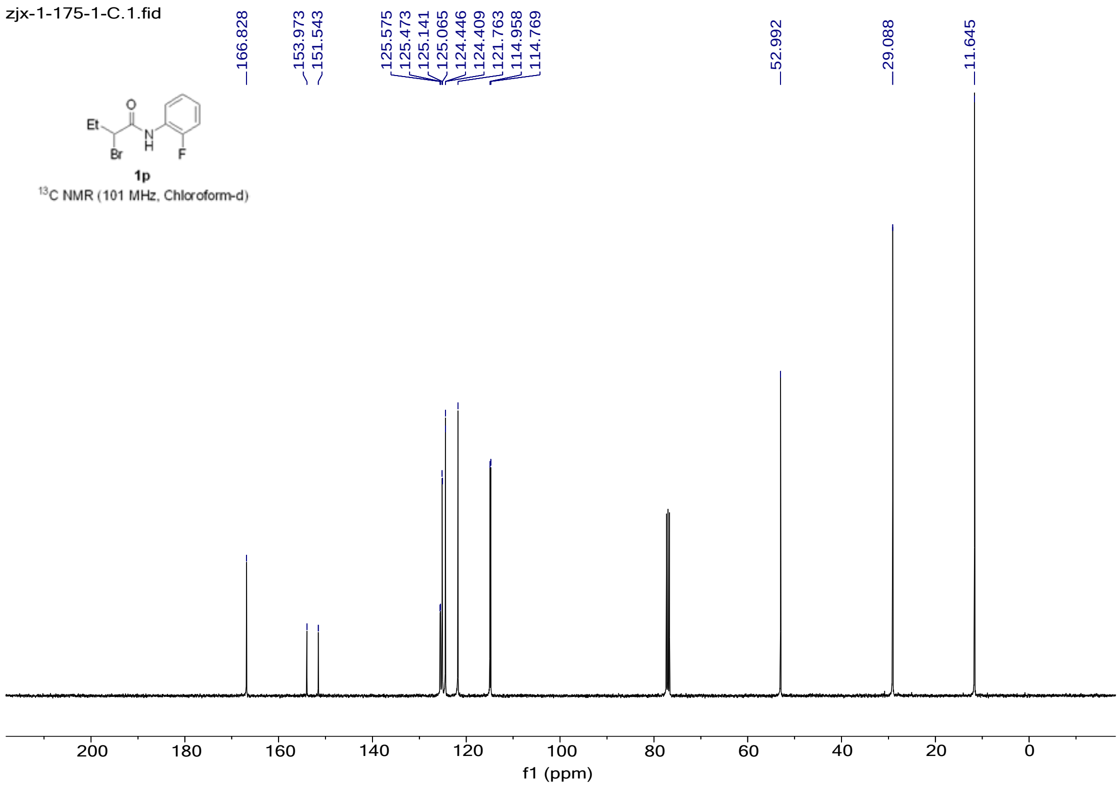

Supplementary Figure 75 ^{1}H-NMR (400 Mz, $CHCl_3$, 25 °C) and ^{13}C-NMR (101 MHz, $CHCl_3$, 25 °C) spectra of **1p**

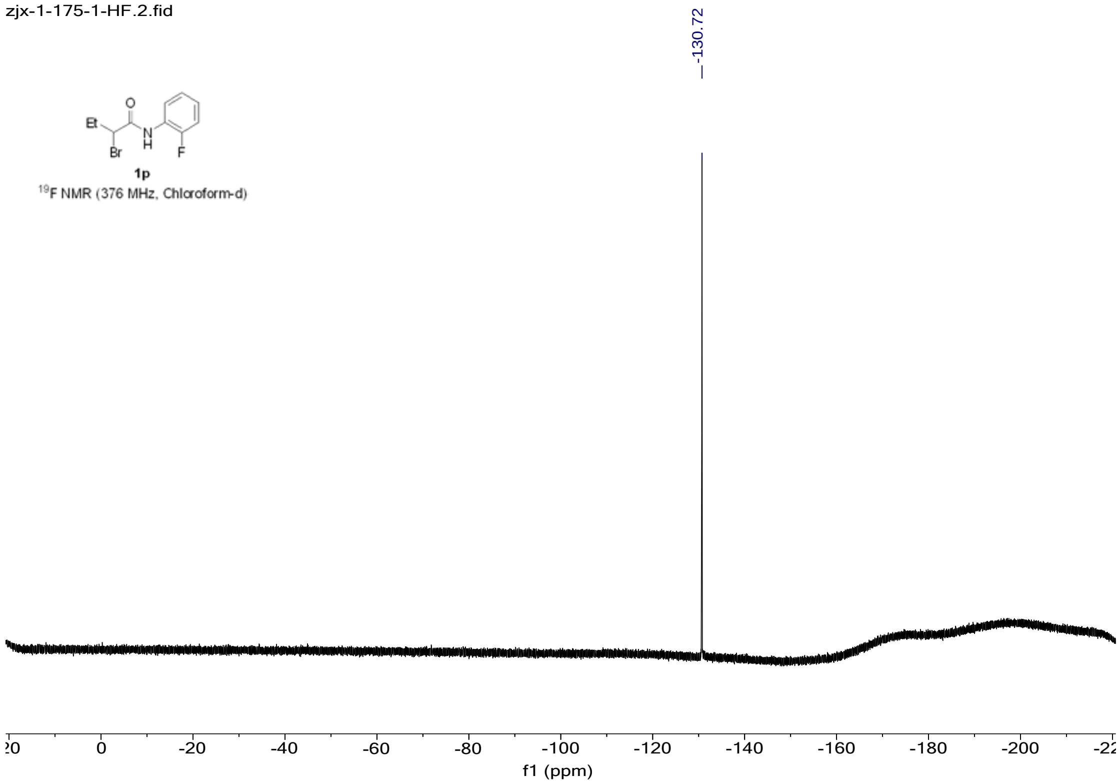

Supplementary Figure 76 ^{19}F-NMR (376 Mz, $CHCl_3$, 25 °C) spectra of **1p**

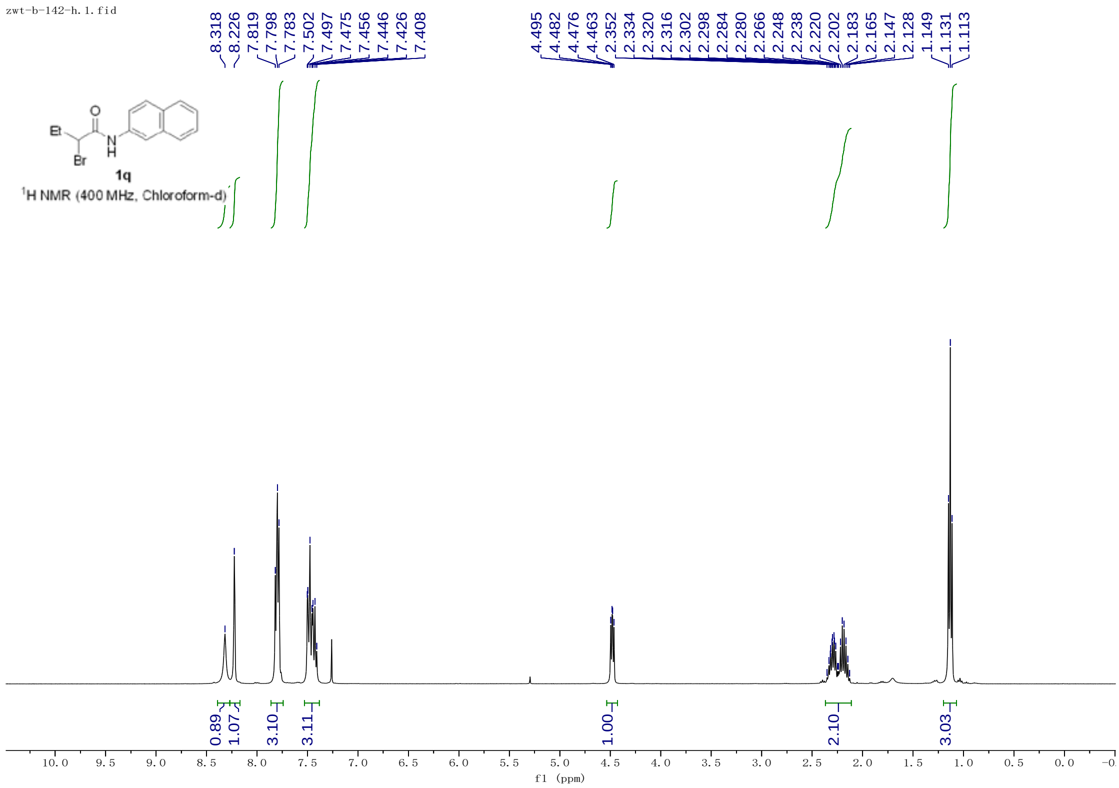

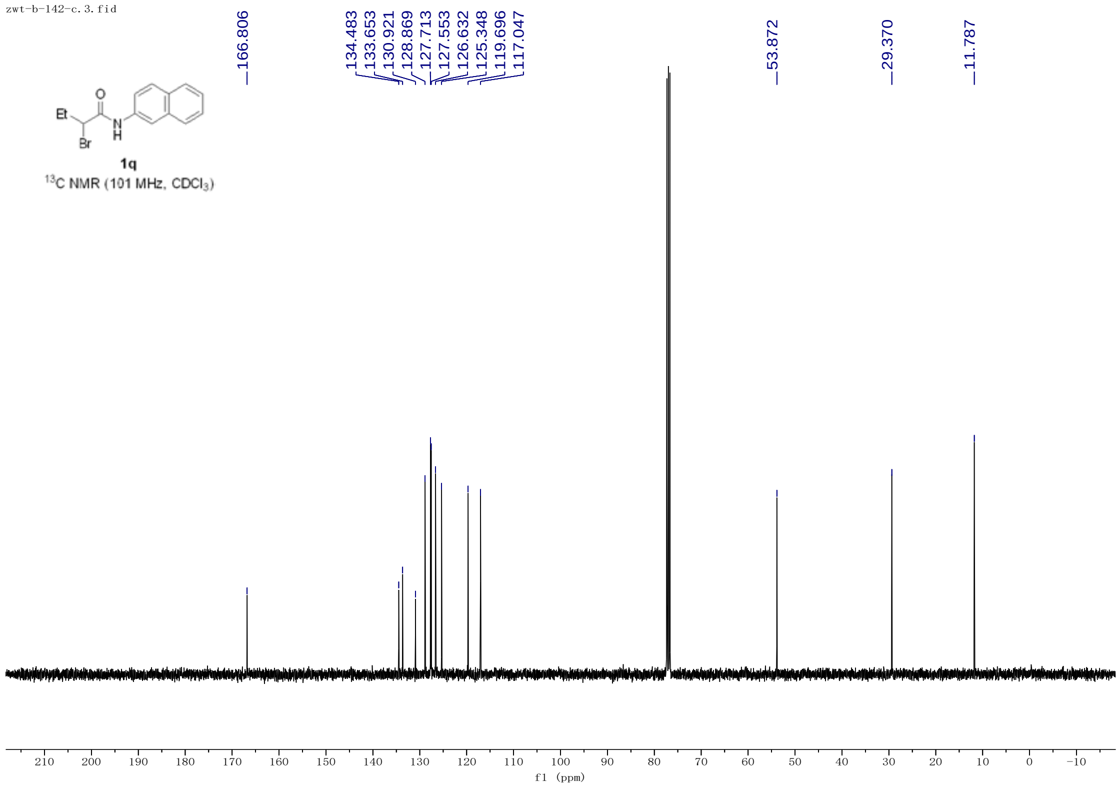

Supplementary Figure 77 ^{1}H-NMR (400 Mz, $CHCl_3$, 25 °C) and ^{13}C-NMR (101 MHz, $CHCl_3$, 25 °C) spectra of **1q**

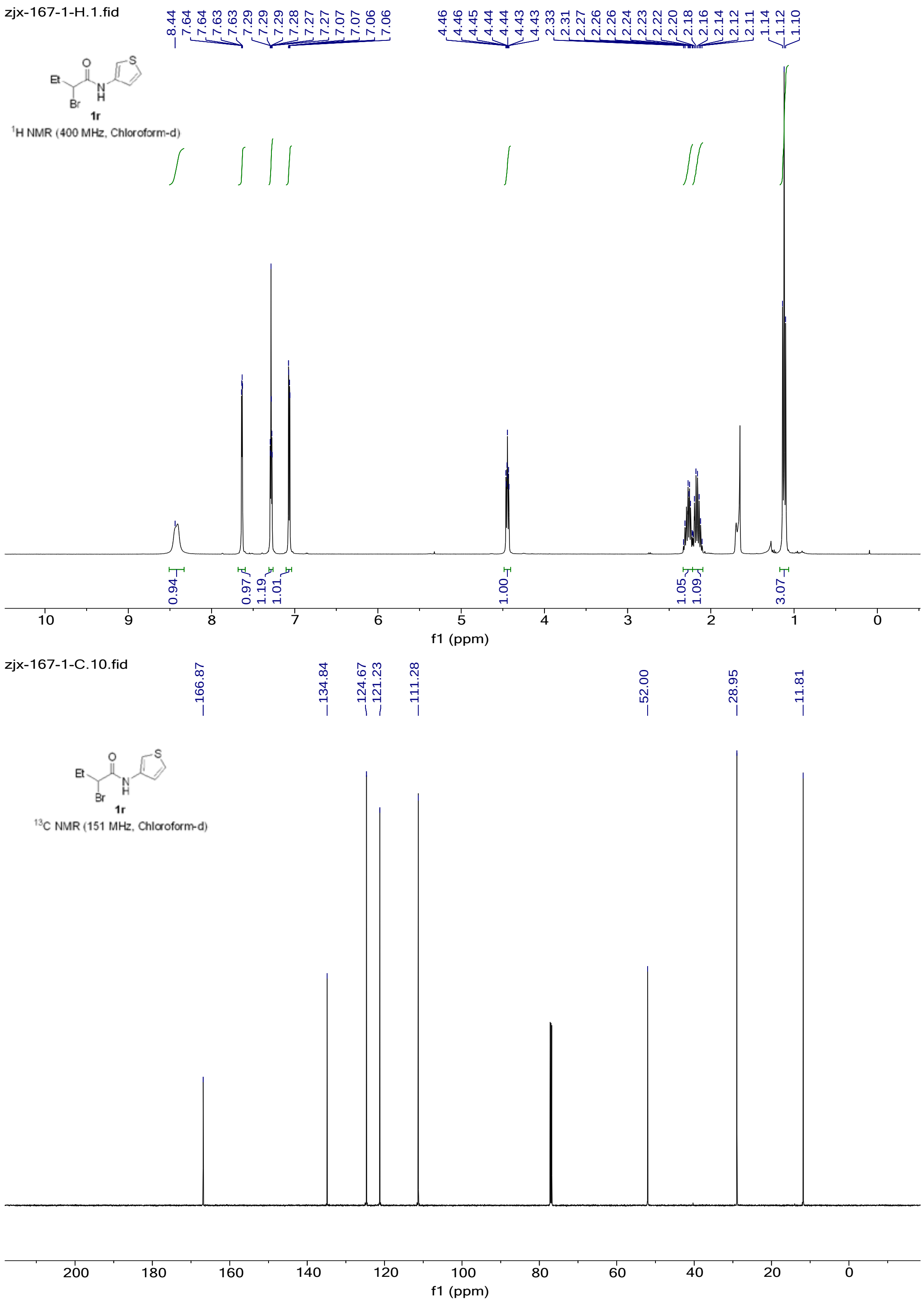

Supplementary Figure 78 ^{1}H-NMR (400 Mz, $CHCl_3$, 25 °C) and ^{13}C-NMR (151 MHz, $CHCl_3$, 25 °C) spectra of **1r**

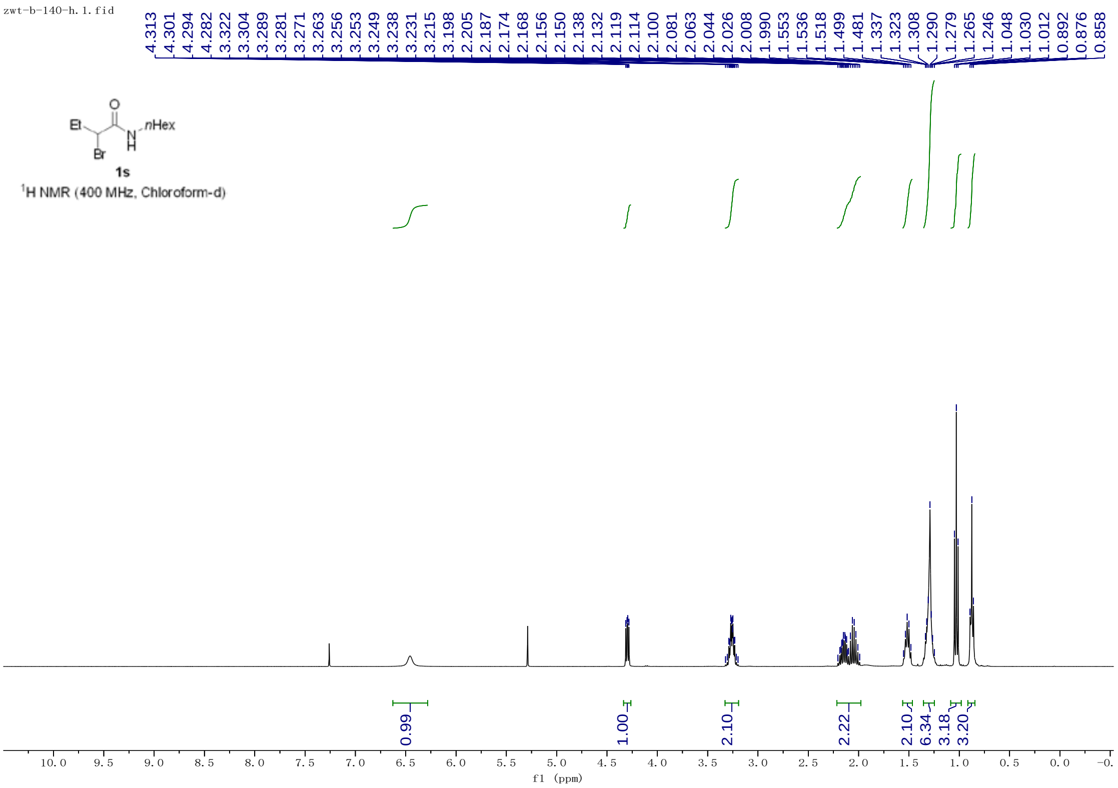

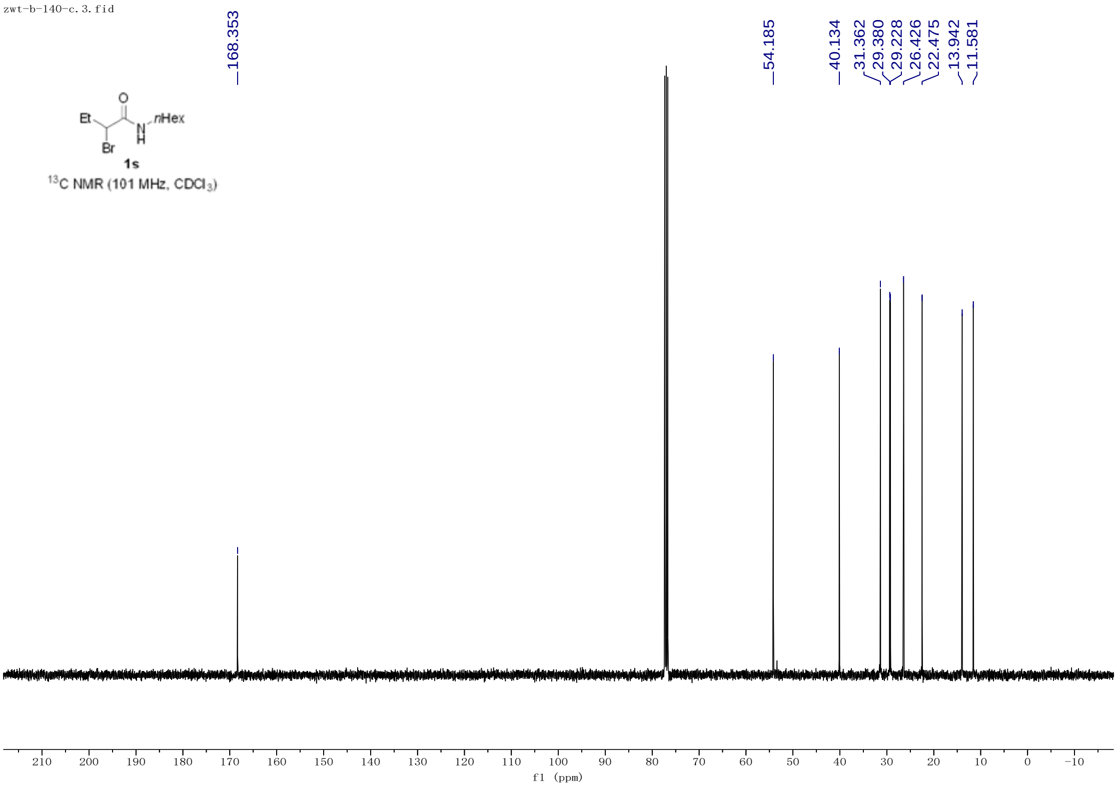

Supplementary Figure 79 ^{1}H-NMR (400 Mz, $CHCl_3$, 25 °C) and ^{13}C-NMR (101 MHz, $CHCl_3$, 25 °C) spectra of **1s**

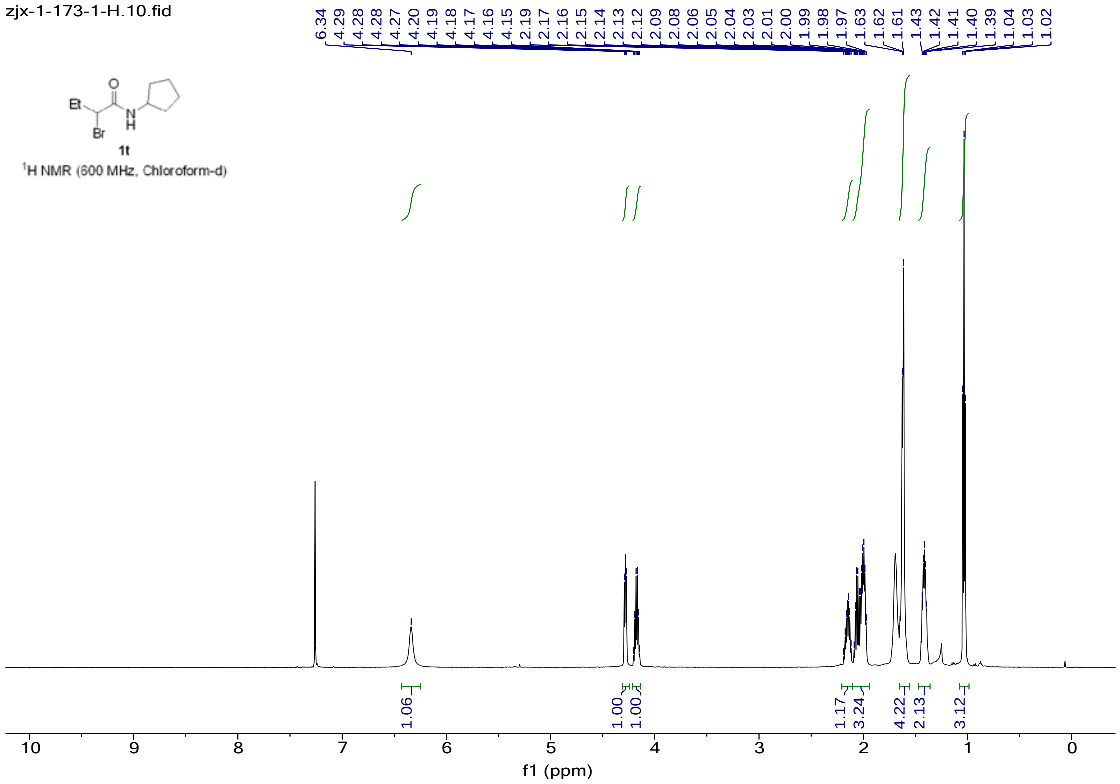

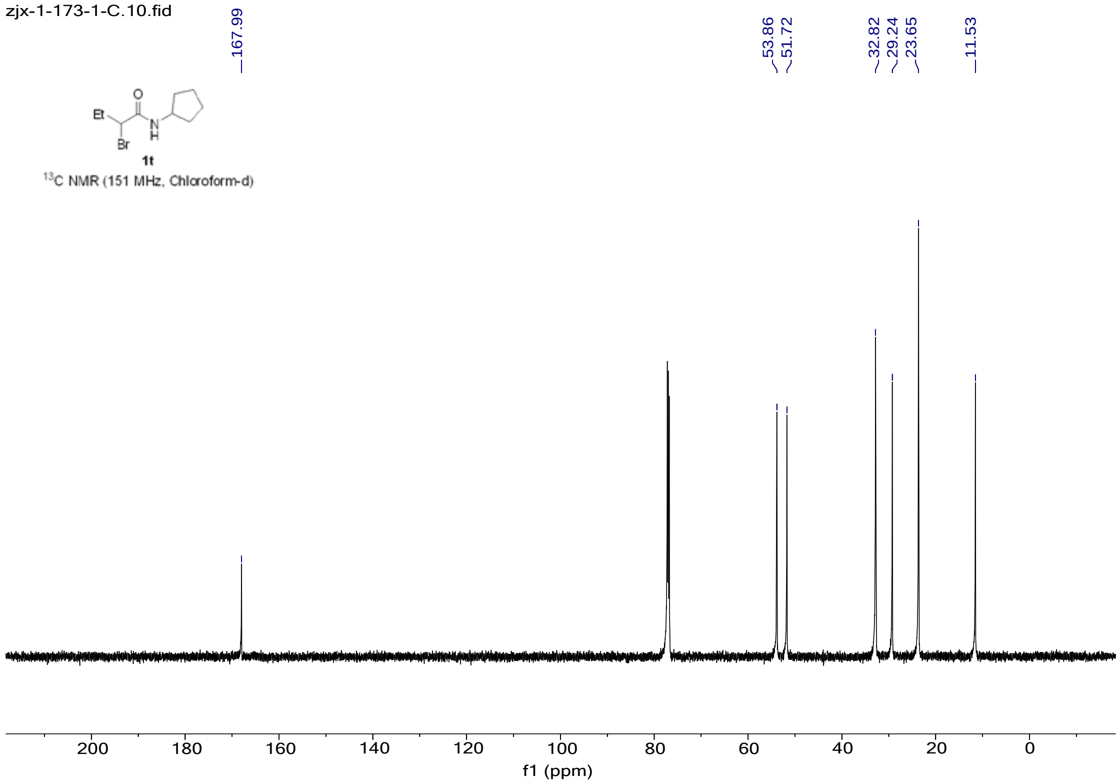

Supplementary Figure 80 ^{1}H-NMR (600 Mz, $CHCl_3$, 25 °C) and ^{13}C-NMR (151 MHz, $CHCl_3$, 25 °C) spectra of **1t**

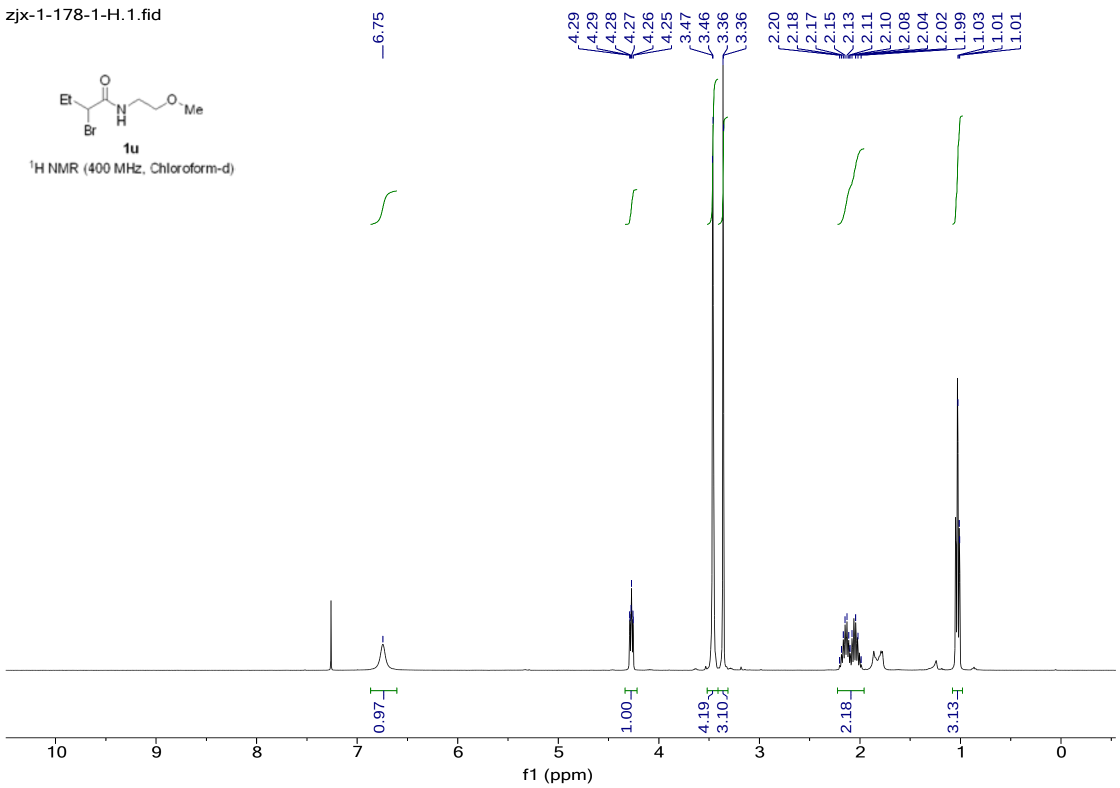

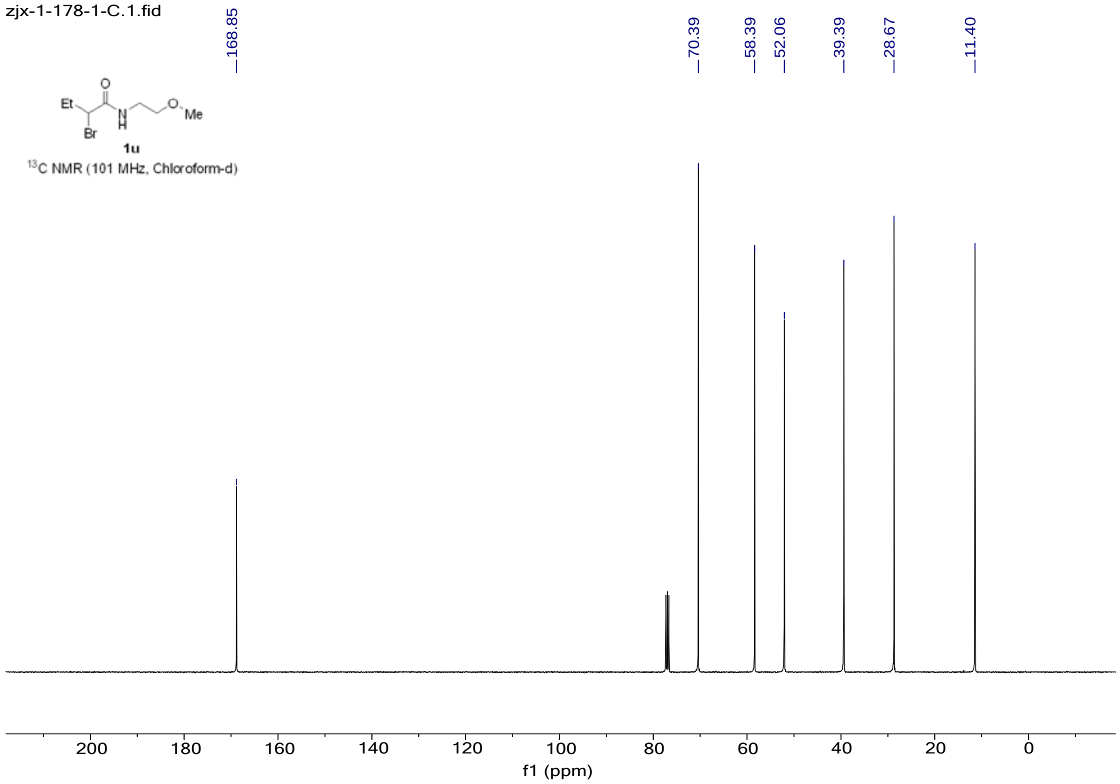

Supplementary Figure 81 ^{1}H-NMR (400 Mz, $CHCl_3$, 25 °C) and ^{13}C-NMR (101 MHz, $CHCl_3$, 25 °C) spectra of **1u**

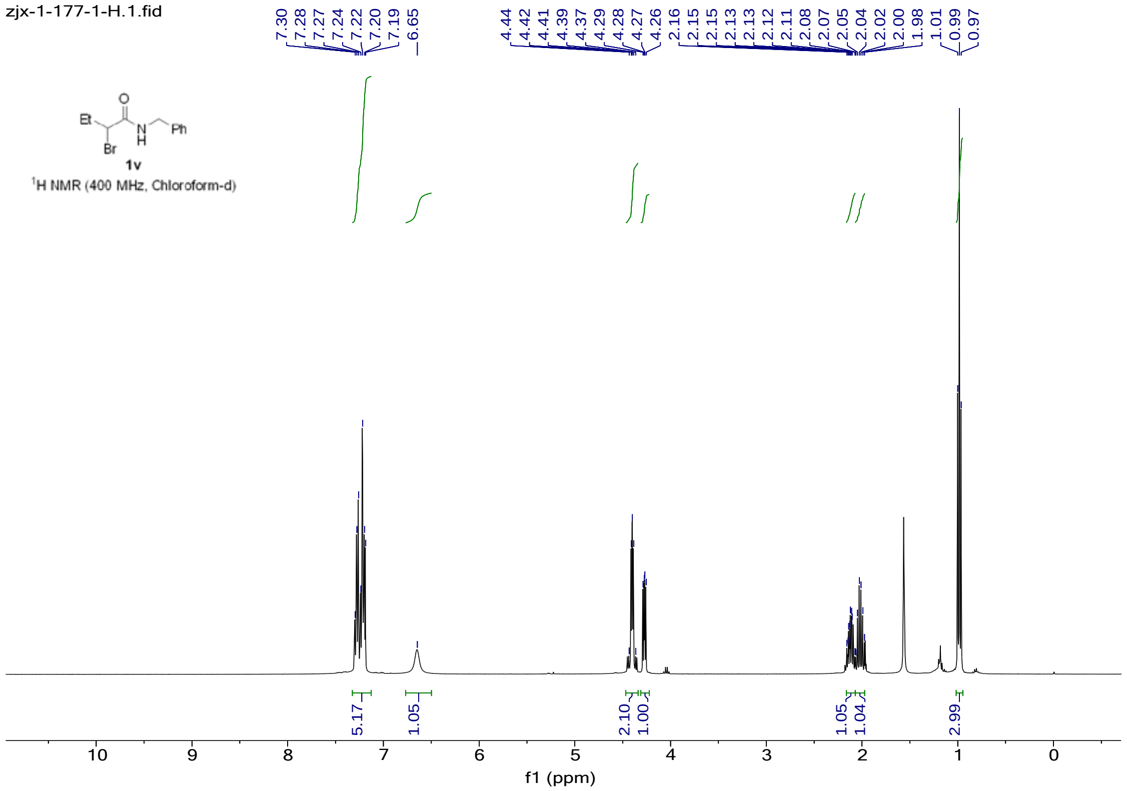

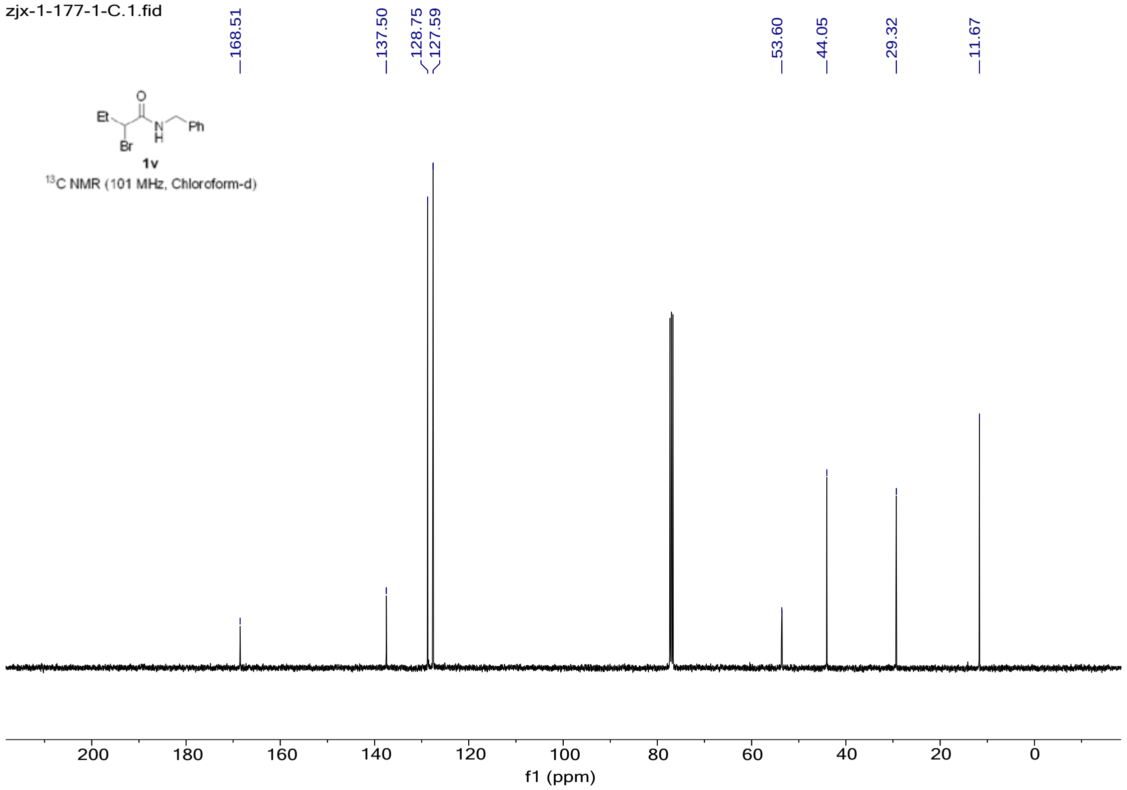

Supplementary Figure 82 ^{1}H-NMR (400 Mz, $CHCl_3$, 25 °C) and ^{13}C-NMR (101 MHz, $CHCl_3$, 25 °C) spectra of **1v**

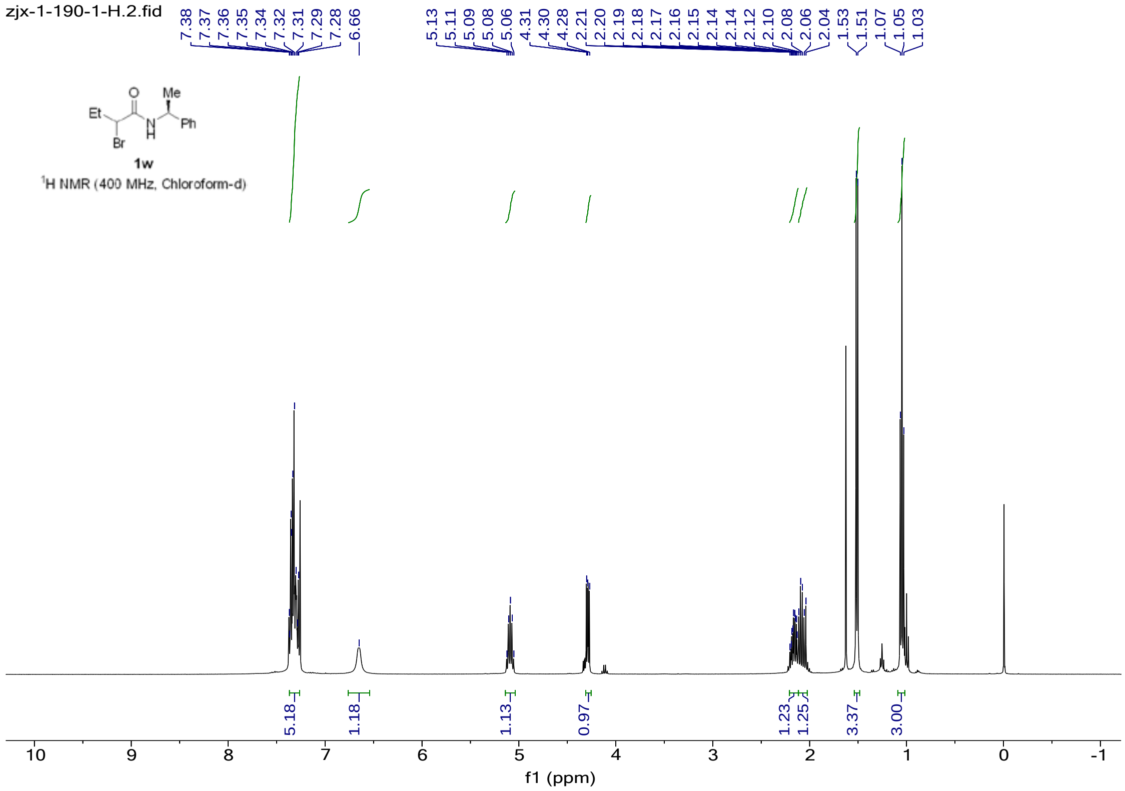

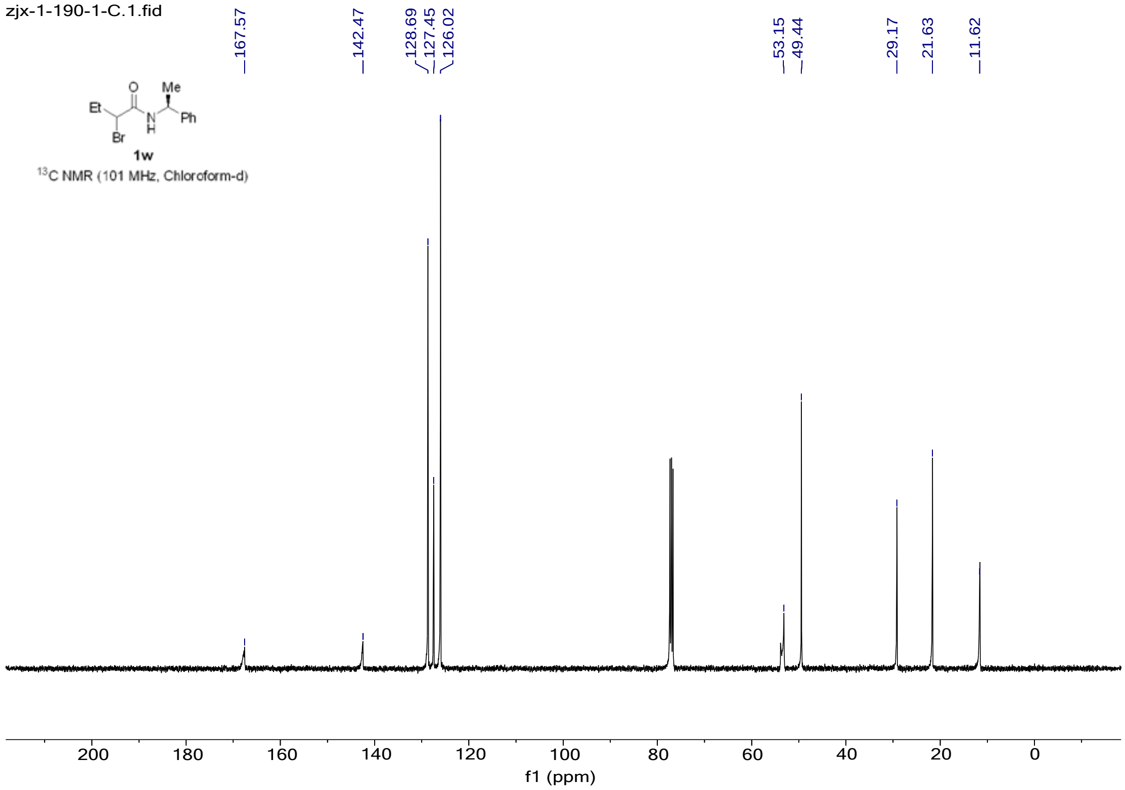

Supplementary Figure 83 ^{1}H-NMR (400 Mz, $CHCl_3$, 25 °C) and ^{13}C-NMR (101 MHz, $CHCl_3$, 25 °C) spectra of **1w**

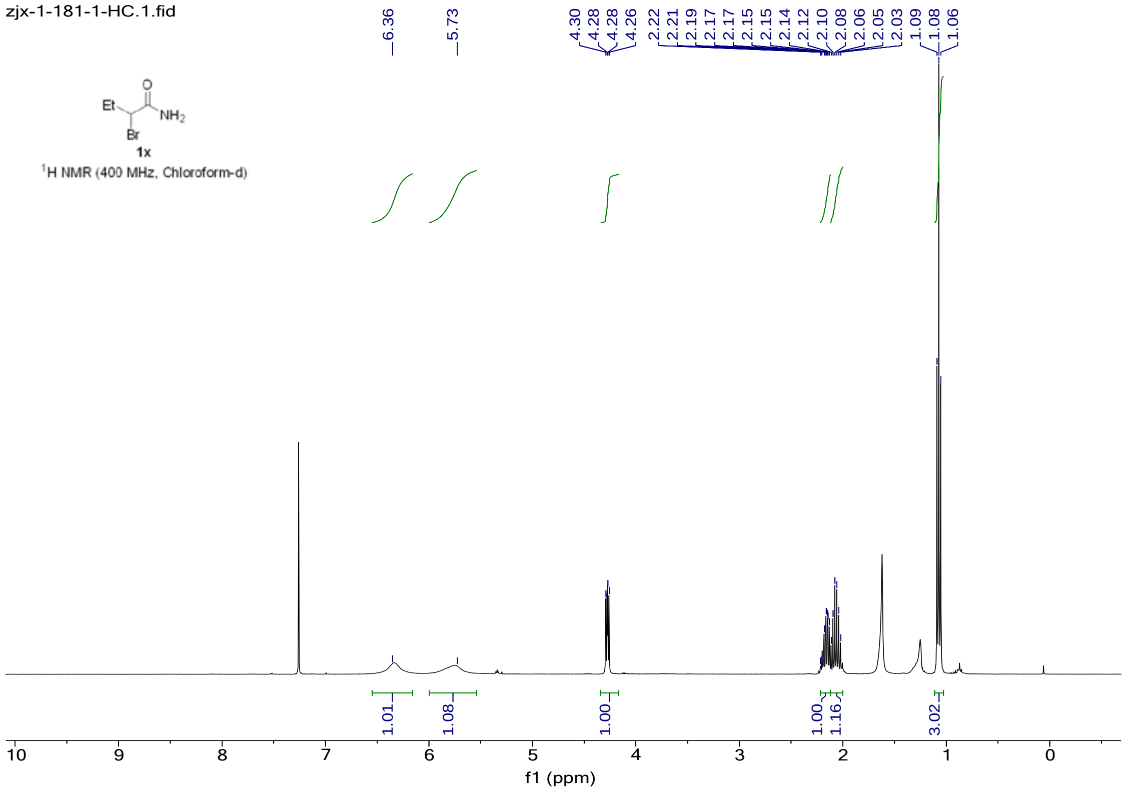

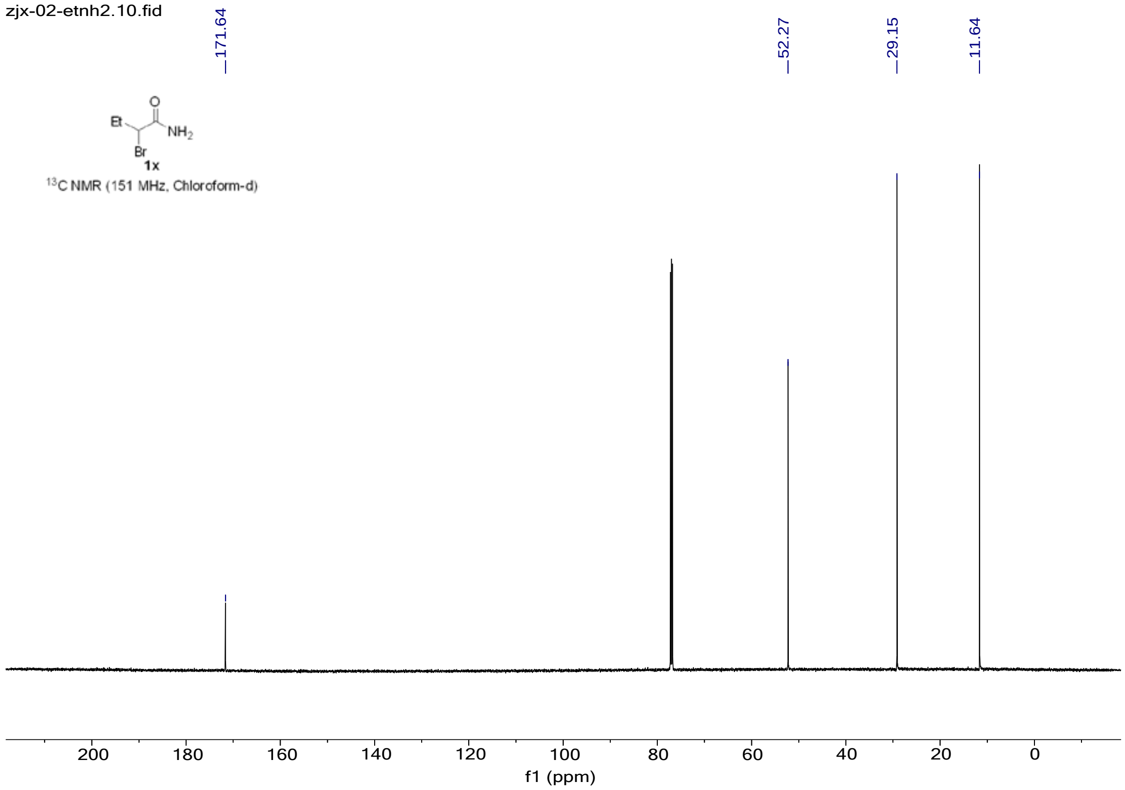

Supplementary Figure 84 ^{1}H-NMR (400 Mz, $CHCl_3$, 25 °C) and ^{13}C-NMR (151 MHz, $CHCl_3$, 25 °C) spectra of **1x**

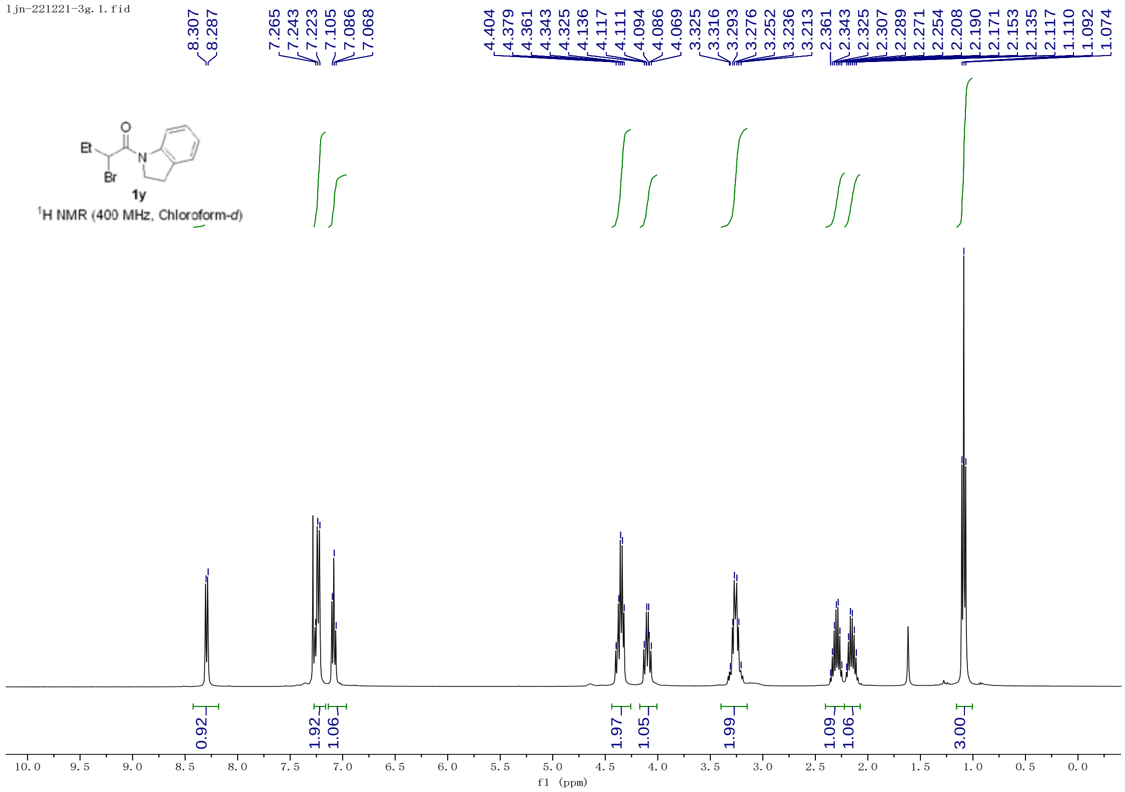

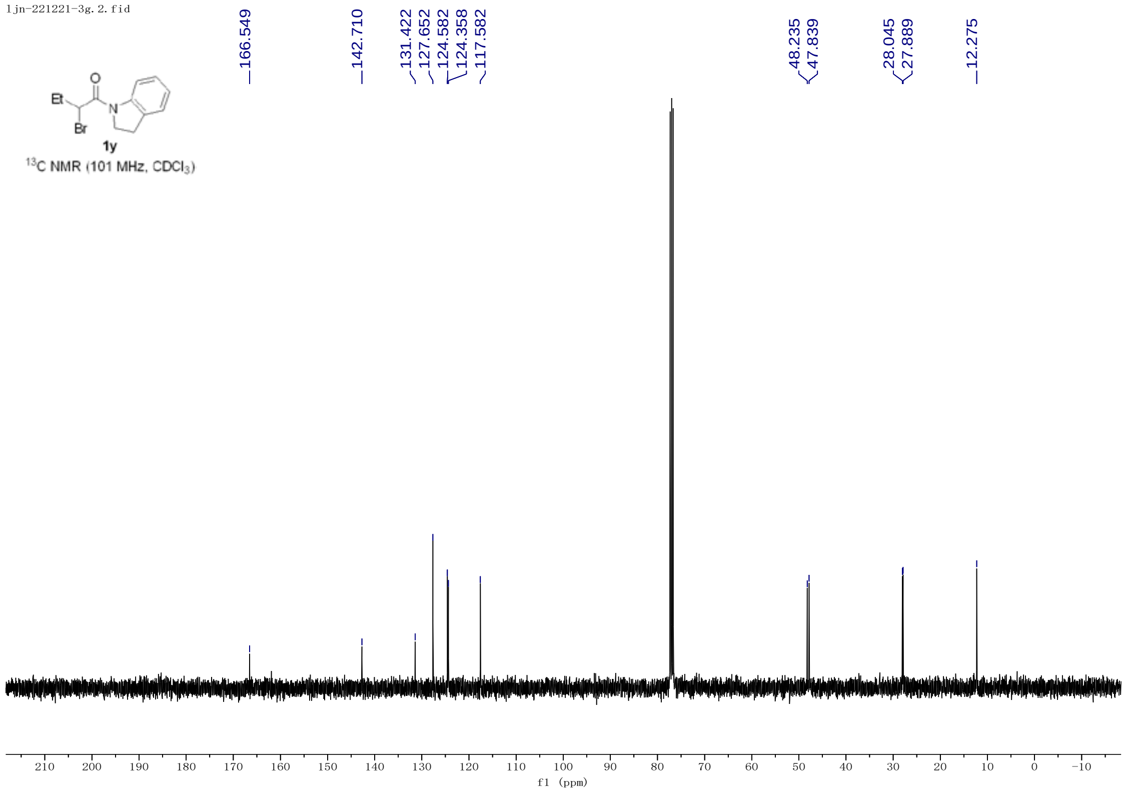

Supplementary Figure 85 ^{1}H-NMR (400 Mz, $CHCl_3$, 25 °C) and ^{13}C-NMR (101 MHz, $CHCl_3$, 25 °C) spectra of **1y**

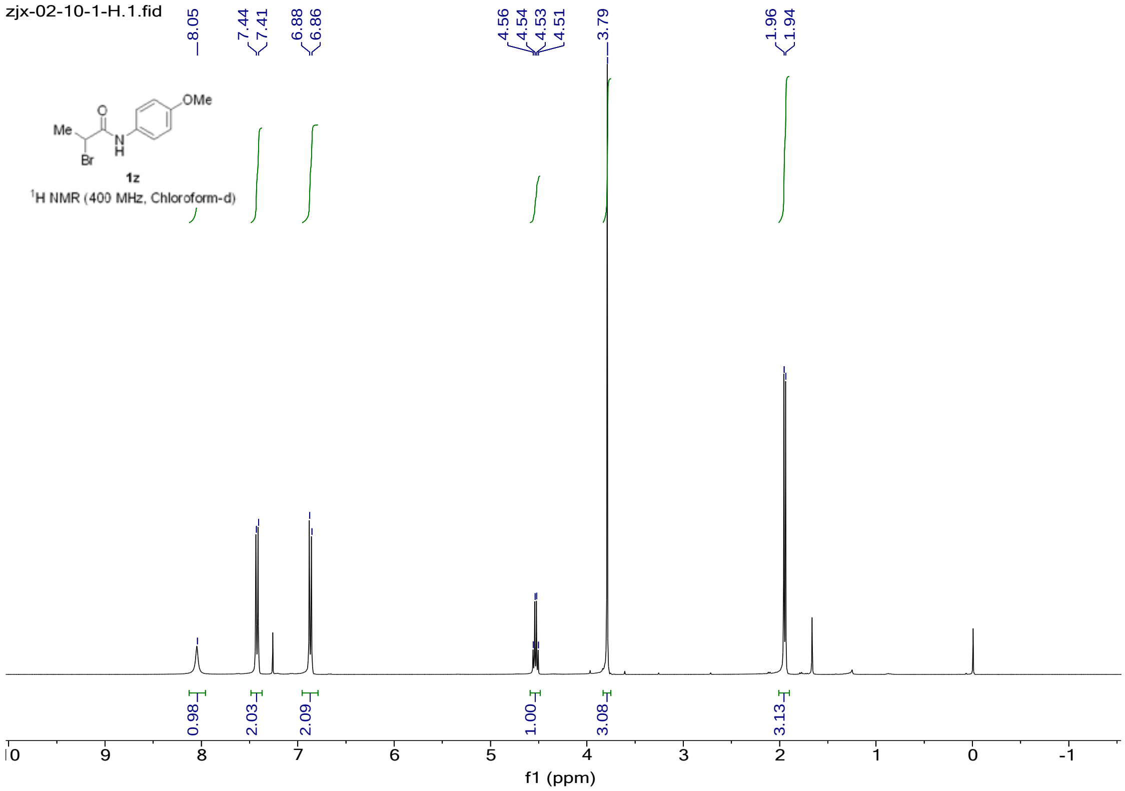

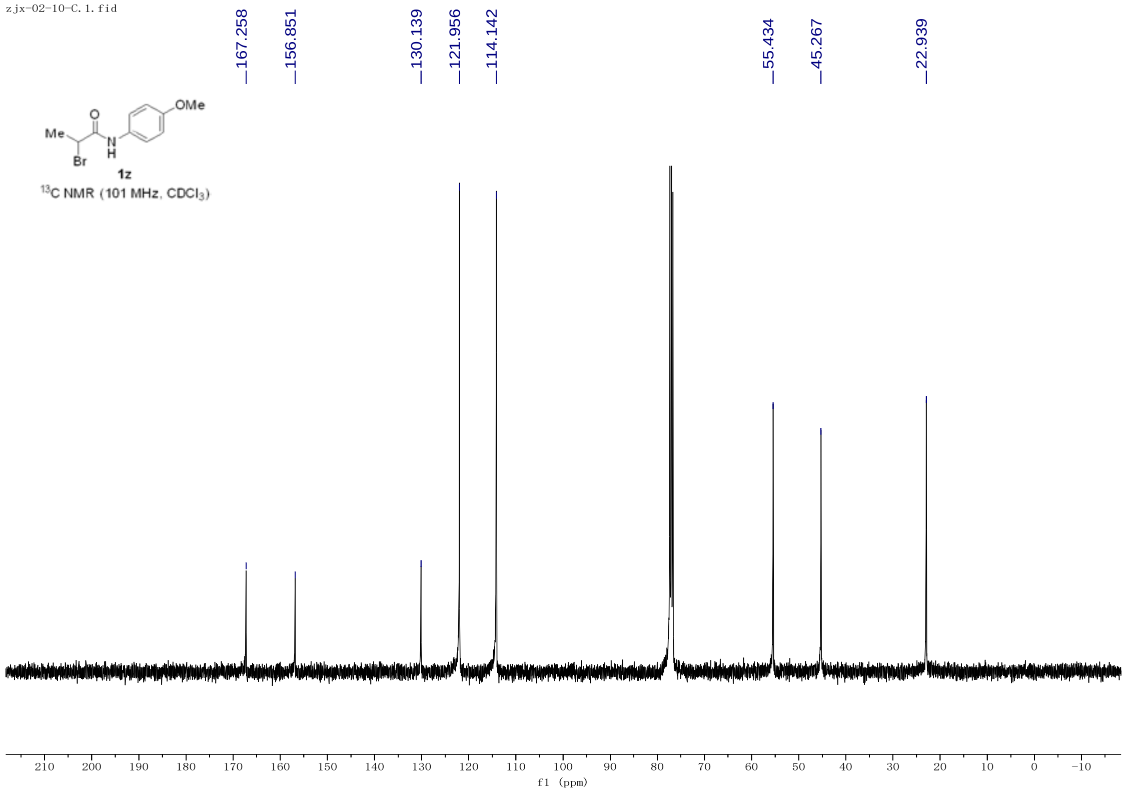

Supplementary Figure 86 ^{1}H-NMR (400 Mz, $CHCl_3$, 25 °C) and ^{13}C-NMR (101 MHz, $CHCl_3$, 25 °C) spectra of **1z**

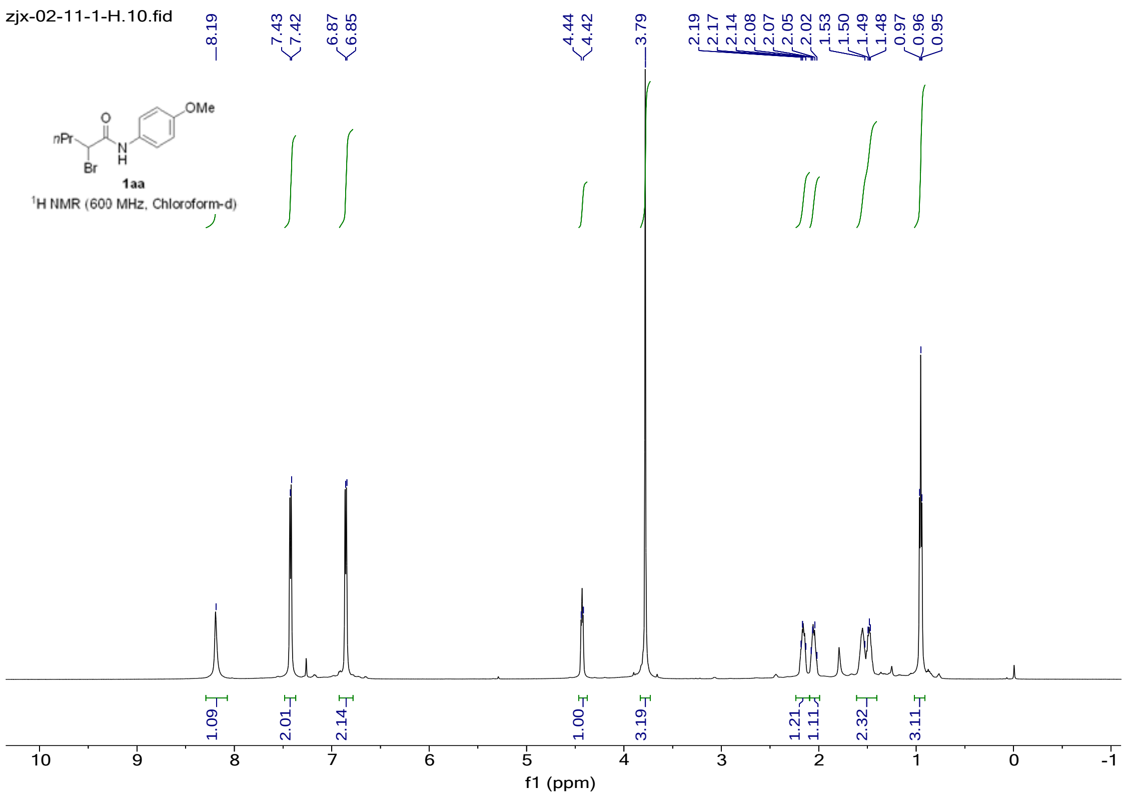

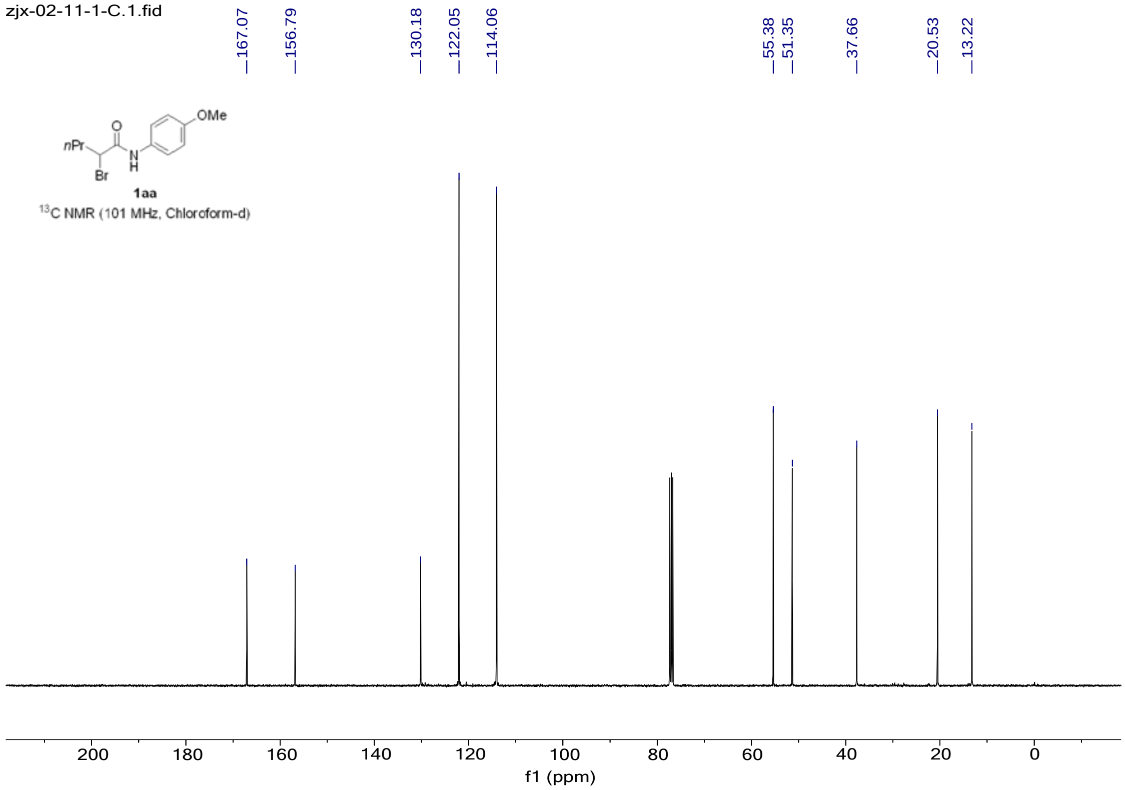

Supplementary Figure 87 ^{1}H-NMR (600 Mz, $CHCl_3$, 25 °C) and ^{13}C-NMR (101 MHz, $CHCl_3$, 25 °C) spectra of **1aa**

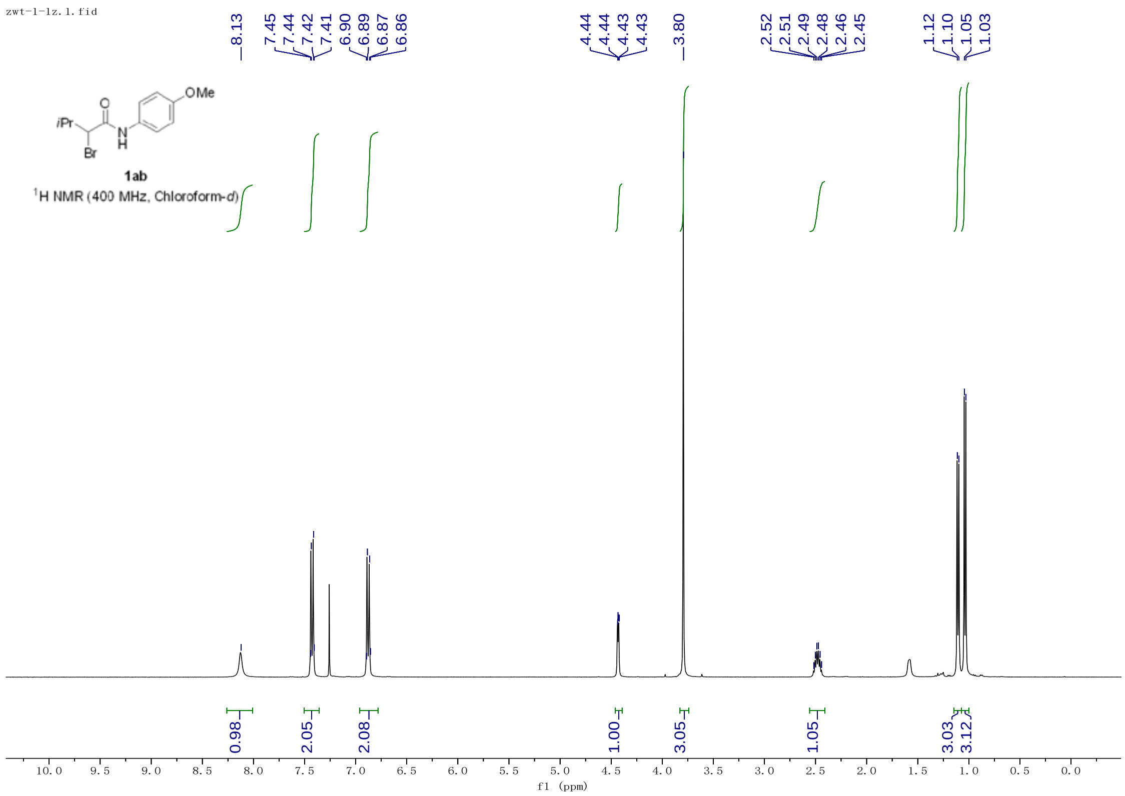

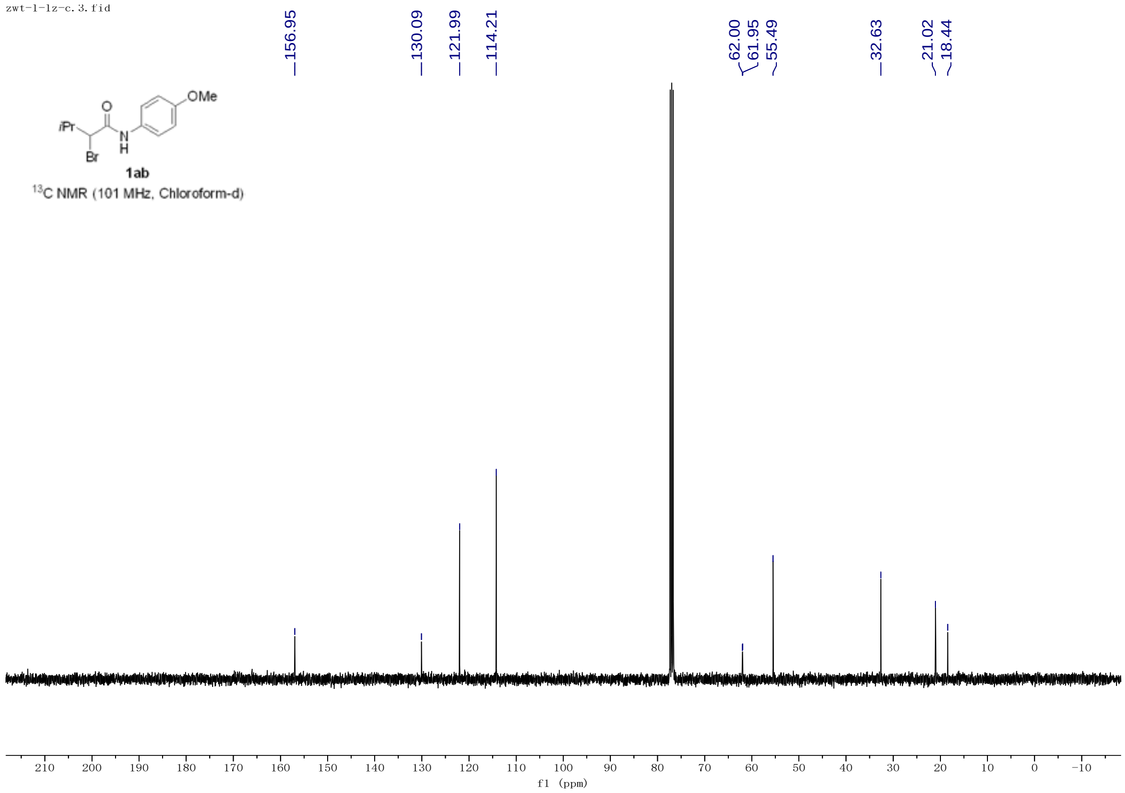

Supplementary Figure 88 ^{1}H-NMR (400 Mz, $CHCl_3$, 25 °C) and ^{13}C-NMR (101 MHz, $CHCl_3$, 25 °C) spectra of **1ab**

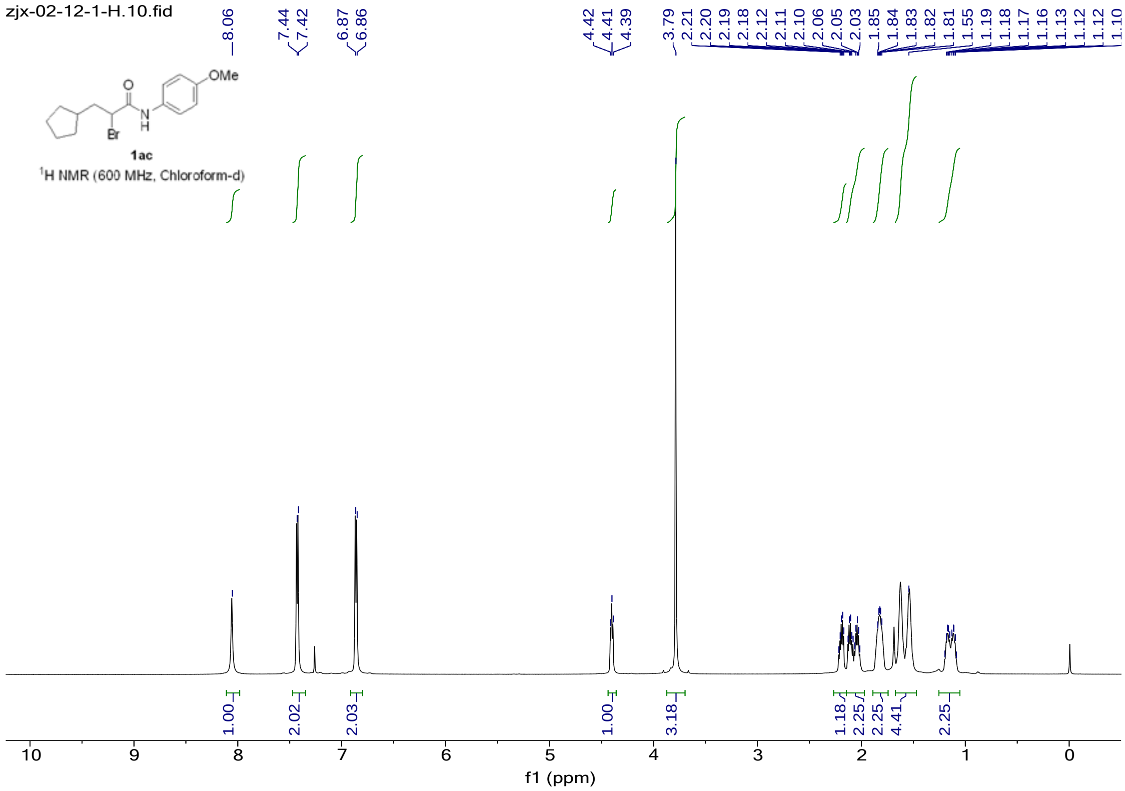

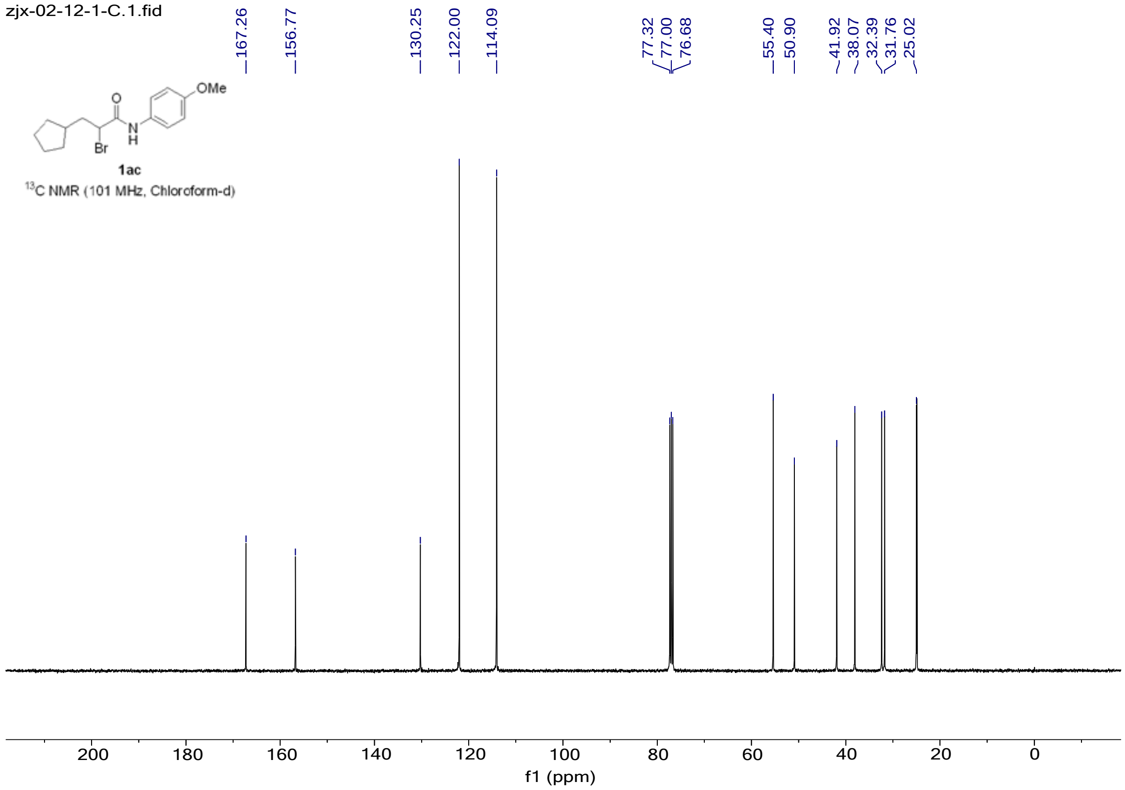

Supplementary Figure 89 ^{1}H-NMR (600 Mz, $CHCl_3$, 25 °C) and ^{13}C-NMR (101 MHz, $CHCl_3$, 25 °C) spectra of **1ac**

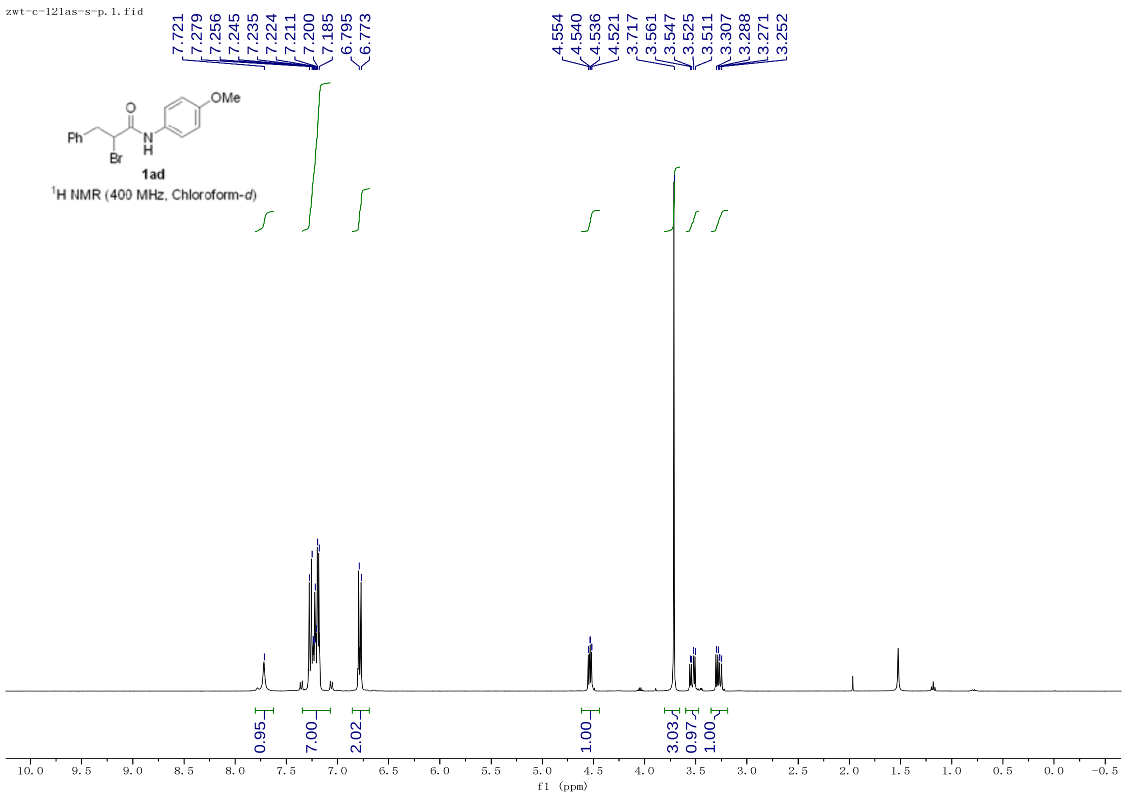

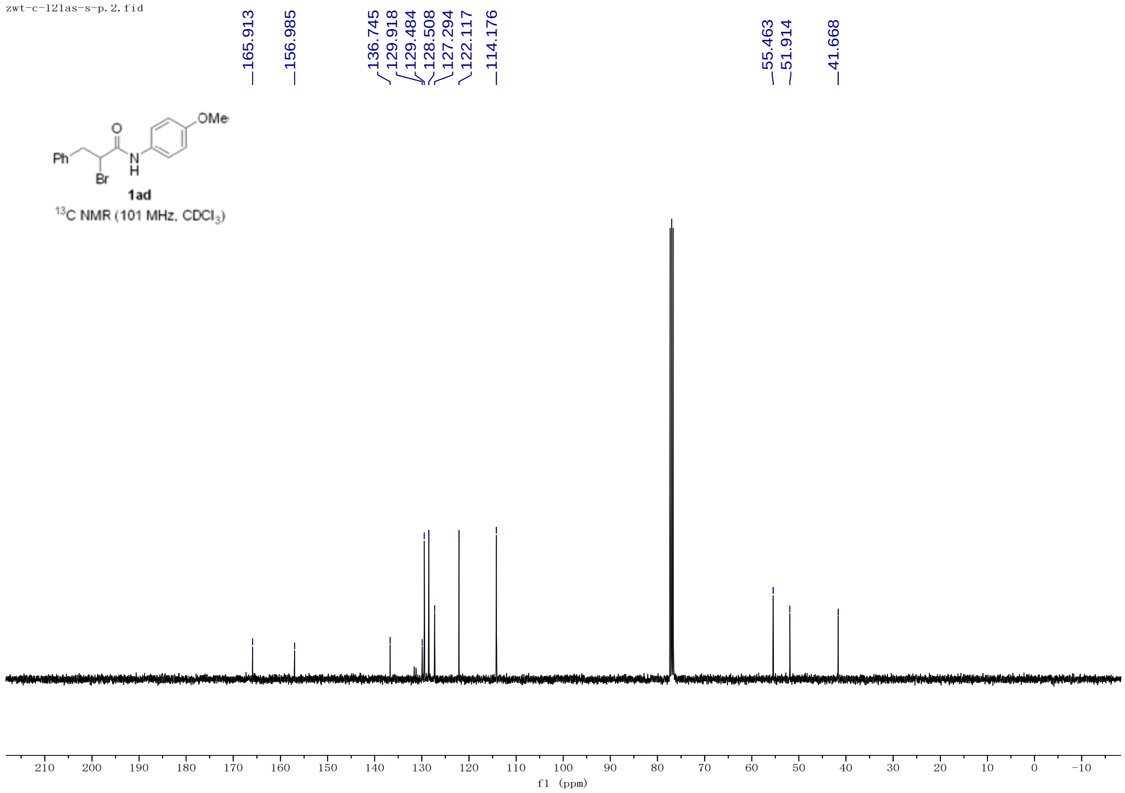

Supplementary Figure 90 ^{1}H-NMR (400 Mz, $CHCl_3$, 25 °C) and ^{13}C-NMR (101 MHz, $CHCl_3$, 25 °C) spectra of **1ad**

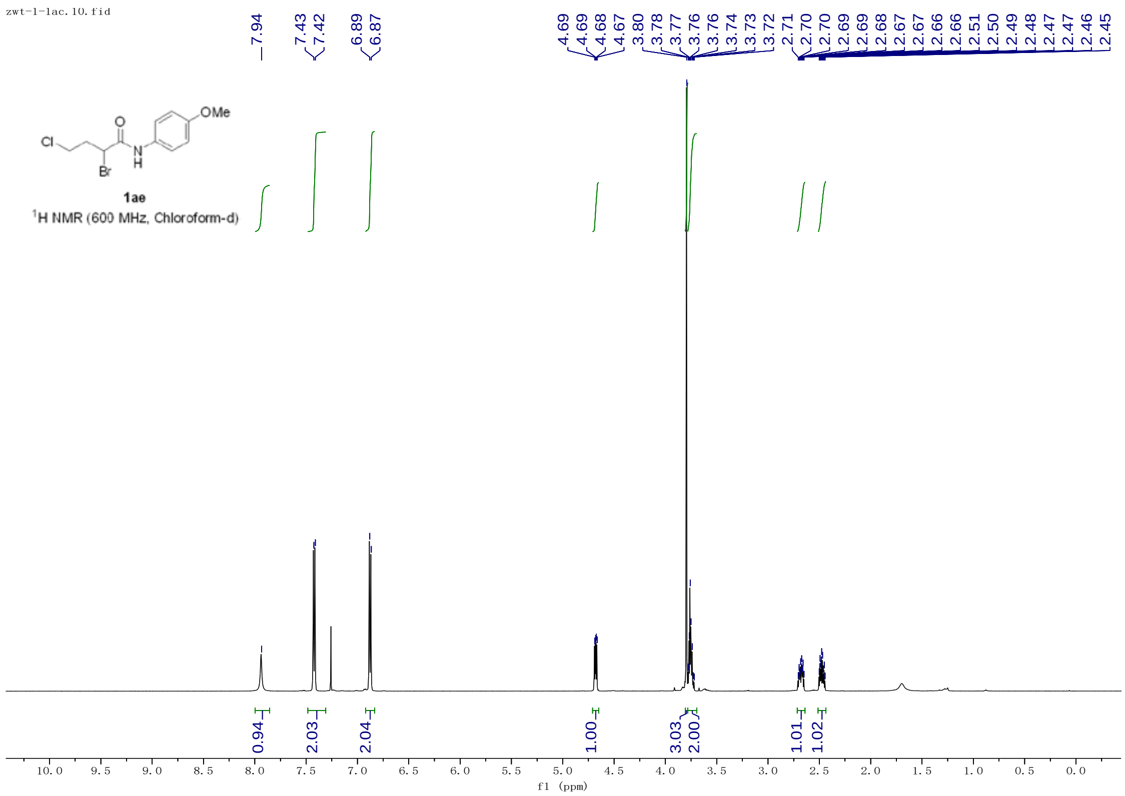

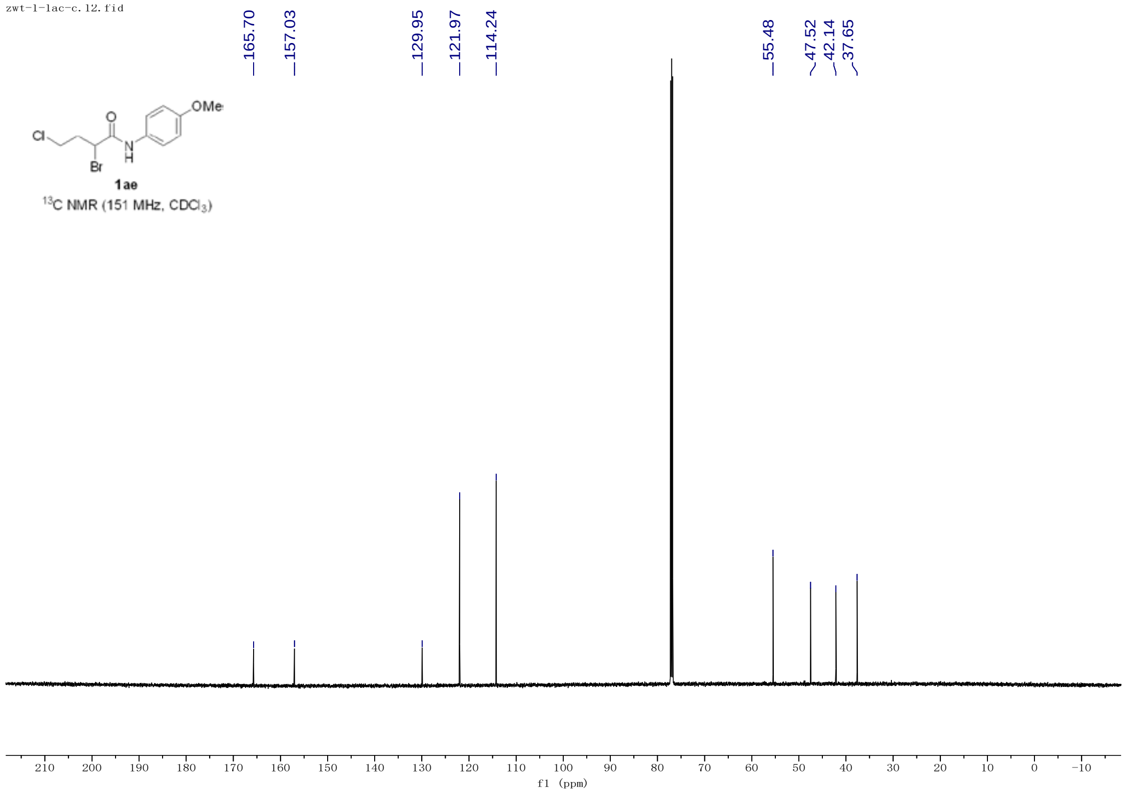

Supplementary Figure 91 ^{1}H-NMR (600 Mz, $CHCl_3$, 25 °C) and ^{13}C-NMR (151 MHz, $CHCl_3$, 25 °C) spectra of **1ae**

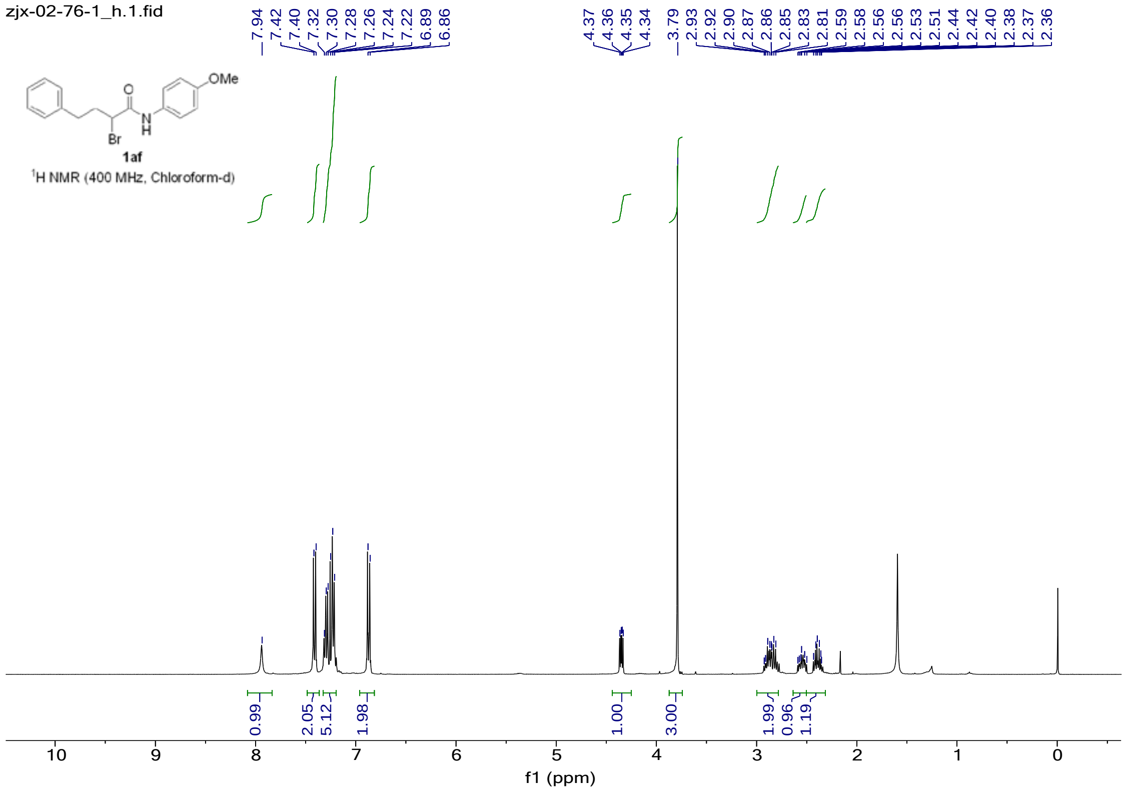

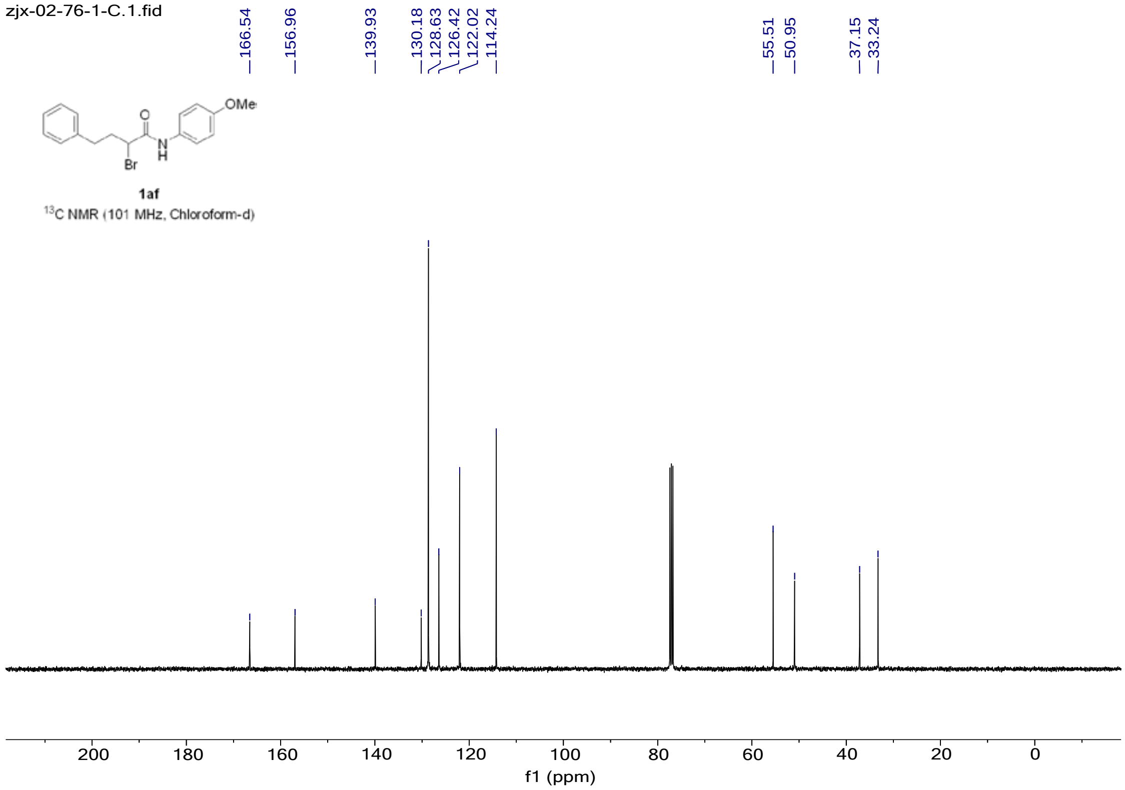

Supplementary Figure 92 ^{1}H-NMR (400 Mz, $CHCl_3$, 25 °C) and ^{13}C-NMR (101 MHz, $CHCl_3$, 25 °C) spectra of **1af**

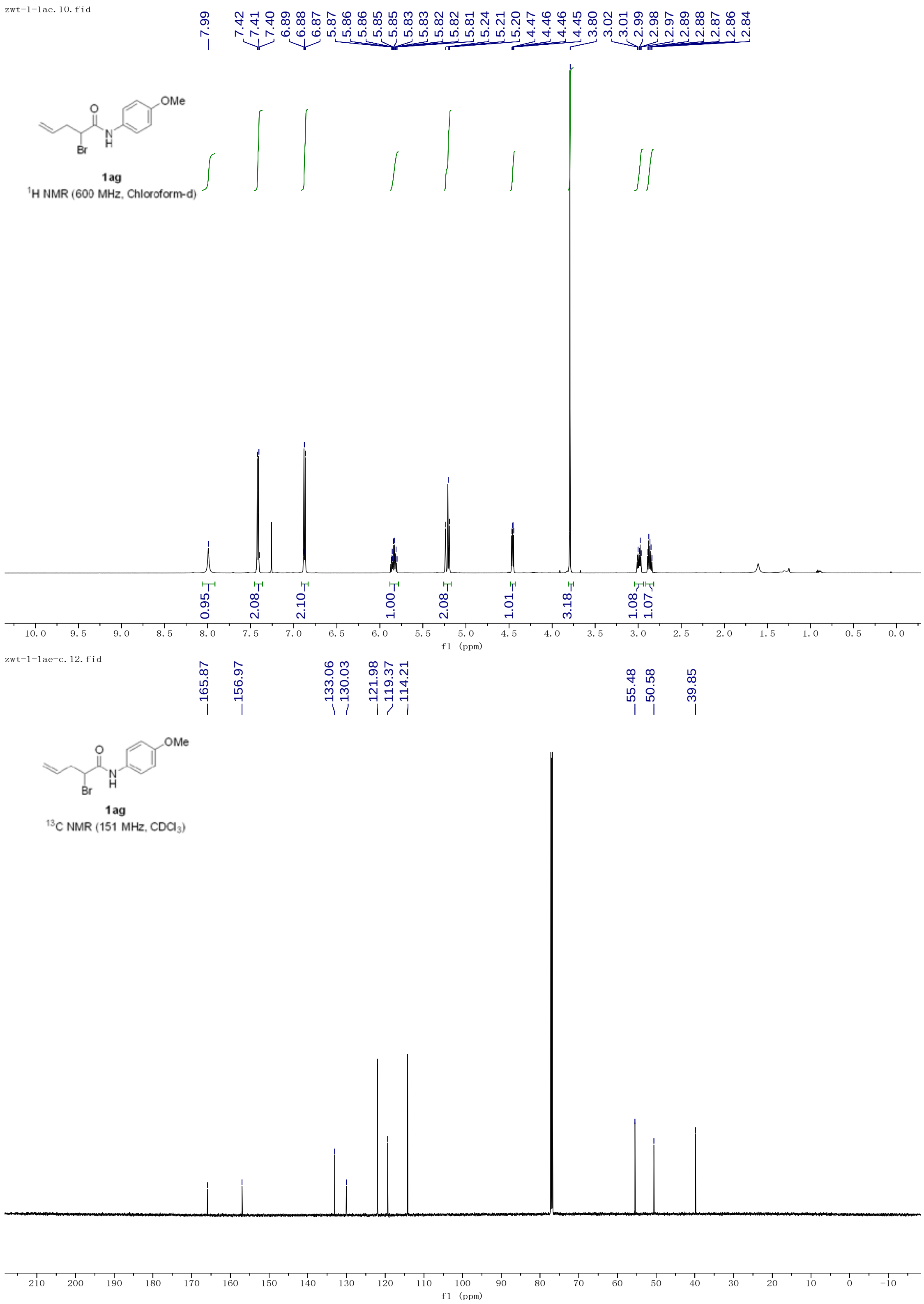

Supplementary Figure 93 ^{1}H-NMR (600 Mz, $CHCl_3$, 25 °C) and ^{13}C-NMR (151 MHz, $CHCl_3$, 25 °C) spectra of **1ag**

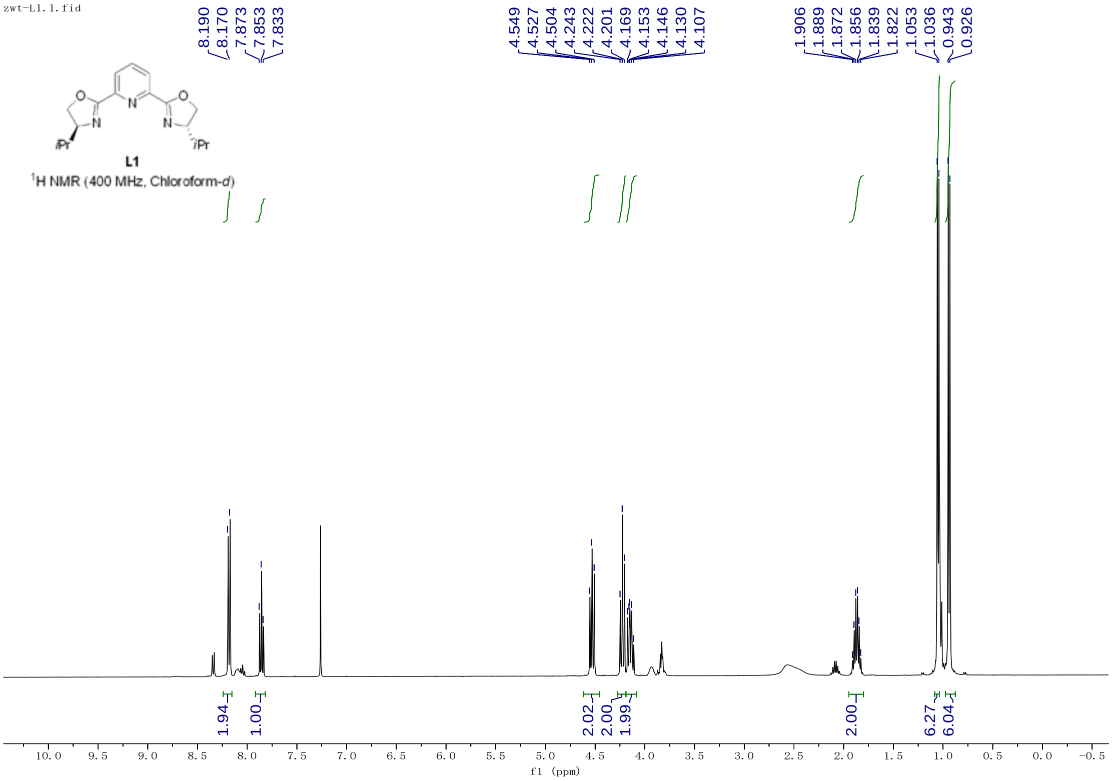

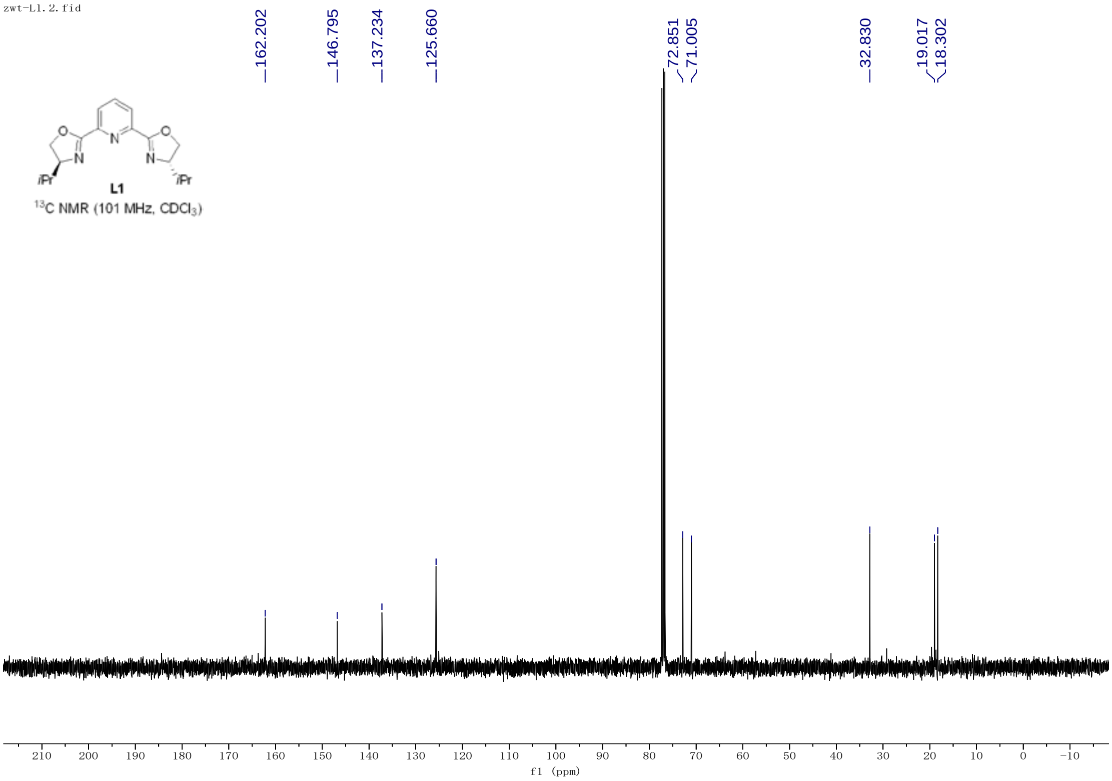

Supplementary Figure 94 ^{1}H-NMR (400 Mz, $CHCl_3$, 25 °C) and ^{13}C-NMR (101 MHz, $CHCl_3$, 25 °C) spectra of **L1**

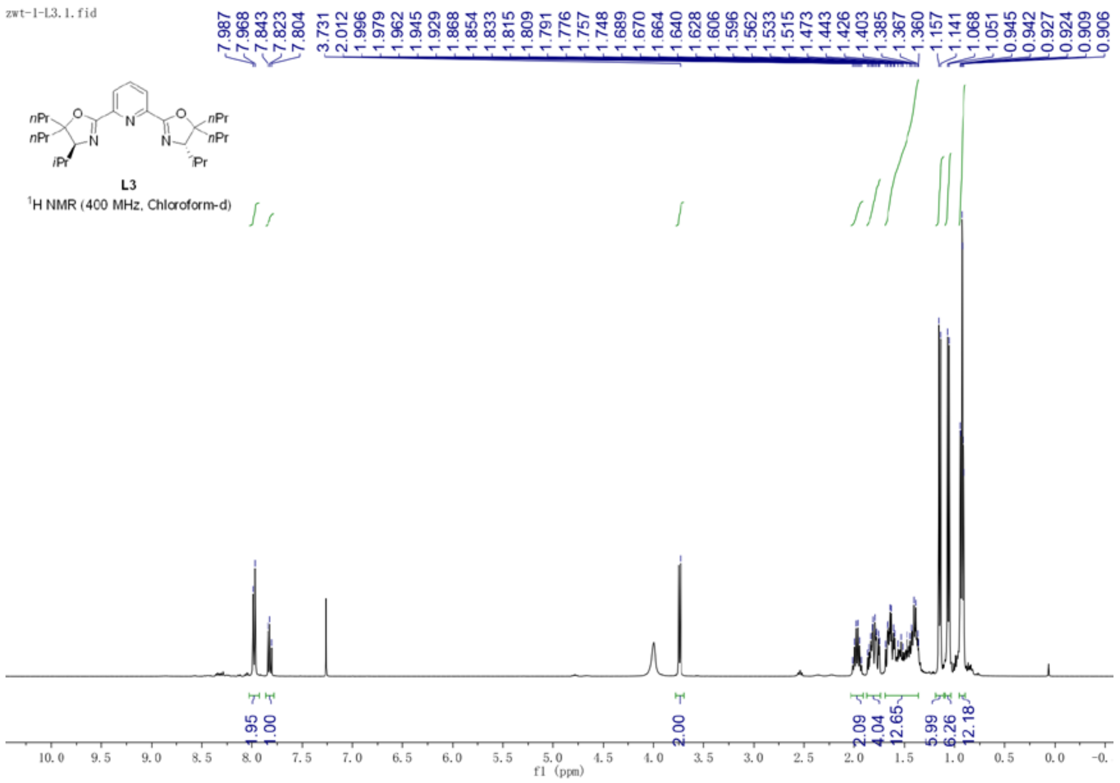

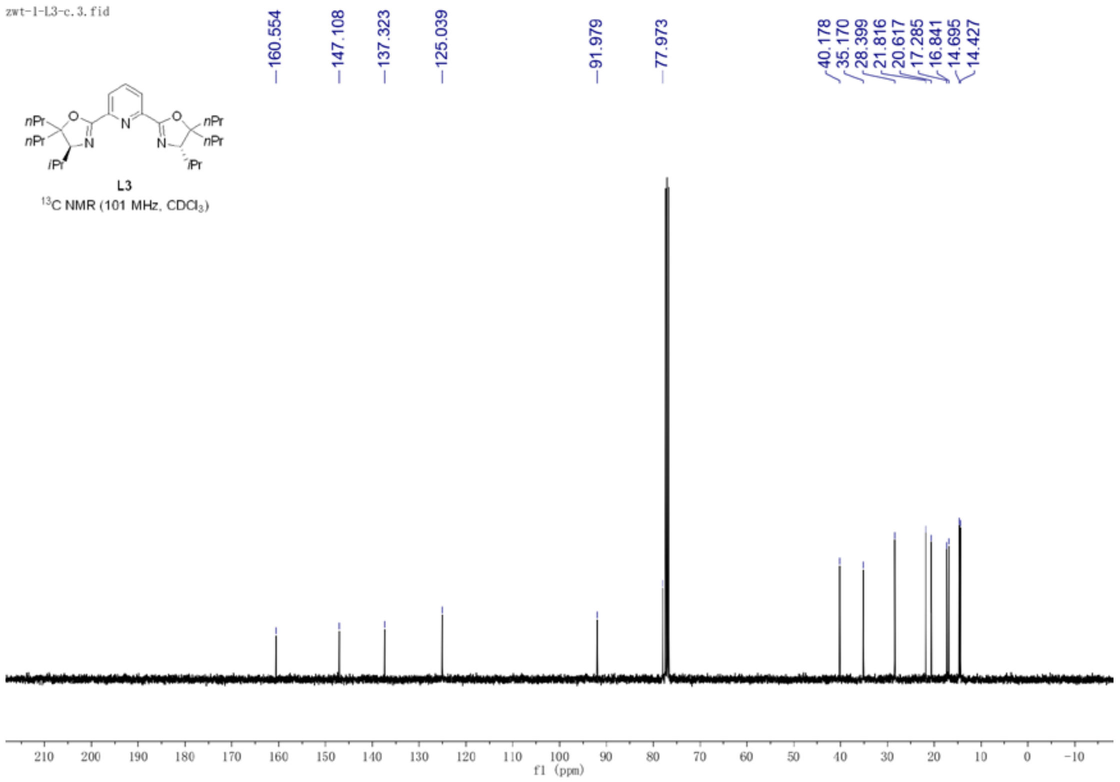

Supplementary Figure 95 ^{1}H-NMR (400 Mz, $CHCl_3$, 25 °C) and ^{13}C-NMR (101 MHz, $CHCl_3$, 25 °C) spectra of **L3**

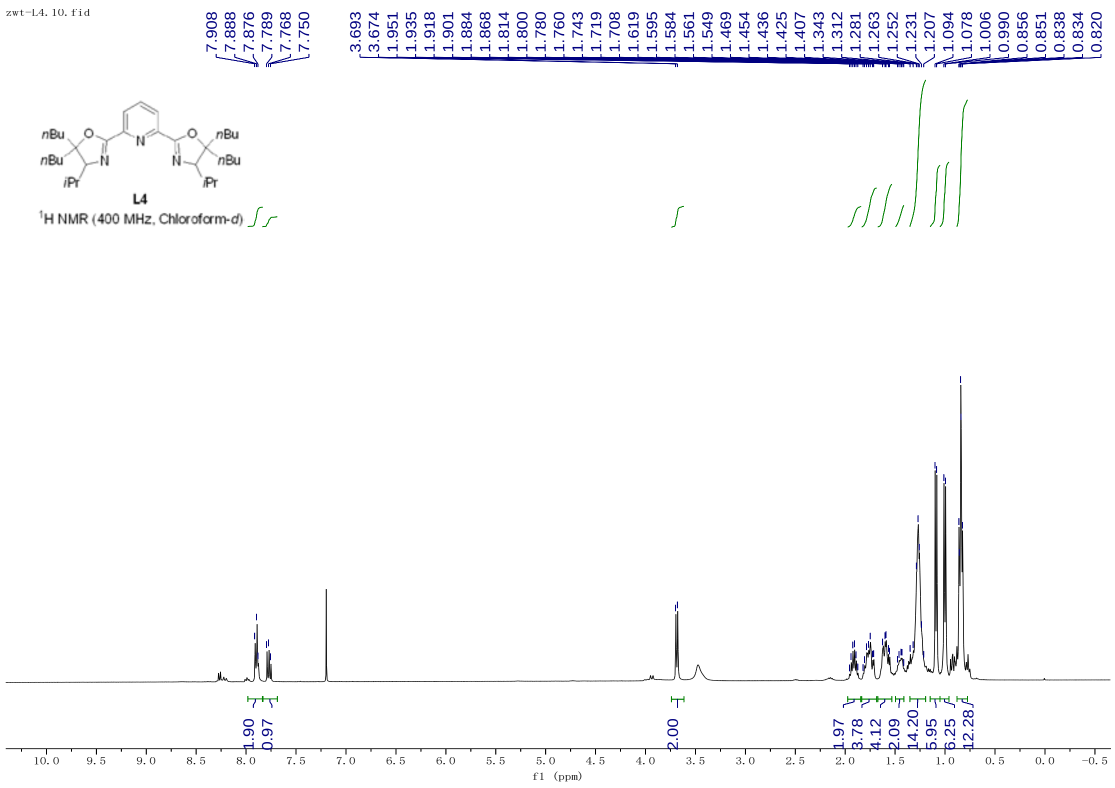

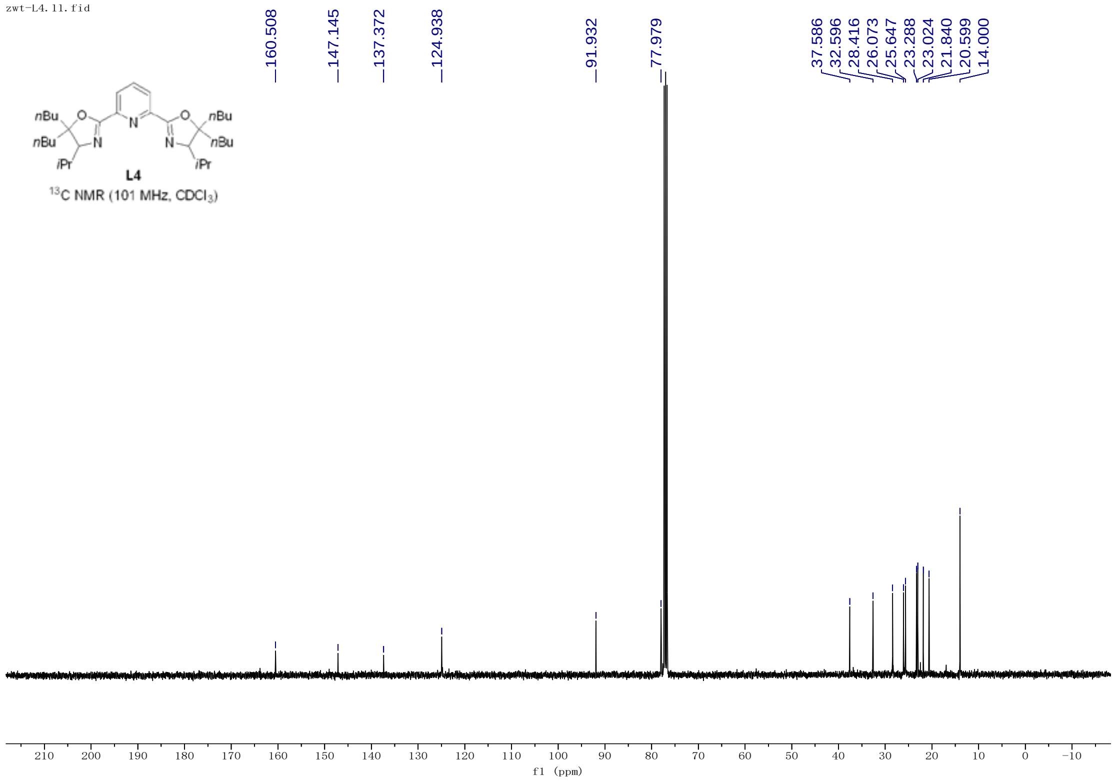

Supplementary Figure 96 ^{1}H-NMR (400 Mz, $CHCl_3$, 25 °C) and ^{13}C-NMR (101 MHz, $CHCl_3$, 25 °C) spectra of **L4**

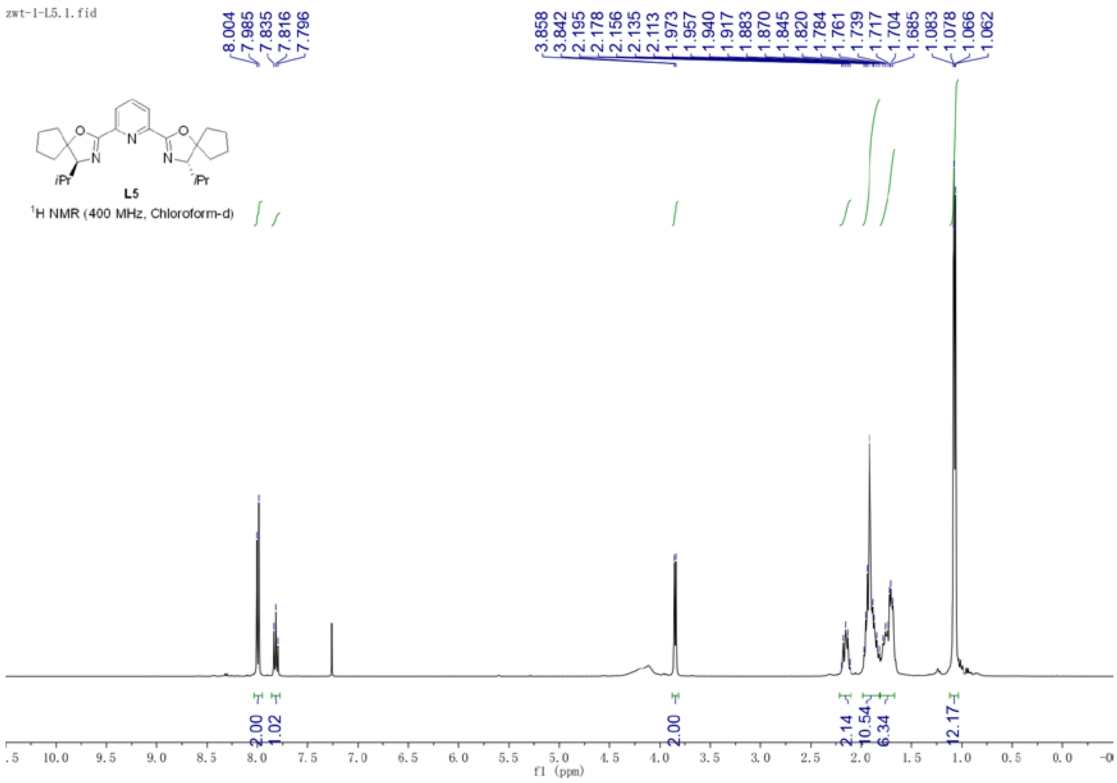

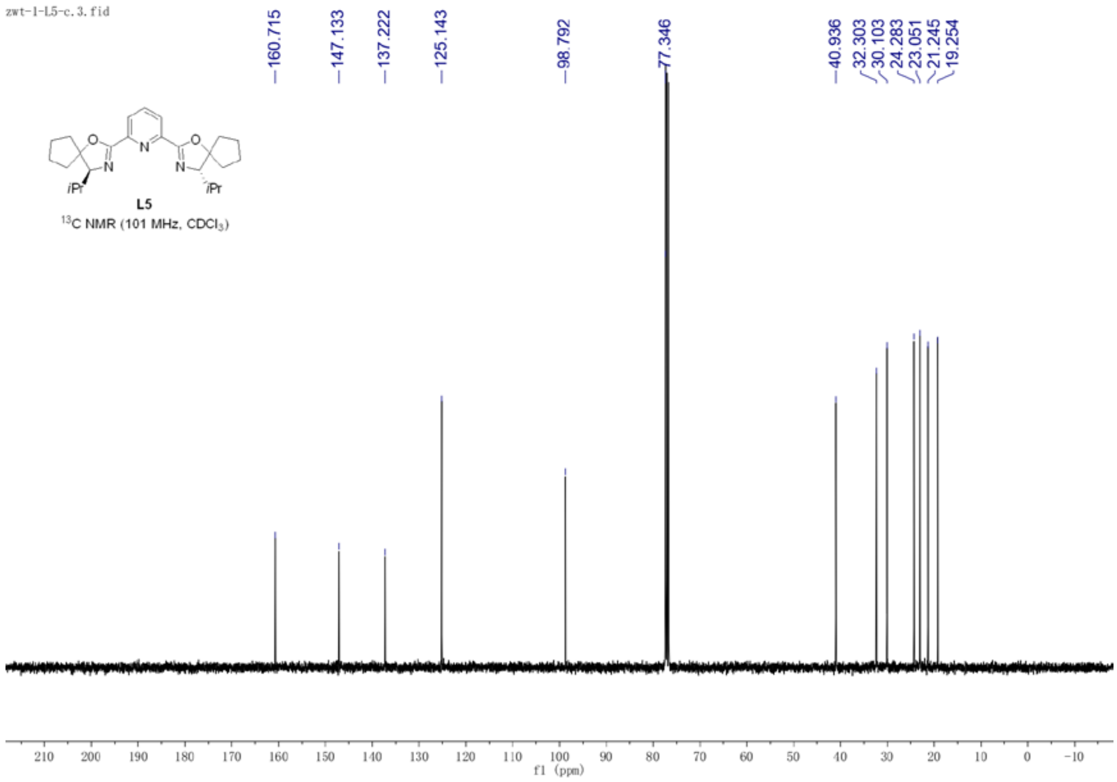

Supplementary Figure 97 ^{1}H-NMR (400 Mz, $CHCl_3$, 25 °C) and ^{13}C-NMR (101 MHz, $CHCl_3$, 25 °C) spectra of **L5**

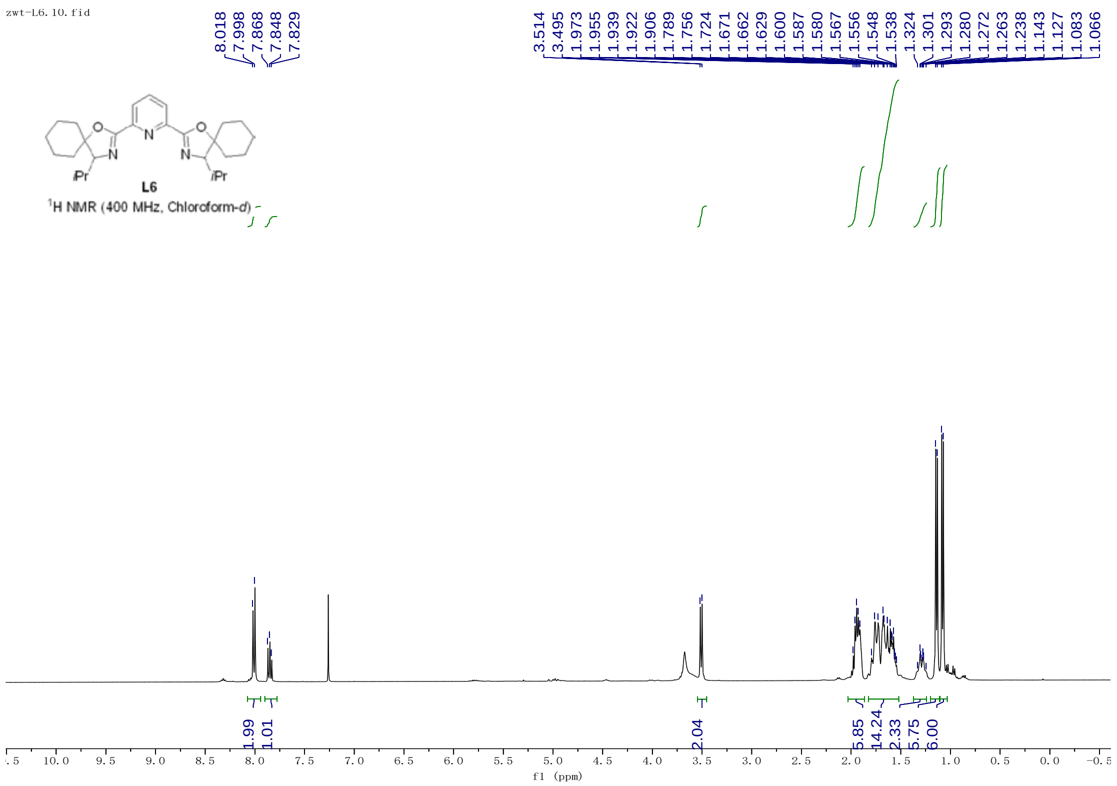

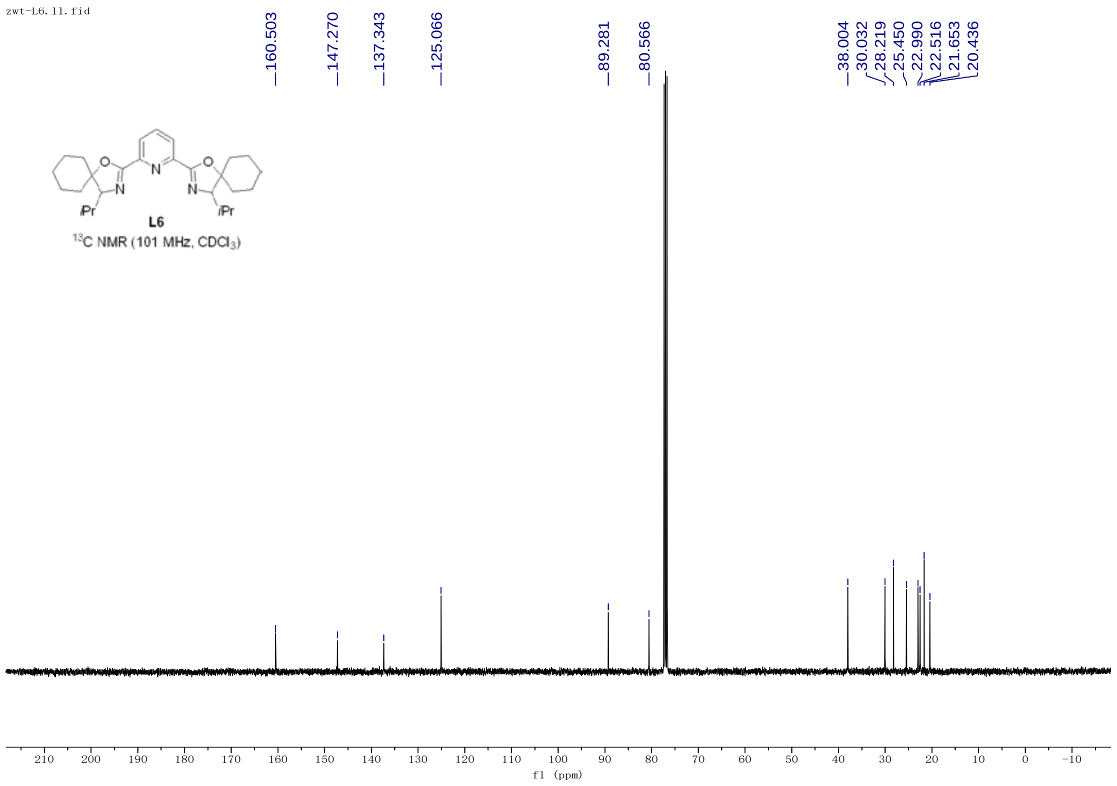

Supplementary Figure 98 ^{1}H-NMR (400 Mz, $CHCl_3$, 25 °C) and ^{13}C-NMR (101 MHz, $CHCl_3$, 25 °C) spectra of **L6**

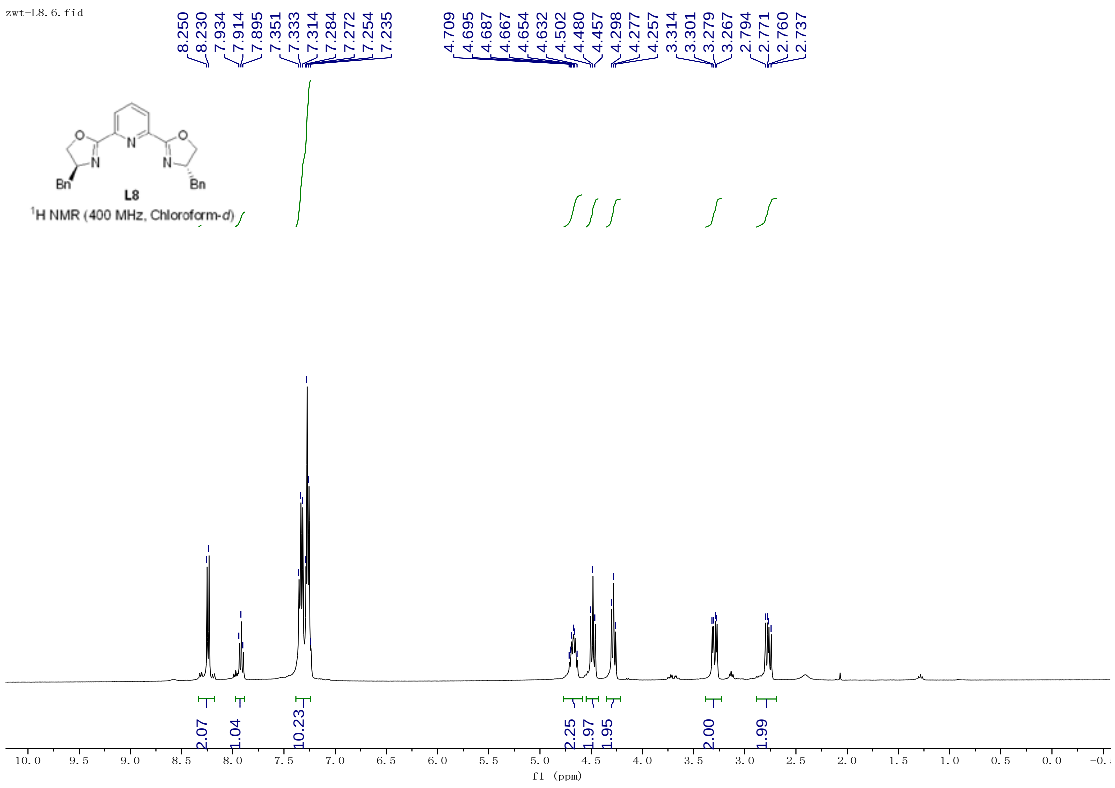

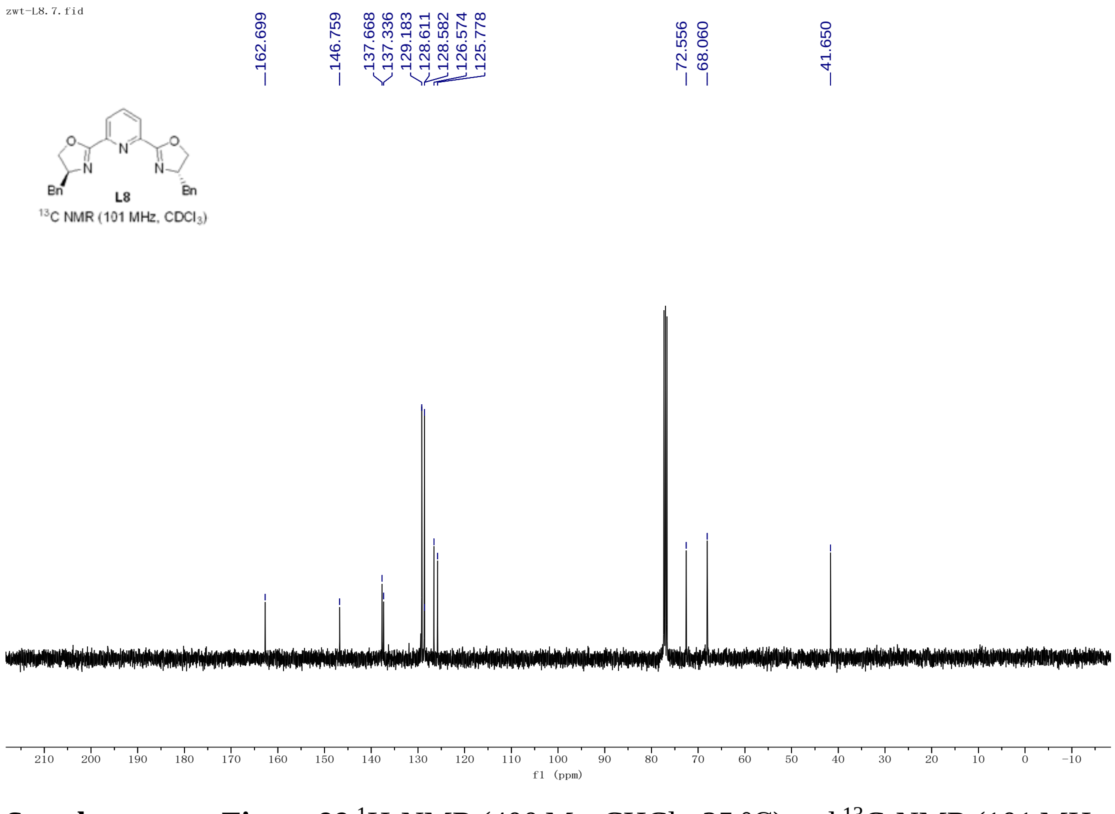

Supplementary Figure 99 ^{1}H-NMR (400 Mz, $CHCl_3$, 25 °C) and ^{13}C-NMR (101 MHz, $CHCl_3$, 25 °C) spectra of **L8**

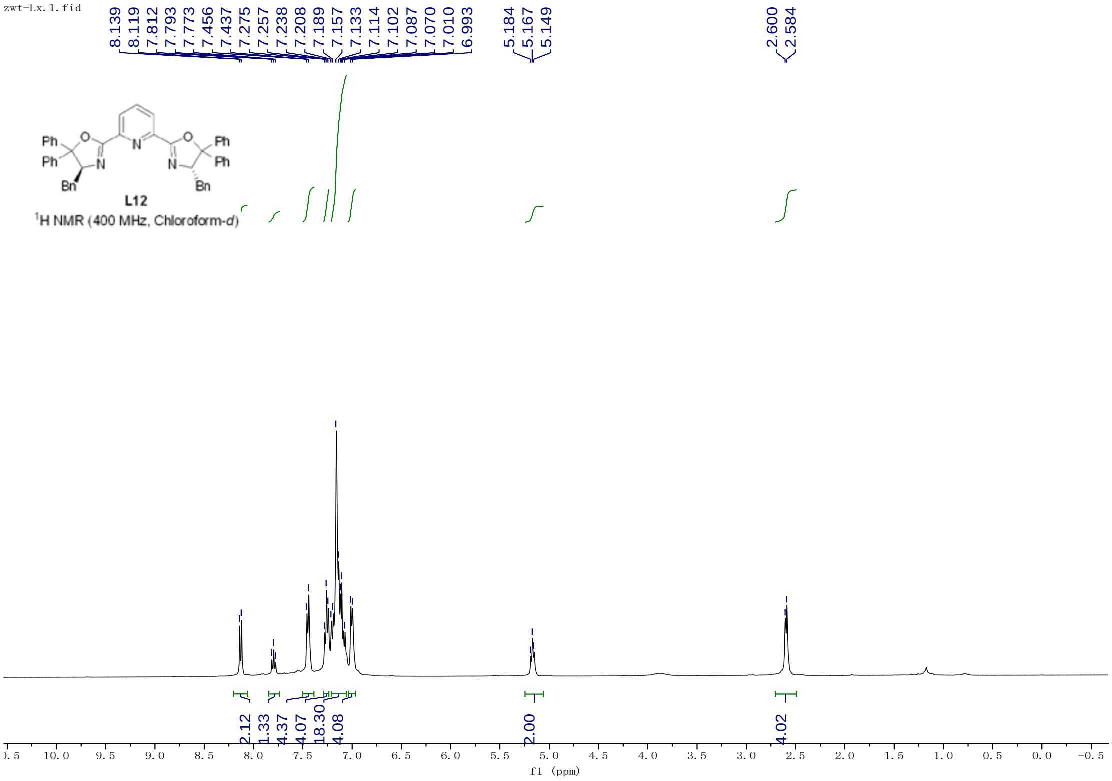

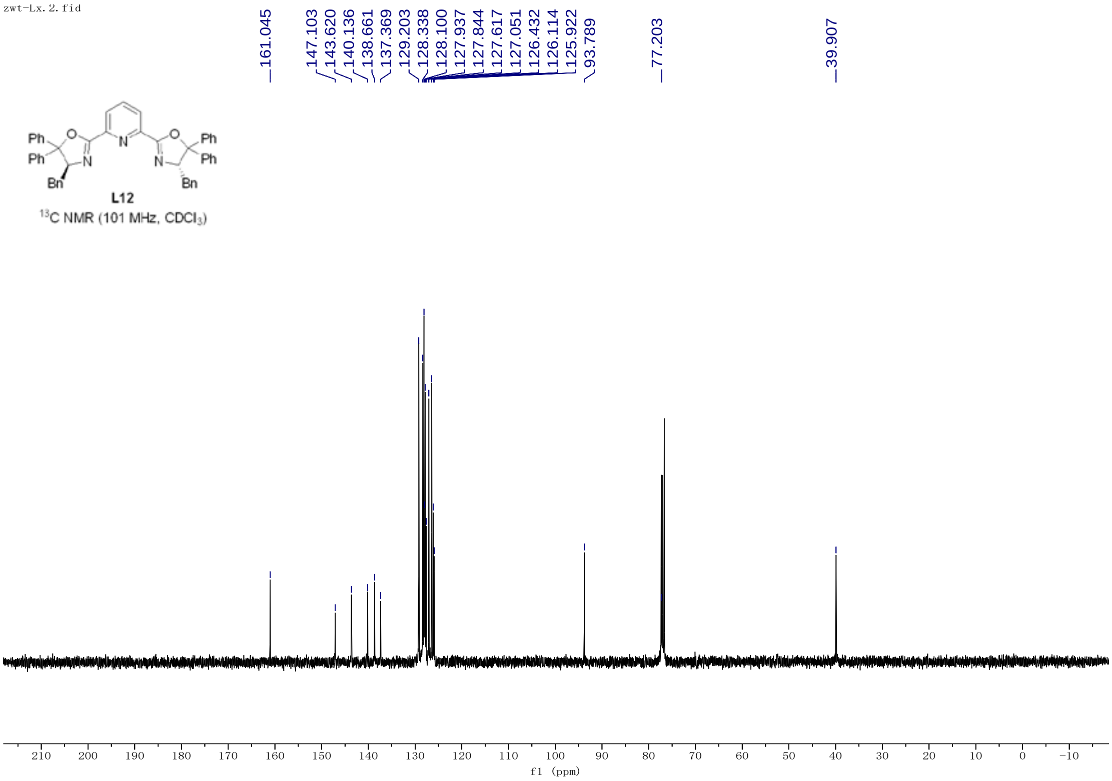

Supplementary Figure 100 ^{1}H-NMR (400 Mz, $CHCl_3$, 25 °C) and ^{13}C-NMR (101 MHz, $CHCl_3$, 25 °C) spectra of **L12**

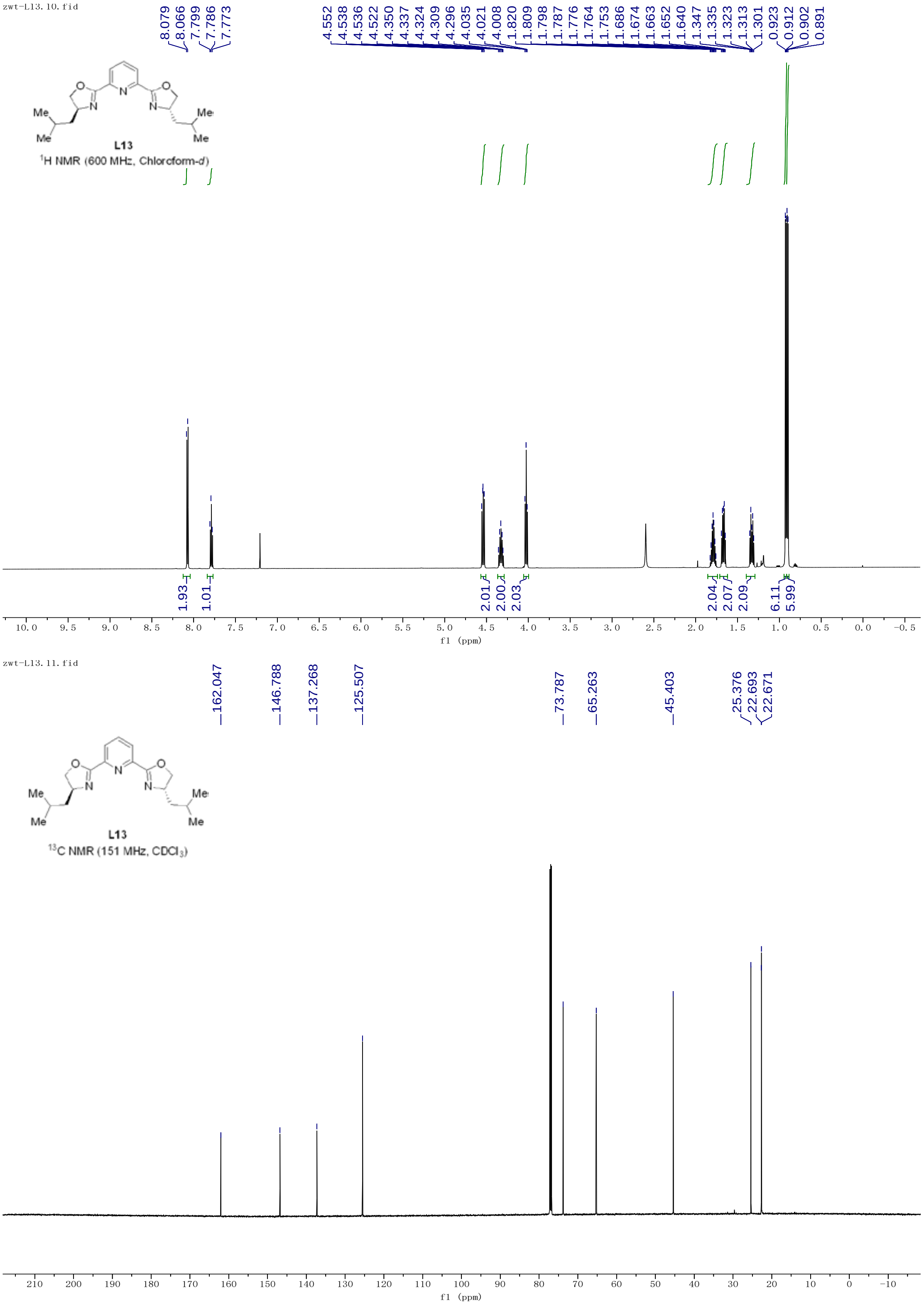

Supplementary Figure 101 ^{1}H-NMR (600 Mz, $CHCl_3$, 25 °C) and ^{13}C-NMR (151 MHz, $CHCl_3$, 25 °C) spectra of **L13**

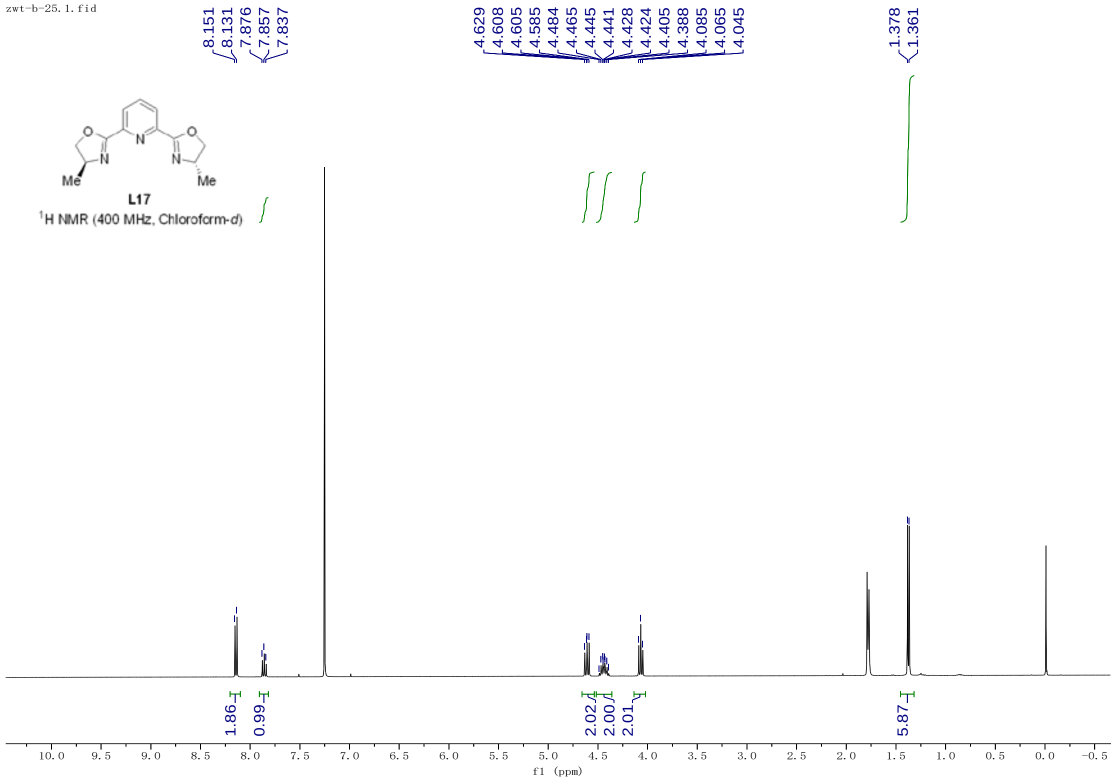

Supplementary Figure 102 ^{1}H-NMR (400 Mz, $CHCl_3$, 25 ℃) spectra of **L17**

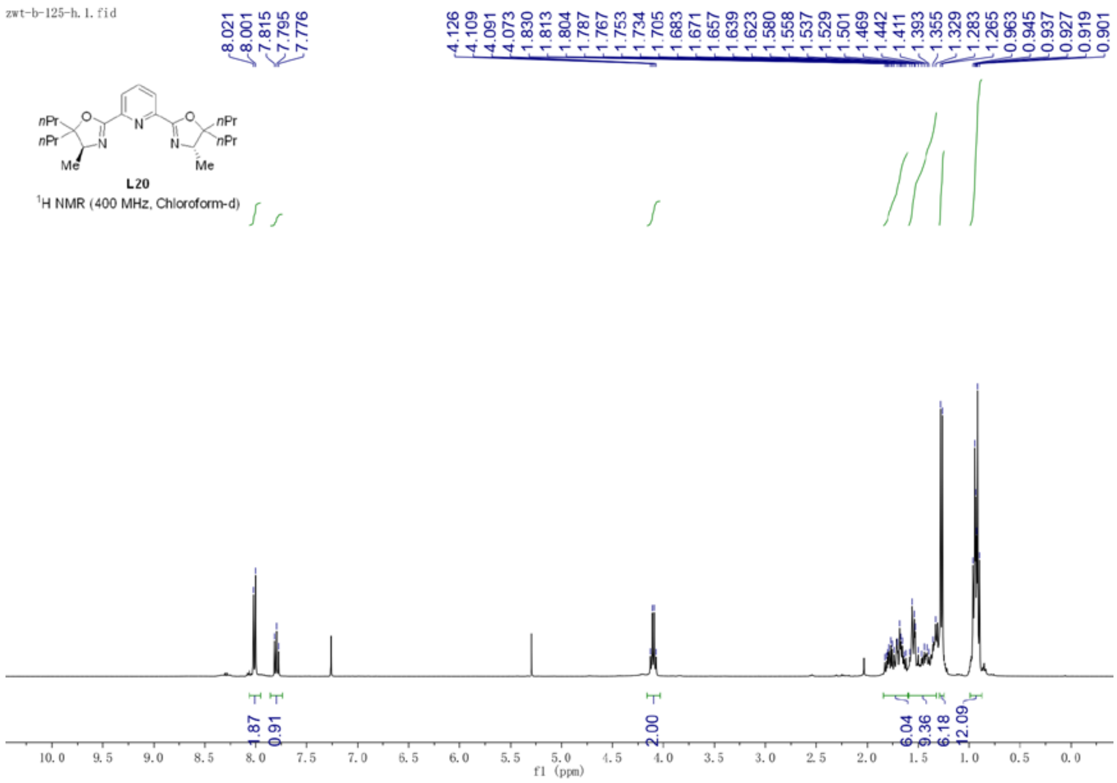

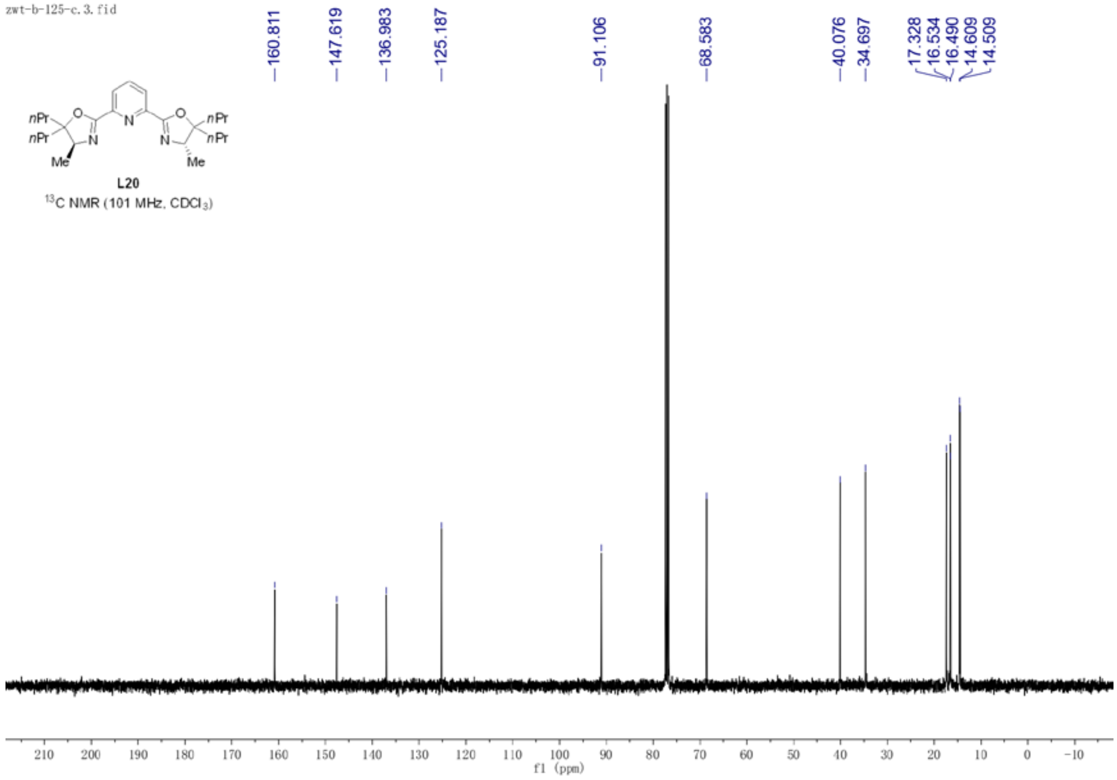

Supplementary Figure 103 ^{1}H-NMR (400 Mz, $CHCl_3$, 25 °C) and ^{13}C-NMR (101 MHz, $CHCl_3$, 25 °C) spectra of **L20**

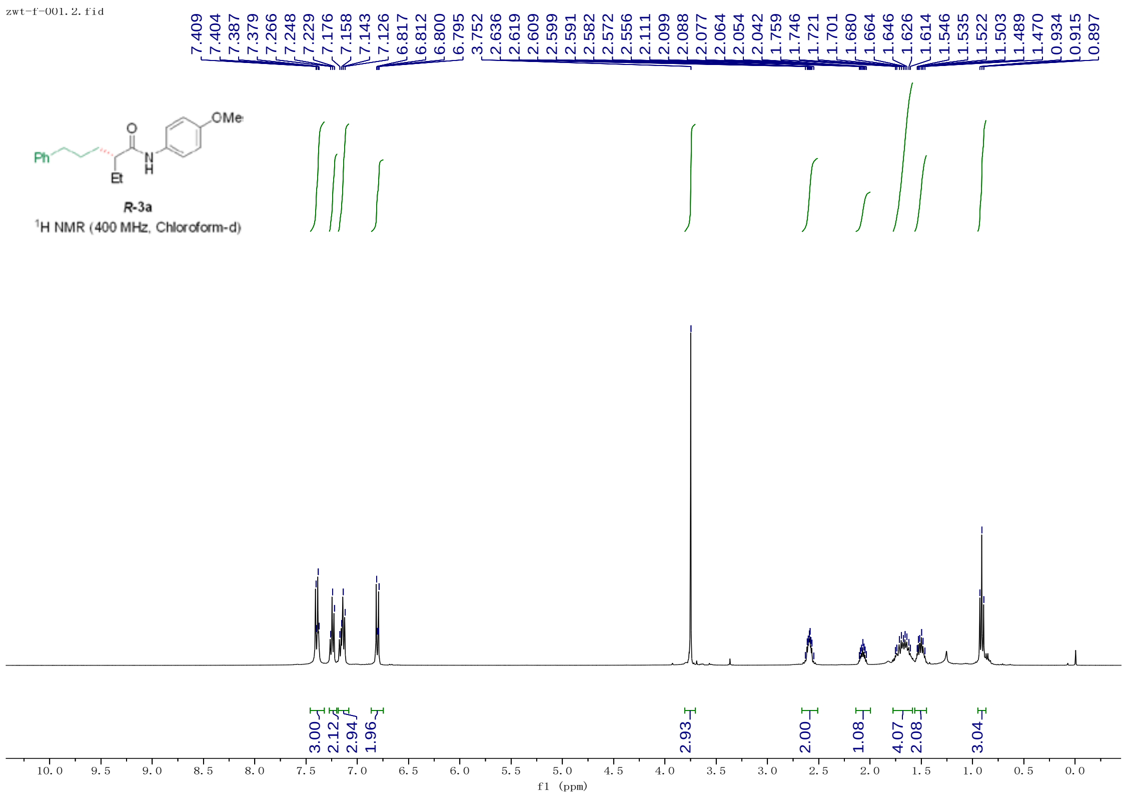

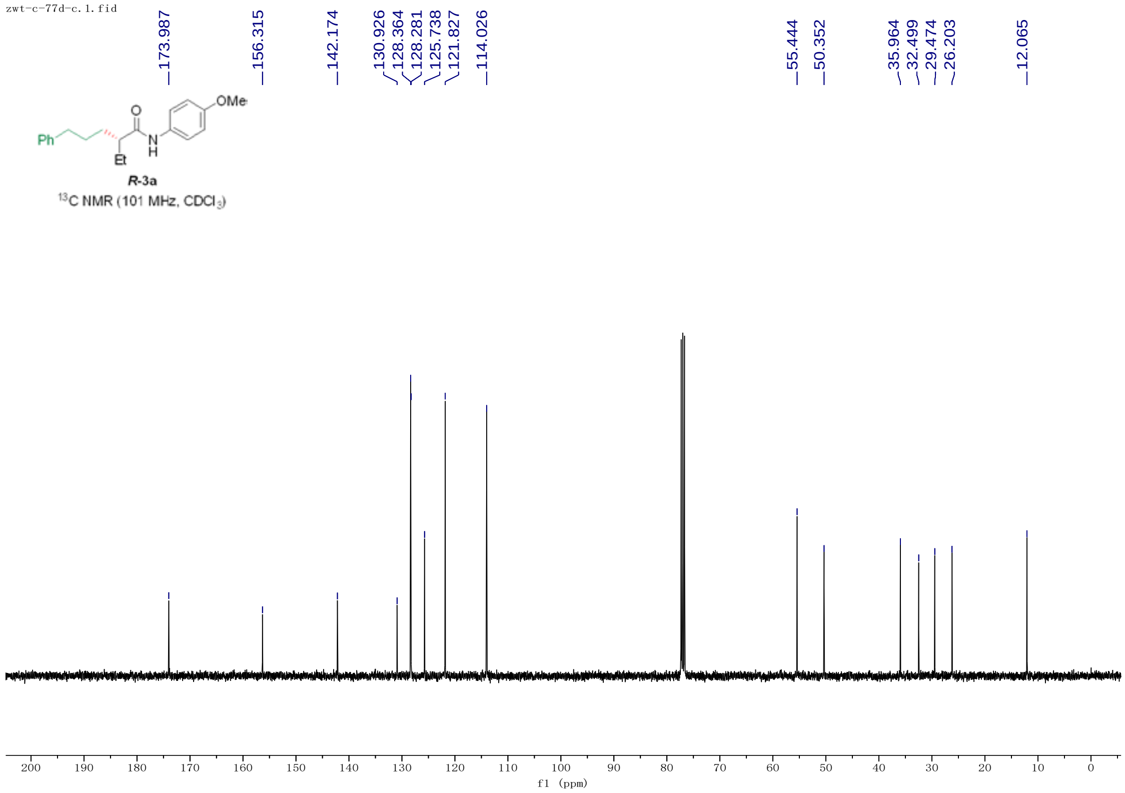

Supplementary Figure 104 ^{1}H-NMR (400 Mz, $CHCl_3$, 25 °C) and ^{13}C-NMR (101 MHz, $CHCl_3$, 25 °C) spectra of ***R*-3a**

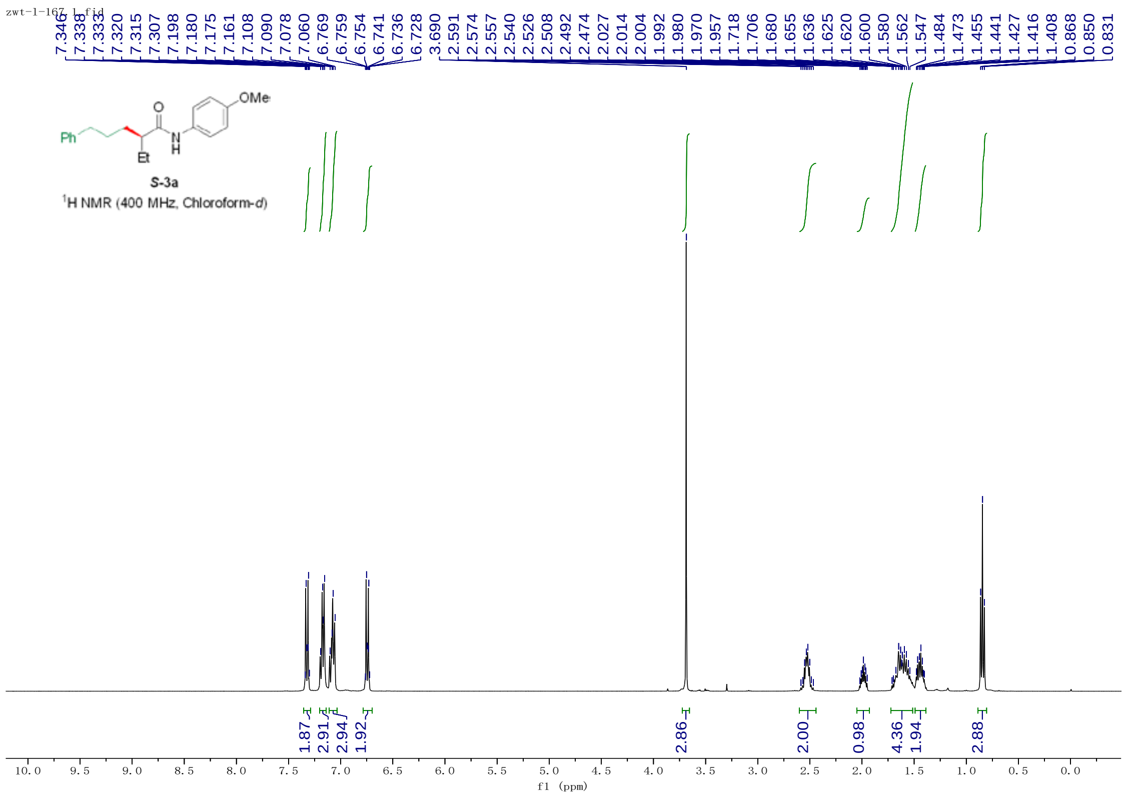

Supplementary Figure 105 ^{1}H-NMR (400 Mz, $CHCl_3$, 25 ℃) spectra of ***S*-3a**

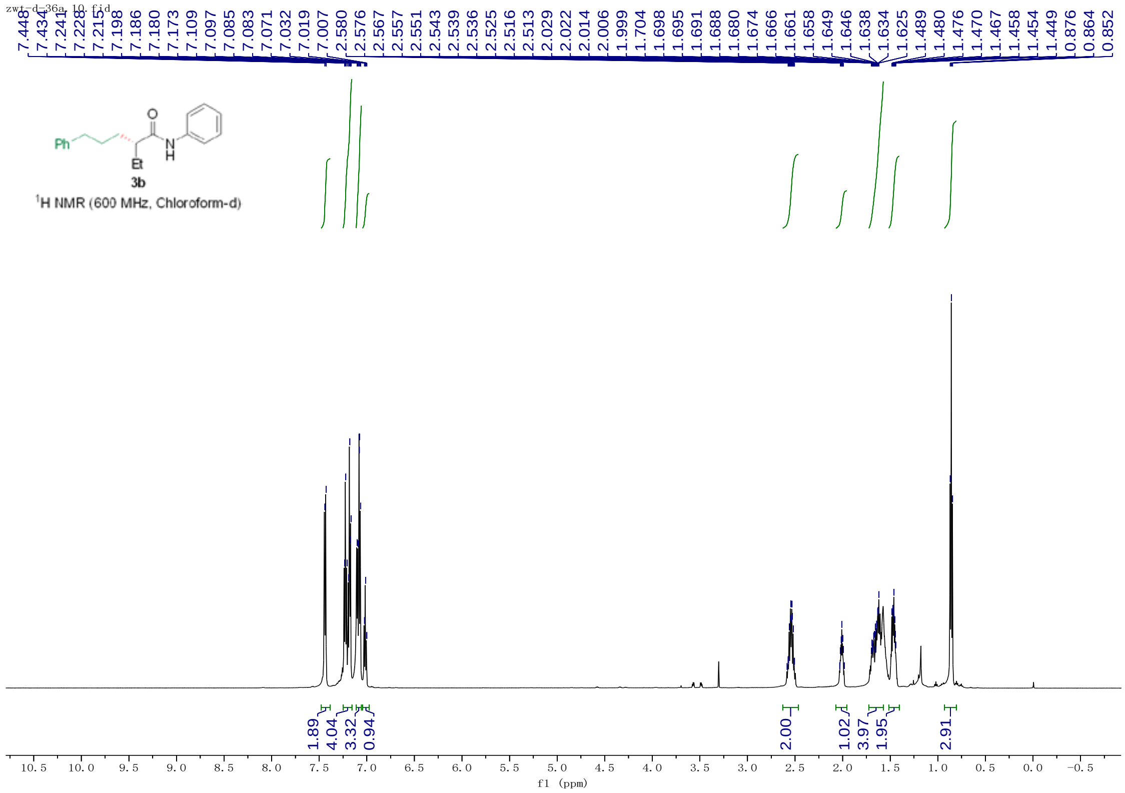

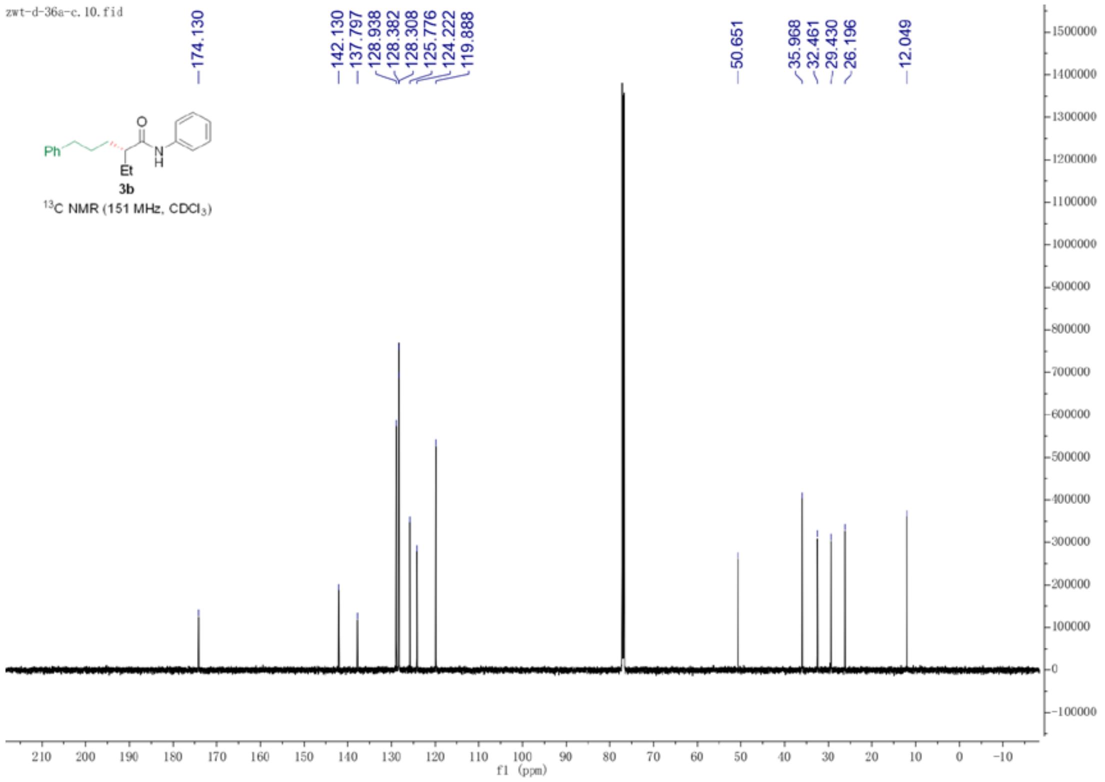

Supplementary Figure 106 ^{1}H-NMR (600 Mz, $CHCl_3$, 25 °C) and ^{13}C-NMR (151 MHz, $CHCl_3$, 25 °C) spectra of **3b**

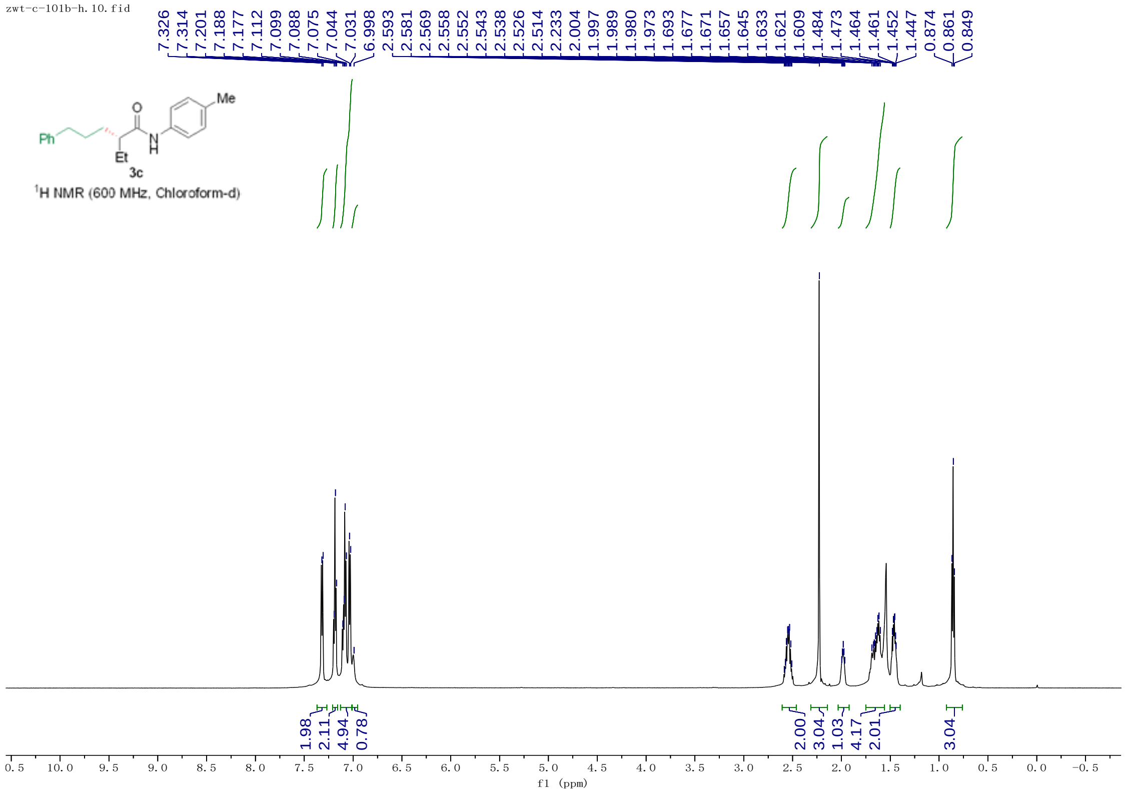

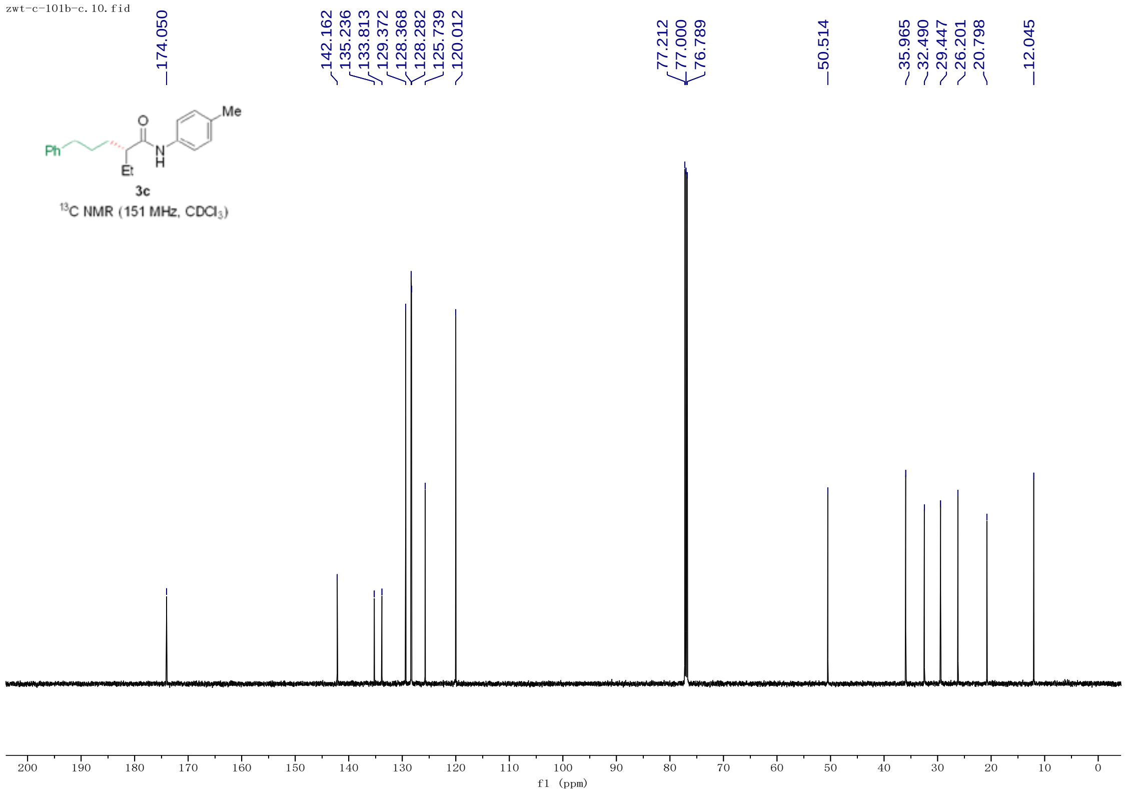

Supplementary Figure 107 ^{1}H-NMR (600 Mz, $CHCl_3$, 25 °C) and ^{13}C-NMR (151 MHz, $CHCl_3$, 25 °C) spectra of **3c**

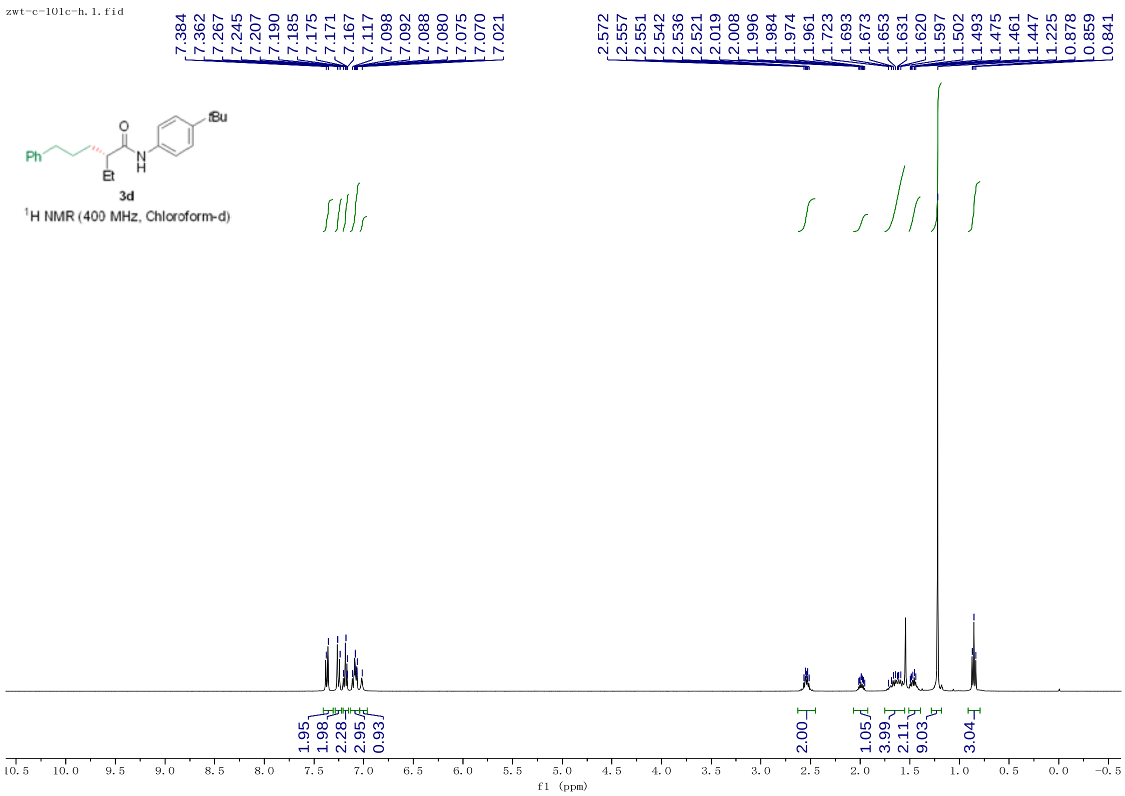

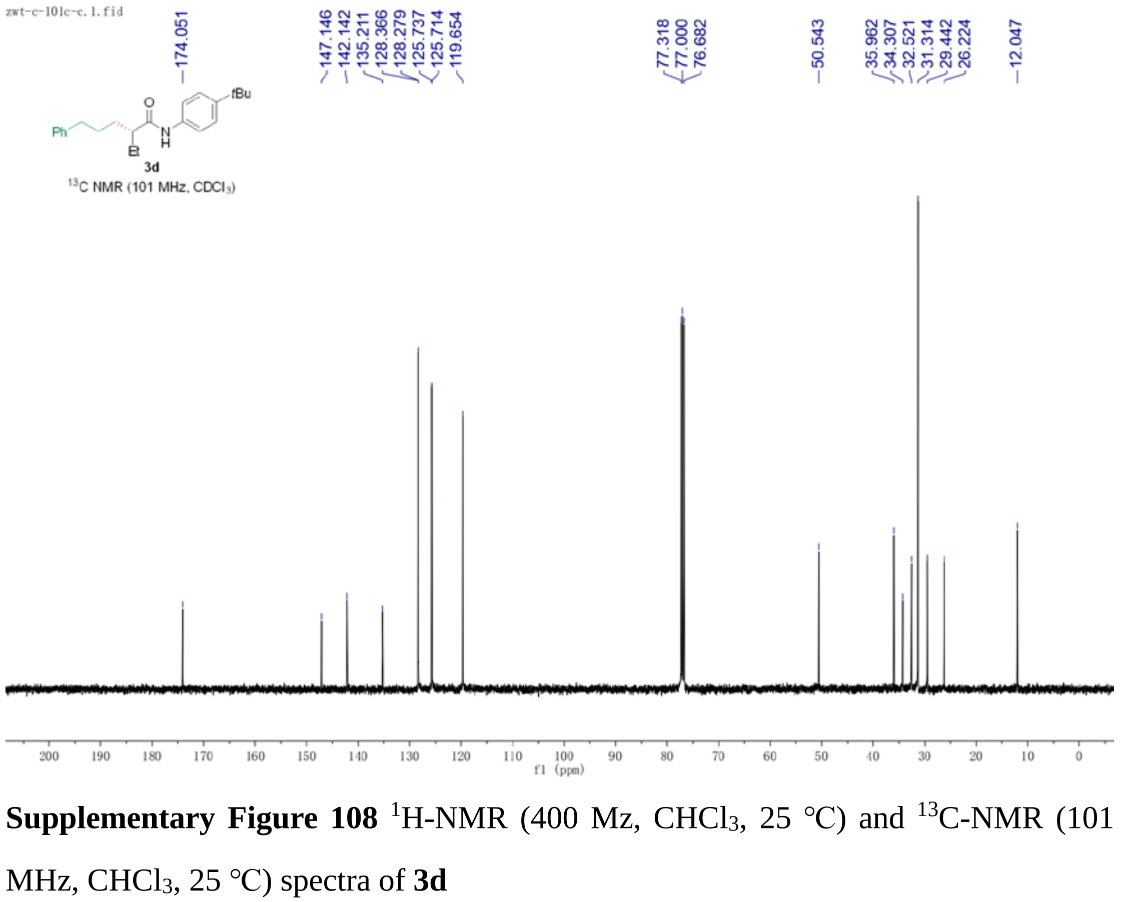

Supplementary Figure 108 ^{1}H-NMR (400 Mz, $CHCl_3$, 25 °C) and ^{13}C-NMR (101 MHz, $CHCl_3$, 25 °C) spectra of **3d**

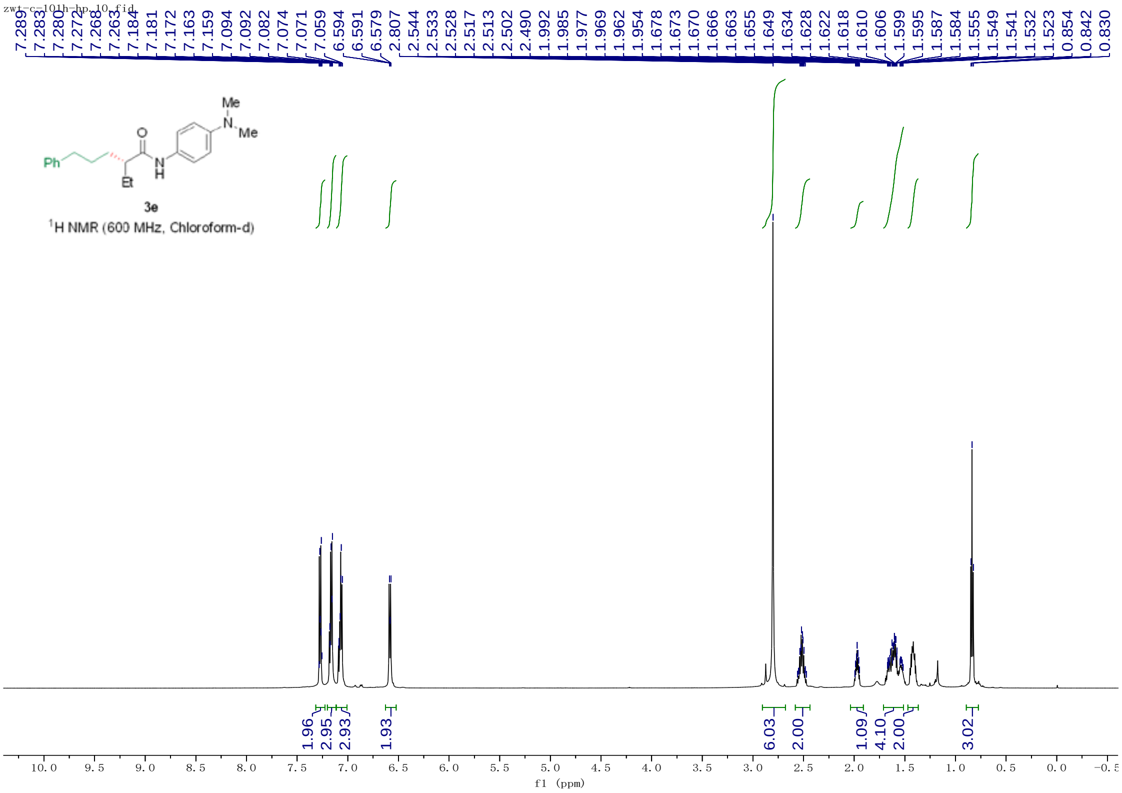

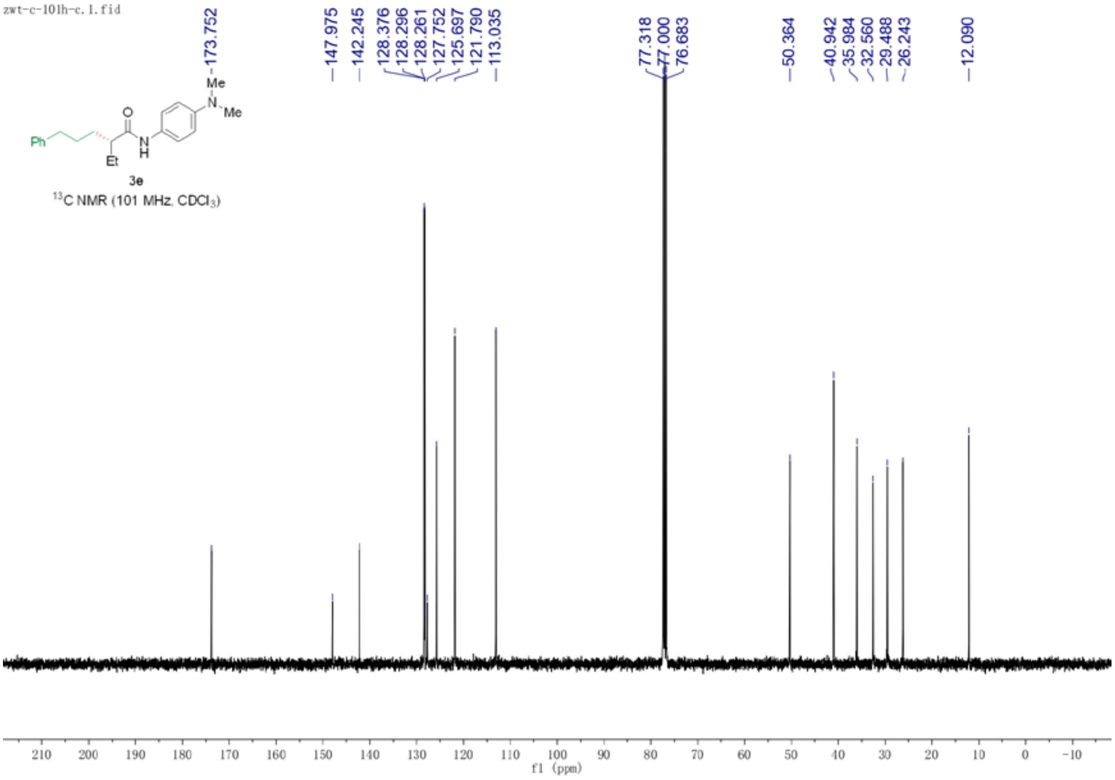

Supplementary Figure 109 ^{1}H-NMR (600 Mz, $CHCl_3$, 25 °C) and ^{13}C-NMR (101 MHz, $CHCl_3$, 25 °C) spectra of **3e**

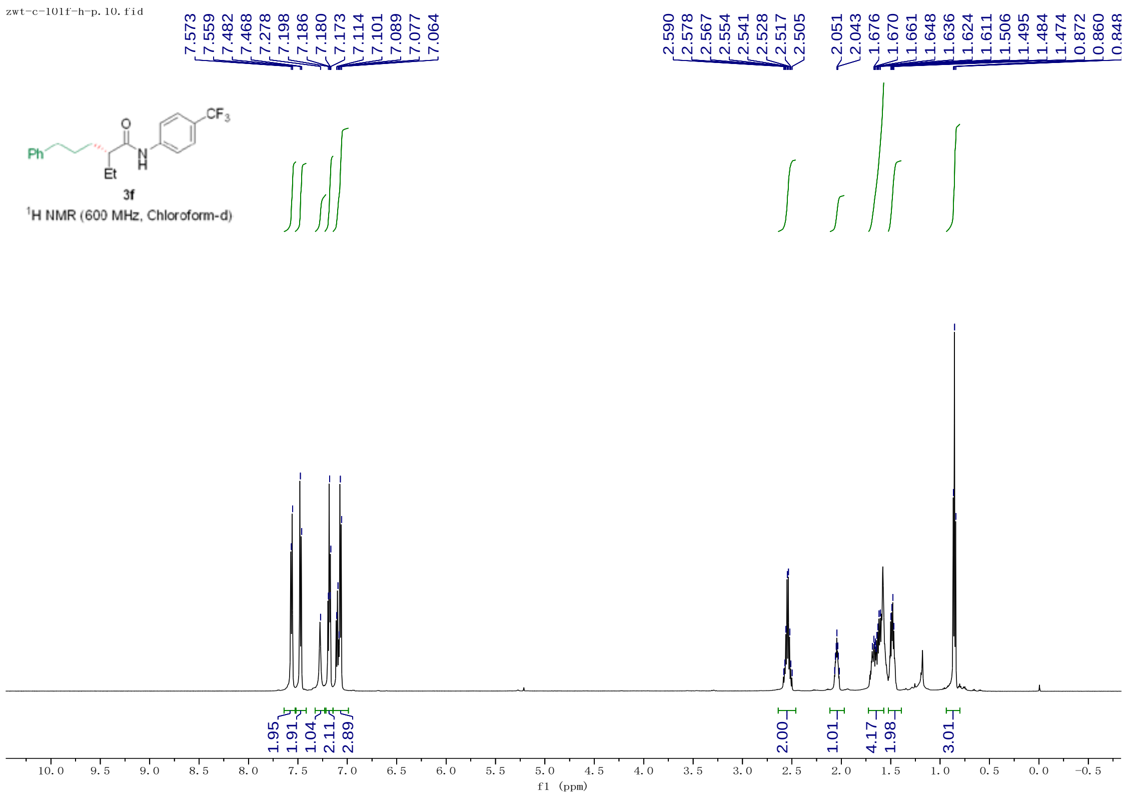

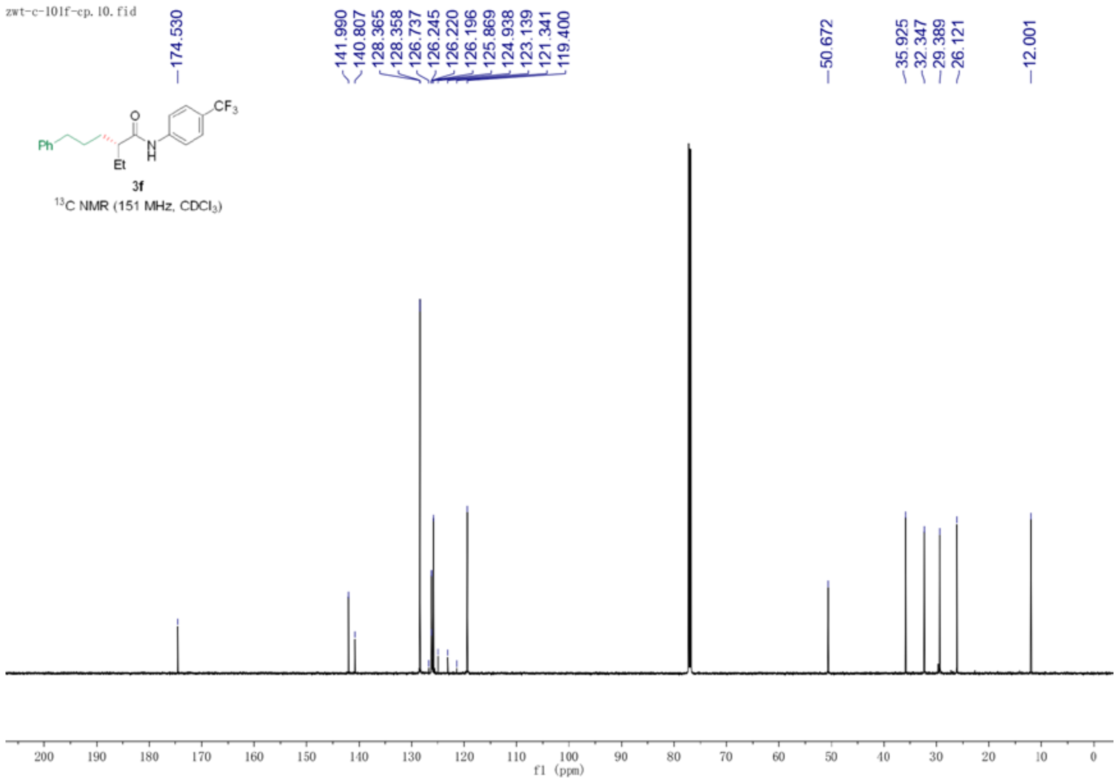

Supplementary Figure 110 ^{1}H-NMR (600 Mz, $CHCl_3$, 25 °C) and ^{13}C-NMR (151 MHz, $CHCl_3$, 25 °C) spectra of **3f**

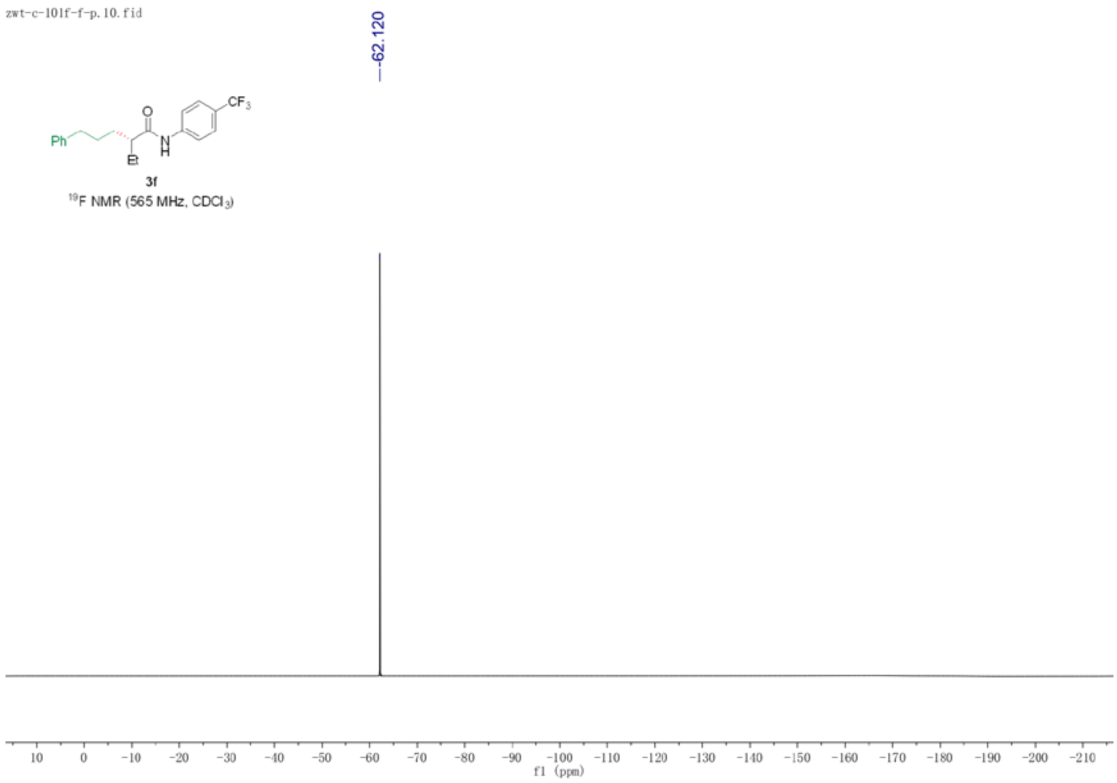

Supplementary Figure 111 ^{19}F-NMR (565 Mz, $CHCl_3$, 25 ℃) spectra of **3f**

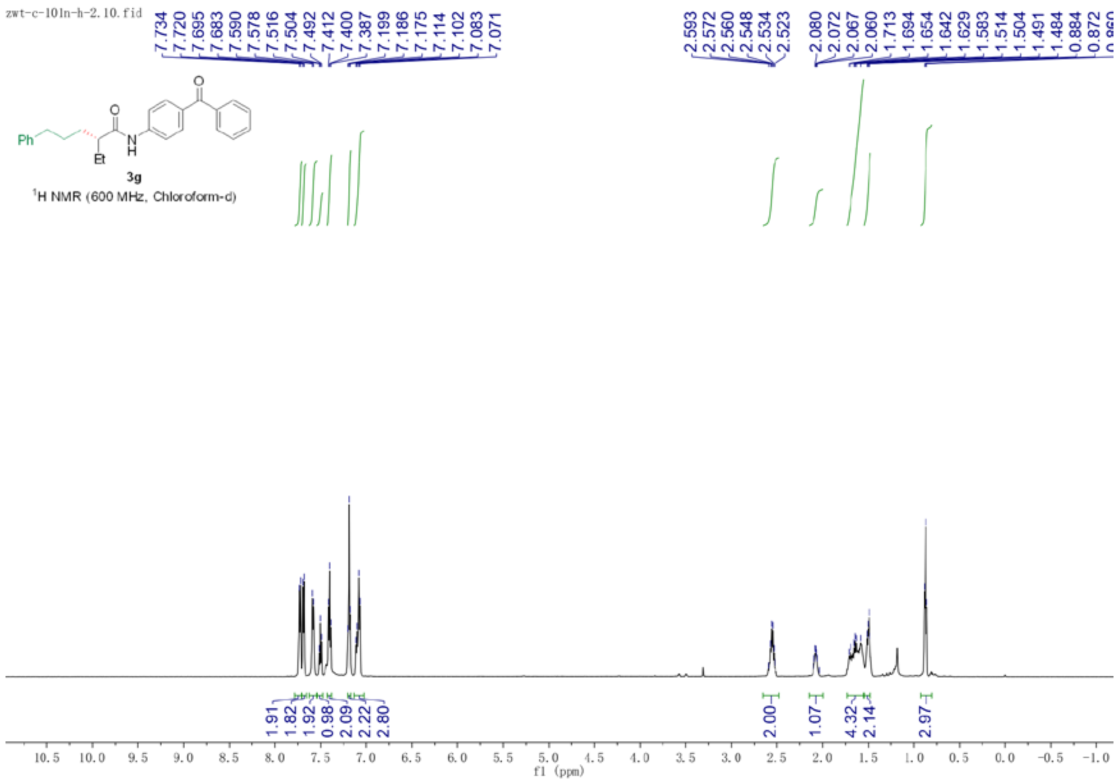

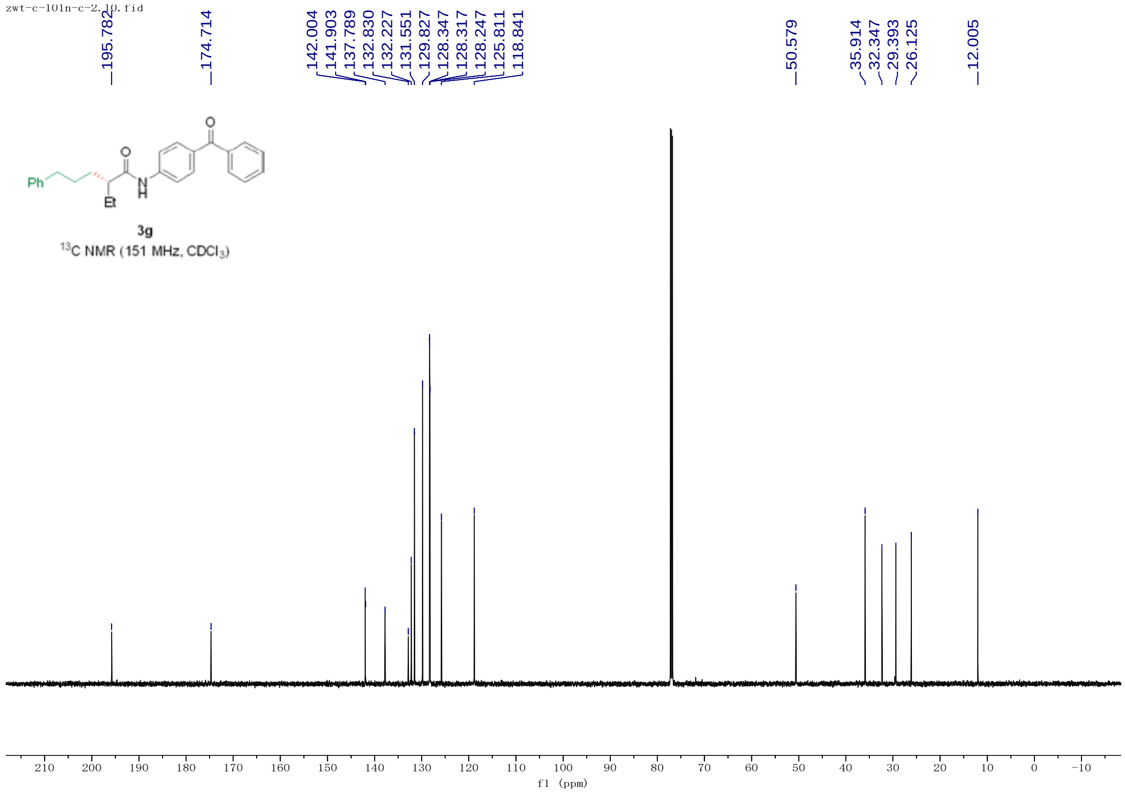

Supplementary Figure 112 ^{1}H-NMR (600 Mz, $CHCl_3$, 25 °C) and ^{13}C-NMR (151 MHz, $CHCl_3$, 25 °C) spectra of **3g**

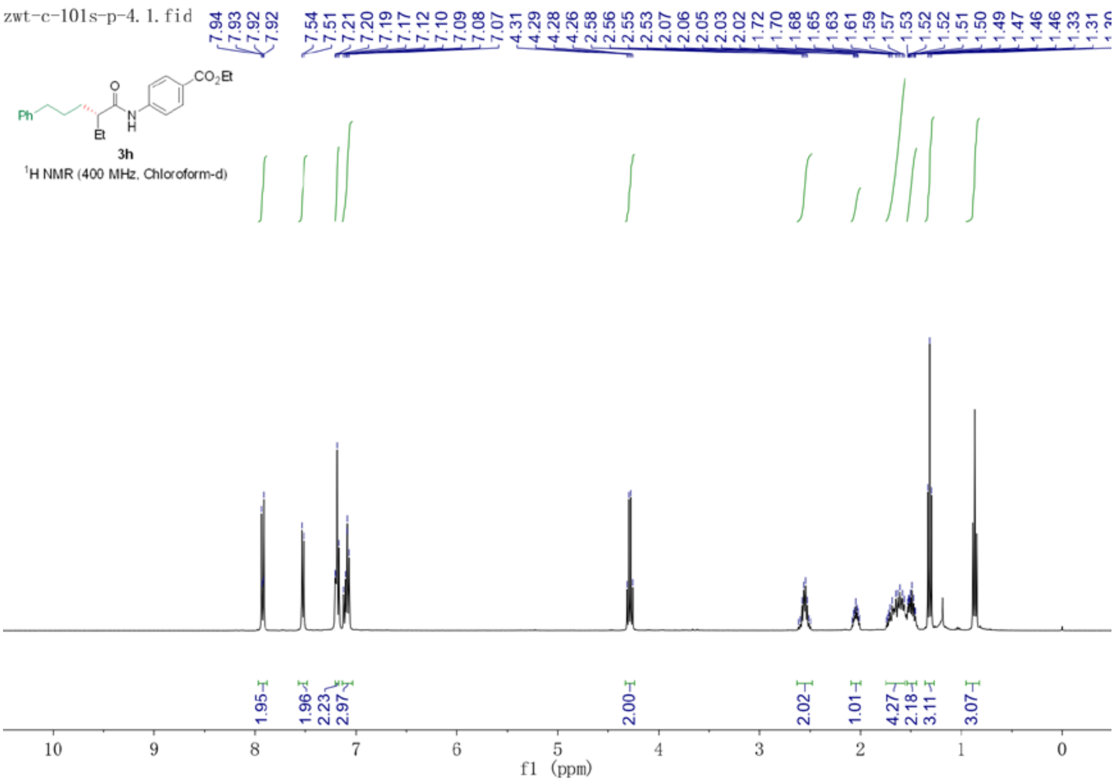

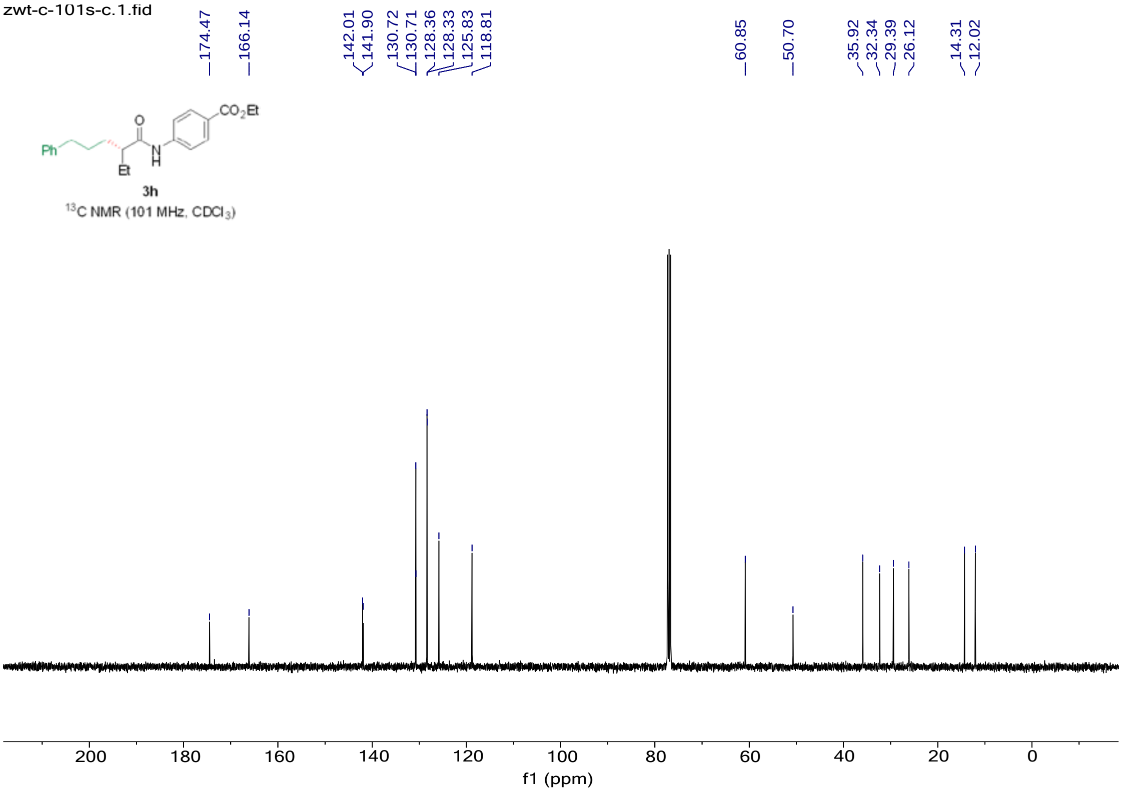

Supplementary Figure 113 ^{1}H-NMR (400 Mz, $CHCl_3$, 25 °C) and ^{13}C-NMR (101 MHz, $CHCl_3$, 25 °C) spectra of **3h**

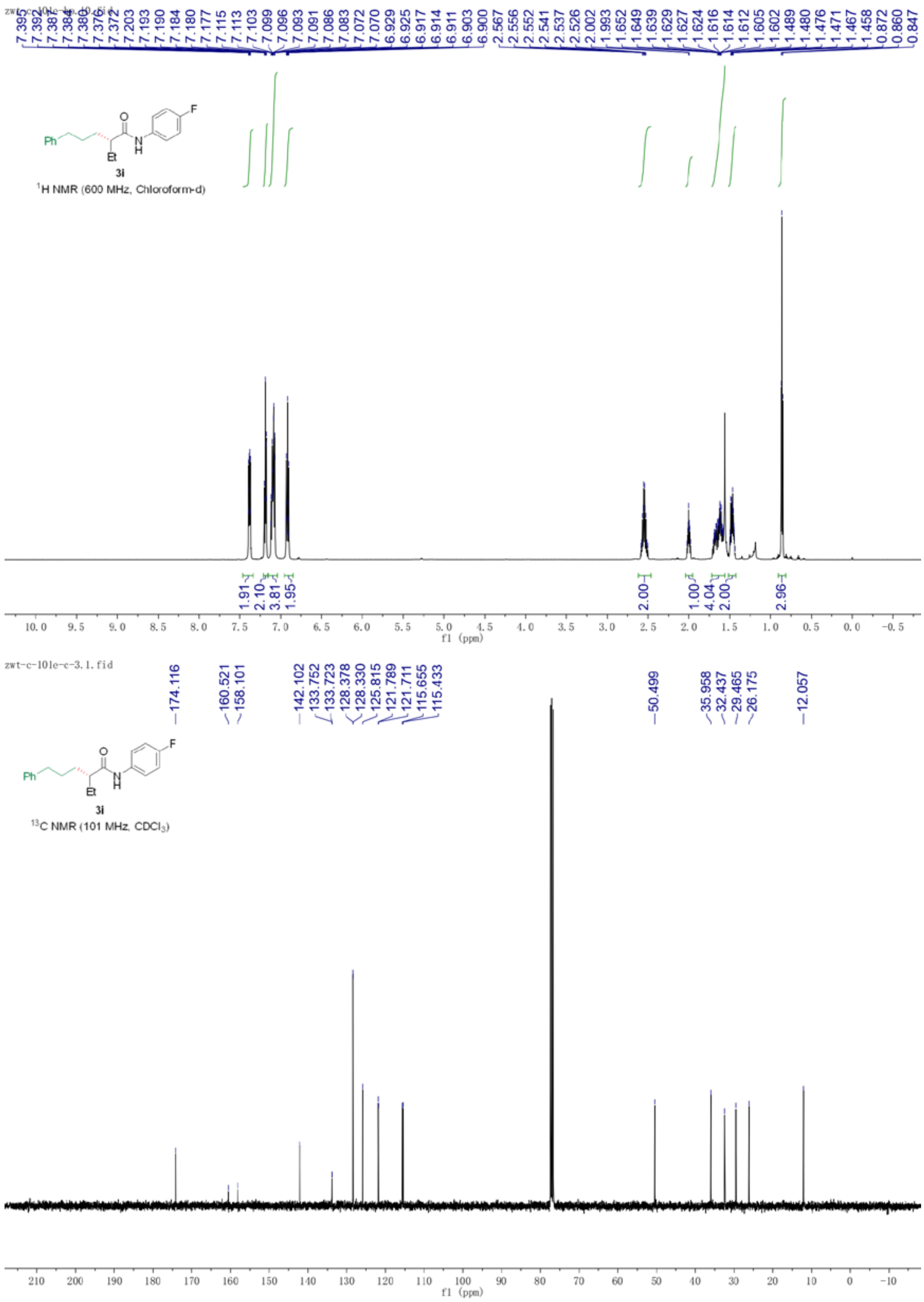

Supplementary Figure 114 ^{1}H-NMR (600 Mz, $CHCl_3$, 25 °C) and ^{13}C-NMR (101 MHz, $CHCl_3$, 25 °C) spectra of **3i**

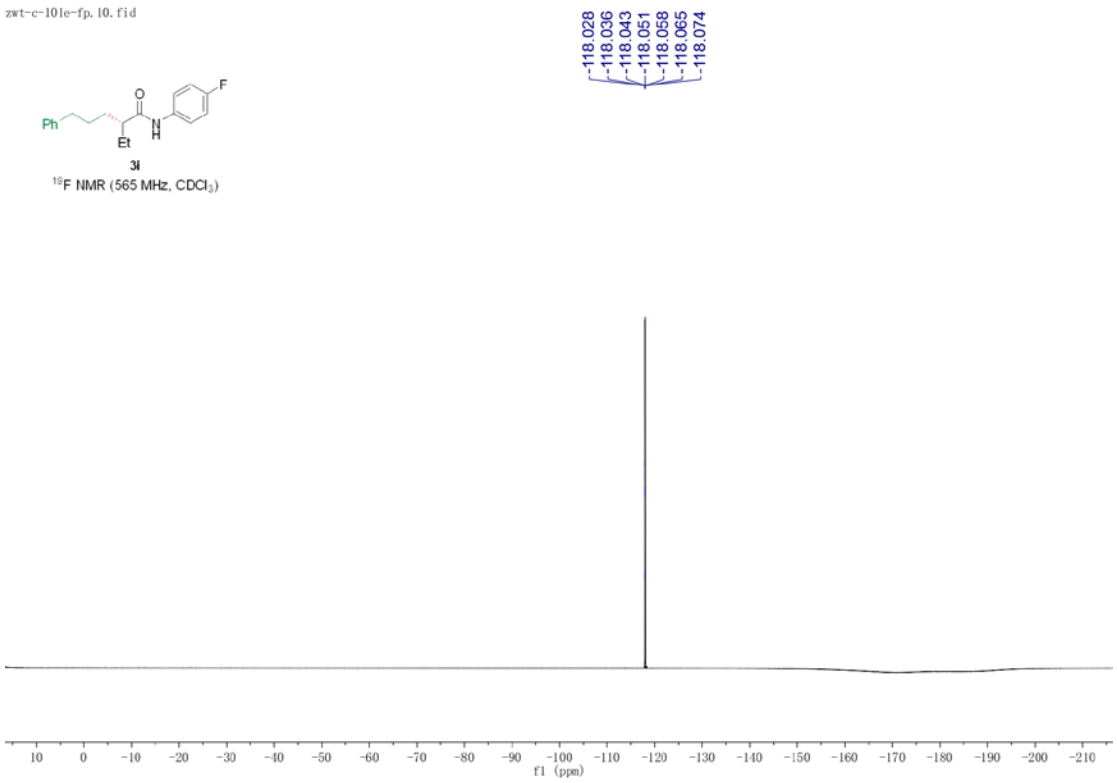

Supplementary Figure 115 ^{19}F-NMR (565 Mz, $CHCl_3$, 25 ℃) spectra of **3i**

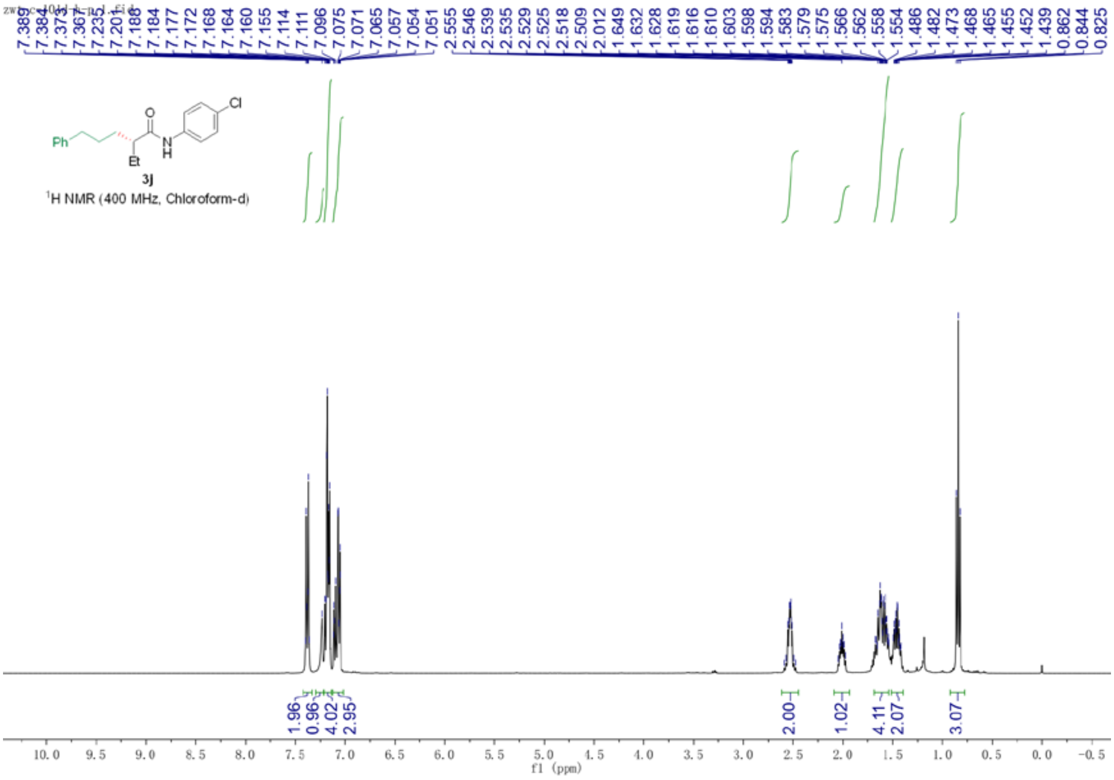

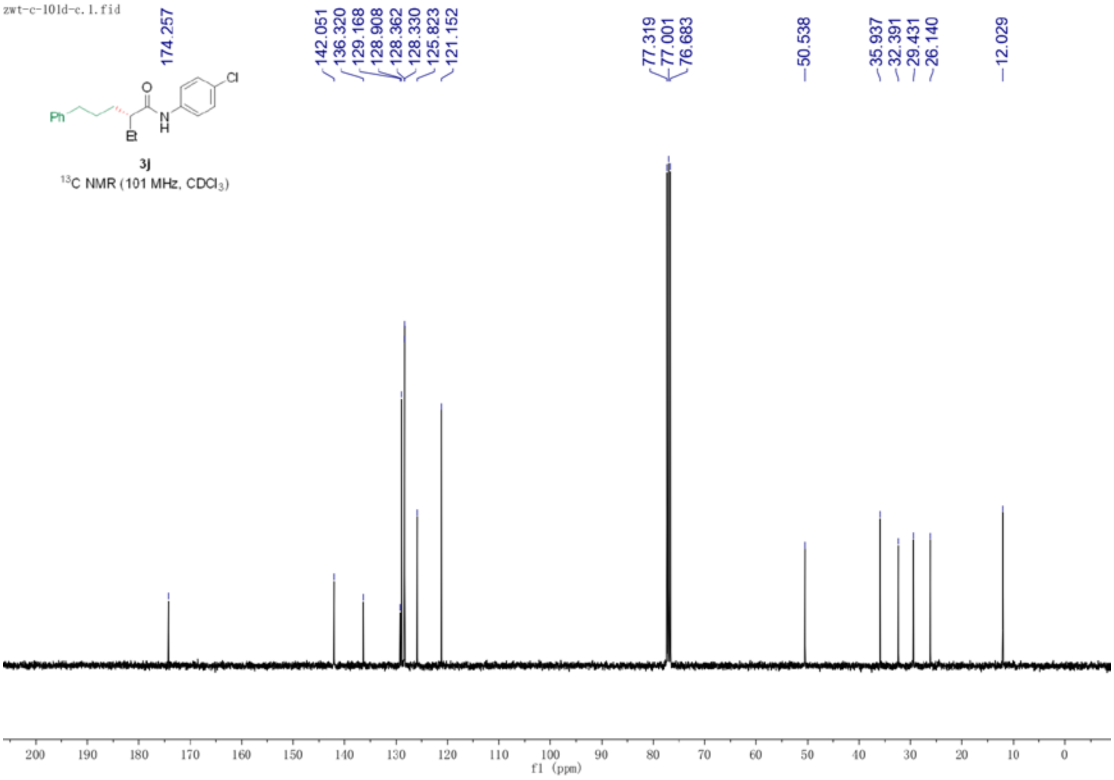

Supplementary Figure 116 ^{1}H-NMR (400 Mz, $CHCl_3$, 25 °C) and ^{13}C-NMR (101 MHz, $CHCl_3$, 25 °C) spectra of **3j**

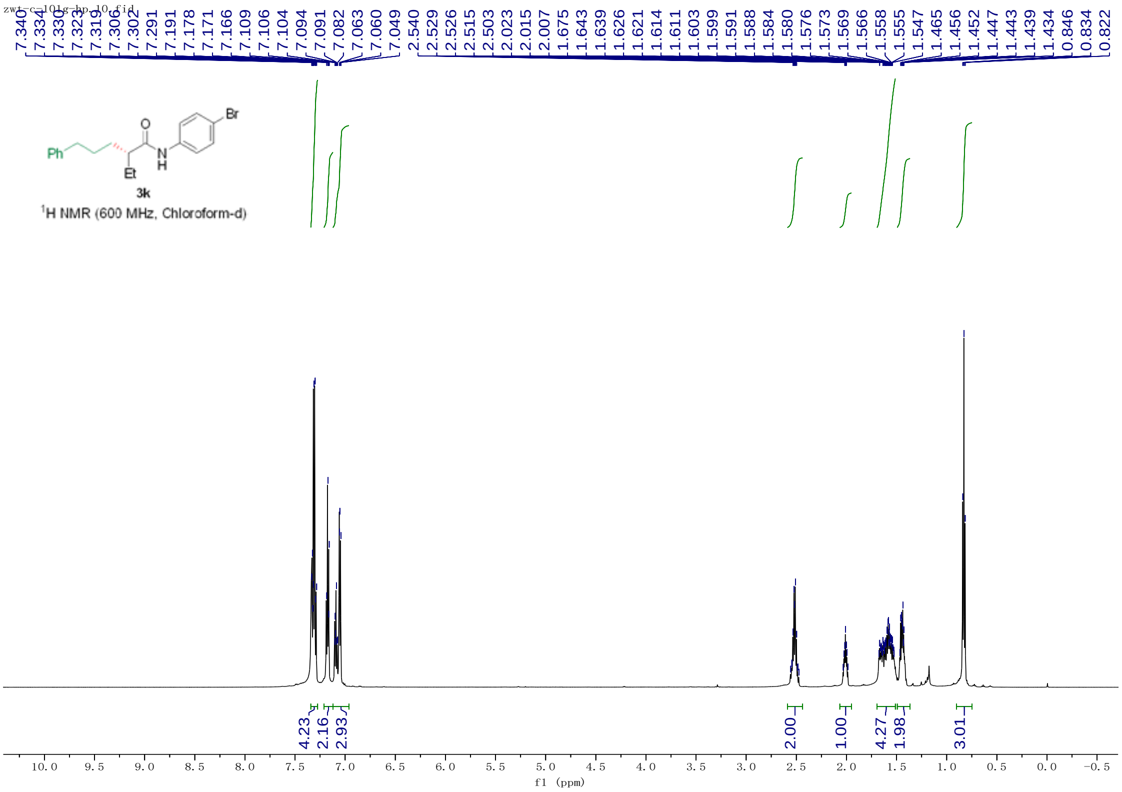

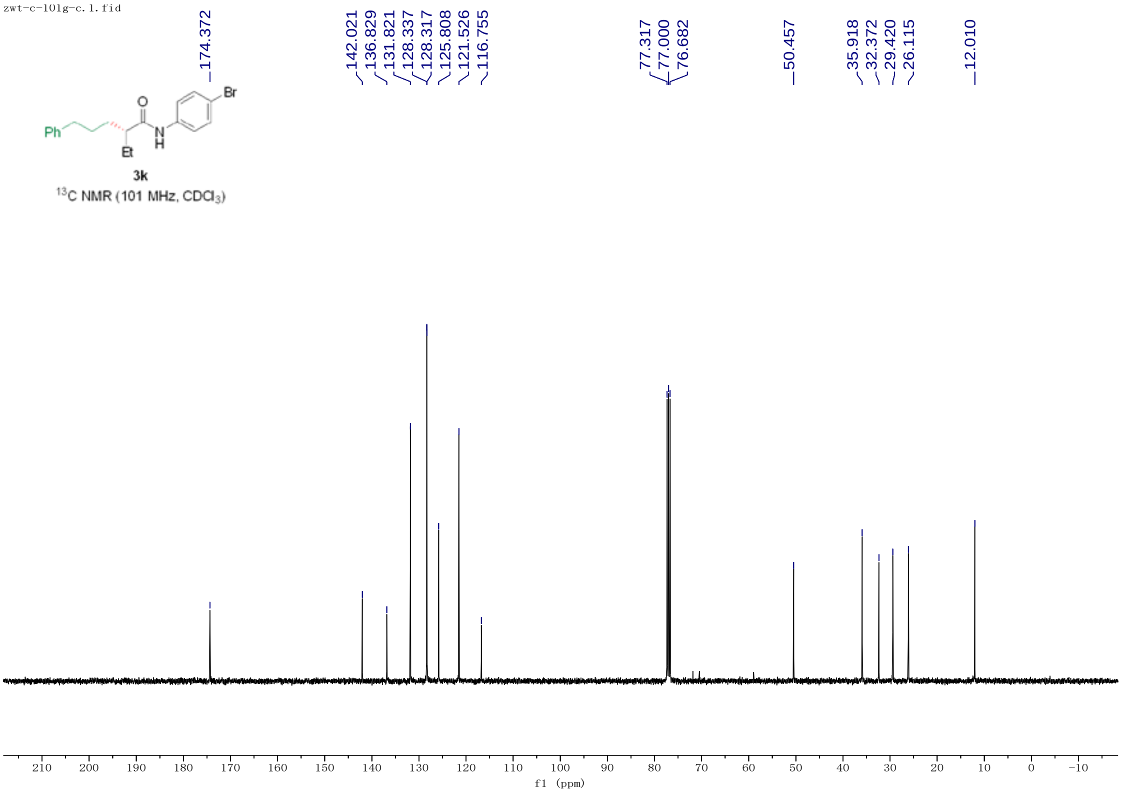

Supplementary Figure 117 ^{1}H-NMR (600 Mz, $CHCl_3$, 25 °C) and ^{13}C-NMR (101 MHz, $CHCl_3$, 25 °C) spectra of **3k**

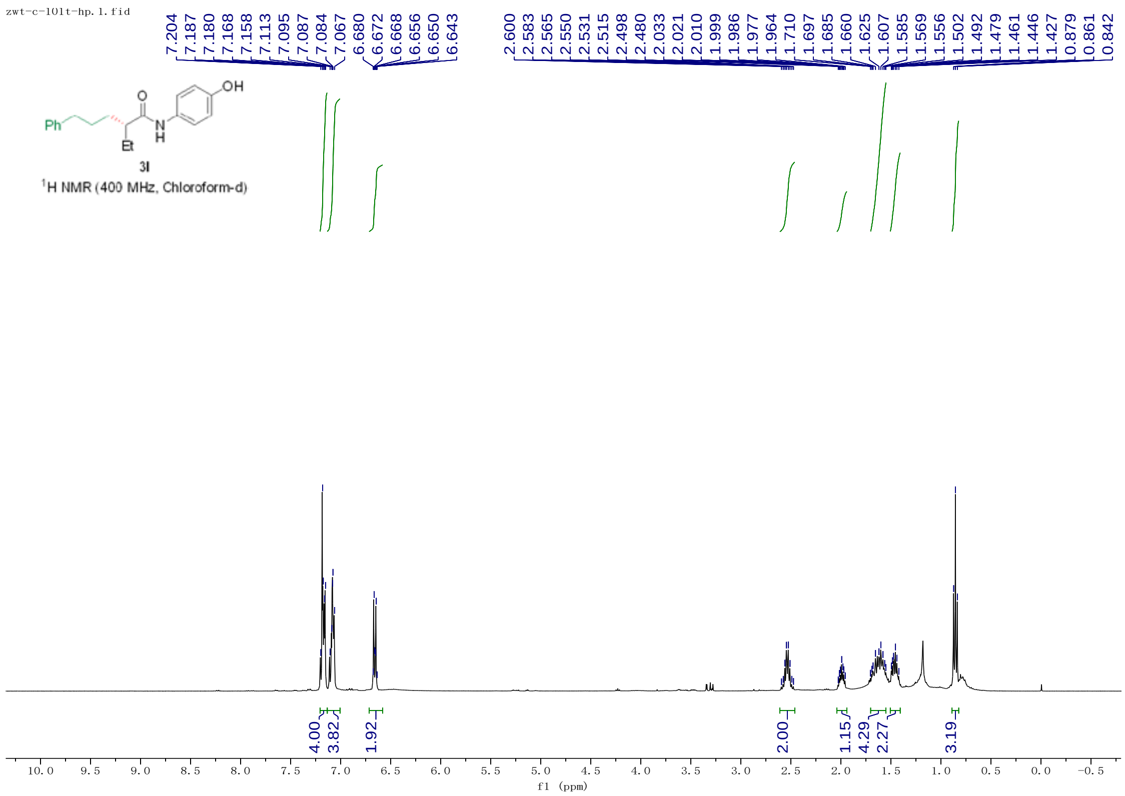

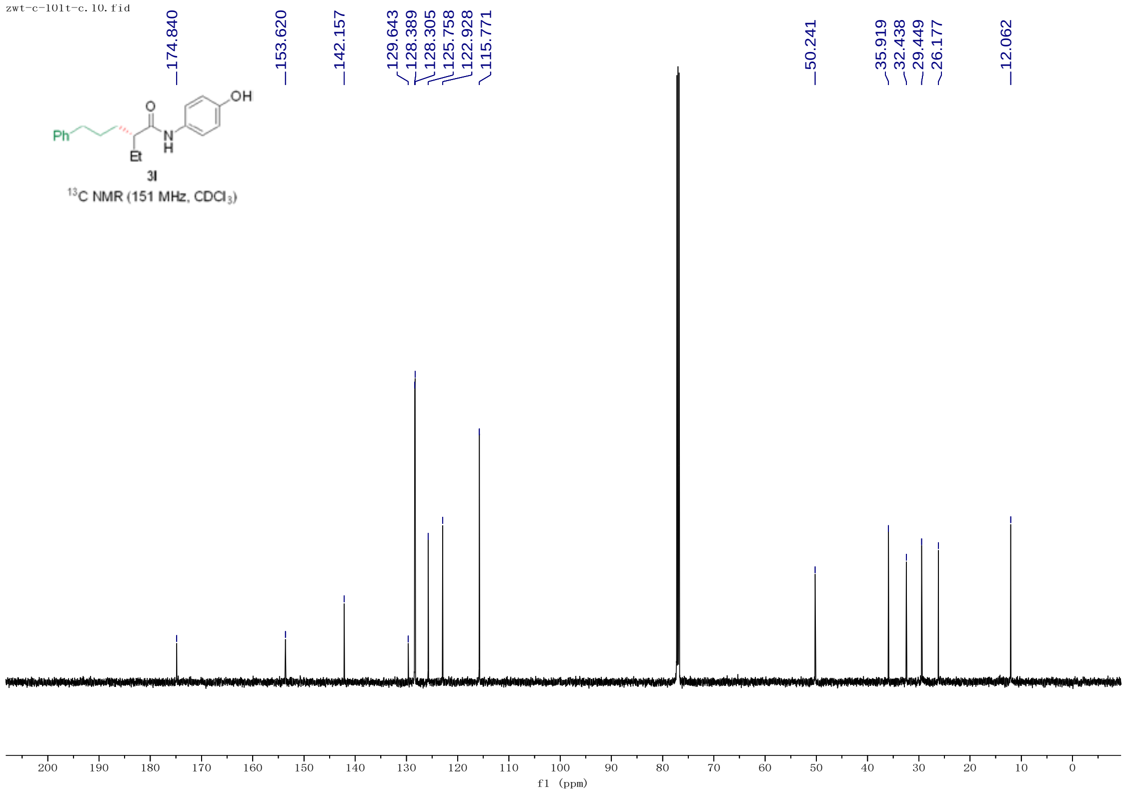

Supplementary Figure 118 ^{1}H-NMR (400 Mz, $CHCl_3$, 25 °C) and ^{13}C-NMR (151 MHz, $CHCl_3$, 25 °C) spectra of **3l**

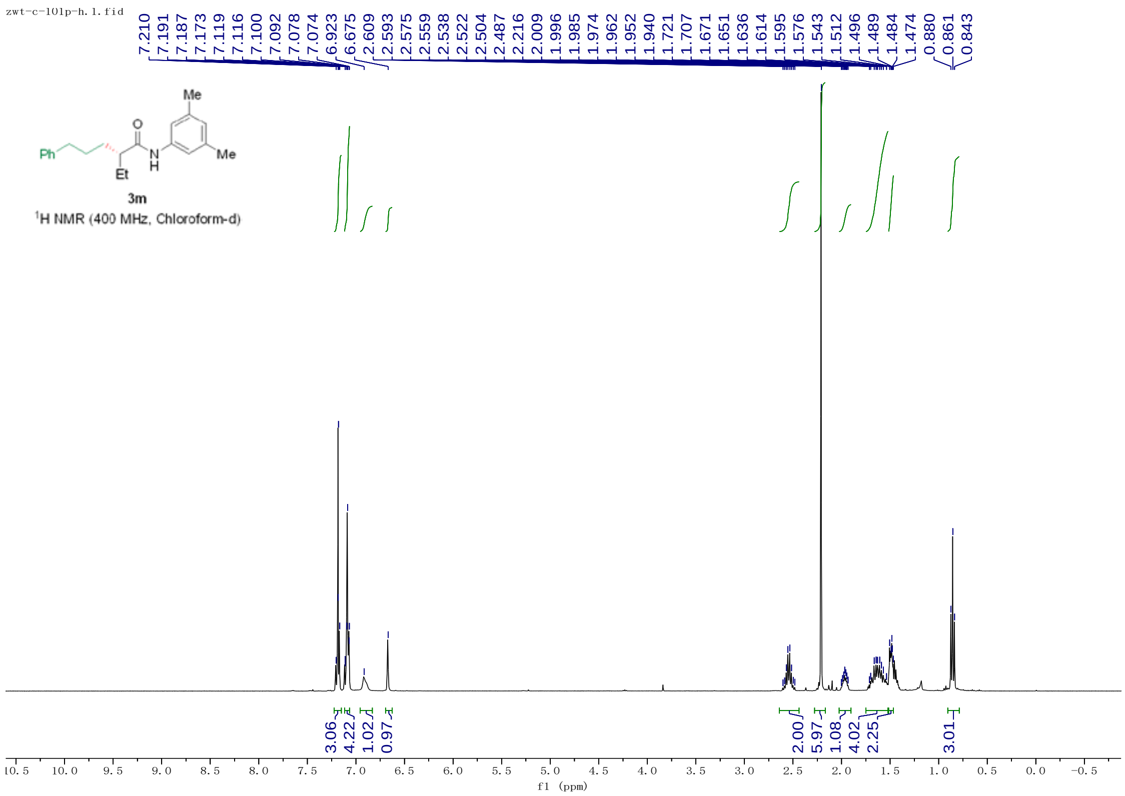

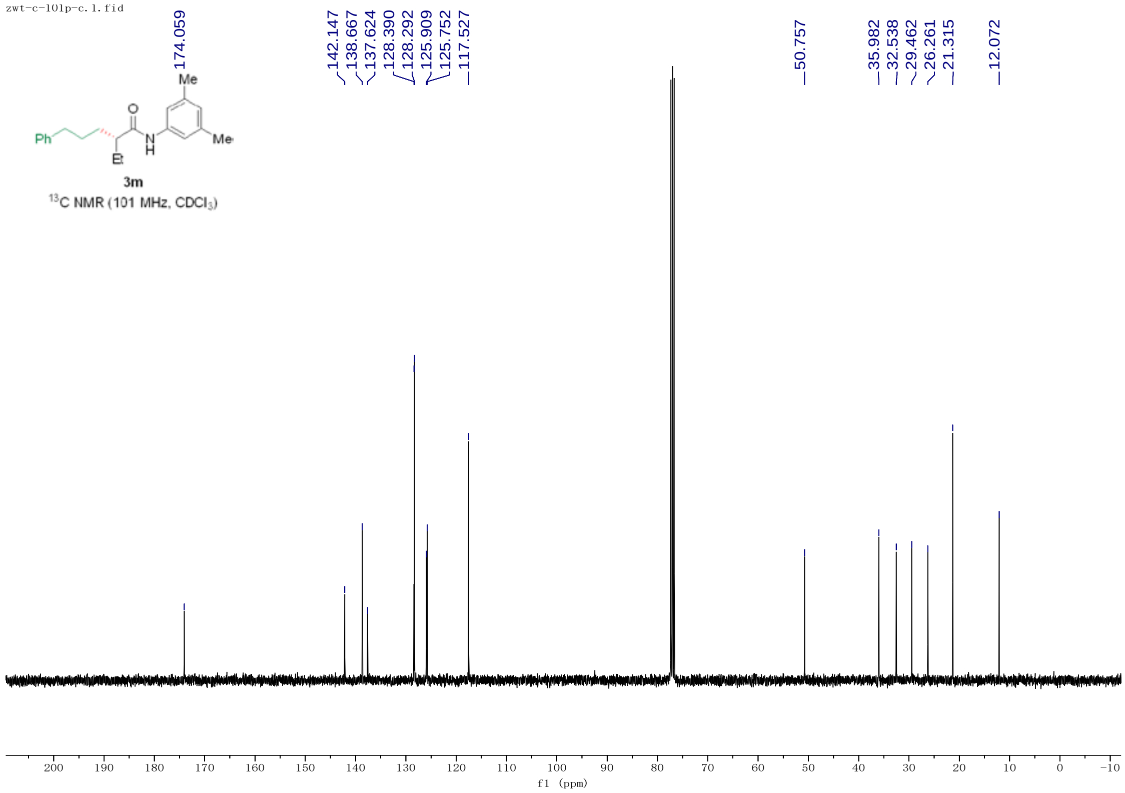

Supplementary Figure 119 ^{1}H-NMR (400 Mz, $CHCl_3$, 25 °C) and ^{13}C-NMR (101 MHz, $CHCl_3$, 25 °C) spectra of **3m**

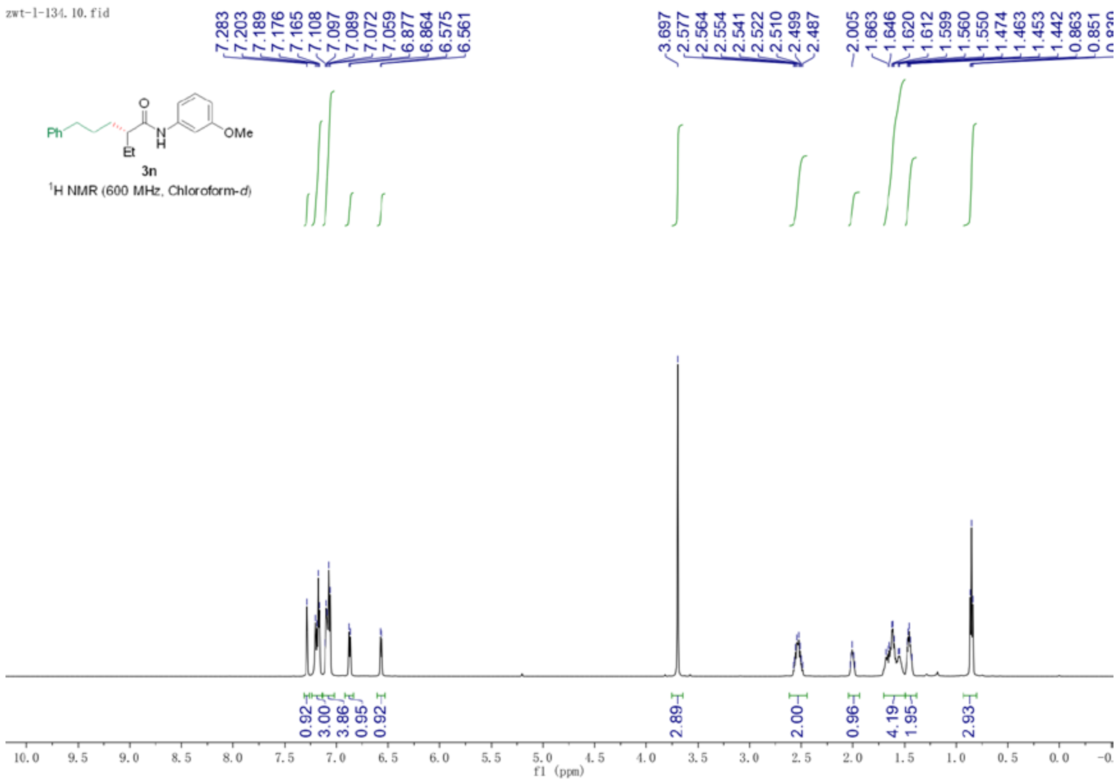

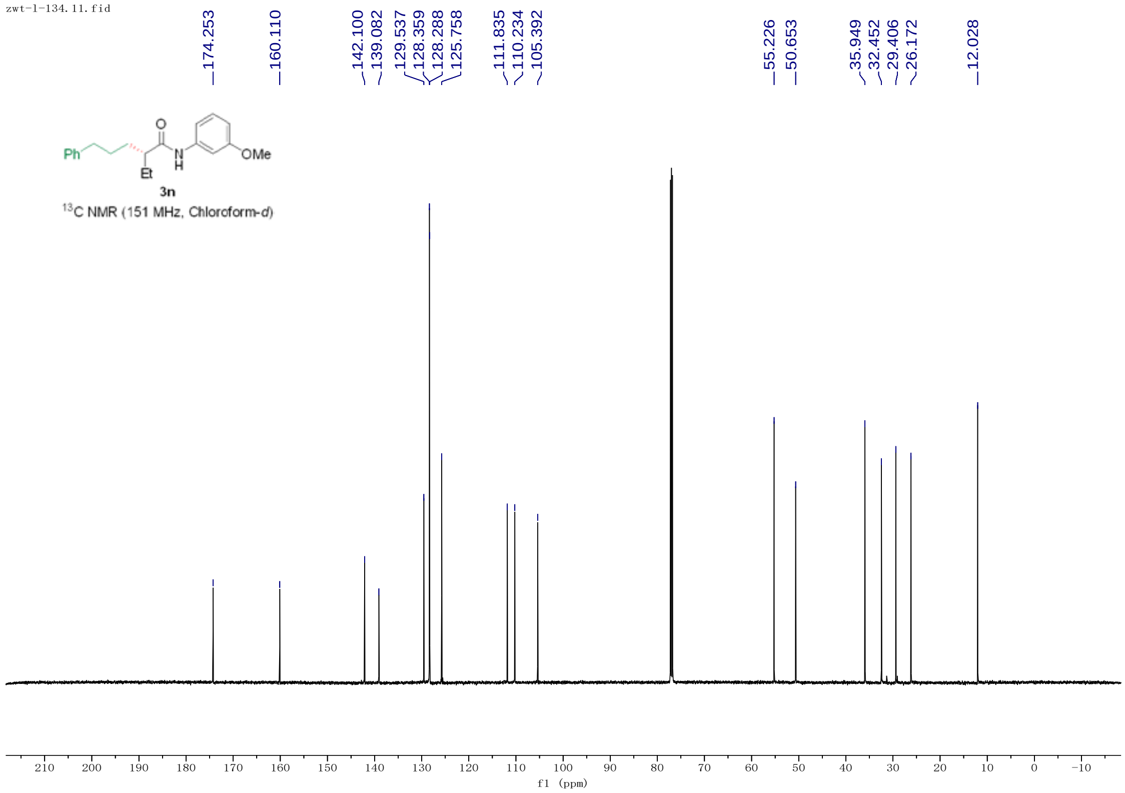

Supplementary Figure 120 ^{1}H-NMR (600 Mz, $CHCl_3$, 25 °C) and ^{13}C-NMR (151 MHz, $CHCl_3$, 25 °C) spectra of **3n**

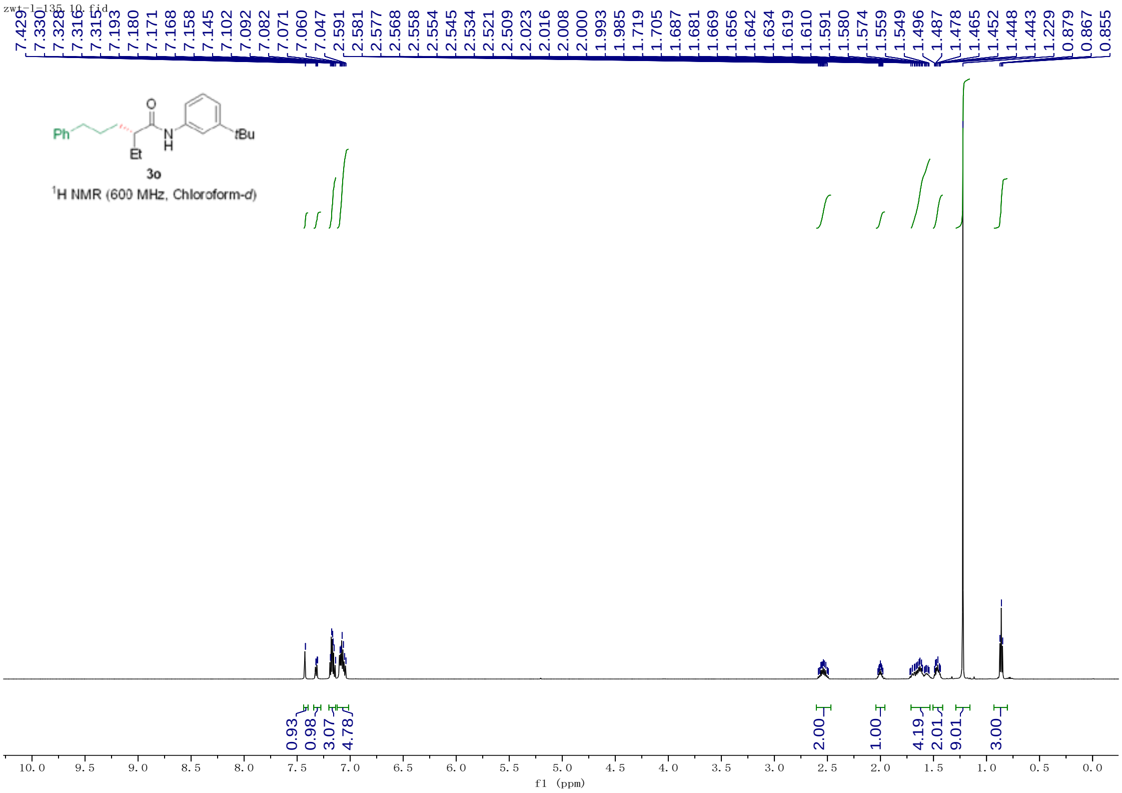

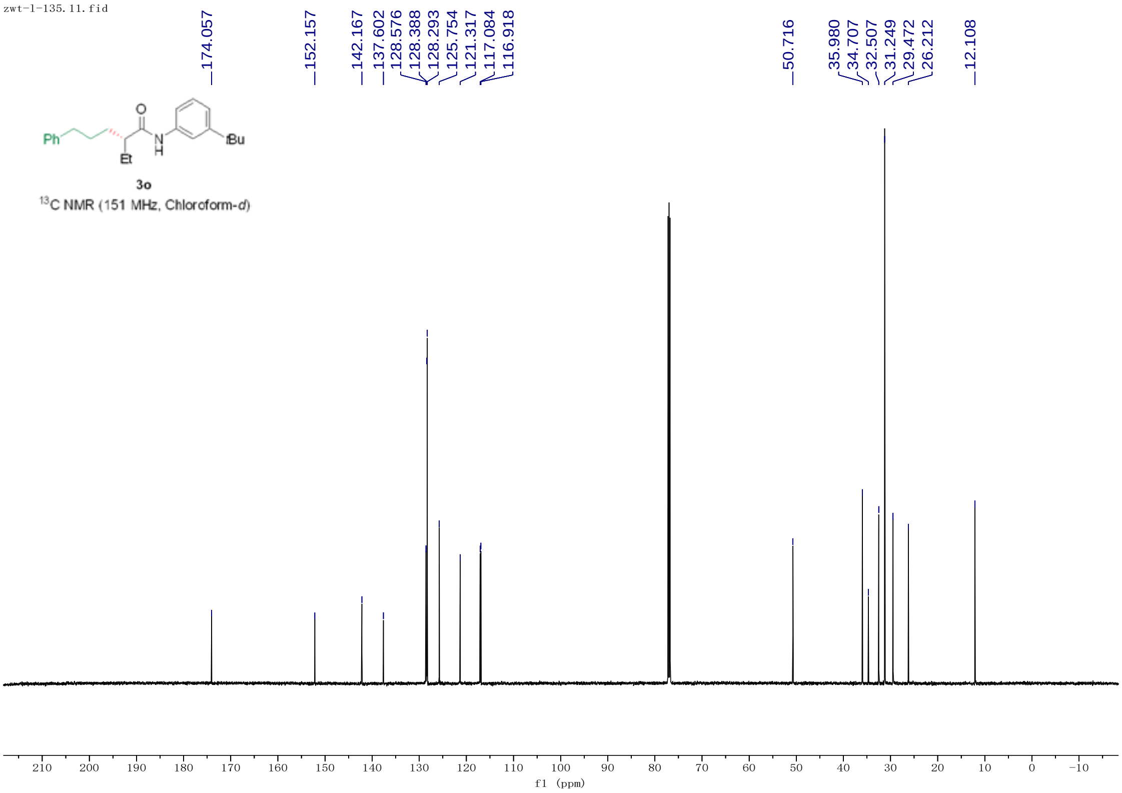

Supplementary Figure 121 ^{1}H-NMR (600 Mz, $CHCl_3$, 25 °C) and ^{13}C-NMR (151 MHz, $CHCl_3$, 25 °C) spectra of **3o**

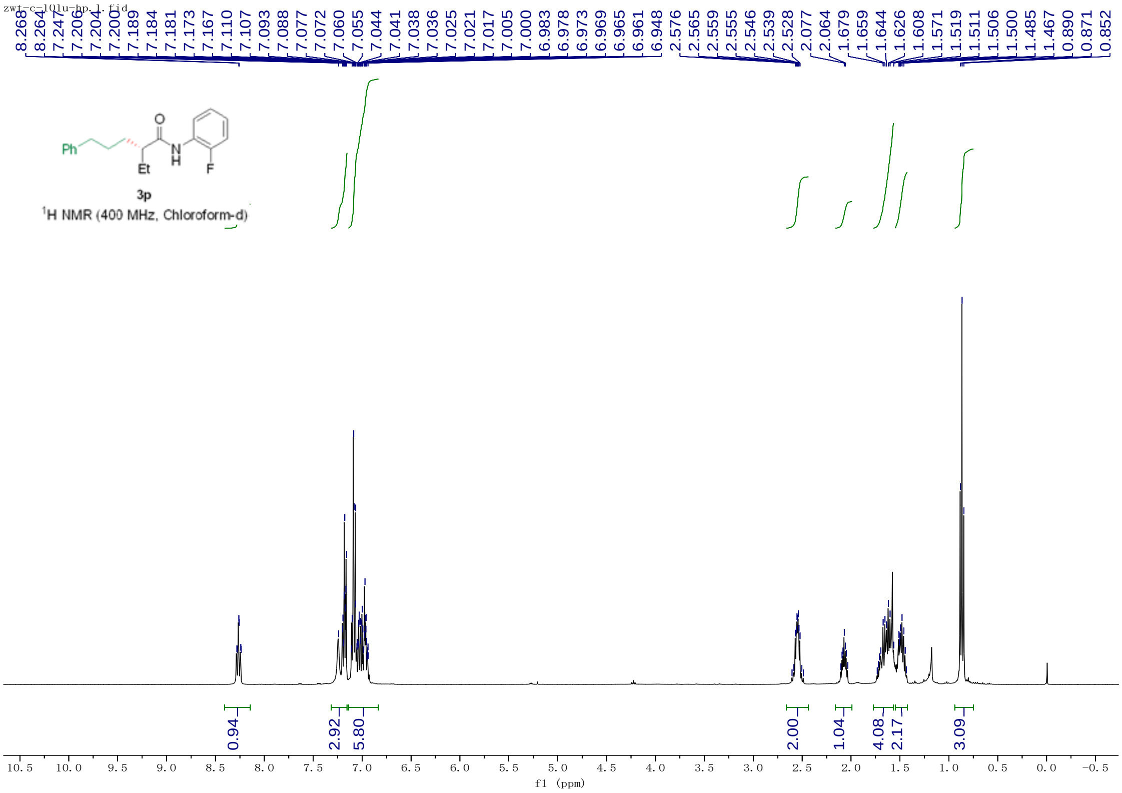

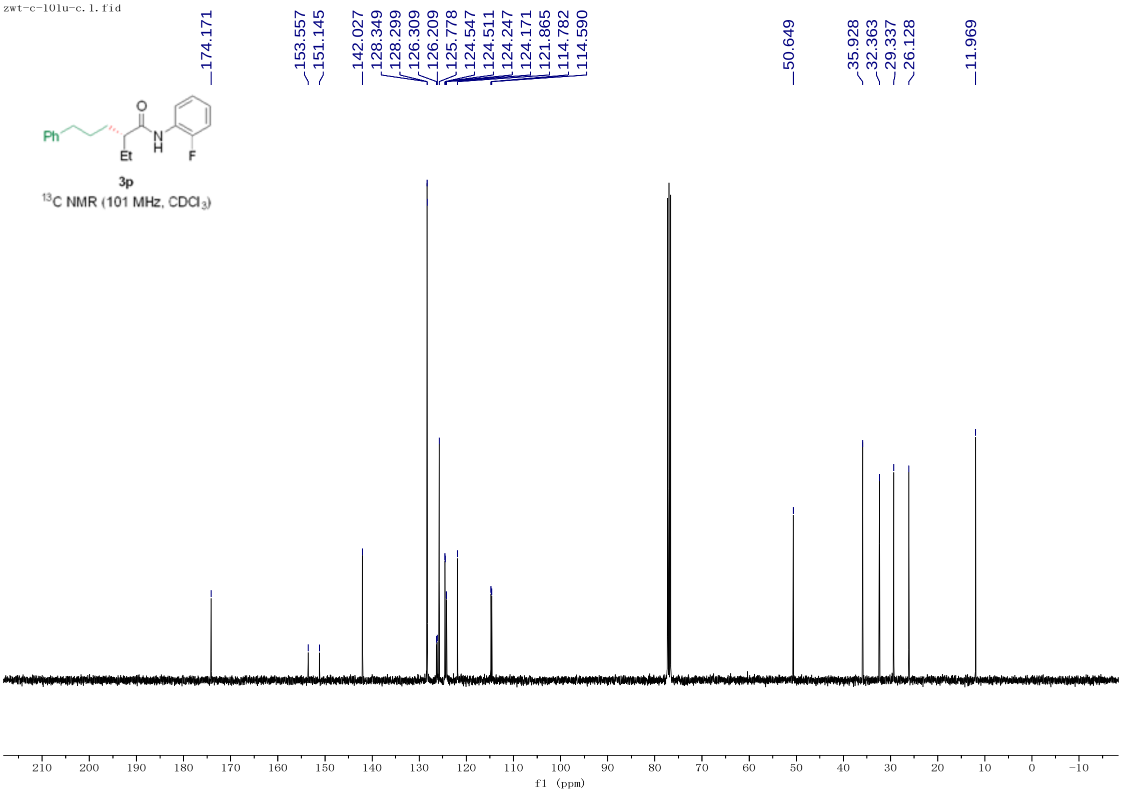

Supplementary Figure 122 ^{1}H-NMR (400 Mz, $CHCl_3$, 25 °C) and ^{13}C-NMR (101 MHz, $CHCl_3$, 25 °C) spectra of **3p**

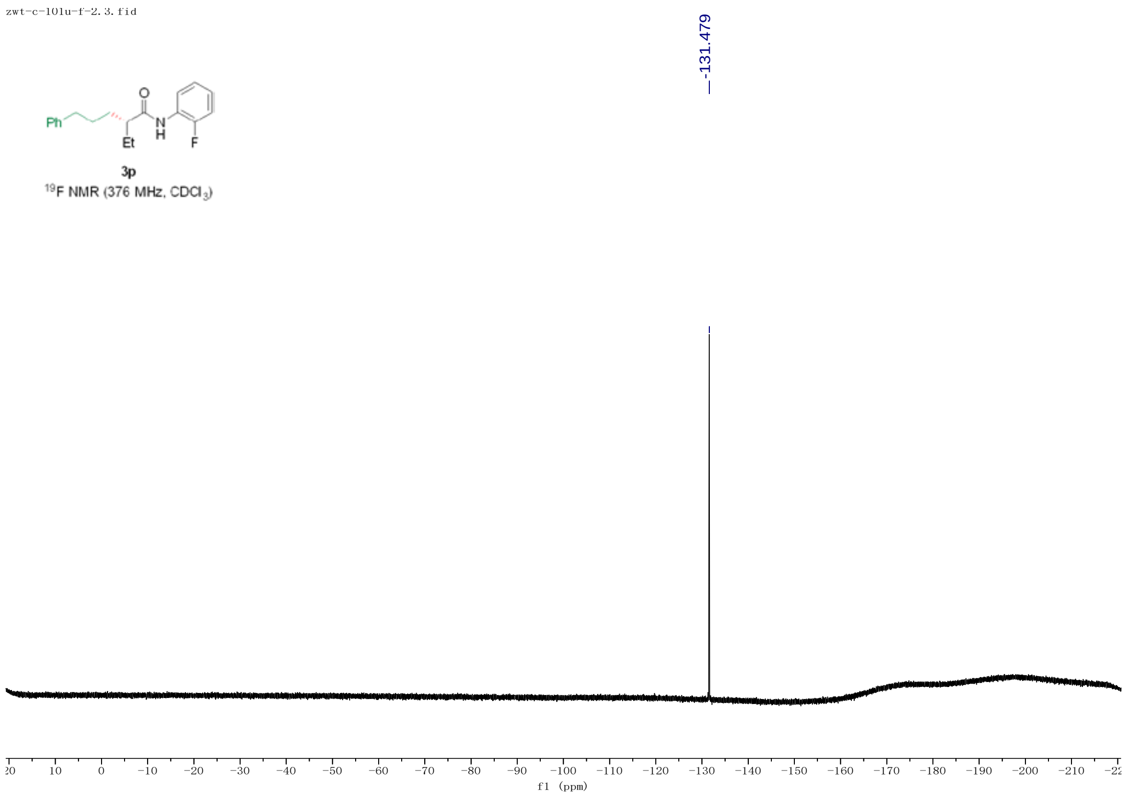

Supplementary Figure 123 ^{19}F-NMR (376 Mz, $CHCl_3$, 25 ℃) spectra of **3p**

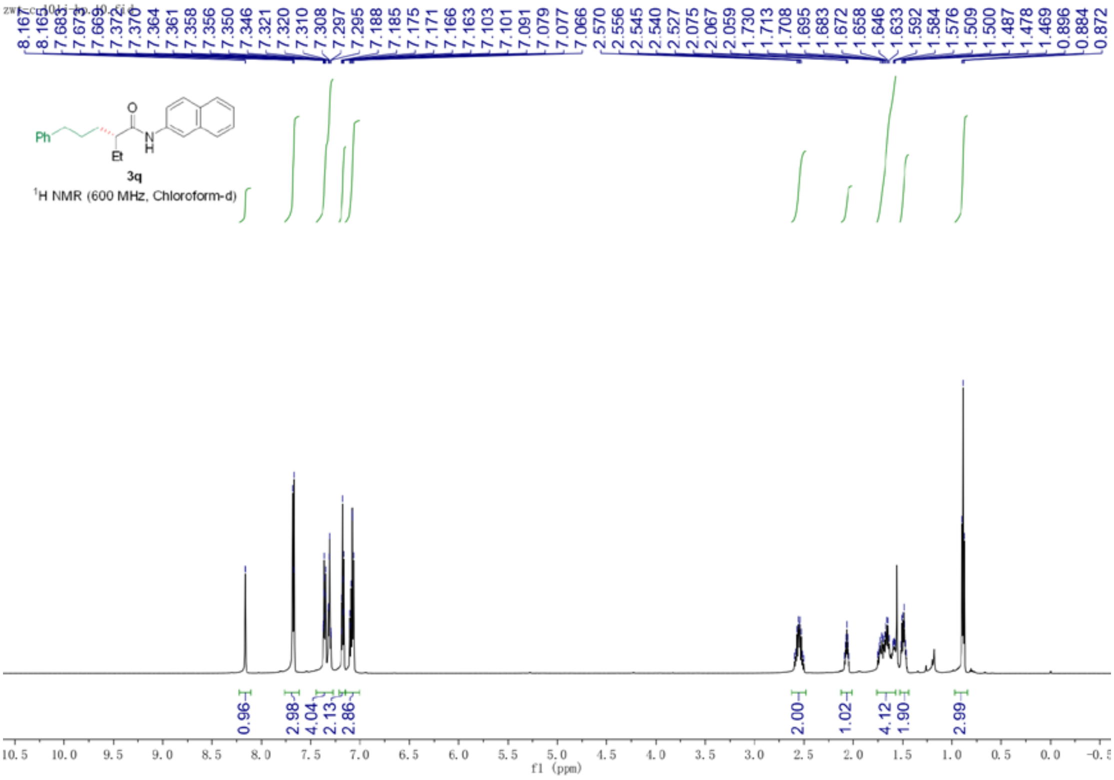

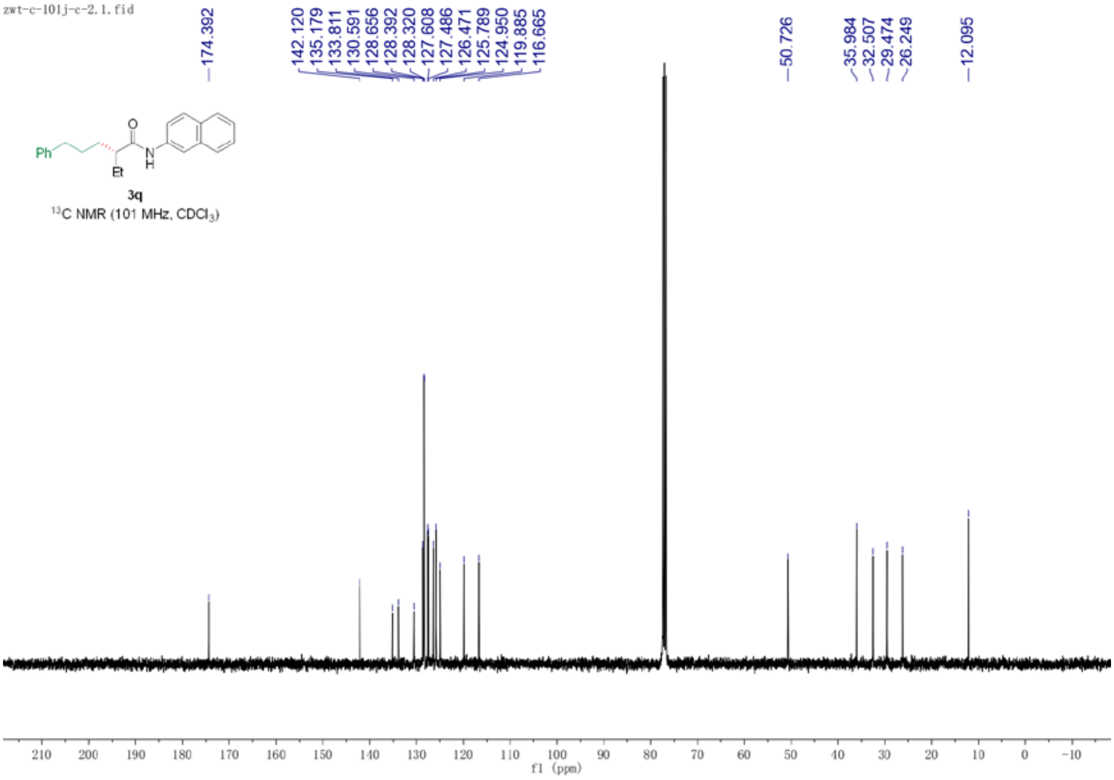

Supplementary Figure 124 ^{1}H-NMR (600 Mz, $CHCl_3$, 25 °C) and ^{13}C-NMR (101 MHz, $CHCl_3$, 25 °C) spectra of **3q**

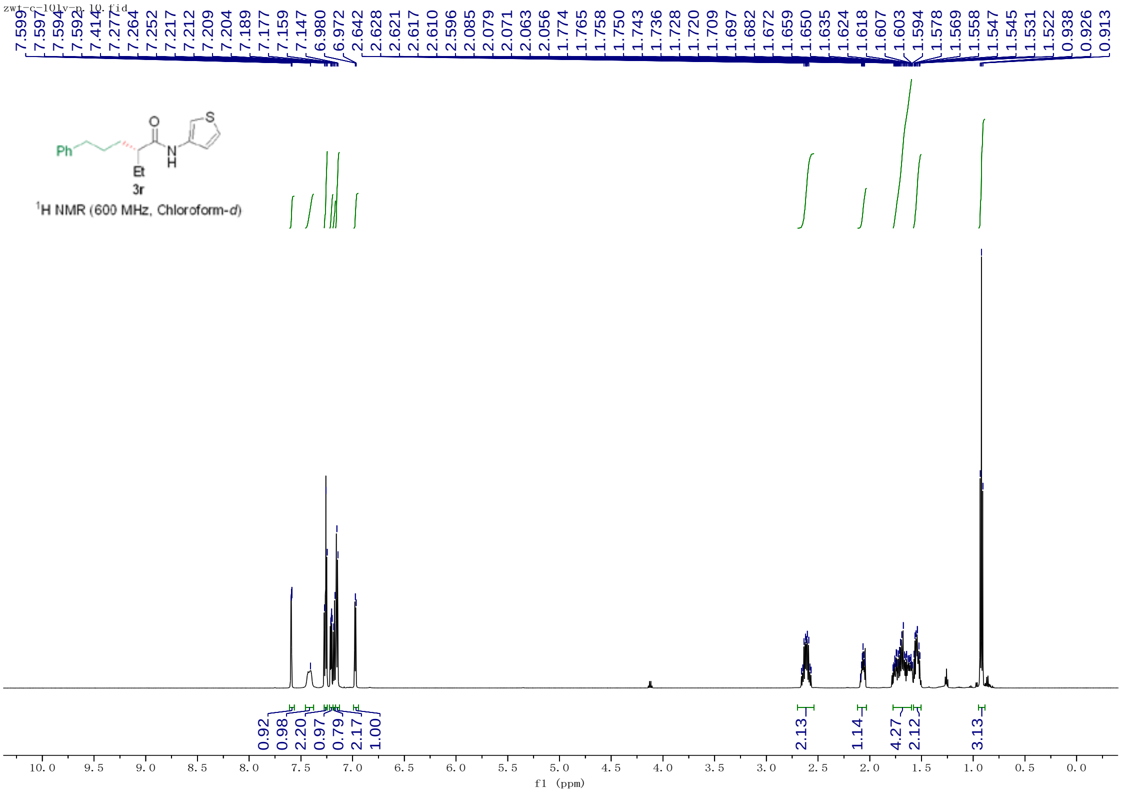

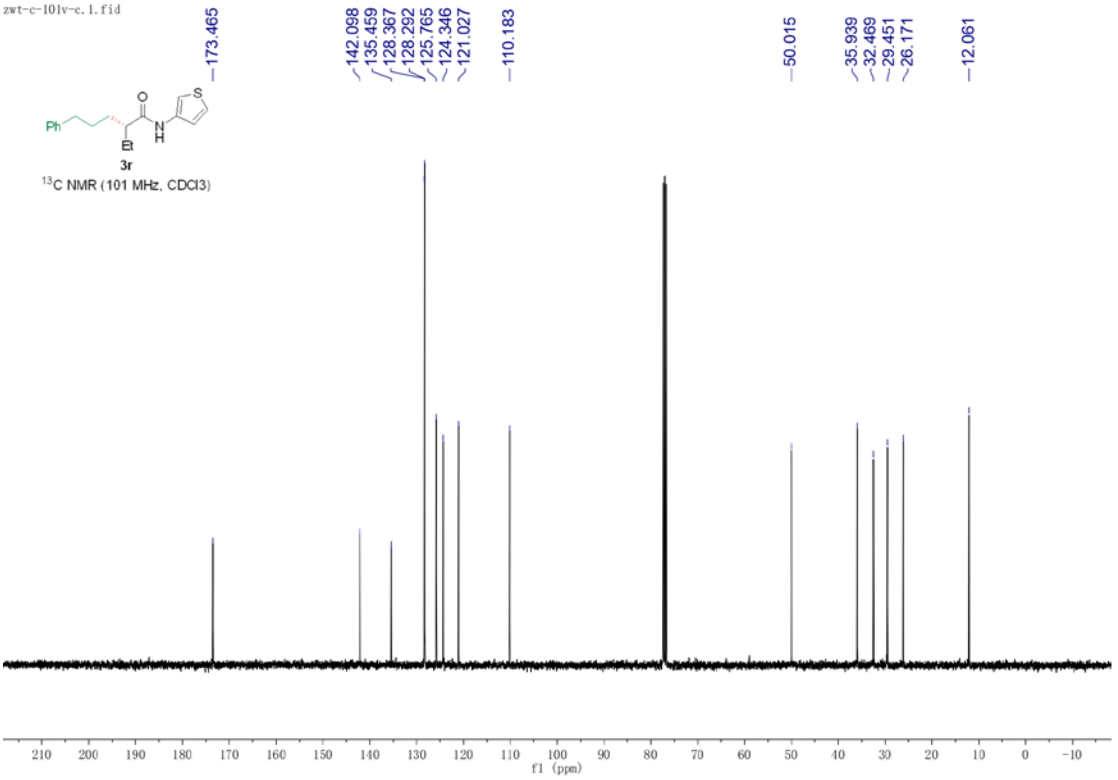

Supplementary Figure 125 ^{1}H-NMR (600 Mz, $CHCl_3$, 25 °C) and ^{13}C-NMR (101 MHz, $CHCl_3$, 25 °C) spectra of **3r**

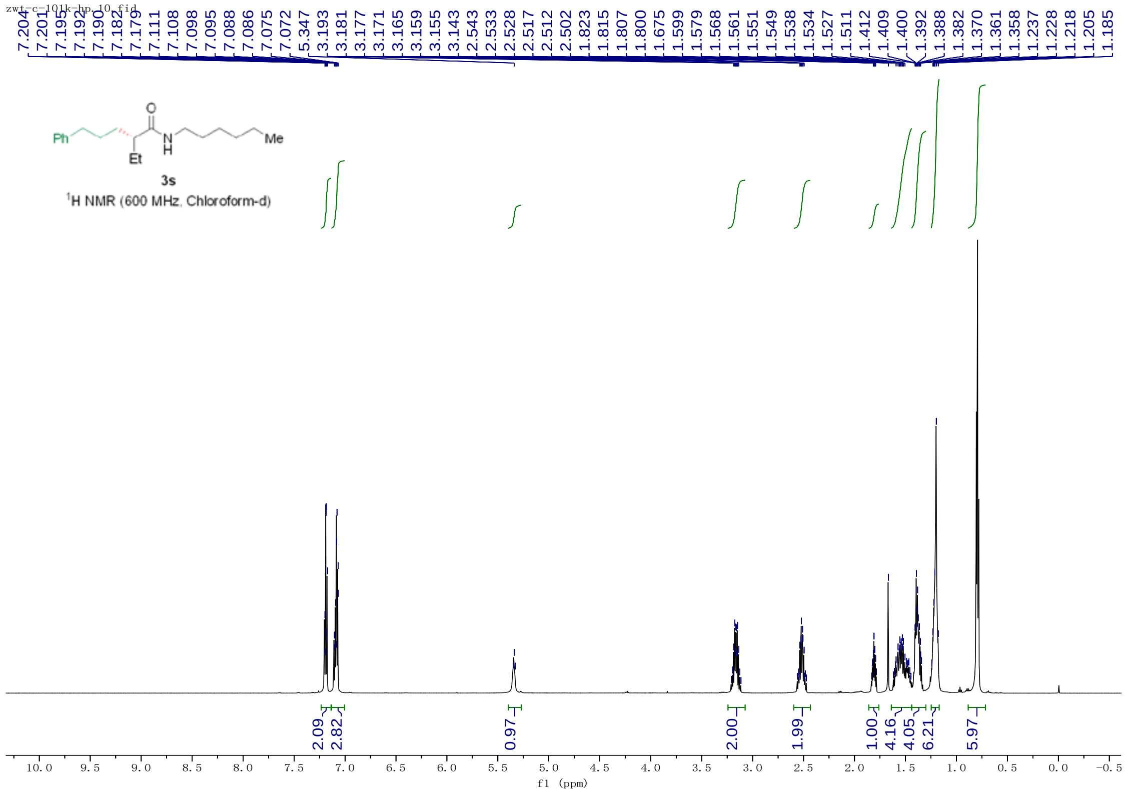

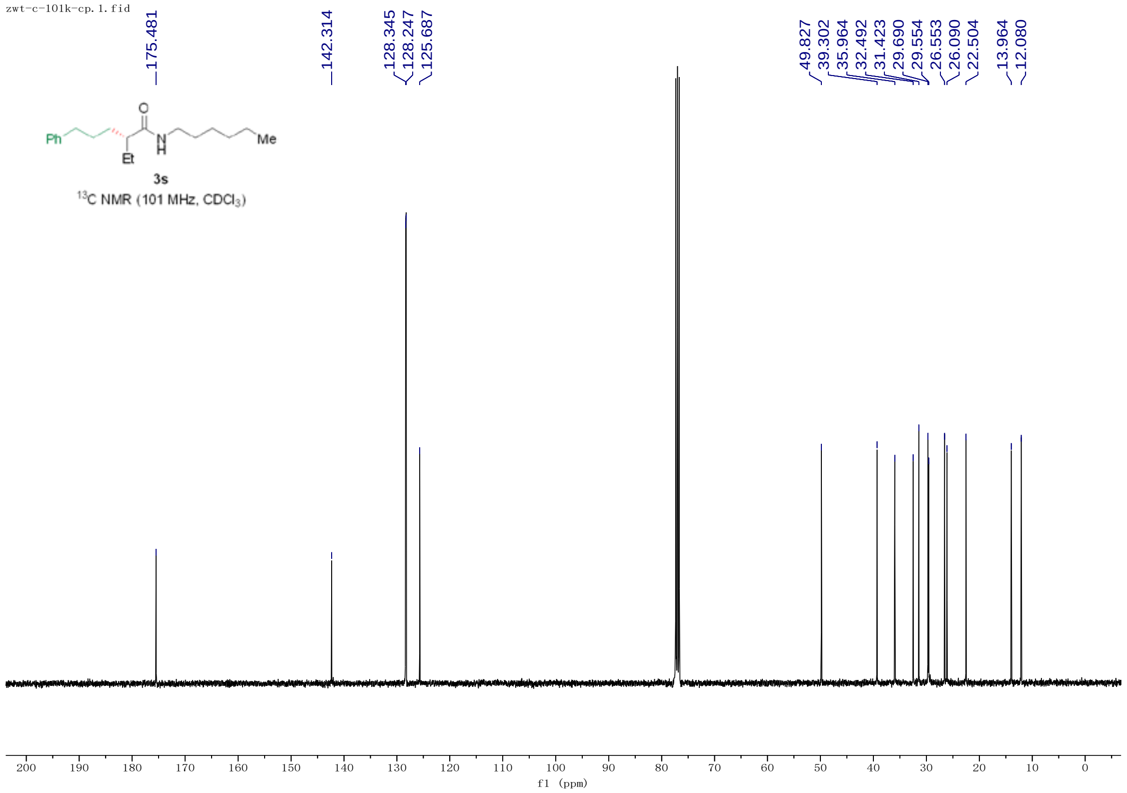

Supplementary Figure 126 ^{1}H-NMR (600 Mz, $CHCl_3$, 25 °C) and ^{13}C-NMR (101 MHz, $CHCl_3$, 25 °C) spectra of **3s**

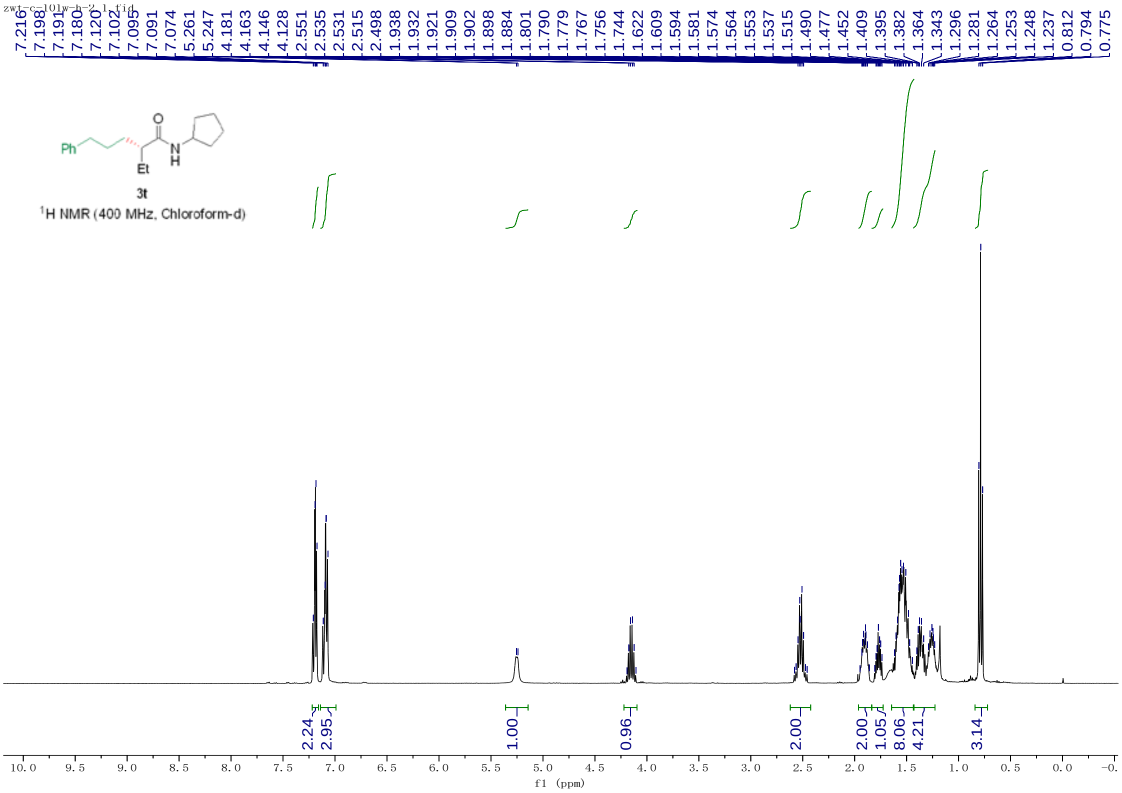

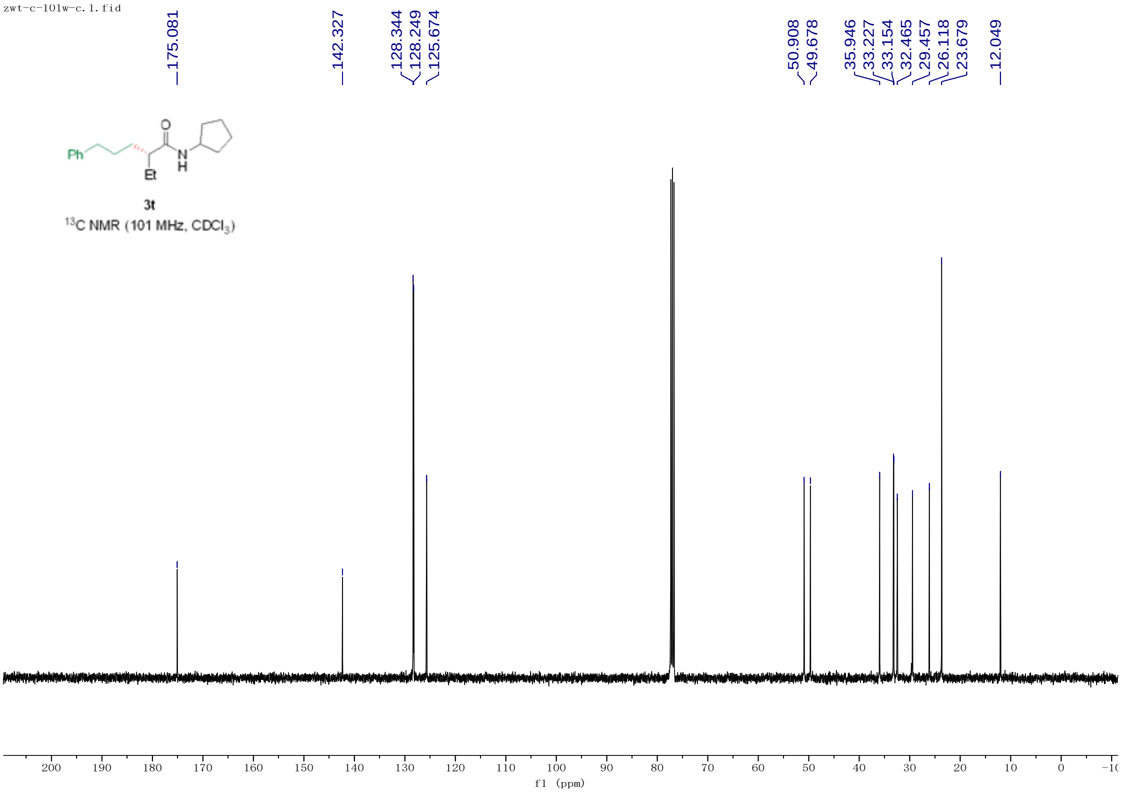

Supplementary Figure 127 ^{1}H-NMR (400 Mz, $CHCl_3$, 25 °C) and ^{13}C-NMR (101 MHz, $CHCl_3$, 25 °C) spectra of **3t**

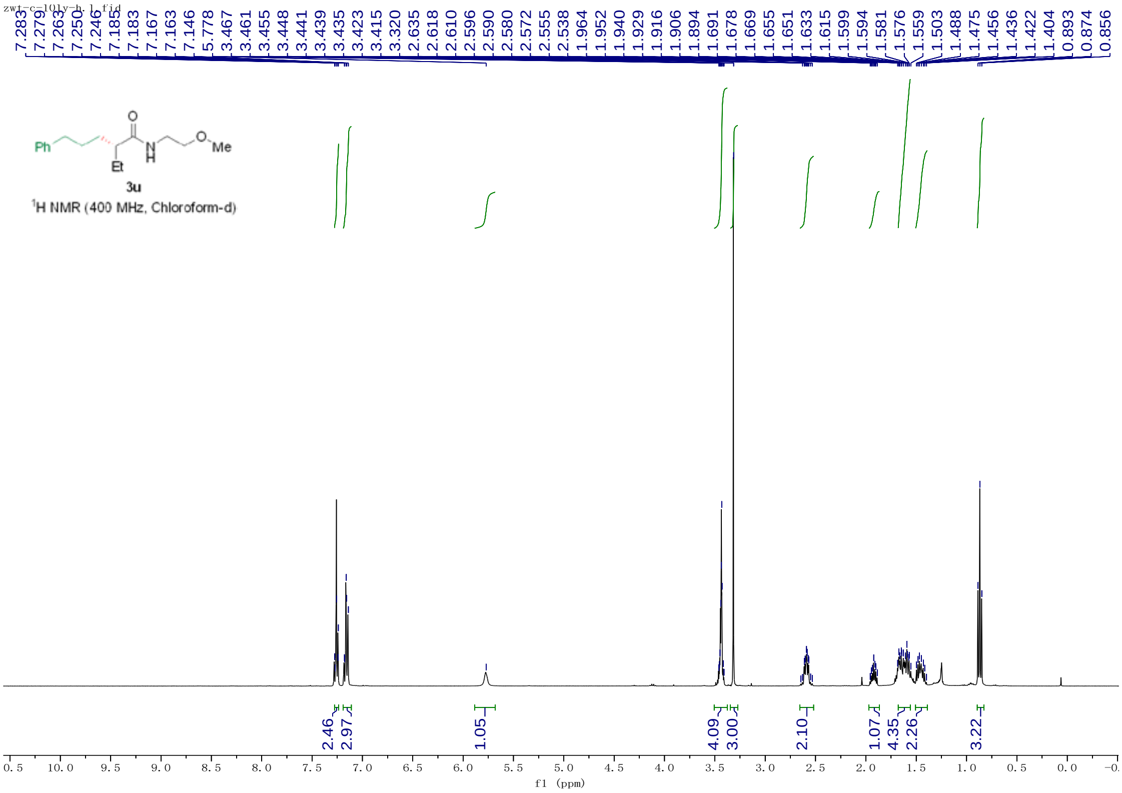

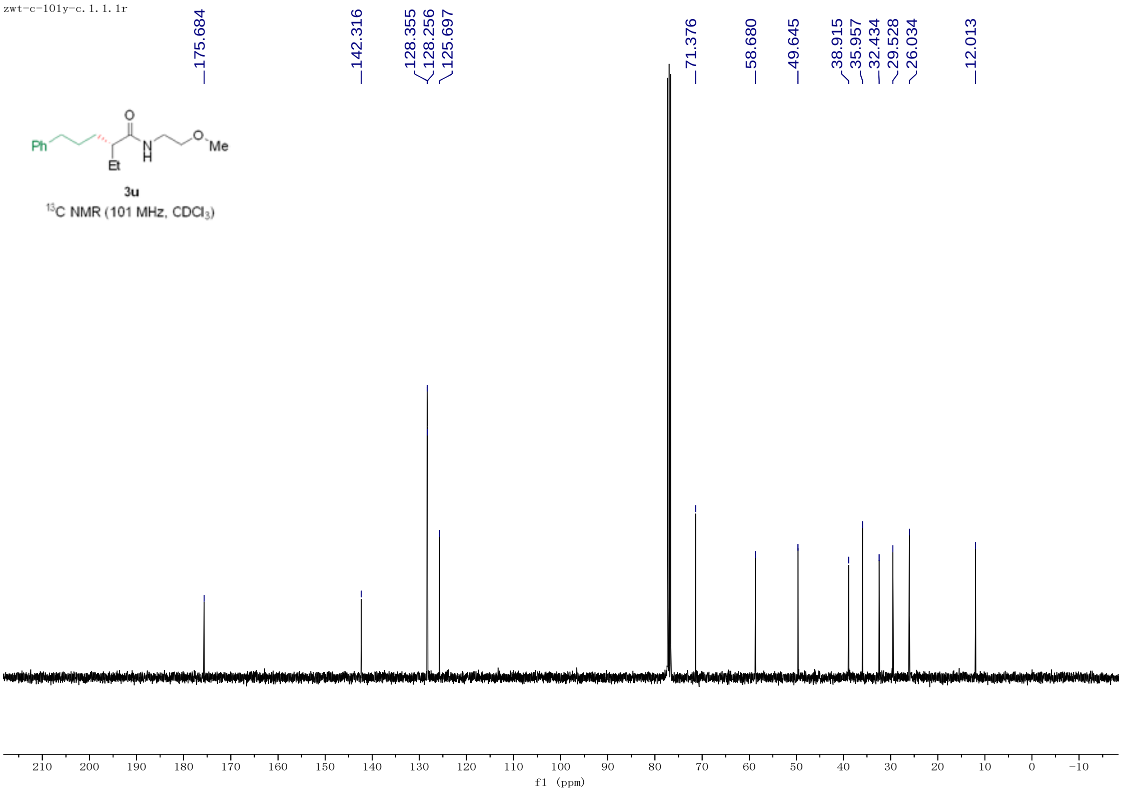

Supplementary Figure 128 ^{1}H-NMR (400 Mz, $CHCl_3$, 25 °C) and ^{13}C-NMR (101 MHz, $CHCl_3$, 25 °C) spectra of **3u**

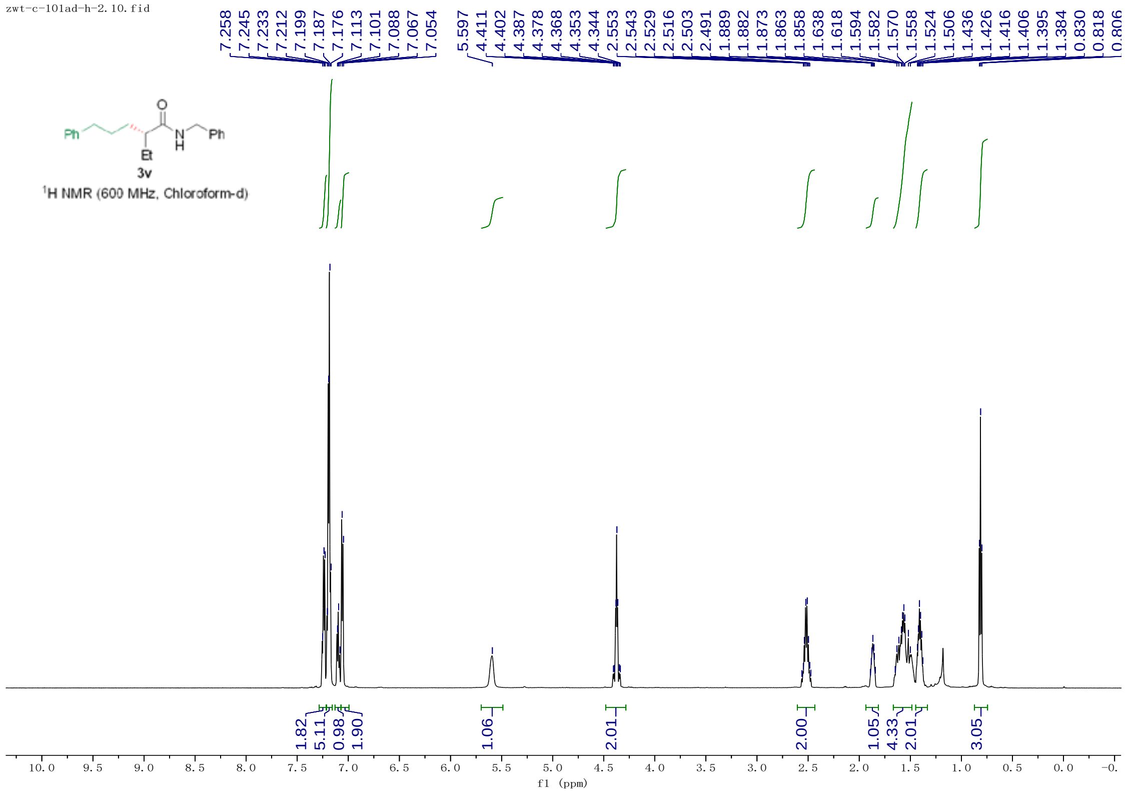

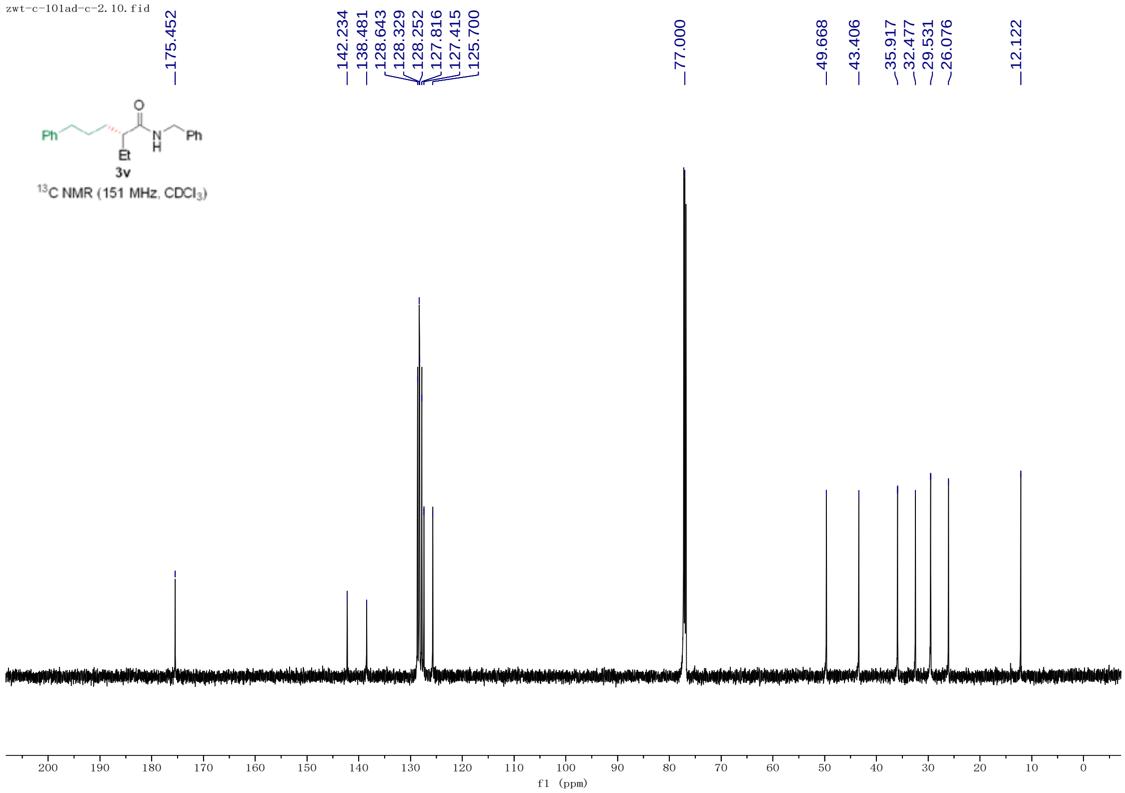

Supplementary Figure 129 ^{1}H-NMR (600 Mz, $CHCl_3$, 25 °C) and ^{13}C-NMR (151 MHz, $CHCl_3$, 25 °C) spectra of **3v**

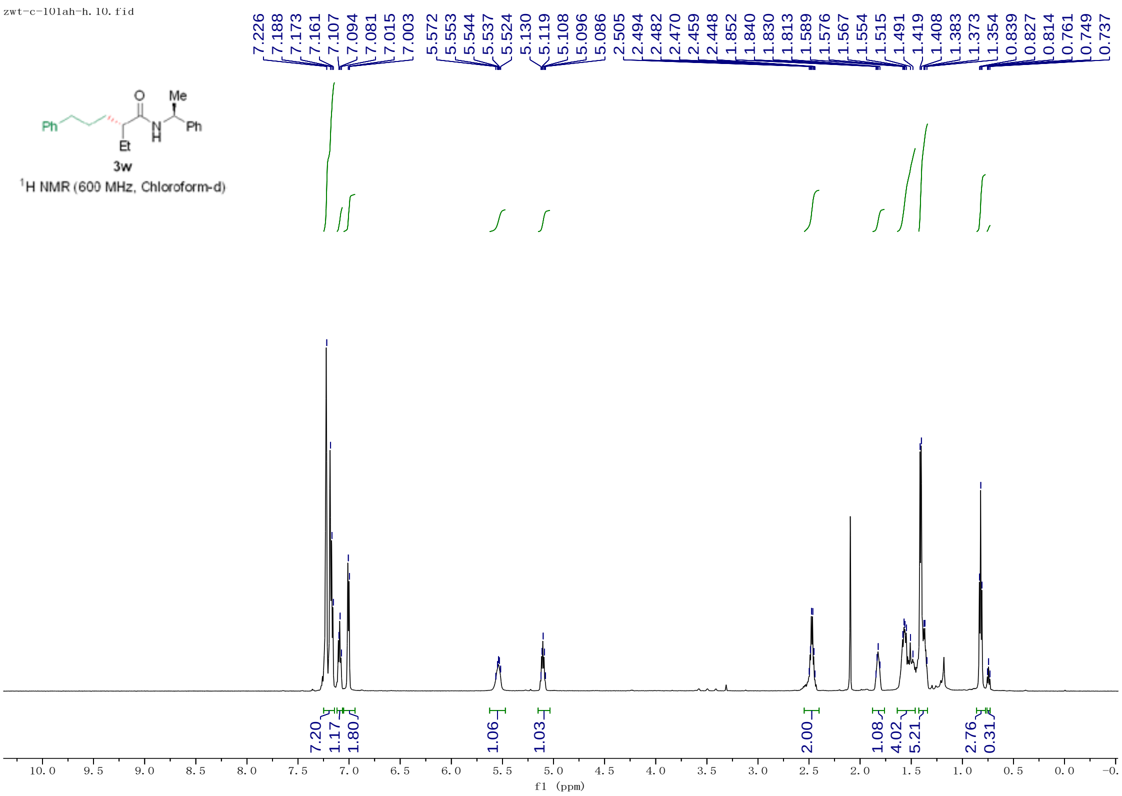

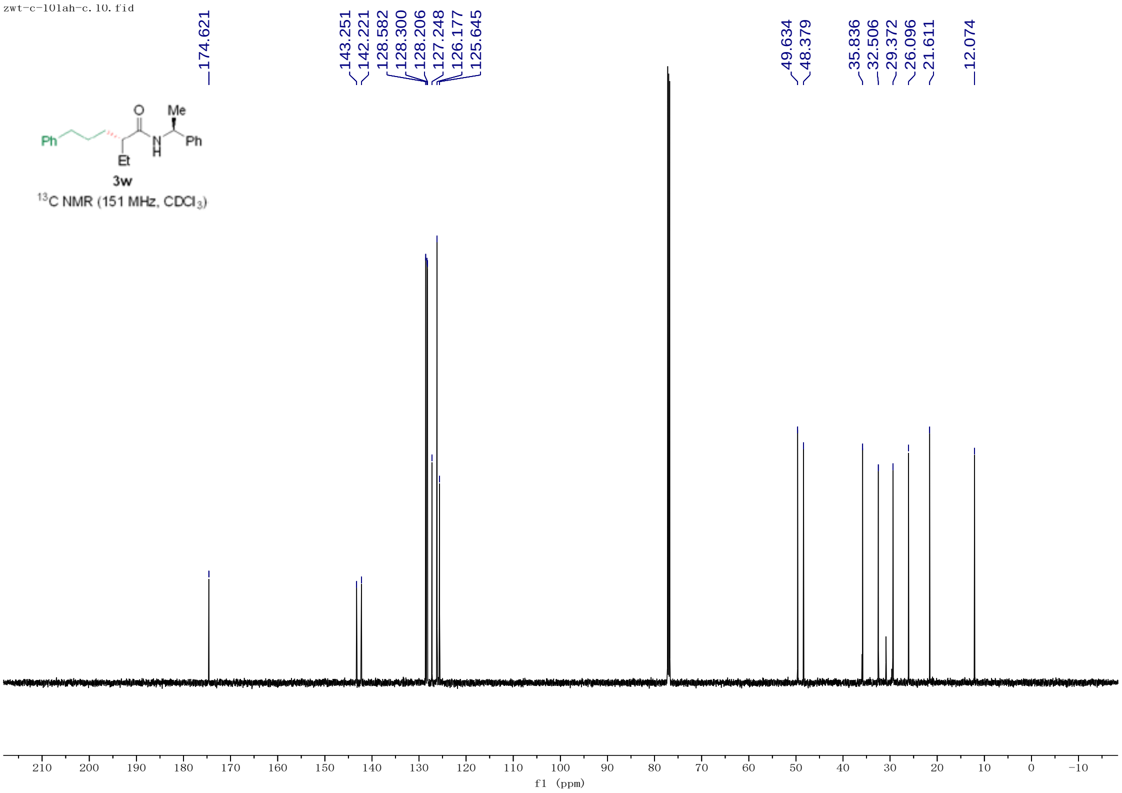

Supplementary Figure 130 ^{1}H-NMR (600 Mz, $CHCl_3$, 25 °C) and ^{13}C-NMR (151 MHz, $CHCl_3$, 25 °C) spectra of **3w**

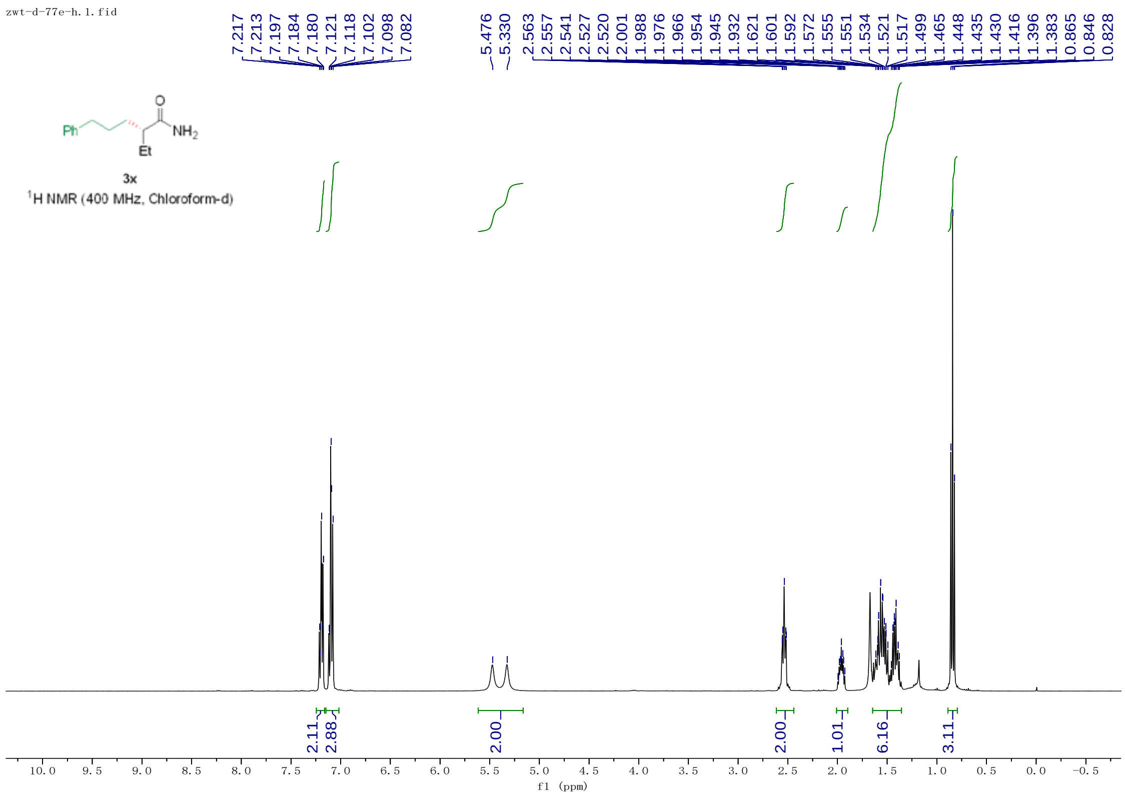

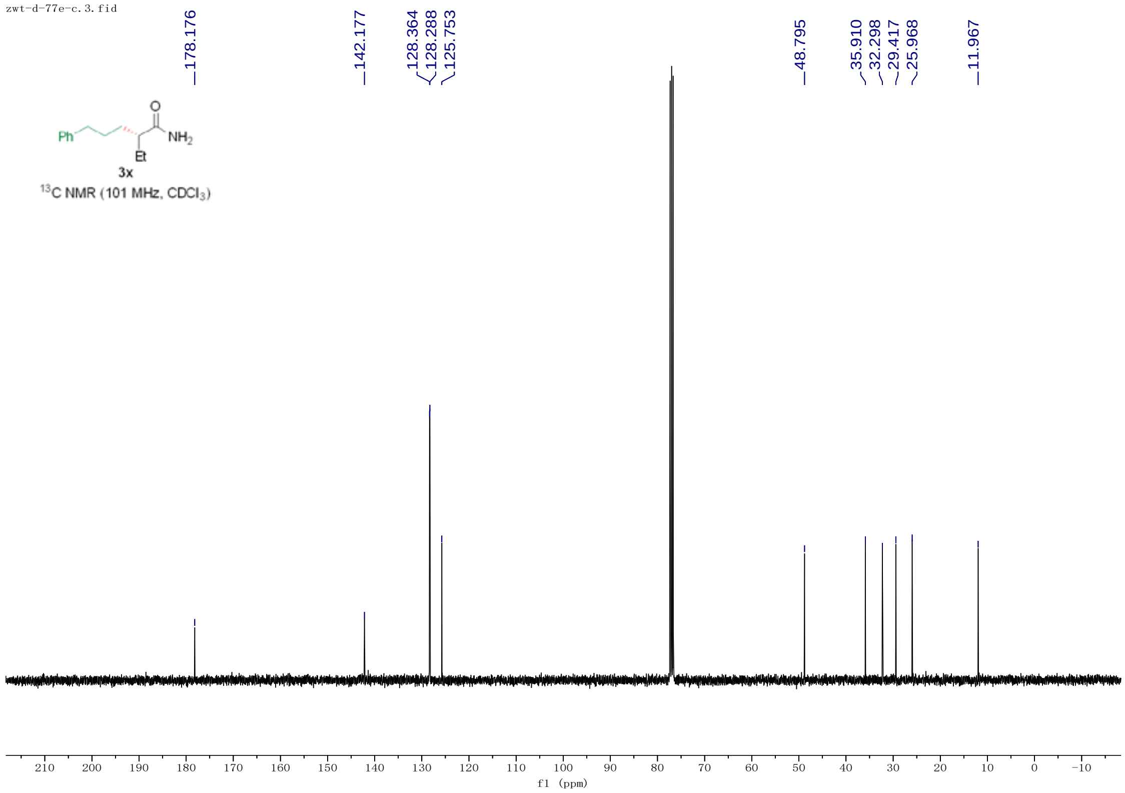

Supplementary Figure 131 ^{1}H-NMR (400 Mz, $CHCl_3$, 25 °C) and ^{13}C-NMR (101 MHz, $CHCl_3$, 25 °C) spectra of **3x**

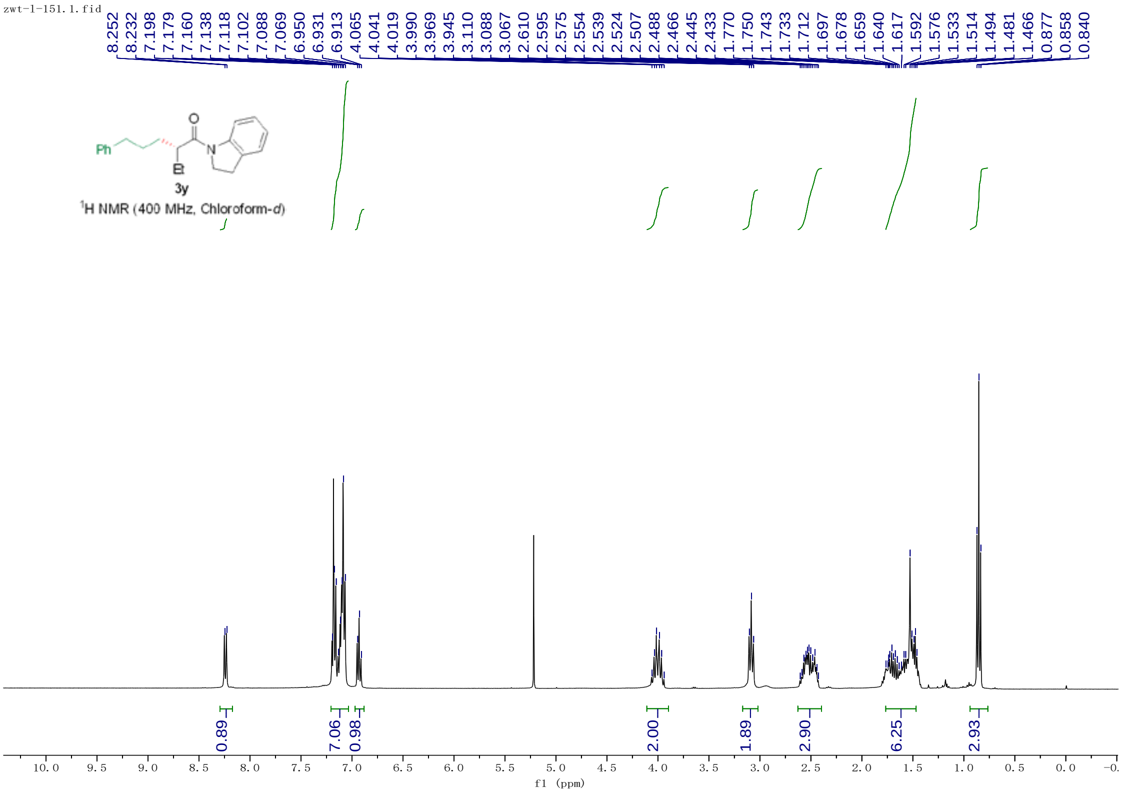

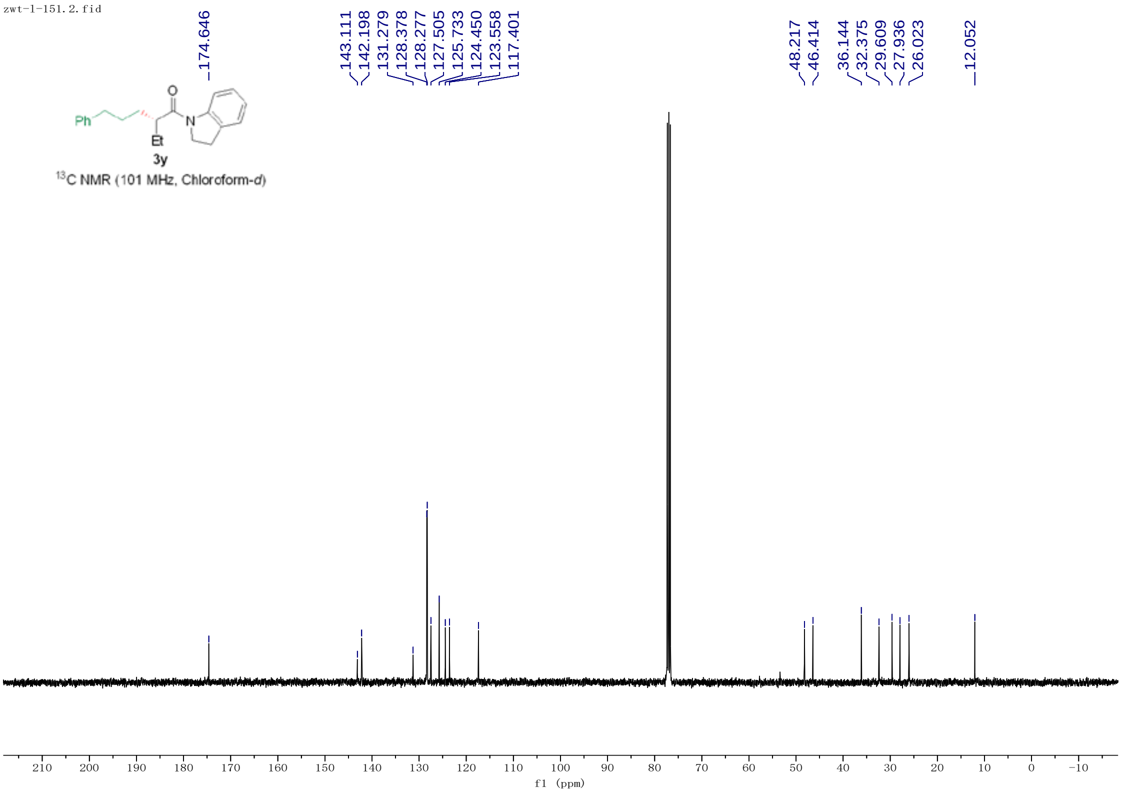

Supplementary Figure 132 ^{1}H-NMR (400 Mz, $CHCl_3$, 25 °C) and ^{13}C-NMR (101 MHz, $CHCl_3$, 25 °C) spectra of **3y**

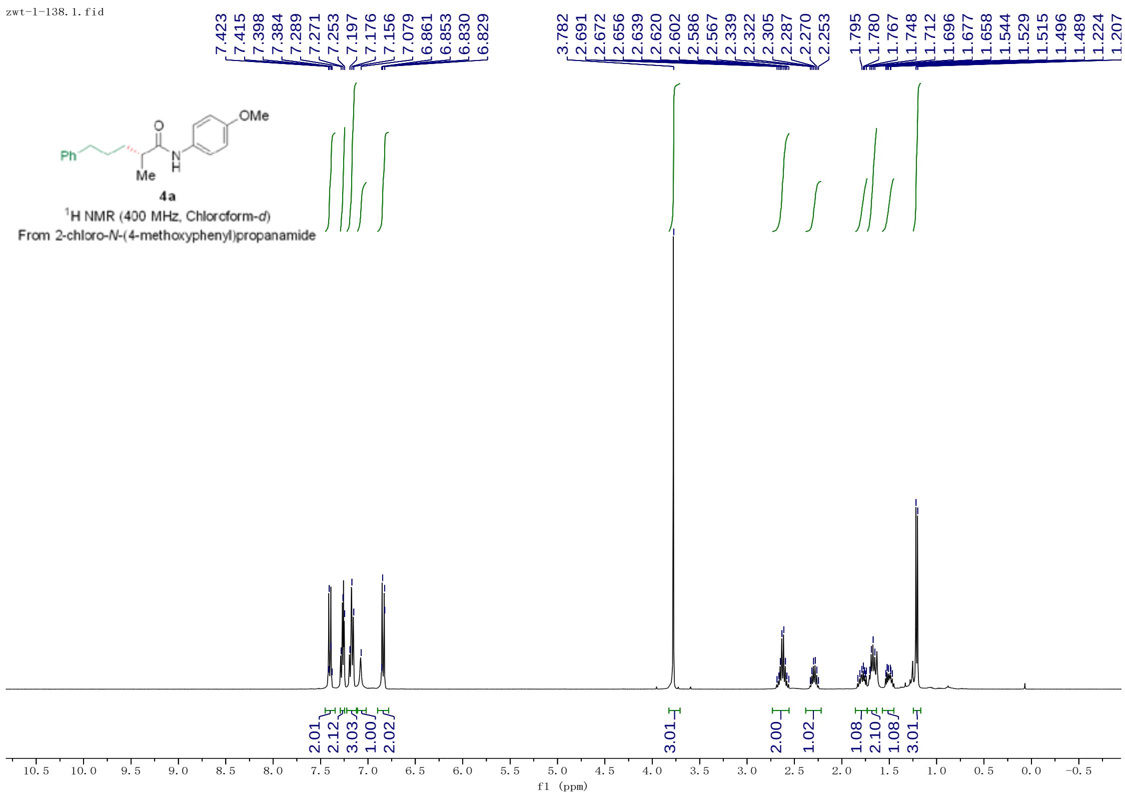

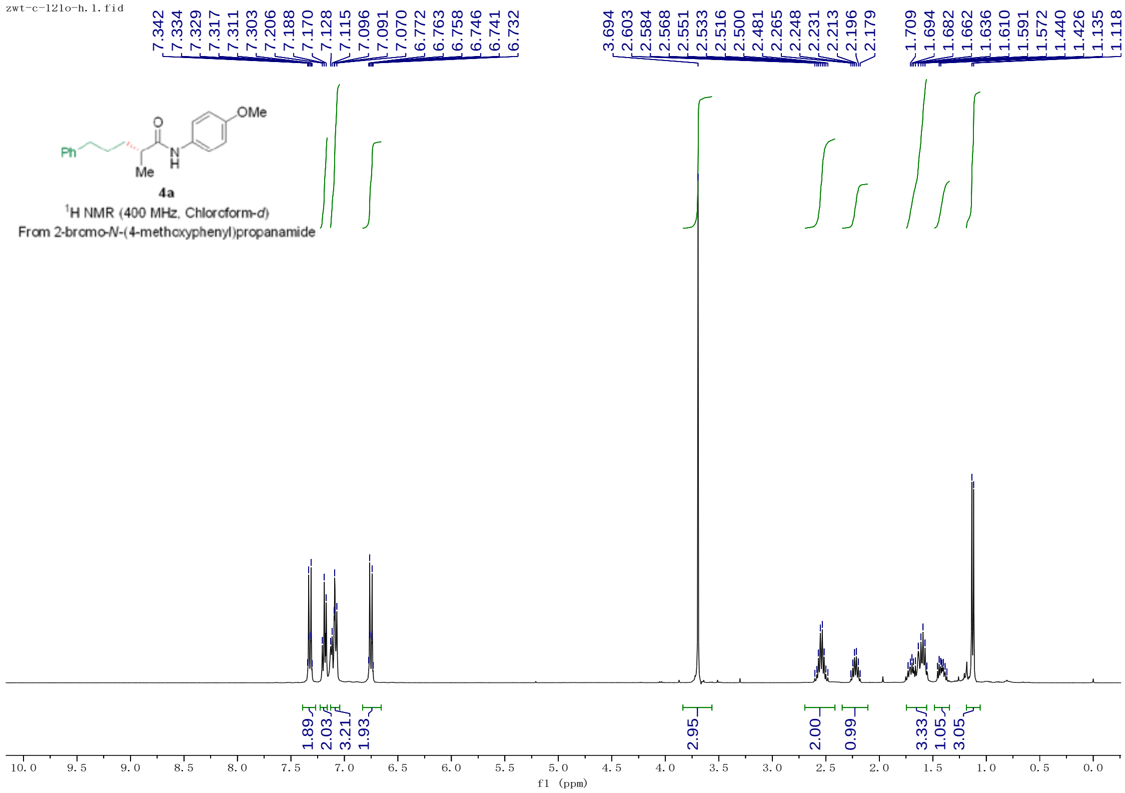

Supplementary Figure 133 ^{1}H-NMR (400 Mz, $CHCl_3$, 25 ℃) spectra of **4a**

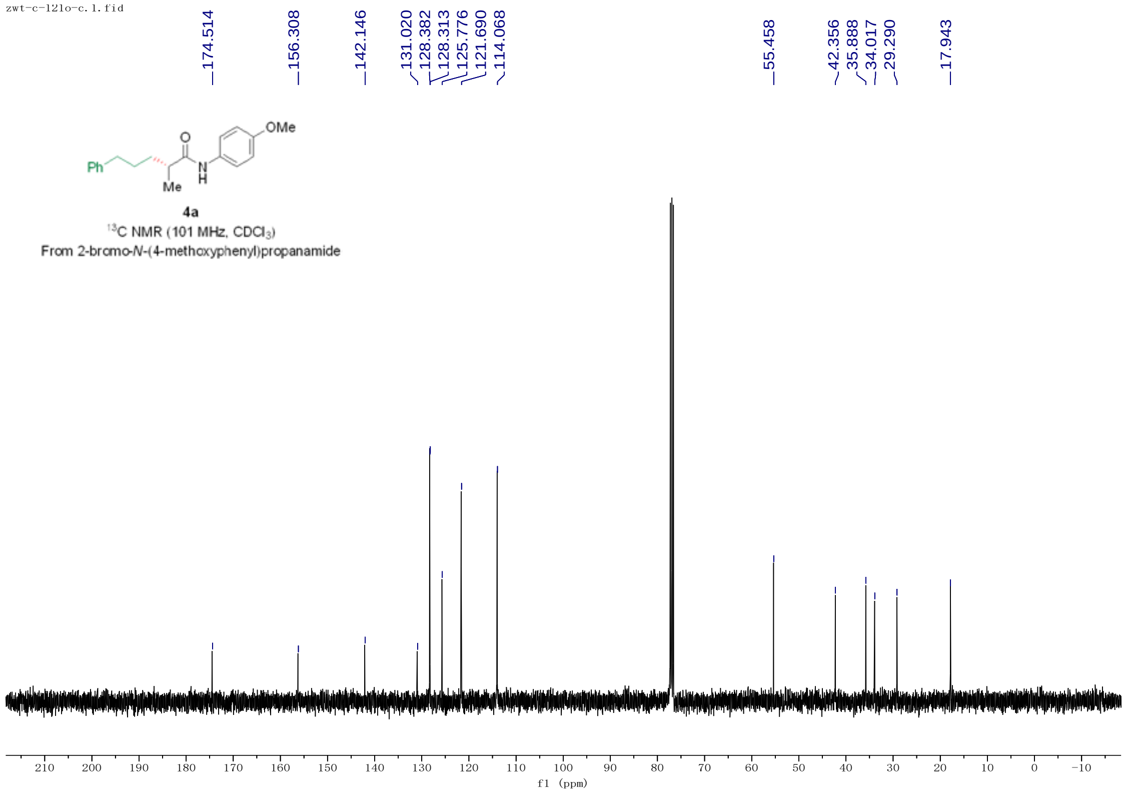

Supplementary Figure 134 ^{13}C-NMR (101 MHz, $CHCl_3$, 25 °C) spectra of **4a**

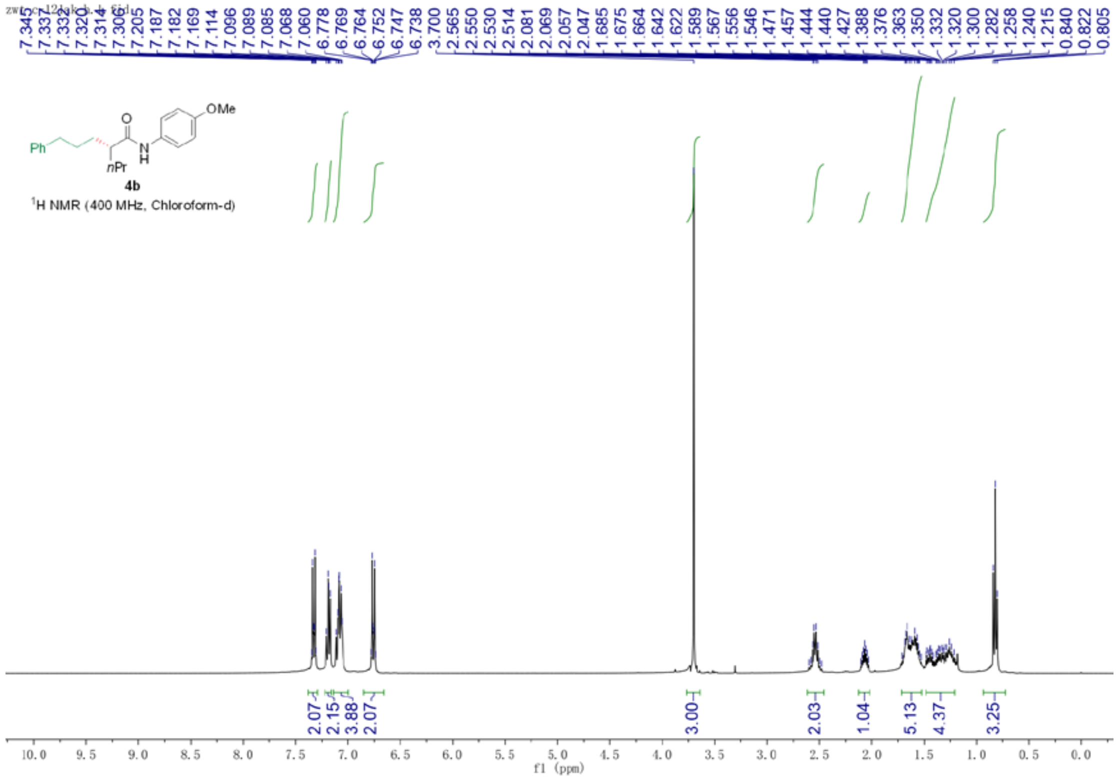

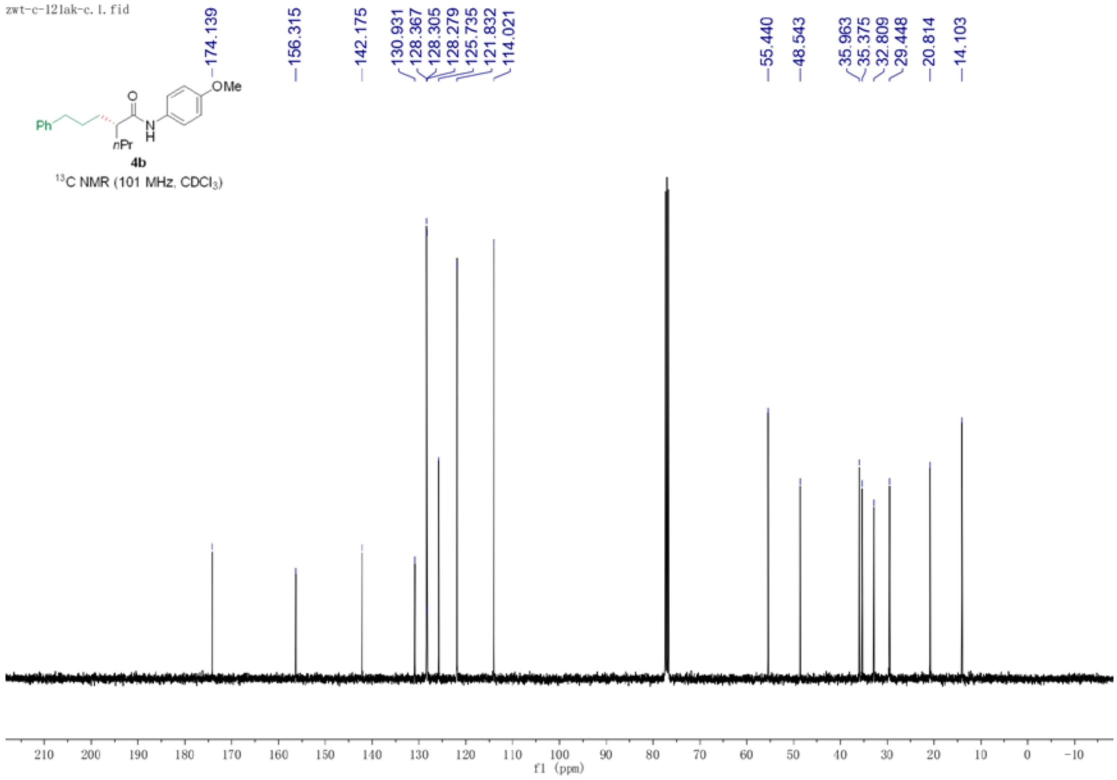

Supplementary Figure 135 ^{1}H-NMR (400 Mz, $CHCl_3$, 25 °C) and ^{13}C-NMR (101 MHz, $CHCl_3$, 25 °C) spectra of **4b**

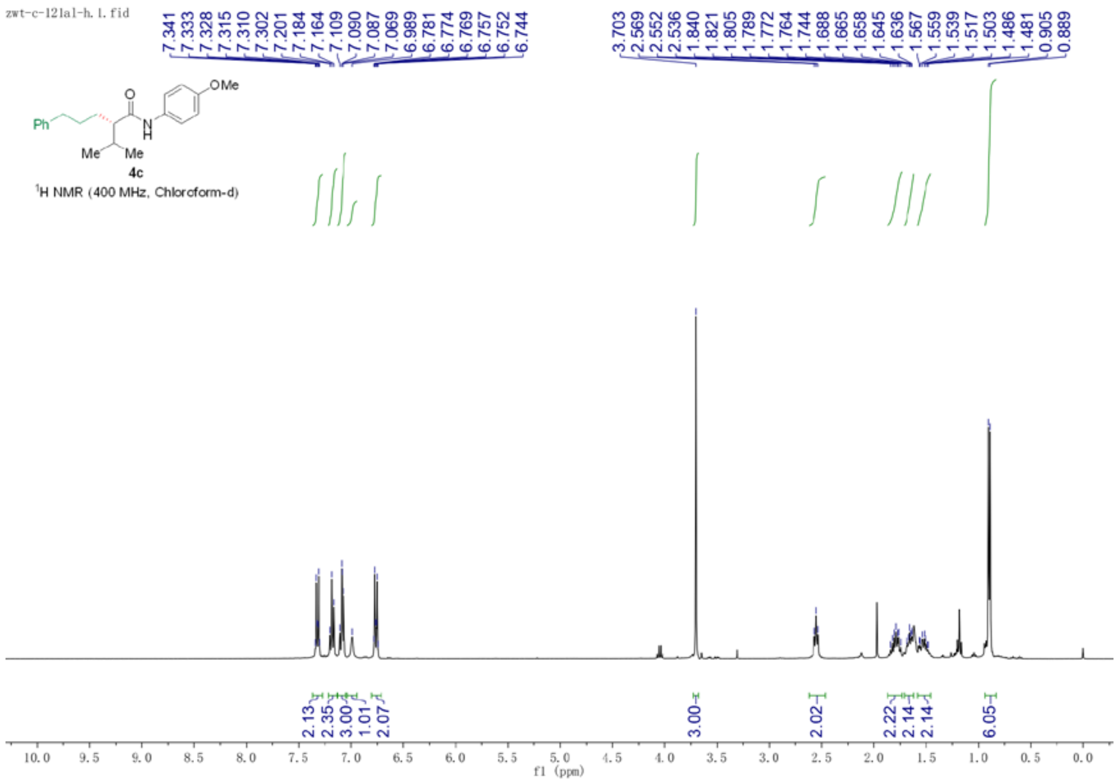

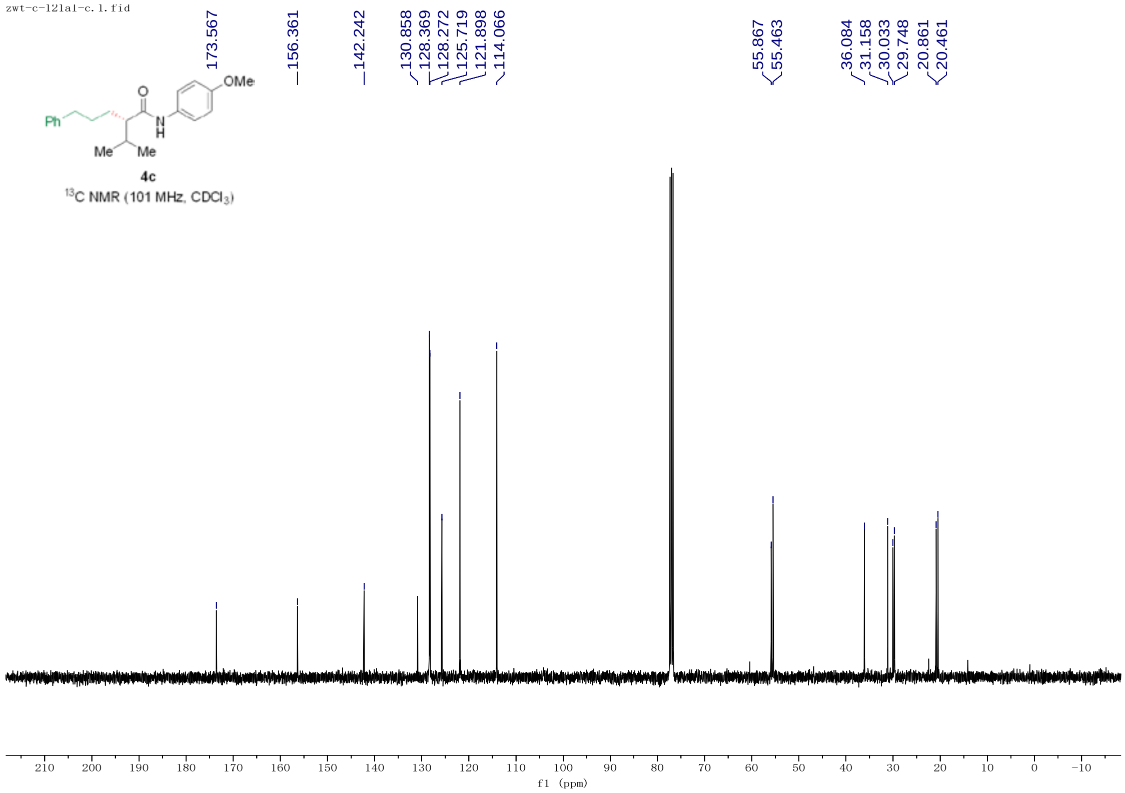

Supplementary Figure 136 ^{1}H-NMR (400 Mz, $CHCl_3$, 25 °C) and ^{13}C-NMR (101 MHz, $CHCl_3$, 25 °C) spectra of **4c**

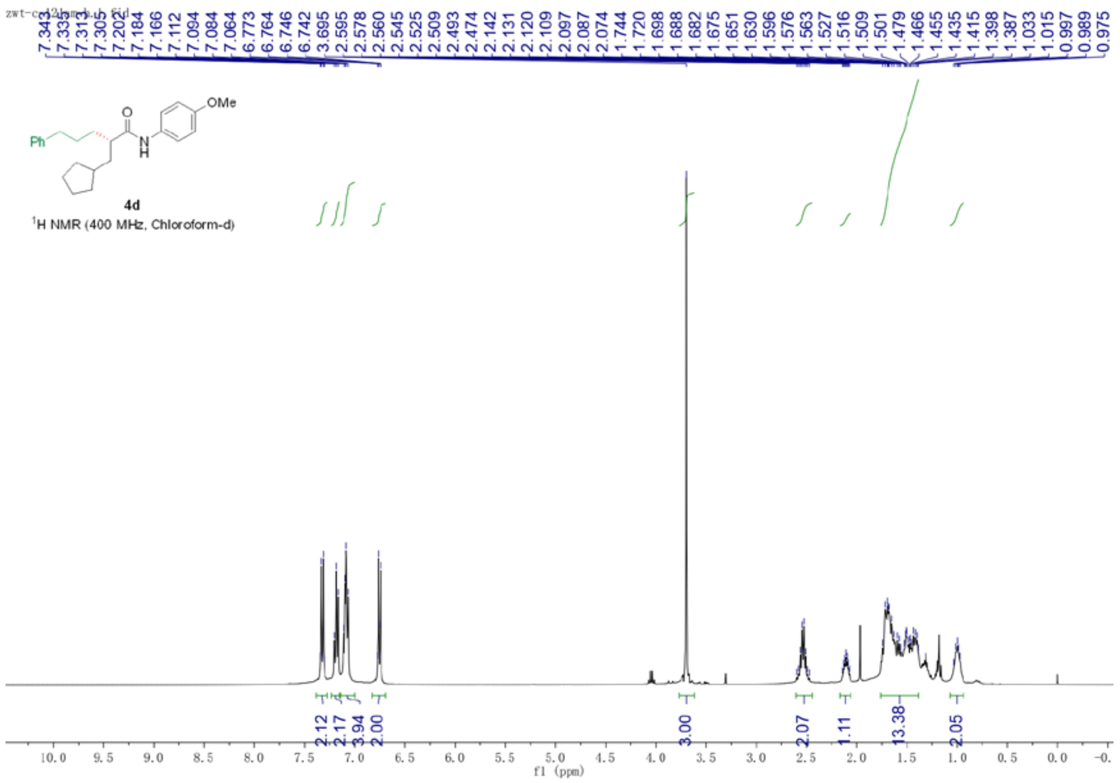

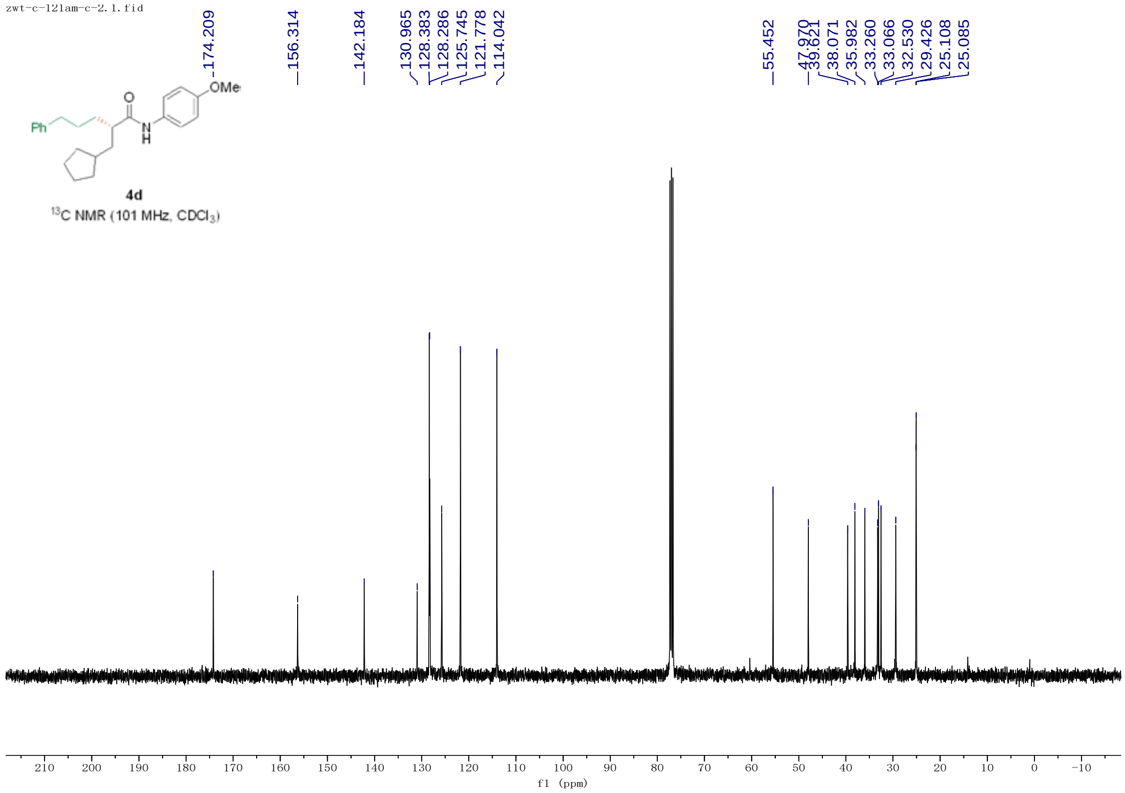

Supplementary Figure 137 ^{1}H-NMR (400 Mz, $CHCl_3$, 25 °C) and ^{13}C-NMR (101 MHz, $CHCl_3$, 25 °C) spectra of **4d**

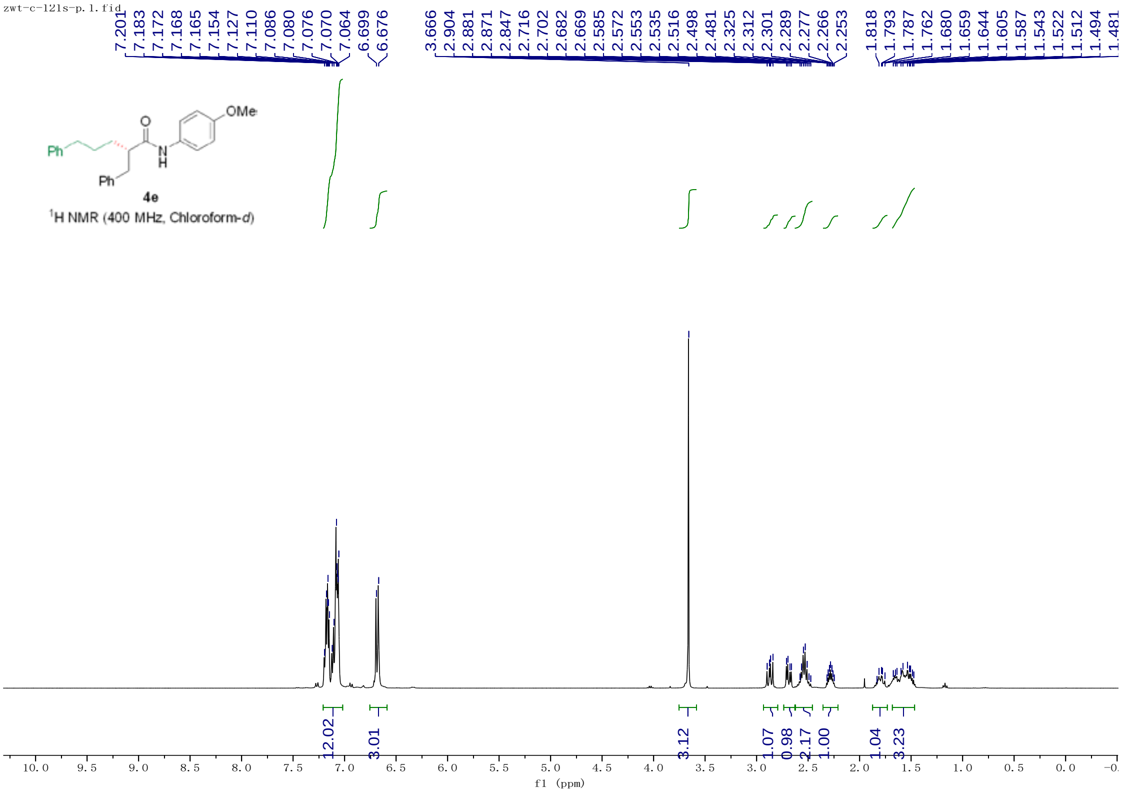

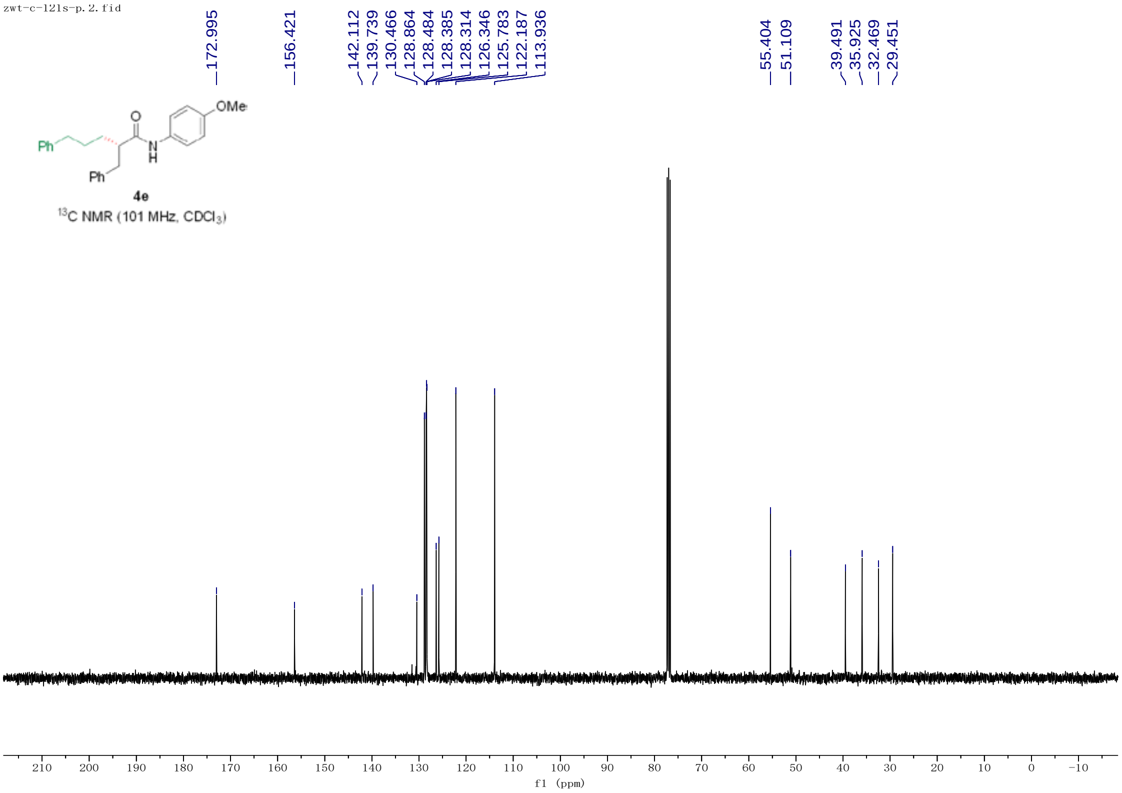

Supplementary Figure 138 ^{1}H-NMR (400 Mz, $CHCl_3$, 25 °C) and ^{13}C-NMR (101 MHz, $CHCl_3$, 25 °C) spectra of **4e**

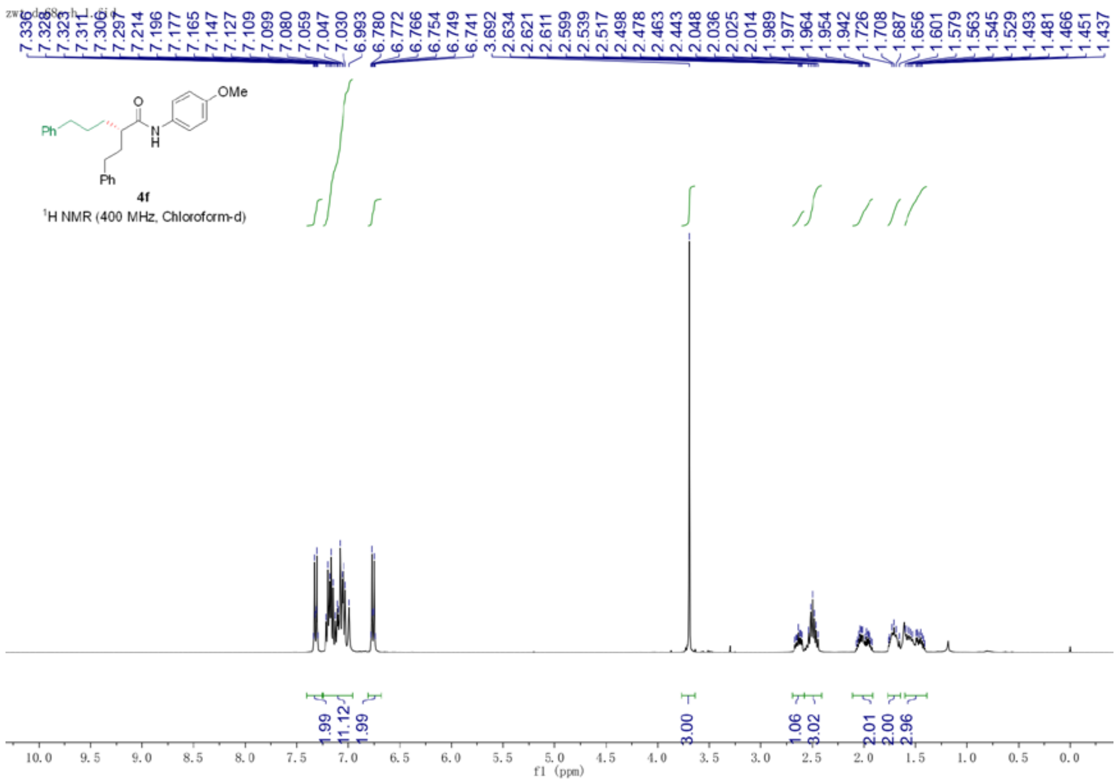

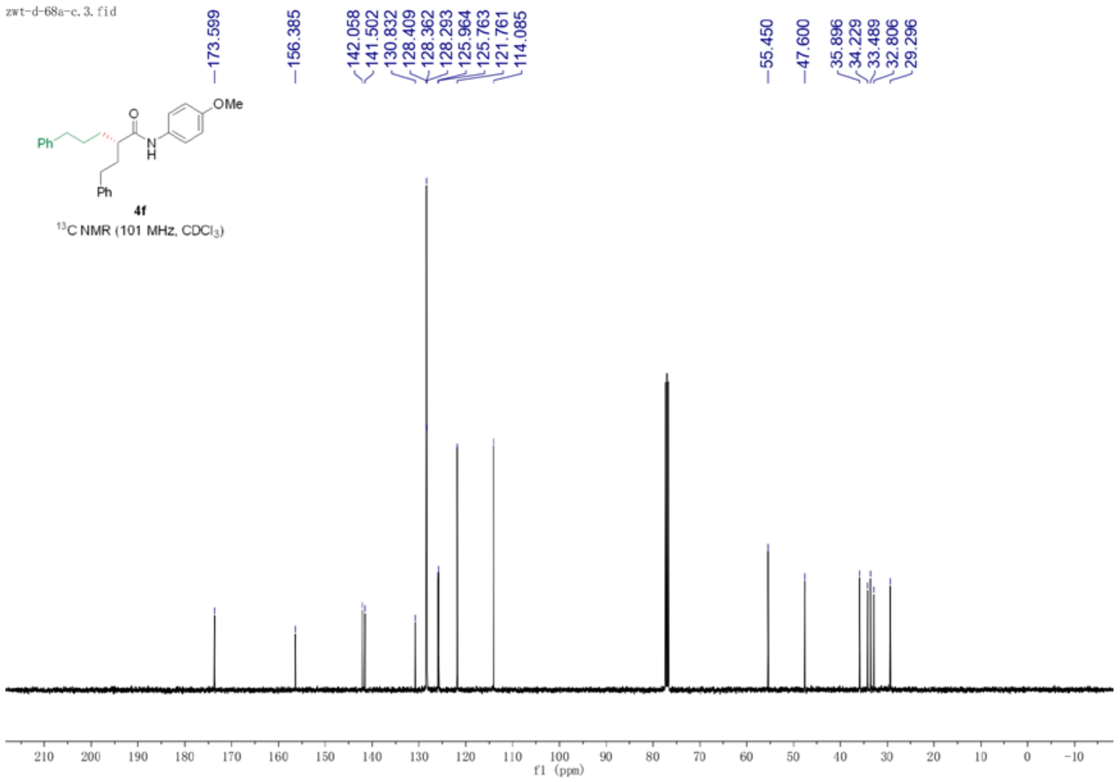

Supplementary Figure 139 ^{1}H-NMR (400 Mz, $CHCl_3$, 25 °C) and ^{13}C-NMR (101 MHz, $CHCl_3$, 25 °C) spectra of **4f**

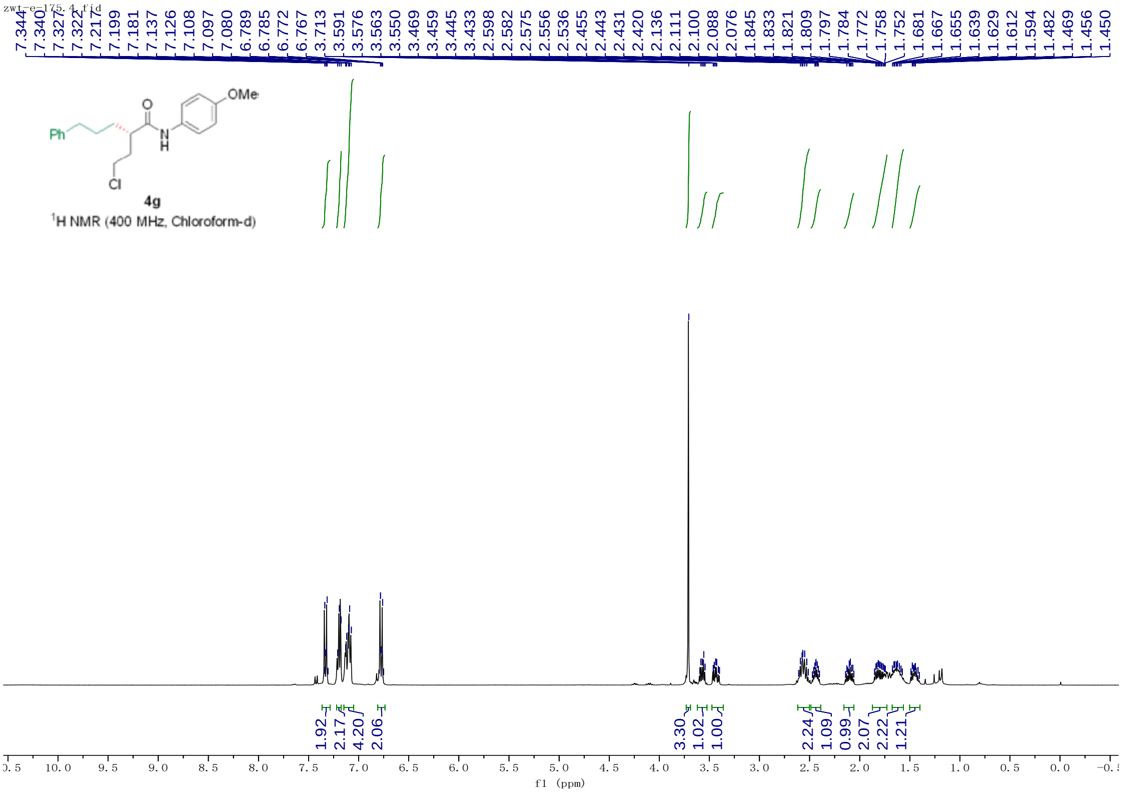

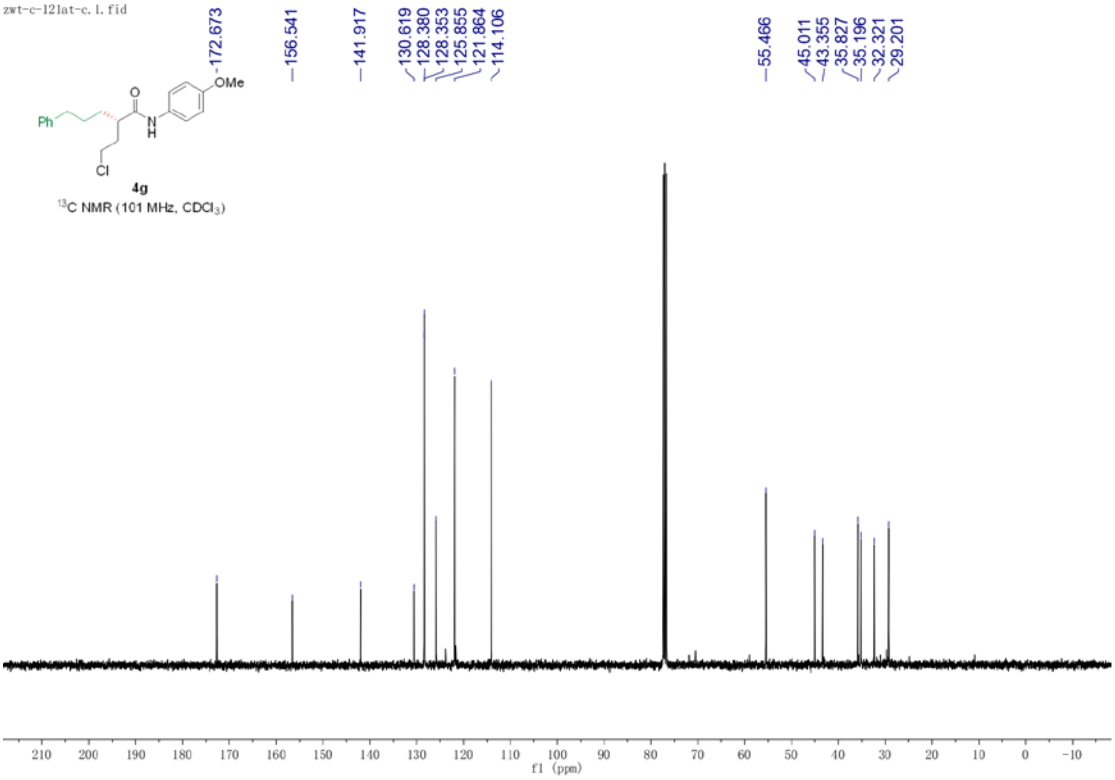

Supplementary Figure 140 ^{1}H-NMR (400 Mz, $CHCl_3$, 25 °C) and ^{13}C-NMR (101 MHz, $CHCl_3$, 25 °C) spectra of **4g**

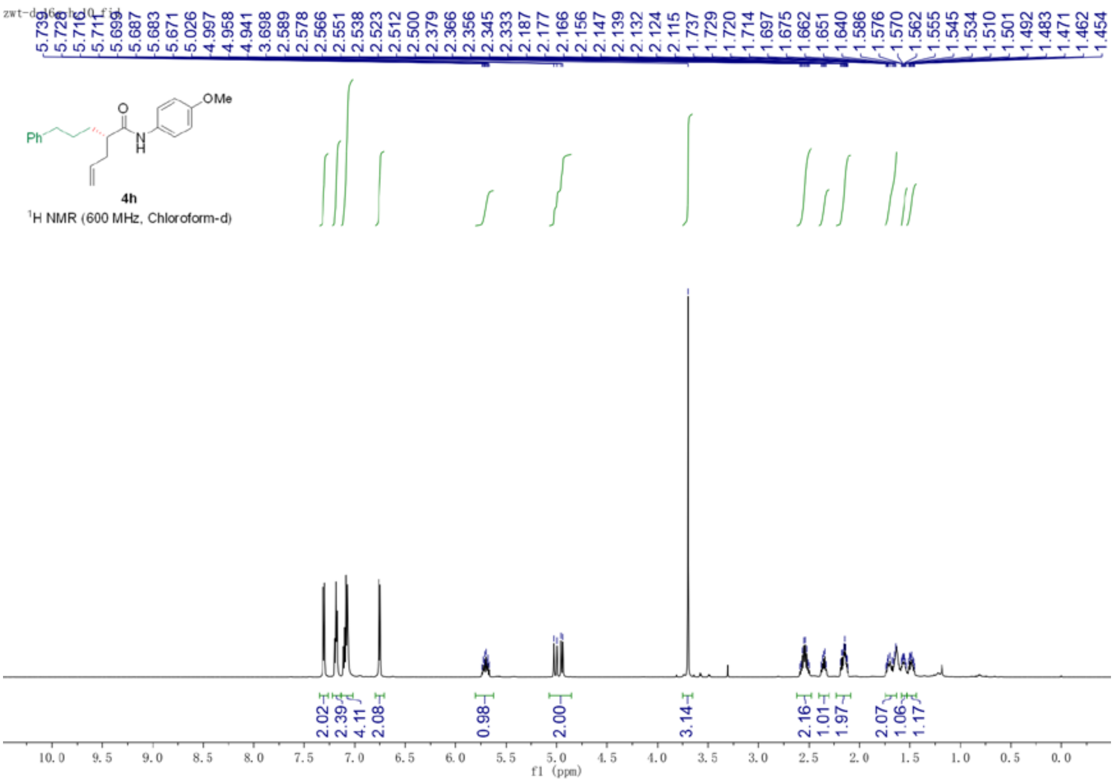

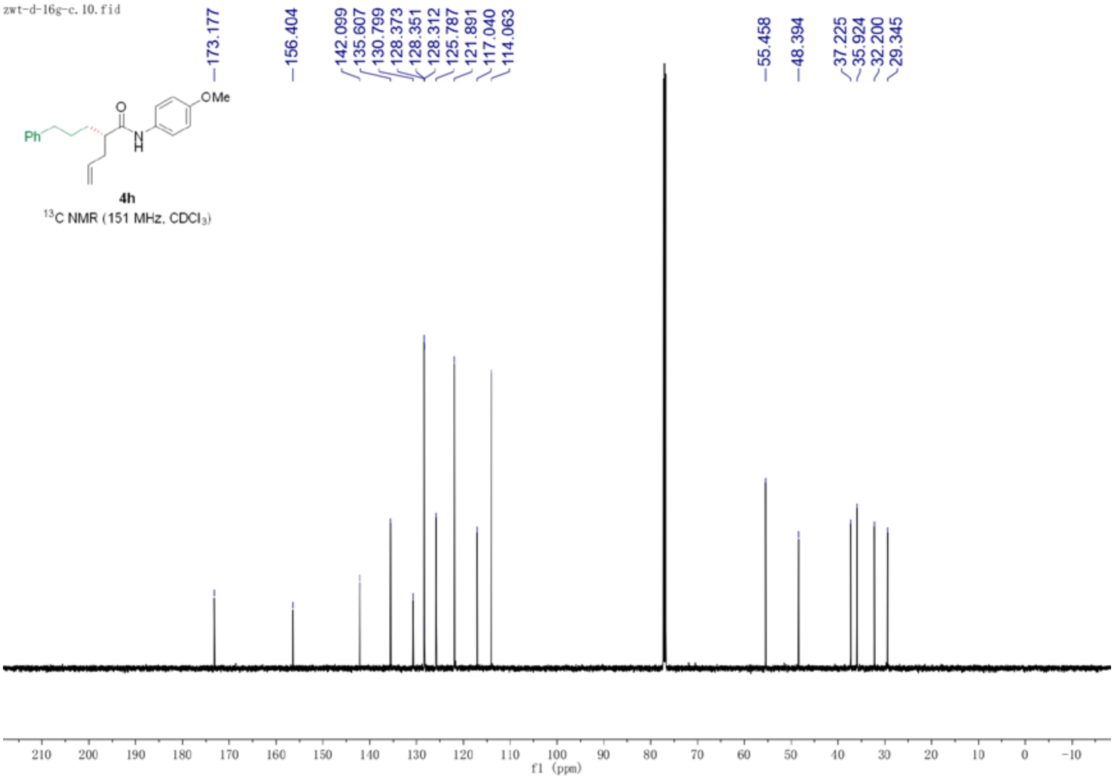

Supplementary Figure 141 ^{1}H-NMR (600 Mz, $CHCl_3$, 25 °C) and ^{13}C-NMR (151 MHz, $CHCl_3$, 25 °C) spectra of **4h**

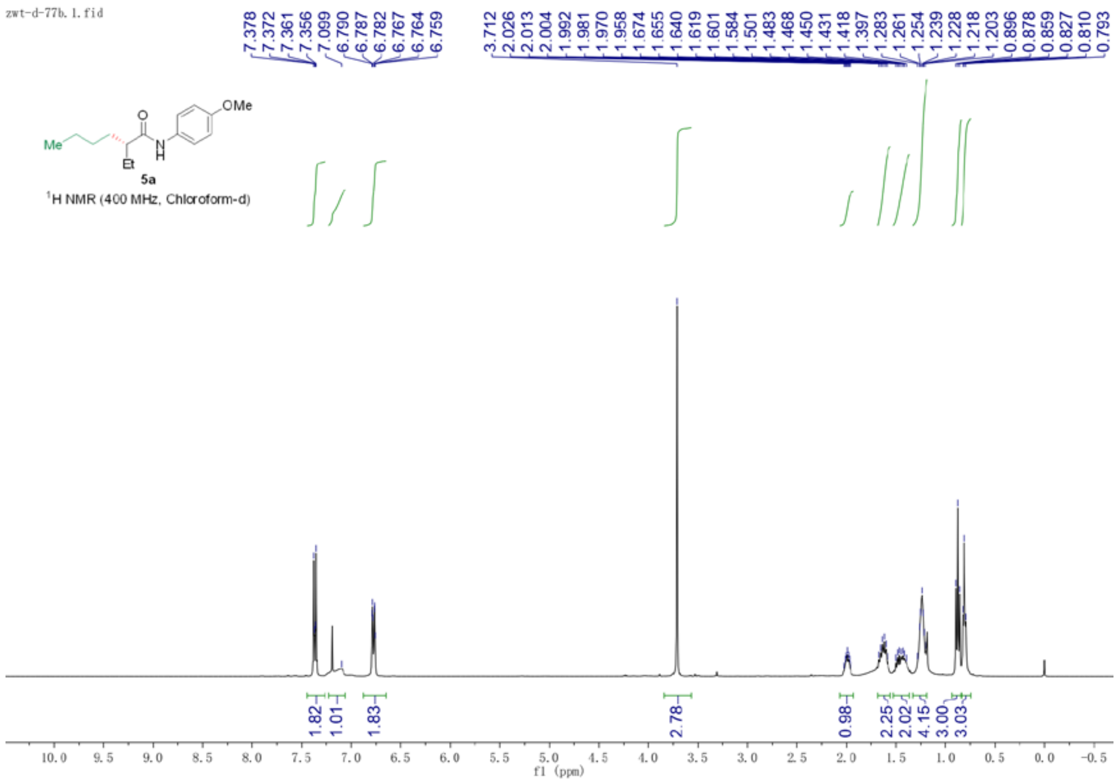

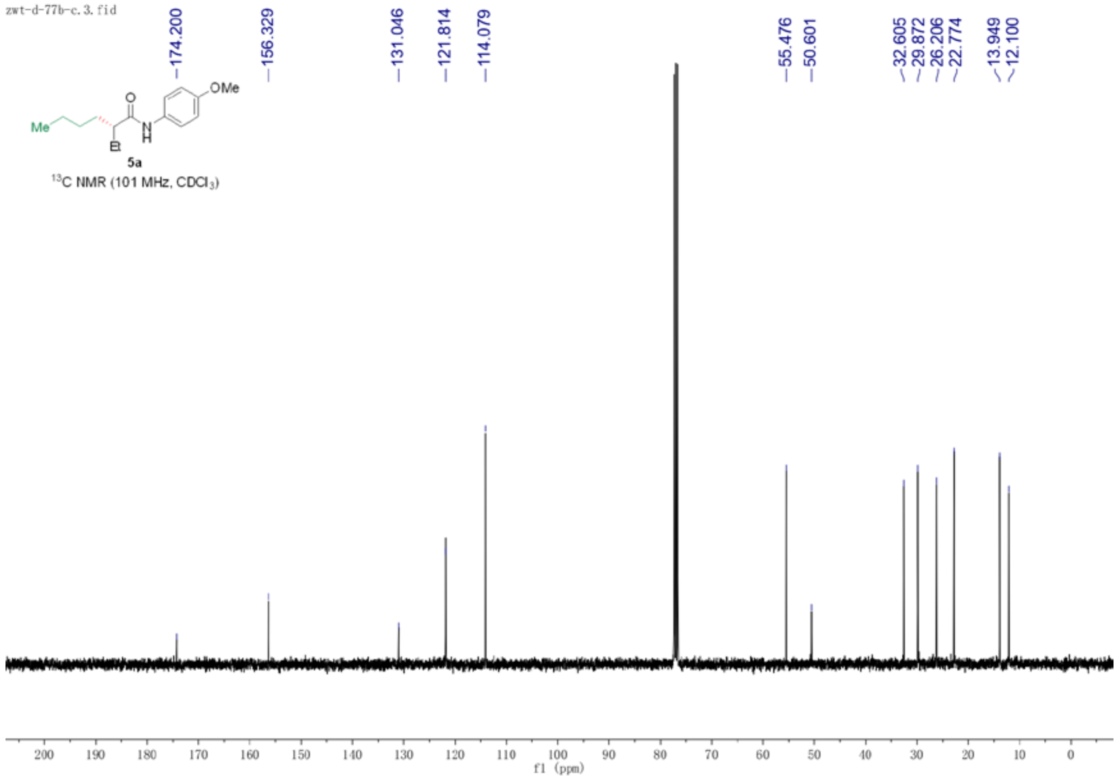

Supplementary Figure 142 ^{1}H-NMR (400 Mz, $CHCl_3$, 25 °C) and ^{13}C-NMR (101 MHz, $CHCl_3$, 25 °C) spectra of **5a**

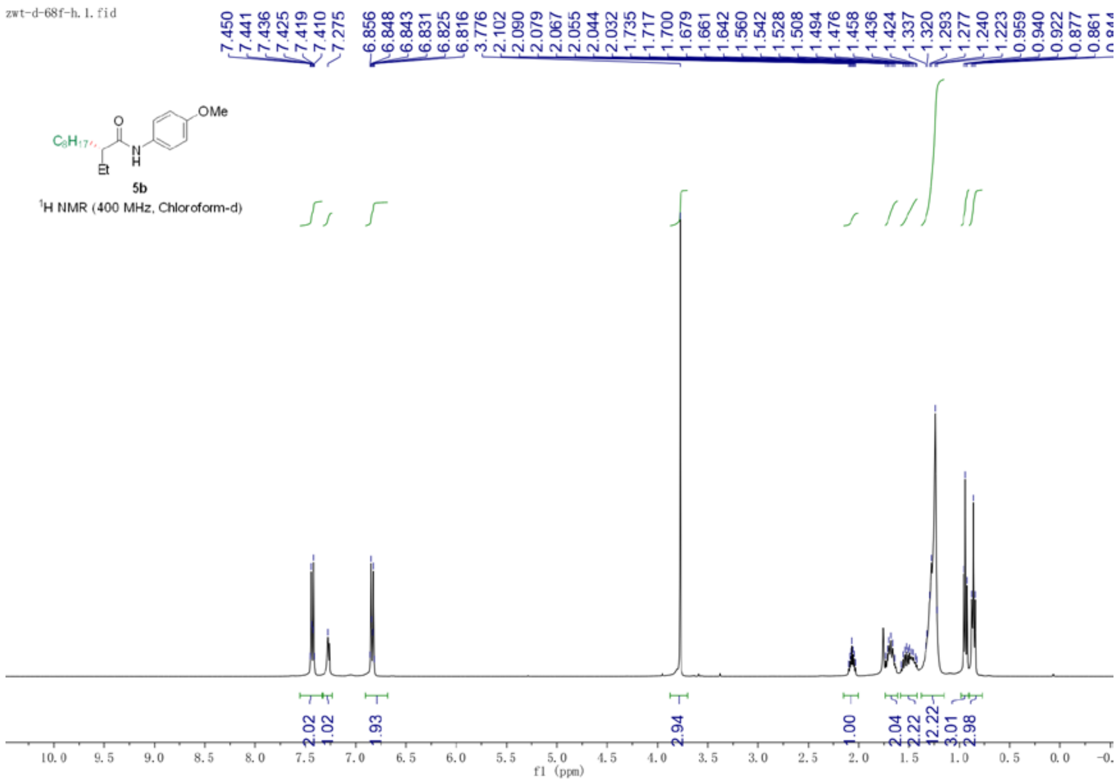

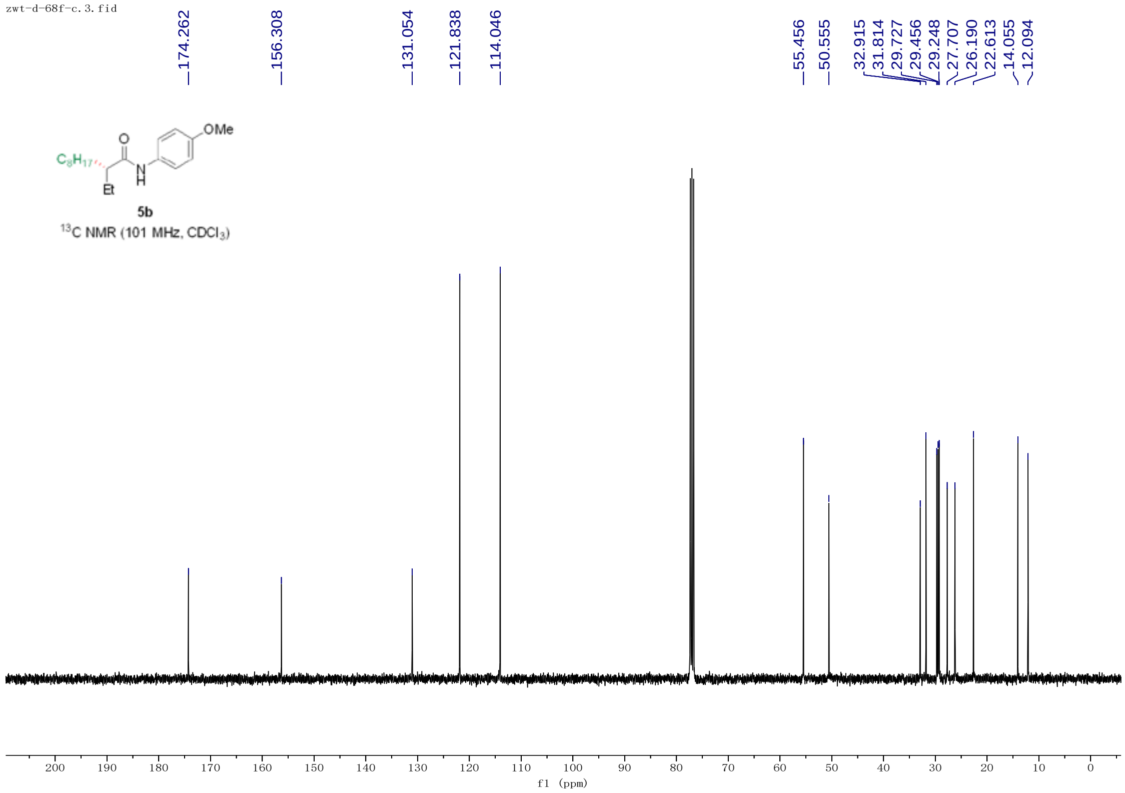

Supplementary Figure 143 ^{1}H-NMR (400 Mz, $CHCl_3$, 25 °C) and ^{13}C-NMR (101 MHz, $CHCl_3$, 25 °C) spectra of **5b**

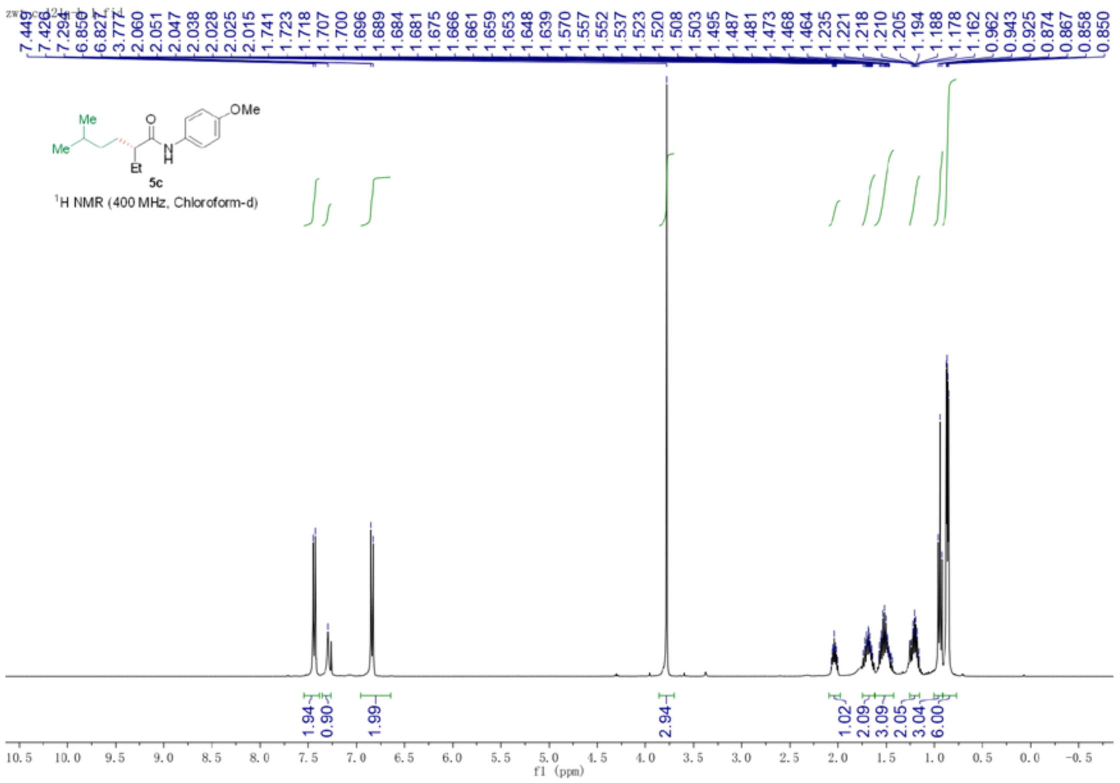

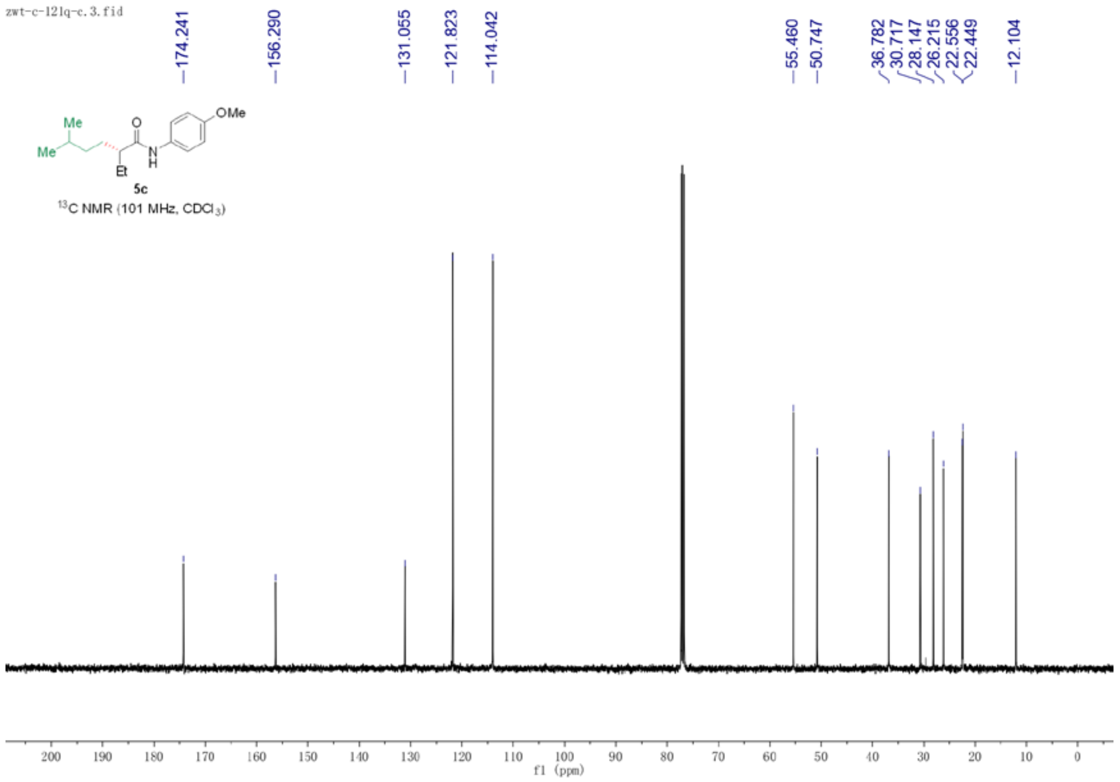

Supplementary Figure 144 ^{1}H-NMR (400 Mz, $CHCl_3$, 25 °C) and ^{13}C-NMR (101 MHz, $CHCl_3$, 25 °C) spectra of **5c**

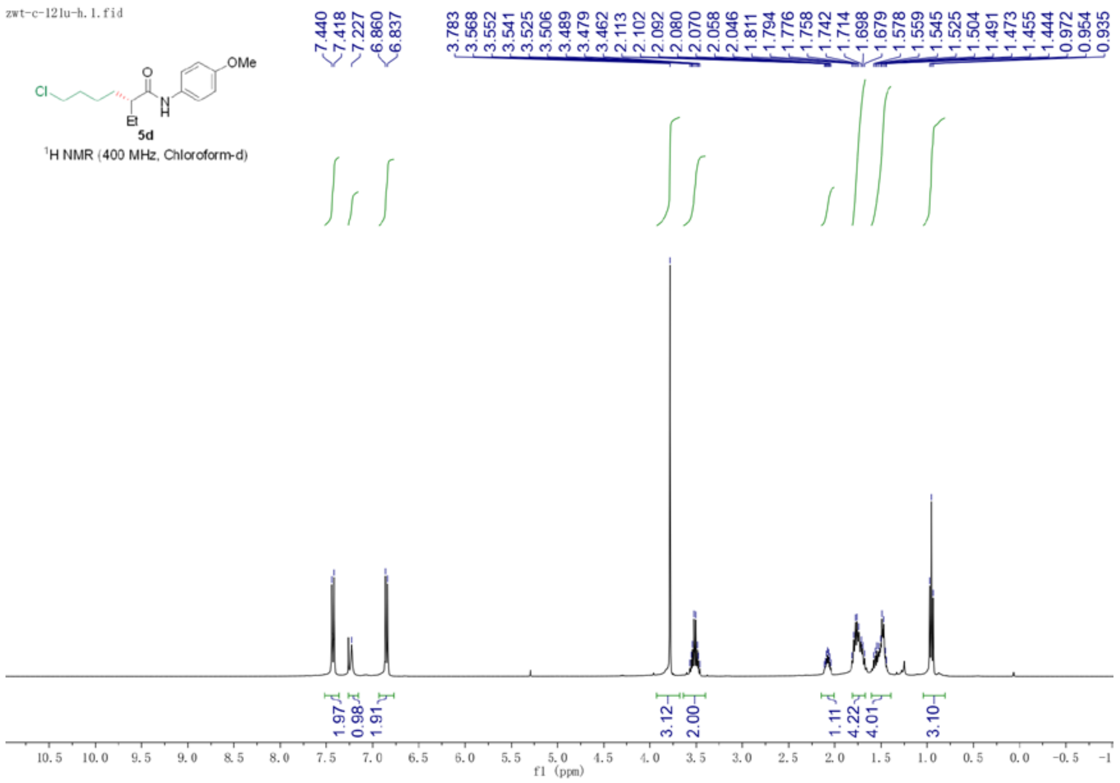

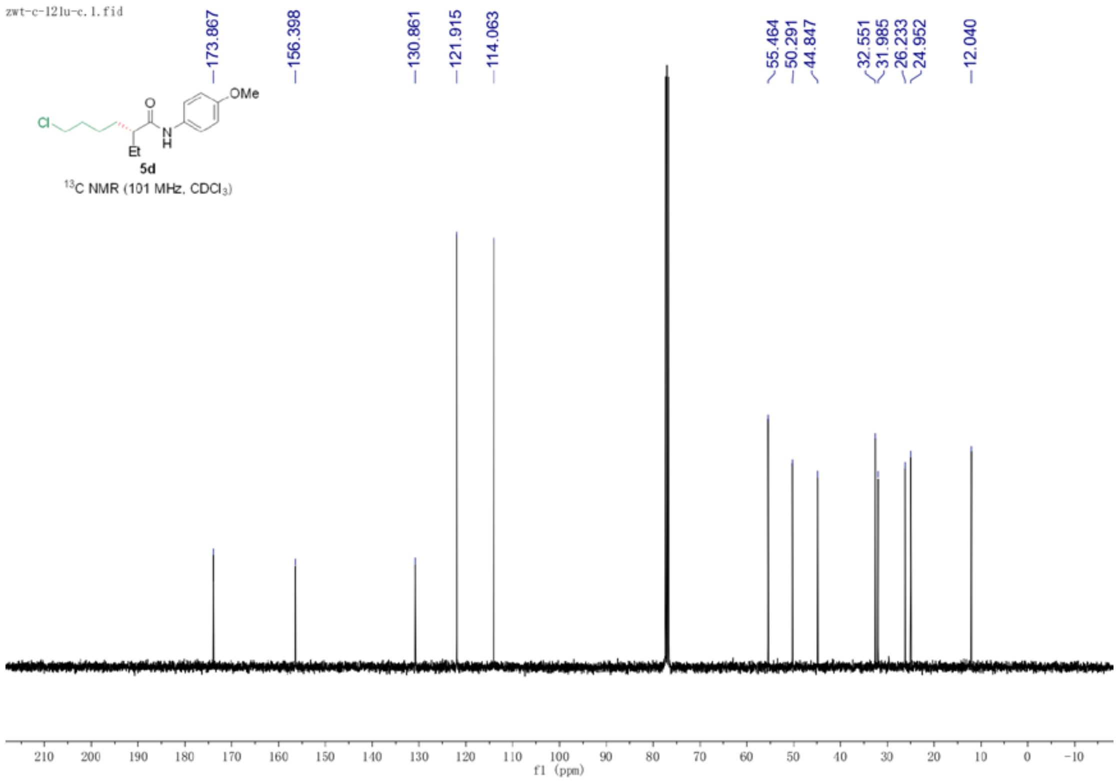

Supplementary Figure 145 ^{1}H-NMR (400 Mz, $CHCl_3$, 25 °C) and ^{13}C-NMR (101 MHz, $CHCl_3$, 25 °C) spectra of **5d**

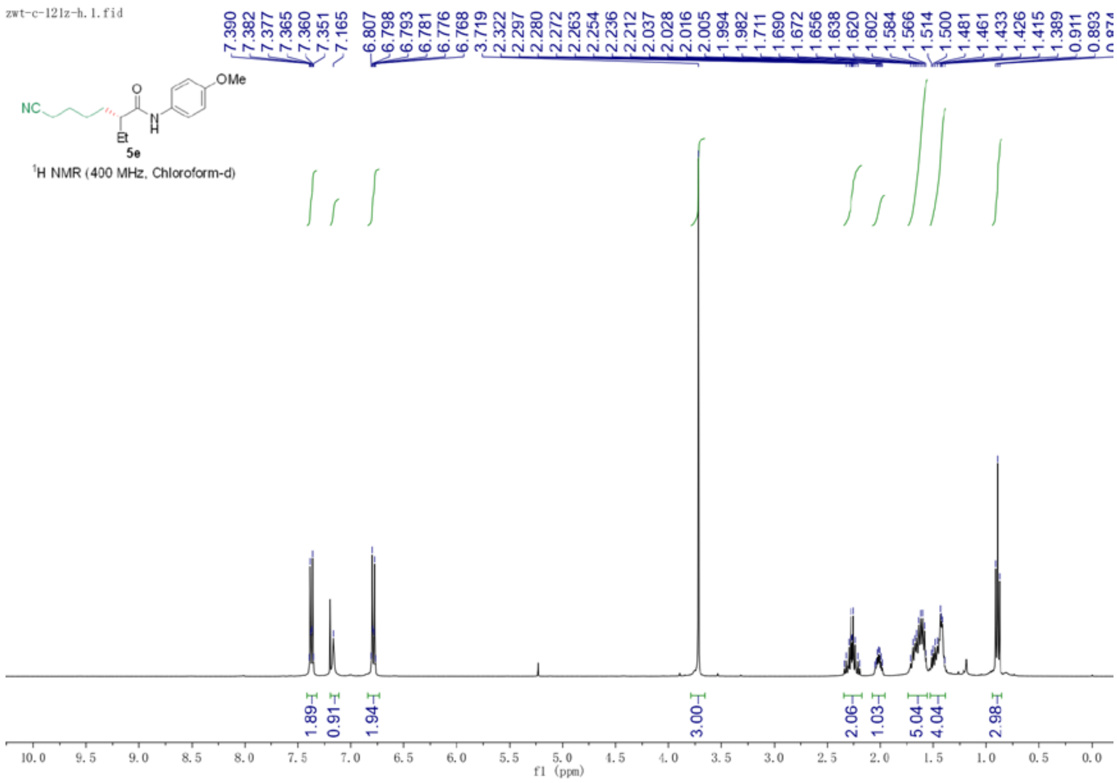

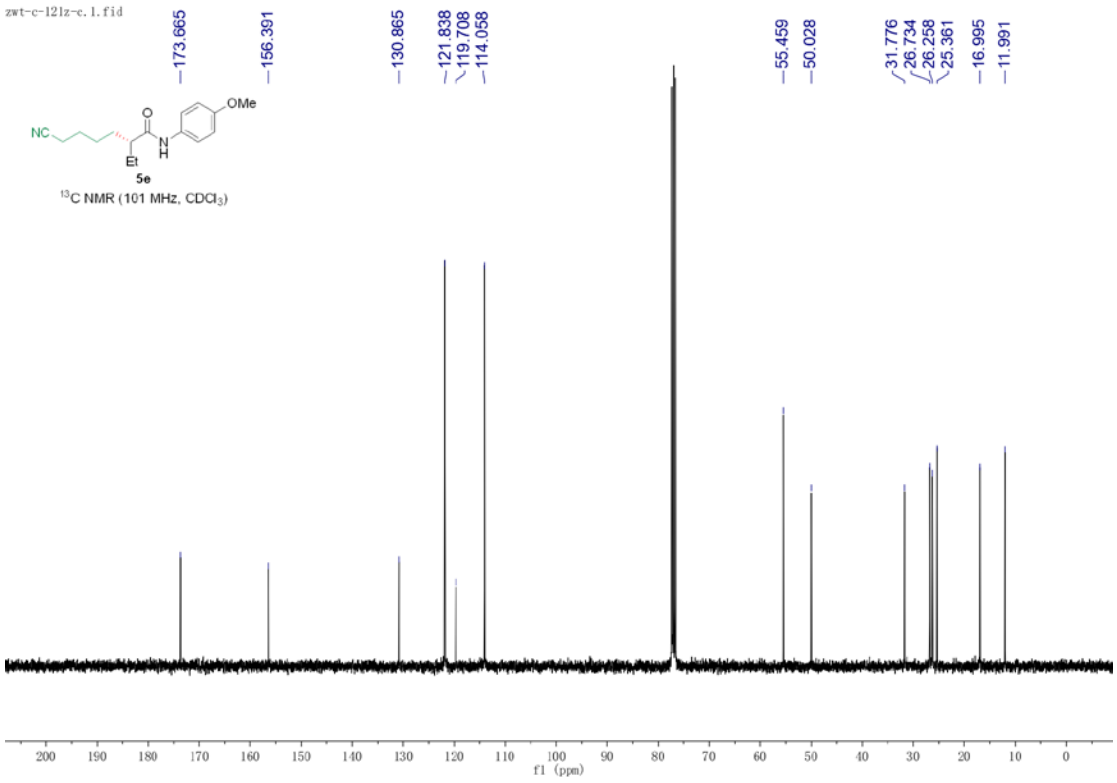

Supplementary Figure 146 ^{1}H-NMR (400 Mz, $CHCl_3$, 25 °C) and ^{13}C-NMR (101 MHz, $CHCl_3$, 25 °C) spectra of **5e**

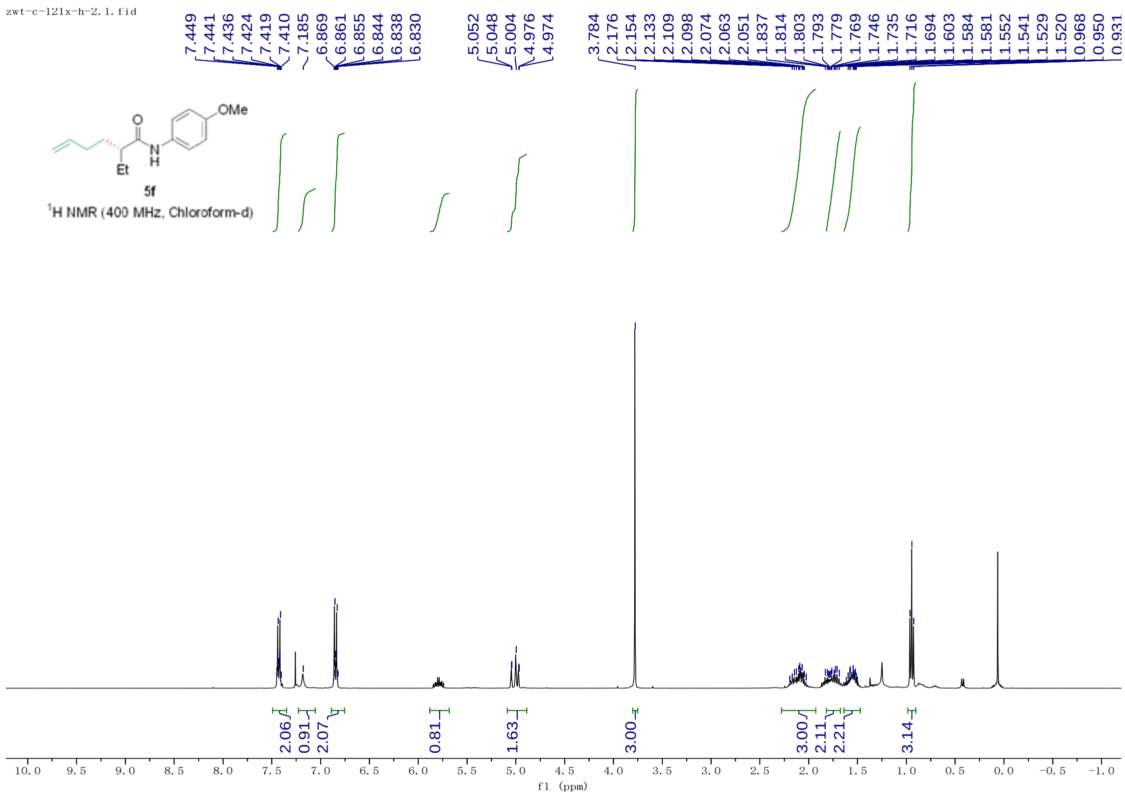

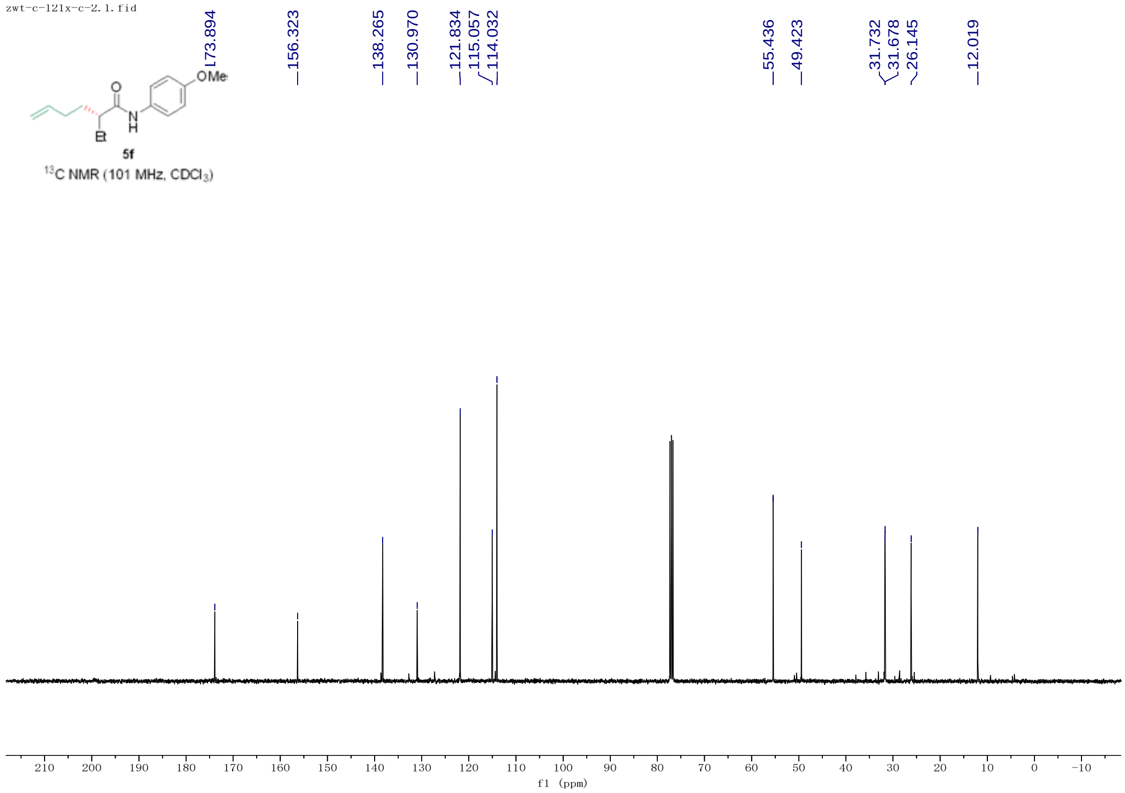

Supplementary Figure 147 ^{1}H-NMR (400 Mz, $CHCl_3$, 25 °C) and ^{13}C-NMR (101 MHz, $CHCl_3$, 25 °C) spectra of **5f**

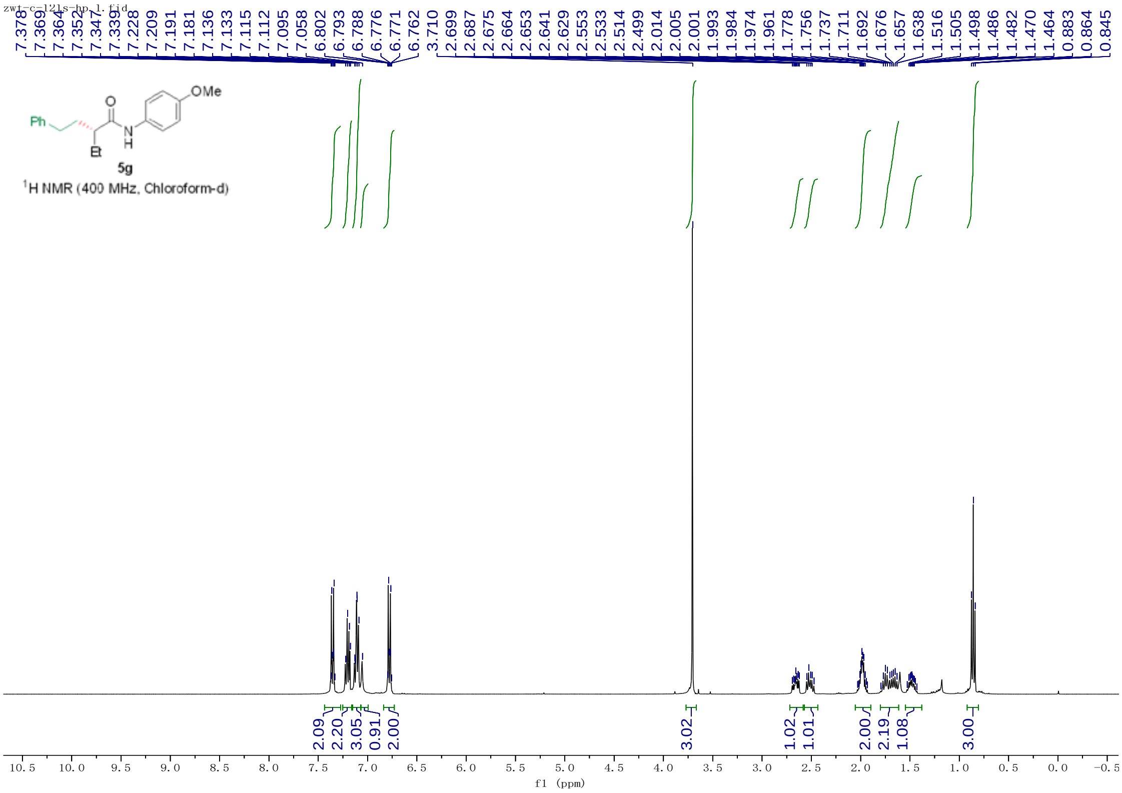

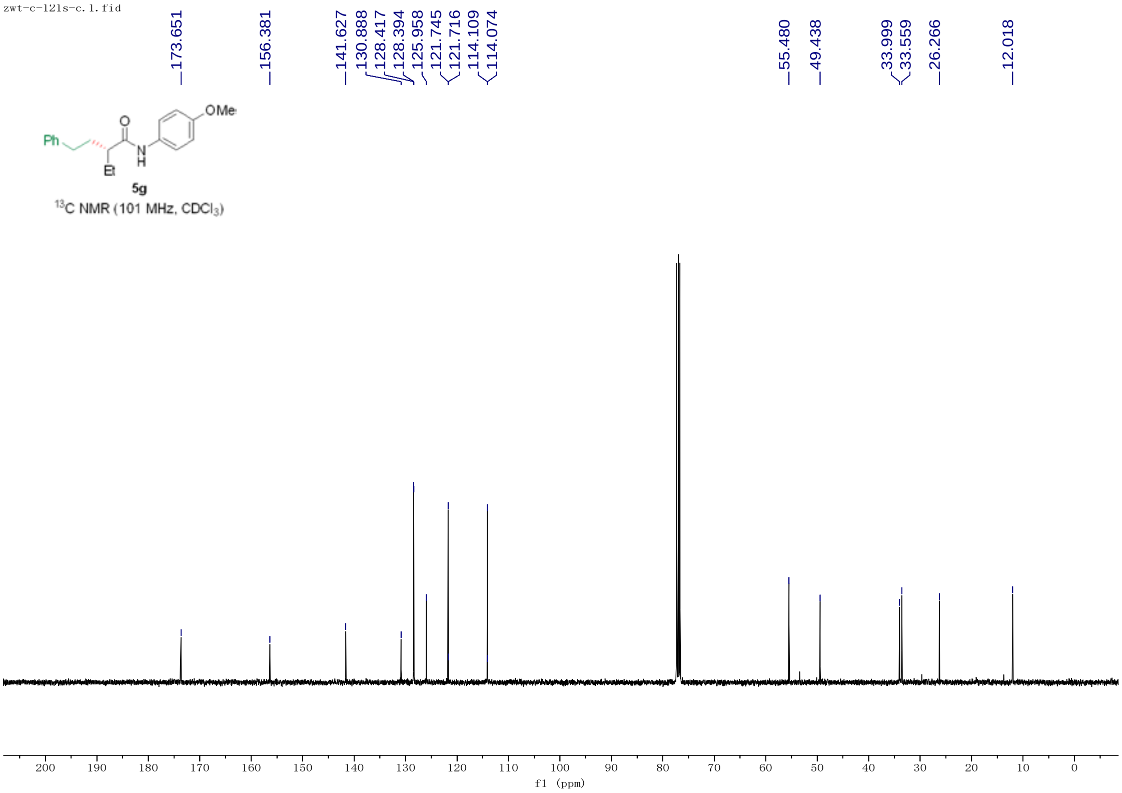

Supplementary Figure 148 ^{1}H-NMR (400 Mz, $CHCl_3$, 25 °C) and ^{13}C-NMR (101 MHz, $CHCl_3$, 25 °C) spectra of **5g**

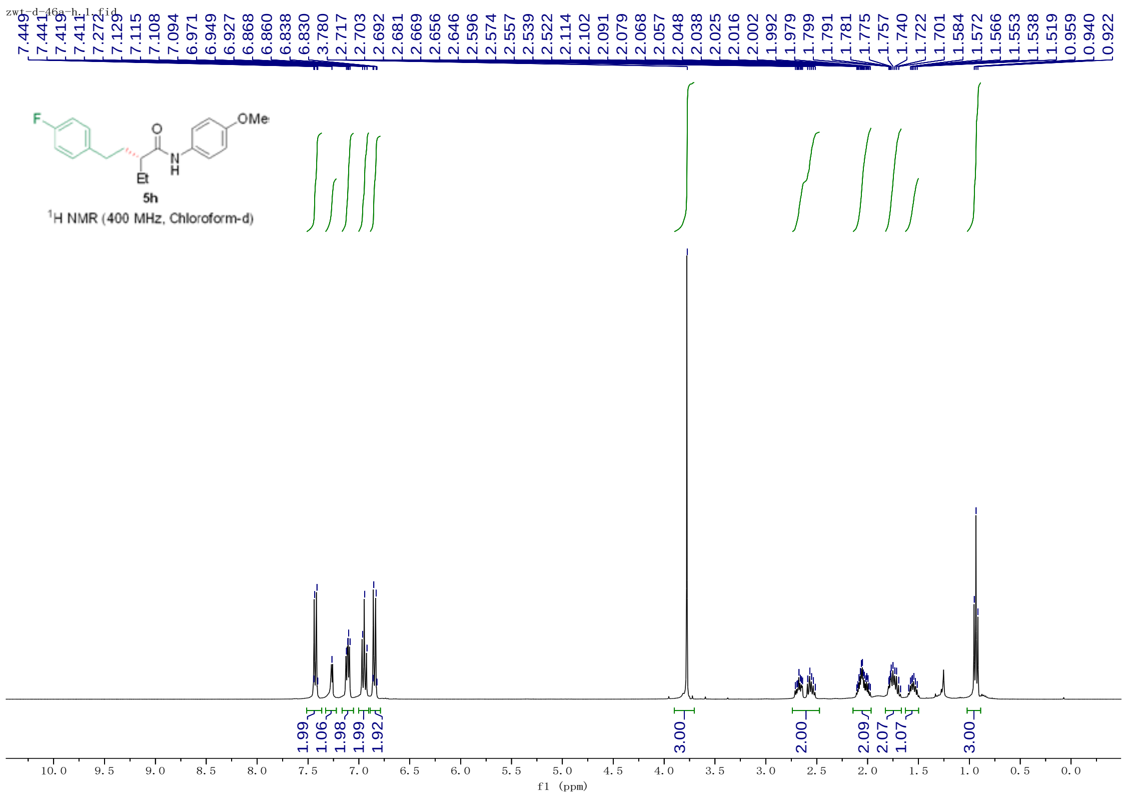

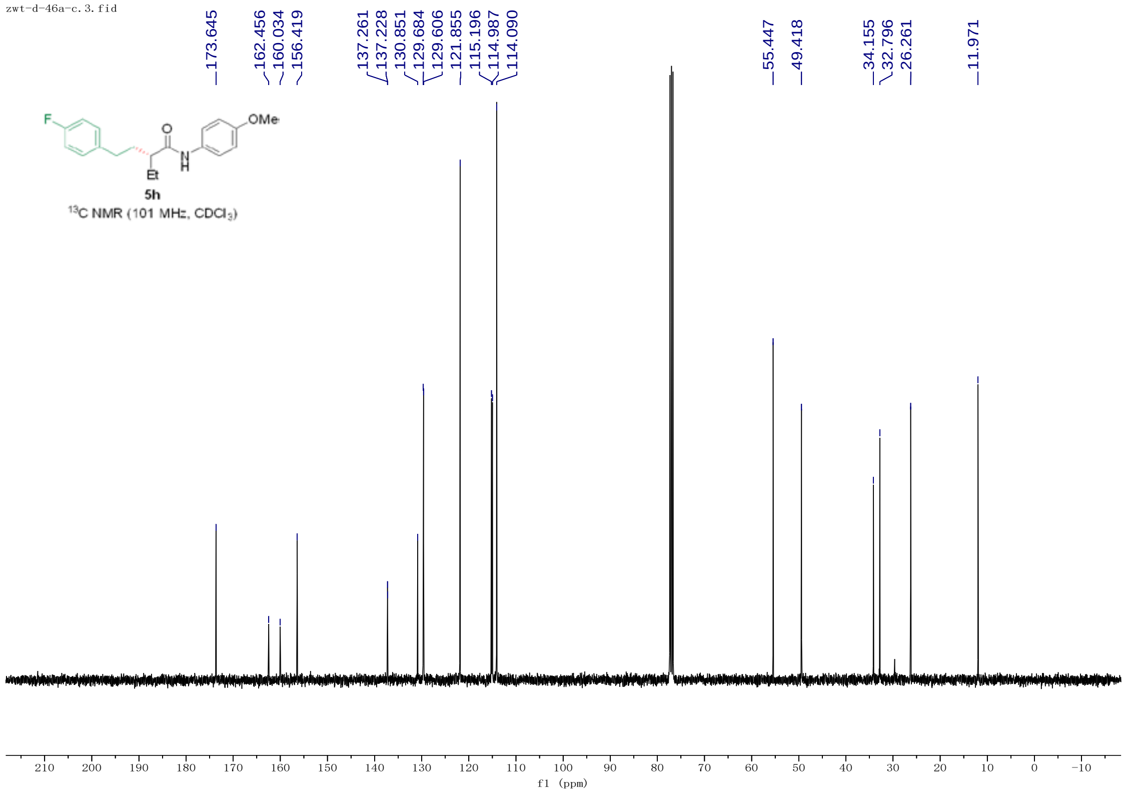

Supplementary Figure 149 ^{1}H-NMR (400 Mz, $CHCl_3$, 25 °C) and ^{13}C-NMR (101 MHz, $CHCl_3$, 25 °C) spectra of **5h**

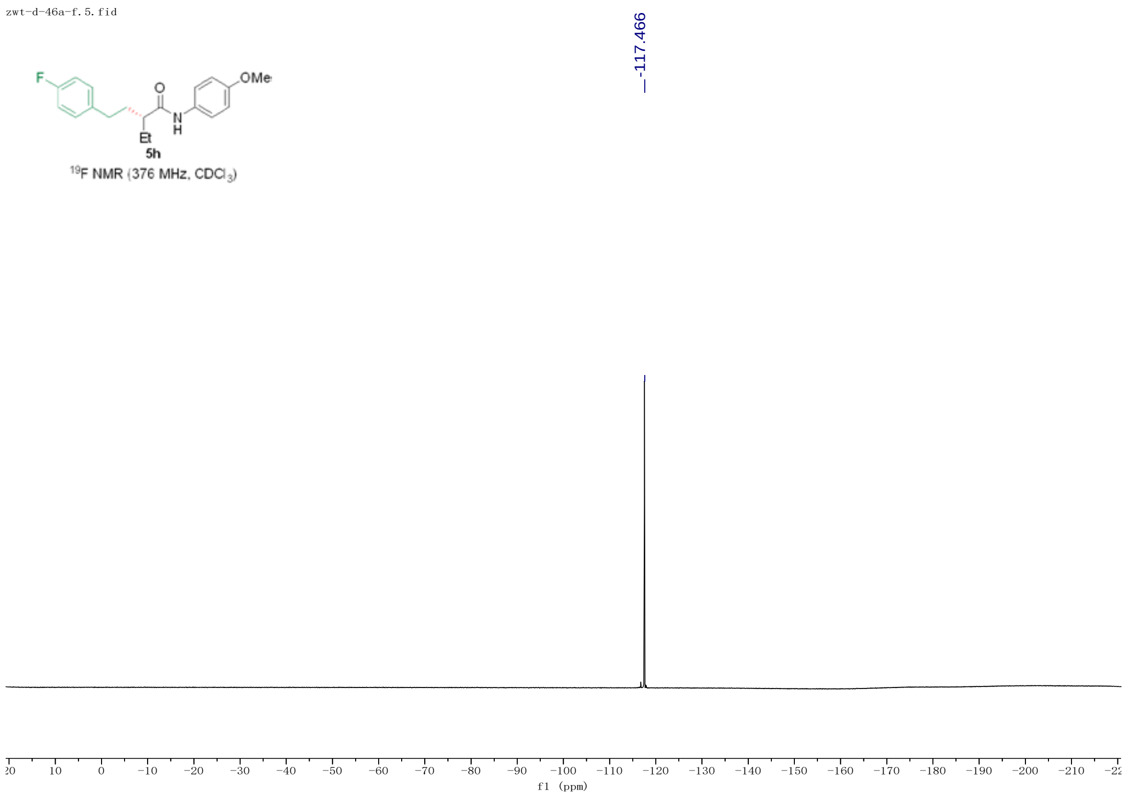

Supplementary Figure 150 ^{19}F-NMR (376 Mz, $CHCl_3$, 25 ℃) spectra of **5h**

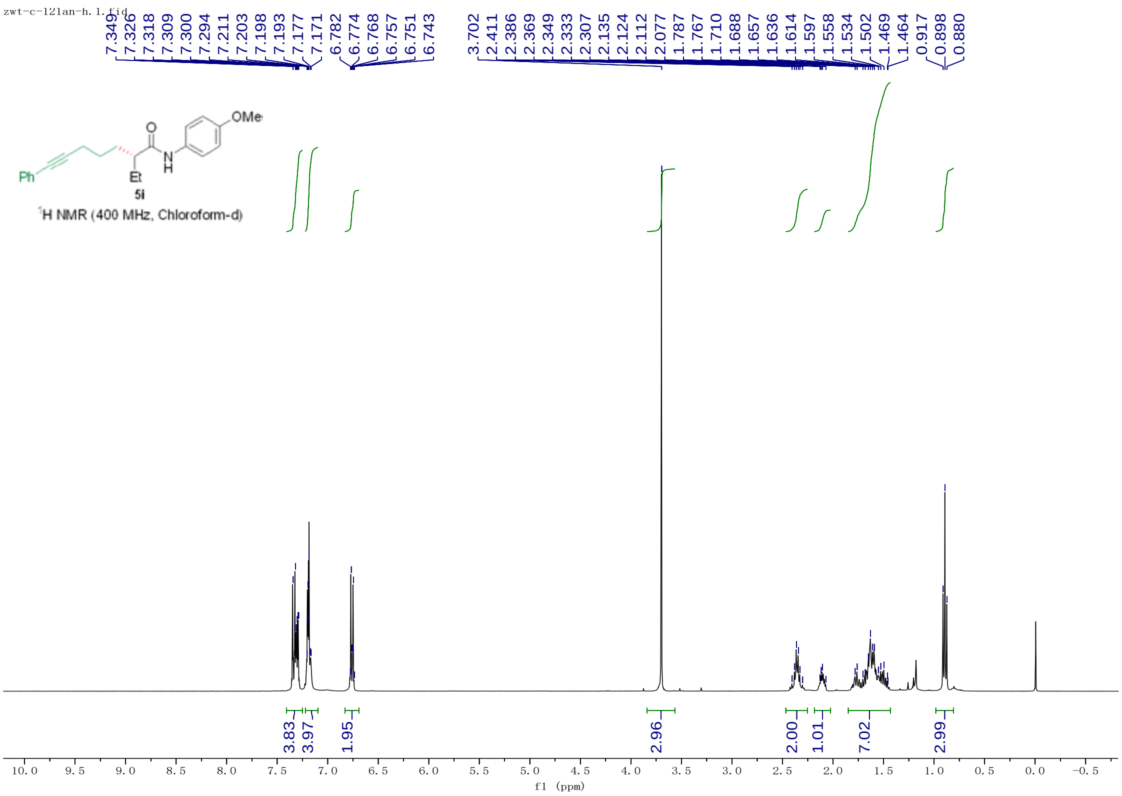

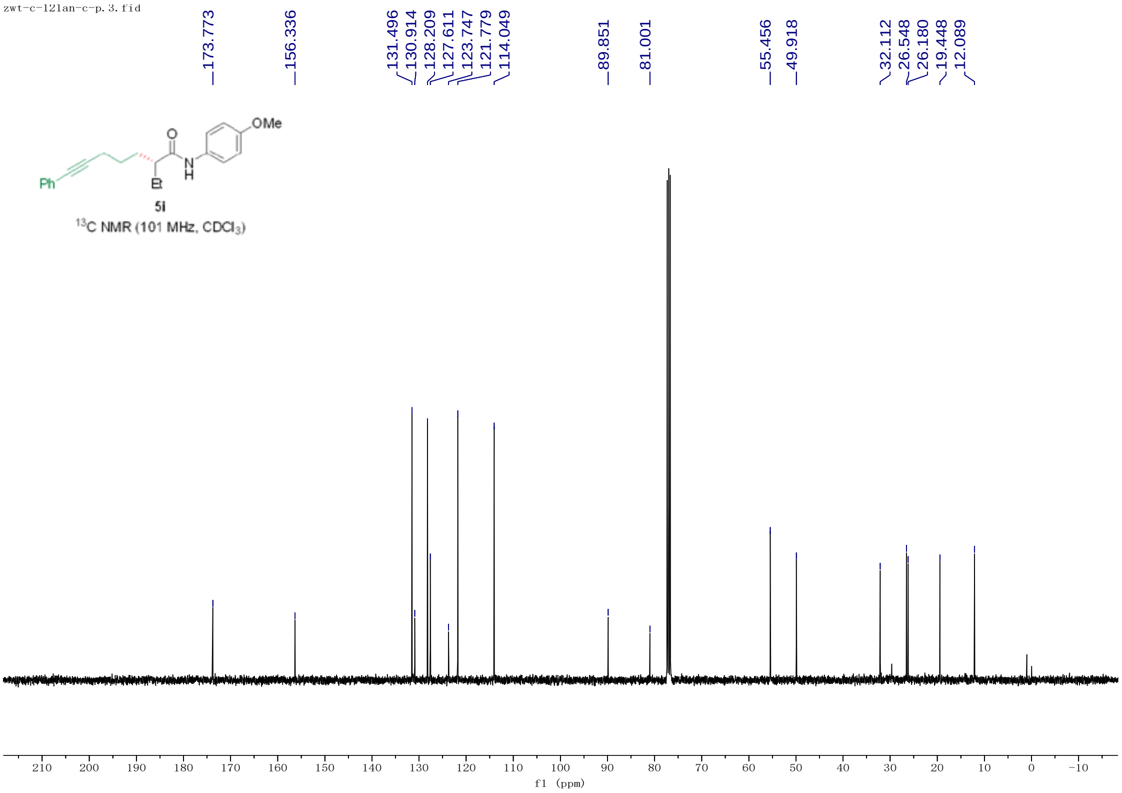

Supplementary Figure 151 ^{1}H-NMR (400 Mz, $CHCl_3$, 25 °C) and ^{13}C-NMR (101 MHz, $CHCl_3$, 25 °C) spectra of **5i**

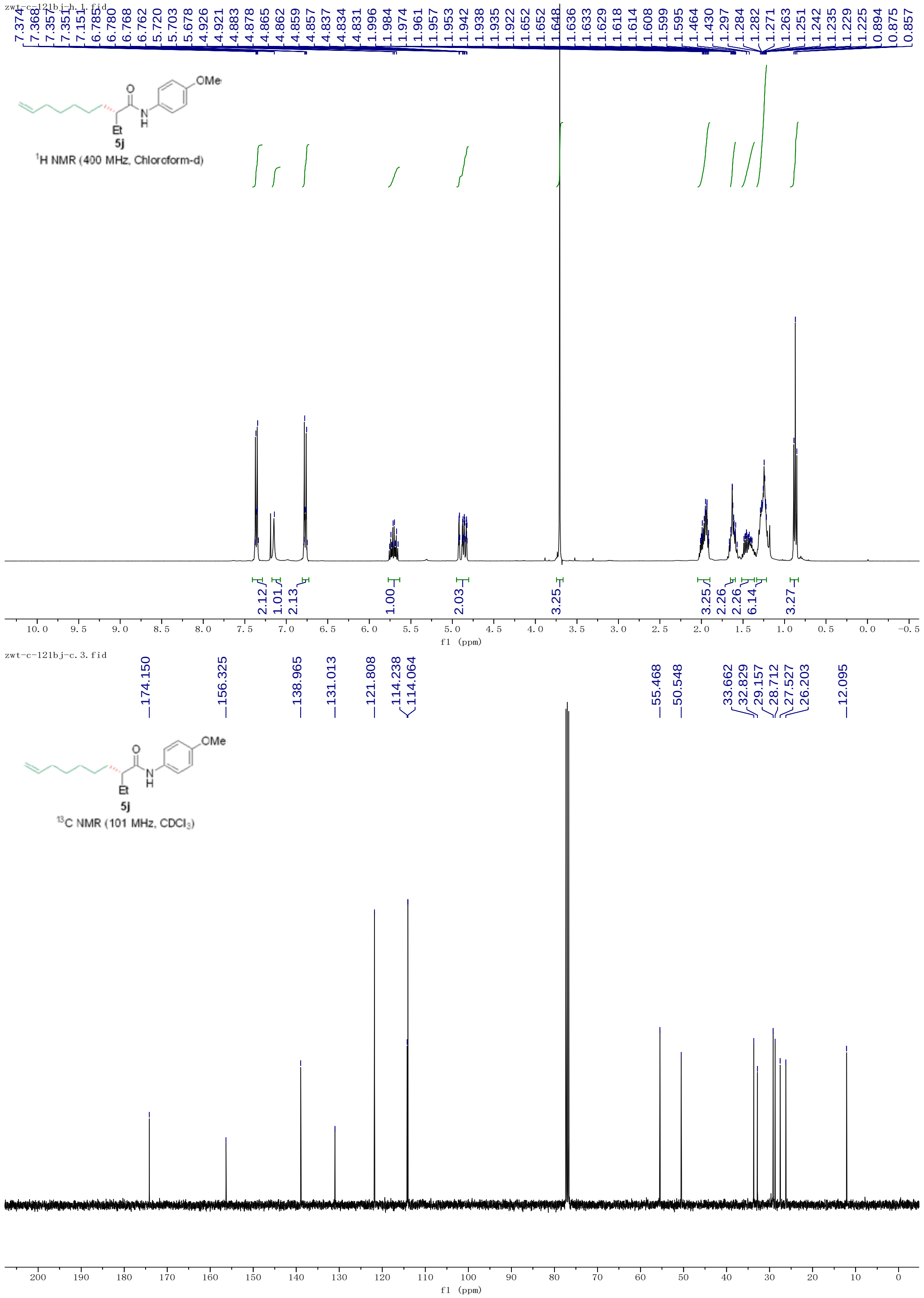

Supplementary Figure 152 ^{1}H-NMR (400 Mz, $CHCl_3$, 25 °C) and ^{13}C-NMR (101 MHz, $CHCl_3$, 25 °C) spectra of **5j**

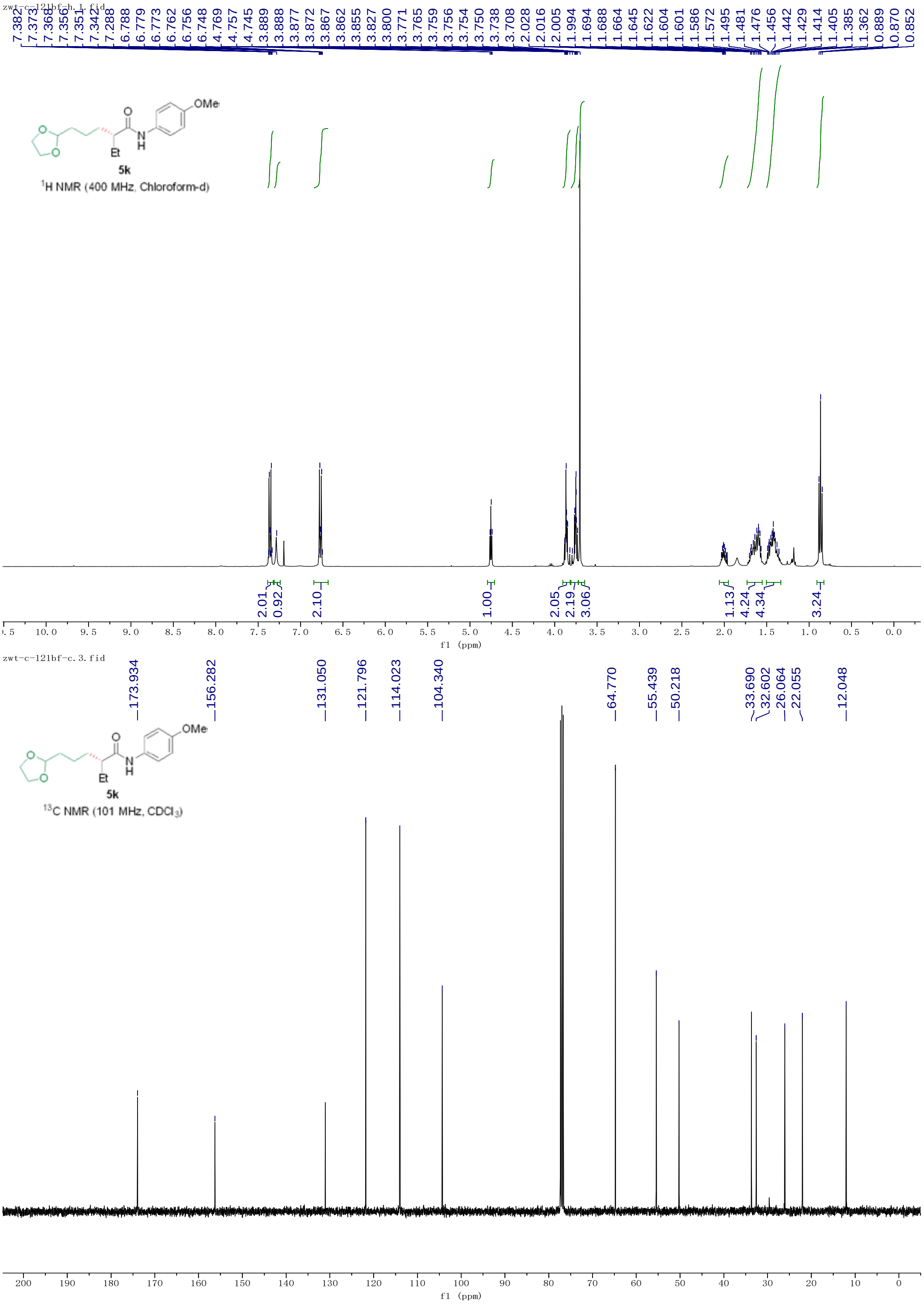

Supplementary Figure 153 ^{1}H-NMR (400 Mz, $CHCl_3$, 25 °C) and ^{13}C-NMR (101 MHz, $CHCl_3$, 25 °C) spectra of **5k**

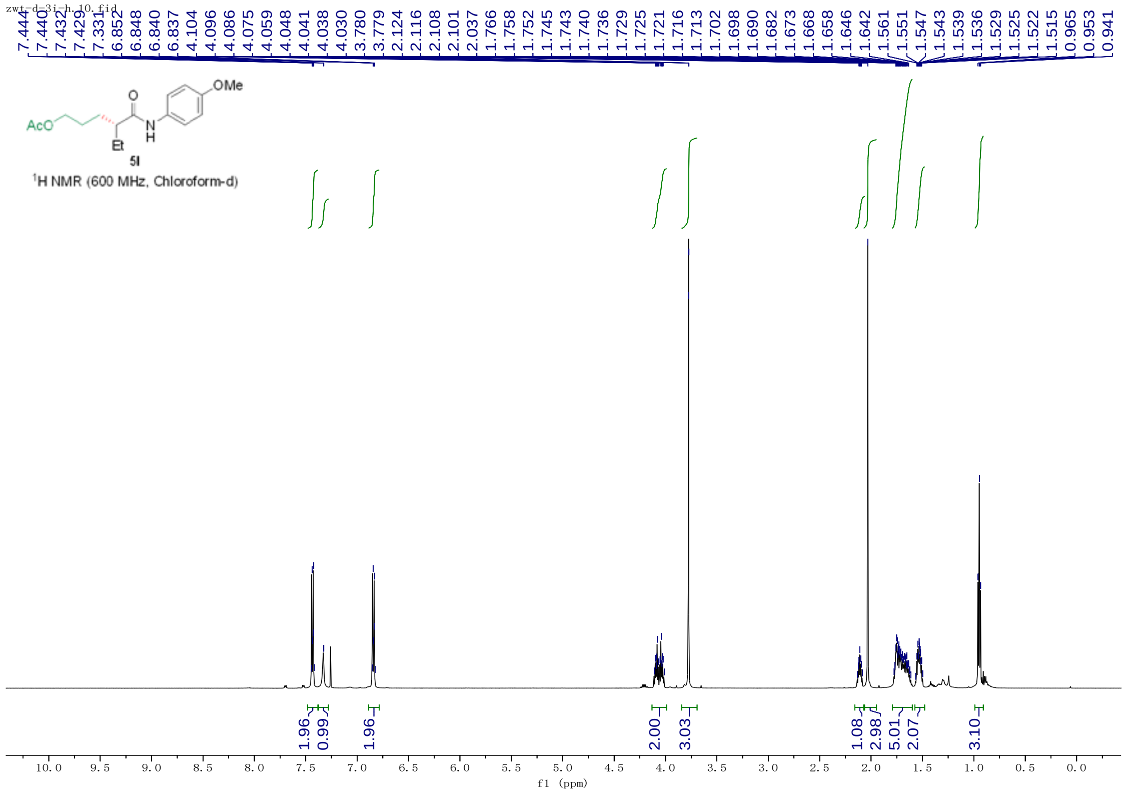

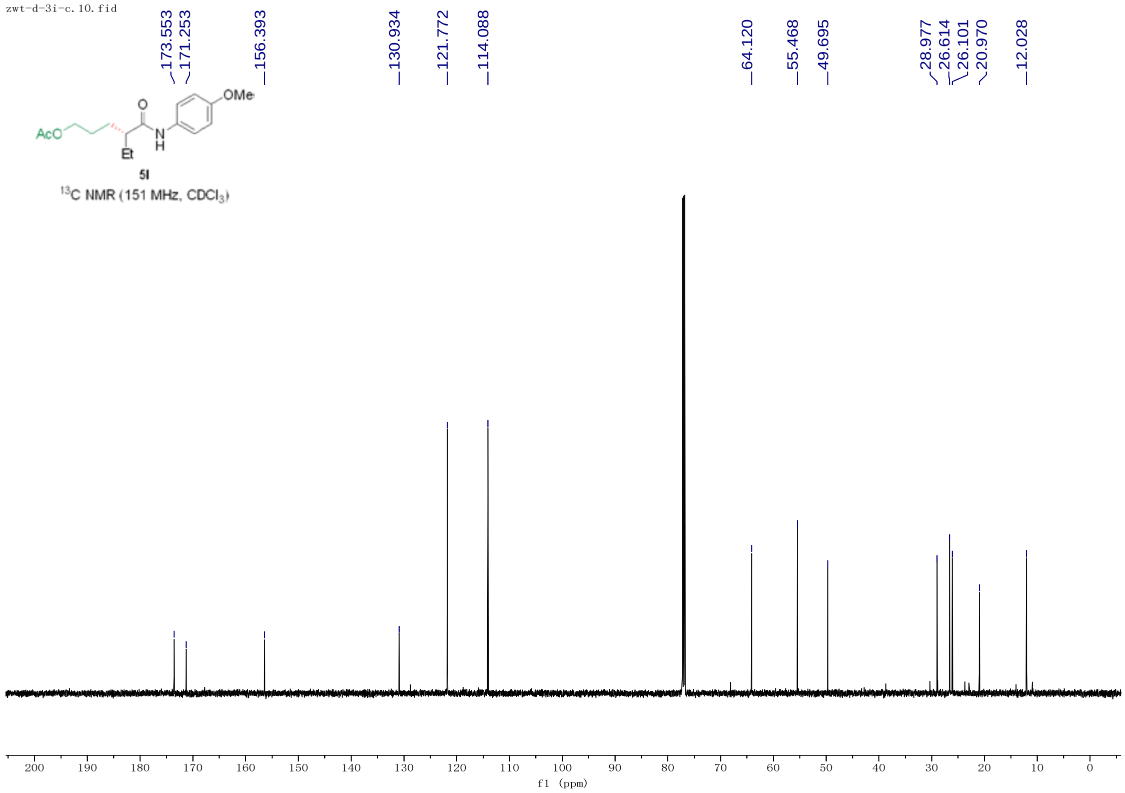

Supplementary Figure 154 ^{1}H-NMR (600 Mz, $CHCl_3$, 25 °C) and ^{13}C-NMR (151 MHz, $CHCl_3$, 25 °C) spectra of **5l**

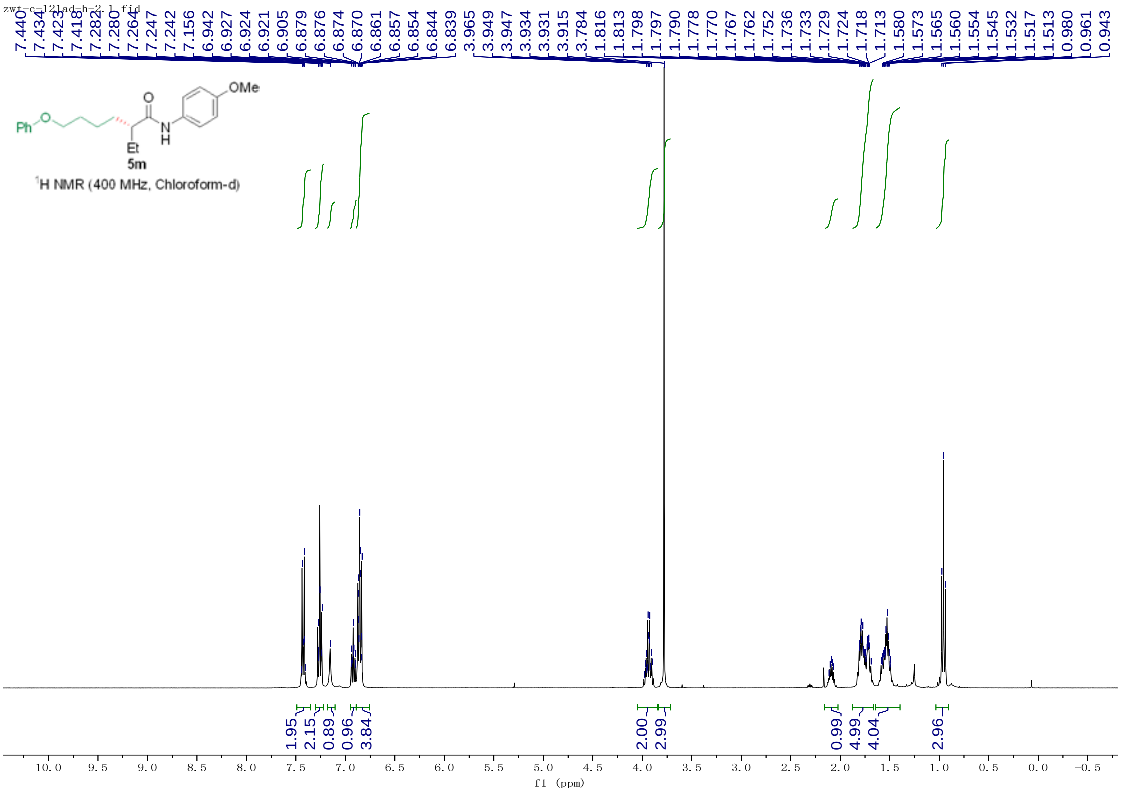

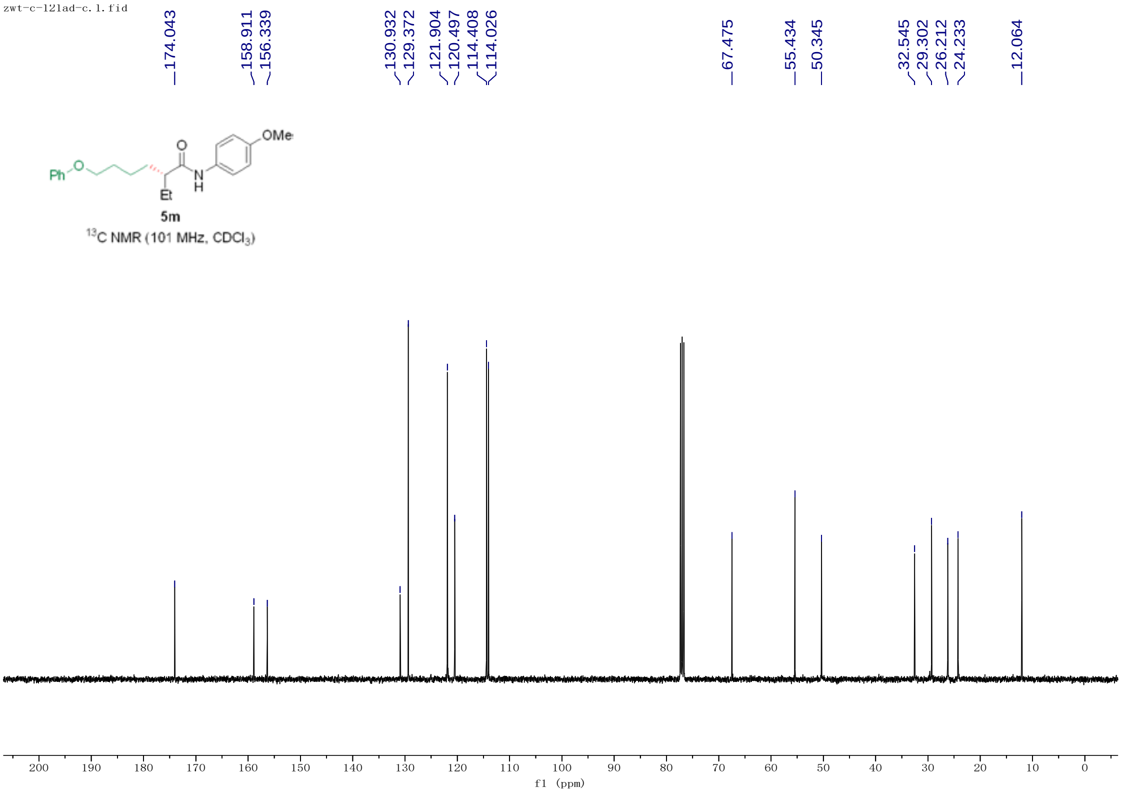

Supplementary Figure 155 ^{1}H-NMR (400 Mz, $CHCl_3$, 25 °C) and ^{13}C-NMR (101 MHz, $CHCl_3$, 25 °C) spectra of **5m**

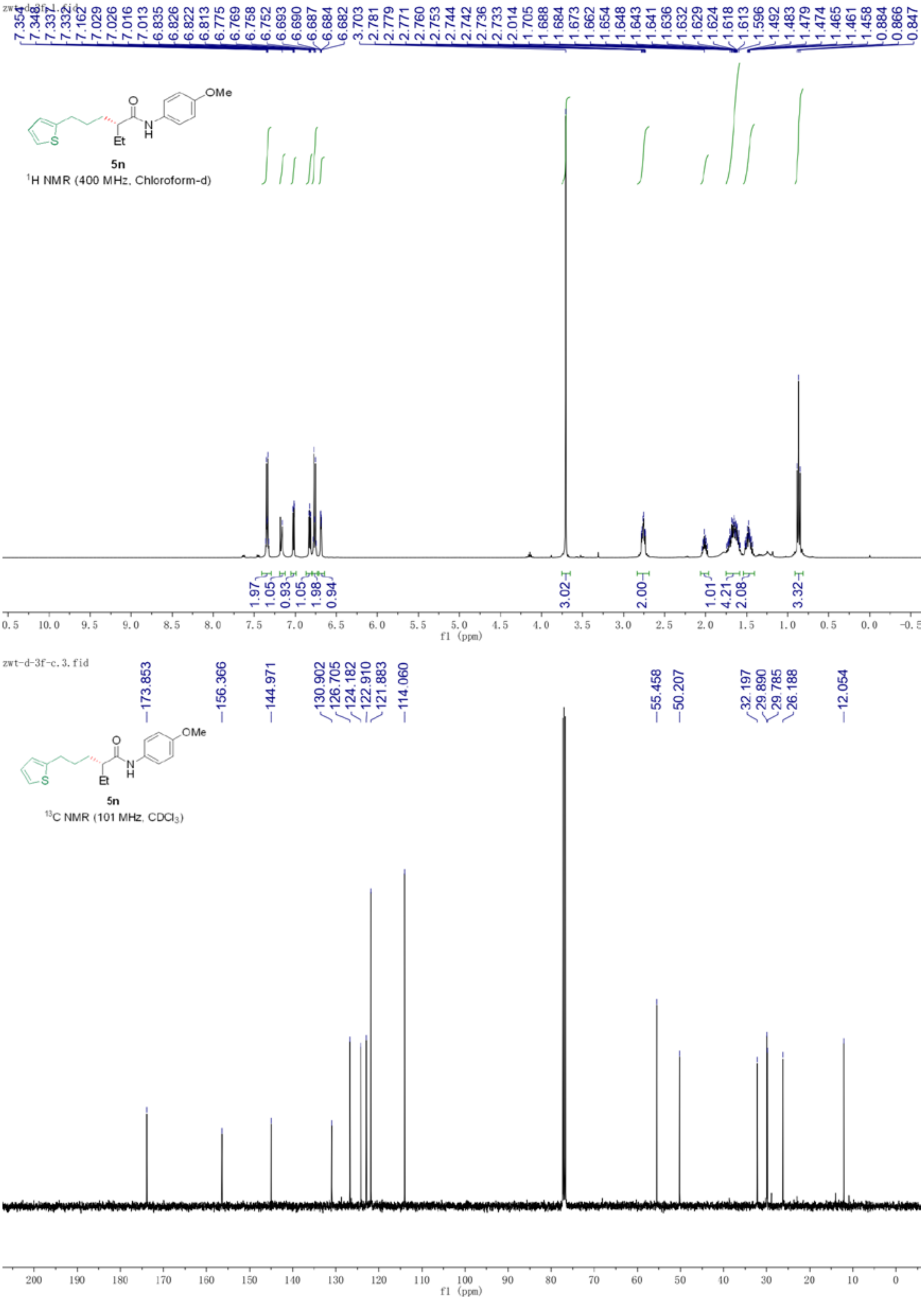

Supplementary Figure 156 ^{1}H-NMR (400 Mz, $CHCl_3$, 25 °C) and ^{13}C-NMR (101 MHz, $CHCl_3$, 25 °C) spectra of **5n**

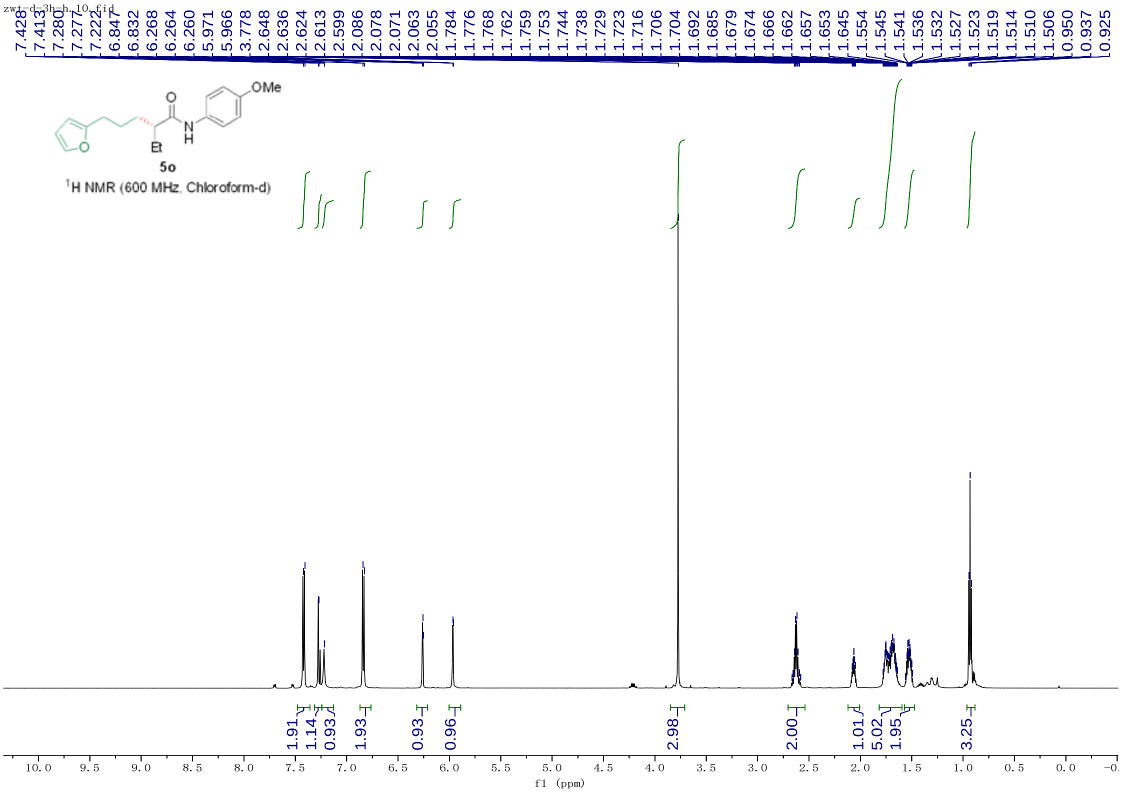

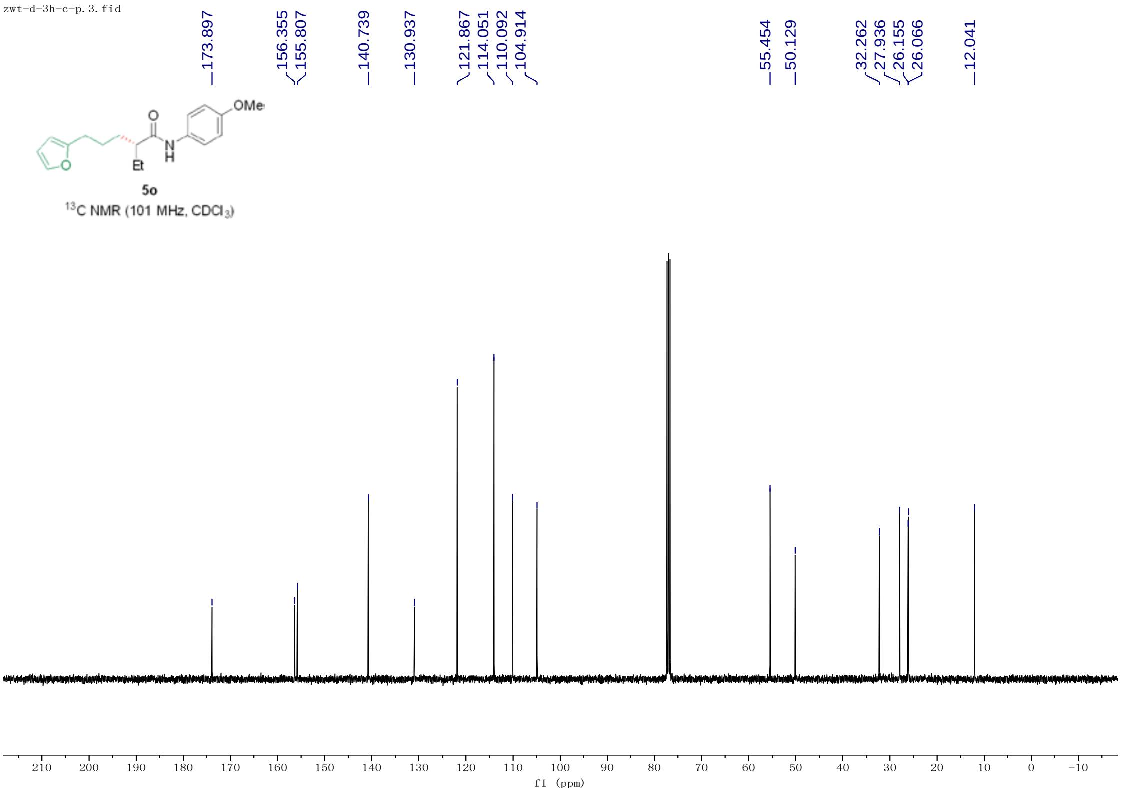

Supplementary Figure 157 ^{1}H-NMR (600 Mz, $CHCl_3$, 25 °C) and ^{13}C-NMR (101 MHz, $CHCl_3$, 25 °C) spectra of **5o**

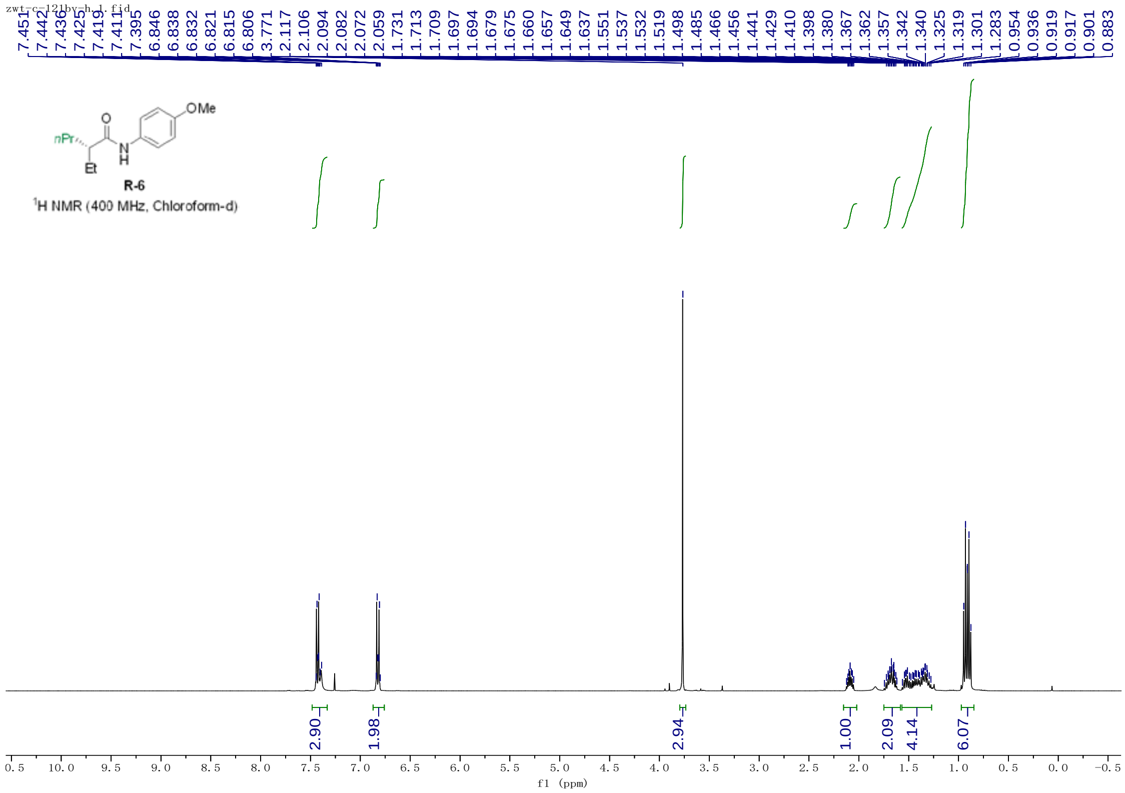

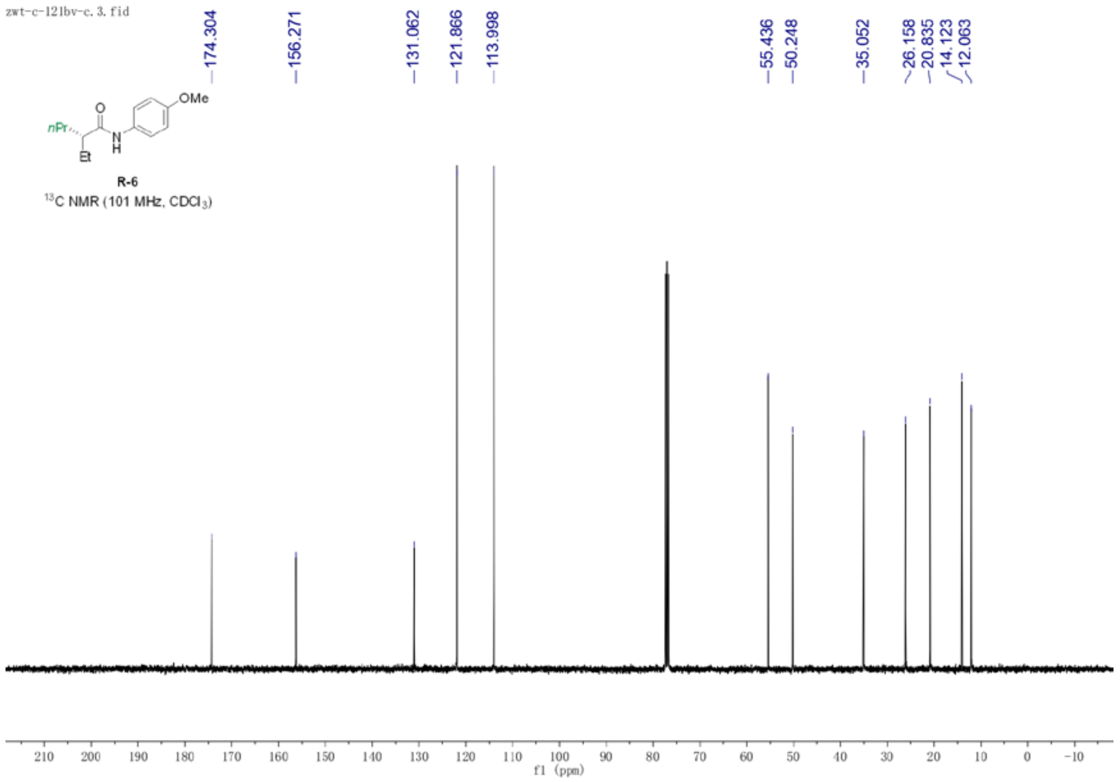

Supplementary Figure 158 ^{1}H-NMR (400 Mz, $CHCl_3$, 25 °C) and ^{13}C-NMR (101 MHz, $CHCl_3$, 25 °C) spectra of ***R*-6**

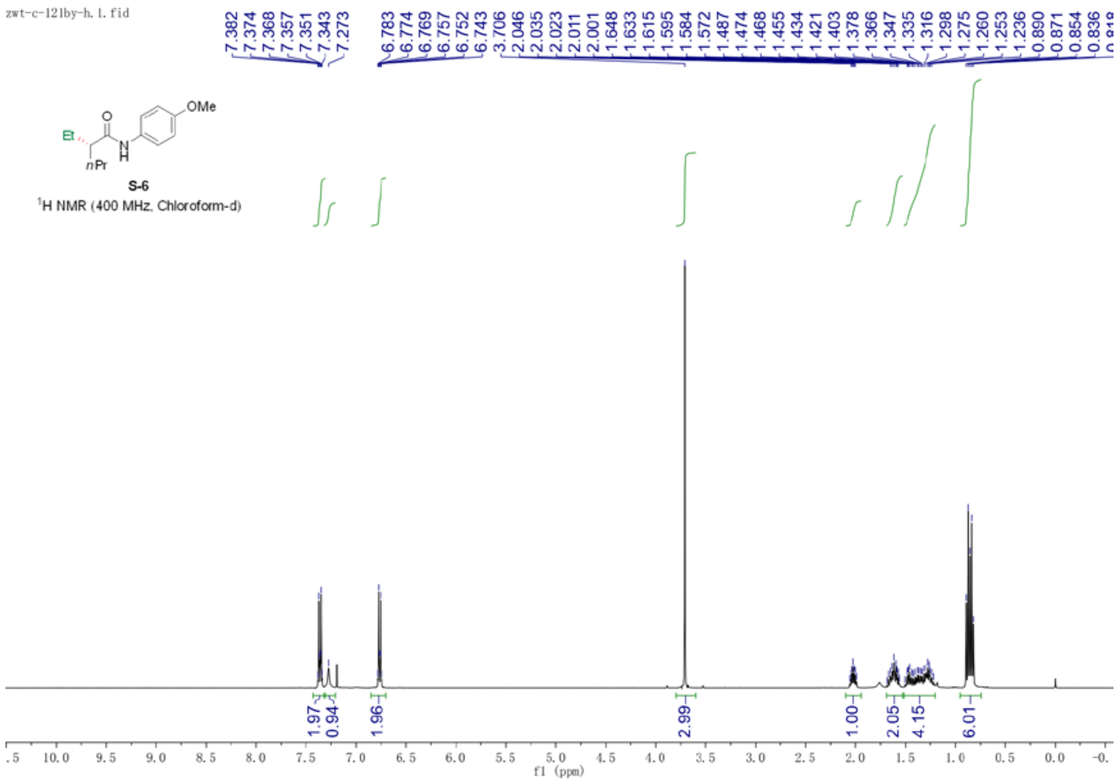

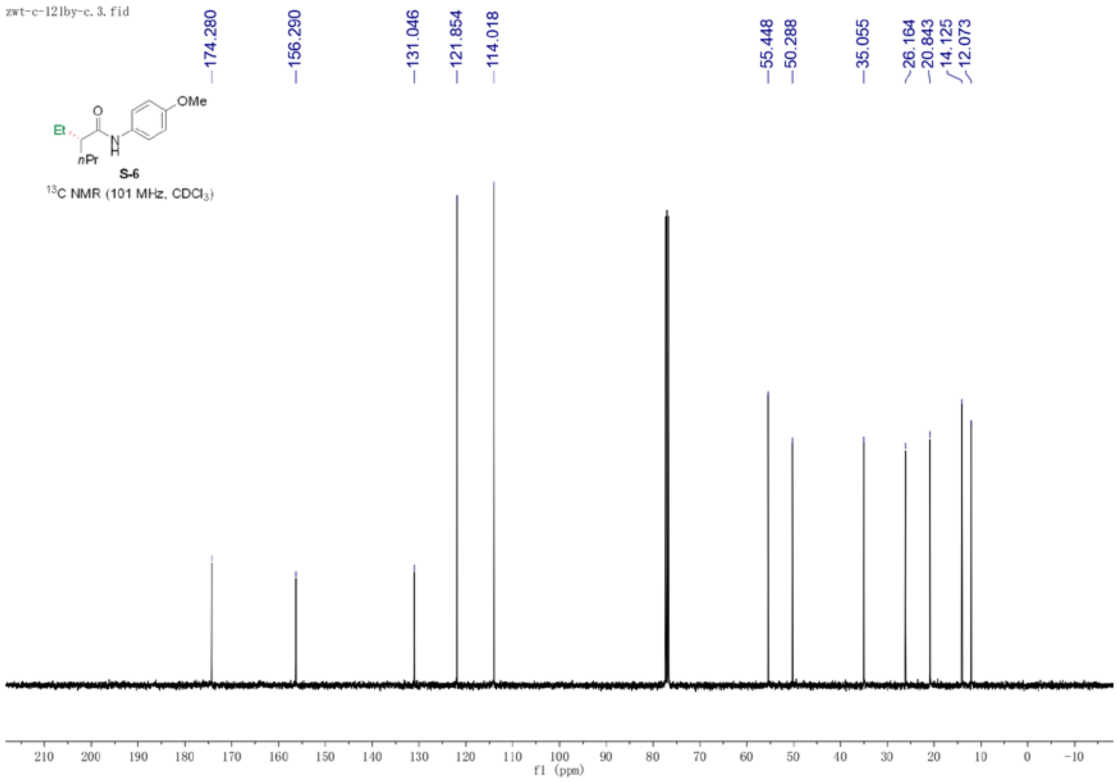

Supplementary Figure 159 ^{1}H-NMR (400 Mz, $CHCl_3$, 25 °C) and ^{13}C-NMR (101 MHz, $CHCl_3$, 25 °C) spectra of ***S*-6**

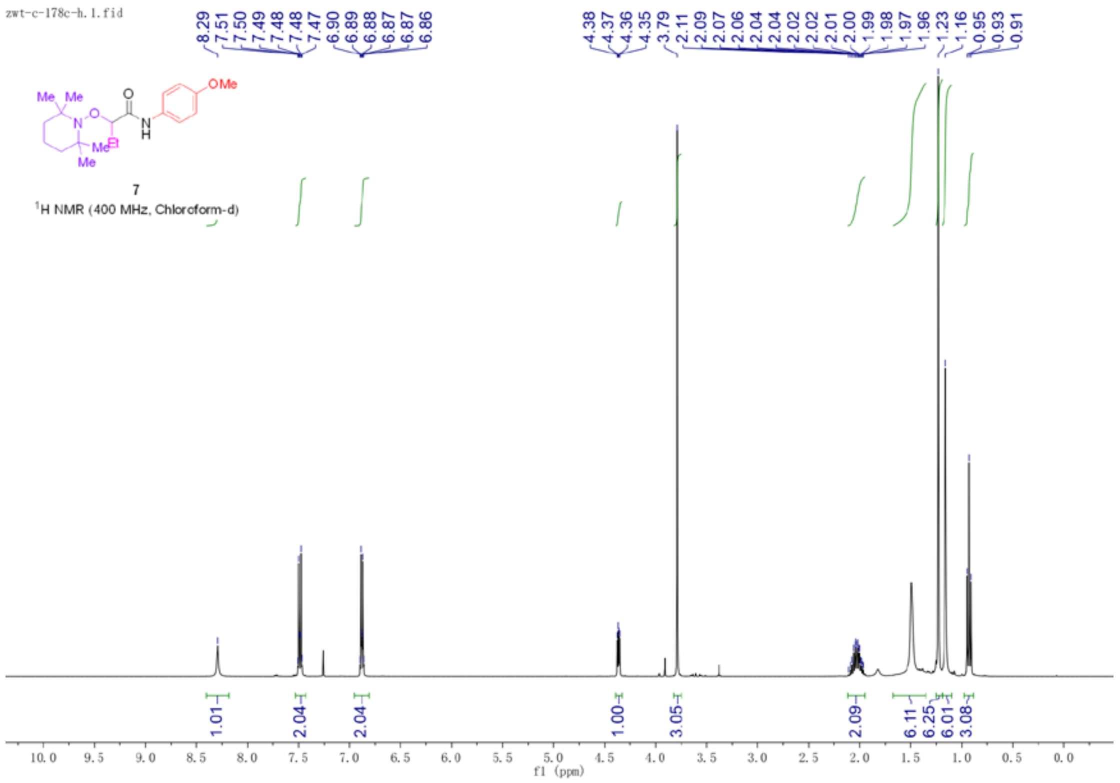

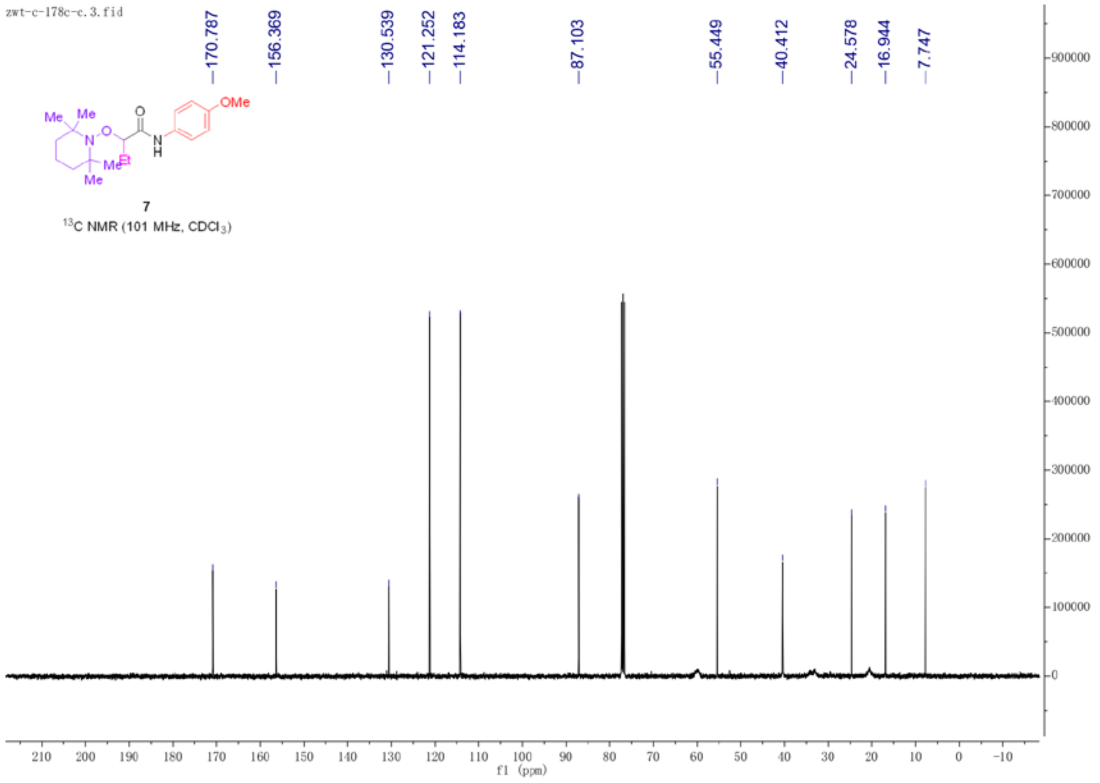

Supplementary Figure 160 ^{1}H-NMR (400 Mz, $CHCl_3$, 25 °C) and ^{13}C-NMR (101 MHz, $CHCl_3$, 25 °C) spectra of **7**

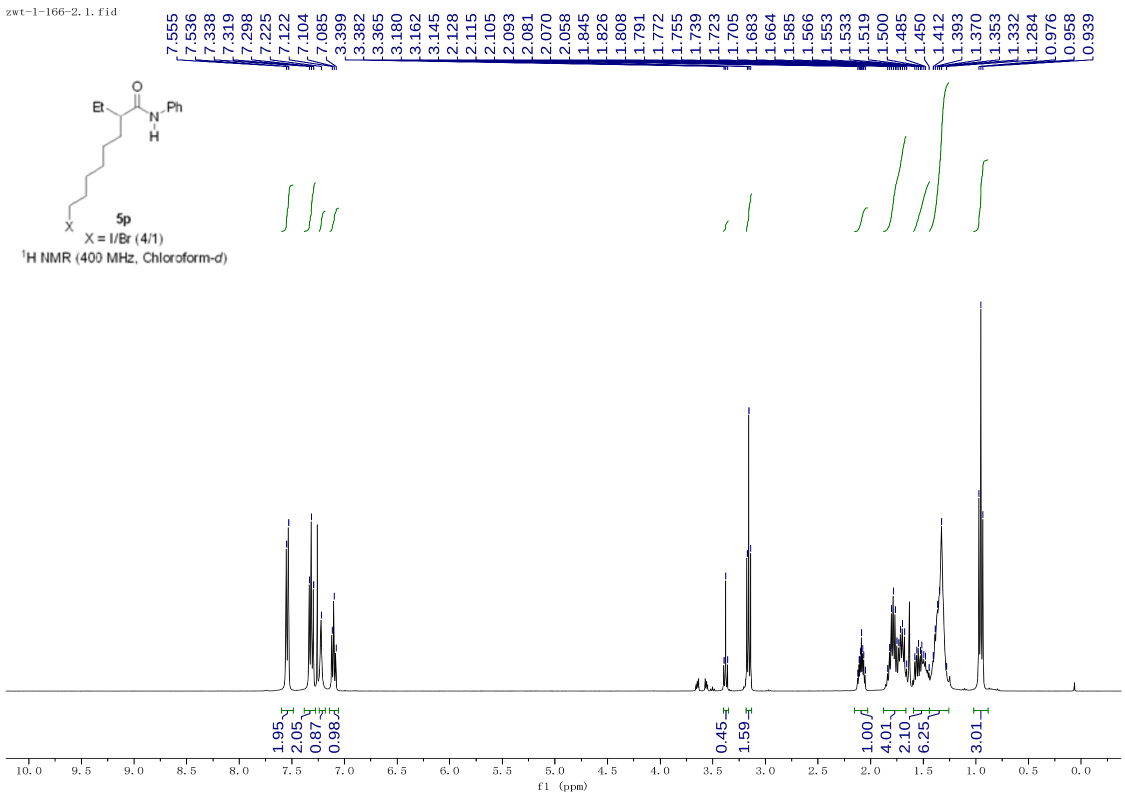

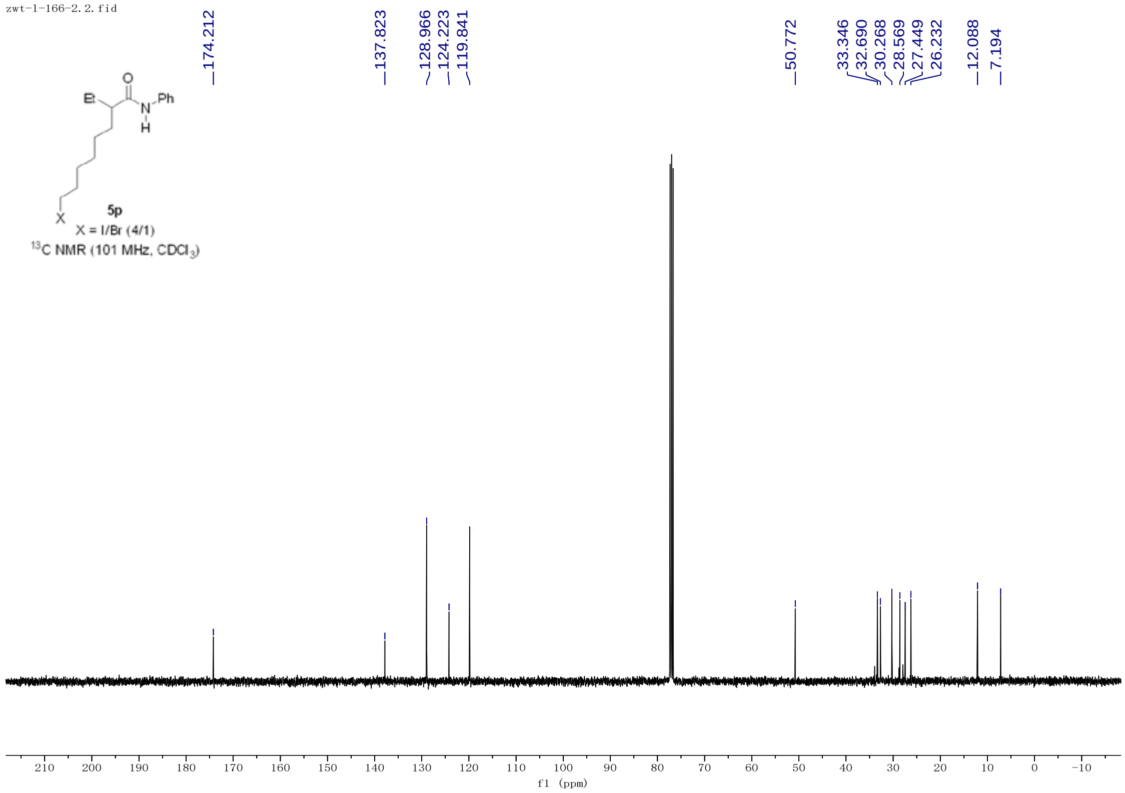

Supplementary Figure 161 ^{1}H-NMR (400 Mz, $CHCl_3$, 25 °C) and ^{13}C-NMR (101 MHz, $CHCl_3$, 25 °C) spectra of **5p**

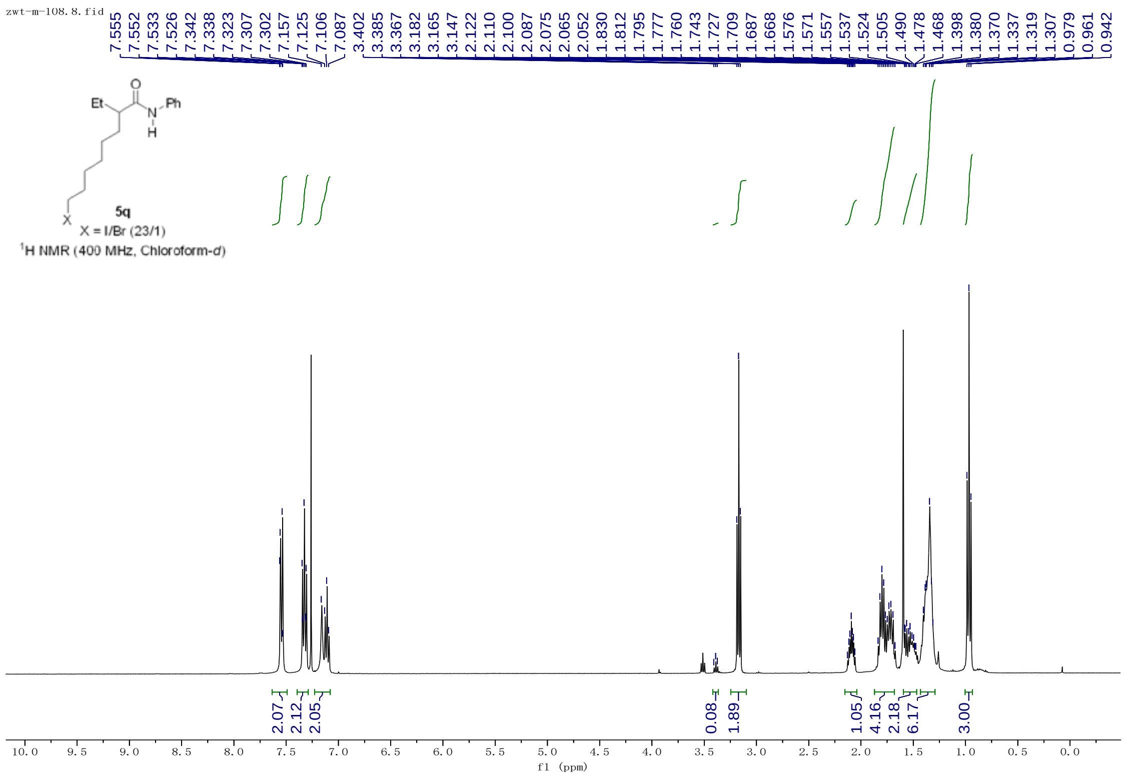

Supplementary Figure 162 ^{1}H-NMR (400 Mz, $CHCl_3$, 25 ℃) spectra of **5q**

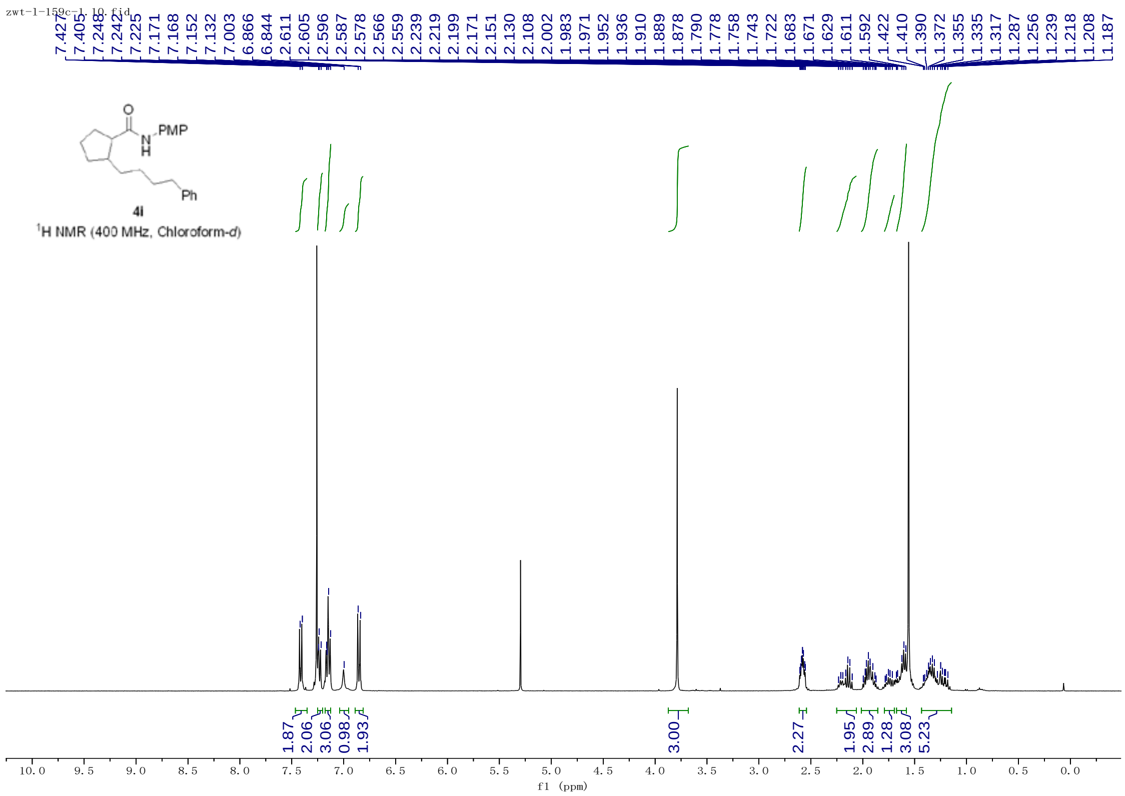

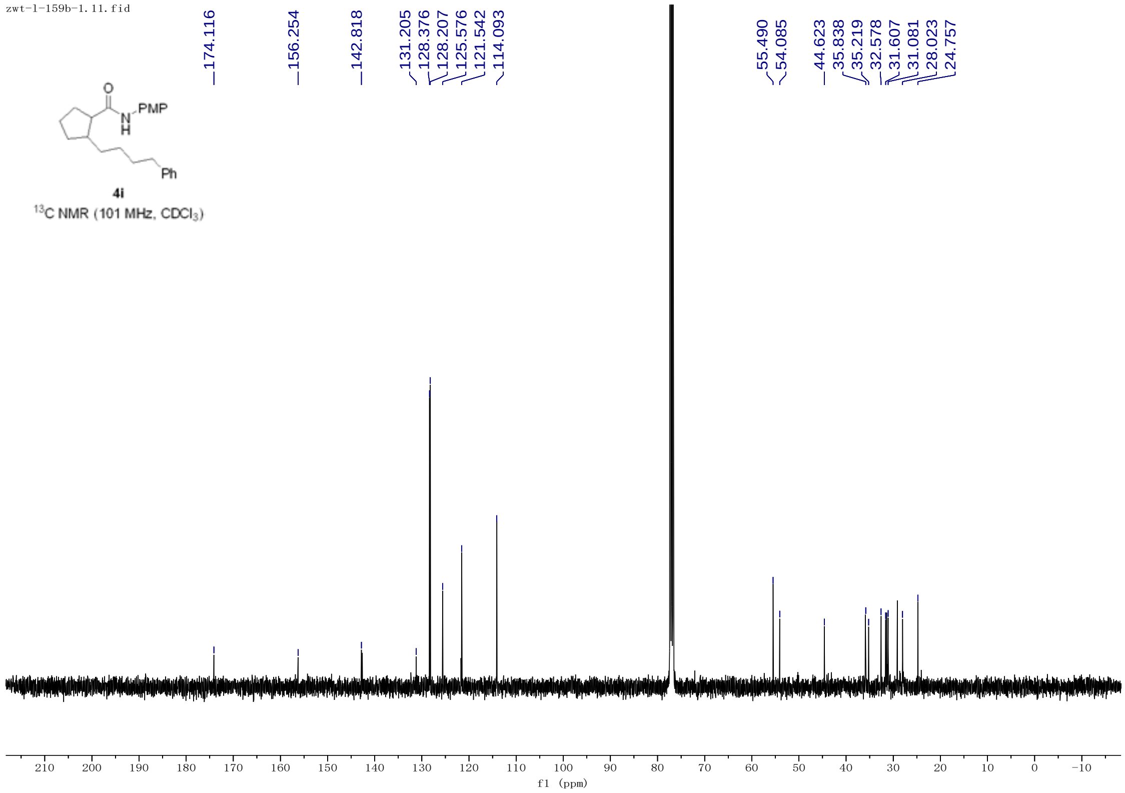

Supplementary Figure 163 ^{1}H-NMR (400 Mz, $CHCl_3$, 25 °C) and ^{13}C-NMR (101 MHz, $CHCl_3$, 25 °C) spectra of **4i**

2.8 X-Ray Diffraction Data of 4f and 7

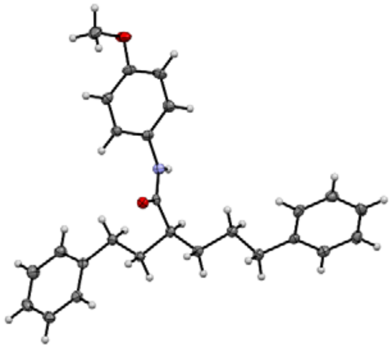

Supplementary Figure 164 X-ray diffraction data of **4f** (CCDC 2089117)

Supplementary Table 14. Crystal data and structure refinement for 4f

Identification code	cxy3239_0m
Empirical formula	$C_{26}H_{29}NO_2$
Formula weight	387.50
Temperature/K	100.0
Crystal system	monoclinic
Space group	$P2_1$
a/Å	10.7559(3)
b/Å	8.4639(2)
c/Å	11.6958(3)
α/°	90
β/°	90.4940(10)
γ/°	90
Volume/Å^3	1064.71(5)
Z	2
ρ_{calc}g/cm^3	1.209
μ/mm^{-1}	0.589
F(000)	416.0

Crystal size/mm^3	0.32 × 0.28 × 0.26
Radiation	CuKα (λ = 1.54178)
2Θ range for data collection/°	7.558 to 136.574
Index ranges	$-12 \leq h \leq 12$, $-10 \leq k \leq 10$, $-13 \leq l \leq 14$
Reflections collected	18892
Independent reflections	3881 [R_{int} = 0.0259, R_{sigma} = 0.0194]
Data/restraints/parameters	3881/1/263
Goodness-of-fit on F^2	0.928
Final R indexes [I>=2σ (I)]	R_1 = 0.0256, wR_2 = 0.0673
Final R indexes [all data]	R_1 = 0.0258, wR_2 = 0.0674
Largest diff. peak/hole / e $Å^{-3}$	0.11/-0.18
Flack parameter	-0.01(4)

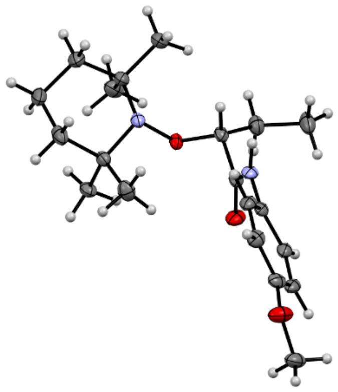

Supplementary Figure 165 X-ray diffraction data of **7** (CCDC 2239323)

Supplementary Table 15. Crystal data and structure refinement for 7

Identification code	ZWTC178C_0ma
Empirical formula	$C_{20}H_{32}N_2O_3$
Formula weight	348.47
Temperature/K	100.0
Crystal system	monoclinic
Space group	Cc
a/Å	10.9356(6)

b/Å	22.2642(12)
c/Å	9.7323(4)
α/°	90
β/°	123.892(2)
γ/°	90
Volume/Å^3	1966.94(18)
Z	4
ρ_{calc}g/cm^3	1.177
μ/mm^{-1}	0.626
F(000)	760.0
Crystal size/mm^3	0.03 × 0.02 × 0.02
Radiation	CuKα (λ = 1.54178)
2Θ range for data collection/°	15.45 to 144.426
Index ranges	-10 ⩽ h ⩽ 13, -27 ⩽ k ⩽ 27, -12 ⩽ l ⩽ 11
Reflections collected	18654
Independent reflections	3503 [R_{in} = 0.0411, R_{sigma} = 0.0328]
Data/restraints/parameters	3503/2/232
Goodness-of-fit on F^2	1.040
Final R indexes [I>=2σ (I)]	R_1 = 0.0276, wR_2 = 0.0723
Final R indexes [all data]	R_1 = 0.0281, wR_2 = 0.0727
Largest diff. peak/hole / e Å^{-3}	0.18/-0.12
Flack parameter	0.03(6)

3. Supplementary References

1. Amador, A. G., Sherbrook, E. M. & Yoon, T. P. Enantioselective Photocatalytic [3 + 2] Cycloadditions of Aryl Cyclopropyl Ketones. *J. Am. Chem. Soc.* **138**, 4722−4725 (2016).
2. Zhu, Y.-Y., Cui, C., Li, N., Wang, B.-W., Wang, Z.-M. & Gao, S. Constructing a Series of Azide-Bridged Cu^{II} Magnetic Low-Dimensional Coordination Polymers by using PyboxLigands. *Eur. J. Inorg. Chem.* **17**, 3101–3111 (2013).
3. Espelt, L. R., McPherson, I. S., Wiensch, E. M. & Yoon, T. P. Enantioselective Conjugate Additions of α-Amino Radicals via Cooperative Photoredox and Lewis Acid Catalysis. *J. Am. Chem. Soc.* **137**, 2452−2455 (2015).
4. Poh, J.-S., Makai, S., Keutz, T., Tran, D. N., Battilocchio, C., Pasau, P. & Ley, S. V. Rapid Asymmetric Synthesis of Disubstituted Allenes by Coupling of Flow-Generated Diazo Compounds and Propargylated Amines. *Angew. Chem. Int. Ed.* **56**, 1864 –1868 (2017).
5. Ginotra, S. K. & Singh,V. K. Enantioselective Oxidation of Olefins Catalyzed by Chiral Copper Bis(oxazolinyl)pyridine Complexes: A Reassessment. *Tetrahedron* **62**, 3573–3581 (2006).
6. Mittapalli, R. R., Guesné, S. J. J., Parker, R. J., Klooster, W. T., Coles, S. J., Skidmore, J., & Dobbs, A. P. The Asymmetric Aza-silyl-Prins Reaction: Synthesis of Enantiopure Piperidines. *Org. Lett.* **21**, 350–355(**2019**).
7. Tsubogo, T., Kano, Y., Ikemoto, K., Yamashita, Y. & Kobayashi, S. Synthesis of Optically Active, Unnatural A-Substituted Glutamic Acid Derivatives by a Chiral Calcium-Catalyzed 1,4-Addition Reaction. *Tetrahedron: Asymmetry* **21**, 1221–1225 (2010).
8. Fischer, C. & Fu, G. C. Asymmetric Nickel-Catalyzed Negishi Cross-Couplings of Secondary α-Bromo Amides with Organozinc Reagents. *J. Am. Chem. Soc.* **127**, 4594-4595 (2005).
9. Schmidt, J., Choi, J., Liu, A. T., Slusarczyk, M. & Fu, G. C. A General, Modular

Method for the Catalytic Asymmetric Synthesis of Alkylboronate Esters. *Science* **354**, 1265–1269 (2016).
